# Supplementary material for: Dissolution of spiral wave’s core using cardiac optogenetics
Source: PLoS Comput Biol. 2023 Dec 7;19(12):e1011660. doi: 10.1371/journal.pcbi.1011660 (PMC10729946; doi:10.1371/journal.pcbi.1011660)
Supplement: Attachment — Submitted filename: Response_Letter.pdf [file pcbi.1011660.s006.pdf]

S. Hussaini *et al.*
Max Planck Institute for
Dynamics and Self-Organization
Göttingen, Germany
stefan.luther@ds.mpg.de

PLOS Computational Biology

October 26, 2023

Dear members of the editorial board and reviewers,

Thank you for your correspondence regarding our manuscript with original title "A mechanism underlying slow termination of arrhythmia" and the enclosed reviewers' reports. We would like to thank the reviewers for their consideration of this work and their valuable assessment of the manuscript. The reviewers' comments inspired us to change the title of the manuscript to "Dissolution of Spiral Wave's Core Using Cardiac Optogenetics".

In the following, we address the reviewer comments, outlining what we have done to incorporate necessary modifications to our manuscript.

Reviewer I

This study by Hussaini et al present a very detailed, well-written and comprehensive combined simulation and experimental study demonstrating a novel mechanism of arrhythmia termination through optical defibrillation. Throughout the comparison between simulations and experiments is strong, which significantly strengthens the study. I have only relatively minor comments.
1. The 2D vs 3D issue is very important. I can understand why the simulations were conducted in 2D, though. The discussion of these issues, however, in the Discussion nicely explains the possible reasons for the discrepancies. In future works, I would urge the authors to consider 3D simulation approaches, along with corresponding models of the decay of the exciting light into the tissue depth. I fear that, whilst this and similar works have shown great promise in 2D simulations and very thin (small mammal) hearts, it remains unclear to me how these effects would translate into a 10mm+ human left ventricle in which transmural excitation will be a significant challenge. I feel that more of a comment in this regard is important to include in the Discussion, in terms of depth-penetration and clinical translation. In particular, as well as the depth of light penetration issue, the dynamics of 3D scroll waves are more complex, particularly with thicker walls. Please comment on the potential implications of this.

We agree with the reviewer. For this, we added the following paragraph to the discussion section of the main manuscript, lines (333-347):
"From a clinical perspective, it is important to understand the possible mechanisms of arrhythmia termination in thick cardiac tissue (e.g, from the left ventricular wall), which is 3D. Previous studies have shown that depending on the thickness of the tissue, a scroll filament can bend, twist, break up, grow and/or shrink inside the bulk of the tissue (Qu et al. (2000)). Presence of inhomogeneities inside the tissue further add to the complexity of the wave dynamics in that, they allow attachment/detachment of the filaments to/from their locations, as well as promote the formation of 'seed waves' which have the potential to regenerate a full scroll (R. Majumder et al. (2011)). These dynamical behaviours can also occur under the influence of illumination on the surface of cardiac

tissue during an arrhythmia. Depending on the true depth of penetration of the applied light, one can expect the tissue substrate to behave inhomogeneously. In this study, we deliberately chose a simple geometry (2D domain) that sufficiently represented the complex dynamics of a single sheet of mouse ventricular tissue by an ionic complex mathematical model. This 2D domain allowed us to highlight the existence of a possible termination mechanism, core dissolution, that could be overlaid by other possible termination mechanisms of arrhythmia during global surface illumination in a complex 3D domain. Nevertheless, it would be interesting to observe how the proposed mechanism of slow termination of a scroll wave would perform in an anatomically realistic simulation domain. We conjecture that the proposed mechanism of slow termination can then be seen to occur when the fibre orientation is mostly homogeneous, does not show sharp changes and light penetration is sufficiently deep."

2. In the conclusions, it states that in the simulations the main mechanisms of defibrillation was "pushing the arm of the spiral wave to the boundary, leading to its termination". In the 2D square model, this has well-defined artificial barriers, which might lead to an artificially-higher termination rate than could be expected with a more continuous 'wrap-around' structure, like the heart. Please could the authors comment on this?
Thank you for your comment. It is quite true that the realistic geometry of the heart is very different from the 2D artificial simulation domain we used. However, our aim was to choose a simple scenario to better understand the mechanism. Hence the choice of 2D. In the realistic geometry, the only natural inexcitable region inside the heart is the atrio-ventricular boundary (NOTE: this differs from 2D, which has inexcitable boundaries on all four sides). However, the structure of the heart is such that all electrical activity within the ventricles has access to this inexcitable boundary and is obliged to cross it at some point, unless the activity is confined to the apex. Furthermore, in 3D, arrhythmia termination occurs not only by collision with the atrio-ventricular border, but also by collision of the rotating scroll filaments. This collision occurs more regularly in an enveloping structure (as in the real heart) than in a flattened structure (2D). Thus, the arrhythmia termination rate is reduced in 3D compared with 2D, but the reduction is not by orders of magnitude.

3. Related to this, it does not appear that this major mechanism (pushing the arm to the boundary) was witnessed in the experimental mouse data. Please could you clarify? Here, it seems that the main mechanism was through prolongation of the APD. Was this mechanism also seen in the simulations?
That is correct, the main mechanism for arrhythmia termination in experimental observation is prolongation of APD. In the numerical simulation, we also observed prolongation of APD. However, the core dissolution mechanism also interacts and, in contrast to the experiments, plays the dominant role in termination of arrhythmia in competition with APD prolongation.

4. In the 2D model, conduction appears isotropic. Please comment on the potential effects of tissue anisotropy.
In our 2D simulations, we used isotropic conduction. This ensured the propagation of a circular wave from a point excitation applied to any part of the simulation domain. It also ensured the appearance of a circular core of a spiral wave, which dissolved in an expanding circle during the termination process. If, however, anisotropy is included, a point excitation would lead to elliptic propagation of excitation waves; a spiral wave will have an elliptic on more complex core structure (depending on the anisotropy) and the core would dissolve with an elliptic shape.

5. It is not clear whether the arrhythmias induced are VT-like or VF-like(?) Fig 1 implies that these were single spiral waves, and thus more like rapid VT rather than the more chaotic fibrillation patterns. The supplemental movies also seem to show far more monomorphic VT-like patterns. In the experiments, only frequencies are quoted. In real defibrillation cases, VF would be the primary arrhythmia which would be treated, and which would have significantly more 'chaotic' wavefront patterns than analysed here. I believe that this might significantly implicate some of the mechanisms

you present here (for example, the 'pushing of the spiral wave'). Please could the authors comment on this important issue.
Thanks to the reviewer for drawing attention to this important point. What we have reported in this paper about the termination mechanism requires a certain periodicity and sustainability of the dynamics to be characterized. However, in a chaotic state where spiral waves are constantly appearing and disappearing, such a characterization could be challenging and bring complications. We believe that for a deeper understanding of such complex dynamics, it would be beneficial to take a look at a simpler dynamics in which a spiral wave propagates.

Reviewer II

This is a manuscript from Stephan Luther's lab, one of the world's leading experts in cardiac dynamics. The lab is renowned for using sophisticated modeling and experimental approaches to explore new low-energy defibrillator strategies. In this study, they conducted in silico and ex vivo investigations to dissect the basic mechanisms of single rotor termination during sub- and super-threshold optogenetic manipulations. The topic is very timely, and the experiments and modeling appear to be well-performed. I have just a few minor comments to improve the solidity, readability, and general interest of the manuscript:
1. Although generating more complex dynamics in a mouse heart is very challenging (and likely physically impossible), multiple coexisting rotors could be easily (?) generated in silico. Therefore, the authors could explore if the core-expansion mechanism represents a predominant termination mechanism in this more realistic scenario as well.
Thank you very much for your comment. As the reviewer mentioned, it is very challenging to create multiple rotors in the mouse heart because it is small and therefore does not provide enough space for multiple spiral waves to propagate. In this study, we deliberately chose a simple geometry (2D domain) that sufficiently represented the complex dynamics of a single sheet of mouse ventricular tissue by an ionic complex mathematical model. This 2D domain allowed us to highlight the existence of a possible termination mechanism, core dissolution, that could be overlaid by other possible termination mechanisms of arrhythmia during global surface illumination in a complex 3D domain.

2. Considering that the VSD used in this experiment should absorb at 470nm, leading to an increase in the fluorescent baseline, I expect that authors have to re-normalize the "voltage" map during optogenetic illumination. If this is the case, it should be stated in the text.
That is correct. We have included the following statements in the methods section of the manuscript, lines(127-130):
We observed the crosstalk between the blue stimulation light and the red excitation light leads to an increase in the fluorescence baseline. Therefore, to counteract this signal artifact, the optical signal is re-normalized by division to the recorded background signal.

3. Figure 2D and Figure 2B in the text seem to refer to incorrect panels.
Thanks for the comment. We have corrected it. Please look at the lines (184 and 187) in the manuscript.

4. The use of two different LI scales in Figure 5 can reduce readability. Consider homogenizing the scale to improve clarity.
Thanks for the comment.

5. In addition to references 17 - 19, two additional works should be cited in relation to lighting patterns illumination: doi.org/10.1038/srep35628; doi.org/10.1113/JP276283.
Thank you very much for the comment. We have included these two references in the manuscript, line (54) and page (16).

Your Sincerely,

S. Hussaini *et al.*
