# Supplementary material for: Dissolution of spiral wave’s core using cardiac optogenetics
Source: PLoS Comput Biol. 2023 Dec 7;19(12):e1011660. doi: 10.1371/journal.pcbi.1011660 (PMC10729946; doi:10.1371/journal.pcbi.1011660)
Supplement: S1 Code — (ZIP) [file pcbi.1011660.s001.zip › S1_Simulation-Code.zip!/SIMULATION/bondarenkomodel/model2012.html]

Generated by Cython 0.20.1post0 (Debian 0.20.1+git90-g0e6e38e-1ubuntu2) on Wed Apr 4 16:53:33 2018

Raw output: model.c

```
 1: #!/usr/bin/env python3
```

```
  __pyx_t_1 = PyDict_New(); if (unlikely(!__pyx_t_1)) {__pyx_filename = __pyx_f[0]; __pyx_lineno = 1; __pyx_clineno = __LINE__; goto __pyx_L1_error;}
  __Pyx_GOTREF(__pyx_t_1);
  if (PyDict_SetItem(__pyx_d, __pyx_n_s_test, __pyx_t_1) < 0) {__pyx_filename = __pyx_f[0]; __pyx_lineno = 1; __pyx_clineno = __LINE__; goto __pyx_L1_error;}
  __Pyx_DECREF(__pyx_t_1); __pyx_t_1 = 0;
```

```
 2: #cython: language_level=3, cdivision=True, wraparound=False, boundscheck=False
```

```
 3: # -*- coding: utf-8 -*-
```

```
 4: """
```

```
 5: Created on Tue Mar 27 15:46:54 2018
```

```
 6:
```

```
 7: @author: shussai
```

```
 8: """
```

```
 9: cimport cython
```

```
 10: cimport numpy as npc
```

```
 11: import numpy as np
```

```
  __pyx_t_1 = __Pyx_Import(__pyx_n_s_numpy, 0, 0); if (unlikely(!__pyx_t_1)) {__pyx_filename = __pyx_f[0]; __pyx_lineno = 11; __pyx_clineno = __LINE__; goto __pyx_L1_error;}
  __Pyx_GOTREF(__pyx_t_1);
  if (PyDict_SetItem(__pyx_d, __pyx_n_s_np, __pyx_t_1) < 0) {__pyx_filename = __pyx_f[0]; __pyx_lineno = 11; __pyx_clineno = __LINE__; goto __pyx_L1_error;}
  __Pyx_DECREF(__pyx_t_1); __pyx_t_1 = 0;
```

```
 12:
```

```
 13: cdef extern from "math.h":\
```

```
 14:
```

```
 15:     cdef double exp(double x)
```

```
 16:     cdef double log(double x)
```

```
 17:
```

```
 18:
```

```
 19:
```

```
 20: cdef class Bondarenko:
```

```
struct __pyx_obj_5model_Bondarenko {
  PyObject_HEAD
  struct __pyx_vtabstruct_5model_Bondarenko *__pyx_vtab;
  double A_cap;
  double V_myo;
  double V_JSR;
  double V_NSR;
  double V_ss;
  double K_0;
  double Na_0;
  double Ca_0;
  double v_1;
  double v_2;
  double v_3;
  double k_mup;
  double t_tr;
  double t_xfer;
  double k_p_a;
  double k_n_a;
  double k_p_b;
  double k_n_b;
  double k_p_c;
  double k_n_c;
  double n;
  double m;
  double G_CaL;
  double E_CaL;
  double k_pcmax;
  double k_pchalf;
  double k_pcb;
  double I_CaLmax;
  double LTRPN_tot;
  double HTRPN_tot;
  double k_p_htrpn;
  double k_n_htrpn;
  double k_p_ltrpn;
  double k_n_ltrpn;
  double CMDN_tot;
  double CSQN_tot;
  double k_CMDN_m;
  double k_CSQN_m;
  double C_m;
  double F;
  double T;
  double R;
  double k_NaCa;
  double k_mNa;
  double k_mCa;
  double k_sat;
  double miu;
  double I_maxNaK;
  double K_mNai;
  double K_mKo;
  double I_maxpCa;
  double k_mpCa;
  double G_Cab;
  double G_Na;
  double G_Nab;
  double G_Ktof;
  double G_Ks;
  double G_Ktos;
  double G_Kur;
  double G_Kss;
  double G_Kr;
  double k_f;
  double k_b;
  double G_ClCa;
  double K_mCl;
  double E_Cl;
  double Laplas_c;
  double Laplas_r;
  double V_ChR2;
  double i_Kss;
  double h;
  double dy;
  double dx;
  double t;
  __Pyx_memviewslice V;
  __Pyx_memviewslice Ca2_i;
  __Pyx_memviewslice Ca2_ss;
  __Pyx_memviewslice Ca2_JSR;
  __Pyx_memviewslice Ca2_NSR;
  __Pyx_memviewslice LTRPNCa;
  __Pyx_memviewslice HTRPNCa;
  __Pyx_memviewslice O;
  __Pyx_memviewslice C1;
  __Pyx_memviewslice C2;
  __Pyx_memviewslice C3;
  __Pyx_memviewslice C4;
  __Pyx_memviewslice I1;
  __Pyx_memviewslice I2;
  __Pyx_memviewslice I3;
  __Pyx_memviewslice Pc1;
  __Pyx_memviewslice Pc2;
  __Pyx_memviewslice Po1;
  __Pyx_memviewslice Po2;
  __Pyx_memviewslice P_RyR;
  __Pyx_memviewslice C_Na3;
  __Pyx_memviewslice C_Na2;
  __Pyx_memviewslice C_Na1;
  __Pyx_memviewslice O_Na;
  __Pyx_memviewslice IF_Na;
  __Pyx_memviewslice I1_Na;
  __Pyx_memviewslice I2_Na;
  __Pyx_memviewslice IC_Na2;
  __Pyx_memviewslice IC_Na3;
  __Pyx_memviewslice Na_i;
  __Pyx_memviewslice K_i;
  __Pyx_memviewslice a_tof;
  __Pyx_memviewslice i_tof;
  __Pyx_memviewslice n_Ks;
  __Pyx_memviewslice a_tos;
  __Pyx_memviewslice i_tos;
  __Pyx_memviewslice a_ur;
  __Pyx_memviewslice i_ur;
  __Pyx_memviewslice a_Kss;
  __Pyx_memviewslice C_K0;
  __Pyx_memviewslice C_K1;
  __Pyx_memviewslice C_K2;
  __Pyx_memviewslice O_K;
  __Pyx_memviewslice I_K;
};
/* … */
struct __pyx_vtabstruct_5model_Bondarenko {
  PyObject *(*local_step)(struct __pyx_obj_5model_Bondarenko *, int __pyx_skip_dispatch);
  double (*f_V)(struct __pyx_obj_5model_Bondarenko *, double, double, double, double, double, double, double, double, double, double, double, double, double, double, double, double);
  double (*f_Ca2_i)(struct __pyx_obj_5model_Bondarenko *, double, double, double, double, double, double, double, double);
  double (*f_Ca2_ss)(struct __pyx_obj_5model_Bondarenko *, double, double, double, double);
  double (*f_Ca2_JSR)(struct __pyx_obj_5model_Bondarenko *, double, double, double);
  double (*f_Ca2_NSR)(struct __pyx_obj_5model_Bondarenko *, double, double, double);
  double (*f_P_RyR)(struct __pyx_obj_5model_Bondarenko *, double, double, double);
  double (*f_LTRPNCa)(struct __pyx_obj_5model_Bondarenko *, double, double);
  double (*f_HTRPNCa)(struct __pyx_obj_5model_Bondarenko *, double, double);
  double (*f_Po1)(struct __pyx_obj_5model_Bondarenko *, double, double, double, double, double);
  double (*f_Po2)(struct __pyx_obj_5model_Bondarenko *, double, double, double);
  double (*f_Pc2)(struct __pyx_obj_5model_Bondarenko *, double, double);
  double (*f_O)(struct __pyx_obj_5model_Bondarenko *, double, double, double, double, double, double, double, double);
  double (*f_C2)(struct __pyx_obj_5model_Bondarenko *, double, double, double, double, double);
  double (*f_C3)(struct __pyx_obj_5model_Bondarenko *, double, double, double, double, double);
  double (*f_C4)(struct __pyx_obj_5model_Bondarenko *, double, double, double, double, double, double, double, double, double, double);
  double (*f_I1)(struct __pyx_obj_5model_Bondarenko *, double, double, double, double, double, double, double, double);
  double (*f_I2)(struct __pyx_obj_5model_Bondarenko *, double, double, double, double, double, double, double, double);
  double (*f_I3)(struct __pyx_obj_5model_Bondarenko *, double, double, double, double, double, double, double, double);
  double (*f_Na_i)(struct __pyx_obj_5model_Bondarenko *, double, double, double, double);
  double (*f_C_Na2)(struct __pyx_obj_5model_Bondarenko *, double, double, double, double, double, double, double, double, double, double);
  double (*f_C_Na1)(struct __pyx_obj_5model_Bondarenko *, double, double, double, double, double, double, double, double, double, double);
  double (*f_O_Na)(struct __pyx_obj_5model_Bondarenko *, double, double, double, double, double, double, double);
  double (*f_IF_Na)(struct __pyx_obj_5model_Bondarenko *, double, double, double, double, double, double, double, double, double, double, double, double, double);
  double (*f_I1_Na)(struct __pyx_obj_5model_Bondarenko *, double, double, double, double, double, double, double);
  double (*f_I2_Na)(struct __pyx_obj_5model_Bondarenko *, double, double, double, double);
  double (*f_IC_Na2)(struct __pyx_obj_5model_Bondarenko *, double, double, double, double, double, double, double, double, double, double);
  double (*f_IC_Na3)(struct __pyx_obj_5model_Bondarenko *, double, double, double, double, double, double, double);
  double (*f_K_i)(struct __pyx_obj_5model_Bondarenko *, double, double, double, double, double, double, double, double);
  double (*f_a_tof)(struct __pyx_obj_5model_Bondarenko *, double, double, double);
  double (*f_i_tof)(struct __pyx_obj_5model_Bondarenko *, double, double, double);
  double (*f_a_tos)(struct __pyx_obj_5model_Bondarenko *, double, double, double);
  double (*f_i_tos)(struct __pyx_obj_5model_Bondarenko *, double, double, double);
  double (*f_n_Ks)(struct __pyx_obj_5model_Bondarenko *, double, double, double);
  double (*f_a_ur)(struct __pyx_obj_5model_Bondarenko *, double, double, double);
  double (*f_i_ur)(struct __pyx_obj_5model_Bondarenko *, double, double, double);
  double (*f_a_Kss)(struct __pyx_obj_5model_Bondarenko *, double, double, double);
  double (*f_C_K1)(struct __pyx_obj_5model_Bondarenko *, double, double, double, double, double);
  double (*f_C_K2)(struct __pyx_obj_5model_Bondarenko *, double, double, double, double, double);
  double (*f_O_K)(struct __pyx_obj_5model_Bondarenko *, double, double, double, double, double, double, double);
  double (*f_I_K)(struct __pyx_obj_5model_Bondarenko *, double, double, double, double);
};
static struct __pyx_vtabstruct_5model_Bondarenko *__pyx_vtabptr_5model_Bondarenko;
```

```
 21:     cdef public double A_cap
```

```
/* Python wrapper */
static PyObject *__pyx_pw_5model_10Bondarenko_5A_cap_1__get__(PyObject *__pyx_v_self); /*proto*/
static PyObject *__pyx_pw_5model_10Bondarenko_5A_cap_1__get__(PyObject *__pyx_v_self) {
  PyObject *__pyx_r = 0;
  __Pyx_RefNannyDeclarations
  __Pyx_RefNannySetupContext("__get__ (wrapper)", 0);
  __pyx_r = __pyx_pf_5model_10Bondarenko_5A_cap___get__(((struct __pyx_obj_5model_Bondarenko *)__pyx_v_self));

  /* function exit code */
  __Pyx_RefNannyFinishContext();
  return __pyx_r;
}

static PyObject *__pyx_pf_5model_10Bondarenko_5A_cap___get__(struct __pyx_obj_5model_Bondarenko *__pyx_v_self) {
  PyObject *__pyx_r = NULL;
  __Pyx_RefNannyDeclarations
  __Pyx_RefNannySetupContext("__get__", 0);
  __Pyx_XDECREF(__pyx_r);
  __pyx_t_1 = PyFloat_FromDouble(__pyx_v_self->A_cap); if (unlikely(!__pyx_t_1)) {__pyx_filename = __pyx_f[0]; __pyx_lineno = 21; __pyx_clineno = __LINE__; goto __pyx_L1_error;}
  __Pyx_GOTREF(__pyx_t_1);
  __pyx_r = __pyx_t_1;
  __pyx_t_1 = 0;
  goto __pyx_L0;

  /* function exit code */
  __pyx_L1_error:;
  __Pyx_XDECREF(__pyx_t_1);
  __Pyx_AddTraceback("model.Bondarenko.A_cap.__get__", __pyx_clineno, __pyx_lineno, __pyx_filename);
  __pyx_r = NULL;
  __pyx_L0:;
  __Pyx_XGIVEREF(__pyx_r);
  __Pyx_RefNannyFinishContext();
  return __pyx_r;
}

/* Python wrapper */
static int __pyx_pw_5model_10Bondarenko_5A_cap_3__set__(PyObject *__pyx_v_self, PyObject *__pyx_v_value); /*proto*/
static int __pyx_pw_5model_10Bondarenko_5A_cap_3__set__(PyObject *__pyx_v_self, PyObject *__pyx_v_value) {
  int __pyx_r;
  __Pyx_RefNannyDeclarations
  __Pyx_RefNannySetupContext("__set__ (wrapper)", 0);
  __pyx_r = __pyx_pf_5model_10Bondarenko_5A_cap_2__set__(((struct __pyx_obj_5model_Bondarenko *)__pyx_v_self), ((PyObject *)__pyx_v_value));

  /* function exit code */
  __Pyx_RefNannyFinishContext();
  return __pyx_r;
}

static int __pyx_pf_5model_10Bondarenko_5A_cap_2__set__(struct __pyx_obj_5model_Bondarenko *__pyx_v_self, PyObject *__pyx_v_value) {
  int __pyx_r;
  __Pyx_RefNannyDeclarations
  __Pyx_RefNannySetupContext("__set__", 0);
  __pyx_t_1 = __pyx_PyFloat_AsDouble(__pyx_v_value); if (unlikely((__pyx_t_1 == (double)-1) && PyErr_Occurred())) {__pyx_filename = __pyx_f[0]; __pyx_lineno = 21; __pyx_clineno = __LINE__; goto __pyx_L1_error;}
  __pyx_v_self->A_cap = __pyx_t_1;

  /* function exit code */
  __pyx_r = 0;
  goto __pyx_L0;
  __pyx_L1_error:;
  __Pyx_AddTraceback("model.Bondarenko.A_cap.__set__", __pyx_clineno, __pyx_lineno, __pyx_filename);
  __pyx_r = -1;
  __pyx_L0:;
  __Pyx_RefNannyFinishContext();
  return __pyx_r;
}
```

```
 22:     cdef public double V_myo
```

```
/* Python wrapper */
static PyObject *__pyx_pw_5model_10Bondarenko_5V_myo_1__get__(PyObject *__pyx_v_self); /*proto*/
static PyObject *__pyx_pw_5model_10Bondarenko_5V_myo_1__get__(PyObject *__pyx_v_self) {
  PyObject *__pyx_r = 0;
  __Pyx_RefNannyDeclarations
  __Pyx_RefNannySetupContext("__get__ (wrapper)", 0);
  __pyx_r = __pyx_pf_5model_10Bondarenko_5V_myo___get__(((struct __pyx_obj_5model_Bondarenko *)__pyx_v_self));

  /* function exit code */
  __Pyx_RefNannyFinishContext();
  return __pyx_r;
}

static PyObject *__pyx_pf_5model_10Bondarenko_5V_myo___get__(struct __pyx_obj_5model_Bondarenko *__pyx_v_self) {
  PyObject *__pyx_r = NULL;
  __Pyx_RefNannyDeclarations
  __Pyx_RefNannySetupContext("__get__", 0);
  __Pyx_XDECREF(__pyx_r);
  __pyx_t_1 = PyFloat_FromDouble(__pyx_v_self->V_myo); if (unlikely(!__pyx_t_1)) {__pyx_filename = __pyx_f[0]; __pyx_lineno = 22; __pyx_clineno = __LINE__; goto __pyx_L1_error;}
  __Pyx_GOTREF(__pyx_t_1);
  __pyx_r = __pyx_t_1;
  __pyx_t_1 = 0;
  goto __pyx_L0;

  /* function exit code */
  __pyx_L1_error:;
  __Pyx_XDECREF(__pyx_t_1);
  __Pyx_AddTraceback("model.Bondarenko.V_myo.__get__", __pyx_clineno, __pyx_lineno, __pyx_filename);
  __pyx_r = NULL;
  __pyx_L0:;
  __Pyx_XGIVEREF(__pyx_r);
  __Pyx_RefNannyFinishContext();
  return __pyx_r;
}

/* Python wrapper */
static int __pyx_pw_5model_10Bondarenko_5V_myo_3__set__(PyObject *__pyx_v_self, PyObject *__pyx_v_value); /*proto*/
static int __pyx_pw_5model_10Bondarenko_5V_myo_3__set__(PyObject *__pyx_v_self, PyObject *__pyx_v_value) {
  int __pyx_r;
  __Pyx_RefNannyDeclarations
  __Pyx_RefNannySetupContext("__set__ (wrapper)", 0);
  __pyx_r = __pyx_pf_5model_10Bondarenko_5V_myo_2__set__(((struct __pyx_obj_5model_Bondarenko *)__pyx_v_self), ((PyObject *)__pyx_v_value));

  /* function exit code */
  __Pyx_RefNannyFinishContext();
  return __pyx_r;
}

static int __pyx_pf_5model_10Bondarenko_5V_myo_2__set__(struct __pyx_obj_5model_Bondarenko *__pyx_v_self, PyObject *__pyx_v_value) {
  int __pyx_r;
  __Pyx_RefNannyDeclarations
  __Pyx_RefNannySetupContext("__set__", 0);
  __pyx_t_1 = __pyx_PyFloat_AsDouble(__pyx_v_value); if (unlikely((__pyx_t_1 == (double)-1) && PyErr_Occurred())) {__pyx_filename = __pyx_f[0]; __pyx_lineno = 22; __pyx_clineno = __LINE__; goto __pyx_L1_error;}
  __pyx_v_self->V_myo = __pyx_t_1;

  /* function exit code */
  __pyx_r = 0;
  goto __pyx_L0;
  __pyx_L1_error:;
  __Pyx_AddTraceback("model.Bondarenko.V_myo.__set__", __pyx_clineno, __pyx_lineno, __pyx_filename);
  __pyx_r = -1;
  __pyx_L0:;
  __Pyx_RefNannyFinishContext();
  return __pyx_r;
}
```

```
 23:     cdef public double V_JSR
```

```
/* Python wrapper */
static PyObject *__pyx_pw_5model_10Bondarenko_5V_JSR_1__get__(PyObject *__pyx_v_self); /*proto*/
static PyObject *__pyx_pw_5model_10Bondarenko_5V_JSR_1__get__(PyObject *__pyx_v_self) {
  PyObject *__pyx_r = 0;
  __Pyx_RefNannyDeclarations
  __Pyx_RefNannySetupContext("__get__ (wrapper)", 0);
  __pyx_r = __pyx_pf_5model_10Bondarenko_5V_JSR___get__(((struct __pyx_obj_5model_Bondarenko *)__pyx_v_self));

  /* function exit code */
  __Pyx_RefNannyFinishContext();
  return __pyx_r;
}

static PyObject *__pyx_pf_5model_10Bondarenko_5V_JSR___get__(struct __pyx_obj_5model_Bondarenko *__pyx_v_self) {
  PyObject *__pyx_r = NULL;
  __Pyx_RefNannyDeclarations
  __Pyx_RefNannySetupContext("__get__", 0);
  __Pyx_XDECREF(__pyx_r);
  __pyx_t_1 = PyFloat_FromDouble(__pyx_v_self->V_JSR); if (unlikely(!__pyx_t_1)) {__pyx_filename = __pyx_f[0]; __pyx_lineno = 23; __pyx_clineno = __LINE__; goto __pyx_L1_error;}
  __Pyx_GOTREF(__pyx_t_1);
  __pyx_r = __pyx_t_1;
  __pyx_t_1 = 0;
  goto __pyx_L0;

  /* function exit code */
  __pyx_L1_error:;
  __Pyx_XDECREF(__pyx_t_1);
  __Pyx_AddTraceback("model.Bondarenko.V_JSR.__get__", __pyx_clineno, __pyx_lineno, __pyx_filename);
  __pyx_r = NULL;
  __pyx_L0:;
  __Pyx_XGIVEREF(__pyx_r);
  __Pyx_RefNannyFinishContext();
  return __pyx_r;
}

/* Python wrapper */
static int __pyx_pw_5model_10Bondarenko_5V_JSR_3__set__(PyObject *__pyx_v_self, PyObject *__pyx_v_value); /*proto*/
static int __pyx_pw_5model_10Bondarenko_5V_JSR_3__set__(PyObject *__pyx_v_self, PyObject *__pyx_v_value) {
  int __pyx_r;
  __Pyx_RefNannyDeclarations
  __Pyx_RefNannySetupContext("__set__ (wrapper)", 0);
  __pyx_r = __pyx_pf_5model_10Bondarenko_5V_JSR_2__set__(((struct __pyx_obj_5model_Bondarenko *)__pyx_v_self), ((PyObject *)__pyx_v_value));

  /* function exit code */
  __Pyx_RefNannyFinishContext();
  return __pyx_r;
}

static int __pyx_pf_5model_10Bondarenko_5V_JSR_2__set__(struct __pyx_obj_5model_Bondarenko *__pyx_v_self, PyObject *__pyx_v_value) {
  int __pyx_r;
  __Pyx_RefNannyDeclarations
  __Pyx_RefNannySetupContext("__set__", 0);
  __pyx_t_1 = __pyx_PyFloat_AsDouble(__pyx_v_value); if (unlikely((__pyx_t_1 == (double)-1) && PyErr_Occurred())) {__pyx_filename = __pyx_f[0]; __pyx_lineno = 23; __pyx_clineno = __LINE__; goto __pyx_L1_error;}
  __pyx_v_self->V_JSR = __pyx_t_1;

  /* function exit code */
  __pyx_r = 0;
  goto __pyx_L0;
  __pyx_L1_error:;
  __Pyx_AddTraceback("model.Bondarenko.V_JSR.__set__", __pyx_clineno, __pyx_lineno, __pyx_filename);
  __pyx_r = -1;
  __pyx_L0:;
  __Pyx_RefNannyFinishContext();
  return __pyx_r;
}
```

```
 24:     cdef public double V_NSR
```

```
/* Python wrapper */
static PyObject *__pyx_pw_5model_10Bondarenko_5V_NSR_1__get__(PyObject *__pyx_v_self); /*proto*/
static PyObject *__pyx_pw_5model_10Bondarenko_5V_NSR_1__get__(PyObject *__pyx_v_self) {
  PyObject *__pyx_r = 0;
  __Pyx_RefNannyDeclarations
  __Pyx_RefNannySetupContext("__get__ (wrapper)", 0);
  __pyx_r = __pyx_pf_5model_10Bondarenko_5V_NSR___get__(((struct __pyx_obj_5model_Bondarenko *)__pyx_v_self));

  /* function exit code */
  __Pyx_RefNannyFinishContext();
  return __pyx_r;
}

static PyObject *__pyx_pf_5model_10Bondarenko_5V_NSR___get__(struct __pyx_obj_5model_Bondarenko *__pyx_v_self) {
  PyObject *__pyx_r = NULL;
  __Pyx_RefNannyDeclarations
  __Pyx_RefNannySetupContext("__get__", 0);
  __Pyx_XDECREF(__pyx_r);
  __pyx_t_1 = PyFloat_FromDouble(__pyx_v_self->V_NSR); if (unlikely(!__pyx_t_1)) {__pyx_filename = __pyx_f[0]; __pyx_lineno = 24; __pyx_clineno = __LINE__; goto __pyx_L1_error;}
  __Pyx_GOTREF(__pyx_t_1);
  __pyx_r = __pyx_t_1;
  __pyx_t_1 = 0;
  goto __pyx_L0;

  /* function exit code */
  __pyx_L1_error:;
  __Pyx_XDECREF(__pyx_t_1);
  __Pyx_AddTraceback("model.Bondarenko.V_NSR.__get__", __pyx_clineno, __pyx_lineno, __pyx_filename);
  __pyx_r = NULL;
  __pyx_L0:;
  __Pyx_XGIVEREF(__pyx_r);
  __Pyx_RefNannyFinishContext();
  return __pyx_r;
}

/* Python wrapper */
static int __pyx_pw_5model_10Bondarenko_5V_NSR_3__set__(PyObject *__pyx_v_self, PyObject *__pyx_v_value); /*proto*/
static int __pyx_pw_5model_10Bondarenko_5V_NSR_3__set__(PyObject *__pyx_v_self, PyObject *__pyx_v_value) {
  int __pyx_r;
  __Pyx_RefNannyDeclarations
  __Pyx_RefNannySetupContext("__set__ (wrapper)", 0);
  __pyx_r = __pyx_pf_5model_10Bondarenko_5V_NSR_2__set__(((struct __pyx_obj_5model_Bondarenko *)__pyx_v_self), ((PyObject *)__pyx_v_value));

  /* function exit code */
  __Pyx_RefNannyFinishContext();
  return __pyx_r;
}

static int __pyx_pf_5model_10Bondarenko_5V_NSR_2__set__(struct __pyx_obj_5model_Bondarenko *__pyx_v_self, PyObject *__pyx_v_value) {
  int __pyx_r;
  __Pyx_RefNannyDeclarations
  __Pyx_RefNannySetupContext("__set__", 0);
  __pyx_t_1 = __pyx_PyFloat_AsDouble(__pyx_v_value); if (unlikely((__pyx_t_1 == (double)-1) && PyErr_Occurred())) {__pyx_filename = __pyx_f[0]; __pyx_lineno = 24; __pyx_clineno = __LINE__; goto __pyx_L1_error;}
  __pyx_v_self->V_NSR = __pyx_t_1;

  /* function exit code */
  __pyx_r = 0;
  goto __pyx_L0;
  __pyx_L1_error:;
  __Pyx_AddTraceback("model.Bondarenko.V_NSR.__set__", __pyx_clineno, __pyx_lineno, __pyx_filename);
  __pyx_r = -1;
  __pyx_L0:;
  __Pyx_RefNannyFinishContext();
  return __pyx_r;
}
```

```
 25:     cdef public double V_ss
```

```
/* Python wrapper */
static PyObject *__pyx_pw_5model_10Bondarenko_4V_ss_1__get__(PyObject *__pyx_v_self); /*proto*/
static PyObject *__pyx_pw_5model_10Bondarenko_4V_ss_1__get__(PyObject *__pyx_v_self) {
  PyObject *__pyx_r = 0;
  __Pyx_RefNannyDeclarations
  __Pyx_RefNannySetupContext("__get__ (wrapper)", 0);
  __pyx_r = __pyx_pf_5model_10Bondarenko_4V_ss___get__(((struct __pyx_obj_5model_Bondarenko *)__pyx_v_self));

  /* function exit code */
  __Pyx_RefNannyFinishContext();
  return __pyx_r;
}

static PyObject *__pyx_pf_5model_10Bondarenko_4V_ss___get__(struct __pyx_obj_5model_Bondarenko *__pyx_v_self) {
  PyObject *__pyx_r = NULL;
  __Pyx_RefNannyDeclarations
  __Pyx_RefNannySetupContext("__get__", 0);
  __Pyx_XDECREF(__pyx_r);
  __pyx_t_1 = PyFloat_FromDouble(__pyx_v_self->V_ss); if (unlikely(!__pyx_t_1)) {__pyx_filename = __pyx_f[0]; __pyx_lineno = 25; __pyx_clineno = __LINE__; goto __pyx_L1_error;}
  __Pyx_GOTREF(__pyx_t_1);
  __pyx_r = __pyx_t_1;
  __pyx_t_1 = 0;
  goto __pyx_L0;

  /* function exit code */
  __pyx_L1_error:;
  __Pyx_XDECREF(__pyx_t_1);
  __Pyx_AddTraceback("model.Bondarenko.V_ss.__get__", __pyx_clineno, __pyx_lineno, __pyx_filename);
  __pyx_r = NULL;
  __pyx_L0:;
  __Pyx_XGIVEREF(__pyx_r);
  __Pyx_RefNannyFinishContext();
  return __pyx_r;
}

/* Python wrapper */
static int __pyx_pw_5model_10Bondarenko_4V_ss_3__set__(PyObject *__pyx_v_self, PyObject *__pyx_v_value); /*proto*/
static int __pyx_pw_5model_10Bondarenko_4V_ss_3__set__(PyObject *__pyx_v_self, PyObject *__pyx_v_value) {
  int __pyx_r;
  __Pyx_RefNannyDeclarations
  __Pyx_RefNannySetupContext("__set__ (wrapper)", 0);
  __pyx_r = __pyx_pf_5model_10Bondarenko_4V_ss_2__set__(((struct __pyx_obj_5model_Bondarenko *)__pyx_v_self), ((PyObject *)__pyx_v_value));

  /* function exit code */
  __Pyx_RefNannyFinishContext();
  return __pyx_r;
}

static int __pyx_pf_5model_10Bondarenko_4V_ss_2__set__(struct __pyx_obj_5model_Bondarenko *__pyx_v_self, PyObject *__pyx_v_value) {
  int __pyx_r;
  __Pyx_RefNannyDeclarations
  __Pyx_RefNannySetupContext("__set__", 0);
  __pyx_t_1 = __pyx_PyFloat_AsDouble(__pyx_v_value); if (unlikely((__pyx_t_1 == (double)-1) && PyErr_Occurred())) {__pyx_filename = __pyx_f[0]; __pyx_lineno = 25; __pyx_clineno = __LINE__; goto __pyx_L1_error;}
  __pyx_v_self->V_ss = __pyx_t_1;

  /* function exit code */
  __pyx_r = 0;
  goto __pyx_L0;
  __pyx_L1_error:;
  __Pyx_AddTraceback("model.Bondarenko.V_ss.__set__", __pyx_clineno, __pyx_lineno, __pyx_filename);
  __pyx_r = -1;
  __pyx_L0:;
  __Pyx_RefNannyFinishContext();
  return __pyx_r;
}
```

```
 26:     cdef public double K_0
```

```
/* Python wrapper */
static PyObject *__pyx_pw_5model_10Bondarenko_3K_0_1__get__(PyObject *__pyx_v_self); /*proto*/
static PyObject *__pyx_pw_5model_10Bondarenko_3K_0_1__get__(PyObject *__pyx_v_self) {
  PyObject *__pyx_r = 0;
  __Pyx_RefNannyDeclarations
  __Pyx_RefNannySetupContext("__get__ (wrapper)", 0);
  __pyx_r = __pyx_pf_5model_10Bondarenko_3K_0___get__(((struct __pyx_obj_5model_Bondarenko *)__pyx_v_self));

  /* function exit code */
  __Pyx_RefNannyFinishContext();
  return __pyx_r;
}

static PyObject *__pyx_pf_5model_10Bondarenko_3K_0___get__(struct __pyx_obj_5model_Bondarenko *__pyx_v_self) {
  PyObject *__pyx_r = NULL;
  __Pyx_RefNannyDeclarations
  __Pyx_RefNannySetupContext("__get__", 0);
  __Pyx_XDECREF(__pyx_r);
  __pyx_t_1 = PyFloat_FromDouble(__pyx_v_self->K_0); if (unlikely(!__pyx_t_1)) {__pyx_filename = __pyx_f[0]; __pyx_lineno = 26; __pyx_clineno = __LINE__; goto __pyx_L1_error;}
  __Pyx_GOTREF(__pyx_t_1);
  __pyx_r = __pyx_t_1;
  __pyx_t_1 = 0;
  goto __pyx_L0;

  /* function exit code */
  __pyx_L1_error:;
  __Pyx_XDECREF(__pyx_t_1);
  __Pyx_AddTraceback("model.Bondarenko.K_0.__get__", __pyx_clineno, __pyx_lineno, __pyx_filename);
  __pyx_r = NULL;
  __pyx_L0:;
  __Pyx_XGIVEREF(__pyx_r);
  __Pyx_RefNannyFinishContext();
  return __pyx_r;
}

/* Python wrapper */
static int __pyx_pw_5model_10Bondarenko_3K_0_3__set__(PyObject *__pyx_v_self, PyObject *__pyx_v_value); /*proto*/
static int __pyx_pw_5model_10Bondarenko_3K_0_3__set__(PyObject *__pyx_v_self, PyObject *__pyx_v_value) {
  int __pyx_r;
  __Pyx_RefNannyDeclarations
  __Pyx_RefNannySetupContext("__set__ (wrapper)", 0);
  __pyx_r = __pyx_pf_5model_10Bondarenko_3K_0_2__set__(((struct __pyx_obj_5model_Bondarenko *)__pyx_v_self), ((PyObject *)__pyx_v_value));

  /* function exit code */
  __Pyx_RefNannyFinishContext();
  return __pyx_r;
}

static int __pyx_pf_5model_10Bondarenko_3K_0_2__set__(struct __pyx_obj_5model_Bondarenko *__pyx_v_self, PyObject *__pyx_v_value) {
  int __pyx_r;
  __Pyx_RefNannyDeclarations
  __Pyx_RefNannySetupContext("__set__", 0);
  __pyx_t_1 = __pyx_PyFloat_AsDouble(__pyx_v_value); if (unlikely((__pyx_t_1 == (double)-1) && PyErr_Occurred())) {__pyx_filename = __pyx_f[0]; __pyx_lineno = 26; __pyx_clineno = __LINE__; goto __pyx_L1_error;}
  __pyx_v_self->K_0 = __pyx_t_1;

  /* function exit code */
  __pyx_r = 0;
  goto __pyx_L0;
  __pyx_L1_error:;
  __Pyx_AddTraceback("model.Bondarenko.K_0.__set__", __pyx_clineno, __pyx_lineno, __pyx_filename);
  __pyx_r = -1;
  __pyx_L0:;
  __Pyx_RefNannyFinishContext();
  return __pyx_r;
}
```

```
 27:     cdef public double Na_0
```

```
/* Python wrapper */
static PyObject *__pyx_pw_5model_10Bondarenko_4Na_0_1__get__(PyObject *__pyx_v_self); /*proto*/
static PyObject *__pyx_pw_5model_10Bondarenko_4Na_0_1__get__(PyObject *__pyx_v_self) {
  PyObject *__pyx_r = 0;
  __Pyx_RefNannyDeclarations
  __Pyx_RefNannySetupContext("__get__ (wrapper)", 0);
  __pyx_r = __pyx_pf_5model_10Bondarenko_4Na_0___get__(((struct __pyx_obj_5model_Bondarenko *)__pyx_v_self));

  /* function exit code */
  __Pyx_RefNannyFinishContext();
  return __pyx_r;
}

static PyObject *__pyx_pf_5model_10Bondarenko_4Na_0___get__(struct __pyx_obj_5model_Bondarenko *__pyx_v_self) {
  PyObject *__pyx_r = NULL;
  __Pyx_RefNannyDeclarations
  __Pyx_RefNannySetupContext("__get__", 0);
  __Pyx_XDECREF(__pyx_r);
  __pyx_t_1 = PyFloat_FromDouble(__pyx_v_self->Na_0); if (unlikely(!__pyx_t_1)) {__pyx_filename = __pyx_f[0]; __pyx_lineno = 27; __pyx_clineno = __LINE__; goto __pyx_L1_error;}
  __Pyx_GOTREF(__pyx_t_1);
  __pyx_r = __pyx_t_1;
  __pyx_t_1 = 0;
  goto __pyx_L0;

  /* function exit code */
  __pyx_L1_error:;
  __Pyx_XDECREF(__pyx_t_1);
  __Pyx_AddTraceback("model.Bondarenko.Na_0.__get__", __pyx_clineno, __pyx_lineno, __pyx_filename);
  __pyx_r = NULL;
  __pyx_L0:;
  __Pyx_XGIVEREF(__pyx_r);
  __Pyx_RefNannyFinishContext();
  return __pyx_r;
}

/* Python wrapper */
static int __pyx_pw_5model_10Bondarenko_4Na_0_3__set__(PyObject *__pyx_v_self, PyObject *__pyx_v_value); /*proto*/
static int __pyx_pw_5model_10Bondarenko_4Na_0_3__set__(PyObject *__pyx_v_self, PyObject *__pyx_v_value) {
  int __pyx_r;
  __Pyx_RefNannyDeclarations
  __Pyx_RefNannySetupContext("__set__ (wrapper)", 0);
  __pyx_r = __pyx_pf_5model_10Bondarenko_4Na_0_2__set__(((struct __pyx_obj_5model_Bondarenko *)__pyx_v_self), ((PyObject *)__pyx_v_value));

  /* function exit code */
  __Pyx_RefNannyFinishContext();
  return __pyx_r;
}

static int __pyx_pf_5model_10Bondarenko_4Na_0_2__set__(struct __pyx_obj_5model_Bondarenko *__pyx_v_self, PyObject *__pyx_v_value) {
  int __pyx_r;
  __Pyx_RefNannyDeclarations
  __Pyx_RefNannySetupContext("__set__", 0);
  __pyx_t_1 = __pyx_PyFloat_AsDouble(__pyx_v_value); if (unlikely((__pyx_t_1 == (double)-1) && PyErr_Occurred())) {__pyx_filename = __pyx_f[0]; __pyx_lineno = 27; __pyx_clineno = __LINE__; goto __pyx_L1_error;}
  __pyx_v_self->Na_0 = __pyx_t_1;

  /* function exit code */
  __pyx_r = 0;
  goto __pyx_L0;
  __pyx_L1_error:;
  __Pyx_AddTraceback("model.Bondarenko.Na_0.__set__", __pyx_clineno, __pyx_lineno, __pyx_filename);
  __pyx_r = -1;
  __pyx_L0:;
  __Pyx_RefNannyFinishContext();
  return __pyx_r;
}
```

```
 28:     cdef public double Ca_0
```

```
/* Python wrapper */
static PyObject *__pyx_pw_5model_10Bondarenko_4Ca_0_1__get__(PyObject *__pyx_v_self); /*proto*/
static PyObject *__pyx_pw_5model_10Bondarenko_4Ca_0_1__get__(PyObject *__pyx_v_self) {
  PyObject *__pyx_r = 0;
  __Pyx_RefNannyDeclarations
  __Pyx_RefNannySetupContext("__get__ (wrapper)", 0);
  __pyx_r = __pyx_pf_5model_10Bondarenko_4Ca_0___get__(((struct __pyx_obj_5model_Bondarenko *)__pyx_v_self));

  /* function exit code */
  __Pyx_RefNannyFinishContext();
  return __pyx_r;
}

static PyObject *__pyx_pf_5model_10Bondarenko_4Ca_0___get__(struct __pyx_obj_5model_Bondarenko *__pyx_v_self) {
  PyObject *__pyx_r = NULL;
  __Pyx_RefNannyDeclarations
  __Pyx_RefNannySetupContext("__get__", 0);
  __Pyx_XDECREF(__pyx_r);
  __pyx_t_1 = PyFloat_FromDouble(__pyx_v_self->Ca_0); if (unlikely(!__pyx_t_1)) {__pyx_filename = __pyx_f[0]; __pyx_lineno = 28; __pyx_clineno = __LINE__; goto __pyx_L1_error;}
  __Pyx_GOTREF(__pyx_t_1);
  __pyx_r = __pyx_t_1;
  __pyx_t_1 = 0;
  goto __pyx_L0;

  /* function exit code */
  __pyx_L1_error:;
  __Pyx_XDECREF(__pyx_t_1);
  __Pyx_AddTraceback("model.Bondarenko.Ca_0.__get__", __pyx_clineno, __pyx_lineno, __pyx_filename);
  __pyx_r = NULL;
  __pyx_L0:;
  __Pyx_XGIVEREF(__pyx_r);
  __Pyx_RefNannyFinishContext();
  return __pyx_r;
}

/* Python wrapper */
static int __pyx_pw_5model_10Bondarenko_4Ca_0_3__set__(PyObject *__pyx_v_self, PyObject *__pyx_v_value); /*proto*/
static int __pyx_pw_5model_10Bondarenko_4Ca_0_3__set__(PyObject *__pyx_v_self, PyObject *__pyx_v_value) {
  int __pyx_r;
  __Pyx_RefNannyDeclarations
  __Pyx_RefNannySetupContext("__set__ (wrapper)", 0);
  __pyx_r = __pyx_pf_5model_10Bondarenko_4Ca_0_2__set__(((struct __pyx_obj_5model_Bondarenko *)__pyx_v_self), ((PyObject *)__pyx_v_value));

  /* function exit code */
  __Pyx_RefNannyFinishContext();
  return __pyx_r;
}

static int __pyx_pf_5model_10Bondarenko_4Ca_0_2__set__(struct __pyx_obj_5model_Bondarenko *__pyx_v_self, PyObject *__pyx_v_value) {
  int __pyx_r;
  __Pyx_RefNannyDeclarations
  __Pyx_RefNannySetupContext("__set__", 0);
  __pyx_t_1 = __pyx_PyFloat_AsDouble(__pyx_v_value); if (unlikely((__pyx_t_1 == (double)-1) && PyErr_Occurred())) {__pyx_filename = __pyx_f[0]; __pyx_lineno = 28; __pyx_clineno = __LINE__; goto __pyx_L1_error;}
  __pyx_v_self->Ca_0 = __pyx_t_1;

  /* function exit code */
  __pyx_r = 0;
  goto __pyx_L0;
  __pyx_L1_error:;
  __Pyx_AddTraceback("model.Bondarenko.Ca_0.__set__", __pyx_clineno, __pyx_lineno, __pyx_filename);
  __pyx_r = -1;
  __pyx_L0:;
  __Pyx_RefNannyFinishContext();
  return __pyx_r;
}
```

```
 29:     cdef public double v_1
```

```
/* Python wrapper */
static PyObject *__pyx_pw_5model_10Bondarenko_3v_1_1__get__(PyObject *__pyx_v_self); /*proto*/
static PyObject *__pyx_pw_5model_10Bondarenko_3v_1_1__get__(PyObject *__pyx_v_self) {
  PyObject *__pyx_r = 0;
  __Pyx_RefNannyDeclarations
  __Pyx_RefNannySetupContext("__get__ (wrapper)", 0);
  __pyx_r = __pyx_pf_5model_10Bondarenko_3v_1___get__(((struct __pyx_obj_5model_Bondarenko *)__pyx_v_self));

  /* function exit code */
  __Pyx_RefNannyFinishContext();
  return __pyx_r;
}

static PyObject *__pyx_pf_5model_10Bondarenko_3v_1___get__(struct __pyx_obj_5model_Bondarenko *__pyx_v_self) {
  PyObject *__pyx_r = NULL;
  __Pyx_RefNannyDeclarations
  __Pyx_RefNannySetupContext("__get__", 0);
  __Pyx_XDECREF(__pyx_r);
  __pyx_t_1 = PyFloat_FromDouble(__pyx_v_self->v_1); if (unlikely(!__pyx_t_1)) {__pyx_filename = __pyx_f[0]; __pyx_lineno = 29; __pyx_clineno = __LINE__; goto __pyx_L1_error;}
  __Pyx_GOTREF(__pyx_t_1);
  __pyx_r = __pyx_t_1;
  __pyx_t_1 = 0;
  goto __pyx_L0;

  /* function exit code */
  __pyx_L1_error:;
  __Pyx_XDECREF(__pyx_t_1);
  __Pyx_AddTraceback("model.Bondarenko.v_1.__get__", __pyx_clineno, __pyx_lineno, __pyx_filename);
  __pyx_r = NULL;
  __pyx_L0:;
  __Pyx_XGIVEREF(__pyx_r);
  __Pyx_RefNannyFinishContext();
  return __pyx_r;
}

/* Python wrapper */
static int __pyx_pw_5model_10Bondarenko_3v_1_3__set__(PyObject *__pyx_v_self, PyObject *__pyx_v_value); /*proto*/
static int __pyx_pw_5model_10Bondarenko_3v_1_3__set__(PyObject *__pyx_v_self, PyObject *__pyx_v_value) {
  int __pyx_r;
  __Pyx_RefNannyDeclarations
  __Pyx_RefNannySetupContext("__set__ (wrapper)", 0);
  __pyx_r = __pyx_pf_5model_10Bondarenko_3v_1_2__set__(((struct __pyx_obj_5model_Bondarenko *)__pyx_v_self), ((PyObject *)__pyx_v_value));

  /* function exit code */
  __Pyx_RefNannyFinishContext();
  return __pyx_r;
}

static int __pyx_pf_5model_10Bondarenko_3v_1_2__set__(struct __pyx_obj_5model_Bondarenko *__pyx_v_self, PyObject *__pyx_v_value) {
  int __pyx_r;
  __Pyx_RefNannyDeclarations
  __Pyx_RefNannySetupContext("__set__", 0);
  __pyx_t_1 = __pyx_PyFloat_AsDouble(__pyx_v_value); if (unlikely((__pyx_t_1 == (double)-1) && PyErr_Occurred())) {__pyx_filename = __pyx_f[0]; __pyx_lineno = 29; __pyx_clineno = __LINE__; goto __pyx_L1_error;}
  __pyx_v_self->v_1 = __pyx_t_1;

  /* function exit code */
  __pyx_r = 0;
  goto __pyx_L0;
  __pyx_L1_error:;
  __Pyx_AddTraceback("model.Bondarenko.v_1.__set__", __pyx_clineno, __pyx_lineno, __pyx_filename);
  __pyx_r = -1;
  __pyx_L0:;
  __Pyx_RefNannyFinishContext();
  return __pyx_r;
}
```

```
 30:     cdef public double v_2
```

```
/* Python wrapper */
static PyObject *__pyx_pw_5model_10Bondarenko_3v_2_1__get__(PyObject *__pyx_v_self); /*proto*/
static PyObject *__pyx_pw_5model_10Bondarenko_3v_2_1__get__(PyObject *__pyx_v_self) {
  PyObject *__pyx_r = 0;
  __Pyx_RefNannyDeclarations
  __Pyx_RefNannySetupContext("__get__ (wrapper)", 0);
  __pyx_r = __pyx_pf_5model_10Bondarenko_3v_2___get__(((struct __pyx_obj_5model_Bondarenko *)__pyx_v_self));

  /* function exit code */
  __Pyx_RefNannyFinishContext();
  return __pyx_r;
}

static PyObject *__pyx_pf_5model_10Bondarenko_3v_2___get__(struct __pyx_obj_5model_Bondarenko *__pyx_v_self) {
  PyObject *__pyx_r = NULL;
  __Pyx_RefNannyDeclarations
  __Pyx_RefNannySetupContext("__get__", 0);
  __Pyx_XDECREF(__pyx_r);
  __pyx_t_1 = PyFloat_FromDouble(__pyx_v_self->v_2); if (unlikely(!__pyx_t_1)) {__pyx_filename = __pyx_f[0]; __pyx_lineno = 30; __pyx_clineno = __LINE__; goto __pyx_L1_error;}
  __Pyx_GOTREF(__pyx_t_1);
  __pyx_r = __pyx_t_1;
  __pyx_t_1 = 0;
  goto __pyx_L0;

  /* function exit code */
  __pyx_L1_error:;
  __Pyx_XDECREF(__pyx_t_1);
  __Pyx_AddTraceback("model.Bondarenko.v_2.__get__", __pyx_clineno, __pyx_lineno, __pyx_filename);
  __pyx_r = NULL;
  __pyx_L0:;
  __Pyx_XGIVEREF(__pyx_r);
  __Pyx_RefNannyFinishContext();
  return __pyx_r;
}

/* Python wrapper */
static int __pyx_pw_5model_10Bondarenko_3v_2_3__set__(PyObject *__pyx_v_self, PyObject *__pyx_v_value); /*proto*/
static int __pyx_pw_5model_10Bondarenko_3v_2_3__set__(PyObject *__pyx_v_self, PyObject *__pyx_v_value) {
  int __pyx_r;
  __Pyx_RefNannyDeclarations
  __Pyx_RefNannySetupContext("__set__ (wrapper)", 0);
  __pyx_r = __pyx_pf_5model_10Bondarenko_3v_2_2__set__(((struct __pyx_obj_5model_Bondarenko *)__pyx_v_self), ((PyObject *)__pyx_v_value));

  /* function exit code */
  __Pyx_RefNannyFinishContext();
  return __pyx_r;
}

static int __pyx_pf_5model_10Bondarenko_3v_2_2__set__(struct __pyx_obj_5model_Bondarenko *__pyx_v_self, PyObject *__pyx_v_value) {
  int __pyx_r;
  __Pyx_RefNannyDeclarations
  __Pyx_RefNannySetupContext("__set__", 0);
  __pyx_t_1 = __pyx_PyFloat_AsDouble(__pyx_v_value); if (unlikely((__pyx_t_1 == (double)-1) && PyErr_Occurred())) {__pyx_filename = __pyx_f[0]; __pyx_lineno = 30; __pyx_clineno = __LINE__; goto __pyx_L1_error;}
  __pyx_v_self->v_2 = __pyx_t_1;

  /* function exit code */
  __pyx_r = 0;
  goto __pyx_L0;
  __pyx_L1_error:;
  __Pyx_AddTraceback("model.Bondarenko.v_2.__set__", __pyx_clineno, __pyx_lineno, __pyx_filename);
  __pyx_r = -1;
  __pyx_L0:;
  __Pyx_RefNannyFinishContext();
  return __pyx_r;
}
```

```
 31:     cdef public double v_3
```

```
/* Python wrapper */
static PyObject *__pyx_pw_5model_10Bondarenko_3v_3_1__get__(PyObject *__pyx_v_self); /*proto*/
static PyObject *__pyx_pw_5model_10Bondarenko_3v_3_1__get__(PyObject *__pyx_v_self) {
  PyObject *__pyx_r = 0;
  __Pyx_RefNannyDeclarations
  __Pyx_RefNannySetupContext("__get__ (wrapper)", 0);
  __pyx_r = __pyx_pf_5model_10Bondarenko_3v_3___get__(((struct __pyx_obj_5model_Bondarenko *)__pyx_v_self));

  /* function exit code */
  __Pyx_RefNannyFinishContext();
  return __pyx_r;
}

static PyObject *__pyx_pf_5model_10Bondarenko_3v_3___get__(struct __pyx_obj_5model_Bondarenko *__pyx_v_self) {
  PyObject *__pyx_r = NULL;
  __Pyx_RefNannyDeclarations
  __Pyx_RefNannySetupContext("__get__", 0);
  __Pyx_XDECREF(__pyx_r);
  __pyx_t_1 = PyFloat_FromDouble(__pyx_v_self->v_3); if (unlikely(!__pyx_t_1)) {__pyx_filename = __pyx_f[0]; __pyx_lineno = 31; __pyx_clineno = __LINE__; goto __pyx_L1_error;}
  __Pyx_GOTREF(__pyx_t_1);
  __pyx_r = __pyx_t_1;
  __pyx_t_1 = 0;
  goto __pyx_L0;

  /* function exit code */
  __pyx_L1_error:;
  __Pyx_XDECREF(__pyx_t_1);
  __Pyx_AddTraceback("model.Bondarenko.v_3.__get__", __pyx_clineno, __pyx_lineno, __pyx_filename);
  __pyx_r = NULL;
  __pyx_L0:;
  __Pyx_XGIVEREF(__pyx_r);
  __Pyx_RefNannyFinishContext();
  return __pyx_r;
}

/* Python wrapper */
static int __pyx_pw_5model_10Bondarenko_3v_3_3__set__(PyObject *__pyx_v_self, PyObject *__pyx_v_value); /*proto*/
static int __pyx_pw_5model_10Bondarenko_3v_3_3__set__(PyObject *__pyx_v_self, PyObject *__pyx_v_value) {
  int __pyx_r;
  __Pyx_RefNannyDeclarations
  __Pyx_RefNannySetupContext("__set__ (wrapper)", 0);
  __pyx_r = __pyx_pf_5model_10Bondarenko_3v_3_2__set__(((struct __pyx_obj_5model_Bondarenko *)__pyx_v_self), ((PyObject *)__pyx_v_value));

  /* function exit code */
  __Pyx_RefNannyFinishContext();
  return __pyx_r;
}

static int __pyx_pf_5model_10Bondarenko_3v_3_2__set__(struct __pyx_obj_5model_Bondarenko *__pyx_v_self, PyObject *__pyx_v_value) {
  int __pyx_r;
  __Pyx_RefNannyDeclarations
  __Pyx_RefNannySetupContext("__set__", 0);
  __pyx_t_1 = __pyx_PyFloat_AsDouble(__pyx_v_value); if (unlikely((__pyx_t_1 == (double)-1) && PyErr_Occurred())) {__pyx_filename = __pyx_f[0]; __pyx_lineno = 31; __pyx_clineno = __LINE__; goto __pyx_L1_error;}
  __pyx_v_self->v_3 = __pyx_t_1;

  /* function exit code */
  __pyx_r = 0;
  goto __pyx_L0;
  __pyx_L1_error:;
  __Pyx_AddTraceback("model.Bondarenko.v_3.__set__", __pyx_clineno, __pyx_lineno, __pyx_filename);
  __pyx_r = -1;
  __pyx_L0:;
  __Pyx_RefNannyFinishContext();
  return __pyx_r;
}
```

```
 32:     cdef public double k_mup
```

```
/* Python wrapper */
static PyObject *__pyx_pw_5model_10Bondarenko_5k_mup_1__get__(PyObject *__pyx_v_self); /*proto*/
static PyObject *__pyx_pw_5model_10Bondarenko_5k_mup_1__get__(PyObject *__pyx_v_self) {
  PyObject *__pyx_r = 0;
  __Pyx_RefNannyDeclarations
  __Pyx_RefNannySetupContext("__get__ (wrapper)", 0);
  __pyx_r = __pyx_pf_5model_10Bondarenko_5k_mup___get__(((struct __pyx_obj_5model_Bondarenko *)__pyx_v_self));

  /* function exit code */
  __Pyx_RefNannyFinishContext();
  return __pyx_r;
}

static PyObject *__pyx_pf_5model_10Bondarenko_5k_mup___get__(struct __pyx_obj_5model_Bondarenko *__pyx_v_self) {
  PyObject *__pyx_r = NULL;
  __Pyx_RefNannyDeclarations
  __Pyx_RefNannySetupContext("__get__", 0);
  __Pyx_XDECREF(__pyx_r);
  __pyx_t_1 = PyFloat_FromDouble(__pyx_v_self->k_mup); if (unlikely(!__pyx_t_1)) {__pyx_filename = __pyx_f[0]; __pyx_lineno = 32; __pyx_clineno = __LINE__; goto __pyx_L1_error;}
  __Pyx_GOTREF(__pyx_t_1);
  __pyx_r = __pyx_t_1;
  __pyx_t_1 = 0;
  goto __pyx_L0;

  /* function exit code */
  __pyx_L1_error:;
  __Pyx_XDECREF(__pyx_t_1);
  __Pyx_AddTraceback("model.Bondarenko.k_mup.__get__", __pyx_clineno, __pyx_lineno, __pyx_filename);
  __pyx_r = NULL;
  __pyx_L0:;
  __Pyx_XGIVEREF(__pyx_r);
  __Pyx_RefNannyFinishContext();
  return __pyx_r;
}

/* Python wrapper */
static int __pyx_pw_5model_10Bondarenko_5k_mup_3__set__(PyObject *__pyx_v_self, PyObject *__pyx_v_value); /*proto*/
static int __pyx_pw_5model_10Bondarenko_5k_mup_3__set__(PyObject *__pyx_v_self, PyObject *__pyx_v_value) {
  int __pyx_r;
  __Pyx_RefNannyDeclarations
  __Pyx_RefNannySetupContext("__set__ (wrapper)", 0);
  __pyx_r = __pyx_pf_5model_10Bondarenko_5k_mup_2__set__(((struct __pyx_obj_5model_Bondarenko *)__pyx_v_self), ((PyObject *)__pyx_v_value));

  /* function exit code */
  __Pyx_RefNannyFinishContext();
  return __pyx_r;
}

static int __pyx_pf_5model_10Bondarenko_5k_mup_2__set__(struct __pyx_obj_5model_Bondarenko *__pyx_v_self, PyObject *__pyx_v_value) {
  int __pyx_r;
  __Pyx_RefNannyDeclarations
  __Pyx_RefNannySetupContext("__set__", 0);
  __pyx_t_1 = __pyx_PyFloat_AsDouble(__pyx_v_value); if (unlikely((__pyx_t_1 == (double)-1) && PyErr_Occurred())) {__pyx_filename = __pyx_f[0]; __pyx_lineno = 32; __pyx_clineno = __LINE__; goto __pyx_L1_error;}
  __pyx_v_self->k_mup = __pyx_t_1;

  /* function exit code */
  __pyx_r = 0;
  goto __pyx_L0;
  __pyx_L1_error:;
  __Pyx_AddTraceback("model.Bondarenko.k_mup.__set__", __pyx_clineno, __pyx_lineno, __pyx_filename);
  __pyx_r = -1;
  __pyx_L0:;
  __Pyx_RefNannyFinishContext();
  return __pyx_r;
}
```

```
 33:     cdef public double t_tr
```

```
/* Python wrapper */
static PyObject *__pyx_pw_5model_10Bondarenko_4t_tr_1__get__(PyObject *__pyx_v_self); /*proto*/
static PyObject *__pyx_pw_5model_10Bondarenko_4t_tr_1__get__(PyObject *__pyx_v_self) {
  PyObject *__pyx_r = 0;
  __Pyx_RefNannyDeclarations
  __Pyx_RefNannySetupContext("__get__ (wrapper)", 0);
  __pyx_r = __pyx_pf_5model_10Bondarenko_4t_tr___get__(((struct __pyx_obj_5model_Bondarenko *)__pyx_v_self));

  /* function exit code */
  __Pyx_RefNannyFinishContext();
  return __pyx_r;
}

static PyObject *__pyx_pf_5model_10Bondarenko_4t_tr___get__(struct __pyx_obj_5model_Bondarenko *__pyx_v_self) {
  PyObject *__pyx_r = NULL;
  __Pyx_RefNannyDeclarations
  __Pyx_RefNannySetupContext("__get__", 0);
  __Pyx_XDECREF(__pyx_r);
  __pyx_t_1 = PyFloat_FromDouble(__pyx_v_self->t_tr); if (unlikely(!__pyx_t_1)) {__pyx_filename = __pyx_f[0]; __pyx_lineno = 33; __pyx_clineno = __LINE__; goto __pyx_L1_error;}
  __Pyx_GOTREF(__pyx_t_1);
  __pyx_r = __pyx_t_1;
  __pyx_t_1 = 0;
  goto __pyx_L0;

  /* function exit code */
  __pyx_L1_error:;
  __Pyx_XDECREF(__pyx_t_1);
  __Pyx_AddTraceback("model.Bondarenko.t_tr.__get__", __pyx_clineno, __pyx_lineno, __pyx_filename);
  __pyx_r = NULL;
  __pyx_L0:;
  __Pyx_XGIVEREF(__pyx_r);
  __Pyx_RefNannyFinishContext();
  return __pyx_r;
}

/* Python wrapper */
static int __pyx_pw_5model_10Bondarenko_4t_tr_3__set__(PyObject *__pyx_v_self, PyObject *__pyx_v_value); /*proto*/
static int __pyx_pw_5model_10Bondarenko_4t_tr_3__set__(PyObject *__pyx_v_self, PyObject *__pyx_v_value) {
  int __pyx_r;
  __Pyx_RefNannyDeclarations
  __Pyx_RefNannySetupContext("__set__ (wrapper)", 0);
  __pyx_r = __pyx_pf_5model_10Bondarenko_4t_tr_2__set__(((struct __pyx_obj_5model_Bondarenko *)__pyx_v_self), ((PyObject *)__pyx_v_value));

  /* function exit code */
  __Pyx_RefNannyFinishContext();
  return __pyx_r;
}

static int __pyx_pf_5model_10Bondarenko_4t_tr_2__set__(struct __pyx_obj_5model_Bondarenko *__pyx_v_self, PyObject *__pyx_v_value) {
  int __pyx_r;
  __Pyx_RefNannyDeclarations
  __Pyx_RefNannySetupContext("__set__", 0);
  __pyx_t_1 = __pyx_PyFloat_AsDouble(__pyx_v_value); if (unlikely((__pyx_t_1 == (double)-1) && PyErr_Occurred())) {__pyx_filename = __pyx_f[0]; __pyx_lineno = 33; __pyx_clineno = __LINE__; goto __pyx_L1_error;}
  __pyx_v_self->t_tr = __pyx_t_1;

  /* function exit code */
  __pyx_r = 0;
  goto __pyx_L0;
  __pyx_L1_error:;
  __Pyx_AddTraceback("model.Bondarenko.t_tr.__set__", __pyx_clineno, __pyx_lineno, __pyx_filename);
  __pyx_r = -1;
  __pyx_L0:;
  __Pyx_RefNannyFinishContext();
  return __pyx_r;
}
```

```
 34:     cdef public double t_xfer
```

```
/* Python wrapper */
static PyObject *__pyx_pw_5model_10Bondarenko_6t_xfer_1__get__(PyObject *__pyx_v_self); /*proto*/
static PyObject *__pyx_pw_5model_10Bondarenko_6t_xfer_1__get__(PyObject *__pyx_v_self) {
  PyObject *__pyx_r = 0;
  __Pyx_RefNannyDeclarations
  __Pyx_RefNannySetupContext("__get__ (wrapper)", 0);
  __pyx_r = __pyx_pf_5model_10Bondarenko_6t_xfer___get__(((struct __pyx_obj_5model_Bondarenko *)__pyx_v_self));

  /* function exit code */
  __Pyx_RefNannyFinishContext();
  return __pyx_r;
}

static PyObject *__pyx_pf_5model_10Bondarenko_6t_xfer___get__(struct __pyx_obj_5model_Bondarenko *__pyx_v_self) {
  PyObject *__pyx_r = NULL;
  __Pyx_RefNannyDeclarations
  __Pyx_RefNannySetupContext("__get__", 0);
  __Pyx_XDECREF(__pyx_r);
  __pyx_t_1 = PyFloat_FromDouble(__pyx_v_self->t_xfer); if (unlikely(!__pyx_t_1)) {__pyx_filename = __pyx_f[0]; __pyx_lineno = 34; __pyx_clineno = __LINE__; goto __pyx_L1_error;}
  __Pyx_GOTREF(__pyx_t_1);
  __pyx_r = __pyx_t_1;
  __pyx_t_1 = 0;
  goto __pyx_L0;

  /* function exit code */
  __pyx_L1_error:;
  __Pyx_XDECREF(__pyx_t_1);
  __Pyx_AddTraceback("model.Bondarenko.t_xfer.__get__", __pyx_clineno, __pyx_lineno, __pyx_filename);
  __pyx_r = NULL;
  __pyx_L0:;
  __Pyx_XGIVEREF(__pyx_r);
  __Pyx_RefNannyFinishContext();
  return __pyx_r;
}

/* Python wrapper */
static int __pyx_pw_5model_10Bondarenko_6t_xfer_3__set__(PyObject *__pyx_v_self, PyObject *__pyx_v_value); /*proto*/
static int __pyx_pw_5model_10Bondarenko_6t_xfer_3__set__(PyObject *__pyx_v_self, PyObject *__pyx_v_value) {
  int __pyx_r;
  __Pyx_RefNannyDeclarations
  __Pyx_RefNannySetupContext("__set__ (wrapper)", 0);
  __pyx_r = __pyx_pf_5model_10Bondarenko_6t_xfer_2__set__(((struct __pyx_obj_5model_Bondarenko *)__pyx_v_self), ((PyObject *)__pyx_v_value));

  /* function exit code */
  __Pyx_RefNannyFinishContext();
  return __pyx_r;
}

static int __pyx_pf_5model_10Bondarenko_6t_xfer_2__set__(struct __pyx_obj_5model_Bondarenko *__pyx_v_self, PyObject *__pyx_v_value) {
  int __pyx_r;
  __Pyx_RefNannyDeclarations
  __Pyx_RefNannySetupContext("__set__", 0);
  __pyx_t_1 = __pyx_PyFloat_AsDouble(__pyx_v_value); if (unlikely((__pyx_t_1 == (double)-1) && PyErr_Occurred())) {__pyx_filename = __pyx_f[0]; __pyx_lineno = 34; __pyx_clineno = __LINE__; goto __pyx_L1_error;}
  __pyx_v_self->t_xfer = __pyx_t_1;

  /* function exit code */
  __pyx_r = 0;
  goto __pyx_L0;
  __pyx_L1_error:;
  __Pyx_AddTraceback("model.Bondarenko.t_xfer.__set__", __pyx_clineno, __pyx_lineno, __pyx_filename);
  __pyx_r = -1;
  __pyx_L0:;
  __Pyx_RefNannyFinishContext();
  return __pyx_r;
}
```

```
 35:     cdef public double k_p_a
```

```
/* Python wrapper */
static PyObject *__pyx_pw_5model_10Bondarenko_5k_p_a_1__get__(PyObject *__pyx_v_self); /*proto*/
static PyObject *__pyx_pw_5model_10Bondarenko_5k_p_a_1__get__(PyObject *__pyx_v_self) {
  PyObject *__pyx_r = 0;
  __Pyx_RefNannyDeclarations
  __Pyx_RefNannySetupContext("__get__ (wrapper)", 0);
  __pyx_r = __pyx_pf_5model_10Bondarenko_5k_p_a___get__(((struct __pyx_obj_5model_Bondarenko *)__pyx_v_self));

  /* function exit code */
  __Pyx_RefNannyFinishContext();
  return __pyx_r;
}

static PyObject *__pyx_pf_5model_10Bondarenko_5k_p_a___get__(struct __pyx_obj_5model_Bondarenko *__pyx_v_self) {
  PyObject *__pyx_r = NULL;
  __Pyx_RefNannyDeclarations
  __Pyx_RefNannySetupContext("__get__", 0);
  __Pyx_XDECREF(__pyx_r);
  __pyx_t_1 = PyFloat_FromDouble(__pyx_v_self->k_p_a); if (unlikely(!__pyx_t_1)) {__pyx_filename = __pyx_f[0]; __pyx_lineno = 35; __pyx_clineno = __LINE__; goto __pyx_L1_error;}
  __Pyx_GOTREF(__pyx_t_1);
  __pyx_r = __pyx_t_1;
  __pyx_t_1 = 0;
  goto __pyx_L0;

  /* function exit code */
  __pyx_L1_error:;
  __Pyx_XDECREF(__pyx_t_1);
  __Pyx_AddTraceback("model.Bondarenko.k_p_a.__get__", __pyx_clineno, __pyx_lineno, __pyx_filename);
  __pyx_r = NULL;
  __pyx_L0:;
  __Pyx_XGIVEREF(__pyx_r);
  __Pyx_RefNannyFinishContext();
  return __pyx_r;
}

/* Python wrapper */
static int __pyx_pw_5model_10Bondarenko_5k_p_a_3__set__(PyObject *__pyx_v_self, PyObject *__pyx_v_value); /*proto*/
static int __pyx_pw_5model_10Bondarenko_5k_p_a_3__set__(PyObject *__pyx_v_self, PyObject *__pyx_v_value) {
  int __pyx_r;
  __Pyx_RefNannyDeclarations
  __Pyx_RefNannySetupContext("__set__ (wrapper)", 0);
  __pyx_r = __pyx_pf_5model_10Bondarenko_5k_p_a_2__set__(((struct __pyx_obj_5model_Bondarenko *)__pyx_v_self), ((PyObject *)__pyx_v_value));

  /* function exit code */
  __Pyx_RefNannyFinishContext();
  return __pyx_r;
}

static int __pyx_pf_5model_10Bondarenko_5k_p_a_2__set__(struct __pyx_obj_5model_Bondarenko *__pyx_v_self, PyObject *__pyx_v_value) {
  int __pyx_r;
  __Pyx_RefNannyDeclarations
  __Pyx_RefNannySetupContext("__set__", 0);
  __pyx_t_1 = __pyx_PyFloat_AsDouble(__pyx_v_value); if (unlikely((__pyx_t_1 == (double)-1) && PyErr_Occurred())) {__pyx_filename = __pyx_f[0]; __pyx_lineno = 35; __pyx_clineno = __LINE__; goto __pyx_L1_error;}
  __pyx_v_self->k_p_a = __pyx_t_1;

  /* function exit code */
  __pyx_r = 0;
  goto __pyx_L0;
  __pyx_L1_error:;
  __Pyx_AddTraceback("model.Bondarenko.k_p_a.__set__", __pyx_clineno, __pyx_lineno, __pyx_filename);
  __pyx_r = -1;
  __pyx_L0:;
  __Pyx_RefNannyFinishContext();
  return __pyx_r;
}
```

```
 36:     cdef public double k_n_a
```

```
/* Python wrapper */
static PyObject *__pyx_pw_5model_10Bondarenko_5k_n_a_1__get__(PyObject *__pyx_v_self); /*proto*/
static PyObject *__pyx_pw_5model_10Bondarenko_5k_n_a_1__get__(PyObject *__pyx_v_self) {
  PyObject *__pyx_r = 0;
  __Pyx_RefNannyDeclarations
  __Pyx_RefNannySetupContext("__get__ (wrapper)", 0);
  __pyx_r = __pyx_pf_5model_10Bondarenko_5k_n_a___get__(((struct __pyx_obj_5model_Bondarenko *)__pyx_v_self));

  /* function exit code */
  __Pyx_RefNannyFinishContext();
  return __pyx_r;
}

static PyObject *__pyx_pf_5model_10Bondarenko_5k_n_a___get__(struct __pyx_obj_5model_Bondarenko *__pyx_v_self) {
  PyObject *__pyx_r = NULL;
  __Pyx_RefNannyDeclarations
  __Pyx_RefNannySetupContext("__get__", 0);
  __Pyx_XDECREF(__pyx_r);
  __pyx_t_1 = PyFloat_FromDouble(__pyx_v_self->k_n_a); if (unlikely(!__pyx_t_1)) {__pyx_filename = __pyx_f[0]; __pyx_lineno = 36; __pyx_clineno = __LINE__; goto __pyx_L1_error;}
  __Pyx_GOTREF(__pyx_t_1);
  __pyx_r = __pyx_t_1;
  __pyx_t_1 = 0;
  goto __pyx_L0;

  /* function exit code */
  __pyx_L1_error:;
  __Pyx_XDECREF(__pyx_t_1);
  __Pyx_AddTraceback("model.Bondarenko.k_n_a.__get__", __pyx_clineno, __pyx_lineno, __pyx_filename);
  __pyx_r = NULL;
  __pyx_L0:;
  __Pyx_XGIVEREF(__pyx_r);
  __Pyx_RefNannyFinishContext();
  return __pyx_r;
}

/* Python wrapper */
static int __pyx_pw_5model_10Bondarenko_5k_n_a_3__set__(PyObject *__pyx_v_self, PyObject *__pyx_v_value); /*proto*/
static int __pyx_pw_5model_10Bondarenko_5k_n_a_3__set__(PyObject *__pyx_v_self, PyObject *__pyx_v_value) {
  int __pyx_r;
  __Pyx_RefNannyDeclarations
  __Pyx_RefNannySetupContext("__set__ (wrapper)", 0);
  __pyx_r = __pyx_pf_5model_10Bondarenko_5k_n_a_2__set__(((struct __pyx_obj_5model_Bondarenko *)__pyx_v_self), ((PyObject *)__pyx_v_value));

  /* function exit code */
  __Pyx_RefNannyFinishContext();
  return __pyx_r;
}

static int __pyx_pf_5model_10Bondarenko_5k_n_a_2__set__(struct __pyx_obj_5model_Bondarenko *__pyx_v_self, PyObject *__pyx_v_value) {
  int __pyx_r;
  __Pyx_RefNannyDeclarations
  __Pyx_RefNannySetupContext("__set__", 0);
  __pyx_t_1 = __pyx_PyFloat_AsDouble(__pyx_v_value); if (unlikely((__pyx_t_1 == (double)-1) && PyErr_Occurred())) {__pyx_filename = __pyx_f[0]; __pyx_lineno = 36; __pyx_clineno = __LINE__; goto __pyx_L1_error;}
  __pyx_v_self->k_n_a = __pyx_t_1;

  /* function exit code */
  __pyx_r = 0;
  goto __pyx_L0;
  __pyx_L1_error:;
  __Pyx_AddTraceback("model.Bondarenko.k_n_a.__set__", __pyx_clineno, __pyx_lineno, __pyx_filename);
  __pyx_r = -1;
  __pyx_L0:;
  __Pyx_RefNannyFinishContext();
  return __pyx_r;
}
```

```
 37:     cdef public double k_p_b
```

```
/* Python wrapper */
static PyObject *__pyx_pw_5model_10Bondarenko_5k_p_b_1__get__(PyObject *__pyx_v_self); /*proto*/
static PyObject *__pyx_pw_5model_10Bondarenko_5k_p_b_1__get__(PyObject *__pyx_v_self) {
  PyObject *__pyx_r = 0;
  __Pyx_RefNannyDeclarations
  __Pyx_RefNannySetupContext("__get__ (wrapper)", 0);
  __pyx_r = __pyx_pf_5model_10Bondarenko_5k_p_b___get__(((struct __pyx_obj_5model_Bondarenko *)__pyx_v_self));

  /* function exit code */
  __Pyx_RefNannyFinishContext();
  return __pyx_r;
}

static PyObject *__pyx_pf_5model_10Bondarenko_5k_p_b___get__(struct __pyx_obj_5model_Bondarenko *__pyx_v_self) {
  PyObject *__pyx_r = NULL;
  __Pyx_RefNannyDeclarations
  __Pyx_RefNannySetupContext("__get__", 0);
  __Pyx_XDECREF(__pyx_r);
  __pyx_t_1 = PyFloat_FromDouble(__pyx_v_self->k_p_b); if (unlikely(!__pyx_t_1)) {__pyx_filename = __pyx_f[0]; __pyx_lineno = 37; __pyx_clineno = __LINE__; goto __pyx_L1_error;}
  __Pyx_GOTREF(__pyx_t_1);
  __pyx_r = __pyx_t_1;
  __pyx_t_1 = 0;
  goto __pyx_L0;

  /* function exit code */
  __pyx_L1_error:;
  __Pyx_XDECREF(__pyx_t_1);
  __Pyx_AddTraceback("model.Bondarenko.k_p_b.__get__", __pyx_clineno, __pyx_lineno, __pyx_filename);
  __pyx_r = NULL;
  __pyx_L0:;
  __Pyx_XGIVEREF(__pyx_r);
  __Pyx_RefNannyFinishContext();
  return __pyx_r;
}

/* Python wrapper */
static int __pyx_pw_5model_10Bondarenko_5k_p_b_3__set__(PyObject *__pyx_v_self, PyObject *__pyx_v_value); /*proto*/
static int __pyx_pw_5model_10Bondarenko_5k_p_b_3__set__(PyObject *__pyx_v_self, PyObject *__pyx_v_value) {
  int __pyx_r;
  __Pyx_RefNannyDeclarations
  __Pyx_RefNannySetupContext("__set__ (wrapper)", 0);
  __pyx_r = __pyx_pf_5model_10Bondarenko_5k_p_b_2__set__(((struct __pyx_obj_5model_Bondarenko *)__pyx_v_self), ((PyObject *)__pyx_v_value));

  /* function exit code */
  __Pyx_RefNannyFinishContext();
  return __pyx_r;
}

static int __pyx_pf_5model_10Bondarenko_5k_p_b_2__set__(struct __pyx_obj_5model_Bondarenko *__pyx_v_self, PyObject *__pyx_v_value) {
  int __pyx_r;
  __Pyx_RefNannyDeclarations
  __Pyx_RefNannySetupContext("__set__", 0);
  __pyx_t_1 = __pyx_PyFloat_AsDouble(__pyx_v_value); if (unlikely((__pyx_t_1 == (double)-1) && PyErr_Occurred())) {__pyx_filename = __pyx_f[0]; __pyx_lineno = 37; __pyx_clineno = __LINE__; goto __pyx_L1_error;}
  __pyx_v_self->k_p_b = __pyx_t_1;

  /* function exit code */
  __pyx_r = 0;
  goto __pyx_L0;
  __pyx_L1_error:;
  __Pyx_AddTraceback("model.Bondarenko.k_p_b.__set__", __pyx_clineno, __pyx_lineno, __pyx_filename);
  __pyx_r = -1;
  __pyx_L0:;
  __Pyx_RefNannyFinishContext();
  return __pyx_r;
}
```

```
 38:     cdef public double k_n_b
```

```
/* Python wrapper */
static PyObject *__pyx_pw_5model_10Bondarenko_5k_n_b_1__get__(PyObject *__pyx_v_self); /*proto*/
static PyObject *__pyx_pw_5model_10Bondarenko_5k_n_b_1__get__(PyObject *__pyx_v_self) {
  PyObject *__pyx_r = 0;
  __Pyx_RefNannyDeclarations
  __Pyx_RefNannySetupContext("__get__ (wrapper)", 0);
  __pyx_r = __pyx_pf_5model_10Bondarenko_5k_n_b___get__(((struct __pyx_obj_5model_Bondarenko *)__pyx_v_self));

  /* function exit code */
  __Pyx_RefNannyFinishContext();
  return __pyx_r;
}

static PyObject *__pyx_pf_5model_10Bondarenko_5k_n_b___get__(struct __pyx_obj_5model_Bondarenko *__pyx_v_self) {
  PyObject *__pyx_r = NULL;
  __Pyx_RefNannyDeclarations
  __Pyx_RefNannySetupContext("__get__", 0);
  __Pyx_XDECREF(__pyx_r);
  __pyx_t_1 = PyFloat_FromDouble(__pyx_v_self->k_n_b); if (unlikely(!__pyx_t_1)) {__pyx_filename = __pyx_f[0]; __pyx_lineno = 38; __pyx_clineno = __LINE__; goto __pyx_L1_error;}
  __Pyx_GOTREF(__pyx_t_1);
  __pyx_r = __pyx_t_1;
  __pyx_t_1 = 0;
  goto __pyx_L0;

  /* function exit code */
  __pyx_L1_error:;
  __Pyx_XDECREF(__pyx_t_1);
  __Pyx_AddTraceback("model.Bondarenko.k_n_b.__get__", __pyx_clineno, __pyx_lineno, __pyx_filename);
  __pyx_r = NULL;
  __pyx_L0:;
  __Pyx_XGIVEREF(__pyx_r);
  __Pyx_RefNannyFinishContext();
  return __pyx_r;
}

/* Python wrapper */
static int __pyx_pw_5model_10Bondarenko_5k_n_b_3__set__(PyObject *__pyx_v_self, PyObject *__pyx_v_value); /*proto*/
static int __pyx_pw_5model_10Bondarenko_5k_n_b_3__set__(PyObject *__pyx_v_self, PyObject *__pyx_v_value) {
  int __pyx_r;
  __Pyx_RefNannyDeclarations
  __Pyx_RefNannySetupContext("__set__ (wrapper)", 0);
  __pyx_r = __pyx_pf_5model_10Bondarenko_5k_n_b_2__set__(((struct __pyx_obj_5model_Bondarenko *)__pyx_v_self), ((PyObject *)__pyx_v_value));

  /* function exit code */
  __Pyx_RefNannyFinishContext();
  return __pyx_r;
}

static int __pyx_pf_5model_10Bondarenko_5k_n_b_2__set__(struct __pyx_obj_5model_Bondarenko *__pyx_v_self, PyObject *__pyx_v_value) {
  int __pyx_r;
  __Pyx_RefNannyDeclarations
  __Pyx_RefNannySetupContext("__set__", 0);
  __pyx_t_1 = __pyx_PyFloat_AsDouble(__pyx_v_value); if (unlikely((__pyx_t_1 == (double)-1) && PyErr_Occurred())) {__pyx_filename = __pyx_f[0]; __pyx_lineno = 38; __pyx_clineno = __LINE__; goto __pyx_L1_error;}
  __pyx_v_self->k_n_b = __pyx_t_1;

  /* function exit code */
  __pyx_r = 0;
  goto __pyx_L0;
  __pyx_L1_error:;
  __Pyx_AddTraceback("model.Bondarenko.k_n_b.__set__", __pyx_clineno, __pyx_lineno, __pyx_filename);
  __pyx_r = -1;
  __pyx_L0:;
  __Pyx_RefNannyFinishContext();
  return __pyx_r;
}
```

```
 39:     cdef public double k_p_c
```

```
/* Python wrapper */
static PyObject *__pyx_pw_5model_10Bondarenko_5k_p_c_1__get__(PyObject *__pyx_v_self); /*proto*/
static PyObject *__pyx_pw_5model_10Bondarenko_5k_p_c_1__get__(PyObject *__pyx_v_self) {
  PyObject *__pyx_r = 0;
  __Pyx_RefNannyDeclarations
  __Pyx_RefNannySetupContext("__get__ (wrapper)", 0);
  __pyx_r = __pyx_pf_5model_10Bondarenko_5k_p_c___get__(((struct __pyx_obj_5model_Bondarenko *)__pyx_v_self));

  /* function exit code */
  __Pyx_RefNannyFinishContext();
  return __pyx_r;
}

static PyObject *__pyx_pf_5model_10Bondarenko_5k_p_c___get__(struct __pyx_obj_5model_Bondarenko *__pyx_v_self) {
  PyObject *__pyx_r = NULL;
  __Pyx_RefNannyDeclarations
  __Pyx_RefNannySetupContext("__get__", 0);
  __Pyx_XDECREF(__pyx_r);
  __pyx_t_1 = PyFloat_FromDouble(__pyx_v_self->k_p_c); if (unlikely(!__pyx_t_1)) {__pyx_filename = __pyx_f[0]; __pyx_lineno = 39; __pyx_clineno = __LINE__; goto __pyx_L1_error;}
  __Pyx_GOTREF(__pyx_t_1);
  __pyx_r = __pyx_t_1;
  __pyx_t_1 = 0;
  goto __pyx_L0;

  /* function exit code */
  __pyx_L1_error:;
  __Pyx_XDECREF(__pyx_t_1);
  __Pyx_AddTraceback("model.Bondarenko.k_p_c.__get__", __pyx_clineno, __pyx_lineno, __pyx_filename);
  __pyx_r = NULL;
  __pyx_L0:;
  __Pyx_XGIVEREF(__pyx_r);
  __Pyx_RefNannyFinishContext();
  return __pyx_r;
}

/* Python wrapper */
static int __pyx_pw_5model_10Bondarenko_5k_p_c_3__set__(PyObject *__pyx_v_self, PyObject *__pyx_v_value); /*proto*/
static int __pyx_pw_5model_10Bondarenko_5k_p_c_3__set__(PyObject *__pyx_v_self, PyObject *__pyx_v_value) {
  int __pyx_r;
  __Pyx_RefNannyDeclarations
  __Pyx_RefNannySetupContext("__set__ (wrapper)", 0);
  __pyx_r = __pyx_pf_5model_10Bondarenko_5k_p_c_2__set__(((struct __pyx_obj_5model_Bondarenko *)__pyx_v_self), ((PyObject *)__pyx_v_value));

  /* function exit code */
  __Pyx_RefNannyFinishContext();
  return __pyx_r;
}

static int __pyx_pf_5model_10Bondarenko_5k_p_c_2__set__(struct __pyx_obj_5model_Bondarenko *__pyx_v_self, PyObject *__pyx_v_value) {
  int __pyx_r;
  __Pyx_RefNannyDeclarations
  __Pyx_RefNannySetupContext("__set__", 0);
  __pyx_t_1 = __pyx_PyFloat_AsDouble(__pyx_v_value); if (unlikely((__pyx_t_1 == (double)-1) && PyErr_Occurred())) {__pyx_filename = __pyx_f[0]; __pyx_lineno = 39; __pyx_clineno = __LINE__; goto __pyx_L1_error;}
  __pyx_v_self->k_p_c = __pyx_t_1;

  /* function exit code */
  __pyx_r = 0;
  goto __pyx_L0;
  __pyx_L1_error:;
  __Pyx_AddTraceback("model.Bondarenko.k_p_c.__set__", __pyx_clineno, __pyx_lineno, __pyx_filename);
  __pyx_r = -1;
  __pyx_L0:;
  __Pyx_RefNannyFinishContext();
  return __pyx_r;
}
```

```
 40:     cdef public double k_n_c
```

```
/* Python wrapper */
static PyObject *__pyx_pw_5model_10Bondarenko_5k_n_c_1__get__(PyObject *__pyx_v_self); /*proto*/
static PyObject *__pyx_pw_5model_10Bondarenko_5k_n_c_1__get__(PyObject *__pyx_v_self) {
  PyObject *__pyx_r = 0;
  __Pyx_RefNannyDeclarations
  __Pyx_RefNannySetupContext("__get__ (wrapper)", 0);
  __pyx_r = __pyx_pf_5model_10Bondarenko_5k_n_c___get__(((struct __pyx_obj_5model_Bondarenko *)__pyx_v_self));

  /* function exit code */
  __Pyx_RefNannyFinishContext();
  return __pyx_r;
}

static PyObject *__pyx_pf_5model_10Bondarenko_5k_n_c___get__(struct __pyx_obj_5model_Bondarenko *__pyx_v_self) {
  PyObject *__pyx_r = NULL;
  __Pyx_RefNannyDeclarations
  __Pyx_RefNannySetupContext("__get__", 0);
  __Pyx_XDECREF(__pyx_r);
  __pyx_t_1 = PyFloat_FromDouble(__pyx_v_self->k_n_c); if (unlikely(!__pyx_t_1)) {__pyx_filename = __pyx_f[0]; __pyx_lineno = 40; __pyx_clineno = __LINE__; goto __pyx_L1_error;}
  __Pyx_GOTREF(__pyx_t_1);
  __pyx_r = __pyx_t_1;
  __pyx_t_1 = 0;
  goto __pyx_L0;

  /* function exit code */
  __pyx_L1_error:;
  __Pyx_XDECREF(__pyx_t_1);
  __Pyx_AddTraceback("model.Bondarenko.k_n_c.__get__", __pyx_clineno, __pyx_lineno, __pyx_filename);
  __pyx_r = NULL;
  __pyx_L0:;
  __Pyx_XGIVEREF(__pyx_r);
  __Pyx_RefNannyFinishContext();
  return __pyx_r;
}

/* Python wrapper */
static int __pyx_pw_5model_10Bondarenko_5k_n_c_3__set__(PyObject *__pyx_v_self, PyObject *__pyx_v_value); /*proto*/
static int __pyx_pw_5model_10Bondarenko_5k_n_c_3__set__(PyObject *__pyx_v_self, PyObject *__pyx_v_value) {
  int __pyx_r;
  __Pyx_RefNannyDeclarations
  __Pyx_RefNannySetupContext("__set__ (wrapper)", 0);
  __pyx_r = __pyx_pf_5model_10Bondarenko_5k_n_c_2__set__(((struct __pyx_obj_5model_Bondarenko *)__pyx_v_self), ((PyObject *)__pyx_v_value));

  /* function exit code */
  __Pyx_RefNannyFinishContext();
  return __pyx_r;
}

static int __pyx_pf_5model_10Bondarenko_5k_n_c_2__set__(struct __pyx_obj_5model_Bondarenko *__pyx_v_self, PyObject *__pyx_v_value) {
  int __pyx_r;
  __Pyx_RefNannyDeclarations
  __Pyx_RefNannySetupContext("__set__", 0);
  __pyx_t_1 = __pyx_PyFloat_AsDouble(__pyx_v_value); if (unlikely((__pyx_t_1 == (double)-1) && PyErr_Occurred())) {__pyx_filename = __pyx_f[0]; __pyx_lineno = 40; __pyx_clineno = __LINE__; goto __pyx_L1_error;}
  __pyx_v_self->k_n_c = __pyx_t_1;

  /* function exit code */
  __pyx_r = 0;
  goto __pyx_L0;
  __pyx_L1_error:;
  __Pyx_AddTraceback("model.Bondarenko.k_n_c.__set__", __pyx_clineno, __pyx_lineno, __pyx_filename);
  __pyx_r = -1;
  __pyx_L0:;
  __Pyx_RefNannyFinishContext();
  return __pyx_r;
}
```

```
 41:     cdef public double n
```

```
/* Python wrapper */
static PyObject *__pyx_pw_5model_10Bondarenko_1n_1__get__(PyObject *__pyx_v_self); /*proto*/
static PyObject *__pyx_pw_5model_10Bondarenko_1n_1__get__(PyObject *__pyx_v_self) {
  PyObject *__pyx_r = 0;
  __Pyx_RefNannyDeclarations
  __Pyx_RefNannySetupContext("__get__ (wrapper)", 0);
  __pyx_r = __pyx_pf_5model_10Bondarenko_1n___get__(((struct __pyx_obj_5model_Bondarenko *)__pyx_v_self));

  /* function exit code */
  __Pyx_RefNannyFinishContext();
  return __pyx_r;
}

static PyObject *__pyx_pf_5model_10Bondarenko_1n___get__(struct __pyx_obj_5model_Bondarenko *__pyx_v_self) {
  PyObject *__pyx_r = NULL;
  __Pyx_RefNannyDeclarations
  __Pyx_RefNannySetupContext("__get__", 0);
  __Pyx_XDECREF(__pyx_r);
  __pyx_t_1 = PyFloat_FromDouble(__pyx_v_self->n); if (unlikely(!__pyx_t_1)) {__pyx_filename = __pyx_f[0]; __pyx_lineno = 41; __pyx_clineno = __LINE__; goto __pyx_L1_error;}
  __Pyx_GOTREF(__pyx_t_1);
  __pyx_r = __pyx_t_1;
  __pyx_t_1 = 0;
  goto __pyx_L0;

  /* function exit code */
  __pyx_L1_error:;
  __Pyx_XDECREF(__pyx_t_1);
  __Pyx_AddTraceback("model.Bondarenko.n.__get__", __pyx_clineno, __pyx_lineno, __pyx_filename);
  __pyx_r = NULL;
  __pyx_L0:;
  __Pyx_XGIVEREF(__pyx_r);
  __Pyx_RefNannyFinishContext();
  return __pyx_r;
}

/* Python wrapper */
static int __pyx_pw_5model_10Bondarenko_1n_3__set__(PyObject *__pyx_v_self, PyObject *__pyx_v_value); /*proto*/
static int __pyx_pw_5model_10Bondarenko_1n_3__set__(PyObject *__pyx_v_self, PyObject *__pyx_v_value) {
  int __pyx_r;
  __Pyx_RefNannyDeclarations
  __Pyx_RefNannySetupContext("__set__ (wrapper)", 0);
  __pyx_r = __pyx_pf_5model_10Bondarenko_1n_2__set__(((struct __pyx_obj_5model_Bondarenko *)__pyx_v_self), ((PyObject *)__pyx_v_value));

  /* function exit code */
  __Pyx_RefNannyFinishContext();
  return __pyx_r;
}

static int __pyx_pf_5model_10Bondarenko_1n_2__set__(struct __pyx_obj_5model_Bondarenko *__pyx_v_self, PyObject *__pyx_v_value) {
  int __pyx_r;
  __Pyx_RefNannyDeclarations
  __Pyx_RefNannySetupContext("__set__", 0);
  __pyx_t_1 = __pyx_PyFloat_AsDouble(__pyx_v_value); if (unlikely((__pyx_t_1 == (double)-1) && PyErr_Occurred())) {__pyx_filename = __pyx_f[0]; __pyx_lineno = 41; __pyx_clineno = __LINE__; goto __pyx_L1_error;}
  __pyx_v_self->n = __pyx_t_1;

  /* function exit code */
  __pyx_r = 0;
  goto __pyx_L0;
  __pyx_L1_error:;
  __Pyx_AddTraceback("model.Bondarenko.n.__set__", __pyx_clineno, __pyx_lineno, __pyx_filename);
  __pyx_r = -1;
  __pyx_L0:;
  __Pyx_RefNannyFinishContext();
  return __pyx_r;
}
```

```
 42:     cdef public double m
```

```
/* Python wrapper */
static PyObject *__pyx_pw_5model_10Bondarenko_1m_1__get__(PyObject *__pyx_v_self); /*proto*/
static PyObject *__pyx_pw_5model_10Bondarenko_1m_1__get__(PyObject *__pyx_v_self) {
  PyObject *__pyx_r = 0;
  __Pyx_RefNannyDeclarations
  __Pyx_RefNannySetupContext("__get__ (wrapper)", 0);
  __pyx_r = __pyx_pf_5model_10Bondarenko_1m___get__(((struct __pyx_obj_5model_Bondarenko *)__pyx_v_self));

  /* function exit code */
  __Pyx_RefNannyFinishContext();
  return __pyx_r;
}

static PyObject *__pyx_pf_5model_10Bondarenko_1m___get__(struct __pyx_obj_5model_Bondarenko *__pyx_v_self) {
  PyObject *__pyx_r = NULL;
  __Pyx_RefNannyDeclarations
  __Pyx_RefNannySetupContext("__get__", 0);
  __Pyx_XDECREF(__pyx_r);
  __pyx_t_1 = PyFloat_FromDouble(__pyx_v_self->m); if (unlikely(!__pyx_t_1)) {__pyx_filename = __pyx_f[0]; __pyx_lineno = 42; __pyx_clineno = __LINE__; goto __pyx_L1_error;}
  __Pyx_GOTREF(__pyx_t_1);
  __pyx_r = __pyx_t_1;
  __pyx_t_1 = 0;
  goto __pyx_L0;

  /* function exit code */
  __pyx_L1_error:;
  __Pyx_XDECREF(__pyx_t_1);
  __Pyx_AddTraceback("model.Bondarenko.m.__get__", __pyx_clineno, __pyx_lineno, __pyx_filename);
  __pyx_r = NULL;
  __pyx_L0:;
  __Pyx_XGIVEREF(__pyx_r);
  __Pyx_RefNannyFinishContext();
  return __pyx_r;
}

/* Python wrapper */
static int __pyx_pw_5model_10Bondarenko_1m_3__set__(PyObject *__pyx_v_self, PyObject *__pyx_v_value); /*proto*/
static int __pyx_pw_5model_10Bondarenko_1m_3__set__(PyObject *__pyx_v_self, PyObject *__pyx_v_value) {
  int __pyx_r;
  __Pyx_RefNannyDeclarations
  __Pyx_RefNannySetupContext("__set__ (wrapper)", 0);
  __pyx_r = __pyx_pf_5model_10Bondarenko_1m_2__set__(((struct __pyx_obj_5model_Bondarenko *)__pyx_v_self), ((PyObject *)__pyx_v_value));

  /* function exit code */
  __Pyx_RefNannyFinishContext();
  return __pyx_r;
}

static int __pyx_pf_5model_10Bondarenko_1m_2__set__(struct __pyx_obj_5model_Bondarenko *__pyx_v_self, PyObject *__pyx_v_value) {
  int __pyx_r;
  __Pyx_RefNannyDeclarations
  __Pyx_RefNannySetupContext("__set__", 0);
  __pyx_t_1 = __pyx_PyFloat_AsDouble(__pyx_v_value); if (unlikely((__pyx_t_1 == (double)-1) && PyErr_Occurred())) {__pyx_filename = __pyx_f[0]; __pyx_lineno = 42; __pyx_clineno = __LINE__; goto __pyx_L1_error;}
  __pyx_v_self->m = __pyx_t_1;

  /* function exit code */
  __pyx_r = 0;
  goto __pyx_L0;
  __pyx_L1_error:;
  __Pyx_AddTraceback("model.Bondarenko.m.__set__", __pyx_clineno, __pyx_lineno, __pyx_filename);
  __pyx_r = -1;
  __pyx_L0:;
  __Pyx_RefNannyFinishContext();
  return __pyx_r;
}
```

```
 43:     cdef public double G_CaL
```

```
/* Python wrapper */
static PyObject *__pyx_pw_5model_10Bondarenko_5G_CaL_1__get__(PyObject *__pyx_v_self); /*proto*/
static PyObject *__pyx_pw_5model_10Bondarenko_5G_CaL_1__get__(PyObject *__pyx_v_self) {
  PyObject *__pyx_r = 0;
  __Pyx_RefNannyDeclarations
  __Pyx_RefNannySetupContext("__get__ (wrapper)", 0);
  __pyx_r = __pyx_pf_5model_10Bondarenko_5G_CaL___get__(((struct __pyx_obj_5model_Bondarenko *)__pyx_v_self));

  /* function exit code */
  __Pyx_RefNannyFinishContext();
  return __pyx_r;
}

static PyObject *__pyx_pf_5model_10Bondarenko_5G_CaL___get__(struct __pyx_obj_5model_Bondarenko *__pyx_v_self) {
  PyObject *__pyx_r = NULL;
  __Pyx_RefNannyDeclarations
  __Pyx_RefNannySetupContext("__get__", 0);
  __Pyx_XDECREF(__pyx_r);
  __pyx_t_1 = PyFloat_FromDouble(__pyx_v_self->G_CaL); if (unlikely(!__pyx_t_1)) {__pyx_filename = __pyx_f[0]; __pyx_lineno = 43; __pyx_clineno = __LINE__; goto __pyx_L1_error;}
  __Pyx_GOTREF(__pyx_t_1);
  __pyx_r = __pyx_t_1;
  __pyx_t_1 = 0;
  goto __pyx_L0;

  /* function exit code */
  __pyx_L1_error:;
  __Pyx_XDECREF(__pyx_t_1);
  __Pyx_AddTraceback("model.Bondarenko.G_CaL.__get__", __pyx_clineno, __pyx_lineno, __pyx_filename);
  __pyx_r = NULL;
  __pyx_L0:;
  __Pyx_XGIVEREF(__pyx_r);
  __Pyx_RefNannyFinishContext();
  return __pyx_r;
}

/* Python wrapper */
static int __pyx_pw_5model_10Bondarenko_5G_CaL_3__set__(PyObject *__pyx_v_self, PyObject *__pyx_v_value); /*proto*/
static int __pyx_pw_5model_10Bondarenko_5G_CaL_3__set__(PyObject *__pyx_v_self, PyObject *__pyx_v_value) {
  int __pyx_r;
  __Pyx_RefNannyDeclarations
  __Pyx_RefNannySetupContext("__set__ (wrapper)", 0);
  __pyx_r = __pyx_pf_5model_10Bondarenko_5G_CaL_2__set__(((struct __pyx_obj_5model_Bondarenko *)__pyx_v_self), ((PyObject *)__pyx_v_value));

  /* function exit code */
  __Pyx_RefNannyFinishContext();
  return __pyx_r;
}

static int __pyx_pf_5model_10Bondarenko_5G_CaL_2__set__(struct __pyx_obj_5model_Bondarenko *__pyx_v_self, PyObject *__pyx_v_value) {
  int __pyx_r;
  __Pyx_RefNannyDeclarations
  __Pyx_RefNannySetupContext("__set__", 0);
  __pyx_t_1 = __pyx_PyFloat_AsDouble(__pyx_v_value); if (unlikely((__pyx_t_1 == (double)-1) && PyErr_Occurred())) {__pyx_filename = __pyx_f[0]; __pyx_lineno = 43; __pyx_clineno = __LINE__; goto __pyx_L1_error;}
  __pyx_v_self->G_CaL = __pyx_t_1;

  /* function exit code */
  __pyx_r = 0;
  goto __pyx_L0;
  __pyx_L1_error:;
  __Pyx_AddTraceback("model.Bondarenko.G_CaL.__set__", __pyx_clineno, __pyx_lineno, __pyx_filename);
  __pyx_r = -1;
  __pyx_L0:;
  __Pyx_RefNannyFinishContext();
  return __pyx_r;
}
```

```
 44:     cdef public double E_CaL
```

```
/* Python wrapper */
static PyObject *__pyx_pw_5model_10Bondarenko_5E_CaL_1__get__(PyObject *__pyx_v_self); /*proto*/
static PyObject *__pyx_pw_5model_10Bondarenko_5E_CaL_1__get__(PyObject *__pyx_v_self) {
  PyObject *__pyx_r = 0;
  __Pyx_RefNannyDeclarations
  __Pyx_RefNannySetupContext("__get__ (wrapper)", 0);
  __pyx_r = __pyx_pf_5model_10Bondarenko_5E_CaL___get__(((struct __pyx_obj_5model_Bondarenko *)__pyx_v_self));

  /* function exit code */
  __Pyx_RefNannyFinishContext();
  return __pyx_r;
}

static PyObject *__pyx_pf_5model_10Bondarenko_5E_CaL___get__(struct __pyx_obj_5model_Bondarenko *__pyx_v_self) {
  PyObject *__pyx_r = NULL;
  __Pyx_RefNannyDeclarations
  __Pyx_RefNannySetupContext("__get__", 0);
  __Pyx_XDECREF(__pyx_r);
  __pyx_t_1 = PyFloat_FromDouble(__pyx_v_self->E_CaL); if (unlikely(!__pyx_t_1)) {__pyx_filename = __pyx_f[0]; __pyx_lineno = 44; __pyx_clineno = __LINE__; goto __pyx_L1_error;}
  __Pyx_GOTREF(__pyx_t_1);
  __pyx_r = __pyx_t_1;
  __pyx_t_1 = 0;
  goto __pyx_L0;

  /* function exit code */
  __pyx_L1_error:;
  __Pyx_XDECREF(__pyx_t_1);
  __Pyx_AddTraceback("model.Bondarenko.E_CaL.__get__", __pyx_clineno, __pyx_lineno, __pyx_filename);
  __pyx_r = NULL;
  __pyx_L0:;
  __Pyx_XGIVEREF(__pyx_r);
  __Pyx_RefNannyFinishContext();
  return __pyx_r;
}

/* Python wrapper */
static int __pyx_pw_5model_10Bondarenko_5E_CaL_3__set__(PyObject *__pyx_v_self, PyObject *__pyx_v_value); /*proto*/
static int __pyx_pw_5model_10Bondarenko_5E_CaL_3__set__(PyObject *__pyx_v_self, PyObject *__pyx_v_value) {
  int __pyx_r;
  __Pyx_RefNannyDeclarations
  __Pyx_RefNannySetupContext("__set__ (wrapper)", 0);
  __pyx_r = __pyx_pf_5model_10Bondarenko_5E_CaL_2__set__(((struct __pyx_obj_5model_Bondarenko *)__pyx_v_self), ((PyObject *)__pyx_v_value));

  /* function exit code */
  __Pyx_RefNannyFinishContext();
  return __pyx_r;
}

static int __pyx_pf_5model_10Bondarenko_5E_CaL_2__set__(struct __pyx_obj_5model_Bondarenko *__pyx_v_self, PyObject *__pyx_v_value) {
  int __pyx_r;
  __Pyx_RefNannyDeclarations
  __Pyx_RefNannySetupContext("__set__", 0);
  __pyx_t_1 = __pyx_PyFloat_AsDouble(__pyx_v_value); if (unlikely((__pyx_t_1 == (double)-1) && PyErr_Occurred())) {__pyx_filename = __pyx_f[0]; __pyx_lineno = 44; __pyx_clineno = __LINE__; goto __pyx_L1_error;}
  __pyx_v_self->E_CaL = __pyx_t_1;

  /* function exit code */
  __pyx_r = 0;
  goto __pyx_L0;
  __pyx_L1_error:;
  __Pyx_AddTraceback("model.Bondarenko.E_CaL.__set__", __pyx_clineno, __pyx_lineno, __pyx_filename);
  __pyx_r = -1;
  __pyx_L0:;
  __Pyx_RefNannyFinishContext();
  return __pyx_r;
}
```

```
 45:     cdef public double k_pcmax
```

```
/* Python wrapper */
static PyObject *__pyx_pw_5model_10Bondarenko_7k_pcmax_1__get__(PyObject *__pyx_v_self); /*proto*/
static PyObject *__pyx_pw_5model_10Bondarenko_7k_pcmax_1__get__(PyObject *__pyx_v_self) {
  PyObject *__pyx_r = 0;
  __Pyx_RefNannyDeclarations
  __Pyx_RefNannySetupContext("__get__ (wrapper)", 0);
  __pyx_r = __pyx_pf_5model_10Bondarenko_7k_pcmax___get__(((struct __pyx_obj_5model_Bondarenko *)__pyx_v_self));

  /* function exit code */
  __Pyx_RefNannyFinishContext();
  return __pyx_r;
}

static PyObject *__pyx_pf_5model_10Bondarenko_7k_pcmax___get__(struct __pyx_obj_5model_Bondarenko *__pyx_v_self) {
  PyObject *__pyx_r = NULL;
  __Pyx_RefNannyDeclarations
  __Pyx_RefNannySetupContext("__get__", 0);
  __Pyx_XDECREF(__pyx_r);
  __pyx_t_1 = PyFloat_FromDouble(__pyx_v_self->k_pcmax); if (unlikely(!__pyx_t_1)) {__pyx_filename = __pyx_f[0]; __pyx_lineno = 45; __pyx_clineno = __LINE__; goto __pyx_L1_error;}
  __Pyx_GOTREF(__pyx_t_1);
  __pyx_r = __pyx_t_1;
  __pyx_t_1 = 0;
  goto __pyx_L0;

  /* function exit code */
  __pyx_L1_error:;
  __Pyx_XDECREF(__pyx_t_1);
  __Pyx_AddTraceback("model.Bondarenko.k_pcmax.__get__", __pyx_clineno, __pyx_lineno, __pyx_filename);
  __pyx_r = NULL;
  __pyx_L0:;
  __Pyx_XGIVEREF(__pyx_r);
  __Pyx_RefNannyFinishContext();
  return __pyx_r;
}

/* Python wrapper */
static int __pyx_pw_5model_10Bondarenko_7k_pcmax_3__set__(PyObject *__pyx_v_self, PyObject *__pyx_v_value); /*proto*/
static int __pyx_pw_5model_10Bondarenko_7k_pcmax_3__set__(PyObject *__pyx_v_self, PyObject *__pyx_v_value) {
  int __pyx_r;
  __Pyx_RefNannyDeclarations
  __Pyx_RefNannySetupContext("__set__ (wrapper)", 0);
  __pyx_r = __pyx_pf_5model_10Bondarenko_7k_pcmax_2__set__(((struct __pyx_obj_5model_Bondarenko *)__pyx_v_self), ((PyObject *)__pyx_v_value));

  /* function exit code */
  __Pyx_RefNannyFinishContext();
  return __pyx_r;
}

static int __pyx_pf_5model_10Bondarenko_7k_pcmax_2__set__(struct __pyx_obj_5model_Bondarenko *__pyx_v_self, PyObject *__pyx_v_value) {
  int __pyx_r;
  __Pyx_RefNannyDeclarations
  __Pyx_RefNannySetupContext("__set__", 0);
  __pyx_t_1 = __pyx_PyFloat_AsDouble(__pyx_v_value); if (unlikely((__pyx_t_1 == (double)-1) && PyErr_Occurred())) {__pyx_filename = __pyx_f[0]; __pyx_lineno = 45; __pyx_clineno = __LINE__; goto __pyx_L1_error;}
  __pyx_v_self->k_pcmax = __pyx_t_1;

  /* function exit code */
  __pyx_r = 0;
  goto __pyx_L0;
  __pyx_L1_error:;
  __Pyx_AddTraceback("model.Bondarenko.k_pcmax.__set__", __pyx_clineno, __pyx_lineno, __pyx_filename);
  __pyx_r = -1;
  __pyx_L0:;
  __Pyx_RefNannyFinishContext();
  return __pyx_r;
}
```

```
 46:     cdef public double k_pchalf
```

```
/* Python wrapper */
static PyObject *__pyx_pw_5model_10Bondarenko_8k_pchalf_1__get__(PyObject *__pyx_v_self); /*proto*/
static PyObject *__pyx_pw_5model_10Bondarenko_8k_pchalf_1__get__(PyObject *__pyx_v_self) {
  PyObject *__pyx_r = 0;
  __Pyx_RefNannyDeclarations
  __Pyx_RefNannySetupContext("__get__ (wrapper)", 0);
  __pyx_r = __pyx_pf_5model_10Bondarenko_8k_pchalf___get__(((struct __pyx_obj_5model_Bondarenko *)__pyx_v_self));

  /* function exit code */
  __Pyx_RefNannyFinishContext();
  return __pyx_r;
}

static PyObject *__pyx_pf_5model_10Bondarenko_8k_pchalf___get__(struct __pyx_obj_5model_Bondarenko *__pyx_v_self) {
  PyObject *__pyx_r = NULL;
  __Pyx_RefNannyDeclarations
  __Pyx_RefNannySetupContext("__get__", 0);
  __Pyx_XDECREF(__pyx_r);
  __pyx_t_1 = PyFloat_FromDouble(__pyx_v_self->k_pchalf); if (unlikely(!__pyx_t_1)) {__pyx_filename = __pyx_f[0]; __pyx_lineno = 46; __pyx_clineno = __LINE__; goto __pyx_L1_error;}
  __Pyx_GOTREF(__pyx_t_1);
  __pyx_r = __pyx_t_1;
  __pyx_t_1 = 0;
  goto __pyx_L0;

  /* function exit code */
  __pyx_L1_error:;
  __Pyx_XDECREF(__pyx_t_1);
  __Pyx_AddTraceback("model.Bondarenko.k_pchalf.__get__", __pyx_clineno, __pyx_lineno, __pyx_filename);
  __pyx_r = NULL;
  __pyx_L0:;
  __Pyx_XGIVEREF(__pyx_r);
  __Pyx_RefNannyFinishContext();
  return __pyx_r;
}

/* Python wrapper */
static int __pyx_pw_5model_10Bondarenko_8k_pchalf_3__set__(PyObject *__pyx_v_self, PyObject *__pyx_v_value); /*proto*/
static int __pyx_pw_5model_10Bondarenko_8k_pchalf_3__set__(PyObject *__pyx_v_self, PyObject *__pyx_v_value) {
  int __pyx_r;
  __Pyx_RefNannyDeclarations
  __Pyx_RefNannySetupContext("__set__ (wrapper)", 0);
  __pyx_r = __pyx_pf_5model_10Bondarenko_8k_pchalf_2__set__(((struct __pyx_obj_5model_Bondarenko *)__pyx_v_self), ((PyObject *)__pyx_v_value));

  /* function exit code */
  __Pyx_RefNannyFinishContext();
  return __pyx_r;
}

static int __pyx_pf_5model_10Bondarenko_8k_pchalf_2__set__(struct __pyx_obj_5model_Bondarenko *__pyx_v_self, PyObject *__pyx_v_value) {
  int __pyx_r;
  __Pyx_RefNannyDeclarations
  __Pyx_RefNannySetupContext("__set__", 0);
  __pyx_t_1 = __pyx_PyFloat_AsDouble(__pyx_v_value); if (unlikely((__pyx_t_1 == (double)-1) && PyErr_Occurred())) {__pyx_filename = __pyx_f[0]; __pyx_lineno = 46; __pyx_clineno = __LINE__; goto __pyx_L1_error;}
  __pyx_v_self->k_pchalf = __pyx_t_1;

  /* function exit code */
  __pyx_r = 0;
  goto __pyx_L0;
  __pyx_L1_error:;
  __Pyx_AddTraceback("model.Bondarenko.k_pchalf.__set__", __pyx_clineno, __pyx_lineno, __pyx_filename);
  __pyx_r = -1;
  __pyx_L0:;
  __Pyx_RefNannyFinishContext();
  return __pyx_r;
}
```

```
 47:     cdef public double k_pcb
```

```
/* Python wrapper */
static PyObject *__pyx_pw_5model_10Bondarenko_5k_pcb_1__get__(PyObject *__pyx_v_self); /*proto*/
static PyObject *__pyx_pw_5model_10Bondarenko_5k_pcb_1__get__(PyObject *__pyx_v_self) {
  PyObject *__pyx_r = 0;
  __Pyx_RefNannyDeclarations
  __Pyx_RefNannySetupContext("__get__ (wrapper)", 0);
  __pyx_r = __pyx_pf_5model_10Bondarenko_5k_pcb___get__(((struct __pyx_obj_5model_Bondarenko *)__pyx_v_self));

  /* function exit code */
  __Pyx_RefNannyFinishContext();
  return __pyx_r;
}

static PyObject *__pyx_pf_5model_10Bondarenko_5k_pcb___get__(struct __pyx_obj_5model_Bondarenko *__pyx_v_self) {
  PyObject *__pyx_r = NULL;
  __Pyx_RefNannyDeclarations
  __Pyx_RefNannySetupContext("__get__", 0);
  __Pyx_XDECREF(__pyx_r);
  __pyx_t_1 = PyFloat_FromDouble(__pyx_v_self->k_pcb); if (unlikely(!__pyx_t_1)) {__pyx_filename = __pyx_f[0]; __pyx_lineno = 47; __pyx_clineno = __LINE__; goto __pyx_L1_error;}
  __Pyx_GOTREF(__pyx_t_1);
  __pyx_r = __pyx_t_1;
  __pyx_t_1 = 0;
  goto __pyx_L0;

  /* function exit code */
  __pyx_L1_error:;
  __Pyx_XDECREF(__pyx_t_1);
  __Pyx_AddTraceback("model.Bondarenko.k_pcb.__get__", __pyx_clineno, __pyx_lineno, __pyx_filename);
  __pyx_r = NULL;
  __pyx_L0:;
  __Pyx_XGIVEREF(__pyx_r);
  __Pyx_RefNannyFinishContext();
  return __pyx_r;
}

/* Python wrapper */
static int __pyx_pw_5model_10Bondarenko_5k_pcb_3__set__(PyObject *__pyx_v_self, PyObject *__pyx_v_value); /*proto*/
static int __pyx_pw_5model_10Bondarenko_5k_pcb_3__set__(PyObject *__pyx_v_self, PyObject *__pyx_v_value) {
  int __pyx_r;
  __Pyx_RefNannyDeclarations
  __Pyx_RefNannySetupContext("__set__ (wrapper)", 0);
  __pyx_r = __pyx_pf_5model_10Bondarenko_5k_pcb_2__set__(((struct __pyx_obj_5model_Bondarenko *)__pyx_v_self), ((PyObject *)__pyx_v_value));

  /* function exit code */
  __Pyx_RefNannyFinishContext();
  return __pyx_r;
}

static int __pyx_pf_5model_10Bondarenko_5k_pcb_2__set__(struct __pyx_obj_5model_Bondarenko *__pyx_v_self, PyObject *__pyx_v_value) {
  int __pyx_r;
  __Pyx_RefNannyDeclarations
  __Pyx_RefNannySetupContext("__set__", 0);
  __pyx_t_1 = __pyx_PyFloat_AsDouble(__pyx_v_value); if (unlikely((__pyx_t_1 == (double)-1) && PyErr_Occurred())) {__pyx_filename = __pyx_f[0]; __pyx_lineno = 47; __pyx_clineno = __LINE__; goto __pyx_L1_error;}
  __pyx_v_self->k_pcb = __pyx_t_1;

  /* function exit code */
  __pyx_r = 0;
  goto __pyx_L0;
  __pyx_L1_error:;
  __Pyx_AddTraceback("model.Bondarenko.k_pcb.__set__", __pyx_clineno, __pyx_lineno, __pyx_filename);
  __pyx_r = -1;
  __pyx_L0:;
  __Pyx_RefNannyFinishContext();
  return __pyx_r;
}
```

```
 48:     cdef public double I_CaLmax
```

```
/* Python wrapper */
static PyObject *__pyx_pw_5model_10Bondarenko_8I_CaLmax_1__get__(PyObject *__pyx_v_self); /*proto*/
static PyObject *__pyx_pw_5model_10Bondarenko_8I_CaLmax_1__get__(PyObject *__pyx_v_self) {
  PyObject *__pyx_r = 0;
  __Pyx_RefNannyDeclarations
  __Pyx_RefNannySetupContext("__get__ (wrapper)", 0);
  __pyx_r = __pyx_pf_5model_10Bondarenko_8I_CaLmax___get__(((struct __pyx_obj_5model_Bondarenko *)__pyx_v_self));

  /* function exit code */
  __Pyx_RefNannyFinishContext();
  return __pyx_r;
}

static PyObject *__pyx_pf_5model_10Bondarenko_8I_CaLmax___get__(struct __pyx_obj_5model_Bondarenko *__pyx_v_self) {
  PyObject *__pyx_r = NULL;
  __Pyx_RefNannyDeclarations
  __Pyx_RefNannySetupContext("__get__", 0);
  __Pyx_XDECREF(__pyx_r);
  __pyx_t_1 = PyFloat_FromDouble(__pyx_v_self->I_CaLmax); if (unlikely(!__pyx_t_1)) {__pyx_filename = __pyx_f[0]; __pyx_lineno = 48; __pyx_clineno = __LINE__; goto __pyx_L1_error;}
  __Pyx_GOTREF(__pyx_t_1);
  __pyx_r = __pyx_t_1;
  __pyx_t_1 = 0;
  goto __pyx_L0;

  /* function exit code */
  __pyx_L1_error:;
  __Pyx_XDECREF(__pyx_t_1);
  __Pyx_AddTraceback("model.Bondarenko.I_CaLmax.__get__", __pyx_clineno, __pyx_lineno, __pyx_filename);
  __pyx_r = NULL;
  __pyx_L0:;
  __Pyx_XGIVEREF(__pyx_r);
  __Pyx_RefNannyFinishContext();
  return __pyx_r;
}

/* Python wrapper */
static int __pyx_pw_5model_10Bondarenko_8I_CaLmax_3__set__(PyObject *__pyx_v_self, PyObject *__pyx_v_value); /*proto*/
static int __pyx_pw_5model_10Bondarenko_8I_CaLmax_3__set__(PyObject *__pyx_v_self, PyObject *__pyx_v_value) {
  int __pyx_r;
  __Pyx_RefNannyDeclarations
  __Pyx_RefNannySetupContext("__set__ (wrapper)", 0);
  __pyx_r = __pyx_pf_5model_10Bondarenko_8I_CaLmax_2__set__(((struct __pyx_obj_5model_Bondarenko *)__pyx_v_self), ((PyObject *)__pyx_v_value));

  /* function exit code */
  __Pyx_RefNannyFinishContext();
  return __pyx_r;
}

static int __pyx_pf_5model_10Bondarenko_8I_CaLmax_2__set__(struct __pyx_obj_5model_Bondarenko *__pyx_v_self, PyObject *__pyx_v_value) {
  int __pyx_r;
  __Pyx_RefNannyDeclarations
  __Pyx_RefNannySetupContext("__set__", 0);
  __pyx_t_1 = __pyx_PyFloat_AsDouble(__pyx_v_value); if (unlikely((__pyx_t_1 == (double)-1) && PyErr_Occurred())) {__pyx_filename = __pyx_f[0]; __pyx_lineno = 48; __pyx_clineno = __LINE__; goto __pyx_L1_error;}
  __pyx_v_self->I_CaLmax = __pyx_t_1;

  /* function exit code */
  __pyx_r = 0;
  goto __pyx_L0;
  __pyx_L1_error:;
  __Pyx_AddTraceback("model.Bondarenko.I_CaLmax.__set__", __pyx_clineno, __pyx_lineno, __pyx_filename);
  __pyx_r = -1;
  __pyx_L0:;
  __Pyx_RefNannyFinishContext();
  return __pyx_r;
}
```

```
 49:     cdef public double LTRPN_tot
```

```
/* Python wrapper */
static PyObject *__pyx_pw_5model_10Bondarenko_9LTRPN_tot_1__get__(PyObject *__pyx_v_self); /*proto*/
static PyObject *__pyx_pw_5model_10Bondarenko_9LTRPN_tot_1__get__(PyObject *__pyx_v_self) {
  PyObject *__pyx_r = 0;
  __Pyx_RefNannyDeclarations
  __Pyx_RefNannySetupContext("__get__ (wrapper)", 0);
  __pyx_r = __pyx_pf_5model_10Bondarenko_9LTRPN_tot___get__(((struct __pyx_obj_5model_Bondarenko *)__pyx_v_self));

  /* function exit code */
  __Pyx_RefNannyFinishContext();
  return __pyx_r;
}

static PyObject *__pyx_pf_5model_10Bondarenko_9LTRPN_tot___get__(struct __pyx_obj_5model_Bondarenko *__pyx_v_self) {
  PyObject *__pyx_r = NULL;
  __Pyx_RefNannyDeclarations
  __Pyx_RefNannySetupContext("__get__", 0);
  __Pyx_XDECREF(__pyx_r);
  __pyx_t_1 = PyFloat_FromDouble(__pyx_v_self->LTRPN_tot); if (unlikely(!__pyx_t_1)) {__pyx_filename = __pyx_f[0]; __pyx_lineno = 49; __pyx_clineno = __LINE__; goto __pyx_L1_error;}
  __Pyx_GOTREF(__pyx_t_1);
  __pyx_r = __pyx_t_1;
  __pyx_t_1 = 0;
  goto __pyx_L0;

  /* function exit code */
  __pyx_L1_error:;
  __Pyx_XDECREF(__pyx_t_1);
  __Pyx_AddTraceback("model.Bondarenko.LTRPN_tot.__get__", __pyx_clineno, __pyx_lineno, __pyx_filename);
  __pyx_r = NULL;
  __pyx_L0:;
  __Pyx_XGIVEREF(__pyx_r);
  __Pyx_RefNannyFinishContext();
  return __pyx_r;
}

/* Python wrapper */
static int __pyx_pw_5model_10Bondarenko_9LTRPN_tot_3__set__(PyObject *__pyx_v_self, PyObject *__pyx_v_value); /*proto*/
static int __pyx_pw_5model_10Bondarenko_9LTRPN_tot_3__set__(PyObject *__pyx_v_self, PyObject *__pyx_v_value) {
  int __pyx_r;
  __Pyx_RefNannyDeclarations
  __Pyx_RefNannySetupContext("__set__ (wrapper)", 0);
  __pyx_r = __pyx_pf_5model_10Bondarenko_9LTRPN_tot_2__set__(((struct __pyx_obj_5model_Bondarenko *)__pyx_v_self), ((PyObject *)__pyx_v_value));

  /* function exit code */
  __Pyx_RefNannyFinishContext();
  return __pyx_r;
}

static int __pyx_pf_5model_10Bondarenko_9LTRPN_tot_2__set__(struct __pyx_obj_5model_Bondarenko *__pyx_v_self, PyObject *__pyx_v_value) {
  int __pyx_r;
  __Pyx_RefNannyDeclarations
  __Pyx_RefNannySetupContext("__set__", 0);
  __pyx_t_1 = __pyx_PyFloat_AsDouble(__pyx_v_value); if (unlikely((__pyx_t_1 == (double)-1) && PyErr_Occurred())) {__pyx_filename = __pyx_f[0]; __pyx_lineno = 49; __pyx_clineno = __LINE__; goto __pyx_L1_error;}
  __pyx_v_self->LTRPN_tot = __pyx_t_1;

  /* function exit code */
  __pyx_r = 0;
  goto __pyx_L0;
  __pyx_L1_error:;
  __Pyx_AddTraceback("model.Bondarenko.LTRPN_tot.__set__", __pyx_clineno, __pyx_lineno, __pyx_filename);
  __pyx_r = -1;
  __pyx_L0:;
  __Pyx_RefNannyFinishContext();
  return __pyx_r;
}
```

```
 50:     cdef public double HTRPN_tot
```

```
/* Python wrapper */
static PyObject *__pyx_pw_5model_10Bondarenko_9HTRPN_tot_1__get__(PyObject *__pyx_v_self); /*proto*/
static PyObject *__pyx_pw_5model_10Bondarenko_9HTRPN_tot_1__get__(PyObject *__pyx_v_self) {
  PyObject *__pyx_r = 0;
  __Pyx_RefNannyDeclarations
  __Pyx_RefNannySetupContext("__get__ (wrapper)", 0);
  __pyx_r = __pyx_pf_5model_10Bondarenko_9HTRPN_tot___get__(((struct __pyx_obj_5model_Bondarenko *)__pyx_v_self));

  /* function exit code */
  __Pyx_RefNannyFinishContext();
  return __pyx_r;
}

static PyObject *__pyx_pf_5model_10Bondarenko_9HTRPN_tot___get__(struct __pyx_obj_5model_Bondarenko *__pyx_v_self) {
  PyObject *__pyx_r = NULL;
  __Pyx_RefNannyDeclarations
  __Pyx_RefNannySetupContext("__get__", 0);
  __Pyx_XDECREF(__pyx_r);
  __pyx_t_1 = PyFloat_FromDouble(__pyx_v_self->HTRPN_tot); if (unlikely(!__pyx_t_1)) {__pyx_filename = __pyx_f[0]; __pyx_lineno = 50; __pyx_clineno = __LINE__; goto __pyx_L1_error;}
  __Pyx_GOTREF(__pyx_t_1);
  __pyx_r = __pyx_t_1;
  __pyx_t_1 = 0;
  goto __pyx_L0;

  /* function exit code */
  __pyx_L1_error:;
  __Pyx_XDECREF(__pyx_t_1);
  __Pyx_AddTraceback("model.Bondarenko.HTRPN_tot.__get__", __pyx_clineno, __pyx_lineno, __pyx_filename);
  __pyx_r = NULL;
  __pyx_L0:;
  __Pyx_XGIVEREF(__pyx_r);
  __Pyx_RefNannyFinishContext();
  return __pyx_r;
}

/* Python wrapper */
static int __pyx_pw_5model_10Bondarenko_9HTRPN_tot_3__set__(PyObject *__pyx_v_self, PyObject *__pyx_v_value); /*proto*/
static int __pyx_pw_5model_10Bondarenko_9HTRPN_tot_3__set__(PyObject *__pyx_v_self, PyObject *__pyx_v_value) {
  int __pyx_r;
  __Pyx_RefNannyDeclarations
  __Pyx_RefNannySetupContext("__set__ (wrapper)", 0);
  __pyx_r = __pyx_pf_5model_10Bondarenko_9HTRPN_tot_2__set__(((struct __pyx_obj_5model_Bondarenko *)__pyx_v_self), ((PyObject *)__pyx_v_value));

  /* function exit code */
  __Pyx_RefNannyFinishContext();
  return __pyx_r;
}

static int __pyx_pf_5model_10Bondarenko_9HTRPN_tot_2__set__(struct __pyx_obj_5model_Bondarenko *__pyx_v_self, PyObject *__pyx_v_value) {
  int __pyx_r;
  __Pyx_RefNannyDeclarations
  __Pyx_RefNannySetupContext("__set__", 0);
  __pyx_t_1 = __pyx_PyFloat_AsDouble(__pyx_v_value); if (unlikely((__pyx_t_1 == (double)-1) && PyErr_Occurred())) {__pyx_filename = __pyx_f[0]; __pyx_lineno = 50; __pyx_clineno = __LINE__; goto __pyx_L1_error;}
  __pyx_v_self->HTRPN_tot = __pyx_t_1;

  /* function exit code */
  __pyx_r = 0;
  goto __pyx_L0;
  __pyx_L1_error:;
  __Pyx_AddTraceback("model.Bondarenko.HTRPN_tot.__set__", __pyx_clineno, __pyx_lineno, __pyx_filename);
  __pyx_r = -1;
  __pyx_L0:;
  __Pyx_RefNannyFinishContext();
  return __pyx_r;
}
```

```
 51:     cdef public double k_p_htrpn
```

```
/* Python wrapper */
static PyObject *__pyx_pw_5model_10Bondarenko_9k_p_htrpn_1__get__(PyObject *__pyx_v_self); /*proto*/
static PyObject *__pyx_pw_5model_10Bondarenko_9k_p_htrpn_1__get__(PyObject *__pyx_v_self) {
  PyObject *__pyx_r = 0;
  __Pyx_RefNannyDeclarations
  __Pyx_RefNannySetupContext("__get__ (wrapper)", 0);
  __pyx_r = __pyx_pf_5model_10Bondarenko_9k_p_htrpn___get__(((struct __pyx_obj_5model_Bondarenko *)__pyx_v_self));

  /* function exit code */
  __Pyx_RefNannyFinishContext();
  return __pyx_r;
}

static PyObject *__pyx_pf_5model_10Bondarenko_9k_p_htrpn___get__(struct __pyx_obj_5model_Bondarenko *__pyx_v_self) {
  PyObject *__pyx_r = NULL;
  __Pyx_RefNannyDeclarations
  __Pyx_RefNannySetupContext("__get__", 0);
  __Pyx_XDECREF(__pyx_r);
  __pyx_t_1 = PyFloat_FromDouble(__pyx_v_self->k_p_htrpn); if (unlikely(!__pyx_t_1)) {__pyx_filename = __pyx_f[0]; __pyx_lineno = 51; __pyx_clineno = __LINE__; goto __pyx_L1_error;}
  __Pyx_GOTREF(__pyx_t_1);
  __pyx_r = __pyx_t_1;
  __pyx_t_1 = 0;
  goto __pyx_L0;

  /* function exit code */
  __pyx_L1_error:;
  __Pyx_XDECREF(__pyx_t_1);
  __Pyx_AddTraceback("model.Bondarenko.k_p_htrpn.__get__", __pyx_clineno, __pyx_lineno, __pyx_filename);
  __pyx_r = NULL;
  __pyx_L0:;
  __Pyx_XGIVEREF(__pyx_r);
  __Pyx_RefNannyFinishContext();
  return __pyx_r;
}

/* Python wrapper */
static int __pyx_pw_5model_10Bondarenko_9k_p_htrpn_3__set__(PyObject *__pyx_v_self, PyObject *__pyx_v_value); /*proto*/
static int __pyx_pw_5model_10Bondarenko_9k_p_htrpn_3__set__(PyObject *__pyx_v_self, PyObject *__pyx_v_value) {
  int __pyx_r;
  __Pyx_RefNannyDeclarations
  __Pyx_RefNannySetupContext("__set__ (wrapper)", 0);
  __pyx_r = __pyx_pf_5model_10Bondarenko_9k_p_htrpn_2__set__(((struct __pyx_obj_5model_Bondarenko *)__pyx_v_self), ((PyObject *)__pyx_v_value));

  /* function exit code */
  __Pyx_RefNannyFinishContext();
  return __pyx_r;
}

static int __pyx_pf_5model_10Bondarenko_9k_p_htrpn_2__set__(struct __pyx_obj_5model_Bondarenko *__pyx_v_self, PyObject *__pyx_v_value) {
  int __pyx_r;
  __Pyx_RefNannyDeclarations
  __Pyx_RefNannySetupContext("__set__", 0);
  __pyx_t_1 = __pyx_PyFloat_AsDouble(__pyx_v_value); if (unlikely((__pyx_t_1 == (double)-1) && PyErr_Occurred())) {__pyx_filename = __pyx_f[0]; __pyx_lineno = 51; __pyx_clineno = __LINE__; goto __pyx_L1_error;}
  __pyx_v_self->k_p_htrpn = __pyx_t_1;

  /* function exit code */
  __pyx_r = 0;
  goto __pyx_L0;
  __pyx_L1_error:;
  __Pyx_AddTraceback("model.Bondarenko.k_p_htrpn.__set__", __pyx_clineno, __pyx_lineno, __pyx_filename);
  __pyx_r = -1;
  __pyx_L0:;
  __Pyx_RefNannyFinishContext();
  return __pyx_r;
}
```

```
 52:     cdef public double k_n_htrpn
```

```
/* Python wrapper */
static PyObject *__pyx_pw_5model_10Bondarenko_9k_n_htrpn_1__get__(PyObject *__pyx_v_self); /*proto*/
static PyObject *__pyx_pw_5model_10Bondarenko_9k_n_htrpn_1__get__(PyObject *__pyx_v_self) {
  PyObject *__pyx_r = 0;
  __Pyx_RefNannyDeclarations
  __Pyx_RefNannySetupContext("__get__ (wrapper)", 0);
  __pyx_r = __pyx_pf_5model_10Bondarenko_9k_n_htrpn___get__(((struct __pyx_obj_5model_Bondarenko *)__pyx_v_self));

  /* function exit code */
  __Pyx_RefNannyFinishContext();
  return __pyx_r;
}

static PyObject *__pyx_pf_5model_10Bondarenko_9k_n_htrpn___get__(struct __pyx_obj_5model_Bondarenko *__pyx_v_self) {
  PyObject *__pyx_r = NULL;
  __Pyx_RefNannyDeclarations
  __Pyx_RefNannySetupContext("__get__", 0);
  __Pyx_XDECREF(__pyx_r);
  __pyx_t_1 = PyFloat_FromDouble(__pyx_v_self->k_n_htrpn); if (unlikely(!__pyx_t_1)) {__pyx_filename = __pyx_f[0]; __pyx_lineno = 52; __pyx_clineno = __LINE__; goto __pyx_L1_error;}
  __Pyx_GOTREF(__pyx_t_1);
  __pyx_r = __pyx_t_1;
  __pyx_t_1 = 0;
  goto __pyx_L0;

  /* function exit code */
  __pyx_L1_error:;
  __Pyx_XDECREF(__pyx_t_1);
  __Pyx_AddTraceback("model.Bondarenko.k_n_htrpn.__get__", __pyx_clineno, __pyx_lineno, __pyx_filename);
  __pyx_r = NULL;
  __pyx_L0:;
  __Pyx_XGIVEREF(__pyx_r);
  __Pyx_RefNannyFinishContext();
  return __pyx_r;
}

/* Python wrapper */
static int __pyx_pw_5model_10Bondarenko_9k_n_htrpn_3__set__(PyObject *__pyx_v_self, PyObject *__pyx_v_value); /*proto*/
static int __pyx_pw_5model_10Bondarenko_9k_n_htrpn_3__set__(PyObject *__pyx_v_self, PyObject *__pyx_v_value) {
  int __pyx_r;
  __Pyx_RefNannyDeclarations
  __Pyx_RefNannySetupContext("__set__ (wrapper)", 0);
  __pyx_r = __pyx_pf_5model_10Bondarenko_9k_n_htrpn_2__set__(((struct __pyx_obj_5model_Bondarenko *)__pyx_v_self), ((PyObject *)__pyx_v_value));

  /* function exit code */
  __Pyx_RefNannyFinishContext();
  return __pyx_r;
}

static int __pyx_pf_5model_10Bondarenko_9k_n_htrpn_2__set__(struct __pyx_obj_5model_Bondarenko *__pyx_v_self, PyObject *__pyx_v_value) {
  int __pyx_r;
  __Pyx_RefNannyDeclarations
  __Pyx_RefNannySetupContext("__set__", 0);
  __pyx_t_1 = __pyx_PyFloat_AsDouble(__pyx_v_value); if (unlikely((__pyx_t_1 == (double)-1) && PyErr_Occurred())) {__pyx_filename = __pyx_f[0]; __pyx_lineno = 52; __pyx_clineno = __LINE__; goto __pyx_L1_error;}
  __pyx_v_self->k_n_htrpn = __pyx_t_1;

  /* function exit code */
  __pyx_r = 0;
  goto __pyx_L0;
  __pyx_L1_error:;
  __Pyx_AddTraceback("model.Bondarenko.k_n_htrpn.__set__", __pyx_clineno, __pyx_lineno, __pyx_filename);
  __pyx_r = -1;
  __pyx_L0:;
  __Pyx_RefNannyFinishContext();
  return __pyx_r;
}
```

```
 53:     cdef public double k_p_ltrpn
```

```
/* Python wrapper */
static PyObject *__pyx_pw_5model_10Bondarenko_9k_p_ltrpn_1__get__(PyObject *__pyx_v_self); /*proto*/
static PyObject *__pyx_pw_5model_10Bondarenko_9k_p_ltrpn_1__get__(PyObject *__pyx_v_self) {
  PyObject *__pyx_r = 0;
  __Pyx_RefNannyDeclarations
  __Pyx_RefNannySetupContext("__get__ (wrapper)", 0);
  __pyx_r = __pyx_pf_5model_10Bondarenko_9k_p_ltrpn___get__(((struct __pyx_obj_5model_Bondarenko *)__pyx_v_self));

  /* function exit code */
  __Pyx_RefNannyFinishContext();
  return __pyx_r;
}

static PyObject *__pyx_pf_5model_10Bondarenko_9k_p_ltrpn___get__(struct __pyx_obj_5model_Bondarenko *__pyx_v_self) {
  PyObject *__pyx_r = NULL;
  __Pyx_RefNannyDeclarations
  __Pyx_RefNannySetupContext("__get__", 0);
  __Pyx_XDECREF(__pyx_r);
  __pyx_t_1 = PyFloat_FromDouble(__pyx_v_self->k_p_ltrpn); if (unlikely(!__pyx_t_1)) {__pyx_filename = __pyx_f[0]; __pyx_lineno = 53; __pyx_clineno = __LINE__; goto __pyx_L1_error;}
  __Pyx_GOTREF(__pyx_t_1);
  __pyx_r = __pyx_t_1;
  __pyx_t_1 = 0;
  goto __pyx_L0;

  /* function exit code */
  __pyx_L1_error:;
  __Pyx_XDECREF(__pyx_t_1);
  __Pyx_AddTraceback("model.Bondarenko.k_p_ltrpn.__get__", __pyx_clineno, __pyx_lineno, __pyx_filename);
  __pyx_r = NULL;
  __pyx_L0:;
  __Pyx_XGIVEREF(__pyx_r);
  __Pyx_RefNannyFinishContext();
  return __pyx_r;
}

/* Python wrapper */
static int __pyx_pw_5model_10Bondarenko_9k_p_ltrpn_3__set__(PyObject *__pyx_v_self, PyObject *__pyx_v_value); /*proto*/
static int __pyx_pw_5model_10Bondarenko_9k_p_ltrpn_3__set__(PyObject *__pyx_v_self, PyObject *__pyx_v_value) {
  int __pyx_r;
  __Pyx_RefNannyDeclarations
  __Pyx_RefNannySetupContext("__set__ (wrapper)", 0);
  __pyx_r = __pyx_pf_5model_10Bondarenko_9k_p_ltrpn_2__set__(((struct __pyx_obj_5model_Bondarenko *)__pyx_v_self), ((PyObject *)__pyx_v_value));

  /* function exit code */
  __Pyx_RefNannyFinishContext();
  return __pyx_r;
}

static int __pyx_pf_5model_10Bondarenko_9k_p_ltrpn_2__set__(struct __pyx_obj_5model_Bondarenko *__pyx_v_self, PyObject *__pyx_v_value) {
  int __pyx_r;
  __Pyx_RefNannyDeclarations
  __Pyx_RefNannySetupContext("__set__", 0);
  __pyx_t_1 = __pyx_PyFloat_AsDouble(__pyx_v_value); if (unlikely((__pyx_t_1 == (double)-1) && PyErr_Occurred())) {__pyx_filename = __pyx_f[0]; __pyx_lineno = 53; __pyx_clineno = __LINE__; goto __pyx_L1_error;}
  __pyx_v_self->k_p_ltrpn = __pyx_t_1;

  /* function exit code */
  __pyx_r = 0;
  goto __pyx_L0;
  __pyx_L1_error:;
  __Pyx_AddTraceback("model.Bondarenko.k_p_ltrpn.__set__", __pyx_clineno, __pyx_lineno, __pyx_filename);
  __pyx_r = -1;
  __pyx_L0:;
  __Pyx_RefNannyFinishContext();
  return __pyx_r;
}
```

```
 54:     cdef public double k_n_ltrpn
```

```
/* Python wrapper */
static PyObject *__pyx_pw_5model_10Bondarenko_9k_n_ltrpn_1__get__(PyObject *__pyx_v_self); /*proto*/
static PyObject *__pyx_pw_5model_10Bondarenko_9k_n_ltrpn_1__get__(PyObject *__pyx_v_self) {
  PyObject *__pyx_r = 0;
  __Pyx_RefNannyDeclarations
  __Pyx_RefNannySetupContext("__get__ (wrapper)", 0);
  __pyx_r = __pyx_pf_5model_10Bondarenko_9k_n_ltrpn___get__(((struct __pyx_obj_5model_Bondarenko *)__pyx_v_self));

  /* function exit code */
  __Pyx_RefNannyFinishContext();
  return __pyx_r;
}

static PyObject *__pyx_pf_5model_10Bondarenko_9k_n_ltrpn___get__(struct __pyx_obj_5model_Bondarenko *__pyx_v_self) {
  PyObject *__pyx_r = NULL;
  __Pyx_RefNannyDeclarations
  __Pyx_RefNannySetupContext("__get__", 0);
  __Pyx_XDECREF(__pyx_r);
  __pyx_t_1 = PyFloat_FromDouble(__pyx_v_self->k_n_ltrpn); if (unlikely(!__pyx_t_1)) {__pyx_filename = __pyx_f[0]; __pyx_lineno = 54; __pyx_clineno = __LINE__; goto __pyx_L1_error;}
  __Pyx_GOTREF(__pyx_t_1);
  __pyx_r = __pyx_t_1;
  __pyx_t_1 = 0;
  goto __pyx_L0;

  /* function exit code */
  __pyx_L1_error:;
  __Pyx_XDECREF(__pyx_t_1);
  __Pyx_AddTraceback("model.Bondarenko.k_n_ltrpn.__get__", __pyx_clineno, __pyx_lineno, __pyx_filename);
  __pyx_r = NULL;
  __pyx_L0:;
  __Pyx_XGIVEREF(__pyx_r);
  __Pyx_RefNannyFinishContext();
  return __pyx_r;
}

/* Python wrapper */
static int __pyx_pw_5model_10Bondarenko_9k_n_ltrpn_3__set__(PyObject *__pyx_v_self, PyObject *__pyx_v_value); /*proto*/
static int __pyx_pw_5model_10Bondarenko_9k_n_ltrpn_3__set__(PyObject *__pyx_v_self, PyObject *__pyx_v_value) {
  int __pyx_r;
  __Pyx_RefNannyDeclarations
  __Pyx_RefNannySetupContext("__set__ (wrapper)", 0);
  __pyx_r = __pyx_pf_5model_10Bondarenko_9k_n_ltrpn_2__set__(((struct __pyx_obj_5model_Bondarenko *)__pyx_v_self), ((PyObject *)__pyx_v_value));

  /* function exit code */
  __Pyx_RefNannyFinishContext();
  return __pyx_r;
}

static int __pyx_pf_5model_10Bondarenko_9k_n_ltrpn_2__set__(struct __pyx_obj_5model_Bondarenko *__pyx_v_self, PyObject *__pyx_v_value) {
  int __pyx_r;
  __Pyx_RefNannyDeclarations
  __Pyx_RefNannySetupContext("__set__", 0);
  __pyx_t_1 = __pyx_PyFloat_AsDouble(__pyx_v_value); if (unlikely((__pyx_t_1 == (double)-1) && PyErr_Occurred())) {__pyx_filename = __pyx_f[0]; __pyx_lineno = 54; __pyx_clineno = __LINE__; goto __pyx_L1_error;}
  __pyx_v_self->k_n_ltrpn = __pyx_t_1;

  /* function exit code */
  __pyx_r = 0;
  goto __pyx_L0;
  __pyx_L1_error:;
  __Pyx_AddTraceback("model.Bondarenko.k_n_ltrpn.__set__", __pyx_clineno, __pyx_lineno, __pyx_filename);
  __pyx_r = -1;
  __pyx_L0:;
  __Pyx_RefNannyFinishContext();
  return __pyx_r;
}
```

```
 55:     cdef public double CMDN_tot
```

```
/* Python wrapper */
static PyObject *__pyx_pw_5model_10Bondarenko_8CMDN_tot_1__get__(PyObject *__pyx_v_self); /*proto*/
static PyObject *__pyx_pw_5model_10Bondarenko_8CMDN_tot_1__get__(PyObject *__pyx_v_self) {
  PyObject *__pyx_r = 0;
  __Pyx_RefNannyDeclarations
  __Pyx_RefNannySetupContext("__get__ (wrapper)", 0);
  __pyx_r = __pyx_pf_5model_10Bondarenko_8CMDN_tot___get__(((struct __pyx_obj_5model_Bondarenko *)__pyx_v_self));

  /* function exit code */
  __Pyx_RefNannyFinishContext();
  return __pyx_r;
}

static PyObject *__pyx_pf_5model_10Bondarenko_8CMDN_tot___get__(struct __pyx_obj_5model_Bondarenko *__pyx_v_self) {
  PyObject *__pyx_r = NULL;
  __Pyx_RefNannyDeclarations
  __Pyx_RefNannySetupContext("__get__", 0);
  __Pyx_XDECREF(__pyx_r);
  __pyx_t_1 = PyFloat_FromDouble(__pyx_v_self->CMDN_tot); if (unlikely(!__pyx_t_1)) {__pyx_filename = __pyx_f[0]; __pyx_lineno = 55; __pyx_clineno = __LINE__; goto __pyx_L1_error;}
  __Pyx_GOTREF(__pyx_t_1);
  __pyx_r = __pyx_t_1;
  __pyx_t_1 = 0;
  goto __pyx_L0;

  /* function exit code */
  __pyx_L1_error:;
  __Pyx_XDECREF(__pyx_t_1);
  __Pyx_AddTraceback("model.Bondarenko.CMDN_tot.__get__", __pyx_clineno, __pyx_lineno, __pyx_filename);
  __pyx_r = NULL;
  __pyx_L0:;
  __Pyx_XGIVEREF(__pyx_r);
  __Pyx_RefNannyFinishContext();
  return __pyx_r;
}

/* Python wrapper */
static int __pyx_pw_5model_10Bondarenko_8CMDN_tot_3__set__(PyObject *__pyx_v_self, PyObject *__pyx_v_value); /*proto*/
static int __pyx_pw_5model_10Bondarenko_8CMDN_tot_3__set__(PyObject *__pyx_v_self, PyObject *__pyx_v_value) {
  int __pyx_r;
  __Pyx_RefNannyDeclarations
  __Pyx_RefNannySetupContext("__set__ (wrapper)", 0);
  __pyx_r = __pyx_pf_5model_10Bondarenko_8CMDN_tot_2__set__(((struct __pyx_obj_5model_Bondarenko *)__pyx_v_self), ((PyObject *)__pyx_v_value));

  /* function exit code */
  __Pyx_RefNannyFinishContext();
  return __pyx_r;
}

static int __pyx_pf_5model_10Bondarenko_8CMDN_tot_2__set__(struct __pyx_obj_5model_Bondarenko *__pyx_v_self, PyObject *__pyx_v_value) {
  int __pyx_r;
  __Pyx_RefNannyDeclarations
  __Pyx_RefNannySetupContext("__set__", 0);
  __pyx_t_1 = __pyx_PyFloat_AsDouble(__pyx_v_value); if (unlikely((__pyx_t_1 == (double)-1) && PyErr_Occurred())) {__pyx_filename = __pyx_f[0]; __pyx_lineno = 55; __pyx_clineno = __LINE__; goto __pyx_L1_error;}
  __pyx_v_self->CMDN_tot = __pyx_t_1;

  /* function exit code */
  __pyx_r = 0;
  goto __pyx_L0;
  __pyx_L1_error:;
  __Pyx_AddTraceback("model.Bondarenko.CMDN_tot.__set__", __pyx_clineno, __pyx_lineno, __pyx_filename);
  __pyx_r = -1;
  __pyx_L0:;
  __Pyx_RefNannyFinishContext();
  return __pyx_r;
}
```

```
 56:     cdef public double CSQN_tot
```

```
/* Python wrapper */
static PyObject *__pyx_pw_5model_10Bondarenko_8CSQN_tot_1__get__(PyObject *__pyx_v_self); /*proto*/
static PyObject *__pyx_pw_5model_10Bondarenko_8CSQN_tot_1__get__(PyObject *__pyx_v_self) {
  PyObject *__pyx_r = 0;
  __Pyx_RefNannyDeclarations
  __Pyx_RefNannySetupContext("__get__ (wrapper)", 0);
  __pyx_r = __pyx_pf_5model_10Bondarenko_8CSQN_tot___get__(((struct __pyx_obj_5model_Bondarenko *)__pyx_v_self));

  /* function exit code */
  __Pyx_RefNannyFinishContext();
  return __pyx_r;
}

static PyObject *__pyx_pf_5model_10Bondarenko_8CSQN_tot___get__(struct __pyx_obj_5model_Bondarenko *__pyx_v_self) {
  PyObject *__pyx_r = NULL;
  __Pyx_RefNannyDeclarations
  __Pyx_RefNannySetupContext("__get__", 0);
  __Pyx_XDECREF(__pyx_r);
  __pyx_t_1 = PyFloat_FromDouble(__pyx_v_self->CSQN_tot); if (unlikely(!__pyx_t_1)) {__pyx_filename = __pyx_f[0]; __pyx_lineno = 56; __pyx_clineno = __LINE__; goto __pyx_L1_error;}
  __Pyx_GOTREF(__pyx_t_1);
  __pyx_r = __pyx_t_1;
  __pyx_t_1 = 0;
  goto __pyx_L0;

  /* function exit code */
  __pyx_L1_error:;
  __Pyx_XDECREF(__pyx_t_1);
  __Pyx_AddTraceback("model.Bondarenko.CSQN_tot.__get__", __pyx_clineno, __pyx_lineno, __pyx_filename);
  __pyx_r = NULL;
  __pyx_L0:;
  __Pyx_XGIVEREF(__pyx_r);
  __Pyx_RefNannyFinishContext();
  return __pyx_r;
}

/* Python wrapper */
static int __pyx_pw_5model_10Bondarenko_8CSQN_tot_3__set__(PyObject *__pyx_v_self, PyObject *__pyx_v_value); /*proto*/
static int __pyx_pw_5model_10Bondarenko_8CSQN_tot_3__set__(PyObject *__pyx_v_self, PyObject *__pyx_v_value) {
  int __pyx_r;
  __Pyx_RefNannyDeclarations
  __Pyx_RefNannySetupContext("__set__ (wrapper)", 0);
  __pyx_r = __pyx_pf_5model_10Bondarenko_8CSQN_tot_2__set__(((struct __pyx_obj_5model_Bondarenko *)__pyx_v_self), ((PyObject *)__pyx_v_value));

  /* function exit code */
  __Pyx_RefNannyFinishContext();
  return __pyx_r;
}

static int __pyx_pf_5model_10Bondarenko_8CSQN_tot_2__set__(struct __pyx_obj_5model_Bondarenko *__pyx_v_self, PyObject *__pyx_v_value) {
  int __pyx_r;
  __Pyx_RefNannyDeclarations
  __Pyx_RefNannySetupContext("__set__", 0);
  __pyx_t_1 = __pyx_PyFloat_AsDouble(__pyx_v_value); if (unlikely((__pyx_t_1 == (double)-1) && PyErr_Occurred())) {__pyx_filename = __pyx_f[0]; __pyx_lineno = 56; __pyx_clineno = __LINE__; goto __pyx_L1_error;}
  __pyx_v_self->CSQN_tot = __pyx_t_1;

  /* function exit code */
  __pyx_r = 0;
  goto __pyx_L0;
  __pyx_L1_error:;
  __Pyx_AddTraceback("model.Bondarenko.CSQN_tot.__set__", __pyx_clineno, __pyx_lineno, __pyx_filename);
  __pyx_r = -1;
  __pyx_L0:;
  __Pyx_RefNannyFinishContext();
  return __pyx_r;
}
```

```
 57:     cdef public double k_CMDN_m
```

```
/* Python wrapper */
static PyObject *__pyx_pw_5model_10Bondarenko_8k_CMDN_m_1__get__(PyObject *__pyx_v_self); /*proto*/
static PyObject *__pyx_pw_5model_10Bondarenko_8k_CMDN_m_1__get__(PyObject *__pyx_v_self) {
  PyObject *__pyx_r = 0;
  __Pyx_RefNannyDeclarations
  __Pyx_RefNannySetupContext("__get__ (wrapper)", 0);
  __pyx_r = __pyx_pf_5model_10Bondarenko_8k_CMDN_m___get__(((struct __pyx_obj_5model_Bondarenko *)__pyx_v_self));

  /* function exit code */
  __Pyx_RefNannyFinishContext();
  return __pyx_r;
}

static PyObject *__pyx_pf_5model_10Bondarenko_8k_CMDN_m___get__(struct __pyx_obj_5model_Bondarenko *__pyx_v_self) {
  PyObject *__pyx_r = NULL;
  __Pyx_RefNannyDeclarations
  __Pyx_RefNannySetupContext("__get__", 0);
  __Pyx_XDECREF(__pyx_r);
  __pyx_t_1 = PyFloat_FromDouble(__pyx_v_self->k_CMDN_m); if (unlikely(!__pyx_t_1)) {__pyx_filename = __pyx_f[0]; __pyx_lineno = 57; __pyx_clineno = __LINE__; goto __pyx_L1_error;}
  __Pyx_GOTREF(__pyx_t_1);
  __pyx_r = __pyx_t_1;
  __pyx_t_1 = 0;
  goto __pyx_L0;

  /* function exit code */
  __pyx_L1_error:;
  __Pyx_XDECREF(__pyx_t_1);
  __Pyx_AddTraceback("model.Bondarenko.k_CMDN_m.__get__", __pyx_clineno, __pyx_lineno, __pyx_filename);
  __pyx_r = NULL;
  __pyx_L0:;
  __Pyx_XGIVEREF(__pyx_r);
  __Pyx_RefNannyFinishContext();
  return __pyx_r;
}

/* Python wrapper */
static int __pyx_pw_5model_10Bondarenko_8k_CMDN_m_3__set__(PyObject *__pyx_v_self, PyObject *__pyx_v_value); /*proto*/
static int __pyx_pw_5model_10Bondarenko_8k_CMDN_m_3__set__(PyObject *__pyx_v_self, PyObject *__pyx_v_value) {
  int __pyx_r;
  __Pyx_RefNannyDeclarations
  __Pyx_RefNannySetupContext("__set__ (wrapper)", 0);
  __pyx_r = __pyx_pf_5model_10Bondarenko_8k_CMDN_m_2__set__(((struct __pyx_obj_5model_Bondarenko *)__pyx_v_self), ((PyObject *)__pyx_v_value));

  /* function exit code */
  __Pyx_RefNannyFinishContext();
  return __pyx_r;
}

static int __pyx_pf_5model_10Bondarenko_8k_CMDN_m_2__set__(struct __pyx_obj_5model_Bondarenko *__pyx_v_self, PyObject *__pyx_v_value) {
  int __pyx_r;
  __Pyx_RefNannyDeclarations
  __Pyx_RefNannySetupContext("__set__", 0);
  __pyx_t_1 = __pyx_PyFloat_AsDouble(__pyx_v_value); if (unlikely((__pyx_t_1 == (double)-1) && PyErr_Occurred())) {__pyx_filename = __pyx_f[0]; __pyx_lineno = 57; __pyx_clineno = __LINE__; goto __pyx_L1_error;}
  __pyx_v_self->k_CMDN_m = __pyx_t_1;

  /* function exit code */
  __pyx_r = 0;
  goto __pyx_L0;
  __pyx_L1_error:;
  __Pyx_AddTraceback("model.Bondarenko.k_CMDN_m.__set__", __pyx_clineno, __pyx_lineno, __pyx_filename);
  __pyx_r = -1;
  __pyx_L0:;
  __Pyx_RefNannyFinishContext();
  return __pyx_r;
}
```

```
 58:     cdef public double k_CSQN_m
```

```
/* Python wrapper */
static PyObject *__pyx_pw_5model_10Bondarenko_8k_CSQN_m_1__get__(PyObject *__pyx_v_self); /*proto*/
static PyObject *__pyx_pw_5model_10Bondarenko_8k_CSQN_m_1__get__(PyObject *__pyx_v_self) {
  PyObject *__pyx_r = 0;
  __Pyx_RefNannyDeclarations
  __Pyx_RefNannySetupContext("__get__ (wrapper)", 0);
  __pyx_r = __pyx_pf_5model_10Bondarenko_8k_CSQN_m___get__(((struct __pyx_obj_5model_Bondarenko *)__pyx_v_self));

  /* function exit code */
  __Pyx_RefNannyFinishContext();
  return __pyx_r;
}

static PyObject *__pyx_pf_5model_10Bondarenko_8k_CSQN_m___get__(struct __pyx_obj_5model_Bondarenko *__pyx_v_self) {
  PyObject *__pyx_r = NULL;
  __Pyx_RefNannyDeclarations
  __Pyx_RefNannySetupContext("__get__", 0);
  __Pyx_XDECREF(__pyx_r);
  __pyx_t_1 = PyFloat_FromDouble(__pyx_v_self->k_CSQN_m); if (unlikely(!__pyx_t_1)) {__pyx_filename = __pyx_f[0]; __pyx_lineno = 58; __pyx_clineno = __LINE__; goto __pyx_L1_error;}
  __Pyx_GOTREF(__pyx_t_1);
  __pyx_r = __pyx_t_1;
  __pyx_t_1 = 0;
  goto __pyx_L0;

  /* function exit code */
  __pyx_L1_error:;
  __Pyx_XDECREF(__pyx_t_1);
  __Pyx_AddTraceback("model.Bondarenko.k_CSQN_m.__get__", __pyx_clineno, __pyx_lineno, __pyx_filename);
  __pyx_r = NULL;
  __pyx_L0:;
  __Pyx_XGIVEREF(__pyx_r);
  __Pyx_RefNannyFinishContext();
  return __pyx_r;
}

/* Python wrapper */
static int __pyx_pw_5model_10Bondarenko_8k_CSQN_m_3__set__(PyObject *__pyx_v_self, PyObject *__pyx_v_value); /*proto*/
static int __pyx_pw_5model_10Bondarenko_8k_CSQN_m_3__set__(PyObject *__pyx_v_self, PyObject *__pyx_v_value) {
  int __pyx_r;
  __Pyx_RefNannyDeclarations
  __Pyx_RefNannySetupContext("__set__ (wrapper)", 0);
  __pyx_r = __pyx_pf_5model_10Bondarenko_8k_CSQN_m_2__set__(((struct __pyx_obj_5model_Bondarenko *)__pyx_v_self), ((PyObject *)__pyx_v_value));

  /* function exit code */
  __Pyx_RefNannyFinishContext();
  return __pyx_r;
}

static int __pyx_pf_5model_10Bondarenko_8k_CSQN_m_2__set__(struct __pyx_obj_5model_Bondarenko *__pyx_v_self, PyObject *__pyx_v_value) {
  int __pyx_r;
  __Pyx_RefNannyDeclarations
  __Pyx_RefNannySetupContext("__set__", 0);
  __pyx_t_1 = __pyx_PyFloat_AsDouble(__pyx_v_value); if (unlikely((__pyx_t_1 == (double)-1) && PyErr_Occurred())) {__pyx_filename = __pyx_f[0]; __pyx_lineno = 58; __pyx_clineno = __LINE__; goto __pyx_L1_error;}
  __pyx_v_self->k_CSQN_m = __pyx_t_1;

  /* function exit code */
  __pyx_r = 0;
  goto __pyx_L0;
  __pyx_L1_error:;
  __Pyx_AddTraceback("model.Bondarenko.k_CSQN_m.__set__", __pyx_clineno, __pyx_lineno, __pyx_filename);
  __pyx_r = -1;
  __pyx_L0:;
  __Pyx_RefNannyFinishContext();
  return __pyx_r;
}
```

```
 59:     cdef public double C_m
```

```
/* Python wrapper */
static PyObject *__pyx_pw_5model_10Bondarenko_3C_m_1__get__(PyObject *__pyx_v_self); /*proto*/
static PyObject *__pyx_pw_5model_10Bondarenko_3C_m_1__get__(PyObject *__pyx_v_self) {
  PyObject *__pyx_r = 0;
  __Pyx_RefNannyDeclarations
  __Pyx_RefNannySetupContext("__get__ (wrapper)", 0);
  __pyx_r = __pyx_pf_5model_10Bondarenko_3C_m___get__(((struct __pyx_obj_5model_Bondarenko *)__pyx_v_self));

  /* function exit code */
  __Pyx_RefNannyFinishContext();
  return __pyx_r;
}

static PyObject *__pyx_pf_5model_10Bondarenko_3C_m___get__(struct __pyx_obj_5model_Bondarenko *__pyx_v_self) {
  PyObject *__pyx_r = NULL;
  __Pyx_RefNannyDeclarations
  __Pyx_RefNannySetupContext("__get__", 0);
  __Pyx_XDECREF(__pyx_r);
  __pyx_t_1 = PyFloat_FromDouble(__pyx_v_self->C_m); if (unlikely(!__pyx_t_1)) {__pyx_filename = __pyx_f[0]; __pyx_lineno = 59; __pyx_clineno = __LINE__; goto __pyx_L1_error;}
  __Pyx_GOTREF(__pyx_t_1);
  __pyx_r = __pyx_t_1;
  __pyx_t_1 = 0;
  goto __pyx_L0;

  /* function exit code */
  __pyx_L1_error:;
  __Pyx_XDECREF(__pyx_t_1);
  __Pyx_AddTraceback("model.Bondarenko.C_m.__get__", __pyx_clineno, __pyx_lineno, __pyx_filename);
  __pyx_r = NULL;
  __pyx_L0:;
  __Pyx_XGIVEREF(__pyx_r);
  __Pyx_RefNannyFinishContext();
  return __pyx_r;
}

/* Python wrapper */
static int __pyx_pw_5model_10Bondarenko_3C_m_3__set__(PyObject *__pyx_v_self, PyObject *__pyx_v_value); /*proto*/
static int __pyx_pw_5model_10Bondarenko_3C_m_3__set__(PyObject *__pyx_v_self, PyObject *__pyx_v_value) {
  int __pyx_r;
  __Pyx_RefNannyDeclarations
  __Pyx_RefNannySetupContext("__set__ (wrapper)", 0);
  __pyx_r = __pyx_pf_5model_10Bondarenko_3C_m_2__set__(((struct __pyx_obj_5model_Bondarenko *)__pyx_v_self), ((PyObject *)__pyx_v_value));

  /* function exit code */
  __Pyx_RefNannyFinishContext();
  return __pyx_r;
}

static int __pyx_pf_5model_10Bondarenko_3C_m_2__set__(struct __pyx_obj_5model_Bondarenko *__pyx_v_self, PyObject *__pyx_v_value) {
  int __pyx_r;
  __Pyx_RefNannyDeclarations
  __Pyx_RefNannySetupContext("__set__", 0);
  __pyx_t_1 = __pyx_PyFloat_AsDouble(__pyx_v_value); if (unlikely((__pyx_t_1 == (double)-1) && PyErr_Occurred())) {__pyx_filename = __pyx_f[0]; __pyx_lineno = 59; __pyx_clineno = __LINE__; goto __pyx_L1_error;}
  __pyx_v_self->C_m = __pyx_t_1;

  /* function exit code */
  __pyx_r = 0;
  goto __pyx_L0;
  __pyx_L1_error:;
  __Pyx_AddTraceback("model.Bondarenko.C_m.__set__", __pyx_clineno, __pyx_lineno, __pyx_filename);
  __pyx_r = -1;
  __pyx_L0:;
  __Pyx_RefNannyFinishContext();
  return __pyx_r;
}
```

```
 60:     cdef public double F
```

```
/* Python wrapper */
static PyObject *__pyx_pw_5model_10Bondarenko_1F_1__get__(PyObject *__pyx_v_self); /*proto*/
static PyObject *__pyx_pw_5model_10Bondarenko_1F_1__get__(PyObject *__pyx_v_self) {
  PyObject *__pyx_r = 0;
  __Pyx_RefNannyDeclarations
  __Pyx_RefNannySetupContext("__get__ (wrapper)", 0);
  __pyx_r = __pyx_pf_5model_10Bondarenko_1F___get__(((struct __pyx_obj_5model_Bondarenko *)__pyx_v_self));

  /* function exit code */
  __Pyx_RefNannyFinishContext();
  return __pyx_r;
}

static PyObject *__pyx_pf_5model_10Bondarenko_1F___get__(struct __pyx_obj_5model_Bondarenko *__pyx_v_self) {
  PyObject *__pyx_r = NULL;
  __Pyx_RefNannyDeclarations
  __Pyx_RefNannySetupContext("__get__", 0);
  __Pyx_XDECREF(__pyx_r);
  __pyx_t_1 = PyFloat_FromDouble(__pyx_v_self->F); if (unlikely(!__pyx_t_1)) {__pyx_filename = __pyx_f[0]; __pyx_lineno = 60; __pyx_clineno = __LINE__; goto __pyx_L1_error;}
  __Pyx_GOTREF(__pyx_t_1);
  __pyx_r = __pyx_t_1;
  __pyx_t_1 = 0;
  goto __pyx_L0;

  /* function exit code */
  __pyx_L1_error:;
  __Pyx_XDECREF(__pyx_t_1);
  __Pyx_AddTraceback("model.Bondarenko.F.__get__", __pyx_clineno, __pyx_lineno, __pyx_filename);
  __pyx_r = NULL;
  __pyx_L0:;
  __Pyx_XGIVEREF(__pyx_r);
  __Pyx_RefNannyFinishContext();
  return __pyx_r;
}

/* Python wrapper */
static int __pyx_pw_5model_10Bondarenko_1F_3__set__(PyObject *__pyx_v_self, PyObject *__pyx_v_value); /*proto*/
static int __pyx_pw_5model_10Bondarenko_1F_3__set__(PyObject *__pyx_v_self, PyObject *__pyx_v_value) {
  int __pyx_r;
  __Pyx_RefNannyDeclarations
  __Pyx_RefNannySetupContext("__set__ (wrapper)", 0);
  __pyx_r = __pyx_pf_5model_10Bondarenko_1F_2__set__(((struct __pyx_obj_5model_Bondarenko *)__pyx_v_self), ((PyObject *)__pyx_v_value));

  /* function exit code */
  __Pyx_RefNannyFinishContext();
  return __pyx_r;
}

static int __pyx_pf_5model_10Bondarenko_1F_2__set__(struct __pyx_obj_5model_Bondarenko *__pyx_v_self, PyObject *__pyx_v_value) {
  int __pyx_r;
  __Pyx_RefNannyDeclarations
  __Pyx_RefNannySetupContext("__set__", 0);
  __pyx_t_1 = __pyx_PyFloat_AsDouble(__pyx_v_value); if (unlikely((__pyx_t_1 == (double)-1) && PyErr_Occurred())) {__pyx_filename = __pyx_f[0]; __pyx_lineno = 60; __pyx_clineno = __LINE__; goto __pyx_L1_error;}
  __pyx_v_self->F = __pyx_t_1;

  /* function exit code */
  __pyx_r = 0;
  goto __pyx_L0;
  __pyx_L1_error:;
  __Pyx_AddTraceback("model.Bondarenko.F.__set__", __pyx_clineno, __pyx_lineno, __pyx_filename);
  __pyx_r = -1;
  __pyx_L0:;
  __Pyx_RefNannyFinishContext();
  return __pyx_r;
}
```

```
 61:     cdef public double T
```

```
/* Python wrapper */
static PyObject *__pyx_pw_5model_10Bondarenko_1T_1__get__(PyObject *__pyx_v_self); /*proto*/
static PyObject *__pyx_pw_5model_10Bondarenko_1T_1__get__(PyObject *__pyx_v_self) {
  PyObject *__pyx_r = 0;
  __Pyx_RefNannyDeclarations
  __Pyx_RefNannySetupContext("__get__ (wrapper)", 0);
  __pyx_r = __pyx_pf_5model_10Bondarenko_1T___get__(((struct __pyx_obj_5model_Bondarenko *)__pyx_v_self));

  /* function exit code */
  __Pyx_RefNannyFinishContext();
  return __pyx_r;
}

static PyObject *__pyx_pf_5model_10Bondarenko_1T___get__(struct __pyx_obj_5model_Bondarenko *__pyx_v_self) {
  PyObject *__pyx_r = NULL;
  __Pyx_RefNannyDeclarations
  __Pyx_RefNannySetupContext("__get__", 0);
  __Pyx_XDECREF(__pyx_r);
  __pyx_t_1 = PyFloat_FromDouble(__pyx_v_self->T); if (unlikely(!__pyx_t_1)) {__pyx_filename = __pyx_f[0]; __pyx_lineno = 61; __pyx_clineno = __LINE__; goto __pyx_L1_error;}
  __Pyx_GOTREF(__pyx_t_1);
  __pyx_r = __pyx_t_1;
  __pyx_t_1 = 0;
  goto __pyx_L0;

  /* function exit code */
  __pyx_L1_error:;
  __Pyx_XDECREF(__pyx_t_1);
  __Pyx_AddTraceback("model.Bondarenko.T.__get__", __pyx_clineno, __pyx_lineno, __pyx_filename);
  __pyx_r = NULL;
  __pyx_L0:;
  __Pyx_XGIVEREF(__pyx_r);
  __Pyx_RefNannyFinishContext();
  return __pyx_r;
}

/* Python wrapper */
static int __pyx_pw_5model_10Bondarenko_1T_3__set__(PyObject *__pyx_v_self, PyObject *__pyx_v_value); /*proto*/
static int __pyx_pw_5model_10Bondarenko_1T_3__set__(PyObject *__pyx_v_self, PyObject *__pyx_v_value) {
  int __pyx_r;
  __Pyx_RefNannyDeclarations
  __Pyx_RefNannySetupContext("__set__ (wrapper)", 0);
  __pyx_r = __pyx_pf_5model_10Bondarenko_1T_2__set__(((struct __pyx_obj_5model_Bondarenko *)__pyx_v_self), ((PyObject *)__pyx_v_value));

  /* function exit code */
  __Pyx_RefNannyFinishContext();
  return __pyx_r;
}

static int __pyx_pf_5model_10Bondarenko_1T_2__set__(struct __pyx_obj_5model_Bondarenko *__pyx_v_self, PyObject *__pyx_v_value) {
  int __pyx_r;
  __Pyx_RefNannyDeclarations
  __Pyx_RefNannySetupContext("__set__", 0);
  __pyx_t_1 = __pyx_PyFloat_AsDouble(__pyx_v_value); if (unlikely((__pyx_t_1 == (double)-1) && PyErr_Occurred())) {__pyx_filename = __pyx_f[0]; __pyx_lineno = 61; __pyx_clineno = __LINE__; goto __pyx_L1_error;}
  __pyx_v_self->T = __pyx_t_1;

  /* function exit code */
  __pyx_r = 0;
  goto __pyx_L0;
  __pyx_L1_error:;
  __Pyx_AddTraceback("model.Bondarenko.T.__set__", __pyx_clineno, __pyx_lineno, __pyx_filename);
  __pyx_r = -1;
  __pyx_L0:;
  __Pyx_RefNannyFinishContext();
  return __pyx_r;
}
```

```
 62:     cdef public double R
```

```
/* Python wrapper */
static PyObject *__pyx_pw_5model_10Bondarenko_1R_1__get__(PyObject *__pyx_v_self); /*proto*/
static PyObject *__pyx_pw_5model_10Bondarenko_1R_1__get__(PyObject *__pyx_v_self) {
  PyObject *__pyx_r = 0;
  __Pyx_RefNannyDeclarations
  __Pyx_RefNannySetupContext("__get__ (wrapper)", 0);
  __pyx_r = __pyx_pf_5model_10Bondarenko_1R___get__(((struct __pyx_obj_5model_Bondarenko *)__pyx_v_self));

  /* function exit code */
  __Pyx_RefNannyFinishContext();
  return __pyx_r;
}

static PyObject *__pyx_pf_5model_10Bondarenko_1R___get__(struct __pyx_obj_5model_Bondarenko *__pyx_v_self) {
  PyObject *__pyx_r = NULL;
  __Pyx_RefNannyDeclarations
  __Pyx_RefNannySetupContext("__get__", 0);
  __Pyx_XDECREF(__pyx_r);
  __pyx_t_1 = PyFloat_FromDouble(__pyx_v_self->R); if (unlikely(!__pyx_t_1)) {__pyx_filename = __pyx_f[0]; __pyx_lineno = 62; __pyx_clineno = __LINE__; goto __pyx_L1_error;}
  __Pyx_GOTREF(__pyx_t_1);
  __pyx_r = __pyx_t_1;
  __pyx_t_1 = 0;
  goto __pyx_L0;

  /* function exit code */
  __pyx_L1_error:;
  __Pyx_XDECREF(__pyx_t_1);
  __Pyx_AddTraceback("model.Bondarenko.R.__get__", __pyx_clineno, __pyx_lineno, __pyx_filename);
  __pyx_r = NULL;
  __pyx_L0:;
  __Pyx_XGIVEREF(__pyx_r);
  __Pyx_RefNannyFinishContext();
  return __pyx_r;
}

/* Python wrapper */
static int __pyx_pw_5model_10Bondarenko_1R_3__set__(PyObject *__pyx_v_self, PyObject *__pyx_v_value); /*proto*/
static int __pyx_pw_5model_10Bondarenko_1R_3__set__(PyObject *__pyx_v_self, PyObject *__pyx_v_value) {
  int __pyx_r;
  __Pyx_RefNannyDeclarations
  __Pyx_RefNannySetupContext("__set__ (wrapper)", 0);
  __pyx_r = __pyx_pf_5model_10Bondarenko_1R_2__set__(((struct __pyx_obj_5model_Bondarenko *)__pyx_v_self), ((PyObject *)__pyx_v_value));

  /* function exit code */
  __Pyx_RefNannyFinishContext();
  return __pyx_r;
}

static int __pyx_pf_5model_10Bondarenko_1R_2__set__(struct __pyx_obj_5model_Bondarenko *__pyx_v_self, PyObject *__pyx_v_value) {
  int __pyx_r;
  __Pyx_RefNannyDeclarations
  __Pyx_RefNannySetupContext("__set__", 0);
  __pyx_t_1 = __pyx_PyFloat_AsDouble(__pyx_v_value); if (unlikely((__pyx_t_1 == (double)-1) && PyErr_Occurred())) {__pyx_filename = __pyx_f[0]; __pyx_lineno = 62; __pyx_clineno = __LINE__; goto __pyx_L1_error;}
  __pyx_v_self->R = __pyx_t_1;

  /* function exit code */
  __pyx_r = 0;
  goto __pyx_L0;
  __pyx_L1_error:;
  __Pyx_AddTraceback("model.Bondarenko.R.__set__", __pyx_clineno, __pyx_lineno, __pyx_filename);
  __pyx_r = -1;
  __pyx_L0:;
  __Pyx_RefNannyFinishContext();
  return __pyx_r;
}
```

```
 63:     cdef public double k_NaCa
```

```
/* Python wrapper */
static PyObject *__pyx_pw_5model_10Bondarenko_6k_NaCa_1__get__(PyObject *__pyx_v_self); /*proto*/
static PyObject *__pyx_pw_5model_10Bondarenko_6k_NaCa_1__get__(PyObject *__pyx_v_self) {
  PyObject *__pyx_r = 0;
  __Pyx_RefNannyDeclarations
  __Pyx_RefNannySetupContext("__get__ (wrapper)", 0);
  __pyx_r = __pyx_pf_5model_10Bondarenko_6k_NaCa___get__(((struct __pyx_obj_5model_Bondarenko *)__pyx_v_self));

  /* function exit code */
  __Pyx_RefNannyFinishContext();
  return __pyx_r;
}

static PyObject *__pyx_pf_5model_10Bondarenko_6k_NaCa___get__(struct __pyx_obj_5model_Bondarenko *__pyx_v_self) {
  PyObject *__pyx_r = NULL;
  __Pyx_RefNannyDeclarations
  __Pyx_RefNannySetupContext("__get__", 0);
  __Pyx_XDECREF(__pyx_r);
  __pyx_t_1 = PyFloat_FromDouble(__pyx_v_self->k_NaCa); if (unlikely(!__pyx_t_1)) {__pyx_filename = __pyx_f[0]; __pyx_lineno = 63; __pyx_clineno = __LINE__; goto __pyx_L1_error;}
  __Pyx_GOTREF(__pyx_t_1);
  __pyx_r = __pyx_t_1;
  __pyx_t_1 = 0;
  goto __pyx_L0;

  /* function exit code */
  __pyx_L1_error:;
  __Pyx_XDECREF(__pyx_t_1);
  __Pyx_AddTraceback("model.Bondarenko.k_NaCa.__get__", __pyx_clineno, __pyx_lineno, __pyx_filename);
  __pyx_r = NULL;
  __pyx_L0:;
  __Pyx_XGIVEREF(__pyx_r);
  __Pyx_RefNannyFinishContext();
  return __pyx_r;
}

/* Python wrapper */
static int __pyx_pw_5model_10Bondarenko_6k_NaCa_3__set__(PyObject *__pyx_v_self, PyObject *__pyx_v_value); /*proto*/
static int __pyx_pw_5model_10Bondarenko_6k_NaCa_3__set__(PyObject *__pyx_v_self, PyObject *__pyx_v_value) {
  int __pyx_r;
  __Pyx_RefNannyDeclarations
  __Pyx_RefNannySetupContext("__set__ (wrapper)", 0);
  __pyx_r = __pyx_pf_5model_10Bondarenko_6k_NaCa_2__set__(((struct __pyx_obj_5model_Bondarenko *)__pyx_v_self), ((PyObject *)__pyx_v_value));

  /* function exit code */
  __Pyx_RefNannyFinishContext();
  return __pyx_r;
}

static int __pyx_pf_5model_10Bondarenko_6k_NaCa_2__set__(struct __pyx_obj_5model_Bondarenko *__pyx_v_self, PyObject *__pyx_v_value) {
  int __pyx_r;
  __Pyx_RefNannyDeclarations
  __Pyx_RefNannySetupContext("__set__", 0);
  __pyx_t_1 = __pyx_PyFloat_AsDouble(__pyx_v_value); if (unlikely((__pyx_t_1 == (double)-1) && PyErr_Occurred())) {__pyx_filename = __pyx_f[0]; __pyx_lineno = 63; __pyx_clineno = __LINE__; goto __pyx_L1_error;}
  __pyx_v_self->k_NaCa = __pyx_t_1;

  /* function exit code */
  __pyx_r = 0;
  goto __pyx_L0;
  __pyx_L1_error:;
  __Pyx_AddTraceback("model.Bondarenko.k_NaCa.__set__", __pyx_clineno, __pyx_lineno, __pyx_filename);
  __pyx_r = -1;
  __pyx_L0:;
  __Pyx_RefNannyFinishContext();
  return __pyx_r;
}
```

```
 64:     cdef public double k_mNa
```

```
/* Python wrapper */
static PyObject *__pyx_pw_5model_10Bondarenko_5k_mNa_1__get__(PyObject *__pyx_v_self); /*proto*/
static PyObject *__pyx_pw_5model_10Bondarenko_5k_mNa_1__get__(PyObject *__pyx_v_self) {
  PyObject *__pyx_r = 0;
  __Pyx_RefNannyDeclarations
  __Pyx_RefNannySetupContext("__get__ (wrapper)", 0);
  __pyx_r = __pyx_pf_5model_10Bondarenko_5k_mNa___get__(((struct __pyx_obj_5model_Bondarenko *)__pyx_v_self));

  /* function exit code */
  __Pyx_RefNannyFinishContext();
  return __pyx_r;
}

static PyObject *__pyx_pf_5model_10Bondarenko_5k_mNa___get__(struct __pyx_obj_5model_Bondarenko *__pyx_v_self) {
  PyObject *__pyx_r = NULL;
  __Pyx_RefNannyDeclarations
  __Pyx_RefNannySetupContext("__get__", 0);
  __Pyx_XDECREF(__pyx_r);
  __pyx_t_1 = PyFloat_FromDouble(__pyx_v_self->k_mNa); if (unlikely(!__pyx_t_1)) {__pyx_filename = __pyx_f[0]; __pyx_lineno = 64; __pyx_clineno = __LINE__; goto __pyx_L1_error;}
  __Pyx_GOTREF(__pyx_t_1);
  __pyx_r = __pyx_t_1;
  __pyx_t_1 = 0;
  goto __pyx_L0;

  /* function exit code */
  __pyx_L1_error:;
  __Pyx_XDECREF(__pyx_t_1);
  __Pyx_AddTraceback("model.Bondarenko.k_mNa.__get__", __pyx_clineno, __pyx_lineno, __pyx_filename);
  __pyx_r = NULL;
  __pyx_L0:;
  __Pyx_XGIVEREF(__pyx_r);
  __Pyx_RefNannyFinishContext();
  return __pyx_r;
}

/* Python wrapper */
static int __pyx_pw_5model_10Bondarenko_5k_mNa_3__set__(PyObject *__pyx_v_self, PyObject *__pyx_v_value); /*proto*/
static int __pyx_pw_5model_10Bondarenko_5k_mNa_3__set__(PyObject *__pyx_v_self, PyObject *__pyx_v_value) {
  int __pyx_r;
  __Pyx_RefNannyDeclarations
  __Pyx_RefNannySetupContext("__set__ (wrapper)", 0);
  __pyx_r = __pyx_pf_5model_10Bondarenko_5k_mNa_2__set__(((struct __pyx_obj_5model_Bondarenko *)__pyx_v_self), ((PyObject *)__pyx_v_value));

  /* function exit code */
  __Pyx_RefNannyFinishContext();
  return __pyx_r;
}

static int __pyx_pf_5model_10Bondarenko_5k_mNa_2__set__(struct __pyx_obj_5model_Bondarenko *__pyx_v_self, PyObject *__pyx_v_value) {
  int __pyx_r;
  __Pyx_RefNannyDeclarations
  __Pyx_RefNannySetupContext("__set__", 0);
  __pyx_t_1 = __pyx_PyFloat_AsDouble(__pyx_v_value); if (unlikely((__pyx_t_1 == (double)-1) && PyErr_Occurred())) {__pyx_filename = __pyx_f[0]; __pyx_lineno = 64; __pyx_clineno = __LINE__; goto __pyx_L1_error;}
  __pyx_v_self->k_mNa = __pyx_t_1;

  /* function exit code */
  __pyx_r = 0;
  goto __pyx_L0;
  __pyx_L1_error:;
  __Pyx_AddTraceback("model.Bondarenko.k_mNa.__set__", __pyx_clineno, __pyx_lineno, __pyx_filename);
  __pyx_r = -1;
  __pyx_L0:;
  __Pyx_RefNannyFinishContext();
  return __pyx_r;
}
```

```
 65:     cdef public double k_mCa
```

```
/* Python wrapper */
static PyObject *__pyx_pw_5model_10Bondarenko_5k_mCa_1__get__(PyObject *__pyx_v_self); /*proto*/
static PyObject *__pyx_pw_5model_10Bondarenko_5k_mCa_1__get__(PyObject *__pyx_v_self) {
  PyObject *__pyx_r = 0;
  __Pyx_RefNannyDeclarations
  __Pyx_RefNannySetupContext("__get__ (wrapper)", 0);
  __pyx_r = __pyx_pf_5model_10Bondarenko_5k_mCa___get__(((struct __pyx_obj_5model_Bondarenko *)__pyx_v_self));

  /* function exit code */
  __Pyx_RefNannyFinishContext();
  return __pyx_r;
}

static PyObject *__pyx_pf_5model_10Bondarenko_5k_mCa___get__(struct __pyx_obj_5model_Bondarenko *__pyx_v_self) {
  PyObject *__pyx_r = NULL;
  __Pyx_RefNannyDeclarations
  __Pyx_RefNannySetupContext("__get__", 0);
  __Pyx_XDECREF(__pyx_r);
  __pyx_t_1 = PyFloat_FromDouble(__pyx_v_self->k_mCa); if (unlikely(!__pyx_t_1)) {__pyx_filename = __pyx_f[0]; __pyx_lineno = 65; __pyx_clineno = __LINE__; goto __pyx_L1_error;}
  __Pyx_GOTREF(__pyx_t_1);
  __pyx_r = __pyx_t_1;
  __pyx_t_1 = 0;
  goto __pyx_L0;

  /* function exit code */
  __pyx_L1_error:;
  __Pyx_XDECREF(__pyx_t_1);
  __Pyx_AddTraceback("model.Bondarenko.k_mCa.__get__", __pyx_clineno, __pyx_lineno, __pyx_filename);
  __pyx_r = NULL;
  __pyx_L0:;
  __Pyx_XGIVEREF(__pyx_r);
  __Pyx_RefNannyFinishContext();
  return __pyx_r;
}

/* Python wrapper */
static int __pyx_pw_5model_10Bondarenko_5k_mCa_3__set__(PyObject *__pyx_v_self, PyObject *__pyx_v_value); /*proto*/
static int __pyx_pw_5model_10Bondarenko_5k_mCa_3__set__(PyObject *__pyx_v_self, PyObject *__pyx_v_value) {
  int __pyx_r;
  __Pyx_RefNannyDeclarations
  __Pyx_RefNannySetupContext("__set__ (wrapper)", 0);
  __pyx_r = __pyx_pf_5model_10Bondarenko_5k_mCa_2__set__(((struct __pyx_obj_5model_Bondarenko *)__pyx_v_self), ((PyObject *)__pyx_v_value));

  /* function exit code */
  __Pyx_RefNannyFinishContext();
  return __pyx_r;
}

static int __pyx_pf_5model_10Bondarenko_5k_mCa_2__set__(struct __pyx_obj_5model_Bondarenko *__pyx_v_self, PyObject *__pyx_v_value) {
  int __pyx_r;
  __Pyx_RefNannyDeclarations
  __Pyx_RefNannySetupContext("__set__", 0);
  __pyx_t_1 = __pyx_PyFloat_AsDouble(__pyx_v_value); if (unlikely((__pyx_t_1 == (double)-1) && PyErr_Occurred())) {__pyx_filename = __pyx_f[0]; __pyx_lineno = 65; __pyx_clineno = __LINE__; goto __pyx_L1_error;}
  __pyx_v_self->k_mCa = __pyx_t_1;

  /* function exit code */
  __pyx_r = 0;
  goto __pyx_L0;
  __pyx_L1_error:;
  __Pyx_AddTraceback("model.Bondarenko.k_mCa.__set__", __pyx_clineno, __pyx_lineno, __pyx_filename);
  __pyx_r = -1;
  __pyx_L0:;
  __Pyx_RefNannyFinishContext();
  return __pyx_r;
}
```

```
 66:     cdef public double k_sat
```

```
/* Python wrapper */
static PyObject *__pyx_pw_5model_10Bondarenko_5k_sat_1__get__(PyObject *__pyx_v_self); /*proto*/
static PyObject *__pyx_pw_5model_10Bondarenko_5k_sat_1__get__(PyObject *__pyx_v_self) {
  PyObject *__pyx_r = 0;
  __Pyx_RefNannyDeclarations
  __Pyx_RefNannySetupContext("__get__ (wrapper)", 0);
  __pyx_r = __pyx_pf_5model_10Bondarenko_5k_sat___get__(((struct __pyx_obj_5model_Bondarenko *)__pyx_v_self));

  /* function exit code */
  __Pyx_RefNannyFinishContext();
  return __pyx_r;
}

static PyObject *__pyx_pf_5model_10Bondarenko_5k_sat___get__(struct __pyx_obj_5model_Bondarenko *__pyx_v_self) {
  PyObject *__pyx_r = NULL;
  __Pyx_RefNannyDeclarations
  __Pyx_RefNannySetupContext("__get__", 0);
  __Pyx_XDECREF(__pyx_r);
  __pyx_t_1 = PyFloat_FromDouble(__pyx_v_self->k_sat); if (unlikely(!__pyx_t_1)) {__pyx_filename = __pyx_f[0]; __pyx_lineno = 66; __pyx_clineno = __LINE__; goto __pyx_L1_error;}
  __Pyx_GOTREF(__pyx_t_1);
  __pyx_r = __pyx_t_1;
  __pyx_t_1 = 0;
  goto __pyx_L0;

  /* function exit code */
  __pyx_L1_error:;
  __Pyx_XDECREF(__pyx_t_1);
  __Pyx_AddTraceback("model.Bondarenko.k_sat.__get__", __pyx_clineno, __pyx_lineno, __pyx_filename);
  __pyx_r = NULL;
  __pyx_L0:;
  __Pyx_XGIVEREF(__pyx_r);
  __Pyx_RefNannyFinishContext();
  return __pyx_r;
}

/* Python wrapper */
static int __pyx_pw_5model_10Bondarenko_5k_sat_3__set__(PyObject *__pyx_v_self, PyObject *__pyx_v_value); /*proto*/
static int __pyx_pw_5model_10Bondarenko_5k_sat_3__set__(PyObject *__pyx_v_self, PyObject *__pyx_v_value) {
  int __pyx_r;
  __Pyx_RefNannyDeclarations
  __Pyx_RefNannySetupContext("__set__ (wrapper)", 0);
  __pyx_r = __pyx_pf_5model_10Bondarenko_5k_sat_2__set__(((struct __pyx_obj_5model_Bondarenko *)__pyx_v_self), ((PyObject *)__pyx_v_value));

  /* function exit code */
  __Pyx_RefNannyFinishContext();
  return __pyx_r;
}

static int __pyx_pf_5model_10Bondarenko_5k_sat_2__set__(struct __pyx_obj_5model_Bondarenko *__pyx_v_self, PyObject *__pyx_v_value) {
  int __pyx_r;
  __Pyx_RefNannyDeclarations
  __Pyx_RefNannySetupContext("__set__", 0);
  __pyx_t_1 = __pyx_PyFloat_AsDouble(__pyx_v_value); if (unlikely((__pyx_t_1 == (double)-1) && PyErr_Occurred())) {__pyx_filename = __pyx_f[0]; __pyx_lineno = 66; __pyx_clineno = __LINE__; goto __pyx_L1_error;}
  __pyx_v_self->k_sat = __pyx_t_1;

  /* function exit code */
  __pyx_r = 0;
  goto __pyx_L0;
  __pyx_L1_error:;
  __Pyx_AddTraceback("model.Bondarenko.k_sat.__set__", __pyx_clineno, __pyx_lineno, __pyx_filename);
  __pyx_r = -1;
  __pyx_L0:;
  __Pyx_RefNannyFinishContext();
  return __pyx_r;
}
```

```
 67:     cdef public double miu
```

```
/* Python wrapper */
static PyObject *__pyx_pw_5model_10Bondarenko_3miu_1__get__(PyObject *__pyx_v_self); /*proto*/
static PyObject *__pyx_pw_5model_10Bondarenko_3miu_1__get__(PyObject *__pyx_v_self) {
  PyObject *__pyx_r = 0;
  __Pyx_RefNannyDeclarations
  __Pyx_RefNannySetupContext("__get__ (wrapper)", 0);
  __pyx_r = __pyx_pf_5model_10Bondarenko_3miu___get__(((struct __pyx_obj_5model_Bondarenko *)__pyx_v_self));

  /* function exit code */
  __Pyx_RefNannyFinishContext();
  return __pyx_r;
}

static PyObject *__pyx_pf_5model_10Bondarenko_3miu___get__(struct __pyx_obj_5model_Bondarenko *__pyx_v_self) {
  PyObject *__pyx_r = NULL;
  __Pyx_RefNannyDeclarations
  __Pyx_RefNannySetupContext("__get__", 0);
  __Pyx_XDECREF(__pyx_r);
  __pyx_t_1 = PyFloat_FromDouble(__pyx_v_self->miu); if (unlikely(!__pyx_t_1)) {__pyx_filename = __pyx_f[0]; __pyx_lineno = 67; __pyx_clineno = __LINE__; goto __pyx_L1_error;}
  __Pyx_GOTREF(__pyx_t_1);
  __pyx_r = __pyx_t_1;
  __pyx_t_1 = 0;
  goto __pyx_L0;

  /* function exit code */
  __pyx_L1_error:;
  __Pyx_XDECREF(__pyx_t_1);
  __Pyx_AddTraceback("model.Bondarenko.miu.__get__", __pyx_clineno, __pyx_lineno, __pyx_filename);
  __pyx_r = NULL;
  __pyx_L0:;
  __Pyx_XGIVEREF(__pyx_r);
  __Pyx_RefNannyFinishContext();
  return __pyx_r;
}

/* Python wrapper */
static int __pyx_pw_5model_10Bondarenko_3miu_3__set__(PyObject *__pyx_v_self, PyObject *__pyx_v_value); /*proto*/
static int __pyx_pw_5model_10Bondarenko_3miu_3__set__(PyObject *__pyx_v_self, PyObject *__pyx_v_value) {
  int __pyx_r;
  __Pyx_RefNannyDeclarations
  __Pyx_RefNannySetupContext("__set__ (wrapper)", 0);
  __pyx_r = __pyx_pf_5model_10Bondarenko_3miu_2__set__(((struct __pyx_obj_5model_Bondarenko *)__pyx_v_self), ((PyObject *)__pyx_v_value));

  /* function exit code */
  __Pyx_RefNannyFinishContext();
  return __pyx_r;
}

static int __pyx_pf_5model_10Bondarenko_3miu_2__set__(struct __pyx_obj_5model_Bondarenko *__pyx_v_self, PyObject *__pyx_v_value) {
  int __pyx_r;
  __Pyx_RefNannyDeclarations
  __Pyx_RefNannySetupContext("__set__", 0);
  __pyx_t_1 = __pyx_PyFloat_AsDouble(__pyx_v_value); if (unlikely((__pyx_t_1 == (double)-1) && PyErr_Occurred())) {__pyx_filename = __pyx_f[0]; __pyx_lineno = 67; __pyx_clineno = __LINE__; goto __pyx_L1_error;}
  __pyx_v_self->miu = __pyx_t_1;

  /* function exit code */
  __pyx_r = 0;
  goto __pyx_L0;
  __pyx_L1_error:;
  __Pyx_AddTraceback("model.Bondarenko.miu.__set__", __pyx_clineno, __pyx_lineno, __pyx_filename);
  __pyx_r = -1;
  __pyx_L0:;
  __Pyx_RefNannyFinishContext();
  return __pyx_r;
}
```

```
 68:     cdef public double I_maxNaK
```

```
/* Python wrapper */
static PyObject *__pyx_pw_5model_10Bondarenko_8I_maxNaK_1__get__(PyObject *__pyx_v_self); /*proto*/
static PyObject *__pyx_pw_5model_10Bondarenko_8I_maxNaK_1__get__(PyObject *__pyx_v_self) {
  PyObject *__pyx_r = 0;
  __Pyx_RefNannyDeclarations
  __Pyx_RefNannySetupContext("__get__ (wrapper)", 0);
  __pyx_r = __pyx_pf_5model_10Bondarenko_8I_maxNaK___get__(((struct __pyx_obj_5model_Bondarenko *)__pyx_v_self));

  /* function exit code */
  __Pyx_RefNannyFinishContext();
  return __pyx_r;
}

static PyObject *__pyx_pf_5model_10Bondarenko_8I_maxNaK___get__(struct __pyx_obj_5model_Bondarenko *__pyx_v_self) {
  PyObject *__pyx_r = NULL;
  __Pyx_RefNannyDeclarations
  __Pyx_RefNannySetupContext("__get__", 0);
  __Pyx_XDECREF(__pyx_r);
  __pyx_t_1 = PyFloat_FromDouble(__pyx_v_self->I_maxNaK); if (unlikely(!__pyx_t_1)) {__pyx_filename = __pyx_f[0]; __pyx_lineno = 68; __pyx_clineno = __LINE__; goto __pyx_L1_error;}
  __Pyx_GOTREF(__pyx_t_1);
  __pyx_r = __pyx_t_1;
  __pyx_t_1 = 0;
  goto __pyx_L0;

  /* function exit code */
  __pyx_L1_error:;
  __Pyx_XDECREF(__pyx_t_1);
  __Pyx_AddTraceback("model.Bondarenko.I_maxNaK.__get__", __pyx_clineno, __pyx_lineno, __pyx_filename);
  __pyx_r = NULL;
  __pyx_L0:;
  __Pyx_XGIVEREF(__pyx_r);
  __Pyx_RefNannyFinishContext();
  return __pyx_r;
}

/* Python wrapper */
static int __pyx_pw_5model_10Bondarenko_8I_maxNaK_3__set__(PyObject *__pyx_v_self, PyObject *__pyx_v_value); /*proto*/
static int __pyx_pw_5model_10Bondarenko_8I_maxNaK_3__set__(PyObject *__pyx_v_self, PyObject *__pyx_v_value) {
  int __pyx_r;
  __Pyx_RefNannyDeclarations
  __Pyx_RefNannySetupContext("__set__ (wrapper)", 0);
  __pyx_r = __pyx_pf_5model_10Bondarenko_8I_maxNaK_2__set__(((struct __pyx_obj_5model_Bondarenko *)__pyx_v_self), ((PyObject *)__pyx_v_value));

  /* function exit code */
  __Pyx_RefNannyFinishContext();
  return __pyx_r;
}

static int __pyx_pf_5model_10Bondarenko_8I_maxNaK_2__set__(struct __pyx_obj_5model_Bondarenko *__pyx_v_self, PyObject *__pyx_v_value) {
  int __pyx_r;
  __Pyx_RefNannyDeclarations
  __Pyx_RefNannySetupContext("__set__", 0);
  __pyx_t_1 = __pyx_PyFloat_AsDouble(__pyx_v_value); if (unlikely((__pyx_t_1 == (double)-1) && PyErr_Occurred())) {__pyx_filename = __pyx_f[0]; __pyx_lineno = 68; __pyx_clineno = __LINE__; goto __pyx_L1_error;}
  __pyx_v_self->I_maxNaK = __pyx_t_1;

  /* function exit code */
  __pyx_r = 0;
  goto __pyx_L0;
  __pyx_L1_error:;
  __Pyx_AddTraceback("model.Bondarenko.I_maxNaK.__set__", __pyx_clineno, __pyx_lineno, __pyx_filename);
  __pyx_r = -1;
  __pyx_L0:;
  __Pyx_RefNannyFinishContext();
  return __pyx_r;
}
```

```
 69:     cdef public double K_mNai
```

```
/* Python wrapper */
static PyObject *__pyx_pw_5model_10Bondarenko_6K_mNai_1__get__(PyObject *__pyx_v_self); /*proto*/
static PyObject *__pyx_pw_5model_10Bondarenko_6K_mNai_1__get__(PyObject *__pyx_v_self) {
  PyObject *__pyx_r = 0;
  __Pyx_RefNannyDeclarations
  __Pyx_RefNannySetupContext("__get__ (wrapper)", 0);
  __pyx_r = __pyx_pf_5model_10Bondarenko_6K_mNai___get__(((struct __pyx_obj_5model_Bondarenko *)__pyx_v_self));

  /* function exit code */
  __Pyx_RefNannyFinishContext();
  return __pyx_r;
}

static PyObject *__pyx_pf_5model_10Bondarenko_6K_mNai___get__(struct __pyx_obj_5model_Bondarenko *__pyx_v_self) {
  PyObject *__pyx_r = NULL;
  __Pyx_RefNannyDeclarations
  __Pyx_RefNannySetupContext("__get__", 0);
  __Pyx_XDECREF(__pyx_r);
  __pyx_t_1 = PyFloat_FromDouble(__pyx_v_self->K_mNai); if (unlikely(!__pyx_t_1)) {__pyx_filename = __pyx_f[0]; __pyx_lineno = 69; __pyx_clineno = __LINE__; goto __pyx_L1_error;}
  __Pyx_GOTREF(__pyx_t_1);
  __pyx_r = __pyx_t_1;
  __pyx_t_1 = 0;
  goto __pyx_L0;

  /* function exit code */
  __pyx_L1_error:;
  __Pyx_XDECREF(__pyx_t_1);
  __Pyx_AddTraceback("model.Bondarenko.K_mNai.__get__", __pyx_clineno, __pyx_lineno, __pyx_filename);
  __pyx_r = NULL;
  __pyx_L0:;
  __Pyx_XGIVEREF(__pyx_r);
  __Pyx_RefNannyFinishContext();
  return __pyx_r;
}

/* Python wrapper */
static int __pyx_pw_5model_10Bondarenko_6K_mNai_3__set__(PyObject *__pyx_v_self, PyObject *__pyx_v_value); /*proto*/
static int __pyx_pw_5model_10Bondarenko_6K_mNai_3__set__(PyObject *__pyx_v_self, PyObject *__pyx_v_value) {
  int __pyx_r;
  __Pyx_RefNannyDeclarations
  __Pyx_RefNannySetupContext("__set__ (wrapper)", 0);
  __pyx_r = __pyx_pf_5model_10Bondarenko_6K_mNai_2__set__(((struct __pyx_obj_5model_Bondarenko *)__pyx_v_self), ((PyObject *)__pyx_v_value));

  /* function exit code */
  __Pyx_RefNannyFinishContext();
  return __pyx_r;
}

static int __pyx_pf_5model_10Bondarenko_6K_mNai_2__set__(struct __pyx_obj_5model_Bondarenko *__pyx_v_self, PyObject *__pyx_v_value) {
  int __pyx_r;
  __Pyx_RefNannyDeclarations
  __Pyx_RefNannySetupContext("__set__", 0);
  __pyx_t_1 = __pyx_PyFloat_AsDouble(__pyx_v_value); if (unlikely((__pyx_t_1 == (double)-1) && PyErr_Occurred())) {__pyx_filename = __pyx_f[0]; __pyx_lineno = 69; __pyx_clineno = __LINE__; goto __pyx_L1_error;}
  __pyx_v_self->K_mNai = __pyx_t_1;

  /* function exit code */
  __pyx_r = 0;
  goto __pyx_L0;
  __pyx_L1_error:;
  __Pyx_AddTraceback("model.Bondarenko.K_mNai.__set__", __pyx_clineno, __pyx_lineno, __pyx_filename);
  __pyx_r = -1;
  __pyx_L0:;
  __Pyx_RefNannyFinishContext();
  return __pyx_r;
}
```

```
 70:     cdef public double K_mKo
```

```
/* Python wrapper */
static PyObject *__pyx_pw_5model_10Bondarenko_5K_mKo_1__get__(PyObject *__pyx_v_self); /*proto*/
static PyObject *__pyx_pw_5model_10Bondarenko_5K_mKo_1__get__(PyObject *__pyx_v_self) {
  PyObject *__pyx_r = 0;
  __Pyx_RefNannyDeclarations
  __Pyx_RefNannySetupContext("__get__ (wrapper)", 0);
  __pyx_r = __pyx_pf_5model_10Bondarenko_5K_mKo___get__(((struct __pyx_obj_5model_Bondarenko *)__pyx_v_self));

  /* function exit code */
  __Pyx_RefNannyFinishContext();
  return __pyx_r;
}

static PyObject *__pyx_pf_5model_10Bondarenko_5K_mKo___get__(struct __pyx_obj_5model_Bondarenko *__pyx_v_self) {
  PyObject *__pyx_r = NULL;
  __Pyx_RefNannyDeclarations
  __Pyx_RefNannySetupContext("__get__", 0);
  __Pyx_XDECREF(__pyx_r);
  __pyx_t_1 = PyFloat_FromDouble(__pyx_v_self->K_mKo); if (unlikely(!__pyx_t_1)) {__pyx_filename = __pyx_f[0]; __pyx_lineno = 70; __pyx_clineno = __LINE__; goto __pyx_L1_error;}
  __Pyx_GOTREF(__pyx_t_1);
  __pyx_r = __pyx_t_1;
  __pyx_t_1 = 0;
  goto __pyx_L0;

  /* function exit code */
  __pyx_L1_error:;
  __Pyx_XDECREF(__pyx_t_1);
  __Pyx_AddTraceback("model.Bondarenko.K_mKo.__get__", __pyx_clineno, __pyx_lineno, __pyx_filename);
  __pyx_r = NULL;
  __pyx_L0:;
  __Pyx_XGIVEREF(__pyx_r);
  __Pyx_RefNannyFinishContext();
  return __pyx_r;
}

/* Python wrapper */
static int __pyx_pw_5model_10Bondarenko_5K_mKo_3__set__(PyObject *__pyx_v_self, PyObject *__pyx_v_value); /*proto*/
static int __pyx_pw_5model_10Bondarenko_5K_mKo_3__set__(PyObject *__pyx_v_self, PyObject *__pyx_v_value) {
  int __pyx_r;
  __Pyx_RefNannyDeclarations
  __Pyx_RefNannySetupContext("__set__ (wrapper)", 0);
  __pyx_r = __pyx_pf_5model_10Bondarenko_5K_mKo_2__set__(((struct __pyx_obj_5model_Bondarenko *)__pyx_v_self), ((PyObject *)__pyx_v_value));

  /* function exit code */
  __Pyx_RefNannyFinishContext();
  return __pyx_r;
}

static int __pyx_pf_5model_10Bondarenko_5K_mKo_2__set__(struct __pyx_obj_5model_Bondarenko *__pyx_v_self, PyObject *__pyx_v_value) {
  int __pyx_r;
  __Pyx_RefNannyDeclarations
  __Pyx_RefNannySetupContext("__set__", 0);
  __pyx_t_1 = __pyx_PyFloat_AsDouble(__pyx_v_value); if (unlikely((__pyx_t_1 == (double)-1) && PyErr_Occurred())) {__pyx_filename = __pyx_f[0]; __pyx_lineno = 70; __pyx_clineno = __LINE__; goto __pyx_L1_error;}
  __pyx_v_self->K_mKo = __pyx_t_1;

  /* function exit code */
  __pyx_r = 0;
  goto __pyx_L0;
  __pyx_L1_error:;
  __Pyx_AddTraceback("model.Bondarenko.K_mKo.__set__", __pyx_clineno, __pyx_lineno, __pyx_filename);
  __pyx_r = -1;
  __pyx_L0:;
  __Pyx_RefNannyFinishContext();
  return __pyx_r;
}
```

```
 71:     cdef public double I_maxpCa
```

```
/* Python wrapper */
static PyObject *__pyx_pw_5model_10Bondarenko_8I_maxpCa_1__get__(PyObject *__pyx_v_self); /*proto*/
static PyObject *__pyx_pw_5model_10Bondarenko_8I_maxpCa_1__get__(PyObject *__pyx_v_self) {
  PyObject *__pyx_r = 0;
  __Pyx_RefNannyDeclarations
  __Pyx_RefNannySetupContext("__get__ (wrapper)", 0);
  __pyx_r = __pyx_pf_5model_10Bondarenko_8I_maxpCa___get__(((struct __pyx_obj_5model_Bondarenko *)__pyx_v_self));

  /* function exit code */
  __Pyx_RefNannyFinishContext();
  return __pyx_r;
}

static PyObject *__pyx_pf_5model_10Bondarenko_8I_maxpCa___get__(struct __pyx_obj_5model_Bondarenko *__pyx_v_self) {
  PyObject *__pyx_r = NULL;
  __Pyx_RefNannyDeclarations
  __Pyx_RefNannySetupContext("__get__", 0);
  __Pyx_XDECREF(__pyx_r);
  __pyx_t_1 = PyFloat_FromDouble(__pyx_v_self->I_maxpCa); if (unlikely(!__pyx_t_1)) {__pyx_filename = __pyx_f[0]; __pyx_lineno = 71; __pyx_clineno = __LINE__; goto __pyx_L1_error;}
  __Pyx_GOTREF(__pyx_t_1);
  __pyx_r = __pyx_t_1;
  __pyx_t_1 = 0;
  goto __pyx_L0;

  /* function exit code */
  __pyx_L1_error:;
  __Pyx_XDECREF(__pyx_t_1);
  __Pyx_AddTraceback("model.Bondarenko.I_maxpCa.__get__", __pyx_clineno, __pyx_lineno, __pyx_filename);
  __pyx_r = NULL;
  __pyx_L0:;
  __Pyx_XGIVEREF(__pyx_r);
  __Pyx_RefNannyFinishContext();
  return __pyx_r;
}

/* Python wrapper */
static int __pyx_pw_5model_10Bondarenko_8I_maxpCa_3__set__(PyObject *__pyx_v_self, PyObject *__pyx_v_value); /*proto*/
static int __pyx_pw_5model_10Bondarenko_8I_maxpCa_3__set__(PyObject *__pyx_v_self, PyObject *__pyx_v_value) {
  int __pyx_r;
  __Pyx_RefNannyDeclarations
  __Pyx_RefNannySetupContext("__set__ (wrapper)", 0);
  __pyx_r = __pyx_pf_5model_10Bondarenko_8I_maxpCa_2__set__(((struct __pyx_obj_5model_Bondarenko *)__pyx_v_self), ((PyObject *)__pyx_v_value));

  /* function exit code */
  __Pyx_RefNannyFinishContext();
  return __pyx_r;
}

static int __pyx_pf_5model_10Bondarenko_8I_maxpCa_2__set__(struct __pyx_obj_5model_Bondarenko *__pyx_v_self, PyObject *__pyx_v_value) {
  int __pyx_r;
  __Pyx_RefNannyDeclarations
  __Pyx_RefNannySetupContext("__set__", 0);
  __pyx_t_1 = __pyx_PyFloat_AsDouble(__pyx_v_value); if (unlikely((__pyx_t_1 == (double)-1) && PyErr_Occurred())) {__pyx_filename = __pyx_f[0]; __pyx_lineno = 71; __pyx_clineno = __LINE__; goto __pyx_L1_error;}
  __pyx_v_self->I_maxpCa = __pyx_t_1;

  /* function exit code */
  __pyx_r = 0;
  goto __pyx_L0;
  __pyx_L1_error:;
  __Pyx_AddTraceback("model.Bondarenko.I_maxpCa.__set__", __pyx_clineno, __pyx_lineno, __pyx_filename);
  __pyx_r = -1;
  __pyx_L0:;
  __Pyx_RefNannyFinishContext();
  return __pyx_r;
}
```

```
 72:     cdef public double k_mpCa
```

```
/* Python wrapper */
static PyObject *__pyx_pw_5model_10Bondarenko_6k_mpCa_1__get__(PyObject *__pyx_v_self); /*proto*/
static PyObject *__pyx_pw_5model_10Bondarenko_6k_mpCa_1__get__(PyObject *__pyx_v_self) {
  PyObject *__pyx_r = 0;
  __Pyx_RefNannyDeclarations
  __Pyx_RefNannySetupContext("__get__ (wrapper)", 0);
  __pyx_r = __pyx_pf_5model_10Bondarenko_6k_mpCa___get__(((struct __pyx_obj_5model_Bondarenko *)__pyx_v_self));

  /* function exit code */
  __Pyx_RefNannyFinishContext();
  return __pyx_r;
}

static PyObject *__pyx_pf_5model_10Bondarenko_6k_mpCa___get__(struct __pyx_obj_5model_Bondarenko *__pyx_v_self) {
  PyObject *__pyx_r = NULL;
  __Pyx_RefNannyDeclarations
  __Pyx_RefNannySetupContext("__get__", 0);
  __Pyx_XDECREF(__pyx_r);
  __pyx_t_1 = PyFloat_FromDouble(__pyx_v_self->k_mpCa); if (unlikely(!__pyx_t_1)) {__pyx_filename = __pyx_f[0]; __pyx_lineno = 72; __pyx_clineno = __LINE__; goto __pyx_L1_error;}
  __Pyx_GOTREF(__pyx_t_1);
  __pyx_r = __pyx_t_1;
  __pyx_t_1 = 0;
  goto __pyx_L0;

  /* function exit code */
  __pyx_L1_error:;
  __Pyx_XDECREF(__pyx_t_1);
  __Pyx_AddTraceback("model.Bondarenko.k_mpCa.__get__", __pyx_clineno, __pyx_lineno, __pyx_filename);
  __pyx_r = NULL;
  __pyx_L0:;
  __Pyx_XGIVEREF(__pyx_r);
  __Pyx_RefNannyFinishContext();
  return __pyx_r;
}

/* Python wrapper */
static int __pyx_pw_5model_10Bondarenko_6k_mpCa_3__set__(PyObject *__pyx_v_self, PyObject *__pyx_v_value); /*proto*/
static int __pyx_pw_5model_10Bondarenko_6k_mpCa_3__set__(PyObject *__pyx_v_self, PyObject *__pyx_v_value) {
  int __pyx_r;
  __Pyx_RefNannyDeclarations
  __Pyx_RefNannySetupContext("__set__ (wrapper)", 0);
  __pyx_r = __pyx_pf_5model_10Bondarenko_6k_mpCa_2__set__(((struct __pyx_obj_5model_Bondarenko *)__pyx_v_self), ((PyObject *)__pyx_v_value));

  /* function exit code */
  __Pyx_RefNannyFinishContext();
  return __pyx_r;
}

static int __pyx_pf_5model_10Bondarenko_6k_mpCa_2__set__(struct __pyx_obj_5model_Bondarenko *__pyx_v_self, PyObject *__pyx_v_value) {
  int __pyx_r;
  __Pyx_RefNannyDeclarations
  __Pyx_RefNannySetupContext("__set__", 0);
  __pyx_t_1 = __pyx_PyFloat_AsDouble(__pyx_v_value); if (unlikely((__pyx_t_1 == (double)-1) && PyErr_Occurred())) {__pyx_filename = __pyx_f[0]; __pyx_lineno = 72; __pyx_clineno = __LINE__; goto __pyx_L1_error;}
  __pyx_v_self->k_mpCa = __pyx_t_1;

  /* function exit code */
  __pyx_r = 0;
  goto __pyx_L0;
  __pyx_L1_error:;
  __Pyx_AddTraceback("model.Bondarenko.k_mpCa.__set__", __pyx_clineno, __pyx_lineno, __pyx_filename);
  __pyx_r = -1;
  __pyx_L0:;
  __Pyx_RefNannyFinishContext();
  return __pyx_r;
}
```

```
 73:     cdef public double G_Cab
```

```
/* Python wrapper */
static PyObject *__pyx_pw_5model_10Bondarenko_5G_Cab_1__get__(PyObject *__pyx_v_self); /*proto*/
static PyObject *__pyx_pw_5model_10Bondarenko_5G_Cab_1__get__(PyObject *__pyx_v_self) {
  PyObject *__pyx_r = 0;
  __Pyx_RefNannyDeclarations
  __Pyx_RefNannySetupContext("__get__ (wrapper)", 0);
  __pyx_r = __pyx_pf_5model_10Bondarenko_5G_Cab___get__(((struct __pyx_obj_5model_Bondarenko *)__pyx_v_self));

  /* function exit code */
  __Pyx_RefNannyFinishContext();
  return __pyx_r;
}

static PyObject *__pyx_pf_5model_10Bondarenko_5G_Cab___get__(struct __pyx_obj_5model_Bondarenko *__pyx_v_self) {
  PyObject *__pyx_r = NULL;
  __Pyx_RefNannyDeclarations
  __Pyx_RefNannySetupContext("__get__", 0);
  __Pyx_XDECREF(__pyx_r);
  __pyx_t_1 = PyFloat_FromDouble(__pyx_v_self->G_Cab); if (unlikely(!__pyx_t_1)) {__pyx_filename = __pyx_f[0]; __pyx_lineno = 73; __pyx_clineno = __LINE__; goto __pyx_L1_error;}
  __Pyx_GOTREF(__pyx_t_1);
  __pyx_r = __pyx_t_1;
  __pyx_t_1 = 0;
  goto __pyx_L0;

  /* function exit code */
  __pyx_L1_error:;
  __Pyx_XDECREF(__pyx_t_1);
  __Pyx_AddTraceback("model.Bondarenko.G_Cab.__get__", __pyx_clineno, __pyx_lineno, __pyx_filename);
  __pyx_r = NULL;
  __pyx_L0:;
  __Pyx_XGIVEREF(__pyx_r);
  __Pyx_RefNannyFinishContext();
  return __pyx_r;
}

/* Python wrapper */
static int __pyx_pw_5model_10Bondarenko_5G_Cab_3__set__(PyObject *__pyx_v_self, PyObject *__pyx_v_value); /*proto*/
static int __pyx_pw_5model_10Bondarenko_5G_Cab_3__set__(PyObject *__pyx_v_self, PyObject *__pyx_v_value) {
  int __pyx_r;
  __Pyx_RefNannyDeclarations
  __Pyx_RefNannySetupContext("__set__ (wrapper)", 0);
  __pyx_r = __pyx_pf_5model_10Bondarenko_5G_Cab_2__set__(((struct __pyx_obj_5model_Bondarenko *)__pyx_v_self), ((PyObject *)__pyx_v_value));

  /* function exit code */
  __Pyx_RefNannyFinishContext();
  return __pyx_r;
}

static int __pyx_pf_5model_10Bondarenko_5G_Cab_2__set__(struct __pyx_obj_5model_Bondarenko *__pyx_v_self, PyObject *__pyx_v_value) {
  int __pyx_r;
  __Pyx_RefNannyDeclarations
  __Pyx_RefNannySetupContext("__set__", 0);
  __pyx_t_1 = __pyx_PyFloat_AsDouble(__pyx_v_value); if (unlikely((__pyx_t_1 == (double)-1) && PyErr_Occurred())) {__pyx_filename = __pyx_f[0]; __pyx_lineno = 73; __pyx_clineno = __LINE__; goto __pyx_L1_error;}
  __pyx_v_self->G_Cab = __pyx_t_1;

  /* function exit code */
  __pyx_r = 0;
  goto __pyx_L0;
  __pyx_L1_error:;
  __Pyx_AddTraceback("model.Bondarenko.G_Cab.__set__", __pyx_clineno, __pyx_lineno, __pyx_filename);
  __pyx_r = -1;
  __pyx_L0:;
  __Pyx_RefNannyFinishContext();
  return __pyx_r;
}
```

```
 74:     cdef public double G_Na
```

```
/* Python wrapper */
static PyObject *__pyx_pw_5model_10Bondarenko_4G_Na_1__get__(PyObject *__pyx_v_self); /*proto*/
static PyObject *__pyx_pw_5model_10Bondarenko_4G_Na_1__get__(PyObject *__pyx_v_self) {
  PyObject *__pyx_r = 0;
  __Pyx_RefNannyDeclarations
  __Pyx_RefNannySetupContext("__get__ (wrapper)", 0);
  __pyx_r = __pyx_pf_5model_10Bondarenko_4G_Na___get__(((struct __pyx_obj_5model_Bondarenko *)__pyx_v_self));

  /* function exit code */
  __Pyx_RefNannyFinishContext();
  return __pyx_r;
}

static PyObject *__pyx_pf_5model_10Bondarenko_4G_Na___get__(struct __pyx_obj_5model_Bondarenko *__pyx_v_self) {
  PyObject *__pyx_r = NULL;
  __Pyx_RefNannyDeclarations
  __Pyx_RefNannySetupContext("__get__", 0);
  __Pyx_XDECREF(__pyx_r);
  __pyx_t_1 = PyFloat_FromDouble(__pyx_v_self->G_Na); if (unlikely(!__pyx_t_1)) {__pyx_filename = __pyx_f[0]; __pyx_lineno = 74; __pyx_clineno = __LINE__; goto __pyx_L1_error;}
  __Pyx_GOTREF(__pyx_t_1);
  __pyx_r = __pyx_t_1;
  __pyx_t_1 = 0;
  goto __pyx_L0;

  /* function exit code */
  __pyx_L1_error:;
  __Pyx_XDECREF(__pyx_t_1);
  __Pyx_AddTraceback("model.Bondarenko.G_Na.__get__", __pyx_clineno, __pyx_lineno, __pyx_filename);
  __pyx_r = NULL;
  __pyx_L0:;
  __Pyx_XGIVEREF(__pyx_r);
  __Pyx_RefNannyFinishContext();
  return __pyx_r;
}

/* Python wrapper */
static int __pyx_pw_5model_10Bondarenko_4G_Na_3__set__(PyObject *__pyx_v_self, PyObject *__pyx_v_value); /*proto*/
static int __pyx_pw_5model_10Bondarenko_4G_Na_3__set__(PyObject *__pyx_v_self, PyObject *__pyx_v_value) {
  int __pyx_r;
  __Pyx_RefNannyDeclarations
  __Pyx_RefNannySetupContext("__set__ (wrapper)", 0);
  __pyx_r = __pyx_pf_5model_10Bondarenko_4G_Na_2__set__(((struct __pyx_obj_5model_Bondarenko *)__pyx_v_self), ((PyObject *)__pyx_v_value));

  /* function exit code */
  __Pyx_RefNannyFinishContext();
  return __pyx_r;
}

static int __pyx_pf_5model_10Bondarenko_4G_Na_2__set__(struct __pyx_obj_5model_Bondarenko *__pyx_v_self, PyObject *__pyx_v_value) {
  int __pyx_r;
  __Pyx_RefNannyDeclarations
  __Pyx_RefNannySetupContext("__set__", 0);
  __pyx_t_1 = __pyx_PyFloat_AsDouble(__pyx_v_value); if (unlikely((__pyx_t_1 == (double)-1) && PyErr_Occurred())) {__pyx_filename = __pyx_f[0]; __pyx_lineno = 74; __pyx_clineno = __LINE__; goto __pyx_L1_error;}
  __pyx_v_self->G_Na = __pyx_t_1;

  /* function exit code */
  __pyx_r = 0;
  goto __pyx_L0;
  __pyx_L1_error:;
  __Pyx_AddTraceback("model.Bondarenko.G_Na.__set__", __pyx_clineno, __pyx_lineno, __pyx_filename);
  __pyx_r = -1;
  __pyx_L0:;
  __Pyx_RefNannyFinishContext();
  return __pyx_r;
}
```

```
 75:     cdef public double G_Nab
```

```
/* Python wrapper */
static PyObject *__pyx_pw_5model_10Bondarenko_5G_Nab_1__get__(PyObject *__pyx_v_self); /*proto*/
static PyObject *__pyx_pw_5model_10Bondarenko_5G_Nab_1__get__(PyObject *__pyx_v_self) {
  PyObject *__pyx_r = 0;
  __Pyx_RefNannyDeclarations
  __Pyx_RefNannySetupContext("__get__ (wrapper)", 0);
  __pyx_r = __pyx_pf_5model_10Bondarenko_5G_Nab___get__(((struct __pyx_obj_5model_Bondarenko *)__pyx_v_self));

  /* function exit code */
  __Pyx_RefNannyFinishContext();
  return __pyx_r;
}

static PyObject *__pyx_pf_5model_10Bondarenko_5G_Nab___get__(struct __pyx_obj_5model_Bondarenko *__pyx_v_self) {
  PyObject *__pyx_r = NULL;
  __Pyx_RefNannyDeclarations
  __Pyx_RefNannySetupContext("__get__", 0);
  __Pyx_XDECREF(__pyx_r);
  __pyx_t_1 = PyFloat_FromDouble(__pyx_v_self->G_Nab); if (unlikely(!__pyx_t_1)) {__pyx_filename = __pyx_f[0]; __pyx_lineno = 75; __pyx_clineno = __LINE__; goto __pyx_L1_error;}
  __Pyx_GOTREF(__pyx_t_1);
  __pyx_r = __pyx_t_1;
  __pyx_t_1 = 0;
  goto __pyx_L0;

  /* function exit code */
  __pyx_L1_error:;
  __Pyx_XDECREF(__pyx_t_1);
  __Pyx_AddTraceback("model.Bondarenko.G_Nab.__get__", __pyx_clineno, __pyx_lineno, __pyx_filename);
  __pyx_r = NULL;
  __pyx_L0:;
  __Pyx_XGIVEREF(__pyx_r);
  __Pyx_RefNannyFinishContext();
  return __pyx_r;
}

/* Python wrapper */
static int __pyx_pw_5model_10Bondarenko_5G_Nab_3__set__(PyObject *__pyx_v_self, PyObject *__pyx_v_value); /*proto*/
static int __pyx_pw_5model_10Bondarenko_5G_Nab_3__set__(PyObject *__pyx_v_self, PyObject *__pyx_v_value) {
  int __pyx_r;
  __Pyx_RefNannyDeclarations
  __Pyx_RefNannySetupContext("__set__ (wrapper)", 0);
  __pyx_r = __pyx_pf_5model_10Bondarenko_5G_Nab_2__set__(((struct __pyx_obj_5model_Bondarenko *)__pyx_v_self), ((PyObject *)__pyx_v_value));

  /* function exit code */
  __Pyx_RefNannyFinishContext();
  return __pyx_r;
}

static int __pyx_pf_5model_10Bondarenko_5G_Nab_2__set__(struct __pyx_obj_5model_Bondarenko *__pyx_v_self, PyObject *__pyx_v_value) {
  int __pyx_r;
  __Pyx_RefNannyDeclarations
  __Pyx_RefNannySetupContext("__set__", 0);
  __pyx_t_1 = __pyx_PyFloat_AsDouble(__pyx_v_value); if (unlikely((__pyx_t_1 == (double)-1) && PyErr_Occurred())) {__pyx_filename = __pyx_f[0]; __pyx_lineno = 75; __pyx_clineno = __LINE__; goto __pyx_L1_error;}
  __pyx_v_self->G_Nab = __pyx_t_1;

  /* function exit code */
  __pyx_r = 0;
  goto __pyx_L0;
  __pyx_L1_error:;
  __Pyx_AddTraceback("model.Bondarenko.G_Nab.__set__", __pyx_clineno, __pyx_lineno, __pyx_filename);
  __pyx_r = -1;
  __pyx_L0:;
  __Pyx_RefNannyFinishContext();
  return __pyx_r;
}
```

```
 76:     cdef public double G_Ktof        #(apex)
```

```
/* Python wrapper */
static PyObject *__pyx_pw_5model_10Bondarenko_6G_Ktof_1__get__(PyObject *__pyx_v_self); /*proto*/
static PyObject *__pyx_pw_5model_10Bondarenko_6G_Ktof_1__get__(PyObject *__pyx_v_self) {
  PyObject *__pyx_r = 0;
  __Pyx_RefNannyDeclarations
  __Pyx_RefNannySetupContext("__get__ (wrapper)", 0);
  __pyx_r = __pyx_pf_5model_10Bondarenko_6G_Ktof___get__(((struct __pyx_obj_5model_Bondarenko *)__pyx_v_self));

  /* function exit code */
  __Pyx_RefNannyFinishContext();
  return __pyx_r;
}

static PyObject *__pyx_pf_5model_10Bondarenko_6G_Ktof___get__(struct __pyx_obj_5model_Bondarenko *__pyx_v_self) {
  PyObject *__pyx_r = NULL;
  __Pyx_RefNannyDeclarations
  __Pyx_RefNannySetupContext("__get__", 0);
  __Pyx_XDECREF(__pyx_r);
  __pyx_t_1 = PyFloat_FromDouble(__pyx_v_self->G_Ktof); if (unlikely(!__pyx_t_1)) {__pyx_filename = __pyx_f[0]; __pyx_lineno = 76; __pyx_clineno = __LINE__; goto __pyx_L1_error;}
  __Pyx_GOTREF(__pyx_t_1);
  __pyx_r = __pyx_t_1;
  __pyx_t_1 = 0;
  goto __pyx_L0;

  /* function exit code */
  __pyx_L1_error:;
  __Pyx_XDECREF(__pyx_t_1);
  __Pyx_AddTraceback("model.Bondarenko.G_Ktof.__get__", __pyx_clineno, __pyx_lineno, __pyx_filename);
  __pyx_r = NULL;
  __pyx_L0:;
  __Pyx_XGIVEREF(__pyx_r);
  __Pyx_RefNannyFinishContext();
  return __pyx_r;
}

/* Python wrapper */
static int __pyx_pw_5model_10Bondarenko_6G_Ktof_3__set__(PyObject *__pyx_v_self, PyObject *__pyx_v_value); /*proto*/
static int __pyx_pw_5model_10Bondarenko_6G_Ktof_3__set__(PyObject *__pyx_v_self, PyObject *__pyx_v_value) {
  int __pyx_r;
  __Pyx_RefNannyDeclarations
  __Pyx_RefNannySetupContext("__set__ (wrapper)", 0);
  __pyx_r = __pyx_pf_5model_10Bondarenko_6G_Ktof_2__set__(((struct __pyx_obj_5model_Bondarenko *)__pyx_v_self), ((PyObject *)__pyx_v_value));

  /* function exit code */
  __Pyx_RefNannyFinishContext();
  return __pyx_r;
}

static int __pyx_pf_5model_10Bondarenko_6G_Ktof_2__set__(struct __pyx_obj_5model_Bondarenko *__pyx_v_self, PyObject *__pyx_v_value) {
  int __pyx_r;
  __Pyx_RefNannyDeclarations
  __Pyx_RefNannySetupContext("__set__", 0);
  __pyx_t_1 = __pyx_PyFloat_AsDouble(__pyx_v_value); if (unlikely((__pyx_t_1 == (double)-1) && PyErr_Occurred())) {__pyx_filename = __pyx_f[0]; __pyx_lineno = 76; __pyx_clineno = __LINE__; goto __pyx_L1_error;}
  __pyx_v_self->G_Ktof = __pyx_t_1;

  /* function exit code */
  __pyx_r = 0;
  goto __pyx_L0;
  __pyx_L1_error:;
  __Pyx_AddTraceback("model.Bondarenko.G_Ktof.__set__", __pyx_clineno, __pyx_lineno, __pyx_filename);
  __pyx_r = -1;
  __pyx_L0:;
  __Pyx_RefNannyFinishContext();
  return __pyx_r;
}
```

```
 77:     #cdef public double G_Ktof       #(septum)
```

```
 78:     cdef public double G_Ks
```

```
/* Python wrapper */
static PyObject *__pyx_pw_5model_10Bondarenko_4G_Ks_1__get__(PyObject *__pyx_v_self); /*proto*/
static PyObject *__pyx_pw_5model_10Bondarenko_4G_Ks_1__get__(PyObject *__pyx_v_self) {
  PyObject *__pyx_r = 0;
  __Pyx_RefNannyDeclarations
  __Pyx_RefNannySetupContext("__get__ (wrapper)", 0);
  __pyx_r = __pyx_pf_5model_10Bondarenko_4G_Ks___get__(((struct __pyx_obj_5model_Bondarenko *)__pyx_v_self));

  /* function exit code */
  __Pyx_RefNannyFinishContext();
  return __pyx_r;
}

static PyObject *__pyx_pf_5model_10Bondarenko_4G_Ks___get__(struct __pyx_obj_5model_Bondarenko *__pyx_v_self) {
  PyObject *__pyx_r = NULL;
  __Pyx_RefNannyDeclarations
  __Pyx_RefNannySetupContext("__get__", 0);
  __Pyx_XDECREF(__pyx_r);
  __pyx_t_1 = PyFloat_FromDouble(__pyx_v_self->G_Ks); if (unlikely(!__pyx_t_1)) {__pyx_filename = __pyx_f[0]; __pyx_lineno = 78; __pyx_clineno = __LINE__; goto __pyx_L1_error;}
  __Pyx_GOTREF(__pyx_t_1);
  __pyx_r = __pyx_t_1;
  __pyx_t_1 = 0;
  goto __pyx_L0;

  /* function exit code */
  __pyx_L1_error:;
  __Pyx_XDECREF(__pyx_t_1);
  __Pyx_AddTraceback("model.Bondarenko.G_Ks.__get__", __pyx_clineno, __pyx_lineno, __pyx_filename);
  __pyx_r = NULL;
  __pyx_L0:;
  __Pyx_XGIVEREF(__pyx_r);
  __Pyx_RefNannyFinishContext();
  return __pyx_r;
}

/* Python wrapper */
static int __pyx_pw_5model_10Bondarenko_4G_Ks_3__set__(PyObject *__pyx_v_self, PyObject *__pyx_v_value); /*proto*/
static int __pyx_pw_5model_10Bondarenko_4G_Ks_3__set__(PyObject *__pyx_v_self, PyObject *__pyx_v_value) {
  int __pyx_r;
  __Pyx_RefNannyDeclarations
  __Pyx_RefNannySetupContext("__set__ (wrapper)", 0);
  __pyx_r = __pyx_pf_5model_10Bondarenko_4G_Ks_2__set__(((struct __pyx_obj_5model_Bondarenko *)__pyx_v_self), ((PyObject *)__pyx_v_value));

  /* function exit code */
  __Pyx_RefNannyFinishContext();
  return __pyx_r;
}

static int __pyx_pf_5model_10Bondarenko_4G_Ks_2__set__(struct __pyx_obj_5model_Bondarenko *__pyx_v_self, PyObject *__pyx_v_value) {
  int __pyx_r;
  __Pyx_RefNannyDeclarations
  __Pyx_RefNannySetupContext("__set__", 0);
  __pyx_t_1 = __pyx_PyFloat_AsDouble(__pyx_v_value); if (unlikely((__pyx_t_1 == (double)-1) && PyErr_Occurred())) {__pyx_filename = __pyx_f[0]; __pyx_lineno = 78; __pyx_clineno = __LINE__; goto __pyx_L1_error;}
  __pyx_v_self->G_Ks = __pyx_t_1;

  /* function exit code */
  __pyx_r = 0;
  goto __pyx_L0;
  __pyx_L1_error:;
  __Pyx_AddTraceback("model.Bondarenko.G_Ks.__set__", __pyx_clineno, __pyx_lineno, __pyx_filename);
  __pyx_r = -1;
  __pyx_L0:;
  __Pyx_RefNannyFinishContext();
  return __pyx_r;
}
```

```
 79:     cdef public double G_Ktos        #(apex)
```

```
/* Python wrapper */
static PyObject *__pyx_pw_5model_10Bondarenko_6G_Ktos_1__get__(PyObject *__pyx_v_self); /*proto*/
static PyObject *__pyx_pw_5model_10Bondarenko_6G_Ktos_1__get__(PyObject *__pyx_v_self) {
  PyObject *__pyx_r = 0;
  __Pyx_RefNannyDeclarations
  __Pyx_RefNannySetupContext("__get__ (wrapper)", 0);
  __pyx_r = __pyx_pf_5model_10Bondarenko_6G_Ktos___get__(((struct __pyx_obj_5model_Bondarenko *)__pyx_v_self));

  /* function exit code */
  __Pyx_RefNannyFinishContext();
  return __pyx_r;
}

static PyObject *__pyx_pf_5model_10Bondarenko_6G_Ktos___get__(struct __pyx_obj_5model_Bondarenko *__pyx_v_self) {
  PyObject *__pyx_r = NULL;
  __Pyx_RefNannyDeclarations
  __Pyx_RefNannySetupContext("__get__", 0);
  __Pyx_XDECREF(__pyx_r);
  __pyx_t_1 = PyFloat_FromDouble(__pyx_v_self->G_Ktos); if (unlikely(!__pyx_t_1)) {__pyx_filename = __pyx_f[0]; __pyx_lineno = 79; __pyx_clineno = __LINE__; goto __pyx_L1_error;}
  __Pyx_GOTREF(__pyx_t_1);
  __pyx_r = __pyx_t_1;
  __pyx_t_1 = 0;
  goto __pyx_L0;

  /* function exit code */
  __pyx_L1_error:;
  __Pyx_XDECREF(__pyx_t_1);
  __Pyx_AddTraceback("model.Bondarenko.G_Ktos.__get__", __pyx_clineno, __pyx_lineno, __pyx_filename);
  __pyx_r = NULL;
  __pyx_L0:;
  __Pyx_XGIVEREF(__pyx_r);
  __Pyx_RefNannyFinishContext();
  return __pyx_r;
}

/* Python wrapper */
static int __pyx_pw_5model_10Bondarenko_6G_Ktos_3__set__(PyObject *__pyx_v_self, PyObject *__pyx_v_value); /*proto*/
static int __pyx_pw_5model_10Bondarenko_6G_Ktos_3__set__(PyObject *__pyx_v_self, PyObject *__pyx_v_value) {
  int __pyx_r;
  __Pyx_RefNannyDeclarations
  __Pyx_RefNannySetupContext("__set__ (wrapper)", 0);
  __pyx_r = __pyx_pf_5model_10Bondarenko_6G_Ktos_2__set__(((struct __pyx_obj_5model_Bondarenko *)__pyx_v_self), ((PyObject *)__pyx_v_value));

  /* function exit code */
  __Pyx_RefNannyFinishContext();
  return __pyx_r;
}

static int __pyx_pf_5model_10Bondarenko_6G_Ktos_2__set__(struct __pyx_obj_5model_Bondarenko *__pyx_v_self, PyObject *__pyx_v_value) {
  int __pyx_r;
  __Pyx_RefNannyDeclarations
  __Pyx_RefNannySetupContext("__set__", 0);
  __pyx_t_1 = __pyx_PyFloat_AsDouble(__pyx_v_value); if (unlikely((__pyx_t_1 == (double)-1) && PyErr_Occurred())) {__pyx_filename = __pyx_f[0]; __pyx_lineno = 79; __pyx_clineno = __LINE__; goto __pyx_L1_error;}
  __pyx_v_self->G_Ktos = __pyx_t_1;

  /* function exit code */
  __pyx_r = 0;
  goto __pyx_L0;
  __pyx_L1_error:;
  __Pyx_AddTraceback("model.Bondarenko.G_Ktos.__set__", __pyx_clineno, __pyx_lineno, __pyx_filename);
  __pyx_r = -1;
  __pyx_L0:;
  __Pyx_RefNannyFinishContext();
  return __pyx_r;
}
```

```
 80:     cdef public double G_Kur         #(apex)
```

```
/* Python wrapper */
static PyObject *__pyx_pw_5model_10Bondarenko_5G_Kur_1__get__(PyObject *__pyx_v_self); /*proto*/
static PyObject *__pyx_pw_5model_10Bondarenko_5G_Kur_1__get__(PyObject *__pyx_v_self) {
  PyObject *__pyx_r = 0;
  __Pyx_RefNannyDeclarations
  __Pyx_RefNannySetupContext("__get__ (wrapper)", 0);
  __pyx_r = __pyx_pf_5model_10Bondarenko_5G_Kur___get__(((struct __pyx_obj_5model_Bondarenko *)__pyx_v_self));

  /* function exit code */
  __Pyx_RefNannyFinishContext();
  return __pyx_r;
}

static PyObject *__pyx_pf_5model_10Bondarenko_5G_Kur___get__(struct __pyx_obj_5model_Bondarenko *__pyx_v_self) {
  PyObject *__pyx_r = NULL;
  __Pyx_RefNannyDeclarations
  __Pyx_RefNannySetupContext("__get__", 0);
  __Pyx_XDECREF(__pyx_r);
  __pyx_t_1 = PyFloat_FromDouble(__pyx_v_self->G_Kur); if (unlikely(!__pyx_t_1)) {__pyx_filename = __pyx_f[0]; __pyx_lineno = 80; __pyx_clineno = __LINE__; goto __pyx_L1_error;}
  __Pyx_GOTREF(__pyx_t_1);
  __pyx_r = __pyx_t_1;
  __pyx_t_1 = 0;
  goto __pyx_L0;

  /* function exit code */
  __pyx_L1_error:;
  __Pyx_XDECREF(__pyx_t_1);
  __Pyx_AddTraceback("model.Bondarenko.G_Kur.__get__", __pyx_clineno, __pyx_lineno, __pyx_filename);
  __pyx_r = NULL;
  __pyx_L0:;
  __Pyx_XGIVEREF(__pyx_r);
  __Pyx_RefNannyFinishContext();
  return __pyx_r;
}

/* Python wrapper */
static int __pyx_pw_5model_10Bondarenko_5G_Kur_3__set__(PyObject *__pyx_v_self, PyObject *__pyx_v_value); /*proto*/
static int __pyx_pw_5model_10Bondarenko_5G_Kur_3__set__(PyObject *__pyx_v_self, PyObject *__pyx_v_value) {
  int __pyx_r;
  __Pyx_RefNannyDeclarations
  __Pyx_RefNannySetupContext("__set__ (wrapper)", 0);
  __pyx_r = __pyx_pf_5model_10Bondarenko_5G_Kur_2__set__(((struct __pyx_obj_5model_Bondarenko *)__pyx_v_self), ((PyObject *)__pyx_v_value));

  /* function exit code */
  __Pyx_RefNannyFinishContext();
  return __pyx_r;
}

static int __pyx_pf_5model_10Bondarenko_5G_Kur_2__set__(struct __pyx_obj_5model_Bondarenko *__pyx_v_self, PyObject *__pyx_v_value) {
  int __pyx_r;
  __Pyx_RefNannyDeclarations
  __Pyx_RefNannySetupContext("__set__", 0);
  __pyx_t_1 = __pyx_PyFloat_AsDouble(__pyx_v_value); if (unlikely((__pyx_t_1 == (double)-1) && PyErr_Occurred())) {__pyx_filename = __pyx_f[0]; __pyx_lineno = 80; __pyx_clineno = __LINE__; goto __pyx_L1_error;}
  __pyx_v_self->G_Kur = __pyx_t_1;

  /* function exit code */
  __pyx_r = 0;
  goto __pyx_L0;
  __pyx_L1_error:;
  __Pyx_AddTraceback("model.Bondarenko.G_Kur.__set__", __pyx_clineno, __pyx_lineno, __pyx_filename);
  __pyx_r = -1;
  __pyx_L0:;
  __Pyx_RefNannyFinishContext();
  return __pyx_r;
}
```

```
 81:     cdef public double G_Kss         #(apex)
```

```
/* Python wrapper */
static PyObject *__pyx_pw_5model_10Bondarenko_5G_Kss_1__get__(PyObject *__pyx_v_self); /*proto*/
static PyObject *__pyx_pw_5model_10Bondarenko_5G_Kss_1__get__(PyObject *__pyx_v_self) {
  PyObject *__pyx_r = 0;
  __Pyx_RefNannyDeclarations
  __Pyx_RefNannySetupContext("__get__ (wrapper)", 0);
  __pyx_r = __pyx_pf_5model_10Bondarenko_5G_Kss___get__(((struct __pyx_obj_5model_Bondarenko *)__pyx_v_self));

  /* function exit code */
  __Pyx_RefNannyFinishContext();
  return __pyx_r;
}

static PyObject *__pyx_pf_5model_10Bondarenko_5G_Kss___get__(struct __pyx_obj_5model_Bondarenko *__pyx_v_self) {
  PyObject *__pyx_r = NULL;
  __Pyx_RefNannyDeclarations
  __Pyx_RefNannySetupContext("__get__", 0);
  __Pyx_XDECREF(__pyx_r);
  __pyx_t_1 = PyFloat_FromDouble(__pyx_v_self->G_Kss); if (unlikely(!__pyx_t_1)) {__pyx_filename = __pyx_f[0]; __pyx_lineno = 81; __pyx_clineno = __LINE__; goto __pyx_L1_error;}
  __Pyx_GOTREF(__pyx_t_1);
  __pyx_r = __pyx_t_1;
  __pyx_t_1 = 0;
  goto __pyx_L0;

  /* function exit code */
  __pyx_L1_error:;
  __Pyx_XDECREF(__pyx_t_1);
  __Pyx_AddTraceback("model.Bondarenko.G_Kss.__get__", __pyx_clineno, __pyx_lineno, __pyx_filename);
  __pyx_r = NULL;
  __pyx_L0:;
  __Pyx_XGIVEREF(__pyx_r);
  __Pyx_RefNannyFinishContext();
  return __pyx_r;
}

/* Python wrapper */
static int __pyx_pw_5model_10Bondarenko_5G_Kss_3__set__(PyObject *__pyx_v_self, PyObject *__pyx_v_value); /*proto*/
static int __pyx_pw_5model_10Bondarenko_5G_Kss_3__set__(PyObject *__pyx_v_self, PyObject *__pyx_v_value) {
  int __pyx_r;
  __Pyx_RefNannyDeclarations
  __Pyx_RefNannySetupContext("__set__ (wrapper)", 0);
  __pyx_r = __pyx_pf_5model_10Bondarenko_5G_Kss_2__set__(((struct __pyx_obj_5model_Bondarenko *)__pyx_v_self), ((PyObject *)__pyx_v_value));

  /* function exit code */
  __Pyx_RefNannyFinishContext();
  return __pyx_r;
}

static int __pyx_pf_5model_10Bondarenko_5G_Kss_2__set__(struct __pyx_obj_5model_Bondarenko *__pyx_v_self, PyObject *__pyx_v_value) {
  int __pyx_r;
  __Pyx_RefNannyDeclarations
  __Pyx_RefNannySetupContext("__set__", 0);
  __pyx_t_1 = __pyx_PyFloat_AsDouble(__pyx_v_value); if (unlikely((__pyx_t_1 == (double)-1) && PyErr_Occurred())) {__pyx_filename = __pyx_f[0]; __pyx_lineno = 81; __pyx_clineno = __LINE__; goto __pyx_L1_error;}
  __pyx_v_self->G_Kss = __pyx_t_1;

  /* function exit code */
  __pyx_r = 0;
  goto __pyx_L0;
  __pyx_L1_error:;
  __Pyx_AddTraceback("model.Bondarenko.G_Kss.__set__", __pyx_clineno, __pyx_lineno, __pyx_filename);
  __pyx_r = -1;
  __pyx_L0:;
  __Pyx_RefNannyFinishContext();
  return __pyx_r;
}
```

```
 82:     #cdef public double G_Ktos       #(septum)
```

```
 83:     #cdef public double G_Kur        #(septum)
```

```
 84:     #cdef public double G_Kss        #(septum)
```

```
 85:     cdef public double G_Kr
```

```
/* Python wrapper */
static PyObject *__pyx_pw_5model_10Bondarenko_4G_Kr_1__get__(PyObject *__pyx_v_self); /*proto*/
static PyObject *__pyx_pw_5model_10Bondarenko_4G_Kr_1__get__(PyObject *__pyx_v_self) {
  PyObject *__pyx_r = 0;
  __Pyx_RefNannyDeclarations
  __Pyx_RefNannySetupContext("__get__ (wrapper)", 0);
  __pyx_r = __pyx_pf_5model_10Bondarenko_4G_Kr___get__(((struct __pyx_obj_5model_Bondarenko *)__pyx_v_self));

  /* function exit code */
  __Pyx_RefNannyFinishContext();
  return __pyx_r;
}

static PyObject *__pyx_pf_5model_10Bondarenko_4G_Kr___get__(struct __pyx_obj_5model_Bondarenko *__pyx_v_self) {
  PyObject *__pyx_r = NULL;
  __Pyx_RefNannyDeclarations
  __Pyx_RefNannySetupContext("__get__", 0);
  __Pyx_XDECREF(__pyx_r);
  __pyx_t_1 = PyFloat_FromDouble(__pyx_v_self->G_Kr); if (unlikely(!__pyx_t_1)) {__pyx_filename = __pyx_f[0]; __pyx_lineno = 85; __pyx_clineno = __LINE__; goto __pyx_L1_error;}
  __Pyx_GOTREF(__pyx_t_1);
  __pyx_r = __pyx_t_1;
  __pyx_t_1 = 0;
  goto __pyx_L0;

  /* function exit code */
  __pyx_L1_error:;
  __Pyx_XDECREF(__pyx_t_1);
  __Pyx_AddTraceback("model.Bondarenko.G_Kr.__get__", __pyx_clineno, __pyx_lineno, __pyx_filename);
  __pyx_r = NULL;
  __pyx_L0:;
  __Pyx_XGIVEREF(__pyx_r);
  __Pyx_RefNannyFinishContext();
  return __pyx_r;
}

/* Python wrapper */
static int __pyx_pw_5model_10Bondarenko_4G_Kr_3__set__(PyObject *__pyx_v_self, PyObject *__pyx_v_value); /*proto*/
static int __pyx_pw_5model_10Bondarenko_4G_Kr_3__set__(PyObject *__pyx_v_self, PyObject *__pyx_v_value) {
  int __pyx_r;
  __Pyx_RefNannyDeclarations
  __Pyx_RefNannySetupContext("__set__ (wrapper)", 0);
  __pyx_r = __pyx_pf_5model_10Bondarenko_4G_Kr_2__set__(((struct __pyx_obj_5model_Bondarenko *)__pyx_v_self), ((PyObject *)__pyx_v_value));

  /* function exit code */
  __Pyx_RefNannyFinishContext();
  return __pyx_r;
}

static int __pyx_pf_5model_10Bondarenko_4G_Kr_2__set__(struct __pyx_obj_5model_Bondarenko *__pyx_v_self, PyObject *__pyx_v_value) {
  int __pyx_r;
  __Pyx_RefNannyDeclarations
  __Pyx_RefNannySetupContext("__set__", 0);
  __pyx_t_1 = __pyx_PyFloat_AsDouble(__pyx_v_value); if (unlikely((__pyx_t_1 == (double)-1) && PyErr_Occurred())) {__pyx_filename = __pyx_f[0]; __pyx_lineno = 85; __pyx_clineno = __LINE__; goto __pyx_L1_error;}
  __pyx_v_self->G_Kr = __pyx_t_1;

  /* function exit code */
  __pyx_r = 0;
  goto __pyx_L0;
  __pyx_L1_error:;
  __Pyx_AddTraceback("model.Bondarenko.G_Kr.__set__", __pyx_clineno, __pyx_lineno, __pyx_filename);
  __pyx_r = -1;
  __pyx_L0:;
  __Pyx_RefNannyFinishContext();
  return __pyx_r;
}
```

```
 86:     cdef public double k_f
```

```
/* Python wrapper */
static PyObject *__pyx_pw_5model_10Bondarenko_3k_f_1__get__(PyObject *__pyx_v_self); /*proto*/
static PyObject *__pyx_pw_5model_10Bondarenko_3k_f_1__get__(PyObject *__pyx_v_self) {
  PyObject *__pyx_r = 0;
  __Pyx_RefNannyDeclarations
  __Pyx_RefNannySetupContext("__get__ (wrapper)", 0);
  __pyx_r = __pyx_pf_5model_10Bondarenko_3k_f___get__(((struct __pyx_obj_5model_Bondarenko *)__pyx_v_self));

  /* function exit code */
  __Pyx_RefNannyFinishContext();
  return __pyx_r;
}

static PyObject *__pyx_pf_5model_10Bondarenko_3k_f___get__(struct __pyx_obj_5model_Bondarenko *__pyx_v_self) {
  PyObject *__pyx_r = NULL;
  __Pyx_RefNannyDeclarations
  __Pyx_RefNannySetupContext("__get__", 0);
  __Pyx_XDECREF(__pyx_r);
  __pyx_t_1 = PyFloat_FromDouble(__pyx_v_self->k_f); if (unlikely(!__pyx_t_1)) {__pyx_filename = __pyx_f[0]; __pyx_lineno = 86; __pyx_clineno = __LINE__; goto __pyx_L1_error;}
  __Pyx_GOTREF(__pyx_t_1);
  __pyx_r = __pyx_t_1;
  __pyx_t_1 = 0;
  goto __pyx_L0;

  /* function exit code */
  __pyx_L1_error:;
  __Pyx_XDECREF(__pyx_t_1);
  __Pyx_AddTraceback("model.Bondarenko.k_f.__get__", __pyx_clineno, __pyx_lineno, __pyx_filename);
  __pyx_r = NULL;
  __pyx_L0:;
  __Pyx_XGIVEREF(__pyx_r);
  __Pyx_RefNannyFinishContext();
  return __pyx_r;
}

/* Python wrapper */
static int __pyx_pw_5model_10Bondarenko_3k_f_3__set__(PyObject *__pyx_v_self, PyObject *__pyx_v_value); /*proto*/
static int __pyx_pw_5model_10Bondarenko_3k_f_3__set__(PyObject *__pyx_v_self, PyObject *__pyx_v_value) {
  int __pyx_r;
  __Pyx_RefNannyDeclarations
  __Pyx_RefNannySetupContext("__set__ (wrapper)", 0);
  __pyx_r = __pyx_pf_5model_10Bondarenko_3k_f_2__set__(((struct __pyx_obj_5model_Bondarenko *)__pyx_v_self), ((PyObject *)__pyx_v_value));

  /* function exit code */
  __Pyx_RefNannyFinishContext();
  return __pyx_r;
}

static int __pyx_pf_5model_10Bondarenko_3k_f_2__set__(struct __pyx_obj_5model_Bondarenko *__pyx_v_self, PyObject *__pyx_v_value) {
  int __pyx_r;
  __Pyx_RefNannyDeclarations
  __Pyx_RefNannySetupContext("__set__", 0);
  __pyx_t_1 = __pyx_PyFloat_AsDouble(__pyx_v_value); if (unlikely((__pyx_t_1 == (double)-1) && PyErr_Occurred())) {__pyx_filename = __pyx_f[0]; __pyx_lineno = 86; __pyx_clineno = __LINE__; goto __pyx_L1_error;}
  __pyx_v_self->k_f = __pyx_t_1;

  /* function exit code */
  __pyx_r = 0;
  goto __pyx_L0;
  __pyx_L1_error:;
  __Pyx_AddTraceback("model.Bondarenko.k_f.__set__", __pyx_clineno, __pyx_lineno, __pyx_filename);
  __pyx_r = -1;
  __pyx_L0:;
  __Pyx_RefNannyFinishContext();
  return __pyx_r;
}
```

```
 87:     cdef public double k_b
```

```
/* Python wrapper */
static PyObject *__pyx_pw_5model_10Bondarenko_3k_b_1__get__(PyObject *__pyx_v_self); /*proto*/
static PyObject *__pyx_pw_5model_10Bondarenko_3k_b_1__get__(PyObject *__pyx_v_self) {
  PyObject *__pyx_r = 0;
  __Pyx_RefNannyDeclarations
  __Pyx_RefNannySetupContext("__get__ (wrapper)", 0);
  __pyx_r = __pyx_pf_5model_10Bondarenko_3k_b___get__(((struct __pyx_obj_5model_Bondarenko *)__pyx_v_self));

  /* function exit code */
  __Pyx_RefNannyFinishContext();
  return __pyx_r;
}

static PyObject *__pyx_pf_5model_10Bondarenko_3k_b___get__(struct __pyx_obj_5model_Bondarenko *__pyx_v_self) {
  PyObject *__pyx_r = NULL;
  __Pyx_RefNannyDeclarations
  __Pyx_RefNannySetupContext("__get__", 0);
  __Pyx_XDECREF(__pyx_r);
  __pyx_t_1 = PyFloat_FromDouble(__pyx_v_self->k_b); if (unlikely(!__pyx_t_1)) {__pyx_filename = __pyx_f[0]; __pyx_lineno = 87; __pyx_clineno = __LINE__; goto __pyx_L1_error;}
  __Pyx_GOTREF(__pyx_t_1);
  __pyx_r = __pyx_t_1;
  __pyx_t_1 = 0;
  goto __pyx_L0;

  /* function exit code */
  __pyx_L1_error:;
  __Pyx_XDECREF(__pyx_t_1);
  __Pyx_AddTraceback("model.Bondarenko.k_b.__get__", __pyx_clineno, __pyx_lineno, __pyx_filename);
  __pyx_r = NULL;
  __pyx_L0:;
  __Pyx_XGIVEREF(__pyx_r);
  __Pyx_RefNannyFinishContext();
  return __pyx_r;
}

/* Python wrapper */
static int __pyx_pw_5model_10Bondarenko_3k_b_3__set__(PyObject *__pyx_v_self, PyObject *__pyx_v_value); /*proto*/
static int __pyx_pw_5model_10Bondarenko_3k_b_3__set__(PyObject *__pyx_v_self, PyObject *__pyx_v_value) {
  int __pyx_r;
  __Pyx_RefNannyDeclarations
  __Pyx_RefNannySetupContext("__set__ (wrapper)", 0);
  __pyx_r = __pyx_pf_5model_10Bondarenko_3k_b_2__set__(((struct __pyx_obj_5model_Bondarenko *)__pyx_v_self), ((PyObject *)__pyx_v_value));

  /* function exit code */
  __Pyx_RefNannyFinishContext();
  return __pyx_r;
}

static int __pyx_pf_5model_10Bondarenko_3k_b_2__set__(struct __pyx_obj_5model_Bondarenko *__pyx_v_self, PyObject *__pyx_v_value) {
  int __pyx_r;
  __Pyx_RefNannyDeclarations
  __Pyx_RefNannySetupContext("__set__", 0);
  __pyx_t_1 = __pyx_PyFloat_AsDouble(__pyx_v_value); if (unlikely((__pyx_t_1 == (double)-1) && PyErr_Occurred())) {__pyx_filename = __pyx_f[0]; __pyx_lineno = 87; __pyx_clineno = __LINE__; goto __pyx_L1_error;}
  __pyx_v_self->k_b = __pyx_t_1;

  /* function exit code */
  __pyx_r = 0;
  goto __pyx_L0;
  __pyx_L1_error:;
  __Pyx_AddTraceback("model.Bondarenko.k_b.__set__", __pyx_clineno, __pyx_lineno, __pyx_filename);
  __pyx_r = -1;
  __pyx_L0:;
  __Pyx_RefNannyFinishContext();
  return __pyx_r;
}
```

```
 88:     cdef public double G_ClCa
```

```
/* Python wrapper */
static PyObject *__pyx_pw_5model_10Bondarenko_6G_ClCa_1__get__(PyObject *__pyx_v_self); /*proto*/
static PyObject *__pyx_pw_5model_10Bondarenko_6G_ClCa_1__get__(PyObject *__pyx_v_self) {
  PyObject *__pyx_r = 0;
  __Pyx_RefNannyDeclarations
  __Pyx_RefNannySetupContext("__get__ (wrapper)", 0);
  __pyx_r = __pyx_pf_5model_10Bondarenko_6G_ClCa___get__(((struct __pyx_obj_5model_Bondarenko *)__pyx_v_self));

  /* function exit code */
  __Pyx_RefNannyFinishContext();
  return __pyx_r;
}

static PyObject *__pyx_pf_5model_10Bondarenko_6G_ClCa___get__(struct __pyx_obj_5model_Bondarenko *__pyx_v_self) {
  PyObject *__pyx_r = NULL;
  __Pyx_RefNannyDeclarations
  __Pyx_RefNannySetupContext("__get__", 0);
  __Pyx_XDECREF(__pyx_r);
  __pyx_t_1 = PyFloat_FromDouble(__pyx_v_self->G_ClCa); if (unlikely(!__pyx_t_1)) {__pyx_filename = __pyx_f[0]; __pyx_lineno = 88; __pyx_clineno = __LINE__; goto __pyx_L1_error;}
  __Pyx_GOTREF(__pyx_t_1);
  __pyx_r = __pyx_t_1;
  __pyx_t_1 = 0;
  goto __pyx_L0;

  /* function exit code */
  __pyx_L1_error:;
  __Pyx_XDECREF(__pyx_t_1);
  __Pyx_AddTraceback("model.Bondarenko.G_ClCa.__get__", __pyx_clineno, __pyx_lineno, __pyx_filename);
  __pyx_r = NULL;
  __pyx_L0:;
  __Pyx_XGIVEREF(__pyx_r);
  __Pyx_RefNannyFinishContext();
  return __pyx_r;
}

/* Python wrapper */
static int __pyx_pw_5model_10Bondarenko_6G_ClCa_3__set__(PyObject *__pyx_v_self, PyObject *__pyx_v_value); /*proto*/
static int __pyx_pw_5model_10Bondarenko_6G_ClCa_3__set__(PyObject *__pyx_v_self, PyObject *__pyx_v_value) {
  int __pyx_r;
  __Pyx_RefNannyDeclarations
  __Pyx_RefNannySetupContext("__set__ (wrapper)", 0);
  __pyx_r = __pyx_pf_5model_10Bondarenko_6G_ClCa_2__set__(((struct __pyx_obj_5model_Bondarenko *)__pyx_v_self), ((PyObject *)__pyx_v_value));

  /* function exit code */
  __Pyx_RefNannyFinishContext();
  return __pyx_r;
}

static int __pyx_pf_5model_10Bondarenko_6G_ClCa_2__set__(struct __pyx_obj_5model_Bondarenko *__pyx_v_self, PyObject *__pyx_v_value) {
  int __pyx_r;
  __Pyx_RefNannyDeclarations
  __Pyx_RefNannySetupContext("__set__", 0);
  __pyx_t_1 = __pyx_PyFloat_AsDouble(__pyx_v_value); if (unlikely((__pyx_t_1 == (double)-1) && PyErr_Occurred())) {__pyx_filename = __pyx_f[0]; __pyx_lineno = 88; __pyx_clineno = __LINE__; goto __pyx_L1_error;}
  __pyx_v_self->G_ClCa = __pyx_t_1;

  /* function exit code */
  __pyx_r = 0;
  goto __pyx_L0;
  __pyx_L1_error:;
  __Pyx_AddTraceback("model.Bondarenko.G_ClCa.__set__", __pyx_clineno, __pyx_lineno, __pyx_filename);
  __pyx_r = -1;
  __pyx_L0:;
  __Pyx_RefNannyFinishContext();
  return __pyx_r;
}
```

```
 89:     cdef public double K_mCl
```

```
/* Python wrapper */
static PyObject *__pyx_pw_5model_10Bondarenko_5K_mCl_1__get__(PyObject *__pyx_v_self); /*proto*/
static PyObject *__pyx_pw_5model_10Bondarenko_5K_mCl_1__get__(PyObject *__pyx_v_self) {
  PyObject *__pyx_r = 0;
  __Pyx_RefNannyDeclarations
  __Pyx_RefNannySetupContext("__get__ (wrapper)", 0);
  __pyx_r = __pyx_pf_5model_10Bondarenko_5K_mCl___get__(((struct __pyx_obj_5model_Bondarenko *)__pyx_v_self));

  /* function exit code */
  __Pyx_RefNannyFinishContext();
  return __pyx_r;
}

static PyObject *__pyx_pf_5model_10Bondarenko_5K_mCl___get__(struct __pyx_obj_5model_Bondarenko *__pyx_v_self) {
  PyObject *__pyx_r = NULL;
  __Pyx_RefNannyDeclarations
  __Pyx_RefNannySetupContext("__get__", 0);
  __Pyx_XDECREF(__pyx_r);
  __pyx_t_1 = PyFloat_FromDouble(__pyx_v_self->K_mCl); if (unlikely(!__pyx_t_1)) {__pyx_filename = __pyx_f[0]; __pyx_lineno = 89; __pyx_clineno = __LINE__; goto __pyx_L1_error;}
  __Pyx_GOTREF(__pyx_t_1);
  __pyx_r = __pyx_t_1;
  __pyx_t_1 = 0;
  goto __pyx_L0;

  /* function exit code */
  __pyx_L1_error:;
  __Pyx_XDECREF(__pyx_t_1);
  __Pyx_AddTraceback("model.Bondarenko.K_mCl.__get__", __pyx_clineno, __pyx_lineno, __pyx_filename);
  __pyx_r = NULL;
  __pyx_L0:;
  __Pyx_XGIVEREF(__pyx_r);
  __Pyx_RefNannyFinishContext();
  return __pyx_r;
}

/* Python wrapper */
static int __pyx_pw_5model_10Bondarenko_5K_mCl_3__set__(PyObject *__pyx_v_self, PyObject *__pyx_v_value); /*proto*/
static int __pyx_pw_5model_10Bondarenko_5K_mCl_3__set__(PyObject *__pyx_v_self, PyObject *__pyx_v_value) {
  int __pyx_r;
  __Pyx_RefNannyDeclarations
  __Pyx_RefNannySetupContext("__set__ (wrapper)", 0);
  __pyx_r = __pyx_pf_5model_10Bondarenko_5K_mCl_2__set__(((struct __pyx_obj_5model_Bondarenko *)__pyx_v_self), ((PyObject *)__pyx_v_value));

  /* function exit code */
  __Pyx_RefNannyFinishContext();
  return __pyx_r;
}

static int __pyx_pf_5model_10Bondarenko_5K_mCl_2__set__(struct __pyx_obj_5model_Bondarenko *__pyx_v_self, PyObject *__pyx_v_value) {
  int __pyx_r;
  __Pyx_RefNannyDeclarations
  __Pyx_RefNannySetupContext("__set__", 0);
  __pyx_t_1 = __pyx_PyFloat_AsDouble(__pyx_v_value); if (unlikely((__pyx_t_1 == (double)-1) && PyErr_Occurred())) {__pyx_filename = __pyx_f[0]; __pyx_lineno = 89; __pyx_clineno = __LINE__; goto __pyx_L1_error;}
  __pyx_v_self->K_mCl = __pyx_t_1;

  /* function exit code */
  __pyx_r = 0;
  goto __pyx_L0;
  __pyx_L1_error:;
  __Pyx_AddTraceback("model.Bondarenko.K_mCl.__set__", __pyx_clineno, __pyx_lineno, __pyx_filename);
  __pyx_r = -1;
  __pyx_L0:;
  __Pyx_RefNannyFinishContext();
  return __pyx_r;
}
```

```
 90:     cdef public double E_Cl
```

```
/* Python wrapper */
static PyObject *__pyx_pw_5model_10Bondarenko_4E_Cl_1__get__(PyObject *__pyx_v_self); /*proto*/
static PyObject *__pyx_pw_5model_10Bondarenko_4E_Cl_1__get__(PyObject *__pyx_v_self) {
  PyObject *__pyx_r = 0;
  __Pyx_RefNannyDeclarations
  __Pyx_RefNannySetupContext("__get__ (wrapper)", 0);
  __pyx_r = __pyx_pf_5model_10Bondarenko_4E_Cl___get__(((struct __pyx_obj_5model_Bondarenko *)__pyx_v_self));

  /* function exit code */
  __Pyx_RefNannyFinishContext();
  return __pyx_r;
}

static PyObject *__pyx_pf_5model_10Bondarenko_4E_Cl___get__(struct __pyx_obj_5model_Bondarenko *__pyx_v_self) {
  PyObject *__pyx_r = NULL;
  __Pyx_RefNannyDeclarations
  __Pyx_RefNannySetupContext("__get__", 0);
  __Pyx_XDECREF(__pyx_r);
  __pyx_t_1 = PyFloat_FromDouble(__pyx_v_self->E_Cl); if (unlikely(!__pyx_t_1)) {__pyx_filename = __pyx_f[0]; __pyx_lineno = 90; __pyx_clineno = __LINE__; goto __pyx_L1_error;}
  __Pyx_GOTREF(__pyx_t_1);
  __pyx_r = __pyx_t_1;
  __pyx_t_1 = 0;
  goto __pyx_L0;

  /* function exit code */
  __pyx_L1_error:;
  __Pyx_XDECREF(__pyx_t_1);
  __Pyx_AddTraceback("model.Bondarenko.E_Cl.__get__", __pyx_clineno, __pyx_lineno, __pyx_filename);
  __pyx_r = NULL;
  __pyx_L0:;
  __Pyx_XGIVEREF(__pyx_r);
  __Pyx_RefNannyFinishContext();
  return __pyx_r;
}

/* Python wrapper */
static int __pyx_pw_5model_10Bondarenko_4E_Cl_3__set__(PyObject *__pyx_v_self, PyObject *__pyx_v_value); /*proto*/
static int __pyx_pw_5model_10Bondarenko_4E_Cl_3__set__(PyObject *__pyx_v_self, PyObject *__pyx_v_value) {
  int __pyx_r;
  __Pyx_RefNannyDeclarations
  __Pyx_RefNannySetupContext("__set__ (wrapper)", 0);
  __pyx_r = __pyx_pf_5model_10Bondarenko_4E_Cl_2__set__(((struct __pyx_obj_5model_Bondarenko *)__pyx_v_self), ((PyObject *)__pyx_v_value));

  /* function exit code */
  __Pyx_RefNannyFinishContext();
  return __pyx_r;
}

static int __pyx_pf_5model_10Bondarenko_4E_Cl_2__set__(struct __pyx_obj_5model_Bondarenko *__pyx_v_self, PyObject *__pyx_v_value) {
  int __pyx_r;
  __Pyx_RefNannyDeclarations
  __Pyx_RefNannySetupContext("__set__", 0);
  __pyx_t_1 = __pyx_PyFloat_AsDouble(__pyx_v_value); if (unlikely((__pyx_t_1 == (double)-1) && PyErr_Occurred())) {__pyx_filename = __pyx_f[0]; __pyx_lineno = 90; __pyx_clineno = __LINE__; goto __pyx_L1_error;}
  __pyx_v_self->E_Cl = __pyx_t_1;

  /* function exit code */
  __pyx_r = 0;
  goto __pyx_L0;
  __pyx_L1_error:;
  __Pyx_AddTraceback("model.Bondarenko.E_Cl.__set__", __pyx_clineno, __pyx_lineno, __pyx_filename);
  __pyx_r = -1;
  __pyx_L0:;
  __Pyx_RefNannyFinishContext();
  return __pyx_r;
}
```

```
 91:     cdef public double Laplas_c
```

```
/* Python wrapper */
static PyObject *__pyx_pw_5model_10Bondarenko_8Laplas_c_1__get__(PyObject *__pyx_v_self); /*proto*/
static PyObject *__pyx_pw_5model_10Bondarenko_8Laplas_c_1__get__(PyObject *__pyx_v_self) {
  PyObject *__pyx_r = 0;
  __Pyx_RefNannyDeclarations
  __Pyx_RefNannySetupContext("__get__ (wrapper)", 0);
  __pyx_r = __pyx_pf_5model_10Bondarenko_8Laplas_c___get__(((struct __pyx_obj_5model_Bondarenko *)__pyx_v_self));

  /* function exit code */
  __Pyx_RefNannyFinishContext();
  return __pyx_r;
}

static PyObject *__pyx_pf_5model_10Bondarenko_8Laplas_c___get__(struct __pyx_obj_5model_Bondarenko *__pyx_v_self) {
  PyObject *__pyx_r = NULL;
  __Pyx_RefNannyDeclarations
  __Pyx_RefNannySetupContext("__get__", 0);
  __Pyx_XDECREF(__pyx_r);
  __pyx_t_1 = PyFloat_FromDouble(__pyx_v_self->Laplas_c); if (unlikely(!__pyx_t_1)) {__pyx_filename = __pyx_f[0]; __pyx_lineno = 91; __pyx_clineno = __LINE__; goto __pyx_L1_error;}
  __Pyx_GOTREF(__pyx_t_1);
  __pyx_r = __pyx_t_1;
  __pyx_t_1 = 0;
  goto __pyx_L0;

  /* function exit code */
  __pyx_L1_error:;
  __Pyx_XDECREF(__pyx_t_1);
  __Pyx_AddTraceback("model.Bondarenko.Laplas_c.__get__", __pyx_clineno, __pyx_lineno, __pyx_filename);
  __pyx_r = NULL;
  __pyx_L0:;
  __Pyx_XGIVEREF(__pyx_r);
  __Pyx_RefNannyFinishContext();
  return __pyx_r;
}

/* Python wrapper */
static int __pyx_pw_5model_10Bondarenko_8Laplas_c_3__set__(PyObject *__pyx_v_self, PyObject *__pyx_v_value); /*proto*/
static int __pyx_pw_5model_10Bondarenko_8Laplas_c_3__set__(PyObject *__pyx_v_self, PyObject *__pyx_v_value) {
  int __pyx_r;
  __Pyx_RefNannyDeclarations
  __Pyx_RefNannySetupContext("__set__ (wrapper)", 0);
  __pyx_r = __pyx_pf_5model_10Bondarenko_8Laplas_c_2__set__(((struct __pyx_obj_5model_Bondarenko *)__pyx_v_self), ((PyObject *)__pyx_v_value));

  /* function exit code */
  __Pyx_RefNannyFinishContext();
  return __pyx_r;
}

static int __pyx_pf_5model_10Bondarenko_8Laplas_c_2__set__(struct __pyx_obj_5model_Bondarenko *__pyx_v_self, PyObject *__pyx_v_value) {
  int __pyx_r;
  __Pyx_RefNannyDeclarations
  __Pyx_RefNannySetupContext("__set__", 0);
  __pyx_t_1 = __pyx_PyFloat_AsDouble(__pyx_v_value); if (unlikely((__pyx_t_1 == (double)-1) && PyErr_Occurred())) {__pyx_filename = __pyx_f[0]; __pyx_lineno = 91; __pyx_clineno = __LINE__; goto __pyx_L1_error;}
  __pyx_v_self->Laplas_c = __pyx_t_1;

  /* function exit code */
  __pyx_r = 0;
  goto __pyx_L0;
  __pyx_L1_error:;
  __Pyx_AddTraceback("model.Bondarenko.Laplas_c.__set__", __pyx_clineno, __pyx_lineno, __pyx_filename);
  __pyx_r = -1;
  __pyx_L0:;
  __Pyx_RefNannyFinishContext();
  return __pyx_r;
}
```

```
 92:     cdef public double Laplas_r
```

```
/* Python wrapper */
static PyObject *__pyx_pw_5model_10Bondarenko_8Laplas_r_1__get__(PyObject *__pyx_v_self); /*proto*/
static PyObject *__pyx_pw_5model_10Bondarenko_8Laplas_r_1__get__(PyObject *__pyx_v_self) {
  PyObject *__pyx_r = 0;
  __Pyx_RefNannyDeclarations
  __Pyx_RefNannySetupContext("__get__ (wrapper)", 0);
  __pyx_r = __pyx_pf_5model_10Bondarenko_8Laplas_r___get__(((struct __pyx_obj_5model_Bondarenko *)__pyx_v_self));

  /* function exit code */
  __Pyx_RefNannyFinishContext();
  return __pyx_r;
}

static PyObject *__pyx_pf_5model_10Bondarenko_8Laplas_r___get__(struct __pyx_obj_5model_Bondarenko *__pyx_v_self) {
  PyObject *__pyx_r = NULL;
  __Pyx_RefNannyDeclarations
  __Pyx_RefNannySetupContext("__get__", 0);
  __Pyx_XDECREF(__pyx_r);
  __pyx_t_1 = PyFloat_FromDouble(__pyx_v_self->Laplas_r); if (unlikely(!__pyx_t_1)) {__pyx_filename = __pyx_f[0]; __pyx_lineno = 92; __pyx_clineno = __LINE__; goto __pyx_L1_error;}
  __Pyx_GOTREF(__pyx_t_1);
  __pyx_r = __pyx_t_1;
  __pyx_t_1 = 0;
  goto __pyx_L0;

  /* function exit code */
  __pyx_L1_error:;
  __Pyx_XDECREF(__pyx_t_1);
  __Pyx_AddTraceback("model.Bondarenko.Laplas_r.__get__", __pyx_clineno, __pyx_lineno, __pyx_filename);
  __pyx_r = NULL;
  __pyx_L0:;
  __Pyx_XGIVEREF(__pyx_r);
  __Pyx_RefNannyFinishContext();
  return __pyx_r;
}

/* Python wrapper */
static int __pyx_pw_5model_10Bondarenko_8Laplas_r_3__set__(PyObject *__pyx_v_self, PyObject *__pyx_v_value); /*proto*/
static int __pyx_pw_5model_10Bondarenko_8Laplas_r_3__set__(PyObject *__pyx_v_self, PyObject *__pyx_v_value) {
  int __pyx_r;
  __Pyx_RefNannyDeclarations
  __Pyx_RefNannySetupContext("__set__ (wrapper)", 0);
  __pyx_r = __pyx_pf_5model_10Bondarenko_8Laplas_r_2__set__(((struct __pyx_obj_5model_Bondarenko *)__pyx_v_self), ((PyObject *)__pyx_v_value));

  /* function exit code */
  __Pyx_RefNannyFinishContext();
  return __pyx_r;
}

static int __pyx_pf_5model_10Bondarenko_8Laplas_r_2__set__(struct __pyx_obj_5model_Bondarenko *__pyx_v_self, PyObject *__pyx_v_value) {
  int __pyx_r;
  __Pyx_RefNannyDeclarations
  __Pyx_RefNannySetupContext("__set__", 0);
  __pyx_t_1 = __pyx_PyFloat_AsDouble(__pyx_v_value); if (unlikely((__pyx_t_1 == (double)-1) && PyErr_Occurred())) {__pyx_filename = __pyx_f[0]; __pyx_lineno = 92; __pyx_clineno = __LINE__; goto __pyx_L1_error;}
  __pyx_v_self->Laplas_r = __pyx_t_1;

  /* function exit code */
  __pyx_r = 0;
  goto __pyx_L0;
  __pyx_L1_error:;
  __Pyx_AddTraceback("model.Bondarenko.Laplas_r.__set__", __pyx_clineno, __pyx_lineno, __pyx_filename);
  __pyx_r = -1;
  __pyx_L0:;
  __Pyx_RefNannyFinishContext();
  return __pyx_r;
}
```

```
 93:     cdef public double V_ChR2
```

```
/* Python wrapper */
static PyObject *__pyx_pw_5model_10Bondarenko_6V_ChR2_1__get__(PyObject *__pyx_v_self); /*proto*/
static PyObject *__pyx_pw_5model_10Bondarenko_6V_ChR2_1__get__(PyObject *__pyx_v_self) {
  PyObject *__pyx_r = 0;
  __Pyx_RefNannyDeclarations
  __Pyx_RefNannySetupContext("__get__ (wrapper)", 0);
  __pyx_r = __pyx_pf_5model_10Bondarenko_6V_ChR2___get__(((struct __pyx_obj_5model_Bondarenko *)__pyx_v_self));

  /* function exit code */
  __Pyx_RefNannyFinishContext();
  return __pyx_r;
}

static PyObject *__pyx_pf_5model_10Bondarenko_6V_ChR2___get__(struct __pyx_obj_5model_Bondarenko *__pyx_v_self) {
  PyObject *__pyx_r = NULL;
  __Pyx_RefNannyDeclarations
  __Pyx_RefNannySetupContext("__get__", 0);
  __Pyx_XDECREF(__pyx_r);
  __pyx_t_1 = PyFloat_FromDouble(__pyx_v_self->V_ChR2); if (unlikely(!__pyx_t_1)) {__pyx_filename = __pyx_f[0]; __pyx_lineno = 93; __pyx_clineno = __LINE__; goto __pyx_L1_error;}
  __Pyx_GOTREF(__pyx_t_1);
  __pyx_r = __pyx_t_1;
  __pyx_t_1 = 0;
  goto __pyx_L0;

  /* function exit code */
  __pyx_L1_error:;
  __Pyx_XDECREF(__pyx_t_1);
  __Pyx_AddTraceback("model.Bondarenko.V_ChR2.__get__", __pyx_clineno, __pyx_lineno, __pyx_filename);
  __pyx_r = NULL;
  __pyx_L0:;
  __Pyx_XGIVEREF(__pyx_r);
  __Pyx_RefNannyFinishContext();
  return __pyx_r;
}

/* Python wrapper */
static int __pyx_pw_5model_10Bondarenko_6V_ChR2_3__set__(PyObject *__pyx_v_self, PyObject *__pyx_v_value); /*proto*/
static int __pyx_pw_5model_10Bondarenko_6V_ChR2_3__set__(PyObject *__pyx_v_self, PyObject *__pyx_v_value) {
  int __pyx_r;
  __Pyx_RefNannyDeclarations
  __Pyx_RefNannySetupContext("__set__ (wrapper)", 0);
  __pyx_r = __pyx_pf_5model_10Bondarenko_6V_ChR2_2__set__(((struct __pyx_obj_5model_Bondarenko *)__pyx_v_self), ((PyObject *)__pyx_v_value));

  /* function exit code */
  __Pyx_RefNannyFinishContext();
  return __pyx_r;
}

static int __pyx_pf_5model_10Bondarenko_6V_ChR2_2__set__(struct __pyx_obj_5model_Bondarenko *__pyx_v_self, PyObject *__pyx_v_value) {
  int __pyx_r;
  __Pyx_RefNannyDeclarations
  __Pyx_RefNannySetupContext("__set__", 0);
  __pyx_t_1 = __pyx_PyFloat_AsDouble(__pyx_v_value); if (unlikely((__pyx_t_1 == (double)-1) && PyErr_Occurred())) {__pyx_filename = __pyx_f[0]; __pyx_lineno = 93; __pyx_clineno = __LINE__; goto __pyx_L1_error;}
  __pyx_v_self->V_ChR2 = __pyx_t_1;

  /* function exit code */
  __pyx_r = 0;
  goto __pyx_L0;
  __pyx_L1_error:;
  __Pyx_AddTraceback("model.Bondarenko.V_ChR2.__set__", __pyx_clineno, __pyx_lineno, __pyx_filename);
  __pyx_r = -1;
  __pyx_L0:;
  __Pyx_RefNannyFinishContext();
  return __pyx_r;
}
```

```
 94:     cdef public double i_Kss
```

```
/* Python wrapper */
static PyObject *__pyx_pw_5model_10Bondarenko_5i_Kss_1__get__(PyObject *__pyx_v_self); /*proto*/
static PyObject *__pyx_pw_5model_10Bondarenko_5i_Kss_1__get__(PyObject *__pyx_v_self) {
  PyObject *__pyx_r = 0;
  __Pyx_RefNannyDeclarations
  __Pyx_RefNannySetupContext("__get__ (wrapper)", 0);
  __pyx_r = __pyx_pf_5model_10Bondarenko_5i_Kss___get__(((struct __pyx_obj_5model_Bondarenko *)__pyx_v_self));

  /* function exit code */
  __Pyx_RefNannyFinishContext();
  return __pyx_r;
}

static PyObject *__pyx_pf_5model_10Bondarenko_5i_Kss___get__(struct __pyx_obj_5model_Bondarenko *__pyx_v_self) {
  PyObject *__pyx_r = NULL;
  __Pyx_RefNannyDeclarations
  __Pyx_RefNannySetupContext("__get__", 0);
  __Pyx_XDECREF(__pyx_r);
  __pyx_t_1 = PyFloat_FromDouble(__pyx_v_self->i_Kss); if (unlikely(!__pyx_t_1)) {__pyx_filename = __pyx_f[0]; __pyx_lineno = 94; __pyx_clineno = __LINE__; goto __pyx_L1_error;}
  __Pyx_GOTREF(__pyx_t_1);
  __pyx_r = __pyx_t_1;
  __pyx_t_1 = 0;
  goto __pyx_L0;

  /* function exit code */
  __pyx_L1_error:;
  __Pyx_XDECREF(__pyx_t_1);
  __Pyx_AddTraceback("model.Bondarenko.i_Kss.__get__", __pyx_clineno, __pyx_lineno, __pyx_filename);
  __pyx_r = NULL;
  __pyx_L0:;
  __Pyx_XGIVEREF(__pyx_r);
  __Pyx_RefNannyFinishContext();
  return __pyx_r;
}

/* Python wrapper */
static int __pyx_pw_5model_10Bondarenko_5i_Kss_3__set__(PyObject *__pyx_v_self, PyObject *__pyx_v_value); /*proto*/
static int __pyx_pw_5model_10Bondarenko_5i_Kss_3__set__(PyObject *__pyx_v_self, PyObject *__pyx_v_value) {
  int __pyx_r;
  __Pyx_RefNannyDeclarations
  __Pyx_RefNannySetupContext("__set__ (wrapper)", 0);
  __pyx_r = __pyx_pf_5model_10Bondarenko_5i_Kss_2__set__(((struct __pyx_obj_5model_Bondarenko *)__pyx_v_self), ((PyObject *)__pyx_v_value));

  /* function exit code */
  __Pyx_RefNannyFinishContext();
  return __pyx_r;
}

static int __pyx_pf_5model_10Bondarenko_5i_Kss_2__set__(struct __pyx_obj_5model_Bondarenko *__pyx_v_self, PyObject *__pyx_v_value) {
  int __pyx_r;
  __Pyx_RefNannyDeclarations
  __Pyx_RefNannySetupContext("__set__", 0);
  __pyx_t_1 = __pyx_PyFloat_AsDouble(__pyx_v_value); if (unlikely((__pyx_t_1 == (double)-1) && PyErr_Occurred())) {__pyx_filename = __pyx_f[0]; __pyx_lineno = 94; __pyx_clineno = __LINE__; goto __pyx_L1_error;}
  __pyx_v_self->i_Kss = __pyx_t_1;

  /* function exit code */
  __pyx_r = 0;
  goto __pyx_L0;
  __pyx_L1_error:;
  __Pyx_AddTraceback("model.Bondarenko.i_Kss.__set__", __pyx_clineno, __pyx_lineno, __pyx_filename);
  __pyx_r = -1;
  __pyx_L0:;
  __Pyx_RefNannyFinishContext();
  return __pyx_r;
}
```

```
 95:     cdef public double h
```

```
/* Python wrapper */
static PyObject *__pyx_pw_5model_10Bondarenko_1h_1__get__(PyObject *__pyx_v_self); /*proto*/
static PyObject *__pyx_pw_5model_10Bondarenko_1h_1__get__(PyObject *__pyx_v_self) {
  PyObject *__pyx_r = 0;
  __Pyx_RefNannyDeclarations
  __Pyx_RefNannySetupContext("__get__ (wrapper)", 0);
  __pyx_r = __pyx_pf_5model_10Bondarenko_1h___get__(((struct __pyx_obj_5model_Bondarenko *)__pyx_v_self));

  /* function exit code */
  __Pyx_RefNannyFinishContext();
  return __pyx_r;
}

static PyObject *__pyx_pf_5model_10Bondarenko_1h___get__(struct __pyx_obj_5model_Bondarenko *__pyx_v_self) {
  PyObject *__pyx_r = NULL;
  __Pyx_RefNannyDeclarations
  __Pyx_RefNannySetupContext("__get__", 0);
  __Pyx_XDECREF(__pyx_r);
  __pyx_t_1 = PyFloat_FromDouble(__pyx_v_self->h); if (unlikely(!__pyx_t_1)) {__pyx_filename = __pyx_f[0]; __pyx_lineno = 95; __pyx_clineno = __LINE__; goto __pyx_L1_error;}
  __Pyx_GOTREF(__pyx_t_1);
  __pyx_r = __pyx_t_1;
  __pyx_t_1 = 0;
  goto __pyx_L0;

  /* function exit code */
  __pyx_L1_error:;
  __Pyx_XDECREF(__pyx_t_1);
  __Pyx_AddTraceback("model.Bondarenko.h.__get__", __pyx_clineno, __pyx_lineno, __pyx_filename);
  __pyx_r = NULL;
  __pyx_L0:;
  __Pyx_XGIVEREF(__pyx_r);
  __Pyx_RefNannyFinishContext();
  return __pyx_r;
}

/* Python wrapper */
static int __pyx_pw_5model_10Bondarenko_1h_3__set__(PyObject *__pyx_v_self, PyObject *__pyx_v_value); /*proto*/
static int __pyx_pw_5model_10Bondarenko_1h_3__set__(PyObject *__pyx_v_self, PyObject *__pyx_v_value) {
  int __pyx_r;
  __Pyx_RefNannyDeclarations
  __Pyx_RefNannySetupContext("__set__ (wrapper)", 0);
  __pyx_r = __pyx_pf_5model_10Bondarenko_1h_2__set__(((struct __pyx_obj_5model_Bondarenko *)__pyx_v_self), ((PyObject *)__pyx_v_value));

  /* function exit code */
  __Pyx_RefNannyFinishContext();
  return __pyx_r;
}

static int __pyx_pf_5model_10Bondarenko_1h_2__set__(struct __pyx_obj_5model_Bondarenko *__pyx_v_self, PyObject *__pyx_v_value) {
  int __pyx_r;
  __Pyx_RefNannyDeclarations
  __Pyx_RefNannySetupContext("__set__", 0);
  __pyx_t_1 = __pyx_PyFloat_AsDouble(__pyx_v_value); if (unlikely((__pyx_t_1 == (double)-1) && PyErr_Occurred())) {__pyx_filename = __pyx_f[0]; __pyx_lineno = 95; __pyx_clineno = __LINE__; goto __pyx_L1_error;}
  __pyx_v_self->h = __pyx_t_1;

  /* function exit code */
  __pyx_r = 0;
  goto __pyx_L0;
  __pyx_L1_error:;
  __Pyx_AddTraceback("model.Bondarenko.h.__set__", __pyx_clineno, __pyx_lineno, __pyx_filename);
  __pyx_r = -1;
  __pyx_L0:;
  __Pyx_RefNannyFinishContext();
  return __pyx_r;
}
```

```
 96:     cdef public double dy, dx
```

```
/* Python wrapper */
static PyObject *__pyx_pw_5model_10Bondarenko_2dy_1__get__(PyObject *__pyx_v_self); /*proto*/
static PyObject *__pyx_pw_5model_10Bondarenko_2dy_1__get__(PyObject *__pyx_v_self) {
  PyObject *__pyx_r = 0;
  __Pyx_RefNannyDeclarations
  __Pyx_RefNannySetupContext("__get__ (wrapper)", 0);
  __pyx_r = __pyx_pf_5model_10Bondarenko_2dy___get__(((struct __pyx_obj_5model_Bondarenko *)__pyx_v_self));

  /* function exit code */
  __Pyx_RefNannyFinishContext();
  return __pyx_r;
}

static PyObject *__pyx_pf_5model_10Bondarenko_2dy___get__(struct __pyx_obj_5model_Bondarenko *__pyx_v_self) {
  PyObject *__pyx_r = NULL;
  __Pyx_RefNannyDeclarations
  __Pyx_RefNannySetupContext("__get__", 0);
  __Pyx_XDECREF(__pyx_r);
  __pyx_t_1 = PyFloat_FromDouble(__pyx_v_self->dy); if (unlikely(!__pyx_t_1)) {__pyx_filename = __pyx_f[0]; __pyx_lineno = 96; __pyx_clineno = __LINE__; goto __pyx_L1_error;}
  __Pyx_GOTREF(__pyx_t_1);
  __pyx_r = __pyx_t_1;
  __pyx_t_1 = 0;
  goto __pyx_L0;

  /* function exit code */
  __pyx_L1_error:;
  __Pyx_XDECREF(__pyx_t_1);
  __Pyx_AddTraceback("model.Bondarenko.dy.__get__", __pyx_clineno, __pyx_lineno, __pyx_filename);
  __pyx_r = NULL;
  __pyx_L0:;
  __Pyx_XGIVEREF(__pyx_r);
  __Pyx_RefNannyFinishContext();
  return __pyx_r;
}

/* Python wrapper */
static int __pyx_pw_5model_10Bondarenko_2dy_3__set__(PyObject *__pyx_v_self, PyObject *__pyx_v_value); /*proto*/
static int __pyx_pw_5model_10Bondarenko_2dy_3__set__(PyObject *__pyx_v_self, PyObject *__pyx_v_value) {
  int __pyx_r;
  __Pyx_RefNannyDeclarations
  __Pyx_RefNannySetupContext("__set__ (wrapper)", 0);
  __pyx_r = __pyx_pf_5model_10Bondarenko_2dy_2__set__(((struct __pyx_obj_5model_Bondarenko *)__pyx_v_self), ((PyObject *)__pyx_v_value));

  /* function exit code */
  __Pyx_RefNannyFinishContext();
  return __pyx_r;
}

static int __pyx_pf_5model_10Bondarenko_2dy_2__set__(struct __pyx_obj_5model_Bondarenko *__pyx_v_self, PyObject *__pyx_v_value) {
  int __pyx_r;
  __Pyx_RefNannyDeclarations
  __Pyx_RefNannySetupContext("__set__", 0);
  __pyx_t_1 = __pyx_PyFloat_AsDouble(__pyx_v_value); if (unlikely((__pyx_t_1 == (double)-1) && PyErr_Occurred())) {__pyx_filename = __pyx_f[0]; __pyx_lineno = 96; __pyx_clineno = __LINE__; goto __pyx_L1_error;}
  __pyx_v_self->dy = __pyx_t_1;

  /* function exit code */
  __pyx_r = 0;
  goto __pyx_L0;
  __pyx_L1_error:;
  __Pyx_AddTraceback("model.Bondarenko.dy.__set__", __pyx_clineno, __pyx_lineno, __pyx_filename);
  __pyx_r = -1;
  __pyx_L0:;
  __Pyx_RefNannyFinishContext();
  return __pyx_r;
}

/* Python wrapper */
static PyObject *__pyx_pw_5model_10Bondarenko_2dx_1__get__(PyObject *__pyx_v_self); /*proto*/
static PyObject *__pyx_pw_5model_10Bondarenko_2dx_1__get__(PyObject *__pyx_v_self) {
  PyObject *__pyx_r = 0;
  __Pyx_RefNannyDeclarations
  __Pyx_RefNannySetupContext("__get__ (wrapper)", 0);
  __pyx_r = __pyx_pf_5model_10Bondarenko_2dx___get__(((struct __pyx_obj_5model_Bondarenko *)__pyx_v_self));

  /* function exit code */
  __Pyx_RefNannyFinishContext();
  return __pyx_r;
}

static PyObject *__pyx_pf_5model_10Bondarenko_2dx___get__(struct __pyx_obj_5model_Bondarenko *__pyx_v_self) {
  PyObject *__pyx_r = NULL;
  __Pyx_RefNannyDeclarations
  __Pyx_RefNannySetupContext("__get__", 0);
  __Pyx_XDECREF(__pyx_r);
  __pyx_t_1 = PyFloat_FromDouble(__pyx_v_self->dx); if (unlikely(!__pyx_t_1)) {__pyx_filename = __pyx_f[0]; __pyx_lineno = 96; __pyx_clineno = __LINE__; goto __pyx_L1_error;}
  __Pyx_GOTREF(__pyx_t_1);
  __pyx_r = __pyx_t_1;
  __pyx_t_1 = 0;
  goto __pyx_L0;

  /* function exit code */
  __pyx_L1_error:;
  __Pyx_XDECREF(__pyx_t_1);
  __Pyx_AddTraceback("model.Bondarenko.dx.__get__", __pyx_clineno, __pyx_lineno, __pyx_filename);
  __pyx_r = NULL;
  __pyx_L0:;
  __Pyx_XGIVEREF(__pyx_r);
  __Pyx_RefNannyFinishContext();
  return __pyx_r;
}

/* Python wrapper */
static int __pyx_pw_5model_10Bondarenko_2dx_3__set__(PyObject *__pyx_v_self, PyObject *__pyx_v_value); /*proto*/
static int __pyx_pw_5model_10Bondarenko_2dx_3__set__(PyObject *__pyx_v_self, PyObject *__pyx_v_value) {
  int __pyx_r;
  __Pyx_RefNannyDeclarations
  __Pyx_RefNannySetupContext("__set__ (wrapper)", 0);
  __pyx_r = __pyx_pf_5model_10Bondarenko_2dx_2__set__(((struct __pyx_obj_5model_Bondarenko *)__pyx_v_self), ((PyObject *)__pyx_v_value));

  /* function exit code */
  __Pyx_RefNannyFinishContext();
  return __pyx_r;
}

static int __pyx_pf_5model_10Bondarenko_2dx_2__set__(struct __pyx_obj_5model_Bondarenko *__pyx_v_self, PyObject *__pyx_v_value) {
  int __pyx_r;
  __Pyx_RefNannyDeclarations
  __Pyx_RefNannySetupContext("__set__", 0);
  __pyx_t_1 = __pyx_PyFloat_AsDouble(__pyx_v_value); if (unlikely((__pyx_t_1 == (double)-1) && PyErr_Occurred())) {__pyx_filename = __pyx_f[0]; __pyx_lineno = 96; __pyx_clineno = __LINE__; goto __pyx_L1_error;}
  __pyx_v_self->dx = __pyx_t_1;

  /* function exit code */
  __pyx_r = 0;
  goto __pyx_L0;
  __pyx_L1_error:;
  __Pyx_AddTraceback("model.Bondarenko.dx.__set__", __pyx_clineno, __pyx_lineno, __pyx_filename);
  __pyx_r = -1;
  __pyx_L0:;
  __Pyx_RefNannyFinishContext();
  return __pyx_r;
}
```

```
 97:
```

```
 98:
```

```
 99:     cdef public double t
```

```
/* Python wrapper */
static PyObject *__pyx_pw_5model_10Bondarenko_1t_1__get__(PyObject *__pyx_v_self); /*proto*/
static PyObject *__pyx_pw_5model_10Bondarenko_1t_1__get__(PyObject *__pyx_v_self) {
  PyObject *__pyx_r = 0;
  __Pyx_RefNannyDeclarations
  __Pyx_RefNannySetupContext("__get__ (wrapper)", 0);
  __pyx_r = __pyx_pf_5model_10Bondarenko_1t___get__(((struct __pyx_obj_5model_Bondarenko *)__pyx_v_self));

  /* function exit code */
  __Pyx_RefNannyFinishContext();
  return __pyx_r;
}

static PyObject *__pyx_pf_5model_10Bondarenko_1t___get__(struct __pyx_obj_5model_Bondarenko *__pyx_v_self) {
  PyObject *__pyx_r = NULL;
  __Pyx_RefNannyDeclarations
  __Pyx_RefNannySetupContext("__get__", 0);
  __Pyx_XDECREF(__pyx_r);
  __pyx_t_1 = PyFloat_FromDouble(__pyx_v_self->t); if (unlikely(!__pyx_t_1)) {__pyx_filename = __pyx_f[0]; __pyx_lineno = 99; __pyx_clineno = __LINE__; goto __pyx_L1_error;}
  __Pyx_GOTREF(__pyx_t_1);
  __pyx_r = __pyx_t_1;
  __pyx_t_1 = 0;
  goto __pyx_L0;

  /* function exit code */
  __pyx_L1_error:;
  __Pyx_XDECREF(__pyx_t_1);
  __Pyx_AddTraceback("model.Bondarenko.t.__get__", __pyx_clineno, __pyx_lineno, __pyx_filename);
  __pyx_r = NULL;
  __pyx_L0:;
  __Pyx_XGIVEREF(__pyx_r);
  __Pyx_RefNannyFinishContext();
  return __pyx_r;
}

/* Python wrapper */
static int __pyx_pw_5model_10Bondarenko_1t_3__set__(PyObject *__pyx_v_self, PyObject *__pyx_v_value); /*proto*/
static int __pyx_pw_5model_10Bondarenko_1t_3__set__(PyObject *__pyx_v_self, PyObject *__pyx_v_value) {
  int __pyx_r;
  __Pyx_RefNannyDeclarations
  __Pyx_RefNannySetupContext("__set__ (wrapper)", 0);
  __pyx_r = __pyx_pf_5model_10Bondarenko_1t_2__set__(((struct __pyx_obj_5model_Bondarenko *)__pyx_v_self), ((PyObject *)__pyx_v_value));

  /* function exit code */
  __Pyx_RefNannyFinishContext();
  return __pyx_r;
}

static int __pyx_pf_5model_10Bondarenko_1t_2__set__(struct __pyx_obj_5model_Bondarenko *__pyx_v_self, PyObject *__pyx_v_value) {
  int __pyx_r;
  __Pyx_RefNannyDeclarations
  __Pyx_RefNannySetupContext("__set__", 0);
  __pyx_t_1 = __pyx_PyFloat_AsDouble(__pyx_v_value); if (unlikely((__pyx_t_1 == (double)-1) && PyErr_Occurred())) {__pyx_filename = __pyx_f[0]; __pyx_lineno = 99; __pyx_clineno = __LINE__; goto __pyx_L1_error;}
  __pyx_v_self->t = __pyx_t_1;

  /* function exit code */
  __pyx_r = 0;
  goto __pyx_L0;
  __pyx_L1_error:;
  __Pyx_AddTraceback("model.Bondarenko.t.__set__", __pyx_clineno, __pyx_lineno, __pyx_filename);
  __pyx_r = -1;
  __pyx_L0:;
  __Pyx_RefNannyFinishContext();
  return __pyx_r;
}
```

```
 100:
```

```
 101:
```

```
 102:
```

```
 103:     cdef double[:, ::1] V
```

```
 104:     cdef double[:, ::1] Ca2_i
```

```
 105:     cdef double[:, ::1] Ca2_ss
```

```
 106:     cdef double[:, ::1] Ca2_JSR
```

```
 107:     cdef double[:, ::1] Ca2_NSR
```

```
 108:     cdef double[:, ::1] LTRPNCa
```

```
 109:     cdef double[:, ::1] HTRPNCa
```

```
 110:     cdef double[:, ::1] O
```

```
 111:     cdef double[:, ::1] C1
```

```
 112:     cdef double[:, ::1] C2
```

```
 113:     cdef double[:, ::1] C3
```

```
 114:     cdef double[:, ::1] C4
```

```
 115:     cdef double[:, ::1] I1
```

```
 116:     cdef double[:, ::1] I2
```

```
 117:     cdef double[:, ::1] I3
```

```
 118:     cdef double[:, ::1] Pc1
```

```
 119:     cdef double[:, ::1] Pc2
```

```
 120:     cdef double[:, ::1] Po1
```

```
 121:     cdef double[:, ::1] Po2
```

```
 122:     cdef double[:, ::1] P_RyR
```

```
 123:     cdef double[:, ::1] C_Na3
```

```
 124:     cdef double[:, ::1] C_Na2
```

```
 125:     cdef double[:, ::1] C_Na1
```

```
 126:     cdef double[:, ::1] O_Na
```

```
 127:     cdef double[:, ::1] IF_Na
```

```
 128:     cdef double[:, ::1] I1_Na
```

```
 129:     cdef double[:, ::1] I2_Na
```

```
 130:     cdef double[:, ::1] IC_Na2
```

```
 131:     cdef double[:, ::1] IC_Na3
```

```
 132:     cdef double[:, ::1] Na_i
```

```
 133:     cdef double[:, ::1] K_i
```

```
 134:     cdef double[:, ::1] a_tof
```

```
 135:     cdef double[:, ::1] i_tof
```

```
 136:     cdef double[:, ::1] n_Ks
```

```
 137:     cdef double[:, ::1] a_tos
```

```
 138:     cdef double[:, ::1] i_tos
```

```
 139:     cdef double[:, ::1] a_ur
```

```
 140:     cdef double[:, ::1] i_ur
```

```
 141:     cdef double[:, ::1] a_Kss
```

```
 142:     cdef double[:, ::1] C_K0
```

```
 143:     cdef double[:, ::1] C_K1
```

```
 144:     cdef double[:, ::1] C_K2
```

```
 145:     cdef double[:, ::1] O_K
```

```
 146:     cdef double[:, ::1] I_K
```

```
 147:
```

```
 148:     @property
```

```
  __pyx_t_2 = PyTuple_New(1); if (unlikely(!__pyx_t_2)) {__pyx_filename = __pyx_f[0]; __pyx_lineno = 148; __pyx_clineno = __LINE__; goto __pyx_L1_error;}
  __Pyx_GOTREF(__pyx_t_2);
  PyTuple_SET_ITEM(__pyx_t_2, 0, __pyx_t_1);
  __Pyx_GIVEREF(__pyx_t_1);
  __pyx_t_1 = 0;
  __pyx_t_1 = __Pyx_PyObject_Call(__pyx_builtin_property, __pyx_t_2, NULL); if (unlikely(!__pyx_t_1)) {__pyx_filename = __pyx_f[0]; __pyx_lineno = 148; __pyx_clineno = __LINE__; goto __pyx_L1_error;}
  __Pyx_GOTREF(__pyx_t_1);
  __Pyx_DECREF(__pyx_t_2); __pyx_t_2 = 0;
  if (PyDict_SetItem((PyObject *)__pyx_ptype_5model_Bondarenko->tp_dict, __pyx_n_s_V, __pyx_t_1) < 0) {__pyx_filename = __pyx_f[0]; __pyx_lineno = 149; __pyx_clineno = __LINE__; goto __pyx_L1_error;}
  __Pyx_DECREF(__pyx_t_1); __pyx_t_1 = 0;
  PyType_Modified(__pyx_ptype_5model_Bondarenko);
```

```
 149:     def V(self):
```

```
/* Python wrapper */
static PyObject *__pyx_pw_5model_10Bondarenko_1V(PyObject *__pyx_v_self, CYTHON_UNUSED PyObject *unused); /*proto*/
static PyObject *__pyx_pw_5model_10Bondarenko_1V(PyObject *__pyx_v_self, CYTHON_UNUSED PyObject *unused) {
  PyObject *__pyx_r = 0;
  __Pyx_RefNannyDeclarations
  __Pyx_RefNannySetupContext("V (wrapper)", 0);
  __pyx_r = __pyx_pf_5model_10Bondarenko_V(((struct __pyx_obj_5model_Bondarenko *)__pyx_v_self));

  /* function exit code */
  __Pyx_RefNannyFinishContext();
  return __pyx_r;
}

static PyObject *__pyx_pf_5model_10Bondarenko_V(struct __pyx_obj_5model_Bondarenko *__pyx_v_self) {
  PyObject *__pyx_r = NULL;
  __Pyx_RefNannyDeclarations
  __Pyx_RefNannySetupContext("V", 0);
/* … */
  /* function exit code */
  __pyx_L1_error:;
  __Pyx_XDECREF(__pyx_t_1);
  __Pyx_XDECREF(__pyx_t_2);
  __Pyx_XDECREF(__pyx_t_3);
  __Pyx_AddTraceback("model.Bondarenko.V", __pyx_clineno, __pyx_lineno, __pyx_filename);
  __pyx_r = NULL;
  __pyx_L0:;
  __Pyx_XGIVEREF(__pyx_r);
  __Pyx_RefNannyFinishContext();
  return __pyx_r;
}
/* … */
  __pyx_t_1 = __Pyx_GetNameInClass((PyObject *)__pyx_ptype_5model_Bondarenko, __pyx_n_s_V); if (unlikely(!__pyx_t_1)) {__pyx_filename = __pyx_f[0]; __pyx_lineno = 149; __pyx_clineno = __LINE__; goto __pyx_L1_error;}
  __Pyx_GOTREF(__pyx_t_1);
```

```
 150:         return np.asarray(self.V)
```

```
  __Pyx_XDECREF(__pyx_r);
  __pyx_t_1 = __Pyx_GetModuleGlobalName(__pyx_n_s_np); if (unlikely(!__pyx_t_1)) {__pyx_filename = __pyx_f[0]; __pyx_lineno = 150; __pyx_clineno = __LINE__; goto __pyx_L1_error;}
  __Pyx_GOTREF(__pyx_t_1);
  __pyx_t_2 = __Pyx_PyObject_GetAttrStr(__pyx_t_1, __pyx_n_s_asarray); if (unlikely(!__pyx_t_2)) {__pyx_filename = __pyx_f[0]; __pyx_lineno = 150; __pyx_clineno = __LINE__; goto __pyx_L1_error;}
  __Pyx_GOTREF(__pyx_t_2);
  __Pyx_DECREF(__pyx_t_1); __pyx_t_1 = 0;
  __pyx_t_1 = __Pyx_PyObject_GetAttrStr(((PyObject *)__pyx_v_self), __pyx_n_s_V); if (unlikely(!__pyx_t_1)) {__pyx_filename = __pyx_f[0]; __pyx_lineno = 150; __pyx_clineno = __LINE__; goto __pyx_L1_error;}
  __Pyx_GOTREF(__pyx_t_1);
  __pyx_t_3 = PyTuple_New(1); if (unlikely(!__pyx_t_3)) {__pyx_filename = __pyx_f[0]; __pyx_lineno = 150; __pyx_clineno = __LINE__; goto __pyx_L1_error;}
  __Pyx_GOTREF(__pyx_t_3);
  PyTuple_SET_ITEM(__pyx_t_3, 0, __pyx_t_1);
  __Pyx_GIVEREF(__pyx_t_1);
  __pyx_t_1 = 0;
  __pyx_t_1 = __Pyx_PyObject_Call(__pyx_t_2, __pyx_t_3, NULL); if (unlikely(!__pyx_t_1)) {__pyx_filename = __pyx_f[0]; __pyx_lineno = 150; __pyx_clineno = __LINE__; goto __pyx_L1_error;}
  __Pyx_GOTREF(__pyx_t_1);
  __Pyx_DECREF(__pyx_t_2); __pyx_t_2 = 0;
  __Pyx_DECREF(__pyx_t_3); __pyx_t_3 = 0;
  __pyx_r = __pyx_t_1;
  __pyx_t_1 = 0;
  goto __pyx_L0;
```

```
 151:     @property
```

```
  __pyx_t_2 = PyTuple_New(1); if (unlikely(!__pyx_t_2)) {__pyx_filename = __pyx_f[0]; __pyx_lineno = 151; __pyx_clineno = __LINE__; goto __pyx_L1_error;}
  __Pyx_GOTREF(__pyx_t_2);
  PyTuple_SET_ITEM(__pyx_t_2, 0, __pyx_t_1);
  __Pyx_GIVEREF(__pyx_t_1);
  __pyx_t_1 = 0;
  __pyx_t_1 = __Pyx_PyObject_Call(__pyx_builtin_property, __pyx_t_2, NULL); if (unlikely(!__pyx_t_1)) {__pyx_filename = __pyx_f[0]; __pyx_lineno = 151; __pyx_clineno = __LINE__; goto __pyx_L1_error;}
  __Pyx_GOTREF(__pyx_t_1);
  __Pyx_DECREF(__pyx_t_2); __pyx_t_2 = 0;
  if (PyDict_SetItem((PyObject *)__pyx_ptype_5model_Bondarenko->tp_dict, __pyx_n_s_Ca2_i, __pyx_t_1) < 0) {__pyx_filename = __pyx_f[0]; __pyx_lineno = 152; __pyx_clineno = __LINE__; goto __pyx_L1_error;}
  __Pyx_DECREF(__pyx_t_1); __pyx_t_1 = 0;
  PyType_Modified(__pyx_ptype_5model_Bondarenko);
```

```
 152:     def Ca2_i(self):
```

```
/* Python wrapper */
static PyObject *__pyx_pw_5model_10Bondarenko_3Ca2_i(PyObject *__pyx_v_self, CYTHON_UNUSED PyObject *unused); /*proto*/
static PyObject *__pyx_pw_5model_10Bondarenko_3Ca2_i(PyObject *__pyx_v_self, CYTHON_UNUSED PyObject *unused) {
  PyObject *__pyx_r = 0;
  __Pyx_RefNannyDeclarations
  __Pyx_RefNannySetupContext("Ca2_i (wrapper)", 0);
  __pyx_r = __pyx_pf_5model_10Bondarenko_2Ca2_i(((struct __pyx_obj_5model_Bondarenko *)__pyx_v_self));

  /* function exit code */
  __Pyx_RefNannyFinishContext();
  return __pyx_r;
}

static PyObject *__pyx_pf_5model_10Bondarenko_2Ca2_i(struct __pyx_obj_5model_Bondarenko *__pyx_v_self) {
  PyObject *__pyx_r = NULL;
  __Pyx_RefNannyDeclarations
  __Pyx_RefNannySetupContext("Ca2_i", 0);
/* … */
  /* function exit code */
  __pyx_L1_error:;
  __Pyx_XDECREF(__pyx_t_1);
  __Pyx_XDECREF(__pyx_t_2);
  __Pyx_XDECREF(__pyx_t_3);
  __Pyx_AddTraceback("model.Bondarenko.Ca2_i", __pyx_clineno, __pyx_lineno, __pyx_filename);
  __pyx_r = NULL;
  __pyx_L0:;
  __Pyx_XGIVEREF(__pyx_r);
  __Pyx_RefNannyFinishContext();
  return __pyx_r;
}
/* … */
  __pyx_t_1 = __Pyx_GetNameInClass((PyObject *)__pyx_ptype_5model_Bondarenko, __pyx_n_s_Ca2_i); if (unlikely(!__pyx_t_1)) {__pyx_filename = __pyx_f[0]; __pyx_lineno = 152; __pyx_clineno = __LINE__; goto __pyx_L1_error;}
  __Pyx_GOTREF(__pyx_t_1);
```

```
 153:         return np.asarray(self.Ca2_i)
```

```
  __Pyx_XDECREF(__pyx_r);
  __pyx_t_1 = __Pyx_GetModuleGlobalName(__pyx_n_s_np); if (unlikely(!__pyx_t_1)) {__pyx_filename = __pyx_f[0]; __pyx_lineno = 153; __pyx_clineno = __LINE__; goto __pyx_L1_error;}
  __Pyx_GOTREF(__pyx_t_1);
  __pyx_t_2 = __Pyx_PyObject_GetAttrStr(__pyx_t_1, __pyx_n_s_asarray); if (unlikely(!__pyx_t_2)) {__pyx_filename = __pyx_f[0]; __pyx_lineno = 153; __pyx_clineno = __LINE__; goto __pyx_L1_error;}
  __Pyx_GOTREF(__pyx_t_2);
  __Pyx_DECREF(__pyx_t_1); __pyx_t_1 = 0;
  __pyx_t_1 = __Pyx_PyObject_GetAttrStr(((PyObject *)__pyx_v_self), __pyx_n_s_Ca2_i); if (unlikely(!__pyx_t_1)) {__pyx_filename = __pyx_f[0]; __pyx_lineno = 153; __pyx_clineno = __LINE__; goto __pyx_L1_error;}
  __Pyx_GOTREF(__pyx_t_1);
  __pyx_t_3 = PyTuple_New(1); if (unlikely(!__pyx_t_3)) {__pyx_filename = __pyx_f[0]; __pyx_lineno = 153; __pyx_clineno = __LINE__; goto __pyx_L1_error;}
  __Pyx_GOTREF(__pyx_t_3);
  PyTuple_SET_ITEM(__pyx_t_3, 0, __pyx_t_1);
  __Pyx_GIVEREF(__pyx_t_1);
  __pyx_t_1 = 0;
  __pyx_t_1 = __Pyx_PyObject_Call(__pyx_t_2, __pyx_t_3, NULL); if (unlikely(!__pyx_t_1)) {__pyx_filename = __pyx_f[0]; __pyx_lineno = 153; __pyx_clineno = __LINE__; goto __pyx_L1_error;}
  __Pyx_GOTREF(__pyx_t_1);
  __Pyx_DECREF(__pyx_t_2); __pyx_t_2 = 0;
  __Pyx_DECREF(__pyx_t_3); __pyx_t_3 = 0;
  __pyx_r = __pyx_t_1;
  __pyx_t_1 = 0;
  goto __pyx_L0;
```

```
 154:
```

```
 155:     #def __init__(self, frq, offtime, duration, dx=.5, dy=.5, shape = (50, 50)):    #initialize the class
```

```
 156:     def __init__(self, dx=.5, dy=.5, shape = (50, 50)):
```

```
/* Python wrapper */
static int __pyx_pw_5model_10Bondarenko_5__init__(PyObject *__pyx_v_self, PyObject *__pyx_args, PyObject *__pyx_kwds); /*proto*/
static int __pyx_pw_5model_10Bondarenko_5__init__(PyObject *__pyx_v_self, PyObject *__pyx_args, PyObject *__pyx_kwds) {
  PyObject *__pyx_v_dx = 0;
  PyObject *__pyx_v_dy = 0;
  PyObject *__pyx_v_shape = 0;
  int __pyx_r;
  __Pyx_RefNannyDeclarations
  __Pyx_RefNannySetupContext("__init__ (wrapper)", 0);
  {
    static PyObject **__pyx_pyargnames[] = {&__pyx_n_s_dx,&__pyx_n_s_dy,&__pyx_n_s_shape,0};
    PyObject* values[3] = {0,0,0};
    values[0] = ((PyObject *)__pyx_float__5);
    values[1] = ((PyObject *)__pyx_float__5);
    values[2] = ((PyObject *)__pyx_tuple_);
    if (unlikely(__pyx_kwds)) {
      Py_ssize_t kw_args;
      const Py_ssize_t pos_args = PyTuple_GET_SIZE(__pyx_args);
      switch (pos_args) {
        case  3: values[2] = PyTuple_GET_ITEM(__pyx_args, 2);
        case  2: values[1] = PyTuple_GET_ITEM(__pyx_args, 1);
        case  1: values[0] = PyTuple_GET_ITEM(__pyx_args, 0);
        case  0: break;
        default: goto __pyx_L5_argtuple_error;
      }
      kw_args = PyDict_Size(__pyx_kwds);
      switch (pos_args) {
        case  0:
        if (kw_args > 0) {
          PyObject* value = PyDict_GetItem(__pyx_kwds, __pyx_n_s_dx);
          if (value) { values[0] = value; kw_args--; }
        }
        case  1:
        if (kw_args > 0) {
          PyObject* value = PyDict_GetItem(__pyx_kwds, __pyx_n_s_dy);
          if (value) { values[1] = value; kw_args--; }
        }
        case  2:
        if (kw_args > 0) {
          PyObject* value = PyDict_GetItem(__pyx_kwds, __pyx_n_s_shape);
          if (value) { values[2] = value; kw_args--; }
        }
      }
      if (unlikely(kw_args > 0)) {
        if (unlikely(__Pyx_ParseOptionalKeywords(__pyx_kwds, __pyx_pyargnames, 0, values, pos_args, "__init__") < 0)) {__pyx_filename = __pyx_f[0]; __pyx_lineno = 156; __pyx_clineno = __LINE__; goto __pyx_L3_error;}
      }
    } else {
      switch (PyTuple_GET_SIZE(__pyx_args)) {
        case  3: values[2] = PyTuple_GET_ITEM(__pyx_args, 2);
        case  2: values[1] = PyTuple_GET_ITEM(__pyx_args, 1);
        case  1: values[0] = PyTuple_GET_ITEM(__pyx_args, 0);
        case  0: break;
        default: goto __pyx_L5_argtuple_error;
      }
    }
    __pyx_v_dx = values[0];
    __pyx_v_dy = values[1];
    __pyx_v_shape = values[2];
  }
  goto __pyx_L4_argument_unpacking_done;
  __pyx_L5_argtuple_error:;
  __Pyx_RaiseArgtupleInvalid("__init__", 0, 0, 3, PyTuple_GET_SIZE(__pyx_args)); {__pyx_filename = __pyx_f[0]; __pyx_lineno = 156; __pyx_clineno = __LINE__; goto __pyx_L3_error;}
  __pyx_L3_error:;
  __Pyx_AddTraceback("model.Bondarenko.__init__", __pyx_clineno, __pyx_lineno, __pyx_filename);
  __Pyx_RefNannyFinishContext();
  return -1;
  __pyx_L4_argument_unpacking_done:;
  __pyx_r = __pyx_pf_5model_10Bondarenko_4__init__(((struct __pyx_obj_5model_Bondarenko *)__pyx_v_self), __pyx_v_dx, __pyx_v_dy, __pyx_v_shape);

  /* function exit code */
  __Pyx_RefNannyFinishContext();
  return __pyx_r;
}

static int __pyx_pf_5model_10Bondarenko_4__init__(struct __pyx_obj_5model_Bondarenko *__pyx_v_self, PyObject *__pyx_v_dx, PyObject *__pyx_v_dy, PyObject *__pyx_v_shape) {
  int __pyx_r;
  __Pyx_RefNannyDeclarations
  __Pyx_RefNannySetupContext("__init__", 0);
/* … */
  __pyx_tuple_ = PyTuple_Pack(2, __pyx_int_50, __pyx_int_50); if (unlikely(!__pyx_tuple_)) {__pyx_filename = __pyx_f[0]; __pyx_lineno = 156; __pyx_clineno = __LINE__; goto __pyx_L1_error;}
  __Pyx_GOTREF(__pyx_tuple_);
  __Pyx_GIVEREF(__pyx_tuple_);
/* … */
  /* function exit code */
  __pyx_r = 0;
  goto __pyx_L0;
  __pyx_L1_error:;
  __Pyx_XDECREF(__pyx_t_3);
  __Pyx_XDECREF(__pyx_t_5);
  __Pyx_XDECREF(__pyx_t_6);
  __PYX_XDEC_MEMVIEW(&__pyx_t_7, 1);
  __PYX_XDEC_MEMVIEW(&__pyx_t_8, 1);
  __PYX_XDEC_MEMVIEW(&__pyx_t_9, 1);
  __PYX_XDEC_MEMVIEW(&__pyx_t_10, 1);
  __PYX_XDEC_MEMVIEW(&__pyx_t_11, 1);
  __PYX_XDEC_MEMVIEW(&__pyx_t_12, 1);
  __PYX_XDEC_MEMVIEW(&__pyx_t_13, 1);
  __PYX_XDEC_MEMVIEW(&__pyx_t_14, 1);
  __PYX_XDEC_MEMVIEW(&__pyx_t_15, 1);
  __PYX_XDEC_MEMVIEW(&__pyx_t_16, 1);
  __PYX_XDEC_MEMVIEW(&__pyx_t_17, 1);
  __PYX_XDEC_MEMVIEW(&__pyx_t_18, 1);
  __PYX_XDEC_MEMVIEW(&__pyx_t_19, 1);
  __PYX_XDEC_MEMVIEW(&__pyx_t_20, 1);
  __PYX_XDEC_MEMVIEW(&__pyx_t_21, 1);
  __PYX_XDEC_MEMVIEW(&__pyx_t_22, 1);
  __PYX_XDEC_MEMVIEW(&__pyx_t_23, 1);
  __PYX_XDEC_MEMVIEW(&__pyx_t_24, 1);
  __PYX_XDEC_MEMVIEW(&__pyx_t_25, 1);
  __PYX_XDEC_MEMVIEW(&__pyx_t_26, 1);
  __PYX_XDEC_MEMVIEW(&__pyx_t_27, 1);
  __PYX_XDEC_MEMVIEW(&__pyx_t_28, 1);
  __PYX_XDEC_MEMVIEW(&__pyx_t_29, 1);
  __PYX_XDEC_MEMVIEW(&__pyx_t_30, 1);
  __PYX_XDEC_MEMVIEW(&__pyx_t_31, 1);
  __PYX_XDEC_MEMVIEW(&__pyx_t_32, 1);
  __PYX_XDEC_MEMVIEW(&__pyx_t_33, 1);
  __PYX_XDEC_MEMVIEW(&__pyx_t_34, 1);
  __PYX_XDEC_MEMVIEW(&__pyx_t_35, 1);
  __PYX_XDEC_MEMVIEW(&__pyx_t_36, 1);
  __PYX_XDEC_MEMVIEW(&__pyx_t_37, 1);
  __PYX_XDEC_MEMVIEW(&__pyx_t_38, 1);
  __PYX_XDEC_MEMVIEW(&__pyx_t_39, 1);
  __PYX_XDEC_MEMVIEW(&__pyx_t_40, 1);
  __PYX_XDEC_MEMVIEW(&__pyx_t_41, 1);
  __PYX_XDEC_MEMVIEW(&__pyx_t_42, 1);
  __PYX_XDEC_MEMVIEW(&__pyx_t_43, 1);
  __PYX_XDEC_MEMVIEW(&__pyx_t_44, 1);
  __Pyx_AddTraceback("model.Bondarenko.__init__", __pyx_clineno, __pyx_lineno, __pyx_filename);
  __pyx_r = -1;
  __pyx_L0:;
  __Pyx_RefNannyFinishContext();
  return __pyx_r;
}
```

```
 157:         if len(shape) != 2:
```

```
  __pyx_t_1 = PyObject_Length(__pyx_v_shape); if (unlikely(__pyx_t_1 == -1)) {__pyx_filename = __pyx_f[0]; __pyx_lineno = 157; __pyx_clineno = __LINE__; goto __pyx_L1_error;}
  __pyx_t_2 = ((__pyx_t_1 != 2) != 0);
  if (__pyx_t_2) {
```

```
 158:             raise ValueError("must be 2D.")
```

```
    __pyx_t_3 = __Pyx_PyObject_Call(__pyx_builtin_ValueError, __pyx_tuple__2, NULL); if (unlikely(!__pyx_t_3)) {__pyx_filename = __pyx_f[0]; __pyx_lineno = 158; __pyx_clineno = __LINE__; goto __pyx_L1_error;}
    __Pyx_GOTREF(__pyx_t_3);
    __Pyx_Raise(__pyx_t_3, 0, 0, 0);
    __Pyx_DECREF(__pyx_t_3); __pyx_t_3 = 0;
    {__pyx_filename = __pyx_f[0]; __pyx_lineno = 158; __pyx_clineno = __LINE__; goto __pyx_L1_error;}
  }
/* … */
  __pyx_tuple__2 = PyTuple_Pack(1, __pyx_kp_u_must_be_2D); if (unlikely(!__pyx_tuple__2)) {__pyx_filename = __pyx_f[0]; __pyx_lineno = 158; __pyx_clineno = __LINE__; goto __pyx_L1_error;}
  __Pyx_GOTREF(__pyx_tuple__2);
  __Pyx_GIVEREF(__pyx_tuple__2);
```

```
 159:
```

```
 160:         self.A_cap = 1.534e-4
```

```
  __pyx_v_self->A_cap = 1.534e-4;
```

```
 161:         self.V_myo = 25.84e-6
```

```
  __pyx_v_self->V_myo = 25.84e-6;
```

```
 162:         self.V_JSR = 0.12e-6
```

```
  __pyx_v_self->V_JSR = 0.12e-6;
```

```
 163:         self.V_NSR = 2.098e-6
```

```
  __pyx_v_self->V_NSR = 2.098e-6;
```

```
 164:         self.V_ss  = 1.485e-9
```

```
  __pyx_v_self->V_ss = 1.485e-9;
```

```
 165:         self.K_0   = 5400.0
```

```
  __pyx_v_self->K_0 = 5400.0;
```

```
 166:         self.Na_0  = 140000.0
```

```
  __pyx_v_self->Na_0 = 140000.0;
```

```
 167:         self.Ca_0  = 1800.0
```

```
  __pyx_v_self->Ca_0 = 1800.0;
```

```
 168:         self.v_1    = 4.5
```

```
  __pyx_v_self->v_1 = 4.5;
```

```
 169:         self.v_2    = 1.74e-5
```

```
  __pyx_v_self->v_2 = 1.74e-5;
```

```
 170:         self.v_3    = 0.45
```

```
  __pyx_v_self->v_3 = 0.45;
```

```
 171:         self.k_mup  = 0.5
```

```
  __pyx_v_self->k_mup = 0.5;
```

```
 172:         self.t_tr   = 20.0
```

```
  __pyx_v_self->t_tr = 20.0;
```

```
 173:         self.t_xfer = 8.0
```

```
  __pyx_v_self->t_xfer = 8.0;
```

```
 174:         self.k_p_a  = 0.006075
```

```
  __pyx_v_self->k_p_a = 0.006075;
```

```
 175:         self.k_n_a  = 0.07125
```

```
  __pyx_v_self->k_n_a = 0.07125;
```

```
 176:         self.k_p_b  = 0.00405
```

```
  __pyx_v_self->k_p_b = 0.00405;
```

```
 177:         self.k_n_b  = 0.965
```

```
  __pyx_v_self->k_n_b = 0.965;
```

```
 178:         self.k_p_c  = 0.009
```

```
  __pyx_v_self->k_p_c = 0.009;
```

```
 179:         self.k_n_c  = 0.0008
```

```
  __pyx_v_self->k_n_c = 0.0008;
```

```
 180:         self.n      = 4.0
```

```
  __pyx_v_self->n = 4.0;
```

```
 181:         self.m      = 3.0
```

```
  __pyx_v_self->m = 3.0;
```

```
 182:         self.G_CaL     = 0.1729
```

```
  __pyx_v_self->G_CaL = 0.1729;
```

```
 183:         self.E_CaL     = 63.0
```

```
  __pyx_v_self->E_CaL = 63.0;
```

```
 184:         self.k_pcmax   = 0.23324
```

```
  __pyx_v_self->k_pcmax = 0.23324;
```

```
 185:         self.k_pchalf  = 20.0
```

```
  __pyx_v_self->k_pchalf = 20.0;
```

```
 186:         self.k_pcb     = 0.0005
```

```
  __pyx_v_self->k_pcb = 0.0005;
```

```
 187:         self.I_CaLmax  = 7.0
```

```
  __pyx_v_self->I_CaLmax = 7.0;
```

```
 188:         self.LTRPN_tot = 70.0
```

```
  __pyx_v_self->LTRPN_tot = 70.0;
```

```
 189:         self.HTRPN_tot = 140.0
```

```
  __pyx_v_self->HTRPN_tot = 140.0;
```

```
 190:         self.k_p_htrpn = 0.00237
```

```
  __pyx_v_self->k_p_htrpn = 0.00237;
```

```
 191:         self.k_n_htrpn = 3.2e-5
```

```
  __pyx_v_self->k_n_htrpn = 3.2e-5;
```

```
 192:         self.k_p_ltrpn = 0.0327
```

```
  __pyx_v_self->k_p_ltrpn = 0.0327;
```

```
 193:         self.k_n_ltrpn = 0.0196
```

```
  __pyx_v_self->k_n_ltrpn = 0.0196;
```

```
 194:         self.CMDN_tot  = 50.0
```

```
  __pyx_v_self->CMDN_tot = 50.0;
```

```
 195:         self.CSQN_tot  = 15000.0
```

```
  __pyx_v_self->CSQN_tot = 15000.0;
```

```
 196:         self.k_CMDN_m  = 0.238
```

```
  __pyx_v_self->k_CMDN_m = 0.238;
```

```
 197:         self.k_CSQN_m  = 800.0
```

```
  __pyx_v_self->k_CSQN_m = 800.0;
```

```
 198:         self.C_m       = 1.0
```

```
  __pyx_v_self->C_m = 1.0;
```

```
 199:         self.F         = 96.5
```

```
  __pyx_v_self->F = 96.5;
```

```
 200:         self.T         = 298.0
```

```
  __pyx_v_self->T = 298.0;
```

```
 201:         self.R         = 8.314
```

```
  __pyx_v_self->R = 8.314;
```

```
 202:         self.k_NaCa    = 292.8
```

```
  __pyx_v_self->k_NaCa = 292.8;
```

```
 203:         self.k_mNa     = 87500.0
```

```
  __pyx_v_self->k_mNa = 87500.0;
```

```
 204:         self.k_mCa     = 1380.0
```

```
  __pyx_v_self->k_mCa = 1380.0;
```

```
 205:         self.k_sat     = 0.1
```

```
  __pyx_v_self->k_sat = 0.1;
```

```
 206:         self.miu       = 0.35
```

```
  __pyx_v_self->miu = 0.35;
```

```
 207:         self.I_maxNaK  = 0.88
```

```
  __pyx_v_self->I_maxNaK = 0.88;
```

```
 208:         self.K_mNai    = 21000.0
```

```
  __pyx_v_self->K_mNai = 21000.0;
```

```
 209:         self.K_mKo     = 1500.0
```

```
  __pyx_v_self->K_mKo = 1500.0;
```

```
 210:         self.I_maxpCa  = 1.0
```

```
  __pyx_v_self->I_maxpCa = 1.0;
```

```
 211:         self.k_mpCa    = 0.5
```

```
  __pyx_v_self->k_mpCa = 0.5;
```

```
 212:         self.G_Cab     = 0.000367
```

```
  __pyx_v_self->G_Cab = 0.000367;
```

```
 213:         self.G_Na      = 13.0
```

```
  __pyx_v_self->G_Na = 13.0;
```

```
 214:         self.G_Nab     = 0.0026
```

```
  __pyx_v_self->G_Nab = 0.0026;
```

```
 215:         self.G_Ktof    = 0.4067       #(apex)
```

```
  __pyx_v_self->G_Ktof = 0.4067;
```

```
 216:         #self.G_Ktof    = 0.0798      #(septum)
```

```
 217:         self.G_Ks      = 0.00575
```

```
  __pyx_v_self->G_Ks = 0.00575;
```

```
 218:         self.G_Ktos    = 0.0          #(apex)
```

```
  __pyx_v_self->G_Ktos = 0.0;
```

```
 219:         self.G_Kur     = 0.160        #(apex)
```

```
  __pyx_v_self->G_Kur = 0.160;
```

```
 220:         self.G_Kss     = 0.050        #(apex)
```

```
  __pyx_v_self->G_Kss = 0.050;
```

```
 221:         #self.G_Ktos    = 0.0629      #(septum)
```

```
 222:         #self.G_Kur     = 0.0975      #(septum)
```

```
 223:         #self.G_Kss     = 0.0324      #(septum)
```

```
 224:         self.G_Kr      = 0.078
```

```
  __pyx_v_self->G_Kr = 0.078;
```

```
 225:         self.k_f       = 0.023761
```

```
  __pyx_v_self->k_f = 0.023761;
```

```
 226:         self.k_b       = 0.036778
```

```
  __pyx_v_self->k_b = 0.036778;
```

```
 227:         self.G_ClCa    = 10.0
```

```
  __pyx_v_self->G_ClCa = 10.0;
```

```
 228:         self.K_mCl     = 10.0
```

```
  __pyx_v_self->K_mCl = 10.0;
```

```
 229:         self.E_Cl      = -40.0
```

```
  __pyx_v_self->E_Cl = -40.0;
```

```
 230:         self.Laplas_c = 0.00422
```

```
  __pyx_v_self->Laplas_c = 0.00422;
```

```
 231:         self.Laplas_r = 3.06129
```

```
  __pyx_v_self->Laplas_r = 3.06129;
```

```
 232:         self.V_ChR2 = 0
```

```
  __pyx_v_self->V_ChR2 = 0.0;
```

```
 233:         self.i_Kss = 1.
```

```
  __pyx_v_self->i_Kss = 1.;
```

```
 234:         self.h = 0.0001
```

```
  __pyx_v_self->h = 0.0001;
```

```
 235:         self.dx = dx
```

```
  __pyx_t_4 = __pyx_PyFloat_AsDouble(__pyx_v_dx); if (unlikely((__pyx_t_4 == (double)-1) && PyErr_Occurred())) {__pyx_filename = __pyx_f[0]; __pyx_lineno = 235; __pyx_clineno = __LINE__; goto __pyx_L1_error;}
  __pyx_v_self->dx = __pyx_t_4;
```

```
 236:         self.dy = dy
```

```
  __pyx_t_4 = __pyx_PyFloat_AsDouble(__pyx_v_dy); if (unlikely((__pyx_t_4 == (double)-1) && PyErr_Occurred())) {__pyx_filename = __pyx_f[0]; __pyx_lineno = 236; __pyx_clineno = __LINE__; goto __pyx_L1_error;}
  __pyx_v_self->dy = __pyx_t_4;
```

```
 237:
```

```
 238:
```

```
 239:
```

```
 240:         self.t = 0.0
```

```
  __pyx_v_self->t = 0.0;
```

```
 241:         self.V = np.full(shape, -82.4202)
```

```
  __pyx_t_3 = __Pyx_GetModuleGlobalName(__pyx_n_s_np); if (unlikely(!__pyx_t_3)) {__pyx_filename = __pyx_f[0]; __pyx_lineno = 241; __pyx_clineno = __LINE__; goto __pyx_L1_error;}
  __Pyx_GOTREF(__pyx_t_3);
  __pyx_t_5 = __Pyx_PyObject_GetAttrStr(__pyx_t_3, __pyx_n_s_full); if (unlikely(!__pyx_t_5)) {__pyx_filename = __pyx_f[0]; __pyx_lineno = 241; __pyx_clineno = __LINE__; goto __pyx_L1_error;}
  __Pyx_GOTREF(__pyx_t_5);
  __Pyx_DECREF(__pyx_t_3); __pyx_t_3 = 0;
  __pyx_t_3 = PyTuple_New(2); if (unlikely(!__pyx_t_3)) {__pyx_filename = __pyx_f[0]; __pyx_lineno = 241; __pyx_clineno = __LINE__; goto __pyx_L1_error;}
  __Pyx_GOTREF(__pyx_t_3);
  __Pyx_INCREF(__pyx_v_shape);
  PyTuple_SET_ITEM(__pyx_t_3, 0, __pyx_v_shape);
  __Pyx_GIVEREF(__pyx_v_shape);
  __Pyx_INCREF(__pyx_float_neg_82_4202);
  PyTuple_SET_ITEM(__pyx_t_3, 1, __pyx_float_neg_82_4202);
  __Pyx_GIVEREF(__pyx_float_neg_82_4202);
  __pyx_t_6 = __Pyx_PyObject_Call(__pyx_t_5, __pyx_t_3, NULL); if (unlikely(!__pyx_t_6)) {__pyx_filename = __pyx_f[0]; __pyx_lineno = 241; __pyx_clineno = __LINE__; goto __pyx_L1_error;}
  __Pyx_GOTREF(__pyx_t_6);
  __Pyx_DECREF(__pyx_t_5); __pyx_t_5 = 0;
  __Pyx_DECREF(__pyx_t_3); __pyx_t_3 = 0;
  if (__Pyx_PyObject_SetAttrStr(((PyObject *)__pyx_v_self), __pyx_n_s_V, __pyx_t_6) < 0) {__pyx_filename = __pyx_f[0]; __pyx_lineno = 241; __pyx_clineno = __LINE__; goto __pyx_L1_error;}
  __Pyx_DECREF(__pyx_t_6); __pyx_t_6 = 0;
```

```
 242:         self.Ca2_i = np.full(shape, 0.115001)
```

```
  __pyx_t_6 = __Pyx_GetModuleGlobalName(__pyx_n_s_np); if (unlikely(!__pyx_t_6)) {__pyx_filename = __pyx_f[0]; __pyx_lineno = 242; __pyx_clineno = __LINE__; goto __pyx_L1_error;}
  __Pyx_GOTREF(__pyx_t_6);
  __pyx_t_3 = __Pyx_PyObject_GetAttrStr(__pyx_t_6, __pyx_n_s_full); if (unlikely(!__pyx_t_3)) {__pyx_filename = __pyx_f[0]; __pyx_lineno = 242; __pyx_clineno = __LINE__; goto __pyx_L1_error;}
  __Pyx_GOTREF(__pyx_t_3);
  __Pyx_DECREF(__pyx_t_6); __pyx_t_6 = 0;
  __pyx_t_6 = PyTuple_New(2); if (unlikely(!__pyx_t_6)) {__pyx_filename = __pyx_f[0]; __pyx_lineno = 242; __pyx_clineno = __LINE__; goto __pyx_L1_error;}
  __Pyx_GOTREF(__pyx_t_6);
  __Pyx_INCREF(__pyx_v_shape);
  PyTuple_SET_ITEM(__pyx_t_6, 0, __pyx_v_shape);
  __Pyx_GIVEREF(__pyx_v_shape);
  __Pyx_INCREF(__pyx_float_0_115001);
  PyTuple_SET_ITEM(__pyx_t_6, 1, __pyx_float_0_115001);
  __Pyx_GIVEREF(__pyx_float_0_115001);
  __pyx_t_5 = __Pyx_PyObject_Call(__pyx_t_3, __pyx_t_6, NULL); if (unlikely(!__pyx_t_5)) {__pyx_filename = __pyx_f[0]; __pyx_lineno = 242; __pyx_clineno = __LINE__; goto __pyx_L1_error;}
  __Pyx_GOTREF(__pyx_t_5);
  __Pyx_DECREF(__pyx_t_3); __pyx_t_3 = 0;
  __Pyx_DECREF(__pyx_t_6); __pyx_t_6 = 0;
  if (__Pyx_PyObject_SetAttrStr(((PyObject *)__pyx_v_self), __pyx_n_s_Ca2_i, __pyx_t_5) < 0) {__pyx_filename = __pyx_f[0]; __pyx_lineno = 242; __pyx_clineno = __LINE__; goto __pyx_L1_error;}
  __Pyx_DECREF(__pyx_t_5); __pyx_t_5 = 0;
```

```
 243:         self.Ca2_ss = np.full(shape, 0.115001)
```

```
  __pyx_t_5 = __Pyx_GetModuleGlobalName(__pyx_n_s_np); if (unlikely(!__pyx_t_5)) {__pyx_filename = __pyx_f[0]; __pyx_lineno = 243; __pyx_clineno = __LINE__; goto __pyx_L1_error;}
  __Pyx_GOTREF(__pyx_t_5);
  __pyx_t_6 = __Pyx_PyObject_GetAttrStr(__pyx_t_5, __pyx_n_s_full); if (unlikely(!__pyx_t_6)) {__pyx_filename = __pyx_f[0]; __pyx_lineno = 243; __pyx_clineno = __LINE__; goto __pyx_L1_error;}
  __Pyx_GOTREF(__pyx_t_6);
  __Pyx_DECREF(__pyx_t_5); __pyx_t_5 = 0;
  __pyx_t_5 = PyTuple_New(2); if (unlikely(!__pyx_t_5)) {__pyx_filename = __pyx_f[0]; __pyx_lineno = 243; __pyx_clineno = __LINE__; goto __pyx_L1_error;}
  __Pyx_GOTREF(__pyx_t_5);
  __Pyx_INCREF(__pyx_v_shape);
  PyTuple_SET_ITEM(__pyx_t_5, 0, __pyx_v_shape);
  __Pyx_GIVEREF(__pyx_v_shape);
  __Pyx_INCREF(__pyx_float_0_115001);
  PyTuple_SET_ITEM(__pyx_t_5, 1, __pyx_float_0_115001);
  __Pyx_GIVEREF(__pyx_float_0_115001);
  __pyx_t_3 = __Pyx_PyObject_Call(__pyx_t_6, __pyx_t_5, NULL); if (unlikely(!__pyx_t_3)) {__pyx_filename = __pyx_f[0]; __pyx_lineno = 243; __pyx_clineno = __LINE__; goto __pyx_L1_error;}
  __Pyx_GOTREF(__pyx_t_3);
  __Pyx_DECREF(__pyx_t_6); __pyx_t_6 = 0;
  __Pyx_DECREF(__pyx_t_5); __pyx_t_5 = 0;
  __pyx_t_7 = __Pyx_PyObject_to_MemoryviewSlice_d_dc_double(__pyx_t_3);
  if (unlikely(!__pyx_t_7.memview)) {__pyx_filename = __pyx_f[0]; __pyx_lineno = 243; __pyx_clineno = __LINE__; goto __pyx_L1_error;}
  __Pyx_DECREF(__pyx_t_3); __pyx_t_3 = 0;
  __PYX_XDEC_MEMVIEW(&__pyx_v_self->Ca2_ss, 0);
  __pyx_v_self->Ca2_ss = __pyx_t_7;
  __pyx_t_7.memview = NULL;
  __pyx_t_7.data = NULL;
```

```
 244:         self.Ca2_JSR = np.full(shape, 1299.5)
```

```
  __pyx_t_3 = __Pyx_GetModuleGlobalName(__pyx_n_s_np); if (unlikely(!__pyx_t_3)) {__pyx_filename = __pyx_f[0]; __pyx_lineno = 244; __pyx_clineno = __LINE__; goto __pyx_L1_error;}
  __Pyx_GOTREF(__pyx_t_3);
  __pyx_t_5 = __Pyx_PyObject_GetAttrStr(__pyx_t_3, __pyx_n_s_full); if (unlikely(!__pyx_t_5)) {__pyx_filename = __pyx_f[0]; __pyx_lineno = 244; __pyx_clineno = __LINE__; goto __pyx_L1_error;}
  __Pyx_GOTREF(__pyx_t_5);
  __Pyx_DECREF(__pyx_t_3); __pyx_t_3 = 0;
  __pyx_t_3 = PyTuple_New(2); if (unlikely(!__pyx_t_3)) {__pyx_filename = __pyx_f[0]; __pyx_lineno = 244; __pyx_clineno = __LINE__; goto __pyx_L1_error;}
  __Pyx_GOTREF(__pyx_t_3);
  __Pyx_INCREF(__pyx_v_shape);
  PyTuple_SET_ITEM(__pyx_t_3, 0, __pyx_v_shape);
  __Pyx_GIVEREF(__pyx_v_shape);
  __Pyx_INCREF(__pyx_float_1299_5);
  PyTuple_SET_ITEM(__pyx_t_3, 1, __pyx_float_1299_5);
  __Pyx_GIVEREF(__pyx_float_1299_5);
  __pyx_t_6 = __Pyx_PyObject_Call(__pyx_t_5, __pyx_t_3, NULL); if (unlikely(!__pyx_t_6)) {__pyx_filename = __pyx_f[0]; __pyx_lineno = 244; __pyx_clineno = __LINE__; goto __pyx_L1_error;}
  __Pyx_GOTREF(__pyx_t_6);
  __Pyx_DECREF(__pyx_t_5); __pyx_t_5 = 0;
  __Pyx_DECREF(__pyx_t_3); __pyx_t_3 = 0;
  __pyx_t_8 = __Pyx_PyObject_to_MemoryviewSlice_d_dc_double(__pyx_t_6);
  if (unlikely(!__pyx_t_8.memview)) {__pyx_filename = __pyx_f[0]; __pyx_lineno = 244; __pyx_clineno = __LINE__; goto __pyx_L1_error;}
  __Pyx_DECREF(__pyx_t_6); __pyx_t_6 = 0;
  __PYX_XDEC_MEMVIEW(&__pyx_v_self->Ca2_JSR, 0);
  __pyx_v_self->Ca2_JSR = __pyx_t_8;
  __pyx_t_8.memview = NULL;
  __pyx_t_8.data = NULL;
```

```
 245:         self.Ca2_NSR = np.full(shape, 1299.5)
```

```
  __pyx_t_6 = __Pyx_GetModuleGlobalName(__pyx_n_s_np); if (unlikely(!__pyx_t_6)) {__pyx_filename = __pyx_f[0]; __pyx_lineno = 245; __pyx_clineno = __LINE__; goto __pyx_L1_error;}
  __Pyx_GOTREF(__pyx_t_6);
  __pyx_t_3 = __Pyx_PyObject_GetAttrStr(__pyx_t_6, __pyx_n_s_full); if (unlikely(!__pyx_t_3)) {__pyx_filename = __pyx_f[0]; __pyx_lineno = 245; __pyx_clineno = __LINE__; goto __pyx_L1_error;}
  __Pyx_GOTREF(__pyx_t_3);
  __Pyx_DECREF(__pyx_t_6); __pyx_t_6 = 0;
  __pyx_t_6 = PyTuple_New(2); if (unlikely(!__pyx_t_6)) {__pyx_filename = __pyx_f[0]; __pyx_lineno = 245; __pyx_clineno = __LINE__; goto __pyx_L1_error;}
  __Pyx_GOTREF(__pyx_t_6);
  __Pyx_INCREF(__pyx_v_shape);
  PyTuple_SET_ITEM(__pyx_t_6, 0, __pyx_v_shape);
  __Pyx_GIVEREF(__pyx_v_shape);
  __Pyx_INCREF(__pyx_float_1299_5);
  PyTuple_SET_ITEM(__pyx_t_6, 1, __pyx_float_1299_5);
  __Pyx_GIVEREF(__pyx_float_1299_5);
  __pyx_t_5 = __Pyx_PyObject_Call(__pyx_t_3, __pyx_t_6, NULL); if (unlikely(!__pyx_t_5)) {__pyx_filename = __pyx_f[0]; __pyx_lineno = 245; __pyx_clineno = __LINE__; goto __pyx_L1_error;}
  __Pyx_GOTREF(__pyx_t_5);
  __Pyx_DECREF(__pyx_t_3); __pyx_t_3 = 0;
  __Pyx_DECREF(__pyx_t_6); __pyx_t_6 = 0;
  __pyx_t_9 = __Pyx_PyObject_to_MemoryviewSlice_d_dc_double(__pyx_t_5);
  if (unlikely(!__pyx_t_9.memview)) {__pyx_filename = __pyx_f[0]; __pyx_lineno = 245; __pyx_clineno = __LINE__; goto __pyx_L1_error;}
  __Pyx_DECREF(__pyx_t_5); __pyx_t_5 = 0;
  __PYX_XDEC_MEMVIEW(&__pyx_v_self->Ca2_NSR, 0);
  __pyx_v_self->Ca2_NSR = __pyx_t_9;
  __pyx_t_9.memview = NULL;
  __pyx_t_9.data = NULL;
```

```
 246:         self.LTRPNCa = np.full(shape, 11.2684)
```

```
  __pyx_t_5 = __Pyx_GetModuleGlobalName(__pyx_n_s_np); if (unlikely(!__pyx_t_5)) {__pyx_filename = __pyx_f[0]; __pyx_lineno = 246; __pyx_clineno = __LINE__; goto __pyx_L1_error;}
  __Pyx_GOTREF(__pyx_t_5);
  __pyx_t_6 = __Pyx_PyObject_GetAttrStr(__pyx_t_5, __pyx_n_s_full); if (unlikely(!__pyx_t_6)) {__pyx_filename = __pyx_f[0]; __pyx_lineno = 246; __pyx_clineno = __LINE__; goto __pyx_L1_error;}
  __Pyx_GOTREF(__pyx_t_6);
  __Pyx_DECREF(__pyx_t_5); __pyx_t_5 = 0;
  __pyx_t_5 = PyTuple_New(2); if (unlikely(!__pyx_t_5)) {__pyx_filename = __pyx_f[0]; __pyx_lineno = 246; __pyx_clineno = __LINE__; goto __pyx_L1_error;}
  __Pyx_GOTREF(__pyx_t_5);
  __Pyx_INCREF(__pyx_v_shape);
  PyTuple_SET_ITEM(__pyx_t_5, 0, __pyx_v_shape);
  __Pyx_GIVEREF(__pyx_v_shape);
  __Pyx_INCREF(__pyx_float_11_2684);
  PyTuple_SET_ITEM(__pyx_t_5, 1, __pyx_float_11_2684);
  __Pyx_GIVEREF(__pyx_float_11_2684);
  __pyx_t_3 = __Pyx_PyObject_Call(__pyx_t_6, __pyx_t_5, NULL); if (unlikely(!__pyx_t_3)) {__pyx_filename = __pyx_f[0]; __pyx_lineno = 246; __pyx_clineno = __LINE__; goto __pyx_L1_error;}
  __Pyx_GOTREF(__pyx_t_3);
  __Pyx_DECREF(__pyx_t_6); __pyx_t_6 = 0;
  __Pyx_DECREF(__pyx_t_5); __pyx_t_5 = 0;
  __pyx_t_10 = __Pyx_PyObject_to_MemoryviewSlice_d_dc_double(__pyx_t_3);
  if (unlikely(!__pyx_t_10.memview)) {__pyx_filename = __pyx_f[0]; __pyx_lineno = 246; __pyx_clineno = __LINE__; goto __pyx_L1_error;}
  __Pyx_DECREF(__pyx_t_3); __pyx_t_3 = 0;
  __PYX_XDEC_MEMVIEW(&__pyx_v_self->LTRPNCa, 0);
  __pyx_v_self->LTRPNCa = __pyx_t_10;
  __pyx_t_10.memview = NULL;
  __pyx_t_10.data = NULL;
```

```
 247:         self.HTRPNCa = np.full(shape, 125.290)
```

```
  __pyx_t_3 = __Pyx_GetModuleGlobalName(__pyx_n_s_np); if (unlikely(!__pyx_t_3)) {__pyx_filename = __pyx_f[0]; __pyx_lineno = 247; __pyx_clineno = __LINE__; goto __pyx_L1_error;}
  __Pyx_GOTREF(__pyx_t_3);
  __pyx_t_5 = __Pyx_PyObject_GetAttrStr(__pyx_t_3, __pyx_n_s_full); if (unlikely(!__pyx_t_5)) {__pyx_filename = __pyx_f[0]; __pyx_lineno = 247; __pyx_clineno = __LINE__; goto __pyx_L1_error;}
  __Pyx_GOTREF(__pyx_t_5);
  __Pyx_DECREF(__pyx_t_3); __pyx_t_3 = 0;
  __pyx_t_3 = PyTuple_New(2); if (unlikely(!__pyx_t_3)) {__pyx_filename = __pyx_f[0]; __pyx_lineno = 247; __pyx_clineno = __LINE__; goto __pyx_L1_error;}
  __Pyx_GOTREF(__pyx_t_3);
  __Pyx_INCREF(__pyx_v_shape);
  PyTuple_SET_ITEM(__pyx_t_3, 0, __pyx_v_shape);
  __Pyx_GIVEREF(__pyx_v_shape);
  __Pyx_INCREF(__pyx_float_125_290);
  PyTuple_SET_ITEM(__pyx_t_3, 1, __pyx_float_125_290);
  __Pyx_GIVEREF(__pyx_float_125_290);
  __pyx_t_6 = __Pyx_PyObject_Call(__pyx_t_5, __pyx_t_3, NULL); if (unlikely(!__pyx_t_6)) {__pyx_filename = __pyx_f[0]; __pyx_lineno = 247; __pyx_clineno = __LINE__; goto __pyx_L1_error;}
  __Pyx_GOTREF(__pyx_t_6);
  __Pyx_DECREF(__pyx_t_5); __pyx_t_5 = 0;
  __Pyx_DECREF(__pyx_t_3); __pyx_t_3 = 0;
  __pyx_t_11 = __Pyx_PyObject_to_MemoryviewSlice_d_dc_double(__pyx_t_6);
  if (unlikely(!__pyx_t_11.memview)) {__pyx_filename = __pyx_f[0]; __pyx_lineno = 247; __pyx_clineno = __LINE__; goto __pyx_L1_error;}
  __Pyx_DECREF(__pyx_t_6); __pyx_t_6 = 0;
  __PYX_XDEC_MEMVIEW(&__pyx_v_self->HTRPNCa, 0);
  __pyx_v_self->HTRPNCa = __pyx_t_11;
  __pyx_t_11.memview = NULL;
  __pyx_t_11.data = NULL;
```

```
 248:         self.O = np.full(shape, 0.930308e-18)
```

```
  __pyx_t_6 = __Pyx_GetModuleGlobalName(__pyx_n_s_np); if (unlikely(!__pyx_t_6)) {__pyx_filename = __pyx_f[0]; __pyx_lineno = 248; __pyx_clineno = __LINE__; goto __pyx_L1_error;}
  __Pyx_GOTREF(__pyx_t_6);
  __pyx_t_3 = __Pyx_PyObject_GetAttrStr(__pyx_t_6, __pyx_n_s_full); if (unlikely(!__pyx_t_3)) {__pyx_filename = __pyx_f[0]; __pyx_lineno = 248; __pyx_clineno = __LINE__; goto __pyx_L1_error;}
  __Pyx_GOTREF(__pyx_t_3);
  __Pyx_DECREF(__pyx_t_6); __pyx_t_6 = 0;
  __pyx_t_6 = PyTuple_New(2); if (unlikely(!__pyx_t_6)) {__pyx_filename = __pyx_f[0]; __pyx_lineno = 248; __pyx_clineno = __LINE__; goto __pyx_L1_error;}
  __Pyx_GOTREF(__pyx_t_6);
  __Pyx_INCREF(__pyx_v_shape);
  PyTuple_SET_ITEM(__pyx_t_6, 0, __pyx_v_shape);
  __Pyx_GIVEREF(__pyx_v_shape);
  __Pyx_INCREF(__pyx_float_0_930308eneg_18);
  PyTuple_SET_ITEM(__pyx_t_6, 1, __pyx_float_0_930308eneg_18);
  __Pyx_GIVEREF(__pyx_float_0_930308eneg_18);
  __pyx_t_5 = __Pyx_PyObject_Call(__pyx_t_3, __pyx_t_6, NULL); if (unlikely(!__pyx_t_5)) {__pyx_filename = __pyx_f[0]; __pyx_lineno = 248; __pyx_clineno = __LINE__; goto __pyx_L1_error;}
  __Pyx_GOTREF(__pyx_t_5);
  __Pyx_DECREF(__pyx_t_3); __pyx_t_3 = 0;
  __Pyx_DECREF(__pyx_t_6); __pyx_t_6 = 0;
  __pyx_t_12 = __Pyx_PyObject_to_MemoryviewSlice_d_dc_double(__pyx_t_5);
  if (unlikely(!__pyx_t_12.memview)) {__pyx_filename = __pyx_f[0]; __pyx_lineno = 248; __pyx_clineno = __LINE__; goto __pyx_L1_error;}
  __Pyx_DECREF(__pyx_t_5); __pyx_t_5 = 0;
  __PYX_XDEC_MEMVIEW(&__pyx_v_self->O, 0);
  __pyx_v_self->O = __pyx_t_12;
  __pyx_t_12.memview = NULL;
  __pyx_t_12.data = NULL;
```

```
 249:         self.C2 = np.full(shape, 0.124216e-3)
```

```
  __pyx_t_5 = __Pyx_GetModuleGlobalName(__pyx_n_s_np); if (unlikely(!__pyx_t_5)) {__pyx_filename = __pyx_f[0]; __pyx_lineno = 249; __pyx_clineno = __LINE__; goto __pyx_L1_error;}
  __Pyx_GOTREF(__pyx_t_5);
  __pyx_t_6 = __Pyx_PyObject_GetAttrStr(__pyx_t_5, __pyx_n_s_full); if (unlikely(!__pyx_t_6)) {__pyx_filename = __pyx_f[0]; __pyx_lineno = 249; __pyx_clineno = __LINE__; goto __pyx_L1_error;}
  __Pyx_GOTREF(__pyx_t_6);
  __Pyx_DECREF(__pyx_t_5); __pyx_t_5 = 0;
  __pyx_t_5 = PyTuple_New(2); if (unlikely(!__pyx_t_5)) {__pyx_filename = __pyx_f[0]; __pyx_lineno = 249; __pyx_clineno = __LINE__; goto __pyx_L1_error;}
  __Pyx_GOTREF(__pyx_t_5);
  __Pyx_INCREF(__pyx_v_shape);
  PyTuple_SET_ITEM(__pyx_t_5, 0, __pyx_v_shape);
  __Pyx_GIVEREF(__pyx_v_shape);
  __Pyx_INCREF(__pyx_float_0_124216eneg_3);
  PyTuple_SET_ITEM(__pyx_t_5, 1, __pyx_float_0_124216eneg_3);
  __Pyx_GIVEREF(__pyx_float_0_124216eneg_3);
  __pyx_t_3 = __Pyx_PyObject_Call(__pyx_t_6, __pyx_t_5, NULL); if (unlikely(!__pyx_t_3)) {__pyx_filename = __pyx_f[0]; __pyx_lineno = 249; __pyx_clineno = __LINE__; goto __pyx_L1_error;}
  __Pyx_GOTREF(__pyx_t_3);
  __Pyx_DECREF(__pyx_t_6); __pyx_t_6 = 0;
  __Pyx_DECREF(__pyx_t_5); __pyx_t_5 = 0;
  __pyx_t_13 = __Pyx_PyObject_to_MemoryviewSlice_d_dc_double(__pyx_t_3);
  if (unlikely(!__pyx_t_13.memview)) {__pyx_filename = __pyx_f[0]; __pyx_lineno = 249; __pyx_clineno = __LINE__; goto __pyx_L1_error;}
  __Pyx_DECREF(__pyx_t_3); __pyx_t_3 = 0;
  __PYX_XDEC_MEMVIEW(&__pyx_v_self->C2, 0);
  __pyx_v_self->C2 = __pyx_t_13;
  __pyx_t_13.memview = NULL;
  __pyx_t_13.data = NULL;
```

```
 250:         self.C3 = np.full(shape, 0.578679e-8)
```

```
  __pyx_t_3 = __Pyx_GetModuleGlobalName(__pyx_n_s_np); if (unlikely(!__pyx_t_3)) {__pyx_filename = __pyx_f[0]; __pyx_lineno = 250; __pyx_clineno = __LINE__; goto __pyx_L1_error;}
  __Pyx_GOTREF(__pyx_t_3);
  __pyx_t_5 = __Pyx_PyObject_GetAttrStr(__pyx_t_3, __pyx_n_s_full); if (unlikely(!__pyx_t_5)) {__pyx_filename = __pyx_f[0]; __pyx_lineno = 250; __pyx_clineno = __LINE__; goto __pyx_L1_error;}
  __Pyx_GOTREF(__pyx_t_5);
  __Pyx_DECREF(__pyx_t_3); __pyx_t_3 = 0;
  __pyx_t_3 = PyTuple_New(2); if (unlikely(!__pyx_t_3)) {__pyx_filename = __pyx_f[0]; __pyx_lineno = 250; __pyx_clineno = __LINE__; goto __pyx_L1_error;}
  __Pyx_GOTREF(__pyx_t_3);
  __Pyx_INCREF(__pyx_v_shape);
  PyTuple_SET_ITEM(__pyx_t_3, 0, __pyx_v_shape);
  __Pyx_GIVEREF(__pyx_v_shape);
  __Pyx_INCREF(__pyx_float_0_578679eneg_8);
  PyTuple_SET_ITEM(__pyx_t_3, 1, __pyx_float_0_578679eneg_8);
  __Pyx_GIVEREF(__pyx_float_0_578679eneg_8);
  __pyx_t_6 = __Pyx_PyObject_Call(__pyx_t_5, __pyx_t_3, NULL); if (unlikely(!__pyx_t_6)) {__pyx_filename = __pyx_f[0]; __pyx_lineno = 250; __pyx_clineno = __LINE__; goto __pyx_L1_error;}
  __Pyx_GOTREF(__pyx_t_6);
  __Pyx_DECREF(__pyx_t_5); __pyx_t_5 = 0;
  __Pyx_DECREF(__pyx_t_3); __pyx_t_3 = 0;
  __pyx_t_14 = __Pyx_PyObject_to_MemoryviewSlice_d_dc_double(__pyx_t_6);
  if (unlikely(!__pyx_t_14.memview)) {__pyx_filename = __pyx_f[0]; __pyx_lineno = 250; __pyx_clineno = __LINE__; goto __pyx_L1_error;}
  __Pyx_DECREF(__pyx_t_6); __pyx_t_6 = 0;
  __PYX_XDEC_MEMVIEW(&__pyx_v_self->C3, 0);
  __pyx_v_self->C3 = __pyx_t_14;
  __pyx_t_14.memview = NULL;
  __pyx_t_14.data = NULL;
```

```
 251:         self.C4 = np.full(shape, 0.119816e-12)
```

```
  __pyx_t_6 = __Pyx_GetModuleGlobalName(__pyx_n_s_np); if (unlikely(!__pyx_t_6)) {__pyx_filename = __pyx_f[0]; __pyx_lineno = 251; __pyx_clineno = __LINE__; goto __pyx_L1_error;}
  __Pyx_GOTREF(__pyx_t_6);
  __pyx_t_3 = __Pyx_PyObject_GetAttrStr(__pyx_t_6, __pyx_n_s_full); if (unlikely(!__pyx_t_3)) {__pyx_filename = __pyx_f[0]; __pyx_lineno = 251; __pyx_clineno = __LINE__; goto __pyx_L1_error;}
  __Pyx_GOTREF(__pyx_t_3);
  __Pyx_DECREF(__pyx_t_6); __pyx_t_6 = 0;
  __pyx_t_6 = PyTuple_New(2); if (unlikely(!__pyx_t_6)) {__pyx_filename = __pyx_f[0]; __pyx_lineno = 251; __pyx_clineno = __LINE__; goto __pyx_L1_error;}
  __Pyx_GOTREF(__pyx_t_6);
  __Pyx_INCREF(__pyx_v_shape);
  PyTuple_SET_ITEM(__pyx_t_6, 0, __pyx_v_shape);
  __Pyx_GIVEREF(__pyx_v_shape);
  __Pyx_INCREF(__pyx_float_0_119816eneg_12);
  PyTuple_SET_ITEM(__pyx_t_6, 1, __pyx_float_0_119816eneg_12);
  __Pyx_GIVEREF(__pyx_float_0_119816eneg_12);
  __pyx_t_5 = __Pyx_PyObject_Call(__pyx_t_3, __pyx_t_6, NULL); if (unlikely(!__pyx_t_5)) {__pyx_filename = __pyx_f[0]; __pyx_lineno = 251; __pyx_clineno = __LINE__; goto __pyx_L1_error;}
  __Pyx_GOTREF(__pyx_t_5);
  __Pyx_DECREF(__pyx_t_3); __pyx_t_3 = 0;
  __Pyx_DECREF(__pyx_t_6); __pyx_t_6 = 0;
  __pyx_t_15 = __Pyx_PyObject_to_MemoryviewSlice_d_dc_double(__pyx_t_5);
  if (unlikely(!__pyx_t_15.memview)) {__pyx_filename = __pyx_f[0]; __pyx_lineno = 251; __pyx_clineno = __LINE__; goto __pyx_L1_error;}
  __Pyx_DECREF(__pyx_t_5); __pyx_t_5 = 0;
  __PYX_XDEC_MEMVIEW(&__pyx_v_self->C4, 0);
  __pyx_v_self->C4 = __pyx_t_15;
  __pyx_t_15.memview = NULL;
  __pyx_t_15.data = NULL;
```

```
 252:         self.I1 = np.full(shape, 0.497923e-18)
```

```
  __pyx_t_5 = __Pyx_GetModuleGlobalName(__pyx_n_s_np); if (unlikely(!__pyx_t_5)) {__pyx_filename = __pyx_f[0]; __pyx_lineno = 252; __pyx_clineno = __LINE__; goto __pyx_L1_error;}
  __Pyx_GOTREF(__pyx_t_5);
  __pyx_t_6 = __Pyx_PyObject_GetAttrStr(__pyx_t_5, __pyx_n_s_full); if (unlikely(!__pyx_t_6)) {__pyx_filename = __pyx_f[0]; __pyx_lineno = 252; __pyx_clineno = __LINE__; goto __pyx_L1_error;}
  __Pyx_GOTREF(__pyx_t_6);
  __Pyx_DECREF(__pyx_t_5); __pyx_t_5 = 0;
  __pyx_t_5 = PyTuple_New(2); if (unlikely(!__pyx_t_5)) {__pyx_filename = __pyx_f[0]; __pyx_lineno = 252; __pyx_clineno = __LINE__; goto __pyx_L1_error;}
  __Pyx_GOTREF(__pyx_t_5);
  __Pyx_INCREF(__pyx_v_shape);
  PyTuple_SET_ITEM(__pyx_t_5, 0, __pyx_v_shape);
  __Pyx_GIVEREF(__pyx_v_shape);
  __Pyx_INCREF(__pyx_float_0_497923eneg_18);
  PyTuple_SET_ITEM(__pyx_t_5, 1, __pyx_float_0_497923eneg_18);
  __Pyx_GIVEREF(__pyx_float_0_497923eneg_18);
  __pyx_t_3 = __Pyx_PyObject_Call(__pyx_t_6, __pyx_t_5, NULL); if (unlikely(!__pyx_t_3)) {__pyx_filename = __pyx_f[0]; __pyx_lineno = 252; __pyx_clineno = __LINE__; goto __pyx_L1_error;}
  __Pyx_GOTREF(__pyx_t_3);
  __Pyx_DECREF(__pyx_t_6); __pyx_t_6 = 0;
  __Pyx_DECREF(__pyx_t_5); __pyx_t_5 = 0;
  __pyx_t_16 = __Pyx_PyObject_to_MemoryviewSlice_d_dc_double(__pyx_t_3);
  if (unlikely(!__pyx_t_16.memview)) {__pyx_filename = __pyx_f[0]; __pyx_lineno = 252; __pyx_clineno = __LINE__; goto __pyx_L1_error;}
  __Pyx_DECREF(__pyx_t_3); __pyx_t_3 = 0;
  __PYX_XDEC_MEMVIEW(&__pyx_v_self->I1, 0);
  __pyx_v_self->I1 = __pyx_t_16;
  __pyx_t_16.memview = NULL;
  __pyx_t_16.data = NULL;
```

```
 253:         self.I2 = np.full(shape, 0.345847e-13)
```

```
  __pyx_t_3 = __Pyx_GetModuleGlobalName(__pyx_n_s_np); if (unlikely(!__pyx_t_3)) {__pyx_filename = __pyx_f[0]; __pyx_lineno = 253; __pyx_clineno = __LINE__; goto __pyx_L1_error;}
  __Pyx_GOTREF(__pyx_t_3);
  __pyx_t_5 = __Pyx_PyObject_GetAttrStr(__pyx_t_3, __pyx_n_s_full); if (unlikely(!__pyx_t_5)) {__pyx_filename = __pyx_f[0]; __pyx_lineno = 253; __pyx_clineno = __LINE__; goto __pyx_L1_error;}
  __Pyx_GOTREF(__pyx_t_5);
  __Pyx_DECREF(__pyx_t_3); __pyx_t_3 = 0;
  __pyx_t_3 = PyTuple_New(2); if (unlikely(!__pyx_t_3)) {__pyx_filename = __pyx_f[0]; __pyx_lineno = 253; __pyx_clineno = __LINE__; goto __pyx_L1_error;}
  __Pyx_GOTREF(__pyx_t_3);
  __Pyx_INCREF(__pyx_v_shape);
  PyTuple_SET_ITEM(__pyx_t_3, 0, __pyx_v_shape);
  __Pyx_GIVEREF(__pyx_v_shape);
  __Pyx_INCREF(__pyx_float_0_345847eneg_13);
  PyTuple_SET_ITEM(__pyx_t_3, 1, __pyx_float_0_345847eneg_13);
  __Pyx_GIVEREF(__pyx_float_0_345847eneg_13);
  __pyx_t_6 = __Pyx_PyObject_Call(__pyx_t_5, __pyx_t_3, NULL); if (unlikely(!__pyx_t_6)) {__pyx_filename = __pyx_f[0]; __pyx_lineno = 253; __pyx_clineno = __LINE__; goto __pyx_L1_error;}
  __Pyx_GOTREF(__pyx_t_6);
  __Pyx_DECREF(__pyx_t_5); __pyx_t_5 = 0;
  __Pyx_DECREF(__pyx_t_3); __pyx_t_3 = 0;
  __pyx_t_17 = __Pyx_PyObject_to_MemoryviewSlice_d_dc_double(__pyx_t_6);
  if (unlikely(!__pyx_t_17.memview)) {__pyx_filename = __pyx_f[0]; __pyx_lineno = 253; __pyx_clineno = __LINE__; goto __pyx_L1_error;}
  __Pyx_DECREF(__pyx_t_6); __pyx_t_6 = 0;
  __PYX_XDEC_MEMVIEW(&__pyx_v_self->I2, 0);
  __pyx_v_self->I2 = __pyx_t_17;
  __pyx_t_17.memview = NULL;
  __pyx_t_17.data = NULL;
```

```
 254:         self.I3 = np.full(shape, 0.185106e-13)
```

```
  __pyx_t_6 = __Pyx_GetModuleGlobalName(__pyx_n_s_np); if (unlikely(!__pyx_t_6)) {__pyx_filename = __pyx_f[0]; __pyx_lineno = 254; __pyx_clineno = __LINE__; goto __pyx_L1_error;}
  __Pyx_GOTREF(__pyx_t_6);
  __pyx_t_3 = __Pyx_PyObject_GetAttrStr(__pyx_t_6, __pyx_n_s_full); if (unlikely(!__pyx_t_3)) {__pyx_filename = __pyx_f[0]; __pyx_lineno = 254; __pyx_clineno = __LINE__; goto __pyx_L1_error;}
  __Pyx_GOTREF(__pyx_t_3);
  __Pyx_DECREF(__pyx_t_6); __pyx_t_6 = 0;
  __pyx_t_6 = PyTuple_New(2); if (unlikely(!__pyx_t_6)) {__pyx_filename = __pyx_f[0]; __pyx_lineno = 254; __pyx_clineno = __LINE__; goto __pyx_L1_error;}
  __Pyx_GOTREF(__pyx_t_6);
  __Pyx_INCREF(__pyx_v_shape);
  PyTuple_SET_ITEM(__pyx_t_6, 0, __pyx_v_shape);
  __Pyx_GIVEREF(__pyx_v_shape);
  __Pyx_INCREF(__pyx_float_0_185106eneg_13);
  PyTuple_SET_ITEM(__pyx_t_6, 1, __pyx_float_0_185106eneg_13);
  __Pyx_GIVEREF(__pyx_float_0_185106eneg_13);
  __pyx_t_5 = __Pyx_PyObject_Call(__pyx_t_3, __pyx_t_6, NULL); if (unlikely(!__pyx_t_5)) {__pyx_filename = __pyx_f[0]; __pyx_lineno = 254; __pyx_clineno = __LINE__; goto __pyx_L1_error;}
  __Pyx_GOTREF(__pyx_t_5);
  __Pyx_DECREF(__pyx_t_3); __pyx_t_3 = 0;
  __Pyx_DECREF(__pyx_t_6); __pyx_t_6 = 0;
  __pyx_t_18 = __Pyx_PyObject_to_MemoryviewSlice_d_dc_double(__pyx_t_5);
  if (unlikely(!__pyx_t_18.memview)) {__pyx_filename = __pyx_f[0]; __pyx_lineno = 254; __pyx_clineno = __LINE__; goto __pyx_L1_error;}
  __Pyx_DECREF(__pyx_t_5); __pyx_t_5 = 0;
  __PYX_XDEC_MEMVIEW(&__pyx_v_self->I3, 0);
  __pyx_v_self->I3 = __pyx_t_18;
  __pyx_t_18.memview = NULL;
  __pyx_t_18.data = NULL;
```

```
 255:         self.Pc2 = np.full(shape, 0.167740e-3)
```

```
  __pyx_t_5 = __Pyx_GetModuleGlobalName(__pyx_n_s_np); if (unlikely(!__pyx_t_5)) {__pyx_filename = __pyx_f[0]; __pyx_lineno = 255; __pyx_clineno = __LINE__; goto __pyx_L1_error;}
  __Pyx_GOTREF(__pyx_t_5);
  __pyx_t_6 = __Pyx_PyObject_GetAttrStr(__pyx_t_5, __pyx_n_s_full); if (unlikely(!__pyx_t_6)) {__pyx_filename = __pyx_f[0]; __pyx_lineno = 255; __pyx_clineno = __LINE__; goto __pyx_L1_error;}
  __Pyx_GOTREF(__pyx_t_6);
  __Pyx_DECREF(__pyx_t_5); __pyx_t_5 = 0;
  __pyx_t_5 = PyTuple_New(2); if (unlikely(!__pyx_t_5)) {__pyx_filename = __pyx_f[0]; __pyx_lineno = 255; __pyx_clineno = __LINE__; goto __pyx_L1_error;}
  __Pyx_GOTREF(__pyx_t_5);
  __Pyx_INCREF(__pyx_v_shape);
  PyTuple_SET_ITEM(__pyx_t_5, 0, __pyx_v_shape);
  __Pyx_GIVEREF(__pyx_v_shape);
  __Pyx_INCREF(__pyx_float_0_167740eneg_3);
  PyTuple_SET_ITEM(__pyx_t_5, 1, __pyx_float_0_167740eneg_3);
  __Pyx_GIVEREF(__pyx_float_0_167740eneg_3);
  __pyx_t_3 = __Pyx_PyObject_Call(__pyx_t_6, __pyx_t_5, NULL); if (unlikely(!__pyx_t_3)) {__pyx_filename = __pyx_f[0]; __pyx_lineno = 255; __pyx_clineno = __LINE__; goto __pyx_L1_error;}
  __Pyx_GOTREF(__pyx_t_3);
  __Pyx_DECREF(__pyx_t_6); __pyx_t_6 = 0;
  __Pyx_DECREF(__pyx_t_5); __pyx_t_5 = 0;
  __pyx_t_19 = __Pyx_PyObject_to_MemoryviewSlice_d_dc_double(__pyx_t_3);
  if (unlikely(!__pyx_t_19.memview)) {__pyx_filename = __pyx_f[0]; __pyx_lineno = 255; __pyx_clineno = __LINE__; goto __pyx_L1_error;}
  __Pyx_DECREF(__pyx_t_3); __pyx_t_3 = 0;
  __PYX_XDEC_MEMVIEW(&__pyx_v_self->Pc2, 0);
  __pyx_v_self->Pc2 = __pyx_t_19;
  __pyx_t_19.memview = NULL;
  __pyx_t_19.data = NULL;
```

```
 256:         self.Po1 = np.full(shape, 0.149102e-4)
```

```
  __pyx_t_3 = __Pyx_GetModuleGlobalName(__pyx_n_s_np); if (unlikely(!__pyx_t_3)) {__pyx_filename = __pyx_f[0]; __pyx_lineno = 256; __pyx_clineno = __LINE__; goto __pyx_L1_error;}
  __Pyx_GOTREF(__pyx_t_3);
  __pyx_t_5 = __Pyx_PyObject_GetAttrStr(__pyx_t_3, __pyx_n_s_full); if (unlikely(!__pyx_t_5)) {__pyx_filename = __pyx_f[0]; __pyx_lineno = 256; __pyx_clineno = __LINE__; goto __pyx_L1_error;}
  __Pyx_GOTREF(__pyx_t_5);
  __Pyx_DECREF(__pyx_t_3); __pyx_t_3 = 0;
  __pyx_t_3 = PyTuple_New(2); if (unlikely(!__pyx_t_3)) {__pyx_filename = __pyx_f[0]; __pyx_lineno = 256; __pyx_clineno = __LINE__; goto __pyx_L1_error;}
  __Pyx_GOTREF(__pyx_t_3);
  __Pyx_INCREF(__pyx_v_shape);
  PyTuple_SET_ITEM(__pyx_t_3, 0, __pyx_v_shape);
  __Pyx_GIVEREF(__pyx_v_shape);
  __Pyx_INCREF(__pyx_float_0_149102eneg_4);
  PyTuple_SET_ITEM(__pyx_t_3, 1, __pyx_float_0_149102eneg_4);
  __Pyx_GIVEREF(__pyx_float_0_149102eneg_4);
  __pyx_t_6 = __Pyx_PyObject_Call(__pyx_t_5, __pyx_t_3, NULL); if (unlikely(!__pyx_t_6)) {__pyx_filename = __pyx_f[0]; __pyx_lineno = 256; __pyx_clineno = __LINE__; goto __pyx_L1_error;}
  __Pyx_GOTREF(__pyx_t_6);
  __Pyx_DECREF(__pyx_t_5); __pyx_t_5 = 0;
  __Pyx_DECREF(__pyx_t_3); __pyx_t_3 = 0;
  __pyx_t_20 = __Pyx_PyObject_to_MemoryviewSlice_d_dc_double(__pyx_t_6);
  if (unlikely(!__pyx_t_20.memview)) {__pyx_filename = __pyx_f[0]; __pyx_lineno = 256; __pyx_clineno = __LINE__; goto __pyx_L1_error;}
  __Pyx_DECREF(__pyx_t_6); __pyx_t_6 = 0;
  __PYX_XDEC_MEMVIEW(&__pyx_v_self->Po1, 0);
  __pyx_v_self->Po1 = __pyx_t_20;
  __pyx_t_20.memview = NULL;
  __pyx_t_20.data = NULL;
```

```
 257:         self.Po2 = np.full(shape, 0.951726e-10)
```

```
  __pyx_t_6 = __Pyx_GetModuleGlobalName(__pyx_n_s_np); if (unlikely(!__pyx_t_6)) {__pyx_filename = __pyx_f[0]; __pyx_lineno = 257; __pyx_clineno = __LINE__; goto __pyx_L1_error;}
  __Pyx_GOTREF(__pyx_t_6);
  __pyx_t_3 = __Pyx_PyObject_GetAttrStr(__pyx_t_6, __pyx_n_s_full); if (unlikely(!__pyx_t_3)) {__pyx_filename = __pyx_f[0]; __pyx_lineno = 257; __pyx_clineno = __LINE__; goto __pyx_L1_error;}
  __Pyx_GOTREF(__pyx_t_3);
  __Pyx_DECREF(__pyx_t_6); __pyx_t_6 = 0;
  __pyx_t_6 = PyTuple_New(2); if (unlikely(!__pyx_t_6)) {__pyx_filename = __pyx_f[0]; __pyx_lineno = 257; __pyx_clineno = __LINE__; goto __pyx_L1_error;}
  __Pyx_GOTREF(__pyx_t_6);
  __Pyx_INCREF(__pyx_v_shape);
  PyTuple_SET_ITEM(__pyx_t_6, 0, __pyx_v_shape);
  __Pyx_GIVEREF(__pyx_v_shape);
  __Pyx_INCREF(__pyx_float_0_951726eneg_10);
  PyTuple_SET_ITEM(__pyx_t_6, 1, __pyx_float_0_951726eneg_10);
  __Pyx_GIVEREF(__pyx_float_0_951726eneg_10);
  __pyx_t_5 = __Pyx_PyObject_Call(__pyx_t_3, __pyx_t_6, NULL); if (unlikely(!__pyx_t_5)) {__pyx_filename = __pyx_f[0]; __pyx_lineno = 257; __pyx_clineno = __LINE__; goto __pyx_L1_error;}
  __Pyx_GOTREF(__pyx_t_5);
  __Pyx_DECREF(__pyx_t_3); __pyx_t_3 = 0;
  __Pyx_DECREF(__pyx_t_6); __pyx_t_6 = 0;
  __pyx_t_21 = __Pyx_PyObject_to_MemoryviewSlice_d_dc_double(__pyx_t_5);
  if (unlikely(!__pyx_t_21.memview)) {__pyx_filename = __pyx_f[0]; __pyx_lineno = 257; __pyx_clineno = __LINE__; goto __pyx_L1_error;}
  __Pyx_DECREF(__pyx_t_5); __pyx_t_5 = 0;
  __PYX_XDEC_MEMVIEW(&__pyx_v_self->Po2, 0);
  __pyx_v_self->Po2 = __pyx_t_21;
  __pyx_t_21.memview = NULL;
  __pyx_t_21.data = NULL;
```

```
 258:         self.P_RyR = np.full(shape, 0.0)
```

```
  __pyx_t_5 = __Pyx_GetModuleGlobalName(__pyx_n_s_np); if (unlikely(!__pyx_t_5)) {__pyx_filename = __pyx_f[0]; __pyx_lineno = 258; __pyx_clineno = __LINE__; goto __pyx_L1_error;}
  __Pyx_GOTREF(__pyx_t_5);
  __pyx_t_6 = __Pyx_PyObject_GetAttrStr(__pyx_t_5, __pyx_n_s_full); if (unlikely(!__pyx_t_6)) {__pyx_filename = __pyx_f[0]; __pyx_lineno = 258; __pyx_clineno = __LINE__; goto __pyx_L1_error;}
  __Pyx_GOTREF(__pyx_t_6);
  __Pyx_DECREF(__pyx_t_5); __pyx_t_5 = 0;
  __pyx_t_5 = PyTuple_New(2); if (unlikely(!__pyx_t_5)) {__pyx_filename = __pyx_f[0]; __pyx_lineno = 258; __pyx_clineno = __LINE__; goto __pyx_L1_error;}
  __Pyx_GOTREF(__pyx_t_5);
  __Pyx_INCREF(__pyx_v_shape);
  PyTuple_SET_ITEM(__pyx_t_5, 0, __pyx_v_shape);
  __Pyx_GIVEREF(__pyx_v_shape);
  __Pyx_INCREF(__pyx_float_0_0);
  PyTuple_SET_ITEM(__pyx_t_5, 1, __pyx_float_0_0);
  __Pyx_GIVEREF(__pyx_float_0_0);
  __pyx_t_3 = __Pyx_PyObject_Call(__pyx_t_6, __pyx_t_5, NULL); if (unlikely(!__pyx_t_3)) {__pyx_filename = __pyx_f[0]; __pyx_lineno = 258; __pyx_clineno = __LINE__; goto __pyx_L1_error;}
  __Pyx_GOTREF(__pyx_t_3);
  __Pyx_DECREF(__pyx_t_6); __pyx_t_6 = 0;
  __Pyx_DECREF(__pyx_t_5); __pyx_t_5 = 0;
  __pyx_t_22 = __Pyx_PyObject_to_MemoryviewSlice_d_dc_double(__pyx_t_3);
  if (unlikely(!__pyx_t_22.memview)) {__pyx_filename = __pyx_f[0]; __pyx_lineno = 258; __pyx_clineno = __LINE__; goto __pyx_L1_error;}
  __Pyx_DECREF(__pyx_t_3); __pyx_t_3 = 0;
  __PYX_XDEC_MEMVIEW(&__pyx_v_self->P_RyR, 0);
  __pyx_v_self->P_RyR = __pyx_t_22;
  __pyx_t_22.memview = NULL;
  __pyx_t_22.data = NULL;
```

```
 259:         self.C_Na2 = np.full(shape, 0.020752)
```

```
  __pyx_t_3 = __Pyx_GetModuleGlobalName(__pyx_n_s_np); if (unlikely(!__pyx_t_3)) {__pyx_filename = __pyx_f[0]; __pyx_lineno = 259; __pyx_clineno = __LINE__; goto __pyx_L1_error;}
  __Pyx_GOTREF(__pyx_t_3);
  __pyx_t_5 = __Pyx_PyObject_GetAttrStr(__pyx_t_3, __pyx_n_s_full); if (unlikely(!__pyx_t_5)) {__pyx_filename = __pyx_f[0]; __pyx_lineno = 259; __pyx_clineno = __LINE__; goto __pyx_L1_error;}
  __Pyx_GOTREF(__pyx_t_5);
  __Pyx_DECREF(__pyx_t_3); __pyx_t_3 = 0;
  __pyx_t_3 = PyTuple_New(2); if (unlikely(!__pyx_t_3)) {__pyx_filename = __pyx_f[0]; __pyx_lineno = 259; __pyx_clineno = __LINE__; goto __pyx_L1_error;}
  __Pyx_GOTREF(__pyx_t_3);
  __Pyx_INCREF(__pyx_v_shape);
  PyTuple_SET_ITEM(__pyx_t_3, 0, __pyx_v_shape);
  __Pyx_GIVEREF(__pyx_v_shape);
  __Pyx_INCREF(__pyx_float_0_020752);
  PyTuple_SET_ITEM(__pyx_t_3, 1, __pyx_float_0_020752);
  __Pyx_GIVEREF(__pyx_float_0_020752);
  __pyx_t_6 = __Pyx_PyObject_Call(__pyx_t_5, __pyx_t_3, NULL); if (unlikely(!__pyx_t_6)) {__pyx_filename = __pyx_f[0]; __pyx_lineno = 259; __pyx_clineno = __LINE__; goto __pyx_L1_error;}
  __Pyx_GOTREF(__pyx_t_6);
  __Pyx_DECREF(__pyx_t_5); __pyx_t_5 = 0;
  __Pyx_DECREF(__pyx_t_3); __pyx_t_3 = 0;
  __pyx_t_23 = __Pyx_PyObject_to_MemoryviewSlice_d_dc_double(__pyx_t_6);
  if (unlikely(!__pyx_t_23.memview)) {__pyx_filename = __pyx_f[0]; __pyx_lineno = 259; __pyx_clineno = __LINE__; goto __pyx_L1_error;}
  __Pyx_DECREF(__pyx_t_6); __pyx_t_6 = 0;
  __PYX_XDEC_MEMVIEW(&__pyx_v_self->C_Na2, 0);
  __pyx_v_self->C_Na2 = __pyx_t_23;
  __pyx_t_23.memview = NULL;
  __pyx_t_23.data = NULL;
```

```
 260:         self.C_Na1 = np.full(shape, 0.279132e-3)
```

```
  __pyx_t_6 = __Pyx_GetModuleGlobalName(__pyx_n_s_np); if (unlikely(!__pyx_t_6)) {__pyx_filename = __pyx_f[0]; __pyx_lineno = 260; __pyx_clineno = __LINE__; goto __pyx_L1_error;}
  __Pyx_GOTREF(__pyx_t_6);
  __pyx_t_3 = __Pyx_PyObject_GetAttrStr(__pyx_t_6, __pyx_n_s_full); if (unlikely(!__pyx_t_3)) {__pyx_filename = __pyx_f[0]; __pyx_lineno = 260; __pyx_clineno = __LINE__; goto __pyx_L1_error;}
  __Pyx_GOTREF(__pyx_t_3);
  __Pyx_DECREF(__pyx_t_6); __pyx_t_6 = 0;
  __pyx_t_6 = PyTuple_New(2); if (unlikely(!__pyx_t_6)) {__pyx_filename = __pyx_f[0]; __pyx_lineno = 260; __pyx_clineno = __LINE__; goto __pyx_L1_error;}
  __Pyx_GOTREF(__pyx_t_6);
  __Pyx_INCREF(__pyx_v_shape);
  PyTuple_SET_ITEM(__pyx_t_6, 0, __pyx_v_shape);
  __Pyx_GIVEREF(__pyx_v_shape);
  __Pyx_INCREF(__pyx_float_0_279132eneg_3);
  PyTuple_SET_ITEM(__pyx_t_6, 1, __pyx_float_0_279132eneg_3);
  __Pyx_GIVEREF(__pyx_float_0_279132eneg_3);
  __pyx_t_5 = __Pyx_PyObject_Call(__pyx_t_3, __pyx_t_6, NULL); if (unlikely(!__pyx_t_5)) {__pyx_filename = __pyx_f[0]; __pyx_lineno = 260; __pyx_clineno = __LINE__; goto __pyx_L1_error;}
  __Pyx_GOTREF(__pyx_t_5);
  __Pyx_DECREF(__pyx_t_3); __pyx_t_3 = 0;
  __Pyx_DECREF(__pyx_t_6); __pyx_t_6 = 0;
  __pyx_t_24 = __Pyx_PyObject_to_MemoryviewSlice_d_dc_double(__pyx_t_5);
  if (unlikely(!__pyx_t_24.memview)) {__pyx_filename = __pyx_f[0]; __pyx_lineno = 260; __pyx_clineno = __LINE__; goto __pyx_L1_error;}
  __Pyx_DECREF(__pyx_t_5); __pyx_t_5 = 0;
  __PYX_XDEC_MEMVIEW(&__pyx_v_self->C_Na1, 0);
  __pyx_v_self->C_Na1 = __pyx_t_24;
  __pyx_t_24.memview = NULL;
  __pyx_t_24.data = NULL;
```

```
 261:         self.O_Na = np.full(shape, 0.713483e-6)
```

```
  __pyx_t_5 = __Pyx_GetModuleGlobalName(__pyx_n_s_np); if (unlikely(!__pyx_t_5)) {__pyx_filename = __pyx_f[0]; __pyx_lineno = 261; __pyx_clineno = __LINE__; goto __pyx_L1_error;}
  __Pyx_GOTREF(__pyx_t_5);
  __pyx_t_6 = __Pyx_PyObject_GetAttrStr(__pyx_t_5, __pyx_n_s_full); if (unlikely(!__pyx_t_6)) {__pyx_filename = __pyx_f[0]; __pyx_lineno = 261; __pyx_clineno = __LINE__; goto __pyx_L1_error;}
  __Pyx_GOTREF(__pyx_t_6);
  __Pyx_DECREF(__pyx_t_5); __pyx_t_5 = 0;
  __pyx_t_5 = PyTuple_New(2); if (unlikely(!__pyx_t_5)) {__pyx_filename = __pyx_f[0]; __pyx_lineno = 261; __pyx_clineno = __LINE__; goto __pyx_L1_error;}
  __Pyx_GOTREF(__pyx_t_5);
  __Pyx_INCREF(__pyx_v_shape);
  PyTuple_SET_ITEM(__pyx_t_5, 0, __pyx_v_shape);
  __Pyx_GIVEREF(__pyx_v_shape);
  __Pyx_INCREF(__pyx_float_0_713483eneg_6);
  PyTuple_SET_ITEM(__pyx_t_5, 1, __pyx_float_0_713483eneg_6);
  __Pyx_GIVEREF(__pyx_float_0_713483eneg_6);
  __pyx_t_3 = __Pyx_PyObject_Call(__pyx_t_6, __pyx_t_5, NULL); if (unlikely(!__pyx_t_3)) {__pyx_filename = __pyx_f[0]; __pyx_lineno = 261; __pyx_clineno = __LINE__; goto __pyx_L1_error;}
  __Pyx_GOTREF(__pyx_t_3);
  __Pyx_DECREF(__pyx_t_6); __pyx_t_6 = 0;
  __Pyx_DECREF(__pyx_t_5); __pyx_t_5 = 0;
  __pyx_t_25 = __Pyx_PyObject_to_MemoryviewSlice_d_dc_double(__pyx_t_3);
  if (unlikely(!__pyx_t_25.memview)) {__pyx_filename = __pyx_f[0]; __pyx_lineno = 261; __pyx_clineno = __LINE__; goto __pyx_L1_error;}
  __Pyx_DECREF(__pyx_t_3); __pyx_t_3 = 0;
  __PYX_XDEC_MEMVIEW(&__pyx_v_self->O_Na, 0);
  __pyx_v_self->O_Na = __pyx_t_25;
  __pyx_t_25.memview = NULL;
  __pyx_t_25.data = NULL;
```

```
 262:         self.IF_Na = np.full(shape, 0.153176e-3)
```

```
  __pyx_t_3 = __Pyx_GetModuleGlobalName(__pyx_n_s_np); if (unlikely(!__pyx_t_3)) {__pyx_filename = __pyx_f[0]; __pyx_lineno = 262; __pyx_clineno = __LINE__; goto __pyx_L1_error;}
  __Pyx_GOTREF(__pyx_t_3);
  __pyx_t_5 = __Pyx_PyObject_GetAttrStr(__pyx_t_3, __pyx_n_s_full); if (unlikely(!__pyx_t_5)) {__pyx_filename = __pyx_f[0]; __pyx_lineno = 262; __pyx_clineno = __LINE__; goto __pyx_L1_error;}
  __Pyx_GOTREF(__pyx_t_5);
  __Pyx_DECREF(__pyx_t_3); __pyx_t_3 = 0;
  __pyx_t_3 = PyTuple_New(2); if (unlikely(!__pyx_t_3)) {__pyx_filename = __pyx_f[0]; __pyx_lineno = 262; __pyx_clineno = __LINE__; goto __pyx_L1_error;}
  __Pyx_GOTREF(__pyx_t_3);
  __Pyx_INCREF(__pyx_v_shape);
  PyTuple_SET_ITEM(__pyx_t_3, 0, __pyx_v_shape);
  __Pyx_GIVEREF(__pyx_v_shape);
  __Pyx_INCREF(__pyx_float_0_153176eneg_3);
  PyTuple_SET_ITEM(__pyx_t_3, 1, __pyx_float_0_153176eneg_3);
  __Pyx_GIVEREF(__pyx_float_0_153176eneg_3);
  __pyx_t_6 = __Pyx_PyObject_Call(__pyx_t_5, __pyx_t_3, NULL); if (unlikely(!__pyx_t_6)) {__pyx_filename = __pyx_f[0]; __pyx_lineno = 262; __pyx_clineno = __LINE__; goto __pyx_L1_error;}
  __Pyx_GOTREF(__pyx_t_6);
  __Pyx_DECREF(__pyx_t_5); __pyx_t_5 = 0;
  __Pyx_DECREF(__pyx_t_3); __pyx_t_3 = 0;
  __pyx_t_26 = __Pyx_PyObject_to_MemoryviewSlice_d_dc_double(__pyx_t_6);
  if (unlikely(!__pyx_t_26.memview)) {__pyx_filename = __pyx_f[0]; __pyx_lineno = 262; __pyx_clineno = __LINE__; goto __pyx_L1_error;}
  __Pyx_DECREF(__pyx_t_6); __pyx_t_6 = 0;
  __PYX_XDEC_MEMVIEW(&__pyx_v_self->IF_Na, 0);
  __pyx_v_self->IF_Na = __pyx_t_26;
  __pyx_t_26.memview = NULL;
  __pyx_t_26.data = NULL;
```

```
 263:         self.I1_Na = np.full(shape, 0.673345e-6)
```

```
  __pyx_t_6 = __Pyx_GetModuleGlobalName(__pyx_n_s_np); if (unlikely(!__pyx_t_6)) {__pyx_filename = __pyx_f[0]; __pyx_lineno = 263; __pyx_clineno = __LINE__; goto __pyx_L1_error;}
  __Pyx_GOTREF(__pyx_t_6);
  __pyx_t_3 = __Pyx_PyObject_GetAttrStr(__pyx_t_6, __pyx_n_s_full); if (unlikely(!__pyx_t_3)) {__pyx_filename = __pyx_f[0]; __pyx_lineno = 263; __pyx_clineno = __LINE__; goto __pyx_L1_error;}
  __Pyx_GOTREF(__pyx_t_3);
  __Pyx_DECREF(__pyx_t_6); __pyx_t_6 = 0;
  __pyx_t_6 = PyTuple_New(2); if (unlikely(!__pyx_t_6)) {__pyx_filename = __pyx_f[0]; __pyx_lineno = 263; __pyx_clineno = __LINE__; goto __pyx_L1_error;}
  __Pyx_GOTREF(__pyx_t_6);
  __Pyx_INCREF(__pyx_v_shape);
  PyTuple_SET_ITEM(__pyx_t_6, 0, __pyx_v_shape);
  __Pyx_GIVEREF(__pyx_v_shape);
  __Pyx_INCREF(__pyx_float_0_673345eneg_6);
  PyTuple_SET_ITEM(__pyx_t_6, 1, __pyx_float_0_673345eneg_6);
  __Pyx_GIVEREF(__pyx_float_0_673345eneg_6);
  __pyx_t_5 = __Pyx_PyObject_Call(__pyx_t_3, __pyx_t_6, NULL); if (unlikely(!__pyx_t_5)) {__pyx_filename = __pyx_f[0]; __pyx_lineno = 263; __pyx_clineno = __LINE__; goto __pyx_L1_error;}
  __Pyx_GOTREF(__pyx_t_5);
  __Pyx_DECREF(__pyx_t_3); __pyx_t_3 = 0;
  __Pyx_DECREF(__pyx_t_6); __pyx_t_6 = 0;
  __pyx_t_27 = __Pyx_PyObject_to_MemoryviewSlice_d_dc_double(__pyx_t_5);
  if (unlikely(!__pyx_t_27.memview)) {__pyx_filename = __pyx_f[0]; __pyx_lineno = 263; __pyx_clineno = __LINE__; goto __pyx_L1_error;}
  __Pyx_DECREF(__pyx_t_5); __pyx_t_5 = 0;
  __PYX_XDEC_MEMVIEW(&__pyx_v_self->I1_Na, 0);
  __pyx_v_self->I1_Na = __pyx_t_27;
  __pyx_t_27.memview = NULL;
  __pyx_t_27.data = NULL;
```

```
 264:         self.I2_Na = np.full(shape, 0.155787e-8)
```

```
  __pyx_t_5 = __Pyx_GetModuleGlobalName(__pyx_n_s_np); if (unlikely(!__pyx_t_5)) {__pyx_filename = __pyx_f[0]; __pyx_lineno = 264; __pyx_clineno = __LINE__; goto __pyx_L1_error;}
  __Pyx_GOTREF(__pyx_t_5);
  __pyx_t_6 = __Pyx_PyObject_GetAttrStr(__pyx_t_5, __pyx_n_s_full); if (unlikely(!__pyx_t_6)) {__pyx_filename = __pyx_f[0]; __pyx_lineno = 264; __pyx_clineno = __LINE__; goto __pyx_L1_error;}
  __Pyx_GOTREF(__pyx_t_6);
  __Pyx_DECREF(__pyx_t_5); __pyx_t_5 = 0;
  __pyx_t_5 = PyTuple_New(2); if (unlikely(!__pyx_t_5)) {__pyx_filename = __pyx_f[0]; __pyx_lineno = 264; __pyx_clineno = __LINE__; goto __pyx_L1_error;}
  __Pyx_GOTREF(__pyx_t_5);
  __Pyx_INCREF(__pyx_v_shape);
  PyTuple_SET_ITEM(__pyx_t_5, 0, __pyx_v_shape);
  __Pyx_GIVEREF(__pyx_v_shape);
  __Pyx_INCREF(__pyx_float_0_155787eneg_8);
  PyTuple_SET_ITEM(__pyx_t_5, 1, __pyx_float_0_155787eneg_8);
  __Pyx_GIVEREF(__pyx_float_0_155787eneg_8);
  __pyx_t_3 = __Pyx_PyObject_Call(__pyx_t_6, __pyx_t_5, NULL); if (unlikely(!__pyx_t_3)) {__pyx_filename = __pyx_f[0]; __pyx_lineno = 264; __pyx_clineno = __LINE__; goto __pyx_L1_error;}
  __Pyx_GOTREF(__pyx_t_3);
  __Pyx_DECREF(__pyx_t_6); __pyx_t_6 = 0;
  __Pyx_DECREF(__pyx_t_5); __pyx_t_5 = 0;
  __pyx_t_28 = __Pyx_PyObject_to_MemoryviewSlice_d_dc_double(__pyx_t_3);
  if (unlikely(!__pyx_t_28.memview)) {__pyx_filename = __pyx_f[0]; __pyx_lineno = 264; __pyx_clineno = __LINE__; goto __pyx_L1_error;}
  __Pyx_DECREF(__pyx_t_3); __pyx_t_3 = 0;
  __PYX_XDEC_MEMVIEW(&__pyx_v_self->I2_Na, 0);
  __pyx_v_self->I2_Na = __pyx_t_28;
  __pyx_t_28.memview = NULL;
  __pyx_t_28.data = NULL;
```

```
 265:         self.IC_Na2 = np.full(shape, 0.0113879)
```

```
  __pyx_t_3 = __Pyx_GetModuleGlobalName(__pyx_n_s_np); if (unlikely(!__pyx_t_3)) {__pyx_filename = __pyx_f[0]; __pyx_lineno = 265; __pyx_clineno = __LINE__; goto __pyx_L1_error;}
  __Pyx_GOTREF(__pyx_t_3);
  __pyx_t_5 = __Pyx_PyObject_GetAttrStr(__pyx_t_3, __pyx_n_s_full); if (unlikely(!__pyx_t_5)) {__pyx_filename = __pyx_f[0]; __pyx_lineno = 265; __pyx_clineno = __LINE__; goto __pyx_L1_error;}
  __Pyx_GOTREF(__pyx_t_5);
  __Pyx_DECREF(__pyx_t_3); __pyx_t_3 = 0;
  __pyx_t_3 = PyTuple_New(2); if (unlikely(!__pyx_t_3)) {__pyx_filename = __pyx_f[0]; __pyx_lineno = 265; __pyx_clineno = __LINE__; goto __pyx_L1_error;}
  __Pyx_GOTREF(__pyx_t_3);
  __Pyx_INCREF(__pyx_v_shape);
  PyTuple_SET_ITEM(__pyx_t_3, 0, __pyx_v_shape);
  __Pyx_GIVEREF(__pyx_v_shape);
  __Pyx_INCREF(__pyx_float_0_0113879);
  PyTuple_SET_ITEM(__pyx_t_3, 1, __pyx_float_0_0113879);
  __Pyx_GIVEREF(__pyx_float_0_0113879);
  __pyx_t_6 = __Pyx_PyObject_Call(__pyx_t_5, __pyx_t_3, NULL); if (unlikely(!__pyx_t_6)) {__pyx_filename = __pyx_f[0]; __pyx_lineno = 265; __pyx_clineno = __LINE__; goto __pyx_L1_error;}
  __Pyx_GOTREF(__pyx_t_6);
  __Pyx_DECREF(__pyx_t_5); __pyx_t_5 = 0;
  __Pyx_DECREF(__pyx_t_3); __pyx_t_3 = 0;
  __pyx_t_29 = __Pyx_PyObject_to_MemoryviewSlice_d_dc_double(__pyx_t_6);
  if (unlikely(!__pyx_t_29.memview)) {__pyx_filename = __pyx_f[0]; __pyx_lineno = 265; __pyx_clineno = __LINE__; goto __pyx_L1_error;}
  __Pyx_DECREF(__pyx_t_6); __pyx_t_6 = 0;
  __PYX_XDEC_MEMVIEW(&__pyx_v_self->IC_Na2, 0);
  __pyx_v_self->IC_Na2 = __pyx_t_29;
  __pyx_t_29.memview = NULL;
  __pyx_t_29.data = NULL;
```

```
 266:         self.IC_Na3 = np.full(shape, 0.342780)
```

```
  __pyx_t_6 = __Pyx_GetModuleGlobalName(__pyx_n_s_np); if (unlikely(!__pyx_t_6)) {__pyx_filename = __pyx_f[0]; __pyx_lineno = 266; __pyx_clineno = __LINE__; goto __pyx_L1_error;}
  __Pyx_GOTREF(__pyx_t_6);
  __pyx_t_3 = __Pyx_PyObject_GetAttrStr(__pyx_t_6, __pyx_n_s_full); if (unlikely(!__pyx_t_3)) {__pyx_filename = __pyx_f[0]; __pyx_lineno = 266; __pyx_clineno = __LINE__; goto __pyx_L1_error;}
  __Pyx_GOTREF(__pyx_t_3);
  __Pyx_DECREF(__pyx_t_6); __pyx_t_6 = 0;
  __pyx_t_6 = PyTuple_New(2); if (unlikely(!__pyx_t_6)) {__pyx_filename = __pyx_f[0]; __pyx_lineno = 266; __pyx_clineno = __LINE__; goto __pyx_L1_error;}
  __Pyx_GOTREF(__pyx_t_6);
  __Pyx_INCREF(__pyx_v_shape);
  PyTuple_SET_ITEM(__pyx_t_6, 0, __pyx_v_shape);
  __Pyx_GIVEREF(__pyx_v_shape);
  __Pyx_INCREF(__pyx_float_0_342780);
  PyTuple_SET_ITEM(__pyx_t_6, 1, __pyx_float_0_342780);
  __Pyx_GIVEREF(__pyx_float_0_342780);
  __pyx_t_5 = __Pyx_PyObject_Call(__pyx_t_3, __pyx_t_6, NULL); if (unlikely(!__pyx_t_5)) {__pyx_filename = __pyx_f[0]; __pyx_lineno = 266; __pyx_clineno = __LINE__; goto __pyx_L1_error;}
  __Pyx_GOTREF(__pyx_t_5);
  __Pyx_DECREF(__pyx_t_3); __pyx_t_3 = 0;
  __Pyx_DECREF(__pyx_t_6); __pyx_t_6 = 0;
  __pyx_t_30 = __Pyx_PyObject_to_MemoryviewSlice_d_dc_double(__pyx_t_5);
  if (unlikely(!__pyx_t_30.memview)) {__pyx_filename = __pyx_f[0]; __pyx_lineno = 266; __pyx_clineno = __LINE__; goto __pyx_L1_error;}
  __Pyx_DECREF(__pyx_t_5); __pyx_t_5 = 0;
  __PYX_XDEC_MEMVIEW(&__pyx_v_self->IC_Na3, 0);
  __pyx_v_self->IC_Na3 = __pyx_t_30;
  __pyx_t_30.memview = NULL;
  __pyx_t_30.data = NULL;
```

```
 267:         self.Na_i = np.full(shape, 14237.1)
```

```
  __pyx_t_5 = __Pyx_GetModuleGlobalName(__pyx_n_s_np); if (unlikely(!__pyx_t_5)) {__pyx_filename = __pyx_f[0]; __pyx_lineno = 267; __pyx_clineno = __LINE__; goto __pyx_L1_error;}
  __Pyx_GOTREF(__pyx_t_5);
  __pyx_t_6 = __Pyx_PyObject_GetAttrStr(__pyx_t_5, __pyx_n_s_full); if (unlikely(!__pyx_t_6)) {__pyx_filename = __pyx_f[0]; __pyx_lineno = 267; __pyx_clineno = __LINE__; goto __pyx_L1_error;}
  __Pyx_GOTREF(__pyx_t_6);
  __Pyx_DECREF(__pyx_t_5); __pyx_t_5 = 0;
  __pyx_t_5 = PyTuple_New(2); if (unlikely(!__pyx_t_5)) {__pyx_filename = __pyx_f[0]; __pyx_lineno = 267; __pyx_clineno = __LINE__; goto __pyx_L1_error;}
  __Pyx_GOTREF(__pyx_t_5);
  __Pyx_INCREF(__pyx_v_shape);
  PyTuple_SET_ITEM(__pyx_t_5, 0, __pyx_v_shape);
  __Pyx_GIVEREF(__pyx_v_shape);
  __Pyx_INCREF(__pyx_float_14237_1);
  PyTuple_SET_ITEM(__pyx_t_5, 1, __pyx_float_14237_1);
  __Pyx_GIVEREF(__pyx_float_14237_1);
  __pyx_t_3 = __Pyx_PyObject_Call(__pyx_t_6, __pyx_t_5, NULL); if (unlikely(!__pyx_t_3)) {__pyx_filename = __pyx_f[0]; __pyx_lineno = 267; __pyx_clineno = __LINE__; goto __pyx_L1_error;}
  __Pyx_GOTREF(__pyx_t_3);
  __Pyx_DECREF(__pyx_t_6); __pyx_t_6 = 0;
  __Pyx_DECREF(__pyx_t_5); __pyx_t_5 = 0;
  __pyx_t_31 = __Pyx_PyObject_to_MemoryviewSlice_d_dc_double(__pyx_t_3);
  if (unlikely(!__pyx_t_31.memview)) {__pyx_filename = __pyx_f[0]; __pyx_lineno = 267; __pyx_clineno = __LINE__; goto __pyx_L1_error;}
  __Pyx_DECREF(__pyx_t_3); __pyx_t_3 = 0;
  __PYX_XDEC_MEMVIEW(&__pyx_v_self->Na_i, 0);
  __pyx_v_self->Na_i = __pyx_t_31;
  __pyx_t_31.memview = NULL;
  __pyx_t_31.data = NULL;
```

```
 268:         self.K_i = np.full(shape, 143720.0)
```

```
  __pyx_t_3 = __Pyx_GetModuleGlobalName(__pyx_n_s_np); if (unlikely(!__pyx_t_3)) {__pyx_filename = __pyx_f[0]; __pyx_lineno = 268; __pyx_clineno = __LINE__; goto __pyx_L1_error;}
  __Pyx_GOTREF(__pyx_t_3);
  __pyx_t_5 = __Pyx_PyObject_GetAttrStr(__pyx_t_3, __pyx_n_s_full); if (unlikely(!__pyx_t_5)) {__pyx_filename = __pyx_f[0]; __pyx_lineno = 268; __pyx_clineno = __LINE__; goto __pyx_L1_error;}
  __Pyx_GOTREF(__pyx_t_5);
  __Pyx_DECREF(__pyx_t_3); __pyx_t_3 = 0;
  __pyx_t_3 = PyTuple_New(2); if (unlikely(!__pyx_t_3)) {__pyx_filename = __pyx_f[0]; __pyx_lineno = 268; __pyx_clineno = __LINE__; goto __pyx_L1_error;}
  __Pyx_GOTREF(__pyx_t_3);
  __Pyx_INCREF(__pyx_v_shape);
  PyTuple_SET_ITEM(__pyx_t_3, 0, __pyx_v_shape);
  __Pyx_GIVEREF(__pyx_v_shape);
  __Pyx_INCREF(__pyx_float_143720_0);
  PyTuple_SET_ITEM(__pyx_t_3, 1, __pyx_float_143720_0);
  __Pyx_GIVEREF(__pyx_float_143720_0);
  __pyx_t_6 = __Pyx_PyObject_Call(__pyx_t_5, __pyx_t_3, NULL); if (unlikely(!__pyx_t_6)) {__pyx_filename = __pyx_f[0]; __pyx_lineno = 268; __pyx_clineno = __LINE__; goto __pyx_L1_error;}
  __Pyx_GOTREF(__pyx_t_6);
  __Pyx_DECREF(__pyx_t_5); __pyx_t_5 = 0;
  __Pyx_DECREF(__pyx_t_3); __pyx_t_3 = 0;
  __pyx_t_32 = __Pyx_PyObject_to_MemoryviewSlice_d_dc_double(__pyx_t_6);
  if (unlikely(!__pyx_t_32.memview)) {__pyx_filename = __pyx_f[0]; __pyx_lineno = 268; __pyx_clineno = __LINE__; goto __pyx_L1_error;}
  __Pyx_DECREF(__pyx_t_6); __pyx_t_6 = 0;
  __PYX_XDEC_MEMVIEW(&__pyx_v_self->K_i, 0);
  __pyx_v_self->K_i = __pyx_t_32;
  __pyx_t_32.memview = NULL;
  __pyx_t_32.data = NULL;
```

```
 269:         self.a_tof = np.full(shape, 0.265563e-2)
```

```
  __pyx_t_6 = __Pyx_GetModuleGlobalName(__pyx_n_s_np); if (unlikely(!__pyx_t_6)) {__pyx_filename = __pyx_f[0]; __pyx_lineno = 269; __pyx_clineno = __LINE__; goto __pyx_L1_error;}
  __Pyx_GOTREF(__pyx_t_6);
  __pyx_t_3 = __Pyx_PyObject_GetAttrStr(__pyx_t_6, __pyx_n_s_full); if (unlikely(!__pyx_t_3)) {__pyx_filename = __pyx_f[0]; __pyx_lineno = 269; __pyx_clineno = __LINE__; goto __pyx_L1_error;}
  __Pyx_GOTREF(__pyx_t_3);
  __Pyx_DECREF(__pyx_t_6); __pyx_t_6 = 0;
  __pyx_t_6 = PyTuple_New(2); if (unlikely(!__pyx_t_6)) {__pyx_filename = __pyx_f[0]; __pyx_lineno = 269; __pyx_clineno = __LINE__; goto __pyx_L1_error;}
  __Pyx_GOTREF(__pyx_t_6);
  __Pyx_INCREF(__pyx_v_shape);
  PyTuple_SET_ITEM(__pyx_t_6, 0, __pyx_v_shape);
  __Pyx_GIVEREF(__pyx_v_shape);
  __Pyx_INCREF(__pyx_float_0_265563eneg_2);
  PyTuple_SET_ITEM(__pyx_t_6, 1, __pyx_float_0_265563eneg_2);
  __Pyx_GIVEREF(__pyx_float_0_265563eneg_2);
  __pyx_t_5 = __Pyx_PyObject_Call(__pyx_t_3, __pyx_t_6, NULL); if (unlikely(!__pyx_t_5)) {__pyx_filename = __pyx_f[0]; __pyx_lineno = 269; __pyx_clineno = __LINE__; goto __pyx_L1_error;}
  __Pyx_GOTREF(__pyx_t_5);
  __Pyx_DECREF(__pyx_t_3); __pyx_t_3 = 0;
  __Pyx_DECREF(__pyx_t_6); __pyx_t_6 = 0;
  __pyx_t_33 = __Pyx_PyObject_to_MemoryviewSlice_d_dc_double(__pyx_t_5);
  if (unlikely(!__pyx_t_33.memview)) {__pyx_filename = __pyx_f[0]; __pyx_lineno = 269; __pyx_clineno = __LINE__; goto __pyx_L1_error;}
  __Pyx_DECREF(__pyx_t_5); __pyx_t_5 = 0;
  __PYX_XDEC_MEMVIEW(&__pyx_v_self->a_tof, 0);
  __pyx_v_self->a_tof = __pyx_t_33;
  __pyx_t_33.memview = NULL;
  __pyx_t_33.data = NULL;
```

```
 270:         self.i_tof = np.full(shape, 0.999977)
```

```
  __pyx_t_5 = __Pyx_GetModuleGlobalName(__pyx_n_s_np); if (unlikely(!__pyx_t_5)) {__pyx_filename = __pyx_f[0]; __pyx_lineno = 270; __pyx_clineno = __LINE__; goto __pyx_L1_error;}
  __Pyx_GOTREF(__pyx_t_5);
  __pyx_t_6 = __Pyx_PyObject_GetAttrStr(__pyx_t_5, __pyx_n_s_full); if (unlikely(!__pyx_t_6)) {__pyx_filename = __pyx_f[0]; __pyx_lineno = 270; __pyx_clineno = __LINE__; goto __pyx_L1_error;}
  __Pyx_GOTREF(__pyx_t_6);
  __Pyx_DECREF(__pyx_t_5); __pyx_t_5 = 0;
  __pyx_t_5 = PyTuple_New(2); if (unlikely(!__pyx_t_5)) {__pyx_filename = __pyx_f[0]; __pyx_lineno = 270; __pyx_clineno = __LINE__; goto __pyx_L1_error;}
  __Pyx_GOTREF(__pyx_t_5);
  __Pyx_INCREF(__pyx_v_shape);
  PyTuple_SET_ITEM(__pyx_t_5, 0, __pyx_v_shape);
  __Pyx_GIVEREF(__pyx_v_shape);
  __Pyx_INCREF(__pyx_float_0_999977);
  PyTuple_SET_ITEM(__pyx_t_5, 1, __pyx_float_0_999977);
  __Pyx_GIVEREF(__pyx_float_0_999977);
  __pyx_t_3 = __Pyx_PyObject_Call(__pyx_t_6, __pyx_t_5, NULL); if (unlikely(!__pyx_t_3)) {__pyx_filename = __pyx_f[0]; __pyx_lineno = 270; __pyx_clineno = __LINE__; goto __pyx_L1_error;}
  __Pyx_GOTREF(__pyx_t_3);
  __Pyx_DECREF(__pyx_t_6); __pyx_t_6 = 0;
  __Pyx_DECREF(__pyx_t_5); __pyx_t_5 = 0;
  __pyx_t_34 = __Pyx_PyObject_to_MemoryviewSlice_d_dc_double(__pyx_t_3);
  if (unlikely(!__pyx_t_34.memview)) {__pyx_filename = __pyx_f[0]; __pyx_lineno = 270; __pyx_clineno = __LINE__; goto __pyx_L1_error;}
  __Pyx_DECREF(__pyx_t_3); __pyx_t_3 = 0;
  __PYX_XDEC_MEMVIEW(&__pyx_v_self->i_tof, 0);
  __pyx_v_self->i_tof = __pyx_t_34;
  __pyx_t_34.memview = NULL;
  __pyx_t_34.data = NULL;
```

```
 271:         self.n_Ks = np.full(shape, 0.262753e-3)
```

```
  __pyx_t_3 = __Pyx_GetModuleGlobalName(__pyx_n_s_np); if (unlikely(!__pyx_t_3)) {__pyx_filename = __pyx_f[0]; __pyx_lineno = 271; __pyx_clineno = __LINE__; goto __pyx_L1_error;}
  __Pyx_GOTREF(__pyx_t_3);
  __pyx_t_5 = __Pyx_PyObject_GetAttrStr(__pyx_t_3, __pyx_n_s_full); if (unlikely(!__pyx_t_5)) {__pyx_filename = __pyx_f[0]; __pyx_lineno = 271; __pyx_clineno = __LINE__; goto __pyx_L1_error;}
  __Pyx_GOTREF(__pyx_t_5);
  __Pyx_DECREF(__pyx_t_3); __pyx_t_3 = 0;
  __pyx_t_3 = PyTuple_New(2); if (unlikely(!__pyx_t_3)) {__pyx_filename = __pyx_f[0]; __pyx_lineno = 271; __pyx_clineno = __LINE__; goto __pyx_L1_error;}
  __Pyx_GOTREF(__pyx_t_3);
  __Pyx_INCREF(__pyx_v_shape);
  PyTuple_SET_ITEM(__pyx_t_3, 0, __pyx_v_shape);
  __Pyx_GIVEREF(__pyx_v_shape);
  __Pyx_INCREF(__pyx_float_0_262753eneg_3);
  PyTuple_SET_ITEM(__pyx_t_3, 1, __pyx_float_0_262753eneg_3);
  __Pyx_GIVEREF(__pyx_float_0_262753eneg_3);
  __pyx_t_6 = __Pyx_PyObject_Call(__pyx_t_5, __pyx_t_3, NULL); if (unlikely(!__pyx_t_6)) {__pyx_filename = __pyx_f[0]; __pyx_lineno = 271; __pyx_clineno = __LINE__; goto __pyx_L1_error;}
  __Pyx_GOTREF(__pyx_t_6);
  __Pyx_DECREF(__pyx_t_5); __pyx_t_5 = 0;
  __Pyx_DECREF(__pyx_t_3); __pyx_t_3 = 0;
  __pyx_t_35 = __Pyx_PyObject_to_MemoryviewSlice_d_dc_double(__pyx_t_6);
  if (unlikely(!__pyx_t_35.memview)) {__pyx_filename = __pyx_f[0]; __pyx_lineno = 271; __pyx_clineno = __LINE__; goto __pyx_L1_error;}
  __Pyx_DECREF(__pyx_t_6); __pyx_t_6 = 0;
  __PYX_XDEC_MEMVIEW(&__pyx_v_self->n_Ks, 0);
  __pyx_v_self->n_Ks = __pyx_t_35;
  __pyx_t_35.memview = NULL;
  __pyx_t_35.data = NULL;
```

```
 272:         self.a_tos = np.full(shape, 0.417069e-3)
```

```
  __pyx_t_6 = __Pyx_GetModuleGlobalName(__pyx_n_s_np); if (unlikely(!__pyx_t_6)) {__pyx_filename = __pyx_f[0]; __pyx_lineno = 272; __pyx_clineno = __LINE__; goto __pyx_L1_error;}
  __Pyx_GOTREF(__pyx_t_6);
  __pyx_t_3 = __Pyx_PyObject_GetAttrStr(__pyx_t_6, __pyx_n_s_full); if (unlikely(!__pyx_t_3)) {__pyx_filename = __pyx_f[0]; __pyx_lineno = 272; __pyx_clineno = __LINE__; goto __pyx_L1_error;}
  __Pyx_GOTREF(__pyx_t_3);
  __Pyx_DECREF(__pyx_t_6); __pyx_t_6 = 0;
  __pyx_t_6 = PyTuple_New(2); if (unlikely(!__pyx_t_6)) {__pyx_filename = __pyx_f[0]; __pyx_lineno = 272; __pyx_clineno = __LINE__; goto __pyx_L1_error;}
  __Pyx_GOTREF(__pyx_t_6);
  __Pyx_INCREF(__pyx_v_shape);
  PyTuple_SET_ITEM(__pyx_t_6, 0, __pyx_v_shape);
  __Pyx_GIVEREF(__pyx_v_shape);
  __Pyx_INCREF(__pyx_float_0_417069eneg_3);
  PyTuple_SET_ITEM(__pyx_t_6, 1, __pyx_float_0_417069eneg_3);
  __Pyx_GIVEREF(__pyx_float_0_417069eneg_3);
  __pyx_t_5 = __Pyx_PyObject_Call(__pyx_t_3, __pyx_t_6, NULL); if (unlikely(!__pyx_t_5)) {__pyx_filename = __pyx_f[0]; __pyx_lineno = 272; __pyx_clineno = __LINE__; goto __pyx_L1_error;}
  __Pyx_GOTREF(__pyx_t_5);
  __Pyx_DECREF(__pyx_t_3); __pyx_t_3 = 0;
  __Pyx_DECREF(__pyx_t_6); __pyx_t_6 = 0;
  __pyx_t_36 = __Pyx_PyObject_to_MemoryviewSlice_d_dc_double(__pyx_t_5);
  if (unlikely(!__pyx_t_36.memview)) {__pyx_filename = __pyx_f[0]; __pyx_lineno = 272; __pyx_clineno = __LINE__; goto __pyx_L1_error;}
  __Pyx_DECREF(__pyx_t_5); __pyx_t_5 = 0;
  __PYX_XDEC_MEMVIEW(&__pyx_v_self->a_tos, 0);
  __pyx_v_self->a_tos = __pyx_t_36;
  __pyx_t_36.memview = NULL;
  __pyx_t_36.data = NULL;
```

```
 273:         self.i_tos = np.full(shape, 0.998543)
```

```
  __pyx_t_5 = __Pyx_GetModuleGlobalName(__pyx_n_s_np); if (unlikely(!__pyx_t_5)) {__pyx_filename = __pyx_f[0]; __pyx_lineno = 273; __pyx_clineno = __LINE__; goto __pyx_L1_error;}
  __Pyx_GOTREF(__pyx_t_5);
  __pyx_t_6 = __Pyx_PyObject_GetAttrStr(__pyx_t_5, __pyx_n_s_full); if (unlikely(!__pyx_t_6)) {__pyx_filename = __pyx_f[0]; __pyx_lineno = 273; __pyx_clineno = __LINE__; goto __pyx_L1_error;}
  __Pyx_GOTREF(__pyx_t_6);
  __Pyx_DECREF(__pyx_t_5); __pyx_t_5 = 0;
  __pyx_t_5 = PyTuple_New(2); if (unlikely(!__pyx_t_5)) {__pyx_filename = __pyx_f[0]; __pyx_lineno = 273; __pyx_clineno = __LINE__; goto __pyx_L1_error;}
  __Pyx_GOTREF(__pyx_t_5);
  __Pyx_INCREF(__pyx_v_shape);
  PyTuple_SET_ITEM(__pyx_t_5, 0, __pyx_v_shape);
  __Pyx_GIVEREF(__pyx_v_shape);
  __Pyx_INCREF(__pyx_float_0_998543);
  PyTuple_SET_ITEM(__pyx_t_5, 1, __pyx_float_0_998543);
  __Pyx_GIVEREF(__pyx_float_0_998543);
  __pyx_t_3 = __Pyx_PyObject_Call(__pyx_t_6, __pyx_t_5, NULL); if (unlikely(!__pyx_t_3)) {__pyx_filename = __pyx_f[0]; __pyx_lineno = 273; __pyx_clineno = __LINE__; goto __pyx_L1_error;}
  __Pyx_GOTREF(__pyx_t_3);
  __Pyx_DECREF(__pyx_t_6); __pyx_t_6 = 0;
  __Pyx_DECREF(__pyx_t_5); __pyx_t_5 = 0;
  __pyx_t_37 = __Pyx_PyObject_to_MemoryviewSlice_d_dc_double(__pyx_t_3);
  if (unlikely(!__pyx_t_37.memview)) {__pyx_filename = __pyx_f[0]; __pyx_lineno = 273; __pyx_clineno = __LINE__; goto __pyx_L1_error;}
  __Pyx_DECREF(__pyx_t_3); __pyx_t_3 = 0;
  __PYX_XDEC_MEMVIEW(&__pyx_v_self->i_tos, 0);
  __pyx_v_self->i_tos = __pyx_t_37;
  __pyx_t_37.memview = NULL;
  __pyx_t_37.data = NULL;
```

```
 274:         self.a_ur = np.full(shape, 0.417069e-3)
```

```
  __pyx_t_3 = __Pyx_GetModuleGlobalName(__pyx_n_s_np); if (unlikely(!__pyx_t_3)) {__pyx_filename = __pyx_f[0]; __pyx_lineno = 274; __pyx_clineno = __LINE__; goto __pyx_L1_error;}
  __Pyx_GOTREF(__pyx_t_3);
  __pyx_t_5 = __Pyx_PyObject_GetAttrStr(__pyx_t_3, __pyx_n_s_full); if (unlikely(!__pyx_t_5)) {__pyx_filename = __pyx_f[0]; __pyx_lineno = 274; __pyx_clineno = __LINE__; goto __pyx_L1_error;}
  __Pyx_GOTREF(__pyx_t_5);
  __Pyx_DECREF(__pyx_t_3); __pyx_t_3 = 0;
  __pyx_t_3 = PyTuple_New(2); if (unlikely(!__pyx_t_3)) {__pyx_filename = __pyx_f[0]; __pyx_lineno = 274; __pyx_clineno = __LINE__; goto __pyx_L1_error;}
  __Pyx_GOTREF(__pyx_t_3);
  __Pyx_INCREF(__pyx_v_shape);
  PyTuple_SET_ITEM(__pyx_t_3, 0, __pyx_v_shape);
  __Pyx_GIVEREF(__pyx_v_shape);
  __Pyx_INCREF(__pyx_float_0_417069eneg_3);
  PyTuple_SET_ITEM(__pyx_t_3, 1, __pyx_float_0_417069eneg_3);
  __Pyx_GIVEREF(__pyx_float_0_417069eneg_3);
  __pyx_t_6 = __Pyx_PyObject_Call(__pyx_t_5, __pyx_t_3, NULL); if (unlikely(!__pyx_t_6)) {__pyx_filename = __pyx_f[0]; __pyx_lineno = 274; __pyx_clineno = __LINE__; goto __pyx_L1_error;}
  __Pyx_GOTREF(__pyx_t_6);
  __Pyx_DECREF(__pyx_t_5); __pyx_t_5 = 0;
  __Pyx_DECREF(__pyx_t_3); __pyx_t_3 = 0;
  __pyx_t_38 = __Pyx_PyObject_to_MemoryviewSlice_d_dc_double(__pyx_t_6);
  if (unlikely(!__pyx_t_38.memview)) {__pyx_filename = __pyx_f[0]; __pyx_lineno = 274; __pyx_clineno = __LINE__; goto __pyx_L1_error;}
  __Pyx_DECREF(__pyx_t_6); __pyx_t_6 = 0;
  __PYX_XDEC_MEMVIEW(&__pyx_v_self->a_ur, 0);
  __pyx_v_self->a_ur = __pyx_t_38;
  __pyx_t_38.memview = NULL;
  __pyx_t_38.data = NULL;
```

```
 275:         self.i_ur = np.full(shape, 0.998543)
```

```
  __pyx_t_6 = __Pyx_GetModuleGlobalName(__pyx_n_s_np); if (unlikely(!__pyx_t_6)) {__pyx_filename = __pyx_f[0]; __pyx_lineno = 275; __pyx_clineno = __LINE__; goto __pyx_L1_error;}
  __Pyx_GOTREF(__pyx_t_6);
  __pyx_t_3 = __Pyx_PyObject_GetAttrStr(__pyx_t_6, __pyx_n_s_full); if (unlikely(!__pyx_t_3)) {__pyx_filename = __pyx_f[0]; __pyx_lineno = 275; __pyx_clineno = __LINE__; goto __pyx_L1_error;}
  __Pyx_GOTREF(__pyx_t_3);
  __Pyx_DECREF(__pyx_t_6); __pyx_t_6 = 0;
  __pyx_t_6 = PyTuple_New(2); if (unlikely(!__pyx_t_6)) {__pyx_filename = __pyx_f[0]; __pyx_lineno = 275; __pyx_clineno = __LINE__; goto __pyx_L1_error;}
  __Pyx_GOTREF(__pyx_t_6);
  __Pyx_INCREF(__pyx_v_shape);
  PyTuple_SET_ITEM(__pyx_t_6, 0, __pyx_v_shape);
  __Pyx_GIVEREF(__pyx_v_shape);
  __Pyx_INCREF(__pyx_float_0_998543);
  PyTuple_SET_ITEM(__pyx_t_6, 1, __pyx_float_0_998543);
  __Pyx_GIVEREF(__pyx_float_0_998543);
  __pyx_t_5 = __Pyx_PyObject_Call(__pyx_t_3, __pyx_t_6, NULL); if (unlikely(!__pyx_t_5)) {__pyx_filename = __pyx_f[0]; __pyx_lineno = 275; __pyx_clineno = __LINE__; goto __pyx_L1_error;}
  __Pyx_GOTREF(__pyx_t_5);
  __Pyx_DECREF(__pyx_t_3); __pyx_t_3 = 0;
  __Pyx_DECREF(__pyx_t_6); __pyx_t_6 = 0;
  __pyx_t_39 = __Pyx_PyObject_to_MemoryviewSlice_d_dc_double(__pyx_t_5);
  if (unlikely(!__pyx_t_39.memview)) {__pyx_filename = __pyx_f[0]; __pyx_lineno = 275; __pyx_clineno = __LINE__; goto __pyx_L1_error;}
  __Pyx_DECREF(__pyx_t_5); __pyx_t_5 = 0;
  __PYX_XDEC_MEMVIEW(&__pyx_v_self->i_ur, 0);
  __pyx_v_self->i_ur = __pyx_t_39;
  __pyx_t_39.memview = NULL;
  __pyx_t_39.data = NULL;
```

```
 276:         self.a_Kss = np.full(shape, 0.417069e-3)
```

```
  __pyx_t_5 = __Pyx_GetModuleGlobalName(__pyx_n_s_np); if (unlikely(!__pyx_t_5)) {__pyx_filename = __pyx_f[0]; __pyx_lineno = 276; __pyx_clineno = __LINE__; goto __pyx_L1_error;}
  __Pyx_GOTREF(__pyx_t_5);
  __pyx_t_6 = __Pyx_PyObject_GetAttrStr(__pyx_t_5, __pyx_n_s_full); if (unlikely(!__pyx_t_6)) {__pyx_filename = __pyx_f[0]; __pyx_lineno = 276; __pyx_clineno = __LINE__; goto __pyx_L1_error;}
  __Pyx_GOTREF(__pyx_t_6);
  __Pyx_DECREF(__pyx_t_5); __pyx_t_5 = 0;
  __pyx_t_5 = PyTuple_New(2); if (unlikely(!__pyx_t_5)) {__pyx_filename = __pyx_f[0]; __pyx_lineno = 276; __pyx_clineno = __LINE__; goto __pyx_L1_error;}
  __Pyx_GOTREF(__pyx_t_5);
  __Pyx_INCREF(__pyx_v_shape);
  PyTuple_SET_ITEM(__pyx_t_5, 0, __pyx_v_shape);
  __Pyx_GIVEREF(__pyx_v_shape);
  __Pyx_INCREF(__pyx_float_0_417069eneg_3);
  PyTuple_SET_ITEM(__pyx_t_5, 1, __pyx_float_0_417069eneg_3);
  __Pyx_GIVEREF(__pyx_float_0_417069eneg_3);
  __pyx_t_3 = __Pyx_PyObject_Call(__pyx_t_6, __pyx_t_5, NULL); if (unlikely(!__pyx_t_3)) {__pyx_filename = __pyx_f[0]; __pyx_lineno = 276; __pyx_clineno = __LINE__; goto __pyx_L1_error;}
  __Pyx_GOTREF(__pyx_t_3);
  __Pyx_DECREF(__pyx_t_6); __pyx_t_6 = 0;
  __Pyx_DECREF(__pyx_t_5); __pyx_t_5 = 0;
  __pyx_t_40 = __Pyx_PyObject_to_MemoryviewSlice_d_dc_double(__pyx_t_3);
  if (unlikely(!__pyx_t_40.memview)) {__pyx_filename = __pyx_f[0]; __pyx_lineno = 276; __pyx_clineno = __LINE__; goto __pyx_L1_error;}
  __Pyx_DECREF(__pyx_t_3); __pyx_t_3 = 0;
  __PYX_XDEC_MEMVIEW(&__pyx_v_self->a_Kss, 0);
  __pyx_v_self->a_Kss = __pyx_t_40;
  __pyx_t_40.memview = NULL;
  __pyx_t_40.data = NULL;
```

```
 277:         self.C_K1 = np.full(shape, 0.992513e-3 )
```

```
  __pyx_t_3 = __Pyx_GetModuleGlobalName(__pyx_n_s_np); if (unlikely(!__pyx_t_3)) {__pyx_filename = __pyx_f[0]; __pyx_lineno = 277; __pyx_clineno = __LINE__; goto __pyx_L1_error;}
  __Pyx_GOTREF(__pyx_t_3);
  __pyx_t_5 = __Pyx_PyObject_GetAttrStr(__pyx_t_3, __pyx_n_s_full); if (unlikely(!__pyx_t_5)) {__pyx_filename = __pyx_f[0]; __pyx_lineno = 277; __pyx_clineno = __LINE__; goto __pyx_L1_error;}
  __Pyx_GOTREF(__pyx_t_5);
  __Pyx_DECREF(__pyx_t_3); __pyx_t_3 = 0;
  __pyx_t_3 = PyTuple_New(2); if (unlikely(!__pyx_t_3)) {__pyx_filename = __pyx_f[0]; __pyx_lineno = 277; __pyx_clineno = __LINE__; goto __pyx_L1_error;}
  __Pyx_GOTREF(__pyx_t_3);
  __Pyx_INCREF(__pyx_v_shape);
  PyTuple_SET_ITEM(__pyx_t_3, 0, __pyx_v_shape);
  __Pyx_GIVEREF(__pyx_v_shape);
  __Pyx_INCREF(__pyx_float_0_992513eneg_3);
  PyTuple_SET_ITEM(__pyx_t_3, 1, __pyx_float_0_992513eneg_3);
  __Pyx_GIVEREF(__pyx_float_0_992513eneg_3);
  __pyx_t_6 = __Pyx_PyObject_Call(__pyx_t_5, __pyx_t_3, NULL); if (unlikely(!__pyx_t_6)) {__pyx_filename = __pyx_f[0]; __pyx_lineno = 277; __pyx_clineno = __LINE__; goto __pyx_L1_error;}
  __Pyx_GOTREF(__pyx_t_6);
  __Pyx_DECREF(__pyx_t_5); __pyx_t_5 = 0;
  __Pyx_DECREF(__pyx_t_3); __pyx_t_3 = 0;
  __pyx_t_41 = __Pyx_PyObject_to_MemoryviewSlice_d_dc_double(__pyx_t_6);
  if (unlikely(!__pyx_t_41.memview)) {__pyx_filename = __pyx_f[0]; __pyx_lineno = 277; __pyx_clineno = __LINE__; goto __pyx_L1_error;}
  __Pyx_DECREF(__pyx_t_6); __pyx_t_6 = 0;
  __PYX_XDEC_MEMVIEW(&__pyx_v_self->C_K1, 0);
  __pyx_v_self->C_K1 = __pyx_t_41;
  __pyx_t_41.memview = NULL;
  __pyx_t_41.data = NULL;
```

```
 278:         self.C_K2 = np.full(shape, 0.641229e-3)
```

```
  __pyx_t_6 = __Pyx_GetModuleGlobalName(__pyx_n_s_np); if (unlikely(!__pyx_t_6)) {__pyx_filename = __pyx_f[0]; __pyx_lineno = 278; __pyx_clineno = __LINE__; goto __pyx_L1_error;}
  __Pyx_GOTREF(__pyx_t_6);
  __pyx_t_3 = __Pyx_PyObject_GetAttrStr(__pyx_t_6, __pyx_n_s_full); if (unlikely(!__pyx_t_3)) {__pyx_filename = __pyx_f[0]; __pyx_lineno = 278; __pyx_clineno = __LINE__; goto __pyx_L1_error;}
  __Pyx_GOTREF(__pyx_t_3);
  __Pyx_DECREF(__pyx_t_6); __pyx_t_6 = 0;
  __pyx_t_6 = PyTuple_New(2); if (unlikely(!__pyx_t_6)) {__pyx_filename = __pyx_f[0]; __pyx_lineno = 278; __pyx_clineno = __LINE__; goto __pyx_L1_error;}
  __Pyx_GOTREF(__pyx_t_6);
  __Pyx_INCREF(__pyx_v_shape);
  PyTuple_SET_ITEM(__pyx_t_6, 0, __pyx_v_shape);
  __Pyx_GIVEREF(__pyx_v_shape);
  __Pyx_INCREF(__pyx_float_0_641229eneg_3);
  PyTuple_SET_ITEM(__pyx_t_6, 1, __pyx_float_0_641229eneg_3);
  __Pyx_GIVEREF(__pyx_float_0_641229eneg_3);
  __pyx_t_5 = __Pyx_PyObject_Call(__pyx_t_3, __pyx_t_6, NULL); if (unlikely(!__pyx_t_5)) {__pyx_filename = __pyx_f[0]; __pyx_lineno = 278; __pyx_clineno = __LINE__; goto __pyx_L1_error;}
  __Pyx_GOTREF(__pyx_t_5);
  __Pyx_DECREF(__pyx_t_3); __pyx_t_3 = 0;
  __Pyx_DECREF(__pyx_t_6); __pyx_t_6 = 0;
  __pyx_t_42 = __Pyx_PyObject_to_MemoryviewSlice_d_dc_double(__pyx_t_5);
  if (unlikely(!__pyx_t_42.memview)) {__pyx_filename = __pyx_f[0]; __pyx_lineno = 278; __pyx_clineno = __LINE__; goto __pyx_L1_error;}
  __Pyx_DECREF(__pyx_t_5); __pyx_t_5 = 0;
  __PYX_XDEC_MEMVIEW(&__pyx_v_self->C_K2, 0);
  __pyx_v_self->C_K2 = __pyx_t_42;
  __pyx_t_42.memview = NULL;
  __pyx_t_42.data = NULL;
```

```
 279:         self.O_K = np.full(shape, 0.175298e-3)
```

```
  __pyx_t_5 = __Pyx_GetModuleGlobalName(__pyx_n_s_np); if (unlikely(!__pyx_t_5)) {__pyx_filename = __pyx_f[0]; __pyx_lineno = 279; __pyx_clineno = __LINE__; goto __pyx_L1_error;}
  __Pyx_GOTREF(__pyx_t_5);
  __pyx_t_6 = __Pyx_PyObject_GetAttrStr(__pyx_t_5, __pyx_n_s_full); if (unlikely(!__pyx_t_6)) {__pyx_filename = __pyx_f[0]; __pyx_lineno = 279; __pyx_clineno = __LINE__; goto __pyx_L1_error;}
  __Pyx_GOTREF(__pyx_t_6);
  __Pyx_DECREF(__pyx_t_5); __pyx_t_5 = 0;
  __pyx_t_5 = PyTuple_New(2); if (unlikely(!__pyx_t_5)) {__pyx_filename = __pyx_f[0]; __pyx_lineno = 279; __pyx_clineno = __LINE__; goto __pyx_L1_error;}
  __Pyx_GOTREF(__pyx_t_5);
  __Pyx_INCREF(__pyx_v_shape);
  PyTuple_SET_ITEM(__pyx_t_5, 0, __pyx_v_shape);
  __Pyx_GIVEREF(__pyx_v_shape);
  __Pyx_INCREF(__pyx_float_0_175298eneg_3);
  PyTuple_SET_ITEM(__pyx_t_5, 1, __pyx_float_0_175298eneg_3);
  __Pyx_GIVEREF(__pyx_float_0_175298eneg_3);
  __pyx_t_3 = __Pyx_PyObject_Call(__pyx_t_6, __pyx_t_5, NULL); if (unlikely(!__pyx_t_3)) {__pyx_filename = __pyx_f[0]; __pyx_lineno = 279; __pyx_clineno = __LINE__; goto __pyx_L1_error;}
  __Pyx_GOTREF(__pyx_t_3);
  __Pyx_DECREF(__pyx_t_6); __pyx_t_6 = 0;
  __Pyx_DECREF(__pyx_t_5); __pyx_t_5 = 0;
  __pyx_t_43 = __Pyx_PyObject_to_MemoryviewSlice_d_dc_double(__pyx_t_3);
  if (unlikely(!__pyx_t_43.memview)) {__pyx_filename = __pyx_f[0]; __pyx_lineno = 279; __pyx_clineno = __LINE__; goto __pyx_L1_error;}
  __Pyx_DECREF(__pyx_t_3); __pyx_t_3 = 0;
  __PYX_XDEC_MEMVIEW(&__pyx_v_self->O_K, 0);
  __pyx_v_self->O_K = __pyx_t_43;
  __pyx_t_43.memview = NULL;
  __pyx_t_43.data = NULL;
```

```
 280:         self.I_K = np.full(shape, 0.319129e-4)
```

```
  __pyx_t_3 = __Pyx_GetModuleGlobalName(__pyx_n_s_np); if (unlikely(!__pyx_t_3)) {__pyx_filename = __pyx_f[0]; __pyx_lineno = 280; __pyx_clineno = __LINE__; goto __pyx_L1_error;}
  __Pyx_GOTREF(__pyx_t_3);
  __pyx_t_5 = __Pyx_PyObject_GetAttrStr(__pyx_t_3, __pyx_n_s_full); if (unlikely(!__pyx_t_5)) {__pyx_filename = __pyx_f[0]; __pyx_lineno = 280; __pyx_clineno = __LINE__; goto __pyx_L1_error;}
  __Pyx_GOTREF(__pyx_t_5);
  __Pyx_DECREF(__pyx_t_3); __pyx_t_3 = 0;
  __pyx_t_3 = PyTuple_New(2); if (unlikely(!__pyx_t_3)) {__pyx_filename = __pyx_f[0]; __pyx_lineno = 280; __pyx_clineno = __LINE__; goto __pyx_L1_error;}
  __Pyx_GOTREF(__pyx_t_3);
  __Pyx_INCREF(__pyx_v_shape);
  PyTuple_SET_ITEM(__pyx_t_3, 0, __pyx_v_shape);
  __Pyx_GIVEREF(__pyx_v_shape);
  __Pyx_INCREF(__pyx_float_0_319129eneg_4);
  PyTuple_SET_ITEM(__pyx_t_3, 1, __pyx_float_0_319129eneg_4);
  __Pyx_GIVEREF(__pyx_float_0_319129eneg_4);
  __pyx_t_6 = __Pyx_PyObject_Call(__pyx_t_5, __pyx_t_3, NULL); if (unlikely(!__pyx_t_6)) {__pyx_filename = __pyx_f[0]; __pyx_lineno = 280; __pyx_clineno = __LINE__; goto __pyx_L1_error;}
  __Pyx_GOTREF(__pyx_t_6);
  __Pyx_DECREF(__pyx_t_5); __pyx_t_5 = 0;
  __Pyx_DECREF(__pyx_t_3); __pyx_t_3 = 0;
  __pyx_t_44 = __Pyx_PyObject_to_MemoryviewSlice_d_dc_double(__pyx_t_6);
  if (unlikely(!__pyx_t_44.memview)) {__pyx_filename = __pyx_f[0]; __pyx_lineno = 280; __pyx_clineno = __LINE__; goto __pyx_L1_error;}
  __Pyx_DECREF(__pyx_t_6); __pyx_t_6 = 0;
  __PYX_XDEC_MEMVIEW(&__pyx_v_self->I_K, 0);
  __pyx_v_self->I_K = __pyx_t_44;
  __pyx_t_44.memview = NULL;
  __pyx_t_44.data = NULL;
```

```
 281:
```

```
 282:
```

```
 283:
```

```
 284:
```

```
 285:     cpdef local_step(self):
```

```
static PyObject *__pyx_pw_5model_10Bondarenko_7local_step(PyObject *__pyx_v_self, CYTHON_UNUSED PyObject *unused); /*proto*/
static PyObject *__pyx_f_5model_10Bondarenko_local_step(struct __pyx_obj_5model_Bondarenko *__pyx_v_self, int __pyx_skip_dispatch) {
  int __pyx_v_nx;
  int __pyx_v_ny;
  CYTHON_UNUSED double __pyx_v_A_cap;
  CYTHON_UNUSED double __pyx_v_V_myo;
  CYTHON_UNUSED double __pyx_v_V_JSR;
  CYTHON_UNUSED double __pyx_v_V_NSR;
  CYTHON_UNUSED double __pyx_v_V_ss;
  double __pyx_v_K_0;
  double __pyx_v_Na_0;
  double __pyx_v_Ca_0;
  double __pyx_v_v_1;
  double __pyx_v_v_2;
  double __pyx_v_v_3;
  double __pyx_v_k_mup;
  double __pyx_v_t_tr;
  double __pyx_v_t_xfer;
  CYTHON_UNUSED double __pyx_v_k_p_a;
  CYTHON_UNUSED double __pyx_v_k_n_a;
  CYTHON_UNUSED double __pyx_v_k_p_b;
  CYTHON_UNUSED double __pyx_v_k_n_b;
  CYTHON_UNUSED double __pyx_v_k_p_c;
  CYTHON_UNUSED double __pyx_v_k_n_c;
  CYTHON_UNUSED double __pyx_v_n;
  CYTHON_UNUSED double __pyx_v_m;
  double __pyx_v_G_CaL;
  double __pyx_v_E_CaL;
  double __pyx_v_k_pcmax;
  double __pyx_v_k_pchalf;
  CYTHON_UNUSED double __pyx_v_k_pcb;
  CYTHON_UNUSED double __pyx_v_I_CaLmax;
  double __pyx_v_LTRPN_tot;
  double __pyx_v_HTRPN_tot;
  double __pyx_v_k_p_htrpn;
  double __pyx_v_k_n_htrpn;
  double __pyx_v_k_p_ltrpn;
  double __pyx_v_k_n_ltrpn;
  double __pyx_v_CMDN_tot;
  double __pyx_v_CSQN_tot;
  double __pyx_v_k_CMDN_m;
  double __pyx_v_k_CSQN_m;
  CYTHON_UNUSED double __pyx_v_C_m;
  double __pyx_v_F;
  double __pyx_v_T;
  double __pyx_v_R;
  double __pyx_v_k_NaCa;
  double __pyx_v_k_mNa;
  double __pyx_v_k_mCa;
  double __pyx_v_k_sat;
  double __pyx_v_miu;
  double __pyx_v_I_maxNaK;
  double __pyx_v_K_mNai;
  double __pyx_v_K_mKo;
  double __pyx_v_I_maxpCa;
  double __pyx_v_k_mpCa;
  double __pyx_v_G_Cab;
  double __pyx_v_G_Na;
  double __pyx_v_G_Nab;
  double __pyx_v_G_Ktof;
  double __pyx_v_G_Ks;
  double __pyx_v_G_Ktos;
  double __pyx_v_G_Kur;
  double __pyx_v_G_Kss;
  double __pyx_v_G_Kr;
  CYTHON_UNUSED double __pyx_v_k_f;
  CYTHON_UNUSED double __pyx_v_k_b;
  double __pyx_v_G_ClCa;
  double __pyx_v_K_mCl;
  double __pyx_v_E_Cl;
  CYTHON_UNUSED double __pyx_v_Laplas_c;
  CYTHON_UNUSED double __pyx_v_Laplas_r;
  CYTHON_UNUSED double __pyx_v_V_ChR2;
  double __pyx_v_i_Kss;
  double __pyx_v_h;
  double __pyx_v_I_stim;
  double __pyx_v_V;
  double __pyx_v_Ca2_i;
  double __pyx_v_Ca2_ss;
  double __pyx_v_Ca2_JSR;
  double __pyx_v_Ca2_NSR;
  double __pyx_v_LTRPNCa;
  double __pyx_v_HTRPNCa;
  double __pyx_v_O;
  double __pyx_v_C2;
  double __pyx_v_C3;
  double __pyx_v_C4;
  double __pyx_v_I1;
  double __pyx_v_I2;
  double __pyx_v_I3;
  double __pyx_v_Pc2;
  double __pyx_v_Po1;
  double __pyx_v_Po2;
  double __pyx_v_P_RyR;
  double __pyx_v_C_Na2;
  double __pyx_v_C_Na1;
  double __pyx_v_O_Na;
  double __pyx_v_IF_Na;
  double __pyx_v_I1_Na;
  double __pyx_v_I2_Na;
  double __pyx_v_IC_Na2;
  double __pyx_v_IC_Na3;
  double __pyx_v_Na_i;
  double __pyx_v_K_i;
  double __pyx_v_a_tof;
  double __pyx_v_i_tof;
  double __pyx_v_n_Ks;
  double __pyx_v_a_tos;
  double __pyx_v_i_tos;
  double __pyx_v_a_ur;
  double __pyx_v_i_ur;
  double __pyx_v_a_Kss;
  double __pyx_v_C_K1;
  double __pyx_v_C_K2;
  double __pyx_v_O_K;
  double __pyx_v_I_K;
  int __pyx_v_j;
  int __pyx_v_k;
  int __pyx_v_x;
  int __pyx_v_y;
  __Pyx_memviewslice __pyx_v_dudx2 = { 0, 0, { 0 }, { 0 }, { 0 } };
  __Pyx_memviewslice __pyx_v_dudy2 = { 0, 0, { 0 }, { 0 }, { 0 } };
  double __pyx_v_B_i;
  double __pyx_v_J_leak;
  double __pyx_v_J_xfer;
  double __pyx_v_J_up;
  double __pyx_v_J_trpn;
  double __pyx_v_E_CaN;
  double __pyx_v_I_Cab;
  double __pyx_v_I_CaL;
  double __pyx_v_I_NaCa;
  double __pyx_v_I_pCa;
  double __pyx_v_k1_Ca2_i;
  double __pyx_v_k2_Ca2_i;
  double __pyx_v_k3_Ca2_i;
  double __pyx_v_k4_Ca2_i;
  double __pyx_v_dCa2_i;
  double __pyx_v_Pc1;
  double __pyx_v_k1_Po1;
  double __pyx_v_k2_Po1;
  double __pyx_v_k3_Po1;
  double __pyx_v_k4_Po1;
  double __pyx_v_dPo1;
  double __pyx_v_k1_Po2;
  double __pyx_v_k2_Po2;
  double __pyx_v_k3_Po2;
  double __pyx_v_k4_Po2;
  double __pyx_v_dPo2;
  double __pyx_v_B_ss;
  double __pyx_v_B_JSR;
  double __pyx_v_J_rel;
  double __pyx_v_J_tr;
  double __pyx_v_k1_Ca2_ss;
  double __pyx_v_k2_Ca2_ss;
  double __pyx_v_k3_Ca2_ss;
  double __pyx_v_k4_Ca2_ss;
  double __pyx_v_dCa2_ss;
  double __pyx_v_k1_Ca2_JSR;
  double __pyx_v_k2_Ca2_JSR;
  double __pyx_v_k3_Ca2_JSR;
  double __pyx_v_k4_Ca2_JSR;
  double __pyx_v_dCa2_JSR;
  double __pyx_v_k1_Ca2_NSR;
  double __pyx_v_k2_Ca2_NSR;
  double __pyx_v_k3_Ca2_NSR;
  double __pyx_v_k4_Ca2_NSR;
  double __pyx_v_dCa2_NSR;
  double __pyx_v_k1_P_RyR;
  double __pyx_v_k2_P_RyR;
  double __pyx_v_k3_P_RyR;
  double __pyx_v_k4_P_RyR;
  double __pyx_v_dP_RyR;
  double __pyx_v_k1_LTRPNCa;
  double __pyx_v_k2_LTRPNCa;
  double __pyx_v_k3_LTRPNCa;
  double __pyx_v_k4_LTRPNCa;
  double __pyx_v_dLTRPNCa;
  double __pyx_v_k1_HTRPNCa;
  double __pyx_v_k2_HTRPNCa;
  double __pyx_v_k3_HTRPNCa;
  double __pyx_v_k4_HTRPNCa;
  double __pyx_v_dHTRPNCa;
  double __pyx_v_k1_Pc2;
  double __pyx_v_k2_Pc2;
  double __pyx_v_k3_Pc2;
  double __pyx_v_k4_Pc2;
  double __pyx_v_dPc2;
  double __pyx_v_g;
  double __pyx_v_a;
  double __pyx_v_B;
  double __pyx_v_k_pcf;
  double __pyx_v_k1_O;
  double __pyx_v_k2_O;
  double __pyx_v_k3_O;
  double __pyx_v_k4_O;
  double __pyx_v_dO;
  double __pyx_v_C1;
  double __pyx_v_k1_c2;
  double __pyx_v_k2_c2;
  double __pyx_v_k3_c2;
  double __pyx_v_k4_c2;
  double __pyx_v_dC2;
  double __pyx_v_k1_c3;
  double __pyx_v_k2_c3;
  double __pyx_v_k3_c3;
  double __pyx_v_k4_c3;
  double __pyx_v_dC3;
  double __pyx_v_k1_c4;
  double __pyx_v_k2_c4;
  double __pyx_v_k3_c4;
  double __pyx_v_k4_c4;
  double __pyx_v_dC4;
  double __pyx_v_k1_I1;
  double __pyx_v_k2_I1;
  double __pyx_v_k3_I1;
  double __pyx_v_k4_I1;
  double __pyx_v_dI1;
  double __pyx_v_k1_I2;
  double __pyx_v_k2_I2;
  double __pyx_v_k3_I2;
  double __pyx_v_k4_I2;
  double __pyx_v_dI2;
  double __pyx_v_k1_I3;
  double __pyx_v_k2_I3;
  double __pyx_v_k3_I3;
  double __pyx_v_k4_I3;
  double __pyx_v_dI3;
  double __pyx_v_E_Na;
  double __pyx_v_I_Na;
  double __pyx_v_I_Nab;
  double __pyx_v_sigma;
  double __pyx_v_f_NaK;
  double __pyx_v_I_NaK;
  double __pyx_v_k1_Na_i;
  double __pyx_v_k2_Na_i;
  double __pyx_v_k3_Na_i;
  double __pyx_v_k4_Na_i;
  double __pyx_v_dNa_i;
  double __pyx_v_C_Na3;
  double __pyx_v_a_Na11;
  double __pyx_v_B_Na11;
  double __pyx_v_B_Na12;
  double __pyx_v_B_Na13;
  double __pyx_v_a_Na3;
  double __pyx_v_B_Na3;
  double __pyx_v_a_Na2;
  double __pyx_v_a_Na13;
  double __pyx_v_B_Na2;
  double __pyx_v_a_Na4;
  double __pyx_v_B_Na4;
  double __pyx_v_a_Na5;
  double __pyx_v_B_Na5;
  double __pyx_v_a_Na12;
  double __pyx_v_k1_C_Na2;
  double __pyx_v_k2_C_Na2;
  double __pyx_v_k3_C_Na2;
  double __pyx_v_k4_C_Na2;
  double __pyx_v_dC_Na2;
  double __pyx_v_k1_C_Na1;
  double __pyx_v_k2_C_Na1;
  double __pyx_v_k3_C_Na1;
  double __pyx_v_k4_C_Na1;
  double __pyx_v_dC_Na1;
  double __pyx_v_k1_O_Na;
  double __pyx_v_k2_O_Na;
  double __pyx_v_k3_O_Na;
  double __pyx_v_k4_O_Na;
  double __pyx_v_dO_Na;
  double __pyx_v_k1_I_FNa;
  double __pyx_v_k2_I_FNa;
  double __pyx_v_k3_I_FNa;
  double __pyx_v_k4_I_FNa;
  double __pyx_v_dIF_Na;
  double __pyx_v_k1_I1_Na;
  double __pyx_v_k2_I1_Na;
  double __pyx_v_k3_I1_Na;
  double __pyx_v_k4_I1_Na;
  double __pyx_v_dI1_Na;
  double __pyx_v_k1_I2_Na;
  double __pyx_v_k2_I2_Na;
  double __pyx_v_k3_I2_Na;
  double __pyx_v_k4_I2_Na;
  double __pyx_v_dI2_Na;
  double __pyx_v_k1_IC_Na2;
  double __pyx_v_k2_IC_Na2;
  double __pyx_v_k3_IC_Na2;
  double __pyx_v_k4_IC_Na2;
  double __pyx_v_dIC_Na2;
  double __pyx_v_k1_IC_Na3;
  double __pyx_v_k2_IC_Na3;
  double __pyx_v_k3_IC_Na3;
  double __pyx_v_k4_IC_Na3;
  double __pyx_v_dIC_Na3;
  double __pyx_v_a_a;
  double __pyx_v_B_a;
  double __pyx_v_a_i;
  double __pyx_v_B_i_Na;
  double __pyx_v_k1_a_tof;
  double __pyx_v_k2_a_tof;
  double __pyx_v_k3_a_tof;
  double __pyx_v_k4_a_tof;
  double __pyx_v_da_tof;
  double __pyx_v_k1_i_tof;
  double __pyx_v_k2_i_tof;
  double __pyx_v_k3_i_tof;
  double __pyx_v_k4_i_tof;
  double __pyx_v_di_tof;
  double __pyx_v_t_tas;
  double __pyx_v_a_ss;
  double __pyx_v_k1_a_tos;
  double __pyx_v_k2_a_tos;
  double __pyx_v_k3_a_tos;
  double __pyx_v_k4_a_tos;
  double __pyx_v_da_tos;
  double __pyx_v_t_tis;
  double __pyx_v_i_ss;
  double __pyx_v_k1_i_tos;
  double __pyx_v_k2_i_tos;
  double __pyx_v_k3_i_tos;
  double __pyx_v_k4_i_tos;
  double __pyx_v_di_tos;
  double __pyx_v_E_K;
  double __pyx_v_I_Ktof;
  double __pyx_v_I_Ktos;
  double __pyx_v_I_K1;
  double __pyx_v_t_Kss;
  double __pyx_v_k1_a_Kss;
  double __pyx_v_k2_a_Kss;
  double __pyx_v_k3_a_Kss;
  double __pyx_v_k4_a_Kss;
  double __pyx_v_da_Kss;
  double __pyx_v_I_Kss;
  double __pyx_v_t_aur;
  double __pyx_v_t_iur;
  double __pyx_v_a_n;
  double __pyx_v_B_n;
  double __pyx_v_k1_n_Ks;
  double __pyx_v_k2_n_Ks;
  double __pyx_v_k3_n_Ks;
  double __pyx_v_k4_n_Ks;
  double __pyx_v_dn_Ks;
  double __pyx_v_I_Ks;
  double __pyx_v_k1_a_ur;
  double __pyx_v_k2_a_ur;
  double __pyx_v_k3_a_ur;
  double __pyx_v_k4_a_ur;
  double __pyx_v_da_ur;
  double __pyx_v_k1_i_ur;
  double __pyx_v_k2_i_ur;
  double __pyx_v_k3_i_ur;
  double __pyx_v_k4_i_ur;
  double __pyx_v_di_ur;
  double __pyx_v_I_Kur;
  double __pyx_v_I_Kr;
  double __pyx_v_k1_K_i;
  double __pyx_v_k2_K_i;
  double __pyx_v_k3_K_i;
  double __pyx_v_k4_K_i;
  double __pyx_v_dK_i;
  double __pyx_v_C_K0;
  double __pyx_v_a_a0;
  double __pyx_v_B_a0;
  double __pyx_v_a_a1;
  double __pyx_v_B_a1;
  double __pyx_v_a_i_r;
  double __pyx_v_B_i_r;
  double __pyx_v_k1_C_K1;
  double __pyx_v_k2_C_K1;
  double __pyx_v_k3_C_K1;
  double __pyx_v_k4_C_K1;
  double __pyx_v_dC_K1;
  double __pyx_v_k1_C_K2;
  double __pyx_v_k2_C_K2;
  double __pyx_v_k3_C_K2;
  double __pyx_v_k4_C_K2;
  double __pyx_v_dC_K2;
  double __pyx_v_k1_O_K;
  double __pyx_v_k2_O_K;
  double __pyx_v_k3_O_K;
  double __pyx_v_k4_O_K;
  double __pyx_v_dO_K;
  double __pyx_v_k1_I_K;
  double __pyx_v_k2_I_K;
  double __pyx_v_k3_I_K;
  double __pyx_v_k4_I_K;
  double __pyx_v_dI_K;
  double __pyx_v_O_ClCa;
  double __pyx_v_I_ClCa;
  double __pyx_v_k1_V;
  double __pyx_v_k2_V;
  double __pyx_v_k3_V;
  double __pyx_v_k4_V;
  PyObject *__pyx_v_dV = NULL;
  PyObject *__pyx_r = NULL;
  __Pyx_RefNannyDeclarations
  __Pyx_RefNannySetupContext("local_step", 0);
  /* Check if called by wrapper */
  if (unlikely(__pyx_skip_dispatch)) ;
  /* Check if overridden in Python */
  else if (unlikely(Py_TYPE(((PyObject *)__pyx_v_self))->tp_dictoffset != 0)) {
    __pyx_t_1 = __Pyx_PyObject_GetAttrStr(((PyObject *)__pyx_v_self), __pyx_n_s_local_step); if (unlikely(!__pyx_t_1)) {__pyx_filename = __pyx_f[0]; __pyx_lineno = 285; __pyx_clineno = __LINE__; goto __pyx_L1_error;}
    __Pyx_GOTREF(__pyx_t_1);
    if (!PyCFunction_Check(__pyx_t_1) || (PyCFunction_GET_FUNCTION(__pyx_t_1) != (PyCFunction)__pyx_pw_5model_10Bondarenko_7local_step)) {
      __Pyx_XDECREF(__pyx_r);
      __pyx_t_2 = __Pyx_PyObject_Call(__pyx_t_1, __pyx_empty_tuple, NULL); if (unlikely(!__pyx_t_2)) {__pyx_filename = __pyx_f[0]; __pyx_lineno = 285; __pyx_clineno = __LINE__; goto __pyx_L1_error;}
      __Pyx_GOTREF(__pyx_t_2);
      __pyx_r = __pyx_t_2;
      __pyx_t_2 = 0;
      __Pyx_DECREF(__pyx_t_1); __pyx_t_1 = 0;
      goto __pyx_L0;
    }
    __Pyx_DECREF(__pyx_t_1); __pyx_t_1 = 0;
  }
/* … */
  /* function exit code */
  __pyx_r = Py_None; __Pyx_INCREF(Py_None);
  goto __pyx_L0;
  __pyx_L1_error:;
  __Pyx_XDECREF(__pyx_t_1);
  __Pyx_XDECREF(__pyx_t_2);
  __Pyx_XDECREF(__pyx_t_5);
  __Pyx_XDECREF(__pyx_t_6);
  __PYX_XDEC_MEMVIEW(&__pyx_t_7, 1);
  __PYX_XDEC_MEMVIEW(&__pyx_t_8, 1);
  __Pyx_XDECREF(__pyx_t_12);
  __Pyx_AddTraceback("model.Bondarenko.local_step", __pyx_clineno, __pyx_lineno, __pyx_filename);
  __pyx_r = 0;
  __pyx_L0:;
  __PYX_XDEC_MEMVIEW(&__pyx_v_dudx2, 1);
  __PYX_XDEC_MEMVIEW(&__pyx_v_dudy2, 1);
  __Pyx_XDECREF(__pyx_v_dV);
  __Pyx_XGIVEREF(__pyx_r);
  __Pyx_RefNannyFinishContext();
  return __pyx_r;
}

/* Python wrapper */
static PyObject *__pyx_pw_5model_10Bondarenko_7local_step(PyObject *__pyx_v_self, CYTHON_UNUSED PyObject *unused); /*proto*/
static PyObject *__pyx_pw_5model_10Bondarenko_7local_step(PyObject *__pyx_v_self, CYTHON_UNUSED PyObject *unused) {
  PyObject *__pyx_r = 0;
  __Pyx_RefNannyDeclarations
  __Pyx_RefNannySetupContext("local_step (wrapper)", 0);
  __pyx_r = __pyx_pf_5model_10Bondarenko_6local_step(((struct __pyx_obj_5model_Bondarenko *)__pyx_v_self));

  /* function exit code */
  __Pyx_RefNannyFinishContext();
  return __pyx_r;
}

static PyObject *__pyx_pf_5model_10Bondarenko_6local_step(struct __pyx_obj_5model_Bondarenko *__pyx_v_self) {
  PyObject *__pyx_r = NULL;
  __Pyx_RefNannyDeclarations
  __Pyx_RefNannySetupContext("local_step", 0);
  __Pyx_XDECREF(__pyx_r);
  __pyx_t_1 = ((struct __pyx_vtabstruct_5model_Bondarenko *)__pyx_v_self->__pyx_vtab)->local_step(__pyx_v_self, 1); if (unlikely(!__pyx_t_1)) {__pyx_filename = __pyx_f[0]; __pyx_lineno = 285; __pyx_clineno = __LINE__; goto __pyx_L1_error;}
  __Pyx_GOTREF(__pyx_t_1);
  __pyx_r = __pyx_t_1;
  __pyx_t_1 = 0;
  goto __pyx_L0;

  /* function exit code */
  __pyx_L1_error:;
  __Pyx_XDECREF(__pyx_t_1);
  __Pyx_AddTraceback("model.Bondarenko.local_step", __pyx_clineno, __pyx_lineno, __pyx_filename);
  __pyx_r = NULL;
  __pyx_L0:;
  __Pyx_XGIVEREF(__pyx_r);
  __Pyx_RefNannyFinishContext();
  return __pyx_r;
}
```

```
 286:
```

```
 287:         cdef int nx = self.V.shape[0]
```

```
  __pyx_t_1 = __Pyx_PyObject_GetAttrStr(((PyObject *)__pyx_v_self), __pyx_n_s_V); if (unlikely(!__pyx_t_1)) {__pyx_filename = __pyx_f[0]; __pyx_lineno = 287; __pyx_clineno = __LINE__; goto __pyx_L1_error;}
  __Pyx_GOTREF(__pyx_t_1);
  __pyx_t_2 = __Pyx_PyObject_GetAttrStr(__pyx_t_1, __pyx_n_s_shape); if (unlikely(!__pyx_t_2)) {__pyx_filename = __pyx_f[0]; __pyx_lineno = 287; __pyx_clineno = __LINE__; goto __pyx_L1_error;}
  __Pyx_GOTREF(__pyx_t_2);
  __Pyx_DECREF(__pyx_t_1); __pyx_t_1 = 0;
  __pyx_t_1 = __Pyx_GetItemInt(__pyx_t_2, 0, long, 1, __Pyx_PyInt_From_long, 0, 0, 0); if (unlikely(__pyx_t_1 == NULL)) {__pyx_filename = __pyx_f[0]; __pyx_lineno = 287; __pyx_clineno = __LINE__; goto __pyx_L1_error;};
  __Pyx_GOTREF(__pyx_t_1);
  __Pyx_DECREF(__pyx_t_2); __pyx_t_2 = 0;
  __pyx_t_3 = __Pyx_PyInt_As_int(__pyx_t_1); if (unlikely((__pyx_t_3 == (int)-1) && PyErr_Occurred())) {__pyx_filename = __pyx_f[0]; __pyx_lineno = 287; __pyx_clineno = __LINE__; goto __pyx_L1_error;}
  __Pyx_DECREF(__pyx_t_1); __pyx_t_1 = 0;
  __pyx_v_nx = __pyx_t_3;
```

```
 288:         cdef int ny = self.V.shape[1]
```

```
  __pyx_t_1 = __Pyx_PyObject_GetAttrStr(((PyObject *)__pyx_v_self), __pyx_n_s_V); if (unlikely(!__pyx_t_1)) {__pyx_filename = __pyx_f[0]; __pyx_lineno = 288; __pyx_clineno = __LINE__; goto __pyx_L1_error;}
  __Pyx_GOTREF(__pyx_t_1);
  __pyx_t_2 = __Pyx_PyObject_GetAttrStr(__pyx_t_1, __pyx_n_s_shape); if (unlikely(!__pyx_t_2)) {__pyx_filename = __pyx_f[0]; __pyx_lineno = 288; __pyx_clineno = __LINE__; goto __pyx_L1_error;}
  __Pyx_GOTREF(__pyx_t_2);
  __Pyx_DECREF(__pyx_t_1); __pyx_t_1 = 0;
  __pyx_t_1 = __Pyx_GetItemInt(__pyx_t_2, 1, long, 1, __Pyx_PyInt_From_long, 0, 0, 0); if (unlikely(__pyx_t_1 == NULL)) {__pyx_filename = __pyx_f[0]; __pyx_lineno = 288; __pyx_clineno = __LINE__; goto __pyx_L1_error;};
  __Pyx_GOTREF(__pyx_t_1);
  __Pyx_DECREF(__pyx_t_2); __pyx_t_2 = 0;
  __pyx_t_3 = __Pyx_PyInt_As_int(__pyx_t_1); if (unlikely((__pyx_t_3 == (int)-1) && PyErr_Occurred())) {__pyx_filename = __pyx_f[0]; __pyx_lineno = 288; __pyx_clineno = __LINE__; goto __pyx_L1_error;}
  __Pyx_DECREF(__pyx_t_1); __pyx_t_1 = 0;
  __pyx_v_ny = __pyx_t_3;
```

```
 289:
```

```
 290:         cdef double A_cap = self.A_cap
```

```
  __pyx_t_4 = __pyx_v_self->A_cap;
  __pyx_v_A_cap = __pyx_t_4;
```

```
 291:         cdef double V_myo = self.V_myo
```

```
  __pyx_t_4 = __pyx_v_self->V_myo;
  __pyx_v_V_myo = __pyx_t_4;
```

```
 292:         cdef double V_JSR = self.V_JSR
```

```
  __pyx_t_4 = __pyx_v_self->V_JSR;
  __pyx_v_V_JSR = __pyx_t_4;
```

```
 293:         cdef double V_NSR = self.V_NSR
```

```
  __pyx_t_4 = __pyx_v_self->V_NSR;
  __pyx_v_V_NSR = __pyx_t_4;
```

```
 294:         cdef double V_ss  = self.V_ss
```

```
  __pyx_t_4 = __pyx_v_self->V_ss;
  __pyx_v_V_ss = __pyx_t_4;
```

```
 295:         cdef double K_0   = self.K_0
```

```
  __pyx_t_4 = __pyx_v_self->K_0;
  __pyx_v_K_0 = __pyx_t_4;
```

```
 296:         cdef double Na_0  = self.Na_0
```

```
  __pyx_t_4 = __pyx_v_self->Na_0;
  __pyx_v_Na_0 = __pyx_t_4;
```

```
 297:         cdef double Ca_0  = self.Ca_0
```

```
  __pyx_t_4 = __pyx_v_self->Ca_0;
  __pyx_v_Ca_0 = __pyx_t_4;
```

```
 298:         cdef double v_1    = self.v_1
```

```
  __pyx_t_4 = __pyx_v_self->v_1;
  __pyx_v_v_1 = __pyx_t_4;
```

```
 299:         cdef double v_2    = self.v_2
```

```
  __pyx_t_4 = __pyx_v_self->v_2;
  __pyx_v_v_2 = __pyx_t_4;
```

```
 300:         cdef double v_3    = self.v_3
```

```
  __pyx_t_4 = __pyx_v_self->v_3;
  __pyx_v_v_3 = __pyx_t_4;
```

```
 301:         cdef double k_mup  = self.k_mup
```

```
  __pyx_t_4 = __pyx_v_self->k_mup;
  __pyx_v_k_mup = __pyx_t_4;
```

```
 302:         cdef double t_tr   = self.t_tr
```

```
  __pyx_t_4 = __pyx_v_self->t_tr;
  __pyx_v_t_tr = __pyx_t_4;
```

```
 303:         cdef double t_xfer = self.t_xfer
```

```
  __pyx_t_4 = __pyx_v_self->t_xfer;
  __pyx_v_t_xfer = __pyx_t_4;
```

```
 304:         cdef double k_p_a  = self.k_p_a
```

```
  __pyx_t_4 = __pyx_v_self->k_p_a;
  __pyx_v_k_p_a = __pyx_t_4;
```

```
 305:         cdef double k_n_a  = self.k_n_a
```

```
  __pyx_t_4 = __pyx_v_self->k_n_a;
  __pyx_v_k_n_a = __pyx_t_4;
```

```
 306:         cdef double k_p_b  = self.k_p_b
```

```
  __pyx_t_4 = __pyx_v_self->k_p_b;
  __pyx_v_k_p_b = __pyx_t_4;
```

```
 307:         cdef double k_n_b  = self.k_n_b
```

```
  __pyx_t_4 = __pyx_v_self->k_n_b;
  __pyx_v_k_n_b = __pyx_t_4;
```

```
 308:         cdef double k_p_c  = self.k_p_c
```

```
  __pyx_t_4 = __pyx_v_self->k_p_c;
  __pyx_v_k_p_c = __pyx_t_4;
```

```
 309:         cdef double k_n_c  = self.k_n_c
```

```
  __pyx_t_4 = __pyx_v_self->k_n_c;
  __pyx_v_k_n_c = __pyx_t_4;
```

```
 310:         cdef double n      = self.n
```

```
  __pyx_t_4 = __pyx_v_self->n;
  __pyx_v_n = __pyx_t_4;
```

```
 311:         cdef double m      = self.m
```

```
  __pyx_t_4 = __pyx_v_self->m;
  __pyx_v_m = __pyx_t_4;
```

```
 312:         cdef double G_CaL     = self.G_CaL
```

```
  __pyx_t_4 = __pyx_v_self->G_CaL;
  __pyx_v_G_CaL = __pyx_t_4;
```

```
 313:         cdef double E_CaL     = self.E_CaL
```

```
  __pyx_t_4 = __pyx_v_self->E_CaL;
  __pyx_v_E_CaL = __pyx_t_4;
```

```
 314:         cdef double k_pcmax   = self.k_pcmax
```

```
  __pyx_t_4 = __pyx_v_self->k_pcmax;
  __pyx_v_k_pcmax = __pyx_t_4;
```

```
 315:         cdef double k_pchalf  = self.k_pchalf
```

```
  __pyx_t_4 = __pyx_v_self->k_pchalf;
  __pyx_v_k_pchalf = __pyx_t_4;
```

```
 316:         cdef double k_pcb     = self.k_pcb
```

```
  __pyx_t_4 = __pyx_v_self->k_pcb;
  __pyx_v_k_pcb = __pyx_t_4;
```

```
 317:         cdef double I_CaLmax  = self.I_CaLmax
```

```
  __pyx_t_4 = __pyx_v_self->I_CaLmax;
  __pyx_v_I_CaLmax = __pyx_t_4;
```

```
 318:         cdef double LTRPN_tot = self.LTRPN_tot
```

```
  __pyx_t_4 = __pyx_v_self->LTRPN_tot;
  __pyx_v_LTRPN_tot = __pyx_t_4;
```

```
 319:         cdef double HTRPN_tot = self.HTRPN_tot
```

```
  __pyx_t_4 = __pyx_v_self->HTRPN_tot;
  __pyx_v_HTRPN_tot = __pyx_t_4;
```

```
 320:         cdef double k_p_htrpn = self.k_p_htrpn
```

```
  __pyx_t_4 = __pyx_v_self->k_p_htrpn;
  __pyx_v_k_p_htrpn = __pyx_t_4;
```

```
 321:         cdef double k_n_htrpn = self.k_n_htrpn
```

```
  __pyx_t_4 = __pyx_v_self->k_n_htrpn;
  __pyx_v_k_n_htrpn = __pyx_t_4;
```

```
 322:         cdef double k_p_ltrpn = self.k_p_ltrpn
```

```
  __pyx_t_4 = __pyx_v_self->k_p_ltrpn;
  __pyx_v_k_p_ltrpn = __pyx_t_4;
```

```
 323:         cdef double k_n_ltrpn = self.k_n_ltrpn
```

```
  __pyx_t_4 = __pyx_v_self->k_n_ltrpn;
  __pyx_v_k_n_ltrpn = __pyx_t_4;
```

```
 324:         cdef double CMDN_tot  = self.CMDN_tot
```

```
  __pyx_t_4 = __pyx_v_self->CMDN_tot;
  __pyx_v_CMDN_tot = __pyx_t_4;
```

```
 325:         cdef double CSQN_tot  = self.CSQN_tot
```

```
  __pyx_t_4 = __pyx_v_self->CSQN_tot;
  __pyx_v_CSQN_tot = __pyx_t_4;
```

```
 326:         cdef double k_CMDN_m  = self.k_CMDN_m
```

```
  __pyx_t_4 = __pyx_v_self->k_CMDN_m;
  __pyx_v_k_CMDN_m = __pyx_t_4;
```

```
 327:         cdef double k_CSQN_m  = self.k_CSQN_m
```

```
  __pyx_t_4 = __pyx_v_self->k_CSQN_m;
  __pyx_v_k_CSQN_m = __pyx_t_4;
```

```
 328:         cdef double C_m       = self.C_m
```

```
  __pyx_t_4 = __pyx_v_self->C_m;
  __pyx_v_C_m = __pyx_t_4;
```

```
 329:         cdef double F         = self.F
```

```
  __pyx_t_4 = __pyx_v_self->F;
  __pyx_v_F = __pyx_t_4;
```

```
 330:         cdef double T         = self.T
```

```
  __pyx_t_4 = __pyx_v_self->T;
  __pyx_v_T = __pyx_t_4;
```

```
 331:         cdef double R         = self.R
```

```
  __pyx_t_4 = __pyx_v_self->R;
  __pyx_v_R = __pyx_t_4;
```

```
 332:         cdef double k_NaCa    = self.k_NaCa
```

```
  __pyx_t_4 = __pyx_v_self->k_NaCa;
  __pyx_v_k_NaCa = __pyx_t_4;
```

```
 333:         cdef double k_mNa     = self.k_mNa
```

```
  __pyx_t_4 = __pyx_v_self->k_mNa;
  __pyx_v_k_mNa = __pyx_t_4;
```

```
 334:         cdef double k_mCa     = self.k_mCa
```

```
  __pyx_t_4 = __pyx_v_self->k_mCa;
  __pyx_v_k_mCa = __pyx_t_4;
```

```
 335:         cdef double k_sat     = self.k_sat
```

```
  __pyx_t_4 = __pyx_v_self->k_sat;
  __pyx_v_k_sat = __pyx_t_4;
```

```
 336:         cdef double miu       = self.miu
```

```
  __pyx_t_4 = __pyx_v_self->miu;
  __pyx_v_miu = __pyx_t_4;
```

```
 337:         cdef double I_maxNaK  = self.I_maxNaK
```

```
  __pyx_t_4 = __pyx_v_self->I_maxNaK;
  __pyx_v_I_maxNaK = __pyx_t_4;
```

```
 338:         cdef double K_mNai    = self.K_mNai
```

```
  __pyx_t_4 = __pyx_v_self->K_mNai;
  __pyx_v_K_mNai = __pyx_t_4;
```

```
 339:         cdef double K_mKo     = self.K_mKo
```

```
  __pyx_t_4 = __pyx_v_self->K_mKo;
  __pyx_v_K_mKo = __pyx_t_4;
```

```
 340:         cdef double I_maxpCa  = self.I_maxpCa
```

```
  __pyx_t_4 = __pyx_v_self->I_maxpCa;
  __pyx_v_I_maxpCa = __pyx_t_4;
```

```
 341:         cdef double k_mpCa    = self.k_mpCa
```

```
  __pyx_t_4 = __pyx_v_self->k_mpCa;
  __pyx_v_k_mpCa = __pyx_t_4;
```

```
 342:         cdef double G_Cab     = self.G_Cab
```

```
  __pyx_t_4 = __pyx_v_self->G_Cab;
  __pyx_v_G_Cab = __pyx_t_4;
```

```
 343:         cdef double G_Na      = self.G_Na
```

```
  __pyx_t_4 = __pyx_v_self->G_Na;
  __pyx_v_G_Na = __pyx_t_4;
```

```
 344:         cdef double G_Nab     = self.G_Nab
```

```
  __pyx_t_4 = __pyx_v_self->G_Nab;
  __pyx_v_G_Nab = __pyx_t_4;
```

```
 345:         cdef double G_Ktof    = self.G_Ktof      #(apex)
```

```
  __pyx_t_4 = __pyx_v_self->G_Ktof;
  __pyx_v_G_Ktof = __pyx_t_4;
```

```
 346:         #cdef double G_Ktof    = self.           #(septum)
```

```
 347:         cdef double G_Ks      = self.G_Ks
```

```
  __pyx_t_4 = __pyx_v_self->G_Ks;
  __pyx_v_G_Ks = __pyx_t_4;
```

```
 348:         cdef double G_Ktos    = self.G_Ktos      #(apex)
```

```
  __pyx_t_4 = __pyx_v_self->G_Ktos;
  __pyx_v_G_Ktos = __pyx_t_4;
```

```
 349:         cdef double G_Kur     = self.G_Kur       #(apex)
```

```
  __pyx_t_4 = __pyx_v_self->G_Kur;
  __pyx_v_G_Kur = __pyx_t_4;
```

```
 350:         cdef double G_Kss     = self.G_Kss       #(apex)
```

```
  __pyx_t_4 = __pyx_v_self->G_Kss;
  __pyx_v_G_Kss = __pyx_t_4;
```

```
 351:         #cdef double G_Ktos    = self.           #(septum)
```

```
 352:         #cdef double G_Kur     = self.           #(septum)
```

```
 353:         #cdef double G_Kss     = self.           #(septum)
```

```
 354:         cdef double G_Kr      = self.G_Kr
```

```
  __pyx_t_4 = __pyx_v_self->G_Kr;
  __pyx_v_G_Kr = __pyx_t_4;
```

```
 355:         cdef double k_f       = self.k_f
```

```
  __pyx_t_4 = __pyx_v_self->k_f;
  __pyx_v_k_f = __pyx_t_4;
```

```
 356:         cdef double k_b       = self.k_b
```

```
  __pyx_t_4 = __pyx_v_self->k_b;
  __pyx_v_k_b = __pyx_t_4;
```

```
 357:         cdef double G_ClCa    = self.G_ClCa
```

```
  __pyx_t_4 = __pyx_v_self->G_ClCa;
  __pyx_v_G_ClCa = __pyx_t_4;
```

```
 358:         cdef double K_mCl     = self.K_mCl
```

```
  __pyx_t_4 = __pyx_v_self->K_mCl;
  __pyx_v_K_mCl = __pyx_t_4;
```

```
 359:         cdef double E_Cl      = self.E_Cl
```

```
  __pyx_t_4 = __pyx_v_self->E_Cl;
  __pyx_v_E_Cl = __pyx_t_4;
```

```
 360:         cdef double Laplas_c = self.Laplas_c
```

```
  __pyx_t_4 = __pyx_v_self->Laplas_c;
  __pyx_v_Laplas_c = __pyx_t_4;
```

```
 361:         cdef double Laplas_r = self.Laplas_r
```

```
  __pyx_t_4 = __pyx_v_self->Laplas_r;
  __pyx_v_Laplas_r = __pyx_t_4;
```

```
 362:         cdef double V_ChR2 = self.V_ChR2
```

```
  __pyx_t_4 = __pyx_v_self->V_ChR2;
  __pyx_v_V_ChR2 = __pyx_t_4;
```

```
 363:         cdef double i_Kss = self.i_Kss
```

```
  __pyx_t_4 = __pyx_v_self->i_Kss;
  __pyx_v_i_Kss = __pyx_t_4;
```

```
 364:         cdef double h = self.h
```

```
  __pyx_t_4 = __pyx_v_self->h;
  __pyx_v_h = __pyx_t_4;
```

```
 365:         cdef double I_stim = 0
```

```
  __pyx_v_I_stim = 0.0;
```

```
 366:
```

```
 367:         cdef double V
```

```
 368:         cdef double Ca2_i
```

```
 369:         cdef double Ca2_ss
```

```
 370:         cdef double Ca2_JSR
```

```
 371:         cdef double Ca2_NSR
```

```
 372:         cdef double LTRPNCa
```

```
 373:         cdef double HTRPNCa
```

```
 374:         cdef double O
```

```
 375:         cdef double C2
```

```
 376:         cdef double C3
```

```
 377:         cdef double C4
```

```
 378:         cdef double I1
```

```
 379:         cdef double I2
```

```
 380:         cdef double I3
```

```
 381:         cdef double Pc2
```

```
 382:         cdef double Po1
```

```
 383:         cdef double Po2
```

```
 384:         cdef double P_RyR
```

```
 385:         cdef double C_Na2
```

```
 386:         cdef double C_Na1
```

```
 387:         cdef double O_Na
```

```
 388:         cdef double IF_Na
```

```
 389:         cdef double I1_Na
```

```
 390:         cdef double I2_Na
```

```
 391:         cdef double IC_Na2
```

```
 392:         cdef double IC_Na3
```

```
 393:         cdef double Na_i
```

```
 394:         cdef double K_i
```

```
 395:         cdef double a_tof
```

```
 396:         cdef double i_tof
```

```
 397:         cdef double n_Ks
```

```
 398:         cdef double a_tos
```

```
 399:         cdef double i_tos
```

```
 400:         cdef double a_ur
```

```
 401:         cdef double i_ur
```

```
 402:         cdef double a_Kss
```

```
 403:         cdef double C_K1
```

```
 404:         cdef double C_K2
```

```
 405:         cdef double O_K
```

```
 406:         cdef double I_K
```

```
 407:
```

```
 408:         cdef int j, k, x, y
```

```
 409:
```

```
 410:         cdef double  [:,::1] dudx2 = np.empty((nx, ny))
```

```
  __pyx_t_1 = __Pyx_GetModuleGlobalName(__pyx_n_s_np); if (unlikely(!__pyx_t_1)) {__pyx_filename = __pyx_f[0]; __pyx_lineno = 410; __pyx_clineno = __LINE__; goto __pyx_L1_error;}
  __Pyx_GOTREF(__pyx_t_1);
  __pyx_t_2 = __Pyx_PyObject_GetAttrStr(__pyx_t_1, __pyx_n_s_empty); if (unlikely(!__pyx_t_2)) {__pyx_filename = __pyx_f[0]; __pyx_lineno = 410; __pyx_clineno = __LINE__; goto __pyx_L1_error;}
  __Pyx_GOTREF(__pyx_t_2);
  __Pyx_DECREF(__pyx_t_1); __pyx_t_1 = 0;
  __pyx_t_1 = __Pyx_PyInt_From_int(__pyx_v_nx); if (unlikely(!__pyx_t_1)) {__pyx_filename = __pyx_f[0]; __pyx_lineno = 410; __pyx_clineno = __LINE__; goto __pyx_L1_error;}
  __Pyx_GOTREF(__pyx_t_1);
  __pyx_t_5 = __Pyx_PyInt_From_int(__pyx_v_ny); if (unlikely(!__pyx_t_5)) {__pyx_filename = __pyx_f[0]; __pyx_lineno = 410; __pyx_clineno = __LINE__; goto __pyx_L1_error;}
  __Pyx_GOTREF(__pyx_t_5);
  __pyx_t_6 = PyTuple_New(2); if (unlikely(!__pyx_t_6)) {__pyx_filename = __pyx_f[0]; __pyx_lineno = 410; __pyx_clineno = __LINE__; goto __pyx_L1_error;}
  __Pyx_GOTREF(__pyx_t_6);
  PyTuple_SET_ITEM(__pyx_t_6, 0, __pyx_t_1);
  __Pyx_GIVEREF(__pyx_t_1);
  PyTuple_SET_ITEM(__pyx_t_6, 1, __pyx_t_5);
  __Pyx_GIVEREF(__pyx_t_5);
  __pyx_t_1 = 0;
  __pyx_t_5 = 0;
  __pyx_t_5 = PyTuple_New(1); if (unlikely(!__pyx_t_5)) {__pyx_filename = __pyx_f[0]; __pyx_lineno = 410; __pyx_clineno = __LINE__; goto __pyx_L1_error;}
  __Pyx_GOTREF(__pyx_t_5);
  PyTuple_SET_ITEM(__pyx_t_5, 0, __pyx_t_6);
  __Pyx_GIVEREF(__pyx_t_6);
  __pyx_t_6 = 0;
  __pyx_t_6 = __Pyx_PyObject_Call(__pyx_t_2, __pyx_t_5, NULL); if (unlikely(!__pyx_t_6)) {__pyx_filename = __pyx_f[0]; __pyx_lineno = 410; __pyx_clineno = __LINE__; goto __pyx_L1_error;}
  __Pyx_GOTREF(__pyx_t_6);
  __Pyx_DECREF(__pyx_t_2); __pyx_t_2 = 0;
  __Pyx_DECREF(__pyx_t_5); __pyx_t_5 = 0;
  __pyx_t_7 = __Pyx_PyObject_to_MemoryviewSlice_d_dc_double(__pyx_t_6);
  if (unlikely(!__pyx_t_7.memview)) {__pyx_filename = __pyx_f[0]; __pyx_lineno = 410; __pyx_clineno = __LINE__; goto __pyx_L1_error;}
  __Pyx_DECREF(__pyx_t_6); __pyx_t_6 = 0;
  __pyx_v_dudx2 = __pyx_t_7;
  __pyx_t_7.memview = NULL;
  __pyx_t_7.data = NULL;
```

```
 411:         cdef double  [:,::1] dudy2 = np.empty((nx, ny))
```

```
  __pyx_t_6 = __Pyx_GetModuleGlobalName(__pyx_n_s_np); if (unlikely(!__pyx_t_6)) {__pyx_filename = __pyx_f[0]; __pyx_lineno = 411; __pyx_clineno = __LINE__; goto __pyx_L1_error;}
  __Pyx_GOTREF(__pyx_t_6);
  __pyx_t_5 = __Pyx_PyObject_GetAttrStr(__pyx_t_6, __pyx_n_s_empty); if (unlikely(!__pyx_t_5)) {__pyx_filename = __pyx_f[0]; __pyx_lineno = 411; __pyx_clineno = __LINE__; goto __pyx_L1_error;}
  __Pyx_GOTREF(__pyx_t_5);
  __Pyx_DECREF(__pyx_t_6); __pyx_t_6 = 0;
  __pyx_t_6 = __Pyx_PyInt_From_int(__pyx_v_nx); if (unlikely(!__pyx_t_6)) {__pyx_filename = __pyx_f[0]; __pyx_lineno = 411; __pyx_clineno = __LINE__; goto __pyx_L1_error;}
  __Pyx_GOTREF(__pyx_t_6);
  __pyx_t_2 = __Pyx_PyInt_From_int(__pyx_v_ny); if (unlikely(!__pyx_t_2)) {__pyx_filename = __pyx_f[0]; __pyx_lineno = 411; __pyx_clineno = __LINE__; goto __pyx_L1_error;}
  __Pyx_GOTREF(__pyx_t_2);
  __pyx_t_1 = PyTuple_New(2); if (unlikely(!__pyx_t_1)) {__pyx_filename = __pyx_f[0]; __pyx_lineno = 411; __pyx_clineno = __LINE__; goto __pyx_L1_error;}
  __Pyx_GOTREF(__pyx_t_1);
  PyTuple_SET_ITEM(__pyx_t_1, 0, __pyx_t_6);
  __Pyx_GIVEREF(__pyx_t_6);
  PyTuple_SET_ITEM(__pyx_t_1, 1, __pyx_t_2);
  __Pyx_GIVEREF(__pyx_t_2);
  __pyx_t_6 = 0;
  __pyx_t_2 = 0;
  __pyx_t_2 = PyTuple_New(1); if (unlikely(!__pyx_t_2)) {__pyx_filename = __pyx_f[0]; __pyx_lineno = 411; __pyx_clineno = __LINE__; goto __pyx_L1_error;}
  __Pyx_GOTREF(__pyx_t_2);
  PyTuple_SET_ITEM(__pyx_t_2, 0, __pyx_t_1);
  __Pyx_GIVEREF(__pyx_t_1);
  __pyx_t_1 = 0;
  __pyx_t_1 = __Pyx_PyObject_Call(__pyx_t_5, __pyx_t_2, NULL); if (unlikely(!__pyx_t_1)) {__pyx_filename = __pyx_f[0]; __pyx_lineno = 411; __pyx_clineno = __LINE__; goto __pyx_L1_error;}
  __Pyx_GOTREF(__pyx_t_1);
  __Pyx_DECREF(__pyx_t_5); __pyx_t_5 = 0;
  __Pyx_DECREF(__pyx_t_2); __pyx_t_2 = 0;
  __pyx_t_8 = __Pyx_PyObject_to_MemoryviewSlice_d_dc_double(__pyx_t_1);
  if (unlikely(!__pyx_t_8.memview)) {__pyx_filename = __pyx_f[0]; __pyx_lineno = 411; __pyx_clineno = __LINE__; goto __pyx_L1_error;}
  __Pyx_DECREF(__pyx_t_1); __pyx_t_1 = 0;
  __pyx_v_dudy2 = __pyx_t_8;
  __pyx_t_8.memview = NULL;
  __pyx_t_8.data = NULL;
```

```
 412:
```

```
 413:
```

```
 414:         for j in range (1, nx-1):
```

```
  __pyx_t_9 = (__pyx_v_nx - 1);
  for (__pyx_t_3 = 1; __pyx_t_3 < __pyx_t_9; __pyx_t_3+=1) {
    __pyx_v_j = __pyx_t_3;
```

```
 415:             for k in range (1, ny-1):
```

```
    __pyx_t_10 = (__pyx_v_ny - 1);
    for (__pyx_t_11 = 1; __pyx_t_11 < __pyx_t_10; __pyx_t_11+=1) {
      __pyx_v_k = __pyx_t_11;
```

```
 416:
```

```
 417:
```

```
 418:                 dudx2[j,k] = (self.V[j+1,k]-2.*self.V[j,k]+self.V[j-1,k])/(self.dx**2)
```

```
      __pyx_t_1 = __Pyx_PyObject_GetAttrStr(((PyObject *)__pyx_v_self), __pyx_n_s_V); if (unlikely(!__pyx_t_1)) {__pyx_filename = __pyx_f[0]; __pyx_lineno = 418; __pyx_clineno = __LINE__; goto __pyx_L1_error;}
      __Pyx_GOTREF(__pyx_t_1);
      __pyx_t_2 = __Pyx_PyInt_From_long((__pyx_v_j + 1)); if (unlikely(!__pyx_t_2)) {__pyx_filename = __pyx_f[0]; __pyx_lineno = 418; __pyx_clineno = __LINE__; goto __pyx_L1_error;}
      __Pyx_GOTREF(__pyx_t_2);
      __pyx_t_5 = __Pyx_PyInt_From_int(__pyx_v_k); if (unlikely(!__pyx_t_5)) {__pyx_filename = __pyx_f[0]; __pyx_lineno = 418; __pyx_clineno = __LINE__; goto __pyx_L1_error;}
      __Pyx_GOTREF(__pyx_t_5);
      __pyx_t_6 = PyTuple_New(2); if (unlikely(!__pyx_t_6)) {__pyx_filename = __pyx_f[0]; __pyx_lineno = 418; __pyx_clineno = __LINE__; goto __pyx_L1_error;}
      __Pyx_GOTREF(__pyx_t_6);
      PyTuple_SET_ITEM(__pyx_t_6, 0, __pyx_t_2);
      __Pyx_GIVEREF(__pyx_t_2);
      PyTuple_SET_ITEM(__pyx_t_6, 1, __pyx_t_5);
      __Pyx_GIVEREF(__pyx_t_5);
      __pyx_t_2 = 0;
      __pyx_t_5 = 0;
      __pyx_t_5 = PyObject_GetItem(__pyx_t_1, __pyx_t_6); if (unlikely(__pyx_t_5 == NULL)) {__pyx_filename = __pyx_f[0]; __pyx_lineno = 418; __pyx_clineno = __LINE__; goto __pyx_L1_error;};
      __Pyx_GOTREF(__pyx_t_5);
      __Pyx_DECREF(__pyx_t_1); __pyx_t_1 = 0;
      __Pyx_DECREF(__pyx_t_6); __pyx_t_6 = 0;
      __pyx_t_6 = __Pyx_PyObject_GetAttrStr(((PyObject *)__pyx_v_self), __pyx_n_s_V); if (unlikely(!__pyx_t_6)) {__pyx_filename = __pyx_f[0]; __pyx_lineno = 418; __pyx_clineno = __LINE__; goto __pyx_L1_error;}
      __Pyx_GOTREF(__pyx_t_6);
      __pyx_t_1 = __Pyx_PyInt_From_int(__pyx_v_j); if (unlikely(!__pyx_t_1)) {__pyx_filename = __pyx_f[0]; __pyx_lineno = 418; __pyx_clineno = __LINE__; goto __pyx_L1_error;}
      __Pyx_GOTREF(__pyx_t_1);
      __pyx_t_2 = __Pyx_PyInt_From_int(__pyx_v_k); if (unlikely(!__pyx_t_2)) {__pyx_filename = __pyx_f[0]; __pyx_lineno = 418; __pyx_clineno = __LINE__; goto __pyx_L1_error;}
      __Pyx_GOTREF(__pyx_t_2);
      __pyx_t_12 = PyTuple_New(2); if (unlikely(!__pyx_t_12)) {__pyx_filename = __pyx_f[0]; __pyx_lineno = 418; __pyx_clineno = __LINE__; goto __pyx_L1_error;}
      __Pyx_GOTREF(__pyx_t_12);
      PyTuple_SET_ITEM(__pyx_t_12, 0, __pyx_t_1);
      __Pyx_GIVEREF(__pyx_t_1);
      PyTuple_SET_ITEM(__pyx_t_12, 1, __pyx_t_2);
      __Pyx_GIVEREF(__pyx_t_2);
      __pyx_t_1 = 0;
      __pyx_t_2 = 0;
      __pyx_t_2 = PyObject_GetItem(__pyx_t_6, __pyx_t_12); if (unlikely(__pyx_t_2 == NULL)) {__pyx_filename = __pyx_f[0]; __pyx_lineno = 418; __pyx_clineno = __LINE__; goto __pyx_L1_error;};
      __Pyx_GOTREF(__pyx_t_2);
      __Pyx_DECREF(__pyx_t_6); __pyx_t_6 = 0;
      __Pyx_DECREF(__pyx_t_12); __pyx_t_12 = 0;
      __pyx_t_12 = PyNumber_Multiply(__pyx_float_2_, __pyx_t_2); if (unlikely(!__pyx_t_12)) {__pyx_filename = __pyx_f[0]; __pyx_lineno = 418; __pyx_clineno = __LINE__; goto __pyx_L1_error;}
      __Pyx_GOTREF(__pyx_t_12);
      __Pyx_DECREF(__pyx_t_2); __pyx_t_2 = 0;
      __pyx_t_2 = PyNumber_Subtract(__pyx_t_5, __pyx_t_12); if (unlikely(!__pyx_t_2)) {__pyx_filename = __pyx_f[0]; __pyx_lineno = 418; __pyx_clineno = __LINE__; goto __pyx_L1_error;}
      __Pyx_GOTREF(__pyx_t_2);
      __Pyx_DECREF(__pyx_t_5); __pyx_t_5 = 0;
      __Pyx_DECREF(__pyx_t_12); __pyx_t_12 = 0;
      __pyx_t_12 = __Pyx_PyObject_GetAttrStr(((PyObject *)__pyx_v_self), __pyx_n_s_V); if (unlikely(!__pyx_t_12)) {__pyx_filename = __pyx_f[0]; __pyx_lineno = 418; __pyx_clineno = __LINE__; goto __pyx_L1_error;}
      __Pyx_GOTREF(__pyx_t_12);
      __pyx_t_5 = __Pyx_PyInt_From_long((__pyx_v_j - 1)); if (unlikely(!__pyx_t_5)) {__pyx_filename = __pyx_f[0]; __pyx_lineno = 418; __pyx_clineno = __LINE__; goto __pyx_L1_error;}
      __Pyx_GOTREF(__pyx_t_5);
      __pyx_t_6 = __Pyx_PyInt_From_int(__pyx_v_k); if (unlikely(!__pyx_t_6)) {__pyx_filename = __pyx_f[0]; __pyx_lineno = 418; __pyx_clineno = __LINE__; goto __pyx_L1_error;}
      __Pyx_GOTREF(__pyx_t_6);
      __pyx_t_1 = PyTuple_New(2); if (unlikely(!__pyx_t_1)) {__pyx_filename = __pyx_f[0]; __pyx_lineno = 418; __pyx_clineno = __LINE__; goto __pyx_L1_error;}
      __Pyx_GOTREF(__pyx_t_1);
      PyTuple_SET_ITEM(__pyx_t_1, 0, __pyx_t_5);
      __Pyx_GIVEREF(__pyx_t_5);
      PyTuple_SET_ITEM(__pyx_t_1, 1, __pyx_t_6);
      __Pyx_GIVEREF(__pyx_t_6);
      __pyx_t_5 = 0;
      __pyx_t_6 = 0;
      __pyx_t_6 = PyObject_GetItem(__pyx_t_12, __pyx_t_1); if (unlikely(__pyx_t_6 == NULL)) {__pyx_filename = __pyx_f[0]; __pyx_lineno = 418; __pyx_clineno = __LINE__; goto __pyx_L1_error;};
      __Pyx_GOTREF(__pyx_t_6);
      __Pyx_DECREF(__pyx_t_12); __pyx_t_12 = 0;
      __Pyx_DECREF(__pyx_t_1); __pyx_t_1 = 0;
      __pyx_t_1 = PyNumber_Add(__pyx_t_2, __pyx_t_6); if (unlikely(!__pyx_t_1)) {__pyx_filename = __pyx_f[0]; __pyx_lineno = 418; __pyx_clineno = __LINE__; goto __pyx_L1_error;}
      __Pyx_GOTREF(__pyx_t_1);
      __Pyx_DECREF(__pyx_t_2); __pyx_t_2 = 0;
      __Pyx_DECREF(__pyx_t_6); __pyx_t_6 = 0;
      __pyx_t_6 = PyFloat_FromDouble(pow(__pyx_v_self->dx, 2.0)); if (unlikely(!__pyx_t_6)) {__pyx_filename = __pyx_f[0]; __pyx_lineno = 418; __pyx_clineno = __LINE__; goto __pyx_L1_error;}
      __Pyx_GOTREF(__pyx_t_6);
      __pyx_t_2 = __Pyx_PyNumber_Divide(__pyx_t_1, __pyx_t_6); if (unlikely(!__pyx_t_2)) {__pyx_filename = __pyx_f[0]; __pyx_lineno = 418; __pyx_clineno = __LINE__; goto __pyx_L1_error;}
      __Pyx_GOTREF(__pyx_t_2);
      __Pyx_DECREF(__pyx_t_1); __pyx_t_1 = 0;
      __Pyx_DECREF(__pyx_t_6); __pyx_t_6 = 0;
      __pyx_t_4 = __pyx_PyFloat_AsDouble(__pyx_t_2); if (unlikely((__pyx_t_4 == (double)-1) && PyErr_Occurred())) {__pyx_filename = __pyx_f[0]; __pyx_lineno = 418; __pyx_clineno = __LINE__; goto __pyx_L1_error;}
      __Pyx_DECREF(__pyx_t_2); __pyx_t_2 = 0;
      __pyx_t_13 = __pyx_v_j;
      __pyx_t_14 = __pyx_v_k;
      *((double *) ( /* dim=1 */ ((char *) (((double *) ( /* dim=0 */ (__pyx_v_dudx2.data + __pyx_t_13 * __pyx_v_dudx2.strides[0]) )) + __pyx_t_14)) )) = __pyx_t_4;
```

```
 419:
```

```
 420:                 dudy2[j,k] = (self.V[j,k+1]-2.*self.V[j,k]+self.V[j,k-1])/(self.dy**2)
```

```
      __pyx_t_2 = __Pyx_PyObject_GetAttrStr(((PyObject *)__pyx_v_self), __pyx_n_s_V); if (unlikely(!__pyx_t_2)) {__pyx_filename = __pyx_f[0]; __pyx_lineno = 420; __pyx_clineno = __LINE__; goto __pyx_L1_error;}
      __Pyx_GOTREF(__pyx_t_2);
      __pyx_t_6 = __Pyx_PyInt_From_int(__pyx_v_j); if (unlikely(!__pyx_t_6)) {__pyx_filename = __pyx_f[0]; __pyx_lineno = 420; __pyx_clineno = __LINE__; goto __pyx_L1_error;}
      __Pyx_GOTREF(__pyx_t_6);
      __pyx_t_1 = __Pyx_PyInt_From_long((__pyx_v_k + 1)); if (unlikely(!__pyx_t_1)) {__pyx_filename = __pyx_f[0]; __pyx_lineno = 420; __pyx_clineno = __LINE__; goto __pyx_L1_error;}
      __Pyx_GOTREF(__pyx_t_1);
      __pyx_t_12 = PyTuple_New(2); if (unlikely(!__pyx_t_12)) {__pyx_filename = __pyx_f[0]; __pyx_lineno = 420; __pyx_clineno = __LINE__; goto __pyx_L1_error;}
      __Pyx_GOTREF(__pyx_t_12);
      PyTuple_SET_ITEM(__pyx_t_12, 0, __pyx_t_6);
      __Pyx_GIVEREF(__pyx_t_6);
      PyTuple_SET_ITEM(__pyx_t_12, 1, __pyx_t_1);
      __Pyx_GIVEREF(__pyx_t_1);
      __pyx_t_6 = 0;
      __pyx_t_1 = 0;
      __pyx_t_1 = PyObject_GetItem(__pyx_t_2, __pyx_t_12); if (unlikely(__pyx_t_1 == NULL)) {__pyx_filename = __pyx_f[0]; __pyx_lineno = 420; __pyx_clineno = __LINE__; goto __pyx_L1_error;};
      __Pyx_GOTREF(__pyx_t_1);
      __Pyx_DECREF(__pyx_t_2); __pyx_t_2 = 0;
      __Pyx_DECREF(__pyx_t_12); __pyx_t_12 = 0;
      __pyx_t_12 = __Pyx_PyObject_GetAttrStr(((PyObject *)__pyx_v_self), __pyx_n_s_V); if (unlikely(!__pyx_t_12)) {__pyx_filename = __pyx_f[0]; __pyx_lineno = 420; __pyx_clineno = __LINE__; goto __pyx_L1_error;}
      __Pyx_GOTREF(__pyx_t_12);
      __pyx_t_2 = __Pyx_PyInt_From_int(__pyx_v_j); if (unlikely(!__pyx_t_2)) {__pyx_filename = __pyx_f[0]; __pyx_lineno = 420; __pyx_clineno = __LINE__; goto __pyx_L1_error;}
      __Pyx_GOTREF(__pyx_t_2);
      __pyx_t_6 = __Pyx_PyInt_From_int(__pyx_v_k); if (unlikely(!__pyx_t_6)) {__pyx_filename = __pyx_f[0]; __pyx_lineno = 420; __pyx_clineno = __LINE__; goto __pyx_L1_error;}
      __Pyx_GOTREF(__pyx_t_6);
      __pyx_t_5 = PyTuple_New(2); if (unlikely(!__pyx_t_5)) {__pyx_filename = __pyx_f[0]; __pyx_lineno = 420; __pyx_clineno = __LINE__; goto __pyx_L1_error;}
      __Pyx_GOTREF(__pyx_t_5);
      PyTuple_SET_ITEM(__pyx_t_5, 0, __pyx_t_2);
      __Pyx_GIVEREF(__pyx_t_2);
      PyTuple_SET_ITEM(__pyx_t_5, 1, __pyx_t_6);
      __Pyx_GIVEREF(__pyx_t_6);
      __pyx_t_2 = 0;
      __pyx_t_6 = 0;
      __pyx_t_6 = PyObject_GetItem(__pyx_t_12, __pyx_t_5); if (unlikely(__pyx_t_6 == NULL)) {__pyx_filename = __pyx_f[0]; __pyx_lineno = 420; __pyx_clineno = __LINE__; goto __pyx_L1_error;};
      __Pyx_GOTREF(__pyx_t_6);
      __Pyx_DECREF(__pyx_t_12); __pyx_t_12 = 0;
      __Pyx_DECREF(__pyx_t_5); __pyx_t_5 = 0;
      __pyx_t_5 = PyNumber_Multiply(__pyx_float_2_, __pyx_t_6); if (unlikely(!__pyx_t_5)) {__pyx_filename = __pyx_f[0]; __pyx_lineno = 420; __pyx_clineno = __LINE__; goto __pyx_L1_error;}
      __Pyx_GOTREF(__pyx_t_5);
      __Pyx_DECREF(__pyx_t_6); __pyx_t_6 = 0;
      __pyx_t_6 = PyNumber_Subtract(__pyx_t_1, __pyx_t_5); if (unlikely(!__pyx_t_6)) {__pyx_filename = __pyx_f[0]; __pyx_lineno = 420; __pyx_clineno = __LINE__; goto __pyx_L1_error;}
      __Pyx_GOTREF(__pyx_t_6);
      __Pyx_DECREF(__pyx_t_1); __pyx_t_1 = 0;
      __Pyx_DECREF(__pyx_t_5); __pyx_t_5 = 0;
      __pyx_t_5 = __Pyx_PyObject_GetAttrStr(((PyObject *)__pyx_v_self), __pyx_n_s_V); if (unlikely(!__pyx_t_5)) {__pyx_filename = __pyx_f[0]; __pyx_lineno = 420; __pyx_clineno = __LINE__; goto __pyx_L1_error;}
      __Pyx_GOTREF(__pyx_t_5);
      __pyx_t_1 = __Pyx_PyInt_From_int(__pyx_v_j); if (unlikely(!__pyx_t_1)) {__pyx_filename = __pyx_f[0]; __pyx_lineno = 420; __pyx_clineno = __LINE__; goto __pyx_L1_error;}
      __Pyx_GOTREF(__pyx_t_1);
      __pyx_t_12 = __Pyx_PyInt_From_long((__pyx_v_k - 1)); if (unlikely(!__pyx_t_12)) {__pyx_filename = __pyx_f[0]; __pyx_lineno = 420; __pyx_clineno = __LINE__; goto __pyx_L1_error;}
      __Pyx_GOTREF(__pyx_t_12);
      __pyx_t_2 = PyTuple_New(2); if (unlikely(!__pyx_t_2)) {__pyx_filename = __pyx_f[0]; __pyx_lineno = 420; __pyx_clineno = __LINE__; goto __pyx_L1_error;}
      __Pyx_GOTREF(__pyx_t_2);
      PyTuple_SET_ITEM(__pyx_t_2, 0, __pyx_t_1);
      __Pyx_GIVEREF(__pyx_t_1);
      PyTuple_SET_ITEM(__pyx_t_2, 1, __pyx_t_12);
      __Pyx_GIVEREF(__pyx_t_12);
      __pyx_t_1 = 0;
      __pyx_t_12 = 0;
      __pyx_t_12 = PyObject_GetItem(__pyx_t_5, __pyx_t_2); if (unlikely(__pyx_t_12 == NULL)) {__pyx_filename = __pyx_f[0]; __pyx_lineno = 420; __pyx_clineno = __LINE__; goto __pyx_L1_error;};
      __Pyx_GOTREF(__pyx_t_12);
      __Pyx_DECREF(__pyx_t_5); __pyx_t_5 = 0;
      __Pyx_DECREF(__pyx_t_2); __pyx_t_2 = 0;
      __pyx_t_2 = PyNumber_Add(__pyx_t_6, __pyx_t_12); if (unlikely(!__pyx_t_2)) {__pyx_filename = __pyx_f[0]; __pyx_lineno = 420; __pyx_clineno = __LINE__; goto __pyx_L1_error;}
      __Pyx_GOTREF(__pyx_t_2);
      __Pyx_DECREF(__pyx_t_6); __pyx_t_6 = 0;
      __Pyx_DECREF(__pyx_t_12); __pyx_t_12 = 0;
      __pyx_t_12 = PyFloat_FromDouble(pow(__pyx_v_self->dy, 2.0)); if (unlikely(!__pyx_t_12)) {__pyx_filename = __pyx_f[0]; __pyx_lineno = 420; __pyx_clineno = __LINE__; goto __pyx_L1_error;}
      __Pyx_GOTREF(__pyx_t_12);
      __pyx_t_6 = __Pyx_PyNumber_Divide(__pyx_t_2, __pyx_t_12); if (unlikely(!__pyx_t_6)) {__pyx_filename = __pyx_f[0]; __pyx_lineno = 420; __pyx_clineno = __LINE__; goto __pyx_L1_error;}
      __Pyx_GOTREF(__pyx_t_6);
      __Pyx_DECREF(__pyx_t_2); __pyx_t_2 = 0;
      __Pyx_DECREF(__pyx_t_12); __pyx_t_12 = 0;
      __pyx_t_4 = __pyx_PyFloat_AsDouble(__pyx_t_6); if (unlikely((__pyx_t_4 == (double)-1) && PyErr_Occurred())) {__pyx_filename = __pyx_f[0]; __pyx_lineno = 420; __pyx_clineno = __LINE__; goto __pyx_L1_error;}
      __Pyx_DECREF(__pyx_t_6); __pyx_t_6 = 0;
      __pyx_t_15 = __pyx_v_j;
      __pyx_t_16 = __pyx_v_k;
      *((double *) ( /* dim=1 */ ((char *) (((double *) ( /* dim=0 */ (__pyx_v_dudy2.data + __pyx_t_15 * __pyx_v_dudy2.strides[0]) )) + __pyx_t_16)) )) = __pyx_t_4;
    }
  }
```

```
 421:
```

```
 422:
```

```
 423:         for x in range (nx):
```

```
  __pyx_t_3 = __pyx_v_nx;
  for (__pyx_t_11 = 0; __pyx_t_11 < __pyx_t_3; __pyx_t_11+=1) {
    __pyx_v_x = __pyx_t_11;
```

```
 424:             for y in range (ny):
```

```
    __pyx_t_17 = __pyx_v_ny;
    for (__pyx_t_18 = 0; __pyx_t_18 < __pyx_t_17; __pyx_t_18+=1) {
      __pyx_v_y = __pyx_t_18;
```

```
 425:                 #print(self.V[x,y])
```

```
 426:                 V = self.V[x, y]
```

```
      __pyx_t_6 = __Pyx_PyObject_GetAttrStr(((PyObject *)__pyx_v_self), __pyx_n_s_V); if (unlikely(!__pyx_t_6)) {__pyx_filename = __pyx_f[0]; __pyx_lineno = 426; __pyx_clineno = __LINE__; goto __pyx_L1_error;}
      __Pyx_GOTREF(__pyx_t_6);
      __pyx_t_12 = __Pyx_PyInt_From_int(__pyx_v_x); if (unlikely(!__pyx_t_12)) {__pyx_filename = __pyx_f[0]; __pyx_lineno = 426; __pyx_clineno = __LINE__; goto __pyx_L1_error;}
      __Pyx_GOTREF(__pyx_t_12);
      __pyx_t_2 = __Pyx_PyInt_From_int(__pyx_v_y); if (unlikely(!__pyx_t_2)) {__pyx_filename = __pyx_f[0]; __pyx_lineno = 426; __pyx_clineno = __LINE__; goto __pyx_L1_error;}
      __Pyx_GOTREF(__pyx_t_2);
      __pyx_t_5 = PyTuple_New(2); if (unlikely(!__pyx_t_5)) {__pyx_filename = __pyx_f[0]; __pyx_lineno = 426; __pyx_clineno = __LINE__; goto __pyx_L1_error;}
      __Pyx_GOTREF(__pyx_t_5);
      PyTuple_SET_ITEM(__pyx_t_5, 0, __pyx_t_12);
      __Pyx_GIVEREF(__pyx_t_12);
      PyTuple_SET_ITEM(__pyx_t_5, 1, __pyx_t_2);
      __Pyx_GIVEREF(__pyx_t_2);
      __pyx_t_12 = 0;
      __pyx_t_2 = 0;
      __pyx_t_2 = PyObject_GetItem(__pyx_t_6, __pyx_t_5); if (unlikely(__pyx_t_2 == NULL)) {__pyx_filename = __pyx_f[0]; __pyx_lineno = 426; __pyx_clineno = __LINE__; goto __pyx_L1_error;};
      __Pyx_GOTREF(__pyx_t_2);
      __Pyx_DECREF(__pyx_t_6); __pyx_t_6 = 0;
      __Pyx_DECREF(__pyx_t_5); __pyx_t_5 = 0;
      __pyx_t_4 = __pyx_PyFloat_AsDouble(__pyx_t_2); if (unlikely((__pyx_t_4 == (double)-1) && PyErr_Occurred())) {__pyx_filename = __pyx_f[0]; __pyx_lineno = 426; __pyx_clineno = __LINE__; goto __pyx_L1_error;}
      __Pyx_DECREF(__pyx_t_2); __pyx_t_2 = 0;
      __pyx_v_V = __pyx_t_4;
```

```
 427:                 I_K = self.I_K[x, y]
```

```
      if (unlikely(!__pyx_v_self->I_K.memview)) {PyErr_SetString(PyExc_AttributeError,"Memoryview is not initialized");{__pyx_filename = __pyx_f[0]; __pyx_lineno = 427; __pyx_clineno = __LINE__; goto __pyx_L1_error;}}
      __pyx_t_19 = __pyx_v_x;
      __pyx_t_20 = __pyx_v_y;
      __pyx_v_I_K = (*((double *) ( /* dim=1 */ ((char *) (((double *) ( /* dim=0 */ (__pyx_v_self->I_K.data + __pyx_t_19 * __pyx_v_self->I_K.strides[0]) )) + __pyx_t_20)) )));
```

```
 428:                 Ca2_i = self.Ca2_i[x, y]
```

```
      __pyx_t_2 = __Pyx_PyObject_GetAttrStr(((PyObject *)__pyx_v_self), __pyx_n_s_Ca2_i); if (unlikely(!__pyx_t_2)) {__pyx_filename = __pyx_f[0]; __pyx_lineno = 428; __pyx_clineno = __LINE__; goto __pyx_L1_error;}
      __Pyx_GOTREF(__pyx_t_2);
      __pyx_t_5 = __Pyx_PyInt_From_int(__pyx_v_x); if (unlikely(!__pyx_t_5)) {__pyx_filename = __pyx_f[0]; __pyx_lineno = 428; __pyx_clineno = __LINE__; goto __pyx_L1_error;}
      __Pyx_GOTREF(__pyx_t_5);
      __pyx_t_6 = __Pyx_PyInt_From_int(__pyx_v_y); if (unlikely(!__pyx_t_6)) {__pyx_filename = __pyx_f[0]; __pyx_lineno = 428; __pyx_clineno = __LINE__; goto __pyx_L1_error;}
      __Pyx_GOTREF(__pyx_t_6);
      __pyx_t_12 = PyTuple_New(2); if (unlikely(!__pyx_t_12)) {__pyx_filename = __pyx_f[0]; __pyx_lineno = 428; __pyx_clineno = __LINE__; goto __pyx_L1_error;}
      __Pyx_GOTREF(__pyx_t_12);
      PyTuple_SET_ITEM(__pyx_t_12, 0, __pyx_t_5);
      __Pyx_GIVEREF(__pyx_t_5);
      PyTuple_SET_ITEM(__pyx_t_12, 1, __pyx_t_6);
      __Pyx_GIVEREF(__pyx_t_6);
      __pyx_t_5 = 0;
      __pyx_t_6 = 0;
      __pyx_t_6 = PyObject_GetItem(__pyx_t_2, __pyx_t_12); if (unlikely(__pyx_t_6 == NULL)) {__pyx_filename = __pyx_f[0]; __pyx_lineno = 428; __pyx_clineno = __LINE__; goto __pyx_L1_error;};
      __Pyx_GOTREF(__pyx_t_6);
      __Pyx_DECREF(__pyx_t_2); __pyx_t_2 = 0;
      __Pyx_DECREF(__pyx_t_12); __pyx_t_12 = 0;
      __pyx_t_4 = __pyx_PyFloat_AsDouble(__pyx_t_6); if (unlikely((__pyx_t_4 == (double)-1) && PyErr_Occurred())) {__pyx_filename = __pyx_f[0]; __pyx_lineno = 428; __pyx_clineno = __LINE__; goto __pyx_L1_error;}
      __Pyx_DECREF(__pyx_t_6); __pyx_t_6 = 0;
      __pyx_v_Ca2_i = __pyx_t_4;
```

```
 429:                 Ca2_ss = self.Ca2_ss[x, y]
```

```
      if (unlikely(!__pyx_v_self->Ca2_ss.memview)) {PyErr_SetString(PyExc_AttributeError,"Memoryview is not initialized");{__pyx_filename = __pyx_f[0]; __pyx_lineno = 429; __pyx_clineno = __LINE__; goto __pyx_L1_error;}}
      __pyx_t_21 = __pyx_v_x;
      __pyx_t_22 = __pyx_v_y;
      __pyx_v_Ca2_ss = (*((double *) ( /* dim=1 */ ((char *) (((double *) ( /* dim=0 */ (__pyx_v_self->Ca2_ss.data + __pyx_t_21 * __pyx_v_self->Ca2_ss.strides[0]) )) + __pyx_t_22)) )));
```

```
 430:                 Ca2_JSR = self.Ca2_JSR[x, y]
```

```
      if (unlikely(!__pyx_v_self->Ca2_JSR.memview)) {PyErr_SetString(PyExc_AttributeError,"Memoryview is not initialized");{__pyx_filename = __pyx_f[0]; __pyx_lineno = 430; __pyx_clineno = __LINE__; goto __pyx_L1_error;}}
      __pyx_t_23 = __pyx_v_x;
      __pyx_t_24 = __pyx_v_y;
      __pyx_v_Ca2_JSR = (*((double *) ( /* dim=1 */ ((char *) (((double *) ( /* dim=0 */ (__pyx_v_self->Ca2_JSR.data + __pyx_t_23 * __pyx_v_self->Ca2_JSR.strides[0]) )) + __pyx_t_24)) )));
```

```
 431:                 Ca2_NSR = self.Ca2_NSR[x, y]
```

```
      if (unlikely(!__pyx_v_self->Ca2_NSR.memview)) {PyErr_SetString(PyExc_AttributeError,"Memoryview is not initialized");{__pyx_filename = __pyx_f[0]; __pyx_lineno = 431; __pyx_clineno = __LINE__; goto __pyx_L1_error;}}
      __pyx_t_25 = __pyx_v_x;
      __pyx_t_26 = __pyx_v_y;
      __pyx_v_Ca2_NSR = (*((double *) ( /* dim=1 */ ((char *) (((double *) ( /* dim=0 */ (__pyx_v_self->Ca2_NSR.data + __pyx_t_25 * __pyx_v_self->Ca2_NSR.strides[0]) )) + __pyx_t_26)) )));
```

```
 432:                 LTRPNCa = self.LTRPNCa[x, y]
```

```
      if (unlikely(!__pyx_v_self->LTRPNCa.memview)) {PyErr_SetString(PyExc_AttributeError,"Memoryview is not initialized");{__pyx_filename = __pyx_f[0]; __pyx_lineno = 432; __pyx_clineno = __LINE__; goto __pyx_L1_error;}}
      __pyx_t_27 = __pyx_v_x;
      __pyx_t_28 = __pyx_v_y;
      __pyx_v_LTRPNCa = (*((double *) ( /* dim=1 */ ((char *) (((double *) ( /* dim=0 */ (__pyx_v_self->LTRPNCa.data + __pyx_t_27 * __pyx_v_self->LTRPNCa.strides[0]) )) + __pyx_t_28)) )));
```

```
 433:                 HTRPNCa = self.HTRPNCa[x, y]
```

```
      if (unlikely(!__pyx_v_self->HTRPNCa.memview)) {PyErr_SetString(PyExc_AttributeError,"Memoryview is not initialized");{__pyx_filename = __pyx_f[0]; __pyx_lineno = 433; __pyx_clineno = __LINE__; goto __pyx_L1_error;}}
      __pyx_t_29 = __pyx_v_x;
      __pyx_t_30 = __pyx_v_y;
      __pyx_v_HTRPNCa = (*((double *) ( /* dim=1 */ ((char *) (((double *) ( /* dim=0 */ (__pyx_v_self->HTRPNCa.data + __pyx_t_29 * __pyx_v_self->HTRPNCa.strides[0]) )) + __pyx_t_30)) )));
```

```
 434:                 O = self.O[x, y]
```

```
      if (unlikely(!__pyx_v_self->O.memview)) {PyErr_SetString(PyExc_AttributeError,"Memoryview is not initialized");{__pyx_filename = __pyx_f[0]; __pyx_lineno = 434; __pyx_clineno = __LINE__; goto __pyx_L1_error;}}
      __pyx_t_31 = __pyx_v_x;
      __pyx_t_32 = __pyx_v_y;
      __pyx_v_O = (*((double *) ( /* dim=1 */ ((char *) (((double *) ( /* dim=0 */ (__pyx_v_self->O.data + __pyx_t_31 * __pyx_v_self->O.strides[0]) )) + __pyx_t_32)) )));
```

```
 435:                 C2 = self.C2[x, y]
```

```
      if (unlikely(!__pyx_v_self->C2.memview)) {PyErr_SetString(PyExc_AttributeError,"Memoryview is not initialized");{__pyx_filename = __pyx_f[0]; __pyx_lineno = 435; __pyx_clineno = __LINE__; goto __pyx_L1_error;}}
      __pyx_t_33 = __pyx_v_x;
      __pyx_t_34 = __pyx_v_y;
      __pyx_v_C2 = (*((double *) ( /* dim=1 */ ((char *) (((double *) ( /* dim=0 */ (__pyx_v_self->C2.data + __pyx_t_33 * __pyx_v_self->C2.strides[0]) )) + __pyx_t_34)) )));
```

```
 436:                 C3 = self.C3[x, y]
```

```
      if (unlikely(!__pyx_v_self->C3.memview)) {PyErr_SetString(PyExc_AttributeError,"Memoryview is not initialized");{__pyx_filename = __pyx_f[0]; __pyx_lineno = 436; __pyx_clineno = __LINE__; goto __pyx_L1_error;}}
      __pyx_t_35 = __pyx_v_x;
      __pyx_t_36 = __pyx_v_y;
      __pyx_v_C3 = (*((double *) ( /* dim=1 */ ((char *) (((double *) ( /* dim=0 */ (__pyx_v_self->C3.data + __pyx_t_35 * __pyx_v_self->C3.strides[0]) )) + __pyx_t_36)) )));
```

```
 437:                 C4 = self.C4[x, y]
```

```
      if (unlikely(!__pyx_v_self->C4.memview)) {PyErr_SetString(PyExc_AttributeError,"Memoryview is not initialized");{__pyx_filename = __pyx_f[0]; __pyx_lineno = 437; __pyx_clineno = __LINE__; goto __pyx_L1_error;}}
      __pyx_t_37 = __pyx_v_x;
      __pyx_t_38 = __pyx_v_y;
      __pyx_v_C4 = (*((double *) ( /* dim=1 */ ((char *) (((double *) ( /* dim=0 */ (__pyx_v_self->C4.data + __pyx_t_37 * __pyx_v_self->C4.strides[0]) )) + __pyx_t_38)) )));
```

```
 438:                 I1 = self.I1[x, y]
```

```
      if (unlikely(!__pyx_v_self->I1.memview)) {PyErr_SetString(PyExc_AttributeError,"Memoryview is not initialized");{__pyx_filename = __pyx_f[0]; __pyx_lineno = 438; __pyx_clineno = __LINE__; goto __pyx_L1_error;}}
      __pyx_t_39 = __pyx_v_x;
      __pyx_t_40 = __pyx_v_y;
      __pyx_v_I1 = (*((double *) ( /* dim=1 */ ((char *) (((double *) ( /* dim=0 */ (__pyx_v_self->I1.data + __pyx_t_39 * __pyx_v_self->I1.strides[0]) )) + __pyx_t_40)) )));
```

```
 439:                 I2 = self.I2[x, y]
```

```
      if (unlikely(!__pyx_v_self->I2.memview)) {PyErr_SetString(PyExc_AttributeError,"Memoryview is not initialized");{__pyx_filename = __pyx_f[0]; __pyx_lineno = 439; __pyx_clineno = __LINE__; goto __pyx_L1_error;}}
      __pyx_t_41 = __pyx_v_x;
      __pyx_t_42 = __pyx_v_y;
      __pyx_v_I2 = (*((double *) ( /* dim=1 */ ((char *) (((double *) ( /* dim=0 */ (__pyx_v_self->I2.data + __pyx_t_41 * __pyx_v_self->I2.strides[0]) )) + __pyx_t_42)) )));
```

```
 440:                 I3 = self.I3[x, y]
```

```
      if (unlikely(!__pyx_v_self->I3.memview)) {PyErr_SetString(PyExc_AttributeError,"Memoryview is not initialized");{__pyx_filename = __pyx_f[0]; __pyx_lineno = 440; __pyx_clineno = __LINE__; goto __pyx_L1_error;}}
      __pyx_t_43 = __pyx_v_x;
      __pyx_t_44 = __pyx_v_y;
      __pyx_v_I3 = (*((double *) ( /* dim=1 */ ((char *) (((double *) ( /* dim=0 */ (__pyx_v_self->I3.data + __pyx_t_43 * __pyx_v_self->I3.strides[0]) )) + __pyx_t_44)) )));
```

```
 441:                 Pc2 = self.Pc2[x, y]
```

```
      if (unlikely(!__pyx_v_self->Pc2.memview)) {PyErr_SetString(PyExc_AttributeError,"Memoryview is not initialized");{__pyx_filename = __pyx_f[0]; __pyx_lineno = 441; __pyx_clineno = __LINE__; goto __pyx_L1_error;}}
      __pyx_t_45 = __pyx_v_x;
      __pyx_t_46 = __pyx_v_y;
      __pyx_v_Pc2 = (*((double *) ( /* dim=1 */ ((char *) (((double *) ( /* dim=0 */ (__pyx_v_self->Pc2.data + __pyx_t_45 * __pyx_v_self->Pc2.strides[0]) )) + __pyx_t_46)) )));
```

```
 442:                 Po1 = self.Po1[x, y]
```

```
      if (unlikely(!__pyx_v_self->Po1.memview)) {PyErr_SetString(PyExc_AttributeError,"Memoryview is not initialized");{__pyx_filename = __pyx_f[0]; __pyx_lineno = 442; __pyx_clineno = __LINE__; goto __pyx_L1_error;}}
      __pyx_t_47 = __pyx_v_x;
      __pyx_t_48 = __pyx_v_y;
      __pyx_v_Po1 = (*((double *) ( /* dim=1 */ ((char *) (((double *) ( /* dim=0 */ (__pyx_v_self->Po1.data + __pyx_t_47 * __pyx_v_self->Po1.strides[0]) )) + __pyx_t_48)) )));
```

```
 443:                 Po2 = self.Po2[x, y]
```

```
      if (unlikely(!__pyx_v_self->Po2.memview)) {PyErr_SetString(PyExc_AttributeError,"Memoryview is not initialized");{__pyx_filename = __pyx_f[0]; __pyx_lineno = 443; __pyx_clineno = __LINE__; goto __pyx_L1_error;}}
      __pyx_t_49 = __pyx_v_x;
      __pyx_t_50 = __pyx_v_y;
      __pyx_v_Po2 = (*((double *) ( /* dim=1 */ ((char *) (((double *) ( /* dim=0 */ (__pyx_v_self->Po2.data + __pyx_t_49 * __pyx_v_self->Po2.strides[0]) )) + __pyx_t_50)) )));
```

```
 444:                 P_RyR = self.P_RyR[x, y]
```

```
      if (unlikely(!__pyx_v_self->P_RyR.memview)) {PyErr_SetString(PyExc_AttributeError,"Memoryview is not initialized");{__pyx_filename = __pyx_f[0]; __pyx_lineno = 444; __pyx_clineno = __LINE__; goto __pyx_L1_error;}}
      __pyx_t_51 = __pyx_v_x;
      __pyx_t_52 = __pyx_v_y;
      __pyx_v_P_RyR = (*((double *) ( /* dim=1 */ ((char *) (((double *) ( /* dim=0 */ (__pyx_v_self->P_RyR.data + __pyx_t_51 * __pyx_v_self->P_RyR.strides[0]) )) + __pyx_t_52)) )));
```

```
 445:                 C_Na2 = self.C_Na2[x, y]
```

```
      if (unlikely(!__pyx_v_self->C_Na2.memview)) {PyErr_SetString(PyExc_AttributeError,"Memoryview is not initialized");{__pyx_filename = __pyx_f[0]; __pyx_lineno = 445; __pyx_clineno = __LINE__; goto __pyx_L1_error;}}
      __pyx_t_53 = __pyx_v_x;
      __pyx_t_54 = __pyx_v_y;
      __pyx_v_C_Na2 = (*((double *) ( /* dim=1 */ ((char *) (((double *) ( /* dim=0 */ (__pyx_v_self->C_Na2.data + __pyx_t_53 * __pyx_v_self->C_Na2.strides[0]) )) + __pyx_t_54)) )));
```

```
 446:                 C_Na1 = self.C_Na1[x, y]
```

```
      if (unlikely(!__pyx_v_self->C_Na1.memview)) {PyErr_SetString(PyExc_AttributeError,"Memoryview is not initialized");{__pyx_filename = __pyx_f[0]; __pyx_lineno = 446; __pyx_clineno = __LINE__; goto __pyx_L1_error;}}
      __pyx_t_55 = __pyx_v_x;
      __pyx_t_56 = __pyx_v_y;
      __pyx_v_C_Na1 = (*((double *) ( /* dim=1 */ ((char *) (((double *) ( /* dim=0 */ (__pyx_v_self->C_Na1.data + __pyx_t_55 * __pyx_v_self->C_Na1.strides[0]) )) + __pyx_t_56)) )));
```

```
 447:                 O_Na = self.O_Na[x, y]
```

```
      if (unlikely(!__pyx_v_self->O_Na.memview)) {PyErr_SetString(PyExc_AttributeError,"Memoryview is not initialized");{__pyx_filename = __pyx_f[0]; __pyx_lineno = 447; __pyx_clineno = __LINE__; goto __pyx_L1_error;}}
      __pyx_t_57 = __pyx_v_x;
      __pyx_t_58 = __pyx_v_y;
      __pyx_v_O_Na = (*((double *) ( /* dim=1 */ ((char *) (((double *) ( /* dim=0 */ (__pyx_v_self->O_Na.data + __pyx_t_57 * __pyx_v_self->O_Na.strides[0]) )) + __pyx_t_58)) )));
```

```
 448:                 IF_Na = self.IF_Na[x, y]
```

```
      if (unlikely(!__pyx_v_self->IF_Na.memview)) {PyErr_SetString(PyExc_AttributeError,"Memoryview is not initialized");{__pyx_filename = __pyx_f[0]; __pyx_lineno = 448; __pyx_clineno = __LINE__; goto __pyx_L1_error;}}
      __pyx_t_59 = __pyx_v_x;
      __pyx_t_60 = __pyx_v_y;
      __pyx_v_IF_Na = (*((double *) ( /* dim=1 */ ((char *) (((double *) ( /* dim=0 */ (__pyx_v_self->IF_Na.data + __pyx_t_59 * __pyx_v_self->IF_Na.strides[0]) )) + __pyx_t_60)) )));
```

```
 449:                 I1_Na = self.I1_Na[x, y]
```

```
      if (unlikely(!__pyx_v_self->I1_Na.memview)) {PyErr_SetString(PyExc_AttributeError,"Memoryview is not initialized");{__pyx_filename = __pyx_f[0]; __pyx_lineno = 449; __pyx_clineno = __LINE__; goto __pyx_L1_error;}}
      __pyx_t_61 = __pyx_v_x;
      __pyx_t_62 = __pyx_v_y;
      __pyx_v_I1_Na = (*((double *) ( /* dim=1 */ ((char *) (((double *) ( /* dim=0 */ (__pyx_v_self->I1_Na.data + __pyx_t_61 * __pyx_v_self->I1_Na.strides[0]) )) + __pyx_t_62)) )));
```

```
 450:                 I2_Na = self.I2_Na[x, y]
```

```
      if (unlikely(!__pyx_v_self->I2_Na.memview)) {PyErr_SetString(PyExc_AttributeError,"Memoryview is not initialized");{__pyx_filename = __pyx_f[0]; __pyx_lineno = 450; __pyx_clineno = __LINE__; goto __pyx_L1_error;}}
      __pyx_t_63 = __pyx_v_x;
      __pyx_t_64 = __pyx_v_y;
      __pyx_v_I2_Na = (*((double *) ( /* dim=1 */ ((char *) (((double *) ( /* dim=0 */ (__pyx_v_self->I2_Na.data + __pyx_t_63 * __pyx_v_self->I2_Na.strides[0]) )) + __pyx_t_64)) )));
```

```
 451:                 IC_Na2 = self.IC_Na2[x, y]
```

```
      if (unlikely(!__pyx_v_self->IC_Na2.memview)) {PyErr_SetString(PyExc_AttributeError,"Memoryview is not initialized");{__pyx_filename = __pyx_f[0]; __pyx_lineno = 451; __pyx_clineno = __LINE__; goto __pyx_L1_error;}}
      __pyx_t_65 = __pyx_v_x;
      __pyx_t_66 = __pyx_v_y;
      __pyx_v_IC_Na2 = (*((double *) ( /* dim=1 */ ((char *) (((double *) ( /* dim=0 */ (__pyx_v_self->IC_Na2.data + __pyx_t_65 * __pyx_v_self->IC_Na2.strides[0]) )) + __pyx_t_66)) )));
```

```
 452:                 IC_Na3 = self.IC_Na3[x, y]
```

```
      if (unlikely(!__pyx_v_self->IC_Na3.memview)) {PyErr_SetString(PyExc_AttributeError,"Memoryview is not initialized");{__pyx_filename = __pyx_f[0]; __pyx_lineno = 452; __pyx_clineno = __LINE__; goto __pyx_L1_error;}}
      __pyx_t_67 = __pyx_v_x;
      __pyx_t_68 = __pyx_v_y;
      __pyx_v_IC_Na3 = (*((double *) ( /* dim=1 */ ((char *) (((double *) ( /* dim=0 */ (__pyx_v_self->IC_Na3.data + __pyx_t_67 * __pyx_v_self->IC_Na3.strides[0]) )) + __pyx_t_68)) )));
```

```
 453:                 Na_i = self.Na_i[x, y]
```

```
      if (unlikely(!__pyx_v_self->Na_i.memview)) {PyErr_SetString(PyExc_AttributeError,"Memoryview is not initialized");{__pyx_filename = __pyx_f[0]; __pyx_lineno = 453; __pyx_clineno = __LINE__; goto __pyx_L1_error;}}
      __pyx_t_69 = __pyx_v_x;
      __pyx_t_70 = __pyx_v_y;
      __pyx_v_Na_i = (*((double *) ( /* dim=1 */ ((char *) (((double *) ( /* dim=0 */ (__pyx_v_self->Na_i.data + __pyx_t_69 * __pyx_v_self->Na_i.strides[0]) )) + __pyx_t_70)) )));
```

```
 454:                 K_i = self.K_i[x, y]
```

```
      if (unlikely(!__pyx_v_self->K_i.memview)) {PyErr_SetString(PyExc_AttributeError,"Memoryview is not initialized");{__pyx_filename = __pyx_f[0]; __pyx_lineno = 454; __pyx_clineno = __LINE__; goto __pyx_L1_error;}}
      __pyx_t_71 = __pyx_v_x;
      __pyx_t_72 = __pyx_v_y;
      __pyx_v_K_i = (*((double *) ( /* dim=1 */ ((char *) (((double *) ( /* dim=0 */ (__pyx_v_self->K_i.data + __pyx_t_71 * __pyx_v_self->K_i.strides[0]) )) + __pyx_t_72)) )));
```

```
 455:                 a_tof = self.a_tof[x, y]
```

```
      if (unlikely(!__pyx_v_self->a_tof.memview)) {PyErr_SetString(PyExc_AttributeError,"Memoryview is not initialized");{__pyx_filename = __pyx_f[0]; __pyx_lineno = 455; __pyx_clineno = __LINE__; goto __pyx_L1_error;}}
      __pyx_t_73 = __pyx_v_x;
      __pyx_t_74 = __pyx_v_y;
      __pyx_v_a_tof = (*((double *) ( /* dim=1 */ ((char *) (((double *) ( /* dim=0 */ (__pyx_v_self->a_tof.data + __pyx_t_73 * __pyx_v_self->a_tof.strides[0]) )) + __pyx_t_74)) )));
```

```
 456:                 i_tof = self.i_tof[x, y]
```

```
      if (unlikely(!__pyx_v_self->i_tof.memview)) {PyErr_SetString(PyExc_AttributeError,"Memoryview is not initialized");{__pyx_filename = __pyx_f[0]; __pyx_lineno = 456; __pyx_clineno = __LINE__; goto __pyx_L1_error;}}
      __pyx_t_75 = __pyx_v_x;
      __pyx_t_76 = __pyx_v_y;
      __pyx_v_i_tof = (*((double *) ( /* dim=1 */ ((char *) (((double *) ( /* dim=0 */ (__pyx_v_self->i_tof.data + __pyx_t_75 * __pyx_v_self->i_tof.strides[0]) )) + __pyx_t_76)) )));
```

```
 457:                 n_Ks = self.n_Ks[x, y]
```

```
      if (unlikely(!__pyx_v_self->n_Ks.memview)) {PyErr_SetString(PyExc_AttributeError,"Memoryview is not initialized");{__pyx_filename = __pyx_f[0]; __pyx_lineno = 457; __pyx_clineno = __LINE__; goto __pyx_L1_error;}}
      __pyx_t_77 = __pyx_v_x;
      __pyx_t_78 = __pyx_v_y;
      __pyx_v_n_Ks = (*((double *) ( /* dim=1 */ ((char *) (((double *) ( /* dim=0 */ (__pyx_v_self->n_Ks.data + __pyx_t_77 * __pyx_v_self->n_Ks.strides[0]) )) + __pyx_t_78)) )));
```

```
 458:                 a_tos = self.a_tos[x, y]
```

```
      if (unlikely(!__pyx_v_self->a_tos.memview)) {PyErr_SetString(PyExc_AttributeError,"Memoryview is not initialized");{__pyx_filename = __pyx_f[0]; __pyx_lineno = 458; __pyx_clineno = __LINE__; goto __pyx_L1_error;}}
      __pyx_t_79 = __pyx_v_x;
      __pyx_t_80 = __pyx_v_y;
      __pyx_v_a_tos = (*((double *) ( /* dim=1 */ ((char *) (((double *) ( /* dim=0 */ (__pyx_v_self->a_tos.data + __pyx_t_79 * __pyx_v_self->a_tos.strides[0]) )) + __pyx_t_80)) )));
```

```
 459:                 i_tos = self.i_tos[x, y]
```

```
      if (unlikely(!__pyx_v_self->i_tos.memview)) {PyErr_SetString(PyExc_AttributeError,"Memoryview is not initialized");{__pyx_filename = __pyx_f[0]; __pyx_lineno = 459; __pyx_clineno = __LINE__; goto __pyx_L1_error;}}
      __pyx_t_81 = __pyx_v_x;
      __pyx_t_82 = __pyx_v_y;
      __pyx_v_i_tos = (*((double *) ( /* dim=1 */ ((char *) (((double *) ( /* dim=0 */ (__pyx_v_self->i_tos.data + __pyx_t_81 * __pyx_v_self->i_tos.strides[0]) )) + __pyx_t_82)) )));
```

```
 460:                 a_ur = self.a_ur[x, y]
```

```
      if (unlikely(!__pyx_v_self->a_ur.memview)) {PyErr_SetString(PyExc_AttributeError,"Memoryview is not initialized");{__pyx_filename = __pyx_f[0]; __pyx_lineno = 460; __pyx_clineno = __LINE__; goto __pyx_L1_error;}}
      __pyx_t_83 = __pyx_v_x;
      __pyx_t_84 = __pyx_v_y;
      __pyx_v_a_ur = (*((double *) ( /* dim=1 */ ((char *) (((double *) ( /* dim=0 */ (__pyx_v_self->a_ur.data + __pyx_t_83 * __pyx_v_self->a_ur.strides[0]) )) + __pyx_t_84)) )));
```

```
 461:                 i_ur = self.i_ur[x, y]
```

```
      if (unlikely(!__pyx_v_self->i_ur.memview)) {PyErr_SetString(PyExc_AttributeError,"Memoryview is not initialized");{__pyx_filename = __pyx_f[0]; __pyx_lineno = 461; __pyx_clineno = __LINE__; goto __pyx_L1_error;}}
      __pyx_t_85 = __pyx_v_x;
      __pyx_t_86 = __pyx_v_y;
      __pyx_v_i_ur = (*((double *) ( /* dim=1 */ ((char *) (((double *) ( /* dim=0 */ (__pyx_v_self->i_ur.data + __pyx_t_85 * __pyx_v_self->i_ur.strides[0]) )) + __pyx_t_86)) )));
```

```
 462:                 a_Kss = self.a_Kss[x, y]
```

```
      if (unlikely(!__pyx_v_self->a_Kss.memview)) {PyErr_SetString(PyExc_AttributeError,"Memoryview is not initialized");{__pyx_filename = __pyx_f[0]; __pyx_lineno = 462; __pyx_clineno = __LINE__; goto __pyx_L1_error;}}
      __pyx_t_87 = __pyx_v_x;
      __pyx_t_88 = __pyx_v_y;
      __pyx_v_a_Kss = (*((double *) ( /* dim=1 */ ((char *) (((double *) ( /* dim=0 */ (__pyx_v_self->a_Kss.data + __pyx_t_87 * __pyx_v_self->a_Kss.strides[0]) )) + __pyx_t_88)) )));
```

```
 463:                 C_K1 = self.C_K1[x, y]
```

```
      if (unlikely(!__pyx_v_self->C_K1.memview)) {PyErr_SetString(PyExc_AttributeError,"Memoryview is not initialized");{__pyx_filename = __pyx_f[0]; __pyx_lineno = 463; __pyx_clineno = __LINE__; goto __pyx_L1_error;}}
      __pyx_t_89 = __pyx_v_x;
      __pyx_t_90 = __pyx_v_y;
      __pyx_v_C_K1 = (*((double *) ( /* dim=1 */ ((char *) (((double *) ( /* dim=0 */ (__pyx_v_self->C_K1.data + __pyx_t_89 * __pyx_v_self->C_K1.strides[0]) )) + __pyx_t_90)) )));
```

```
 464:                 C_K2 = self.C_K2[x, y]
```

```
      if (unlikely(!__pyx_v_self->C_K2.memview)) {PyErr_SetString(PyExc_AttributeError,"Memoryview is not initialized");{__pyx_filename = __pyx_f[0]; __pyx_lineno = 464; __pyx_clineno = __LINE__; goto __pyx_L1_error;}}
      __pyx_t_91 = __pyx_v_x;
      __pyx_t_92 = __pyx_v_y;
      __pyx_v_C_K2 = (*((double *) ( /* dim=1 */ ((char *) (((double *) ( /* dim=0 */ (__pyx_v_self->C_K2.data + __pyx_t_91 * __pyx_v_self->C_K2.strides[0]) )) + __pyx_t_92)) )));
```

```
 465:                 O_K = self.O_K[x, y]
```

```
      if (unlikely(!__pyx_v_self->O_K.memview)) {PyErr_SetString(PyExc_AttributeError,"Memoryview is not initialized");{__pyx_filename = __pyx_f[0]; __pyx_lineno = 465; __pyx_clineno = __LINE__; goto __pyx_L1_error;}}
      __pyx_t_93 = __pyx_v_x;
      __pyx_t_94 = __pyx_v_y;
      __pyx_v_O_K = (*((double *) ( /* dim=1 */ ((char *) (((double *) ( /* dim=0 */ (__pyx_v_self->O_K.data + __pyx_t_93 * __pyx_v_self->O_K.strides[0]) )) + __pyx_t_94)) )));
```

```
 466:                 I_K = self.I_K[x, y]
```

```
      if (unlikely(!__pyx_v_self->I_K.memview)) {PyErr_SetString(PyExc_AttributeError,"Memoryview is not initialized");{__pyx_filename = __pyx_f[0]; __pyx_lineno = 466; __pyx_clineno = __LINE__; goto __pyx_L1_error;}}
      __pyx_t_95 = __pyx_v_x;
      __pyx_t_96 = __pyx_v_y;
      __pyx_v_I_K = (*((double *) ( /* dim=1 */ ((char *) (((double *) ( /* dim=0 */ (__pyx_v_self->I_K.data + __pyx_t_95 * __pyx_v_self->I_K.strides[0]) )) + __pyx_t_96)) )));
```

```
 467:
```

```
 468:
```

```
 469:                 B_i = 1./(1 + (CMDN_tot*k_CMDN_m)/((k_CMDN_m + Ca2_i)**2))
```

```
      __pyx_v_B_i = (1. / (1.0 + ((__pyx_v_CMDN_tot * __pyx_v_k_CMDN_m) / pow((__pyx_v_k_CMDN_m + __pyx_v_Ca2_i), 2.0))));
```

```
 470:
```

```
 471:
```

```
 472:                 J_leak = v_2*(Ca2_NSR - Ca2_i)
```

```
      __pyx_v_J_leak = (__pyx_v_v_2 * (__pyx_v_Ca2_NSR - __pyx_v_Ca2_i));
```

```
 473:
```

```
 474:
```

```
 475:                 J_xfer = (Ca2_ss - Ca2_i)/t_xfer
```

```
      __pyx_v_J_xfer = ((__pyx_v_Ca2_ss - __pyx_v_Ca2_i) / __pyx_v_t_xfer);
```

```
 476:
```

```
 477:
```

```
 478:                 J_up = v_3*((Ca2_i**2)/(k_mup**2 + Ca2_i**2))
```

```
      __pyx_v_J_up = (__pyx_v_v_3 * (pow(__pyx_v_Ca2_i, 2.0) / (pow(__pyx_v_k_mup, 2.0) + pow(__pyx_v_Ca2_i, 2.0))));
```

```
 479:
```

```
 480:
```

```
 481:                 J_trpn = (k_p_htrpn*Ca2_i*(HTRPN_tot - HTRPNCa) - k_n_htrpn*HTRPNCa
```

```
 482:                         + k_p_ltrpn*Ca2_i*(LTRPN_tot - LTRPNCa) - k_n_ltrpn*LTRPNCa)
```

```
      __pyx_v_J_trpn = (((((__pyx_v_k_p_htrpn * __pyx_v_Ca2_i) * (__pyx_v_HTRPN_tot - __pyx_v_HTRPNCa)) - (__pyx_v_k_n_htrpn * __pyx_v_HTRPNCa)) + ((__pyx_v_k_p_ltrpn * __pyx_v_Ca2_i) * (__pyx_v_LTRPN_tot - __pyx_v_LTRPNCa))) - (__pyx_v_k_n_ltrpn * __pyx_v_LTRPNCa));
```

```
 483:
```

```
 484:
```

```
 485:                 E_CaN = ((R*T)/(2*F))*log(Ca_0/Ca2_i)
```

```
      __pyx_v_E_CaN = (((__pyx_v_R * __pyx_v_T) / (2.0 * __pyx_v_F)) * log((__pyx_v_Ca_0 / __pyx_v_Ca2_i)));
```

```
 486:
```

```
 487:
```

```
 488:                 I_Cab = G_Cab*(V - E_CaN)
```

```
      __pyx_v_I_Cab = (__pyx_v_G_Cab * (__pyx_v_V - __pyx_v_E_CaN));
```

```
 489:
```

```
 490:
```

```
 491:                 I_CaL = G_CaL*O*(V - E_CaL)
```

```
      __pyx_v_I_CaL = ((__pyx_v_G_CaL * __pyx_v_O) * (__pyx_v_V - __pyx_v_E_CaL));
```

```
 492:
```

```
 493:
```

```
 494:                 I_NaCa = (k_NaCa*(1./(k_mNa**3 + Na_0**3))*(1./(k_mCa + Ca_0))*(1./(1 + k_sat*exp(((miu-1)*V*F)/(R*T))))*
```

```
      __pyx_v_I_NaCa = ((((__pyx_v_k_NaCa * (1. / (pow(__pyx_v_k_mNa, 3.0) + pow(__pyx_v_Na_0, 3.0)))) * (1. / (__pyx_v_k_mCa + __pyx_v_Ca_0))) * (1. / (1.0 + (__pyx_v_k_sat * exp(((((__pyx_v_miu - 1.0) * __pyx_v_V) * __pyx_v_F) / (__pyx_v_R * __pyx_v_T))))))) * (((exp((((__pyx_v_miu * __pyx_v_V) * __pyx_v_F) / (__pyx_v_R * __pyx_v_T))) * pow(__pyx_v_Na_i, 3.0)) * __pyx_v_Ca_0) - ((exp(((((__pyx_v_miu - 1.0) * __pyx_v_V) * __pyx_v_F) / (__pyx_v_R * __pyx_v_T))) * pow(__pyx_v_Na_0, 3.0)) * __pyx_v_Ca2_i)));
```

```
 495:                             (exp((miu*V*F)/(R*T))*Na_i**3*Ca_0 - exp((miu - 1)*V*F/(R*T))*Na_0**3*Ca2_i))
```

```
 496:
```

```
 497:
```

```
 498:                 I_pCa = I_maxpCa*((Ca2_i**2)/(k_mpCa**2 + Ca2_i**2))
```

```
      __pyx_v_I_pCa = (__pyx_v_I_maxpCa * (pow(__pyx_v_Ca2_i, 2.0) / (pow(__pyx_v_k_mpCa, 2.0) + pow(__pyx_v_Ca2_i, 2.0))));
```

```
 499:
```

```
 500:
```

```
 501:                 k1_Ca2_i = self.f_Ca2_i(B_i, J_leak, J_xfer, J_up, J_trpn, I_Cab, I_NaCa, I_pCa)
```

```
      __pyx_v_k1_Ca2_i = ((struct __pyx_vtabstruct_5model_Bondarenko *)__pyx_v_self->__pyx_vtab)->f_Ca2_i(__pyx_v_self, __pyx_v_B_i, __pyx_v_J_leak, __pyx_v_J_xfer, __pyx_v_J_up, __pyx_v_J_trpn, __pyx_v_I_Cab, __pyx_v_I_NaCa, __pyx_v_I_pCa);
```

```
 502:
```

```
 503:
```

```
 504:                 k2_Ca2_i = self.f_Ca2_i(B_i + (1./2)*k1_Ca2_i, J_leak + (1./2)*k1_Ca2_i, J_xfer + (1./2)*k1_Ca2_i, J_up + (1./2)*k1_Ca2_i,
```

```
      __pyx_v_k2_Ca2_i = ((struct __pyx_vtabstruct_5model_Bondarenko *)__pyx_v_self->__pyx_vtab)->f_Ca2_i(__pyx_v_self, (__pyx_v_B_i + ((1. / 2.0) * __pyx_v_k1_Ca2_i)), (__pyx_v_J_leak + ((1. / 2.0) * __pyx_v_k1_Ca2_i)), (__pyx_v_J_xfer + ((1. / 2.0) * __pyx_v_k1_Ca2_i)), (__pyx_v_J_up + ((1. / 2.0) * __pyx_v_k1_Ca2_i)), (__pyx_v_J_trpn + ((1. / 2.0) * __pyx_v_k1_Ca2_i)), (__pyx_v_I_Cab + ((1. / 2.0) * __pyx_v_k1_Ca2_i)), (__pyx_v_I_NaCa + ((1. / 2.0) * __pyx_v_k1_Ca2_i)), (__pyx_v_I_pCa + ((1. / 2.0) * __pyx_v_k1_Ca2_i)));
```

```
 505:                                  J_trpn + (1./2)*k1_Ca2_i, I_Cab + (1./2)*k1_Ca2_i, I_NaCa + (1./2)*k1_Ca2_i, I_pCa + (1./2)*k1_Ca2_i)
```

```
 506:
```

```
 507:
```

```
 508:                 k3_Ca2_i = self.f_Ca2_i(B_i + (1./2)*k2_Ca2_i, J_leak + (1./2)*k2_Ca2_i, J_xfer + (1./2)*k2_Ca2_i,J_up + (1./2)*k2_Ca2_i,
```

```
      __pyx_v_k3_Ca2_i = ((struct __pyx_vtabstruct_5model_Bondarenko *)__pyx_v_self->__pyx_vtab)->f_Ca2_i(__pyx_v_self, (__pyx_v_B_i + ((1. / 2.0) * __pyx_v_k2_Ca2_i)), (__pyx_v_J_leak + ((1. / 2.0) * __pyx_v_k2_Ca2_i)), (__pyx_v_J_xfer + ((1. / 2.0) * __pyx_v_k2_Ca2_i)), (__pyx_v_J_up + ((1. / 2.0) * __pyx_v_k2_Ca2_i)), (__pyx_v_J_trpn + ((1. / 2.0) * __pyx_v_k2_Ca2_i)), (__pyx_v_I_Cab + ((1. / 2.0) * __pyx_v_k2_Ca2_i)), (__pyx_v_I_NaCa + ((1. / 2.0) * __pyx_v_k2_Ca2_i)), (__pyx_v_I_pCa + ((1. / 2.0) * __pyx_v_k2_Ca2_i)));
```

```
 509:                                  J_trpn + (1./2)*k2_Ca2_i, I_Cab + (1./2)*k2_Ca2_i, I_NaCa + (1./2)*k2_Ca2_i, I_pCa + (1./2)*k2_Ca2_i)
```

```
 510:
```

```
 511:
```

```
 512:                 k4_Ca2_i = self.f_Ca2_i(B_i + k3_Ca2_i, J_leak + k3_Ca2_i, J_xfer + k3_Ca2_i, J_up + k3_Ca2_i,
```

```
      __pyx_v_k4_Ca2_i = ((struct __pyx_vtabstruct_5model_Bondarenko *)__pyx_v_self->__pyx_vtab)->f_Ca2_i(__pyx_v_self, (__pyx_v_B_i + __pyx_v_k3_Ca2_i), (__pyx_v_J_leak + __pyx_v_k3_Ca2_i), (__pyx_v_J_xfer + __pyx_v_k3_Ca2_i), (__pyx_v_J_up + __pyx_v_k3_Ca2_i), (__pyx_v_J_trpn + __pyx_v_k3_Ca2_i), (__pyx_v_I_Cab + __pyx_v_k3_Ca2_i), (__pyx_v_I_NaCa + __pyx_v_k3_Ca2_i), (__pyx_v_I_pCa + __pyx_v_k3_Ca2_i));
```

```
 513:                                  J_trpn + k3_Ca2_i, I_Cab + k3_Ca2_i, I_NaCa + k3_Ca2_i, I_pCa + k3_Ca2_i)
```

```
 514:
```

```
 515:
```

```
 516:                 dCa2_i = (1./6)*(k1_Ca2_i + 2*k2_Ca2_i + 2*k3_Ca2_i + k4_Ca2_i)
```

```
      __pyx_v_dCa2_i = ((1. / 6.0) * (((__pyx_v_k1_Ca2_i + (2.0 * __pyx_v_k2_Ca2_i)) + (2.0 * __pyx_v_k3_Ca2_i)) + __pyx_v_k4_Ca2_i));
```

```
 517:
```

```
 518:
```

```
 519:                 Pc1 = 1 - (Pc2 + Po1 + Po2)
```

```
      __pyx_v_Pc1 = (1.0 - ((__pyx_v_Pc2 + __pyx_v_Po1) + __pyx_v_Po2));
```

```
 520:
```

```
 521:
```

```
 522:                 k1_Po1 =  self.f_Po1(Ca2_ss, Pc1, Po1, Po2, Pc2)
```

```
      __pyx_v_k1_Po1 = ((struct __pyx_vtabstruct_5model_Bondarenko *)__pyx_v_self->__pyx_vtab)->f_Po1(__pyx_v_self, __pyx_v_Ca2_ss, __pyx_v_Pc1, __pyx_v_Po1, __pyx_v_Po2, __pyx_v_Pc2);
```

```
 523:
```

```
 524:                 k2_Po1 =  self.f_Po1(Ca2_ss + (1./2)*k1_Po1, Pc1 + (1./2)*k1_Po1, Po1  + (1./2)*k1_Po1,
```

```
      __pyx_v_k2_Po1 = ((struct __pyx_vtabstruct_5model_Bondarenko *)__pyx_v_self->__pyx_vtab)->f_Po1(__pyx_v_self, (__pyx_v_Ca2_ss + ((1. / 2.0) * __pyx_v_k1_Po1)), (__pyx_v_Pc1 + ((1. / 2.0) * __pyx_v_k1_Po1)), (__pyx_v_Po1 + ((1. / 2.0) * __pyx_v_k1_Po1)), (__pyx_v_Po2 + ((1. / 2.0) * __pyx_v_k1_Po1)), (__pyx_v_Pc2 + ((1. / 2.0) * __pyx_v_k1_Po1)));
```

```
 525:                               Po2 + (1./2)*k1_Po1, Pc2 + (1./2)*k1_Po1)
```

```
 526:
```

```
 527:                 k3_Po1 = self. f_Po1(Ca2_ss + (1./2)*k2_Po1, Pc1 + (1./2)*k2_Po1, Po1  + (1./2)*k2_Po1,
```

```
      __pyx_v_k3_Po1 = ((struct __pyx_vtabstruct_5model_Bondarenko *)__pyx_v_self->__pyx_vtab)->f_Po1(__pyx_v_self, (__pyx_v_Ca2_ss + ((1. / 2.0) * __pyx_v_k2_Po1)), (__pyx_v_Pc1 + ((1. / 2.0) * __pyx_v_k2_Po1)), (__pyx_v_Po1 + ((1. / 2.0) * __pyx_v_k2_Po1)), (__pyx_v_Po2 + ((1. / 2.0) * __pyx_v_k2_Po1)), (__pyx_v_Pc2 + ((1. / 2.0) * __pyx_v_k2_Po1)));
```

```
 528:                               Po2 + (1./2)*k2_Po1, Pc2 + (1./2)*k2_Po1)
```

```
 529:
```

```
 530:                 k4_Po1 = self.f_Po1(Ca2_ss + k3_Po1, Pc1 + k3_Po1, Po1 + k3_Po1, Po2 + k3_Po1, Pc2 + k3_Po1)
```

```
      __pyx_v_k4_Po1 = ((struct __pyx_vtabstruct_5model_Bondarenko *)__pyx_v_self->__pyx_vtab)->f_Po1(__pyx_v_self, (__pyx_v_Ca2_ss + __pyx_v_k3_Po1), (__pyx_v_Pc1 + __pyx_v_k3_Po1), (__pyx_v_Po1 + __pyx_v_k3_Po1), (__pyx_v_Po2 + __pyx_v_k3_Po1), (__pyx_v_Pc2 + __pyx_v_k3_Po1));
```

```
 531:
```

```
 532:
```

```
 533:                 dPo1 = (1./6)*(k1_Po1 + 2*k2_Po1 + 2*k3_Po1 + k4_Po1)
```

```
      __pyx_v_dPo1 = ((1. / 6.0) * (((__pyx_v_k1_Po1 + (2.0 * __pyx_v_k2_Po1)) + (2.0 * __pyx_v_k3_Po1)) + __pyx_v_k4_Po1));
```

```
 534:
```

```
 535:
```

```
 536:
```

```
 537:                 k1_Po2 = self.f_Po2(Ca2_ss, Po1, Po2)
```

```
      __pyx_v_k1_Po2 = ((struct __pyx_vtabstruct_5model_Bondarenko *)__pyx_v_self->__pyx_vtab)->f_Po2(__pyx_v_self, __pyx_v_Ca2_ss, __pyx_v_Po1, __pyx_v_Po2);
```

```
 538:
```

```
 539:                 k2_Po2 = self.f_Po2(Ca2_ss + (1./2)*k1_Po2, Po1 + (1./2)*k1_Po2, Po2 + (1./2)*k1_Po2)
```

```
      __pyx_v_k2_Po2 = ((struct __pyx_vtabstruct_5model_Bondarenko *)__pyx_v_self->__pyx_vtab)->f_Po2(__pyx_v_self, (__pyx_v_Ca2_ss + ((1. / 2.0) * __pyx_v_k1_Po2)), (__pyx_v_Po1 + ((1. / 2.0) * __pyx_v_k1_Po2)), (__pyx_v_Po2 + ((1. / 2.0) * __pyx_v_k1_Po2)));
```

```
 540:
```

```
 541:                 k3_Po2 = self.f_Po2(Ca2_ss + (1./2)*k2_Po2, Po1 + (1./2)*k2_Po2, Po2 + (1./2)*k2_Po2)
```

```
      __pyx_v_k3_Po2 = ((struct __pyx_vtabstruct_5model_Bondarenko *)__pyx_v_self->__pyx_vtab)->f_Po2(__pyx_v_self, (__pyx_v_Ca2_ss + ((1. / 2.0) * __pyx_v_k2_Po2)), (__pyx_v_Po1 + ((1. / 2.0) * __pyx_v_k2_Po2)), (__pyx_v_Po2 + ((1. / 2.0) * __pyx_v_k2_Po2)));
```

```
 542:
```

```
 543:                 k4_Po2 = self.f_Po2(Ca2_ss + k3_Po2, Po1 + k3_Po2, Po2 + k3_Po2)
```

```
      __pyx_v_k4_Po2 = ((struct __pyx_vtabstruct_5model_Bondarenko *)__pyx_v_self->__pyx_vtab)->f_Po2(__pyx_v_self, (__pyx_v_Ca2_ss + __pyx_v_k3_Po2), (__pyx_v_Po1 + __pyx_v_k3_Po2), (__pyx_v_Po2 + __pyx_v_k3_Po2));
```

```
 544:
```

```
 545:
```

```
 546:                 dPo2 = (1./6)*(k1_Po2 + 2*k2_Po2 + 2*k3_Po2 + k4_Po2)
```

```
      __pyx_v_dPo2 = ((1. / 6.0) * (((__pyx_v_k1_Po2 + (2.0 * __pyx_v_k2_Po2)) + (2.0 * __pyx_v_k3_Po2)) + __pyx_v_k4_Po2));
```

```
 547:
```

```
 548:
```

```
 549:
```

```
 550:                 B_ss = (1. + (CMDN_tot * k_CMDN_m)/(k_CMDN_m + Ca2_ss)**2)**(-1)
```

```
      __pyx_v_B_ss = pow((1. + ((__pyx_v_CMDN_tot * __pyx_v_k_CMDN_m) / pow((__pyx_v_k_CMDN_m + __pyx_v_Ca2_ss), 2.0))), -1.0);
```

```
 551:
```

```
 552:                 B_JSR = (1. + (CSQN_tot * k_CSQN_m)/(k_CSQN_m + Ca2_JSR)**2)**(-1)
```

```
      __pyx_v_B_JSR = pow((1. + ((__pyx_v_CSQN_tot * __pyx_v_k_CSQN_m) / pow((__pyx_v_k_CSQN_m + __pyx_v_Ca2_JSR), 2.0))), -1.0);
```

```
 553:
```

```
 554:                 J_rel = v_1*(Po1 + Po2)*(Ca2_NSR - Ca2_ss)*P_RyR
```

```
      __pyx_v_J_rel = (((__pyx_v_v_1 * (__pyx_v_Po1 + __pyx_v_Po2)) * (__pyx_v_Ca2_NSR - __pyx_v_Ca2_ss)) * __pyx_v_P_RyR);
```

```
 555:
```

```
 556:                 J_tr = (Ca2_NSR - Ca2_JSR)/t_tr
```

```
      __pyx_v_J_tr = ((__pyx_v_Ca2_NSR - __pyx_v_Ca2_JSR) / __pyx_v_t_tr);
```

```
 557:
```

```
 558:
```

```
 559:                 k1_Ca2_ss = self.f_Ca2_ss(B_ss, J_rel, J_xfer, I_CaL)
```

```
      __pyx_v_k1_Ca2_ss = ((struct __pyx_vtabstruct_5model_Bondarenko *)__pyx_v_self->__pyx_vtab)->f_Ca2_ss(__pyx_v_self, __pyx_v_B_ss, __pyx_v_J_rel, __pyx_v_J_xfer, __pyx_v_I_CaL);
```

```
 560:
```

```
 561:                 k2_Ca2_ss = self.f_Ca2_ss(B_ss + (1./2)*k1_Ca2_ss, J_rel + (1./2)*k1_Ca2_ss, J_xfer + (1./2)*k1_Ca2_ss, I_CaL + (1./2)*k1_Ca2_ss)
```

```
      __pyx_v_k2_Ca2_ss = ((struct __pyx_vtabstruct_5model_Bondarenko *)__pyx_v_self->__pyx_vtab)->f_Ca2_ss(__pyx_v_self, (__pyx_v_B_ss + ((1. / 2.0) * __pyx_v_k1_Ca2_ss)), (__pyx_v_J_rel + ((1. / 2.0) * __pyx_v_k1_Ca2_ss)), (__pyx_v_J_xfer + ((1. / 2.0) * __pyx_v_k1_Ca2_ss)), (__pyx_v_I_CaL + ((1. / 2.0) * __pyx_v_k1_Ca2_ss)));
```

```
 562:
```

```
 563:                 k3_Ca2_ss = self.f_Ca2_ss(B_ss + (1./2)*k2_Ca2_ss, J_rel + (1./2)*k2_Ca2_ss, J_xfer + (1./2)*k2_Ca2_ss, I_CaL + (1./2)*k2_Ca2_ss)
```

```
      __pyx_v_k3_Ca2_ss = ((struct __pyx_vtabstruct_5model_Bondarenko *)__pyx_v_self->__pyx_vtab)->f_Ca2_ss(__pyx_v_self, (__pyx_v_B_ss + ((1. / 2.0) * __pyx_v_k2_Ca2_ss)), (__pyx_v_J_rel + ((1. / 2.0) * __pyx_v_k2_Ca2_ss)), (__pyx_v_J_xfer + ((1. / 2.0) * __pyx_v_k2_Ca2_ss)), (__pyx_v_I_CaL + ((1. / 2.0) * __pyx_v_k2_Ca2_ss)));
```

```
 564:
```

```
 565:                 k4_Ca2_ss = self.f_Ca2_ss(B_ss + k3_Ca2_ss, J_rel + k3_Ca2_ss, J_xfer + k3_Ca2_ss, I_CaL + k3_Ca2_ss)
```

```
      __pyx_v_k4_Ca2_ss = ((struct __pyx_vtabstruct_5model_Bondarenko *)__pyx_v_self->__pyx_vtab)->f_Ca2_ss(__pyx_v_self, (__pyx_v_B_ss + __pyx_v_k3_Ca2_ss), (__pyx_v_J_rel + __pyx_v_k3_Ca2_ss), (__pyx_v_J_xfer + __pyx_v_k3_Ca2_ss), (__pyx_v_I_CaL + __pyx_v_k3_Ca2_ss));
```

```
 566:
```

```
 567:
```

```
 568:                 dCa2_ss =  (1./6)*(k1_Ca2_ss + 2*k2_Ca2_ss + 2*k3_Ca2_ss + k4_Ca2_ss)
```

```
      __pyx_v_dCa2_ss = ((1. / 6.0) * (((__pyx_v_k1_Ca2_ss + (2.0 * __pyx_v_k2_Ca2_ss)) + (2.0 * __pyx_v_k3_Ca2_ss)) + __pyx_v_k4_Ca2_ss));
```

```
 569:
```

```
 570:
```

```
 571:                 k1_Ca2_JSR = self.f_Ca2_JSR(B_JSR, J_tr, J_rel)
```

```
      __pyx_v_k1_Ca2_JSR = ((struct __pyx_vtabstruct_5model_Bondarenko *)__pyx_v_self->__pyx_vtab)->f_Ca2_JSR(__pyx_v_self, __pyx_v_B_JSR, __pyx_v_J_tr, __pyx_v_J_rel);
```

```
 572:
```

```
 573:                 k2_Ca2_JSR = self.f_Ca2_JSR(B_JSR + (1./2)*k1_Ca2_JSR, J_tr + (1./2)*k1_Ca2_JSR, J_rel + (1./2)*k1_Ca2_JSR)
```

```
      __pyx_v_k2_Ca2_JSR = ((struct __pyx_vtabstruct_5model_Bondarenko *)__pyx_v_self->__pyx_vtab)->f_Ca2_JSR(__pyx_v_self, (__pyx_v_B_JSR + ((1. / 2.0) * __pyx_v_k1_Ca2_JSR)), (__pyx_v_J_tr + ((1. / 2.0) * __pyx_v_k1_Ca2_JSR)), (__pyx_v_J_rel + ((1. / 2.0) * __pyx_v_k1_Ca2_JSR)));
```

```
 574:
```

```
 575:                 k3_Ca2_JSR = self.f_Ca2_JSR(B_JSR + (1./2)*k2_Ca2_JSR, J_tr + (1./2)*k2_Ca2_JSR, J_rel + (1./2)*k2_Ca2_JSR)
```

```
      __pyx_v_k3_Ca2_JSR = ((struct __pyx_vtabstruct_5model_Bondarenko *)__pyx_v_self->__pyx_vtab)->f_Ca2_JSR(__pyx_v_self, (__pyx_v_B_JSR + ((1. / 2.0) * __pyx_v_k2_Ca2_JSR)), (__pyx_v_J_tr + ((1. / 2.0) * __pyx_v_k2_Ca2_JSR)), (__pyx_v_J_rel + ((1. / 2.0) * __pyx_v_k2_Ca2_JSR)));
```

```
 576:
```

```
 577:                 k4_Ca2_JSR = self.f_Ca2_JSR(B_JSR + k3_Ca2_JSR, J_tr + k3_Ca2_JSR, J_rel + k3_Ca2_JSR)
```

```
      __pyx_v_k4_Ca2_JSR = ((struct __pyx_vtabstruct_5model_Bondarenko *)__pyx_v_self->__pyx_vtab)->f_Ca2_JSR(__pyx_v_self, (__pyx_v_B_JSR + __pyx_v_k3_Ca2_JSR), (__pyx_v_J_tr + __pyx_v_k3_Ca2_JSR), (__pyx_v_J_rel + __pyx_v_k3_Ca2_JSR));
```

```
 578:
```

```
 579:
```

```
 580:                 dCa2_JSR = (1./6)*(k1_Ca2_JSR + 2*k2_Ca2_JSR + 2*k3_Ca2_JSR + k4_Ca2_JSR)
```

```
      __pyx_v_dCa2_JSR = ((1. / 6.0) * (((__pyx_v_k1_Ca2_JSR + (2.0 * __pyx_v_k2_Ca2_JSR)) + (2.0 * __pyx_v_k3_Ca2_JSR)) + __pyx_v_k4_Ca2_JSR));
```

```
 581:
```

```
 582:
```

```
 583:
```

```
 584:                 k1_Ca2_NSR = self.f_Ca2_NSR( J_up, J_leak, J_tr)
```

```
      __pyx_v_k1_Ca2_NSR = ((struct __pyx_vtabstruct_5model_Bondarenko *)__pyx_v_self->__pyx_vtab)->f_Ca2_NSR(__pyx_v_self, __pyx_v_J_up, __pyx_v_J_leak, __pyx_v_J_tr);
```

```
 585:
```

```
 586:                 k2_Ca2_NSR = self.f_Ca2_NSR( J_up + (1./2)*k1_Ca2_NSR, J_leak + (1./2)*k1_Ca2_NSR, J_tr + (1./2)*k1_Ca2_NSR)
```

```
      __pyx_v_k2_Ca2_NSR = ((struct __pyx_vtabstruct_5model_Bondarenko *)__pyx_v_self->__pyx_vtab)->f_Ca2_NSR(__pyx_v_self, (__pyx_v_J_up + ((1. / 2.0) * __pyx_v_k1_Ca2_NSR)), (__pyx_v_J_leak + ((1. / 2.0) * __pyx_v_k1_Ca2_NSR)), (__pyx_v_J_tr + ((1. / 2.0) * __pyx_v_k1_Ca2_NSR)));
```

```
 587:
```

```
 588:                 k3_Ca2_NSR = self.f_Ca2_NSR( J_up + (1./2)*k2_Ca2_NSR, J_leak + (1./2)*k2_Ca2_NSR, J_tr + (1./2)*k2_Ca2_NSR)
```

```
      __pyx_v_k3_Ca2_NSR = ((struct __pyx_vtabstruct_5model_Bondarenko *)__pyx_v_self->__pyx_vtab)->f_Ca2_NSR(__pyx_v_self, (__pyx_v_J_up + ((1. / 2.0) * __pyx_v_k2_Ca2_NSR)), (__pyx_v_J_leak + ((1. / 2.0) * __pyx_v_k2_Ca2_NSR)), (__pyx_v_J_tr + ((1. / 2.0) * __pyx_v_k2_Ca2_NSR)));
```

```
 589:
```

```
 590:                 k4_Ca2_NSR = self.f_Ca2_NSR( J_up + k3_Ca2_NSR, J_leak + k3_Ca2_NSR, J_tr + k3_Ca2_NSR)
```

```
      __pyx_v_k4_Ca2_NSR = ((struct __pyx_vtabstruct_5model_Bondarenko *)__pyx_v_self->__pyx_vtab)->f_Ca2_NSR(__pyx_v_self, (__pyx_v_J_up + __pyx_v_k3_Ca2_NSR), (__pyx_v_J_leak + __pyx_v_k3_Ca2_NSR), (__pyx_v_J_tr + __pyx_v_k3_Ca2_NSR));
```

```
 591:
```

```
 592:
```

```
 593:                 dCa2_NSR = (1./6)*(k1_Ca2_NSR + 2*k2_Ca2_NSR + 2*k3_Ca2_NSR + k4_Ca2_NSR)
```

```
      __pyx_v_dCa2_NSR = ((1. / 6.0) * (((__pyx_v_k1_Ca2_NSR + (2.0 * __pyx_v_k2_Ca2_NSR)) + (2.0 * __pyx_v_k3_Ca2_NSR)) + __pyx_v_k4_Ca2_NSR));
```

```
 594:
```

```
 595:
```

```
 596:
```

```
 597:                 k1_P_RyR = self.f_P_RyR(P_RyR, I_CaL, V)
```

```
      __pyx_v_k1_P_RyR = ((struct __pyx_vtabstruct_5model_Bondarenko *)__pyx_v_self->__pyx_vtab)->f_P_RyR(__pyx_v_self, __pyx_v_P_RyR, __pyx_v_I_CaL, __pyx_v_V);
```

```
 598:
```

```
 599:                 k2_P_RyR = self.f_P_RyR(P_RyR + (1./2)*k1_P_RyR, I_CaL + (1./2)*k1_P_RyR, V + (1./2)*k1_P_RyR)
```

```
      __pyx_v_k2_P_RyR = ((struct __pyx_vtabstruct_5model_Bondarenko *)__pyx_v_self->__pyx_vtab)->f_P_RyR(__pyx_v_self, (__pyx_v_P_RyR + ((1. / 2.0) * __pyx_v_k1_P_RyR)), (__pyx_v_I_CaL + ((1. / 2.0) * __pyx_v_k1_P_RyR)), (__pyx_v_V + ((1. / 2.0) * __pyx_v_k1_P_RyR)));
```

```
 600:
```

```
 601:                 k3_P_RyR = self.f_P_RyR(P_RyR + (1./2)*k2_P_RyR, I_CaL + (1./2)*k2_P_RyR, V +( 1./2)*k2_P_RyR)
```

```
      __pyx_v_k3_P_RyR = ((struct __pyx_vtabstruct_5model_Bondarenko *)__pyx_v_self->__pyx_vtab)->f_P_RyR(__pyx_v_self, (__pyx_v_P_RyR + ((1. / 2.0) * __pyx_v_k2_P_RyR)), (__pyx_v_I_CaL + ((1. / 2.0) * __pyx_v_k2_P_RyR)), (__pyx_v_V + ((1. / 2.0) * __pyx_v_k2_P_RyR)));
```

```
 602:
```

```
 603:                 k4_P_RyR = self.f_P_RyR(P_RyR + k3_P_RyR, I_CaL + k3_P_RyR, V + k3_P_RyR)
```

```
      __pyx_v_k4_P_RyR = ((struct __pyx_vtabstruct_5model_Bondarenko *)__pyx_v_self->__pyx_vtab)->f_P_RyR(__pyx_v_self, (__pyx_v_P_RyR + __pyx_v_k3_P_RyR), (__pyx_v_I_CaL + __pyx_v_k3_P_RyR), (__pyx_v_V + __pyx_v_k3_P_RyR));
```

```
 604:
```

```
 605:
```

```
 606:                 dP_RyR = (1./6)*(k1_P_RyR + 2*k2_P_RyR + 2*k3_P_RyR + k4_P_RyR)
```

```
      __pyx_v_dP_RyR = ((1. / 6.0) * (((__pyx_v_k1_P_RyR + (2.0 * __pyx_v_k2_P_RyR)) + (2.0 * __pyx_v_k3_P_RyR)) + __pyx_v_k4_P_RyR));
```

```
 607:
```

```
 608:
```

```
 609:
```

```
 610:                 k1_LTRPNCa = self.f_LTRPNCa(Ca2_i, LTRPNCa)
```

```
      __pyx_v_k1_LTRPNCa = ((struct __pyx_vtabstruct_5model_Bondarenko *)__pyx_v_self->__pyx_vtab)->f_LTRPNCa(__pyx_v_self, __pyx_v_Ca2_i, __pyx_v_LTRPNCa);
```

```
 611:
```

```
 612:                 k2_LTRPNCa = self.f_LTRPNCa(Ca2_i + (1./2)*k1_LTRPNCa, LTRPNCa + (1./2)*k1_LTRPNCa)
```

```
      __pyx_v_k2_LTRPNCa = ((struct __pyx_vtabstruct_5model_Bondarenko *)__pyx_v_self->__pyx_vtab)->f_LTRPNCa(__pyx_v_self, (__pyx_v_Ca2_i + ((1. / 2.0) * __pyx_v_k1_LTRPNCa)), (__pyx_v_LTRPNCa + ((1. / 2.0) * __pyx_v_k1_LTRPNCa)));
```

```
 613:
```

```
 614:                 k3_LTRPNCa = self.f_LTRPNCa(Ca2_i + (1./2)*k2_LTRPNCa, LTRPNCa + (1./2)*k2_LTRPNCa)
```

```
      __pyx_v_k3_LTRPNCa = ((struct __pyx_vtabstruct_5model_Bondarenko *)__pyx_v_self->__pyx_vtab)->f_LTRPNCa(__pyx_v_self, (__pyx_v_Ca2_i + ((1. / 2.0) * __pyx_v_k2_LTRPNCa)), (__pyx_v_LTRPNCa + ((1. / 2.0) * __pyx_v_k2_LTRPNCa)));
```

```
 615:
```

```
 616:                 k4_LTRPNCa = self.f_LTRPNCa(Ca2_i + k3_LTRPNCa, LTRPNCa + k3_LTRPNCa)
```

```
      __pyx_v_k4_LTRPNCa = ((struct __pyx_vtabstruct_5model_Bondarenko *)__pyx_v_self->__pyx_vtab)->f_LTRPNCa(__pyx_v_self, (__pyx_v_Ca2_i + __pyx_v_k3_LTRPNCa), (__pyx_v_LTRPNCa + __pyx_v_k3_LTRPNCa));
```

```
 617:
```

```
 618:
```

```
 619:                 dLTRPNCa = (1./6)*(k1_LTRPNCa + 2*k2_LTRPNCa + 2*k3_LTRPNCa + k4_LTRPNCa)
```

```
      __pyx_v_dLTRPNCa = ((1. / 6.0) * (((__pyx_v_k1_LTRPNCa + (2.0 * __pyx_v_k2_LTRPNCa)) + (2.0 * __pyx_v_k3_LTRPNCa)) + __pyx_v_k4_LTRPNCa));
```

```
 620:
```

```
 621:
```

```
 622:                 k1_HTRPNCa = self.f_HTRPNCa(Ca2_i, HTRPNCa)
```

```
      __pyx_v_k1_HTRPNCa = ((struct __pyx_vtabstruct_5model_Bondarenko *)__pyx_v_self->__pyx_vtab)->f_HTRPNCa(__pyx_v_self, __pyx_v_Ca2_i, __pyx_v_HTRPNCa);
```

```
 623:
```

```
 624:                 k2_HTRPNCa = self.f_HTRPNCa(Ca2_i +( 1./2)*k1_HTRPNCa, HTRPNCa + (1./2)*k1_HTRPNCa)
```

```
      __pyx_v_k2_HTRPNCa = ((struct __pyx_vtabstruct_5model_Bondarenko *)__pyx_v_self->__pyx_vtab)->f_HTRPNCa(__pyx_v_self, (__pyx_v_Ca2_i + ((1. / 2.0) * __pyx_v_k1_HTRPNCa)), (__pyx_v_HTRPNCa + ((1. / 2.0) * __pyx_v_k1_HTRPNCa)));
```

```
 625:
```

```
 626:                 k3_HTRPNCa = self.f_HTRPNCa(Ca2_i + (1./2)*k2_HTRPNCa, HTRPNCa + (1./2)*k2_HTRPNCa)
```

```
      __pyx_v_k3_HTRPNCa = ((struct __pyx_vtabstruct_5model_Bondarenko *)__pyx_v_self->__pyx_vtab)->f_HTRPNCa(__pyx_v_self, (__pyx_v_Ca2_i + ((1. / 2.0) * __pyx_v_k2_HTRPNCa)), (__pyx_v_HTRPNCa + ((1. / 2.0) * __pyx_v_k2_HTRPNCa)));
```

```
 627:
```

```
 628:                 k4_HTRPNCa = self.f_HTRPNCa(Ca2_i + k3_HTRPNCa, HTRPNCa + k3_HTRPNCa)
```

```
      __pyx_v_k4_HTRPNCa = ((struct __pyx_vtabstruct_5model_Bondarenko *)__pyx_v_self->__pyx_vtab)->f_HTRPNCa(__pyx_v_self, (__pyx_v_Ca2_i + __pyx_v_k3_HTRPNCa), (__pyx_v_HTRPNCa + __pyx_v_k3_HTRPNCa));
```

```
 629:
```

```
 630:
```

```
 631:                 dHTRPNCa = (1./6)*(k1_HTRPNCa + 2*k2_HTRPNCa + 2*k3_HTRPNCa + k4_HTRPNCa)
```

```
      __pyx_v_dHTRPNCa = ((1. / 6.0) * (((__pyx_v_k1_HTRPNCa + (2.0 * __pyx_v_k2_HTRPNCa)) + (2.0 * __pyx_v_k3_HTRPNCa)) + __pyx_v_k4_HTRPNCa));
```

```
 632:
```

```
 633:
```

```
 634:
```

```
 635:                 k1_Pc2 = self.f_Pc2(Po1, Pc2)
```

```
      __pyx_v_k1_Pc2 = ((struct __pyx_vtabstruct_5model_Bondarenko *)__pyx_v_self->__pyx_vtab)->f_Pc2(__pyx_v_self, __pyx_v_Po1, __pyx_v_Pc2);
```

```
 636:
```

```
 637:                 k2_Pc2 = self.f_Pc2(Po1 + (1./2)*k1_Pc2, Pc2+ (1./2)*k1_Pc2)
```

```
      __pyx_v_k2_Pc2 = ((struct __pyx_vtabstruct_5model_Bondarenko *)__pyx_v_self->__pyx_vtab)->f_Pc2(__pyx_v_self, (__pyx_v_Po1 + ((1. / 2.0) * __pyx_v_k1_Pc2)), (__pyx_v_Pc2 + ((1. / 2.0) * __pyx_v_k1_Pc2)));
```

```
 638:
```

```
 639:                 k3_Pc2 = self.f_Pc2(Po1 + (1./2)*k2_Pc2, Pc2+ (1./2)*k2_Pc2)
```

```
      __pyx_v_k3_Pc2 = ((struct __pyx_vtabstruct_5model_Bondarenko *)__pyx_v_self->__pyx_vtab)->f_Pc2(__pyx_v_self, (__pyx_v_Po1 + ((1. / 2.0) * __pyx_v_k2_Pc2)), (__pyx_v_Pc2 + ((1. / 2.0) * __pyx_v_k2_Pc2)));
```

```
 640:
```

```
 641:                 k4_Pc2 = self.f_Pc2(Po1 + k3_Pc2, Pc2+ k3_Pc2)
```

```
      __pyx_v_k4_Pc2 = ((struct __pyx_vtabstruct_5model_Bondarenko *)__pyx_v_self->__pyx_vtab)->f_Pc2(__pyx_v_self, (__pyx_v_Po1 + __pyx_v_k3_Pc2), (__pyx_v_Pc2 + __pyx_v_k3_Pc2));
```

```
 642:
```

```
 643:
```

```
 644:                 dPc2 = (1./6)*(k1_Pc2 + 2*k2_Pc2 + 2*k3_Pc2 + k4_Pc2)
```

```
      __pyx_v_dPc2 = ((1. / 6.0) * (((__pyx_v_k1_Pc2 + (2.0 * __pyx_v_k2_Pc2)) + (2.0 * __pyx_v_k3_Pc2)) + __pyx_v_k4_Pc2));
```

```
 645:
```

```
 646:
```

```
 647:                 g = (k_pcmax*Ca2_ss)/(k_pchalf + Ca2_ss)
```

```
      __pyx_v_g = ((__pyx_v_k_pcmax * __pyx_v_Ca2_ss) / (__pyx_v_k_pchalf + __pyx_v_Ca2_ss));
```

```
 648:
```

```
 649:                 a = ((0.4*exp((V+12.0)/10.0)*(1 + 0.7*exp(-(V+40.0)**2/10.0) -
```

```
 650:                         0.75*exp(-(V+20.0)**2/400.0)))/(1 + 0.12*exp((V+12.0)/10.0)))
```

```
      __pyx_v_a = (((0.4 * exp(((__pyx_v_V + 12.0) / 10.0))) * ((1.0 + (0.7 * exp(((-pow((__pyx_v_V + 40.0), 2.0)) / 10.0)))) - (0.75 * exp(((-pow((__pyx_v_V + 20.0), 2.0)) / 400.0))))) / (1.0 + (0.12 * exp(((__pyx_v_V + 12.0) / 10.0)))));
```

```
 651:
```

```
 652:                 B = 0.05*exp(-(V+12.0)/13.0)
```

```
      __pyx_v_B = (0.05 * exp(((-(__pyx_v_V + 12.0)) / 13.0)));
```

```
 653:
```

```
 654:                 k_pcf = 13.0 *(1-exp(-((V+14.5)**2)/100.))
```

```
      __pyx_v_k_pcf = (13.0 * (1.0 - exp(((-pow((__pyx_v_V + 14.5), 2.0)) / 100.))));
```

```
 655:
```

```
 656:
```

```
 657:                 k1_O = self.f_O(a, C4, B, O, I1, g, I2, k_pcf)
```

```
      __pyx_v_k1_O = ((struct __pyx_vtabstruct_5model_Bondarenko *)__pyx_v_self->__pyx_vtab)->f_O(__pyx_v_self, __pyx_v_a, __pyx_v_C4, __pyx_v_B, __pyx_v_O, __pyx_v_I1, __pyx_v_g, __pyx_v_I2, __pyx_v_k_pcf);
```

```
 658:
```

```
 659:
```

```
 660:                 k2_O = self.f_O(a + (1./2)*k1_O, C4 + (1./2)*k1_O, B + (1./2)*k1_O, O + (1./2)*k1_O, I1 + (1./2)*k1_O, g + (1./2)*k1_O,
```

```
      __pyx_v_k2_O = ((struct __pyx_vtabstruct_5model_Bondarenko *)__pyx_v_self->__pyx_vtab)->f_O(__pyx_v_self, (__pyx_v_a + ((1. / 2.0) * __pyx_v_k1_O)), (__pyx_v_C4 + ((1. / 2.0) * __pyx_v_k1_O)), (__pyx_v_B + ((1. / 2.0) * __pyx_v_k1_O)), (__pyx_v_O + ((1. / 2.0) * __pyx_v_k1_O)), (__pyx_v_I1 + ((1. / 2.0) * __pyx_v_k1_O)), (__pyx_v_g + ((1. / 2.0) * __pyx_v_k1_O)), (__pyx_v_I2 + ((1. / 2.0) * __pyx_v_k1_O)), (__pyx_v_k_pcf + ((1. / 2.0) * __pyx_v_k1_O)));
```

```
 661:                          I2 + (1./2)*k1_O, k_pcf + (1./2)*k1_O )
```

```
 662:
```

```
 663:
```

```
 664:                 k3_O = self.f_O(a + (1./2)*k2_O, C4 + (1./2)*k2_O, B+(1./2)*k2_O, O + (1./2)*k2_O, I1 + (1./2)*k2_O, g + (1./2)*k1_O,
```

```
      __pyx_v_k3_O = ((struct __pyx_vtabstruct_5model_Bondarenko *)__pyx_v_self->__pyx_vtab)->f_O(__pyx_v_self, (__pyx_v_a + ((1. / 2.0) * __pyx_v_k2_O)), (__pyx_v_C4 + ((1. / 2.0) * __pyx_v_k2_O)), (__pyx_v_B + ((1. / 2.0) * __pyx_v_k2_O)), (__pyx_v_O + ((1. / 2.0) * __pyx_v_k2_O)), (__pyx_v_I1 + ((1. / 2.0) * __pyx_v_k2_O)), (__pyx_v_g + ((1. / 2.0) * __pyx_v_k1_O)), (__pyx_v_I2 + ((1. / 2.0) * __pyx_v_k2_O)), (__pyx_v_k_pcf + ((1. / 2.0) * __pyx_v_k2_O)));
```

```
 665:                          I2 + (1./2)*k2_O, k_pcf + (1./2)*k2_O )
```

```
 666:
```

```
 667:
```

```
 668:                 k4_O = self.f_O(a+k3_O, C4 + k3_O, B + k3_O, O + k3_O, I1 + k3_O, g + k3_O, I2 + k3_O, k_pcf + k3_O )
```

```
      __pyx_v_k4_O = ((struct __pyx_vtabstruct_5model_Bondarenko *)__pyx_v_self->__pyx_vtab)->f_O(__pyx_v_self, (__pyx_v_a + __pyx_v_k3_O), (__pyx_v_C4 + __pyx_v_k3_O), (__pyx_v_B + __pyx_v_k3_O), (__pyx_v_O + __pyx_v_k3_O), (__pyx_v_I1 + __pyx_v_k3_O), (__pyx_v_g + __pyx_v_k3_O), (__pyx_v_I2 + __pyx_v_k3_O), (__pyx_v_k_pcf + __pyx_v_k3_O));
```

```
 669:
```

```
 670:
```

```
 671:                 dO = (1./6)*(k1_O + 2*k2_O + 2*k3_O + k4_O)
```

```
      __pyx_v_dO = ((1. / 6.0) * (((__pyx_v_k1_O + (2.0 * __pyx_v_k2_O)) + (2.0 * __pyx_v_k3_O)) + __pyx_v_k4_O));
```

```
 672:
```

```
 673:
```

```
 674:
```

```
 675:                 C1 = 1 - (O + C2 + C3 + C4 + I1 + I2 + I3)
```

```
      __pyx_v_C1 = (1.0 - ((((((__pyx_v_O + __pyx_v_C2) + __pyx_v_C3) + __pyx_v_C4) + __pyx_v_I1) + __pyx_v_I2) + __pyx_v_I3));
```

```
 676:
```

```
 677:
```

```
 678:
```

```
 679:
```

```
 680:                 k1_c2 = self.f_C2(C1, C2, C3, a, B )
```

```
      __pyx_v_k1_c2 = ((struct __pyx_vtabstruct_5model_Bondarenko *)__pyx_v_self->__pyx_vtab)->f_C2(__pyx_v_self, __pyx_v_C1, __pyx_v_C2, __pyx_v_C3, __pyx_v_a, __pyx_v_B);
```

```
 681:
```

```
 682:
```

```
 683:                 k2_c2 = self.f_C2(C1+(1./2)*k1_c2, C2+(1./2)*k1_c2, C3+(1./2)*k1_c2, a+(1./2)*k1_c2, B+(1./2)*k1_c2)
```

```
      __pyx_v_k2_c2 = ((struct __pyx_vtabstruct_5model_Bondarenko *)__pyx_v_self->__pyx_vtab)->f_C2(__pyx_v_self, (__pyx_v_C1 + ((1. / 2.0) * __pyx_v_k1_c2)), (__pyx_v_C2 + ((1. / 2.0) * __pyx_v_k1_c2)), (__pyx_v_C3 + ((1. / 2.0) * __pyx_v_k1_c2)), (__pyx_v_a + ((1. / 2.0) * __pyx_v_k1_c2)), (__pyx_v_B + ((1. / 2.0) * __pyx_v_k1_c2)));
```

```
 684:
```

```
 685:
```

```
 686:                 k3_c2 = self.f_C2(C1+(1./2)*k2_c2, C2+(1./2)*k2_c2, C3+(1./2)*k2_c2, a+(1./2)*k2_c2, B+(1./2)*k2_c2)
```

```
      __pyx_v_k3_c2 = ((struct __pyx_vtabstruct_5model_Bondarenko *)__pyx_v_self->__pyx_vtab)->f_C2(__pyx_v_self, (__pyx_v_C1 + ((1. / 2.0) * __pyx_v_k2_c2)), (__pyx_v_C2 + ((1. / 2.0) * __pyx_v_k2_c2)), (__pyx_v_C3 + ((1. / 2.0) * __pyx_v_k2_c2)), (__pyx_v_a + ((1. / 2.0) * __pyx_v_k2_c2)), (__pyx_v_B + ((1. / 2.0) * __pyx_v_k2_c2)));
```

```
 687:
```

```
 688:
```

```
 689:                 k4_c2 = self.f_C2(C1 + k3_c2, C2 + k3_c2, C3 + k3_c2, a + k3_c2, B + k3_c2)
```

```
      __pyx_v_k4_c2 = ((struct __pyx_vtabstruct_5model_Bondarenko *)__pyx_v_self->__pyx_vtab)->f_C2(__pyx_v_self, (__pyx_v_C1 + __pyx_v_k3_c2), (__pyx_v_C2 + __pyx_v_k3_c2), (__pyx_v_C3 + __pyx_v_k3_c2), (__pyx_v_a + __pyx_v_k3_c2), (__pyx_v_B + __pyx_v_k3_c2));
```

```
 690:
```

```
 691:
```

```
 692:                 dC2 = (1./6)*(k1_c2 + 2*k2_c2 + 2*k3_c2 + k4_c2)
```

```
      __pyx_v_dC2 = ((1. / 6.0) * (((__pyx_v_k1_c2 + (2.0 * __pyx_v_k2_c2)) + (2.0 * __pyx_v_k3_c2)) + __pyx_v_k4_c2));
```

```
 693:
```

```
 694:
```

```
 695:
```

```
 696:                 k1_c3 = self.f_C3(C2, C3, C4, a, B)
```

```
      __pyx_v_k1_c3 = ((struct __pyx_vtabstruct_5model_Bondarenko *)__pyx_v_self->__pyx_vtab)->f_C3(__pyx_v_self, __pyx_v_C2, __pyx_v_C3, __pyx_v_C4, __pyx_v_a, __pyx_v_B);
```

```
 697:
```

```
 698:
```

```
 699:                 k2_c3 = self.f_C3(C2 + (1./2)*k1_c3, C3 + (1./2)*k1_c3, C4 + (1./2)*k1_c3, a + (1./2)*k1_c3, B + (1./2)*k1_c3)
```

```
      __pyx_v_k2_c3 = ((struct __pyx_vtabstruct_5model_Bondarenko *)__pyx_v_self->__pyx_vtab)->f_C3(__pyx_v_self, (__pyx_v_C2 + ((1. / 2.0) * __pyx_v_k1_c3)), (__pyx_v_C3 + ((1. / 2.0) * __pyx_v_k1_c3)), (__pyx_v_C4 + ((1. / 2.0) * __pyx_v_k1_c3)), (__pyx_v_a + ((1. / 2.0) * __pyx_v_k1_c3)), (__pyx_v_B + ((1. / 2.0) * __pyx_v_k1_c3)));
```

```
 700:
```

```
 701:
```

```
 702:                 k3_c3 = self.f_C3(C2 + (1./2)*k2_c3, C3 + (1./2)*k2_c3, C4 + (1./2)*k2_c3, a + (1./2)*k2_c3, B + (1./2)*k2_c3)
```

```
      __pyx_v_k3_c3 = ((struct __pyx_vtabstruct_5model_Bondarenko *)__pyx_v_self->__pyx_vtab)->f_C3(__pyx_v_self, (__pyx_v_C2 + ((1. / 2.0) * __pyx_v_k2_c3)), (__pyx_v_C3 + ((1. / 2.0) * __pyx_v_k2_c3)), (__pyx_v_C4 + ((1. / 2.0) * __pyx_v_k2_c3)), (__pyx_v_a + ((1. / 2.0) * __pyx_v_k2_c3)), (__pyx_v_B + ((1. / 2.0) * __pyx_v_k2_c3)));
```

```
 703:
```

```
 704:
```

```
 705:                 k4_c3 = self.f_C3(C2 + k3_c3, C3 + k3_c3, C4 + k3_c3, a + k3_c3, B + k3_c3)
```

```
      __pyx_v_k4_c3 = ((struct __pyx_vtabstruct_5model_Bondarenko *)__pyx_v_self->__pyx_vtab)->f_C3(__pyx_v_self, (__pyx_v_C2 + __pyx_v_k3_c3), (__pyx_v_C3 + __pyx_v_k3_c3), (__pyx_v_C4 + __pyx_v_k3_c3), (__pyx_v_a + __pyx_v_k3_c3), (__pyx_v_B + __pyx_v_k3_c3));
```

```
 706:
```

```
 707:
```

```
 708:                 dC3 = (1./6)*(k1_c3 + 2*k2_c3 + 2*k3_c3 + k4_c3)
```

```
      __pyx_v_dC3 = ((1. / 6.0) * (((__pyx_v_k1_c3 + (2.0 * __pyx_v_k2_c3)) + (2.0 * __pyx_v_k3_c3)) + __pyx_v_k4_c3));
```

```
 709:
```

```
 710:
```

```
 711:
```

```
 712:
```

```
 713:
```

```
 714:                 k1_c4 = self.f_C4(C3, C4, O, I1, I2, I3, a, B, g, k_pcf)
```

```
      __pyx_v_k1_c4 = ((struct __pyx_vtabstruct_5model_Bondarenko *)__pyx_v_self->__pyx_vtab)->f_C4(__pyx_v_self, __pyx_v_C3, __pyx_v_C4, __pyx_v_O, __pyx_v_I1, __pyx_v_I2, __pyx_v_I3, __pyx_v_a, __pyx_v_B, __pyx_v_g, __pyx_v_k_pcf);
```

```
 715:
```

```
 716:
```

```
 717:                 k2_c4 = self.f_C4(C3 + (1./2)*k1_c4, C4+(1./2)*k1_c4, O + (1./2)*k1_c4, I1 + (1./2)*k1_c4,
```

```
      __pyx_v_k2_c4 = ((struct __pyx_vtabstruct_5model_Bondarenko *)__pyx_v_self->__pyx_vtab)->f_C4(__pyx_v_self, (__pyx_v_C3 + ((1. / 2.0) * __pyx_v_k1_c4)), (__pyx_v_C4 + ((1. / 2.0) * __pyx_v_k1_c4)), (__pyx_v_O + ((1. / 2.0) * __pyx_v_k1_c4)), (__pyx_v_I1 + ((1. / 2.0) * __pyx_v_k1_c4)), (__pyx_v_I2 + ((1. / 2.0) * __pyx_v_k1_c4)), (__pyx_v_I3 + ((1. / 2.0) * __pyx_v_k1_c4)), (__pyx_v_a + ((1. / 2.0) * __pyx_v_k1_c4)), (__pyx_v_B + ((1. / 2.0) * __pyx_v_k1_c4)), (__pyx_v_g + ((1. / 2.0) * __pyx_v_k1_c4)), (__pyx_v_k_pcf + ((1. / 2.0) * __pyx_v_k1_c4)));
```

```
 718:                              I2 + (1./2)*k1_c4, I3+(1./2)*k1_c4, a + (1./2)*k1_c4, B + (1./2)*k1_c4, g + (1./2)*k1_c4, k_pcf + (1./2)*k1_c4)
```

```
 719:
```

```
 720:
```

```
 721:                 k3_c4 = self.f_C4(C3 + (1./2)*k2_c4, C4 + (1./2)*k2_c4, O + (1./2)*k2_c4, I1 + (1./2)*k2_c4,
```

```
      __pyx_v_k3_c4 = ((struct __pyx_vtabstruct_5model_Bondarenko *)__pyx_v_self->__pyx_vtab)->f_C4(__pyx_v_self, (__pyx_v_C3 + ((1. / 2.0) * __pyx_v_k2_c4)), (__pyx_v_C4 + ((1. / 2.0) * __pyx_v_k2_c4)), (__pyx_v_O + ((1. / 2.0) * __pyx_v_k2_c4)), (__pyx_v_I1 + ((1. / 2.0) * __pyx_v_k2_c4)), (__pyx_v_I2 + ((1. / 2.0) * __pyx_v_k2_c4)), (__pyx_v_I3 + ((1. / 2.0) * __pyx_v_k2_c4)), (__pyx_v_a + ((1. / 2.0) * __pyx_v_k2_c4)), (__pyx_v_B + ((1. / 2.0) * __pyx_v_k2_c4)), (__pyx_v_g + ((1. / 2.0) * __pyx_v_k2_c4)), (__pyx_v_k_pcf + ((1. / 2.0) * __pyx_v_k2_c4)));
```

```
 722:                              I2 + (1./2)*k2_c4, I3 + (1./2)*k2_c4, a + (1./2)*k2_c4, B + (1./2)*k2_c4, g + (1./2)*k2_c4, k_pcf + (1./2)*k2_c4)
```

```
 723:
```

```
 724:
```

```
 725:                 k4_c4 = self.f_C4(C3 + k3_c4, C4 + k3_c4, O + k3_c4, I1 + k3_c4,
```

```
      __pyx_v_k4_c4 = ((struct __pyx_vtabstruct_5model_Bondarenko *)__pyx_v_self->__pyx_vtab)->f_C4(__pyx_v_self, (__pyx_v_C3 + __pyx_v_k3_c4), (__pyx_v_C4 + __pyx_v_k3_c4), (__pyx_v_O + __pyx_v_k3_c4), (__pyx_v_I1 + __pyx_v_k3_c4), (__pyx_v_I2 + __pyx_v_k3_c4), (__pyx_v_I3 + __pyx_v_k3_c4), (__pyx_v_a + __pyx_v_k3_c4), (__pyx_v_B + __pyx_v_k3_c4), (__pyx_v_g + __pyx_v_k3_c4), (__pyx_v_k_pcf + __pyx_v_k3_c4));
```

```
 726:                              I2 + k3_c4, I3 + k3_c4, a + k3_c4, B + k3_c4, g + k3_c4, k_pcf + k3_c4)
```

```
 727:
```

```
 728:
```

```
 729:                 dC4 = (1./6)*(k1_c4 + 2*k2_c4 + 2*k3_c4 + k4_c4)
```

```
      __pyx_v_dC4 = ((1. / 6.0) * (((__pyx_v_k1_c4 + (2.0 * __pyx_v_k2_c4)) + (2.0 * __pyx_v_k3_c4)) + __pyx_v_k4_c4));
```

```
 730:
```

```
 731:
```

```
 732:
```

```
 733:
```

```
 734:                 k1_I1 = self.f_I1(O, I1, I3, C4,a, B, g, k_pcf )
```

```
      __pyx_v_k1_I1 = ((struct __pyx_vtabstruct_5model_Bondarenko *)__pyx_v_self->__pyx_vtab)->f_I1(__pyx_v_self, __pyx_v_O, __pyx_v_I1, __pyx_v_I3, __pyx_v_C4, __pyx_v_a, __pyx_v_B, __pyx_v_g, __pyx_v_k_pcf);
```

```
 735:
```

```
 736:
```

```
 737:                 k2_I1 = self.f_I1(O + (1./2)*k1_I1, I1 + (1./2)*k1_I1, I3 + (1./2)*k1_I1, C4 + (1./2)*k1_I1, a + (1./2)*k1_I1
```

```
      __pyx_v_k2_I1 = ((struct __pyx_vtabstruct_5model_Bondarenko *)__pyx_v_self->__pyx_vtab)->f_I1(__pyx_v_self, (__pyx_v_O + ((1. / 2.0) * __pyx_v_k1_I1)), (__pyx_v_I1 + ((1. / 2.0) * __pyx_v_k1_I1)), (__pyx_v_I3 + ((1. / 2.0) * __pyx_v_k1_I1)), (__pyx_v_C4 + ((1. / 2.0) * __pyx_v_k1_I1)), (__pyx_v_a + ((1. / 2.0) * __pyx_v_k1_I1)), (__pyx_v_B + ((1. / 2.0) * __pyx_v_k1_I1)), (__pyx_v_g + ((1. / 2.0) * __pyx_v_k1_I1)), (__pyx_v_k_pcf + ((1. / 2.0) * __pyx_v_k1_I1)));
```

```
 738:                         , B + (1./2)*k1_I1, g + (1./2)*k1_I1, k_pcf + (1./2)*k1_I1)
```

```
 739:
```

```
 740:
```

```
 741:                 k3_I1 = self.f_I1(O + (1./2)*k2_I1, I1 + (1./2)*k2_I1, I3 + (1./2)*k2_I1, C4 + (1./2)*k2_I1, a + (1./2)*k2_I1
```

```
      __pyx_v_k3_I1 = ((struct __pyx_vtabstruct_5model_Bondarenko *)__pyx_v_self->__pyx_vtab)->f_I1(__pyx_v_self, (__pyx_v_O + ((1. / 2.0) * __pyx_v_k2_I1)), (__pyx_v_I1 + ((1. / 2.0) * __pyx_v_k2_I1)), (__pyx_v_I3 + ((1. / 2.0) * __pyx_v_k2_I1)), (__pyx_v_C4 + ((1. / 2.0) * __pyx_v_k2_I1)), (__pyx_v_a + ((1. / 2.0) * __pyx_v_k2_I1)), (__pyx_v_B + ((1. / 2.0) * __pyx_v_k2_I1)), (__pyx_v_g + ((1. / 2.0) * __pyx_v_k2_I1)), (__pyx_v_k_pcf + ((1. / 2.0) * __pyx_v_k2_I1)));
```

```
 742:                         , B + (1./2)*k2_I1, g + (1./2)*k2_I1, k_pcf + (1./2)*k2_I1)
```

```
 743:
```

```
 744:
```

```
 745:                 k4_I1 = self.f_I1(O + k3_I1, I1 + k3_I1, I3 + k3_I1, C4 + k3_I1, a + k3_I1, B + k3_I1,
```

```
      __pyx_v_k4_I1 = ((struct __pyx_vtabstruct_5model_Bondarenko *)__pyx_v_self->__pyx_vtab)->f_I1(__pyx_v_self, (__pyx_v_O + __pyx_v_k3_I1), (__pyx_v_I1 + __pyx_v_k3_I1), (__pyx_v_I3 + __pyx_v_k3_I1), (__pyx_v_C4 + __pyx_v_k3_I1), (__pyx_v_a + __pyx_v_k3_I1), (__pyx_v_B + __pyx_v_k3_I1), (__pyx_v_g + __pyx_v_k3_I1), (__pyx_v_k_pcf + __pyx_v_k3_I1));
```

```
 746:                            g + k3_I1, k_pcf + k3_I1)
```

```
 747:
```

```
 748:
```

```
 749:                 dI1 = (1./6)*(k1_I1 + 2*k2_I1 + 2*k3_I1 + k4_I1)
```

```
      __pyx_v_dI1 = ((1. / 6.0) * (((__pyx_v_k1_I1 + (2.0 * __pyx_v_k2_I1)) + (2.0 * __pyx_v_k3_I1)) + __pyx_v_k4_I1));
```

```
 750:
```

```
 751:
```

```
 752:
```

```
 753:
```

```
 754:                 k1_I2 = self.f_I2(O, I2, I3, C4, a, B, g, k_pcf)
```

```
      __pyx_v_k1_I2 = ((struct __pyx_vtabstruct_5model_Bondarenko *)__pyx_v_self->__pyx_vtab)->f_I2(__pyx_v_self, __pyx_v_O, __pyx_v_I2, __pyx_v_I3, __pyx_v_C4, __pyx_v_a, __pyx_v_B, __pyx_v_g, __pyx_v_k_pcf);
```

```
 755:
```

```
 756:
```

```
 757:                 k2_I2 = self.f_I2(O + (1./2)*k1_I2, I2 + (1./2)*k1_I2, I3+(1./2)*k1_I2, C4+(1./2)*k1_I2, a+(1./2)*k1_I2,
```

```
      __pyx_v_k2_I2 = ((struct __pyx_vtabstruct_5model_Bondarenko *)__pyx_v_self->__pyx_vtab)->f_I2(__pyx_v_self, (__pyx_v_O + ((1. / 2.0) * __pyx_v_k1_I2)), (__pyx_v_I2 + ((1. / 2.0) * __pyx_v_k1_I2)), (__pyx_v_I3 + ((1. / 2.0) * __pyx_v_k1_I2)), (__pyx_v_C4 + ((1. / 2.0) * __pyx_v_k1_I2)), (__pyx_v_a + ((1. / 2.0) * __pyx_v_k1_I2)), (__pyx_v_B + ((1. / 2.0) * __pyx_v_k1_I2)), (__pyx_v_g + ((1. / 2.0) * __pyx_v_k1_I2)), (__pyx_v_k_pcf + ((1. / 2.0) * __pyx_v_k1_I2)));
```

```
 758:                            B+(1./2)*k1_I2, g+(1./2)*k1_I2, k_pcf+(1./2)*k1_I2)
```

```
 759:
```

```
 760:
```

```
 761:                 k3_I2 = self.f_I2(O + (1./2)*k2_I2, I2 + (1./2)*k2_I2, I3 + (1./2)*k2_I2, C4 + (1./2)*k2_I2, a+(1./2)*k2_I2,
```

```
      __pyx_v_k3_I2 = ((struct __pyx_vtabstruct_5model_Bondarenko *)__pyx_v_self->__pyx_vtab)->f_I2(__pyx_v_self, (__pyx_v_O + ((1. / 2.0) * __pyx_v_k2_I2)), (__pyx_v_I2 + ((1. / 2.0) * __pyx_v_k2_I2)), (__pyx_v_I3 + ((1. / 2.0) * __pyx_v_k2_I2)), (__pyx_v_C4 + ((1. / 2.0) * __pyx_v_k2_I2)), (__pyx_v_a + ((1. / 2.0) * __pyx_v_k2_I2)), (__pyx_v_B + ((1. / 2.0) * __pyx_v_k2_I2)), (__pyx_v_g + ((1. / 2.0) * __pyx_v_k2_I2)), (__pyx_v_k_pcf + ((1. / 2.0) * __pyx_v_k2_I2)));
```

```
 762:                            B + (1./2)*k2_I2, g + (1./2)*k2_I2, k_pcf + (1./2)*k2_I2)
```

```
 763:
```

```
 764:
```

```
 765:                 k4_I2 = self.f_I2(O + k3_I2, I2 + k3_I2, I3 + k3_I2, C4 + k3_I2, a + k3_I2, B + k3_I2,
```

```
      __pyx_v_k4_I2 = ((struct __pyx_vtabstruct_5model_Bondarenko *)__pyx_v_self->__pyx_vtab)->f_I2(__pyx_v_self, (__pyx_v_O + __pyx_v_k3_I2), (__pyx_v_I2 + __pyx_v_k3_I2), (__pyx_v_I3 + __pyx_v_k3_I2), (__pyx_v_C4 + __pyx_v_k3_I2), (__pyx_v_a + __pyx_v_k3_I2), (__pyx_v_B + __pyx_v_k3_I2), (__pyx_v_g + __pyx_v_k3_I2), (__pyx_v_k_pcf + __pyx_v_k3_I2));
```

```
 766:                            g + k3_I2, k_pcf + k3_I2)
```

```
 767:
```

```
 768:
```

```
 769:
```

```
 770:                 dI2 = (1./6)*(k1_I2 + 2*k2_I2 + 2*k3_I2 + k4_I2)
```

```
      __pyx_v_dI2 = ((1. / 6.0) * (((__pyx_v_k1_I2 + (2.0 * __pyx_v_k2_I2)) + (2.0 * __pyx_v_k3_I2)) + __pyx_v_k4_I2));
```

```
 771:
```

```
 772:
```

```
 773:
```

```
 774:
```

```
 775:                 k1_I3 = self.f_I3(I1, I2, I3, C4, a, B, g, k_pcf)
```

```
      __pyx_v_k1_I3 = ((struct __pyx_vtabstruct_5model_Bondarenko *)__pyx_v_self->__pyx_vtab)->f_I3(__pyx_v_self, __pyx_v_I1, __pyx_v_I2, __pyx_v_I3, __pyx_v_C4, __pyx_v_a, __pyx_v_B, __pyx_v_g, __pyx_v_k_pcf);
```

```
 776:
```

```
 777:
```

```
 778:                 k2_I3 = self.f_I3(I1 + (1./2)*k1_I3, I2 + (1./2)*k1_I3, I3 + (1./2)*k1_I3, C4 + (1./2)*k1_I3, a + (1./2)*k1_I3,
```

```
      __pyx_v_k2_I3 = ((struct __pyx_vtabstruct_5model_Bondarenko *)__pyx_v_self->__pyx_vtab)->f_I3(__pyx_v_self, (__pyx_v_I1 + ((1. / 2.0) * __pyx_v_k1_I3)), (__pyx_v_I2 + ((1. / 2.0) * __pyx_v_k1_I3)), (__pyx_v_I3 + ((1. / 2.0) * __pyx_v_k1_I3)), (__pyx_v_C4 + ((1. / 2.0) * __pyx_v_k1_I3)), (__pyx_v_a + ((1. / 2.0) * __pyx_v_k1_I3)), (__pyx_v_B + ((1. / 2.0) * __pyx_v_k1_I3)), (__pyx_v_g + ((1. / 2.0) * __pyx_v_k1_I3)), (__pyx_v_k_pcf + ((1. / 2.0) * __pyx_v_k1_I3)));
```

```
 779:                            B + (1./2)*k1_I3, g + (1./2)*k1_I3, k_pcf + (1./2)*k1_I3)
```

```
 780:
```

```
 781:
```

```
 782:                 k3_I3 = self.f_I3(I1 + (1./2)*k2_I3, I2 + (1./2)*k2_I3, I3 + (1./2)*k2_I3, C4 + (1./2)*k2_I3, a+ (1./2)*k2_I3,
```

```
      __pyx_v_k3_I3 = ((struct __pyx_vtabstruct_5model_Bondarenko *)__pyx_v_self->__pyx_vtab)->f_I3(__pyx_v_self, (__pyx_v_I1 + ((1. / 2.0) * __pyx_v_k2_I3)), (__pyx_v_I2 + ((1. / 2.0) * __pyx_v_k2_I3)), (__pyx_v_I3 + ((1. / 2.0) * __pyx_v_k2_I3)), (__pyx_v_C4 + ((1. / 2.0) * __pyx_v_k2_I3)), (__pyx_v_a + ((1. / 2.0) * __pyx_v_k2_I3)), (__pyx_v_B + ((1. / 2.0) * __pyx_v_k2_I3)), (__pyx_v_g + ((1. / 2.0) * __pyx_v_k2_I3)), (__pyx_v_k_pcf + ((1. / 2.0) * __pyx_v_k2_I3)));
```

```
 783:                            B + (1./2)*k2_I3, g+(1./2)*k2_I3, k_pcf+(1./2)*k2_I3)
```

```
 784:
```

```
 785:
```

```
 786:                 k4_I3 = self.f_I3(I1 + k3_I3, I2 + k3_I3, I3 + k3_I3, C4 + k3_I3, a + k3_I3, B + k3_I3,
```

```
      __pyx_v_k4_I3 = ((struct __pyx_vtabstruct_5model_Bondarenko *)__pyx_v_self->__pyx_vtab)->f_I3(__pyx_v_self, (__pyx_v_I1 + __pyx_v_k3_I3), (__pyx_v_I2 + __pyx_v_k3_I3), (__pyx_v_I3 + __pyx_v_k3_I3), (__pyx_v_C4 + __pyx_v_k3_I3), (__pyx_v_a + __pyx_v_k3_I3), (__pyx_v_B + __pyx_v_k3_I3), (__pyx_v_g + __pyx_v_k3_I3), (__pyx_v_k_pcf + __pyx_v_k3_I3));
```

```
 787:                                   g + k3_I3, k_pcf + k3_I3)
```

```
 788:
```

```
 789:
```

```
 790:                 dI3 = (1./6)*(k1_I3 + 2*k2_I3 + 2*k3_I3 + k4_I3)
```

```
      __pyx_v_dI3 = ((1. / 6.0) * (((__pyx_v_k1_I3 + (2.0 * __pyx_v_k2_I3)) + (2.0 * __pyx_v_k3_I3)) + __pyx_v_k4_I3));
```

```
 791:
```

```
 792:
```

```
 793:
```

```
 794:
```

```
 795:                 E_Na = (R*T/F)*log((0.9*Na_0 + 0.1*K_0)/(0.9*Na_i + 0.1*K_i))
```

```
      __pyx_v_E_Na = (((__pyx_v_R * __pyx_v_T) / __pyx_v_F) * log((((0.9 * __pyx_v_Na_0) + (0.1 * __pyx_v_K_0)) / ((0.9 * __pyx_v_Na_i) + (0.1 * __pyx_v_K_i)))));
```

```
 796:
```

```
 797:                 I_Na = G_Na*O_Na*(V - E_Na)
```

```
      __pyx_v_I_Na = ((__pyx_v_G_Na * __pyx_v_O_Na) * (__pyx_v_V - __pyx_v_E_Na));
```

```
 798:
```

```
 799:                 I_Nab = G_Nab*(V - E_Na)
```

```
      __pyx_v_I_Nab = (__pyx_v_G_Nab * (__pyx_v_V - __pyx_v_E_Na));
```

```
 800:
```

```
 801:
```

```
 802:                 sigma = (1./7)*(exp(Na_0/67300.0)-1)
```

```
      __pyx_v_sigma = ((1. / 7.0) * (exp((__pyx_v_Na_0 / 67300.0)) - 1.0));
```

```
 803:
```

```
 804:                 f_NaK = 1./(1 + 0.1245*exp((-0.1*V*F)/(R*T)) + 0.0365*sigma*exp
```

```
      __pyx_v_f_NaK = (1. / ((1.0 + (0.1245 * exp((((-0.1 * __pyx_v_V) * __pyx_v_F) / (__pyx_v_R * __pyx_v_T))))) + ((0.0365 * __pyx_v_sigma) * exp((((-__pyx_v_V) * __pyx_v_F) / (__pyx_v_R * __pyx_v_T))))));
```

```
 805:                      ((-V*F)/(R*T)))
```

```
 806:
```

```
 807:                 I_NaK = I_maxNaK*f_NaK*(1./(1 + (K_mNai/Na_i)**(3./2)))*((K_0)/(K_0 + K_mKo))
```

```
      __pyx_v_I_NaK = (((__pyx_v_I_maxNaK * __pyx_v_f_NaK) * (1. / (1.0 + pow((__pyx_v_K_mNai / __pyx_v_Na_i), (3. / 2.0))))) * (__pyx_v_K_0 / (__pyx_v_K_0 + __pyx_v_K_mKo)));
```

```
 808:
```

```
 809:
```

```
 810:
```

```
 811:                 k1_Na_i = self.f_Na_i(I_Na, I_Nab, I_NaCa, I_NaK)
```

```
      __pyx_v_k1_Na_i = ((struct __pyx_vtabstruct_5model_Bondarenko *)__pyx_v_self->__pyx_vtab)->f_Na_i(__pyx_v_self, __pyx_v_I_Na, __pyx_v_I_Nab, __pyx_v_I_NaCa, __pyx_v_I_NaK);
```

```
 812:
```

```
 813:                 k2_Na_i = self.f_Na_i(I_Na + (1./2)*k1_Na_i, I_Nab + (1./2)*k1_Na_i, I_NaCa+(1./2)*k1_Na_i, I_NaK + (1./2)*k1_Na_i)
```

```
      __pyx_v_k2_Na_i = ((struct __pyx_vtabstruct_5model_Bondarenko *)__pyx_v_self->__pyx_vtab)->f_Na_i(__pyx_v_self, (__pyx_v_I_Na + ((1. / 2.0) * __pyx_v_k1_Na_i)), (__pyx_v_I_Nab + ((1. / 2.0) * __pyx_v_k1_Na_i)), (__pyx_v_I_NaCa + ((1. / 2.0) * __pyx_v_k1_Na_i)), (__pyx_v_I_NaK + ((1. / 2.0) * __pyx_v_k1_Na_i)));
```

```
 814:
```

```
 815:                 k3_Na_i = self.f_Na_i(I_Na + (1./2)*k2_Na_i, I_Nab + (1./2)*k2_Na_i, I_NaCa+(1./2)*k2_Na_i, I_NaK + (1./2)*k2_Na_i)
```

```
      __pyx_v_k3_Na_i = ((struct __pyx_vtabstruct_5model_Bondarenko *)__pyx_v_self->__pyx_vtab)->f_Na_i(__pyx_v_self, (__pyx_v_I_Na + ((1. / 2.0) * __pyx_v_k2_Na_i)), (__pyx_v_I_Nab + ((1. / 2.0) * __pyx_v_k2_Na_i)), (__pyx_v_I_NaCa + ((1. / 2.0) * __pyx_v_k2_Na_i)), (__pyx_v_I_NaK + ((1. / 2.0) * __pyx_v_k2_Na_i)));
```

```
 816:
```

```
 817:                 k4_Na_i = self.f_Na_i(I_Na + k3_Na_i, I_Nab + k3_Na_i, I_NaCa + k3_Na_i, I_NaK + k3_Na_i)
```

```
      __pyx_v_k4_Na_i = ((struct __pyx_vtabstruct_5model_Bondarenko *)__pyx_v_self->__pyx_vtab)->f_Na_i(__pyx_v_self, (__pyx_v_I_Na + __pyx_v_k3_Na_i), (__pyx_v_I_Nab + __pyx_v_k3_Na_i), (__pyx_v_I_NaCa + __pyx_v_k3_Na_i), (__pyx_v_I_NaK + __pyx_v_k3_Na_i));
```

```
 818:
```

```
 819:
```

```
 820:                 dNa_i = (1./6)*(k1_Na_i + 2*k2_Na_i + 2*k3_Na_i + k4_Na_i)
```

```
      __pyx_v_dNa_i = ((1. / 6.0) * (((__pyx_v_k1_Na_i + (2.0 * __pyx_v_k2_Na_i)) + (2.0 * __pyx_v_k3_Na_i)) + __pyx_v_k4_Na_i));
```

```
 821:
```

```
 822:
```

```
 823:
```

```
 824:                 C_Na3 = 1 - (O_Na + C_Na1 + C_Na2 + IF_Na + I1_Na + I2_Na + IC_Na2 + IC_Na3)
```

```
      __pyx_v_C_Na3 = (1.0 - (((((((__pyx_v_O_Na + __pyx_v_C_Na1) + __pyx_v_C_Na2) + __pyx_v_IF_Na) + __pyx_v_I1_Na) + __pyx_v_I2_Na) + __pyx_v_IC_Na2) + __pyx_v_IC_Na3));
```

```
 825:
```

```
 826:                 a_Na11 = 3.802/(0.1027*exp(-(V+2.5)/17.) + 0.2*exp(-(V+2.5)/150.))
```

```
      __pyx_v_a_Na11 = (3.802 / ((0.1027 * exp(((-(__pyx_v_V + 2.5)) / 17.))) + (0.2 * exp(((-(__pyx_v_V + 2.5)) / 150.)))));
```

```
 827:
```

```
 828:                 B_Na11 = 0.1917*exp(-(V+2.5)/20.3)
```

```
      __pyx_v_B_Na11 = (0.1917 * exp(((-(__pyx_v_V + 2.5)) / 20.3)));
```

```
 829:
```

```
 830:                 B_Na12 = 0.2*exp(-(V-2.5)/20.3)
```

```
      __pyx_v_B_Na12 = (0.2 * exp(((-(__pyx_v_V - 2.5)) / 20.3)));
```

```
 831:
```

```
 832:                 B_Na13 = 0.22*exp(-(V-7.5)/20.3)
```

```
      __pyx_v_B_Na13 = (0.22 * exp(((-(__pyx_v_V - 7.5)) / 20.3)));
```

```
 833:
```

```
 834:                 a_Na3 = 7e-7*exp(-(V+7.)/7.7)
```

```
      __pyx_v_a_Na3 = (7e-7 * exp(((-(__pyx_v_V + 7.)) / 7.7)));
```

```
 835:
```

```
 836:                 B_Na3 = 0.0084 + 0.00002*(V + 7.)
```

```
      __pyx_v_B_Na3 = (0.0084 + (0.00002 * (__pyx_v_V + 7.)));
```

```
 837:
```

```
 838:                 a_Na2 = 1./(0.188495*exp(-(V+7.)/16.6) + 0.393956)
```

```
      __pyx_v_a_Na2 = (1. / ((0.188495 * exp(((-(__pyx_v_V + 7.)) / 16.6))) + 0.393956));
```

```
 839:
```

```
 840:                 a_Na13 = 3.802/(0.1027*exp(-(V+2.5)/12.) + 0.25*exp(-(V+2.5)/150.))
```

```
      __pyx_v_a_Na13 = (3.802 / ((0.1027 * exp(((-(__pyx_v_V + 2.5)) / 12.))) + (0.25 * exp(((-(__pyx_v_V + 2.5)) / 150.)))));
```

```
 841:
```

```
 842:                 B_Na2 = (a_Na13*a_Na2*a_Na3)/(B_Na13*B_Na3)
```

```
      __pyx_v_B_Na2 = (((__pyx_v_a_Na13 * __pyx_v_a_Na2) * __pyx_v_a_Na3) / (__pyx_v_B_Na13 * __pyx_v_B_Na3));
```

```
 843:
```

```
 844:                 a_Na4 = a_Na2/1000.0
```

```
      __pyx_v_a_Na4 = (__pyx_v_a_Na2 / 1000.0);
```

```
 845:
```

```
 846:                 B_Na4 = a_Na3
```

```
      __pyx_v_B_Na4 = __pyx_v_a_Na3;
```

```
 847:
```

```
 848:                 a_Na5 = a_Na2/95000.
```

```
      __pyx_v_a_Na5 = (__pyx_v_a_Na2 / 95000.);
```

```
 849:
```

```
 850:                 B_Na5 = a_Na3/50.
```

```
      __pyx_v_B_Na5 = (__pyx_v_a_Na3 / 50.);
```

```
 851:
```

```
 852:                 a_Na12 = 3.802/(0.1027*exp(-(V + 2.5)/15.) + 0.23*exp(-(V + 2.5)/150.))
```

```
      __pyx_v_a_Na12 = (3.802 / ((0.1027 * exp(((-(__pyx_v_V + 2.5)) / 15.))) + (0.23 * exp(((-(__pyx_v_V + 2.5)) / 150.)))));
```

```
 853:
```

```
 854:
```

```
 855:
```

```
 856:                 k1_C_Na2 = self.f_C_Na2(a_Na11, C_Na3, B_Na11, C_Na2, B_Na12,
```

```
      __pyx_v_k1_C_Na2 = ((struct __pyx_vtabstruct_5model_Bondarenko *)__pyx_v_self->__pyx_vtab)->f_C_Na2(__pyx_v_self, __pyx_v_a_Na11, __pyx_v_C_Na3, __pyx_v_B_Na11, __pyx_v_C_Na2, __pyx_v_B_Na12, __pyx_v_a_Na12, __pyx_v_a_Na3, __pyx_v_IC_Na2, __pyx_v_B_Na3, __pyx_v_C_Na1);
```

```
 857:                                    a_Na12, a_Na3, IC_Na2, B_Na3, C_Na1)
```

```
 858:
```

```
 859:                 k2_C_Na2 = self.f_C_Na2(a_Na11 + (1./2)*k1_C_Na2, C_Na3 + (1./2)*k1_C_Na2,
```

```
      __pyx_v_k2_C_Na2 = ((struct __pyx_vtabstruct_5model_Bondarenko *)__pyx_v_self->__pyx_vtab)->f_C_Na2(__pyx_v_self, (__pyx_v_a_Na11 + ((1. / 2.0) * __pyx_v_k1_C_Na2)), (__pyx_v_C_Na3 + ((1. / 2.0) * __pyx_v_k1_C_Na2)), (__pyx_v_B_Na11 + ((1. / 2.0) * __pyx_v_k1_C_Na2)), (__pyx_v_C_Na2 + ((1. / 2.0) * __pyx_v_k1_C_Na2)), (__pyx_v_B_Na12 + ((1. / 2.0) * __pyx_v_k1_C_Na2)), (__pyx_v_a_Na12 + ((1. / 2.0) * __pyx_v_k1_C_Na2)), (__pyx_v_a_Na3 + ((1. / 2.0) * __pyx_v_k1_C_Na2)), (__pyx_v_IC_Na2 + ((1. / 2.0) * __pyx_v_k1_C_Na2)), (__pyx_v_B_Na3 + ((1. / 2.0) * __pyx_v_k1_C_Na2)), (__pyx_v_C_Na1 + ((1. / 2.0) * __pyx_v_k1_C_Na2)));
```

```
 860:                                    B_Na11 + (1./2)*k1_C_Na2 , C_Na2 + (1./2)*k1_C_Na2, B_Na12 + (1./2)*k1_C_Na2,
```

```
 861:                                    a_Na12 + (1./2)*k1_C_Na2, a_Na3 + (1./2)*k1_C_Na2, IC_Na2 + (1./2)*k1_C_Na2,
```

```
 862:                                    B_Na3 + (1./2)*k1_C_Na2, C_Na1 + (1./2)*k1_C_Na2)
```

```
 863:
```

```
 864:                 k3_C_Na2 = self.f_C_Na2(a_Na11 + (1./2)*k2_C_Na2, C_Na3 + (1./2)*k2_C_Na2,
```

```
      __pyx_v_k3_C_Na2 = ((struct __pyx_vtabstruct_5model_Bondarenko *)__pyx_v_self->__pyx_vtab)->f_C_Na2(__pyx_v_self, (__pyx_v_a_Na11 + ((1. / 2.0) * __pyx_v_k2_C_Na2)), (__pyx_v_C_Na3 + ((1. / 2.0) * __pyx_v_k2_C_Na2)), (__pyx_v_B_Na11 + ((1. / 2.0) * __pyx_v_k2_C_Na2)), (__pyx_v_C_Na2 + ((1. / 2.0) * __pyx_v_k2_C_Na2)), (__pyx_v_B_Na12 + ((1. / 2.0) * __pyx_v_k2_C_Na2)), (__pyx_v_a_Na12 + ((1. / 2.0) * __pyx_v_k2_C_Na2)), __pyx_v_a_Na3, (__pyx_v_IC_Na2 + ((1. / 2.0) * __pyx_v_k2_C_Na2)), (__pyx_v_B_Na3 + ((1. / 2.0) * __pyx_v_k2_C_Na2)), (__pyx_v_C_Na1 + ((1. / 2.0) * __pyx_v_k2_C_Na2)));
```

```
 865:                                    B_Na11 + (1./2)*k2_C_Na2 , C_Na2 + (1./2)*k2_C_Na2,
```

```
 866:                                    B_Na12 + (1./2)*k2_C_Na2, a_Na12 + (1./2)*k2_C_Na2,
```

```
 867:                                    a_Na3, IC_Na2 + (1./2)*k2_C_Na2, B_Na3 + (1./2)*k2_C_Na2,
```

```
 868:                                    C_Na1 + (1./2)*k2_C_Na2)
```

```
 869:
```

```
 870:                 k4_C_Na2 = self.f_C_Na2(a_Na11 + k3_C_Na2, C_Na3 + k3_C_Na2, B_Na11 + k3_C_Na2 ,
```

```
      __pyx_v_k4_C_Na2 = ((struct __pyx_vtabstruct_5model_Bondarenko *)__pyx_v_self->__pyx_vtab)->f_C_Na2(__pyx_v_self, (__pyx_v_a_Na11 + __pyx_v_k3_C_Na2), (__pyx_v_C_Na3 + __pyx_v_k3_C_Na2), (__pyx_v_B_Na11 + __pyx_v_k3_C_Na2), (__pyx_v_C_Na2 + __pyx_v_k3_C_Na2), (__pyx_v_B_Na12 + __pyx_v_k3_C_Na2), (__pyx_v_a_Na12 + __pyx_v_k3_C_Na2), (__pyx_v_a_Na3 + __pyx_v_k3_C_Na2), (__pyx_v_IC_Na2 + __pyx_v_k3_C_Na2), (__pyx_v_B_Na3 + __pyx_v_k3_C_Na2), (__pyx_v_C_Na1 + __pyx_v_k3_C_Na2));
```

```
 871:                                    C_Na2 + k3_C_Na2,  B_Na12 + k3_C_Na2, a_Na12 + k3_C_Na2,
```

```
 872:                                     a_Na3 + k3_C_Na2, IC_Na2 + k3_C_Na2, B_Na3 + k3_C_Na2 ,
```

```
 873:                                     C_Na1 + k3_C_Na2)
```

```
 874:
```

```
 875:
```

```
 876:                 dC_Na2 = (1./6)*(k1_C_Na2 + 2*k2_C_Na2 + 2*k3_C_Na2 + k4_C_Na2)
```

```
      __pyx_v_dC_Na2 = ((1. / 6.0) * (((__pyx_v_k1_C_Na2 + (2.0 * __pyx_v_k2_C_Na2)) + (2.0 * __pyx_v_k3_C_Na2)) + __pyx_v_k4_C_Na2));
```

```
 877:
```

```
 878:
```

```
 879:
```

```
 880:
```

```
 881:                 k1_C_Na1 = self.f_C_Na1(a_Na12, C_Na2, B_Na12, C_Na1, B_Na13,
```

```
      __pyx_v_k1_C_Na1 = ((struct __pyx_vtabstruct_5model_Bondarenko *)__pyx_v_self->__pyx_vtab)->f_C_Na1(__pyx_v_self, __pyx_v_a_Na12, __pyx_v_C_Na2, __pyx_v_B_Na12, __pyx_v_C_Na1, __pyx_v_B_Na13, __pyx_v_O_Na, __pyx_v_a_Na13, __pyx_v_a_Na3, __pyx_v_IF_Na, __pyx_v_B_Na3);
```

```
 882:                                    O_Na, a_Na13, a_Na3, IF_Na, B_Na3)
```

```
 883:
```

```
 884:                 k2_C_Na1 = self.f_C_Na1(a_Na12 + (1./2)*k1_C_Na1, C_Na2 + (1./2)*k1_C_Na1, B_Na12 + (1./2)*k1_C_Na1,
```

```
      __pyx_v_k2_C_Na1 = ((struct __pyx_vtabstruct_5model_Bondarenko *)__pyx_v_self->__pyx_vtab)->f_C_Na1(__pyx_v_self, (__pyx_v_a_Na12 + ((1. / 2.0) * __pyx_v_k1_C_Na1)), (__pyx_v_C_Na2 + ((1. / 2.0) * __pyx_v_k1_C_Na1)), (__pyx_v_B_Na12 + ((1. / 2.0) * __pyx_v_k1_C_Na1)), (__pyx_v_C_Na1 + ((1. / 2.0) * __pyx_v_k1_C_Na1)), (__pyx_v_B_Na13 + ((1. / 2.0) * __pyx_v_k1_C_Na1)), (__pyx_v_O_Na + ((1. / 2.0) * __pyx_v_k1_C_Na1)), (__pyx_v_a_Na13 + ((1. / 2.0) * __pyx_v_k1_C_Na1)), (__pyx_v_a_Na3 + ((1. / 2.0) * __pyx_v_k1_C_Na1)), (__pyx_v_IF_Na + ((1. / 2.0) * __pyx_v_k1_C_Na1)), (__pyx_v_B_Na3 + ((1. / 2.0) * __pyx_v_k1_C_Na1)));
```

```
 885:                                    C_Na1 + (1./2)*k1_C_Na1, B_Na13 + (1./2)*k1_C_Na1,O_Na + (1./2)*k1_C_Na1,
```

```
 886:                                     a_Na13 + (1./2)*k1_C_Na1, a_Na3 + (1./2)*k1_C_Na1, IF_Na + (1./2)*k1_C_Na1,
```

```
 887:                                     B_Na3 + (1./2)*k1_C_Na1)
```

```
 888:
```

```
 889:                 k3_C_Na1 = self.f_C_Na1(a_Na12 + (1./2)*k2_C_Na1 , C_Na2 + (1./2)*k2_C_Na1 , B_Na12 + (1./2)*k2_C_Na1 ,
```

```
      __pyx_v_k3_C_Na1 = ((struct __pyx_vtabstruct_5model_Bondarenko *)__pyx_v_self->__pyx_vtab)->f_C_Na1(__pyx_v_self, (__pyx_v_a_Na12 + ((1. / 2.0) * __pyx_v_k2_C_Na1)), (__pyx_v_C_Na2 + ((1. / 2.0) * __pyx_v_k2_C_Na1)), (__pyx_v_B_Na12 + ((1. / 2.0) * __pyx_v_k2_C_Na1)), (__pyx_v_C_Na1 + ((1. / 2.0) * __pyx_v_k2_C_Na1)), (__pyx_v_B_Na13 + ((1. / 2.0) * __pyx_v_k2_C_Na1)), (__pyx_v_O_Na + ((1. / 2.0) * __pyx_v_k2_C_Na1)), (__pyx_v_a_Na13 + ((1. / 2.0) * __pyx_v_k2_C_Na1)), (__pyx_v_a_Na3 + ((1. / 2.0) * __pyx_v_k2_C_Na1)), (__pyx_v_IF_Na + ((1. / 2.0) * __pyx_v_k2_C_Na1)), (__pyx_v_B_Na3 + ((1. / 2.0) * __pyx_v_k2_C_Na1)));
```

```
 890:                                    C_Na1 + (1./2)*k2_C_Na1 , B_Na13 + (1./2)*k2_C_Na1 , O_Na + (1./2)*k2_C_Na1 ,
```

```
 891:                                     a_Na13 + (1./2)*k2_C_Na1 , a_Na3 + (1./2)*k2_C_Na1 , IF_Na + (1./2)*k2_C_Na1 ,
```

```
 892:                                      B_Na3 + (1./2)*k2_C_Na1 )
```

```
 893:
```

```
 894:                 k4_C_Na1 = self.f_C_Na1(a_Na12 + k3_C_Na1 , C_Na2 + k3_C_Na1 , B_Na12 + k3_C_Na1 ,
```

```
      __pyx_v_k4_C_Na1 = ((struct __pyx_vtabstruct_5model_Bondarenko *)__pyx_v_self->__pyx_vtab)->f_C_Na1(__pyx_v_self, (__pyx_v_a_Na12 + __pyx_v_k3_C_Na1), (__pyx_v_C_Na2 + __pyx_v_k3_C_Na1), (__pyx_v_B_Na12 + __pyx_v_k3_C_Na1), (__pyx_v_C_Na1 + __pyx_v_k3_C_Na1), (__pyx_v_B_Na13 + __pyx_v_k3_C_Na1), (__pyx_v_O_Na + __pyx_v_k3_C_Na1), (__pyx_v_a_Na13 + __pyx_v_k3_C_Na1), (__pyx_v_a_Na3 + __pyx_v_k3_C_Na1), (__pyx_v_IF_Na + __pyx_v_k3_C_Na1), (__pyx_v_B_Na3 + __pyx_v_k3_C_Na1));
```

```
 895:                                      C_Na1 + k3_C_Na1 , B_Na13 + k3_C_Na1 , O_Na + k3_C_Na1 ,
```

```
 896:                                     a_Na13 + k3_C_Na1 , a_Na3 + k3_C_Na1 , IF_Na + k3_C_Na1 ,
```

```
 897:                                     B_Na3 + k3_C_Na1 )
```

```
 898:
```

```
 899:
```

```
 900:                 dC_Na1 = (1./6)*(k1_C_Na1 + 2*k2_C_Na1 + 2*k3_C_Na1 + k4_C_Na1)
```

```
      __pyx_v_dC_Na1 = ((1. / 6.0) * (((__pyx_v_k1_C_Na1 + (2.0 * __pyx_v_k2_C_Na1)) + (2.0 * __pyx_v_k3_C_Na1)) + __pyx_v_k4_C_Na1));
```

```
 901:
```

```
 902:
```

```
 903:
```

```
 904:
```

```
 905:                 k1_O_Na = self.f_O_Na(a_Na13, C_Na1, B_Na13, O_Na, B_Na2, IF_Na, a_Na2)
```

```
      __pyx_v_k1_O_Na = ((struct __pyx_vtabstruct_5model_Bondarenko *)__pyx_v_self->__pyx_vtab)->f_O_Na(__pyx_v_self, __pyx_v_a_Na13, __pyx_v_C_Na1, __pyx_v_B_Na13, __pyx_v_O_Na, __pyx_v_B_Na2, __pyx_v_IF_Na, __pyx_v_a_Na2);
```

```
 906:
```

```
 907:                 k2_O_Na = self.f_O_Na(a_Na13 + (1./2)*k1_O_Na, C_Na1 + (1./2)*k1_O_Na, B_Na13 + (1./2)*k1_O_Na,
```

```
      __pyx_v_k2_O_Na = ((struct __pyx_vtabstruct_5model_Bondarenko *)__pyx_v_self->__pyx_vtab)->f_O_Na(__pyx_v_self, (__pyx_v_a_Na13 + ((1. / 2.0) * __pyx_v_k1_O_Na)), (__pyx_v_C_Na1 + ((1. / 2.0) * __pyx_v_k1_O_Na)), (__pyx_v_B_Na13 + ((1. / 2.0) * __pyx_v_k1_O_Na)), (__pyx_v_O_Na + ((1. / 2.0) * __pyx_v_k1_O_Na)), (__pyx_v_B_Na2 + ((1. / 2.0) * __pyx_v_k1_O_Na)), (__pyx_v_IF_Na + ((1. / 2.0) * __pyx_v_k1_O_Na)), (__pyx_v_a_Na2 + ((1. / 2.0) * __pyx_v_k1_O_Na)));
```

```
 908:                                O_Na + (1./2)*k1_O_Na, B_Na2 + (1./2)*k1_O_Na, IF_Na + (1./2)*k1_O_Na,
```

```
 909:                                a_Na2 + (1./2)*k1_O_Na)
```

```
 910:
```

```
 911:                 k3_O_Na = self.f_O_Na(a_Na13 + (1./2)*k2_O_Na, C_Na1 + (1./2)*k2_O_Na, B_Na13 + (1./2)*k2_O_Na,
```

```
      __pyx_v_k3_O_Na = ((struct __pyx_vtabstruct_5model_Bondarenko *)__pyx_v_self->__pyx_vtab)->f_O_Na(__pyx_v_self, (__pyx_v_a_Na13 + ((1. / 2.0) * __pyx_v_k2_O_Na)), (__pyx_v_C_Na1 + ((1. / 2.0) * __pyx_v_k2_O_Na)), (__pyx_v_B_Na13 + ((1. / 2.0) * __pyx_v_k2_O_Na)), (__pyx_v_O_Na + ((1. / 2.0) * __pyx_v_k2_O_Na)), (__pyx_v_B_Na2 + ((1. / 2.0) * __pyx_v_k2_O_Na)), (__pyx_v_IF_Na + ((1. / 2.0) * __pyx_v_k2_O_Na)), (__pyx_v_a_Na2 + ((1. / 2.0) * __pyx_v_k2_O_Na)));
```

```
 912:                                O_Na + (1./2)*k2_O_Na, B_Na2 + (1./2)*k2_O_Na, IF_Na + (1./2)*k2_O_Na,
```

```
 913:                                a_Na2 + (1./2)*k2_O_Na)
```

```
 914:
```

```
 915:                 k4_O_Na = self.f_O_Na(a_Na13 + k3_O_Na, C_Na1 + k3_O_Na, B_Na13 + k3_O_Na, O_Na + k3_O_Na,
```

```
      __pyx_v_k4_O_Na = ((struct __pyx_vtabstruct_5model_Bondarenko *)__pyx_v_self->__pyx_vtab)->f_O_Na(__pyx_v_self, (__pyx_v_a_Na13 + __pyx_v_k3_O_Na), (__pyx_v_C_Na1 + __pyx_v_k3_O_Na), (__pyx_v_B_Na13 + __pyx_v_k3_O_Na), (__pyx_v_O_Na + __pyx_v_k3_O_Na), (__pyx_v_B_Na2 + __pyx_v_k3_O_Na), (__pyx_v_IF_Na + __pyx_v_k3_O_Na), (__pyx_v_a_Na2 + __pyx_v_k3_O_Na));
```

```
 916:                                B_Na2 + k3_O_Na, IF_Na + k3_O_Na, a_Na2 + k3_O_Na)
```

```
 917:
```

```
 918:
```

```
 919:                 dO_Na = (1./6)*(k1_O_Na + 2*k2_O_Na + 2*k3_O_Na + k4_O_Na)
```

```
      __pyx_v_dO_Na = ((1. / 6.0) * (((__pyx_v_k1_O_Na + (2.0 * __pyx_v_k2_O_Na)) + (2.0 * __pyx_v_k3_O_Na)) + __pyx_v_k4_O_Na));
```

```
 920:
```

```
 921:
```

```
 922:
```

```
 923:
```

```
 924:                 k1_I_FNa = self.f_IF_Na(a_Na2, O_Na, B_Na2, IF_Na, B_Na3, C_Na1, a_Na3,
```

```
      __pyx_v_k1_I_FNa = ((struct __pyx_vtabstruct_5model_Bondarenko *)__pyx_v_self->__pyx_vtab)->f_IF_Na(__pyx_v_self, __pyx_v_a_Na2, __pyx_v_O_Na, __pyx_v_B_Na2, __pyx_v_IF_Na, __pyx_v_B_Na3, __pyx_v_C_Na1, __pyx_v_a_Na3, __pyx_v_B_Na4, __pyx_v_I1_Na, __pyx_v_a_Na4, __pyx_v_a_Na12, __pyx_v_IC_Na2, __pyx_v_B_Na12);
```

```
 925:                                  B_Na4, I1_Na, a_Na4, a_Na12, IC_Na2, B_Na12)
```

```
 926:
```

```
 927:                 k2_I_FNa = self.f_IF_Na(a_Na2 + (1./2)*k1_I_FNa, O_Na+ (1./2)*k1_I_FNa, B_Na2 + (1./2)*k1_I_FNa,
```

```
      __pyx_v_k2_I_FNa = ((struct __pyx_vtabstruct_5model_Bondarenko *)__pyx_v_self->__pyx_vtab)->f_IF_Na(__pyx_v_self, (__pyx_v_a_Na2 + ((1. / 2.0) * __pyx_v_k1_I_FNa)), (__pyx_v_O_Na + ((1. / 2.0) * __pyx_v_k1_I_FNa)), (__pyx_v_B_Na2 + ((1. / 2.0) * __pyx_v_k1_I_FNa)), (__pyx_v_IF_Na + ((1. / 2.0) * __pyx_v_k1_I_FNa)), (__pyx_v_B_Na3 + ((1. / 2.0) * __pyx_v_k1_I_FNa)), (__pyx_v_C_Na1 + ((1. / 2.0) * __pyx_v_k1_I_FNa)), (__pyx_v_a_Na3 + ((1. / 2.0) * __pyx_v_k1_I_FNa)), (__pyx_v_B_Na4 + ((1. / 2.0) * __pyx_v_k1_I_FNa)), (__pyx_v_I1_Na + ((1. / 2.0) * __pyx_v_k1_I_FNa)), (__pyx_v_a_Na4 + ((1. / 2.0) * __pyx_v_k1_I_FNa)), (__pyx_v_a_Na12 + ((1. / 2.0) * __pyx_v_k1_I_FNa)), (__pyx_v_IC_Na2 + ((1. / 2.0) * __pyx_v_k1_I_FNa)), (__pyx_v_B_Na12 + ((1. / 2.0) * __pyx_v_k1_I_FNa)));
```

```
 928:                                  IF_Na + (1./2)*k1_I_FNa, B_Na3 + (1./2)*k1_I_FNa, C_Na1 + (1./2)*k1_I_FNa,
```

```
 929:                                  a_Na3 + (1./2)*k1_I_FNa, B_Na4 + (1./2)*k1_I_FNa, I1_Na + (1./2)*k1_I_FNa,
```

```
 930:                                  a_Na4 + (1./2)*k1_I_FNa, a_Na12 + (1./2)*k1_I_FNa, IC_Na2 + (1./2)*k1_I_FNa,
```

```
 931:                                  B_Na12 + (1./2)*k1_I_FNa)
```

```
 932:
```

```
 933:                 k3_I_FNa = self.f_IF_Na(a_Na2 + (1./2)*k2_I_FNa, O_Na+ (1./2)*k2_I_FNa, B_Na2 + (1./2)*k2_I_FNa,
```

```
      __pyx_v_k3_I_FNa = ((struct __pyx_vtabstruct_5model_Bondarenko *)__pyx_v_self->__pyx_vtab)->f_IF_Na(__pyx_v_self, (__pyx_v_a_Na2 + ((1. / 2.0) * __pyx_v_k2_I_FNa)), (__pyx_v_O_Na + ((1. / 2.0) * __pyx_v_k2_I_FNa)), (__pyx_v_B_Na2 + ((1. / 2.0) * __pyx_v_k2_I_FNa)), (__pyx_v_IF_Na + ((1. / 2.0) * __pyx_v_k2_I_FNa)), (__pyx_v_B_Na3 + ((1. / 2.0) * __pyx_v_k2_I_FNa)), (__pyx_v_C_Na1 + ((1. / 2.0) * __pyx_v_k2_I_FNa)), (__pyx_v_a_Na3 + ((1. / 2.0) * __pyx_v_k2_I_FNa)), (__pyx_v_B_Na4 + ((1. / 2.0) * __pyx_v_k2_I_FNa)), (__pyx_v_I1_Na + ((1. / 2.0) * __pyx_v_k2_I_FNa)), (__pyx_v_a_Na4 + ((1. / 2.0) * __pyx_v_k2_I_FNa)), (__pyx_v_a_Na12 + ((1. / 2.0) * __pyx_v_k2_I_FNa)), (__pyx_v_IC_Na2 + ((1. / 2.0) * __pyx_v_k2_I_FNa)), (__pyx_v_B_Na12 + ((1. / 2.0) * __pyx_v_k2_I_FNa)));
```

```
 934:                                  IF_Na + (1./2)*k2_I_FNa, B_Na3 + (1./2)*k2_I_FNa, C_Na1 + (1./2)*k2_I_FNa,
```

```
 935:                                  a_Na3 + (1./2)*k2_I_FNa, B_Na4 + (1./2)*k2_I_FNa, I1_Na + (1./2)*k2_I_FNa,
```

```
 936:                                  a_Na4 + (1./2)*k2_I_FNa, a_Na12 + (1./2)*k2_I_FNa, IC_Na2 + (1./2)*k2_I_FNa,
```

```
 937:                                  B_Na12 + (1./2)*k2_I_FNa)
```

```
 938:
```

```
 939:                 k4_I_FNa = self.f_IF_Na(a_Na2 + k3_I_FNa, O_Na+ k3_I_FNa, B_Na2 + k3_I_FNa,
```

```
      __pyx_v_k4_I_FNa = ((struct __pyx_vtabstruct_5model_Bondarenko *)__pyx_v_self->__pyx_vtab)->f_IF_Na(__pyx_v_self, (__pyx_v_a_Na2 + __pyx_v_k3_I_FNa), (__pyx_v_O_Na + __pyx_v_k3_I_FNa), (__pyx_v_B_Na2 + __pyx_v_k3_I_FNa), (__pyx_v_IF_Na + __pyx_v_k3_I_FNa), (__pyx_v_B_Na3 + __pyx_v_k3_I_FNa), (__pyx_v_C_Na1 + __pyx_v_k3_I_FNa), (__pyx_v_a_Na3 + __pyx_v_k3_I_FNa), (__pyx_v_B_Na4 + __pyx_v_k3_I_FNa), (__pyx_v_I1_Na + __pyx_v_k3_I_FNa), (__pyx_v_a_Na4 + __pyx_v_k3_I_FNa), (__pyx_v_a_Na12 + __pyx_v_k3_I_FNa), (__pyx_v_IC_Na2 + __pyx_v_k3_I_FNa), (__pyx_v_B_Na12 + __pyx_v_k3_I_FNa));
```

```
 940:                                  IF_Na + k3_I_FNa, B_Na3 + k3_I_FNa, C_Na1 + k3_I_FNa,
```

```
 941:                                  a_Na3 + k3_I_FNa, B_Na4 + k3_I_FNa, I1_Na + k3_I_FNa,
```

```
 942:                                  a_Na4 + k3_I_FNa, a_Na12 + k3_I_FNa, IC_Na2 + k3_I_FNa,
```

```
 943:                                  B_Na12 + k3_I_FNa)
```

```
 944:
```

```
 945:                 dIF_Na = (1./6)*(k1_I_FNa + 2*k2_I_FNa + 2*k3_I_FNa + k4_I_FNa)
```

```
      __pyx_v_dIF_Na = ((1. / 6.0) * (((__pyx_v_k1_I_FNa + (2.0 * __pyx_v_k2_I_FNa)) + (2.0 * __pyx_v_k3_I_FNa)) + __pyx_v_k4_I_FNa));
```

```
 946:
```

```
 947:
```

```
 948:
```

```
 949:
```

```
 950:                 k1_I1_Na = self.f_I1_Na(a_Na4, IF_Na, B_Na4, I1_Na, B_Na5, I2_Na, a_Na5)
```

```
      __pyx_v_k1_I1_Na = ((struct __pyx_vtabstruct_5model_Bondarenko *)__pyx_v_self->__pyx_vtab)->f_I1_Na(__pyx_v_self, __pyx_v_a_Na4, __pyx_v_IF_Na, __pyx_v_B_Na4, __pyx_v_I1_Na, __pyx_v_B_Na5, __pyx_v_I2_Na, __pyx_v_a_Na5);
```

```
 951:
```

```
 952:                 k2_I1_Na = self.f_I1_Na(a_Na4 + (1./2)*k1_I1_Na, IF_Na + (1./2)*k1_I1_Na, B_Na4 + (1./2)*k1_I1_Na,
```

```
      __pyx_v_k2_I1_Na = ((struct __pyx_vtabstruct_5model_Bondarenko *)__pyx_v_self->__pyx_vtab)->f_I1_Na(__pyx_v_self, (__pyx_v_a_Na4 + ((1. / 2.0) * __pyx_v_k1_I1_Na)), (__pyx_v_IF_Na + ((1. / 2.0) * __pyx_v_k1_I1_Na)), (__pyx_v_B_Na4 + ((1. / 2.0) * __pyx_v_k1_I1_Na)), (__pyx_v_I1_Na + ((1. / 2.0) * __pyx_v_k1_I1_Na)), (__pyx_v_B_Na5 + ((1. / 2.0) * __pyx_v_k1_I1_Na)), (__pyx_v_I2_Na + ((1. / 2.0) * __pyx_v_k1_I1_Na)), (__pyx_v_a_Na5 + ((1. / 2.0) * __pyx_v_k1_I1_Na)));
```

```
 953:                                  I1_Na + (1./2)*k1_I1_Na, B_Na5 + (1./2)*k1_I1_Na, I2_Na + (1./2)*k1_I1_Na,
```

```
 954:                                  a_Na5 + (1./2)*k1_I1_Na)
```

```
 955:
```

```
 956:                 k3_I1_Na = self.f_I1_Na(a_Na4 + (1./2)*k2_I1_Na, IF_Na + (1./2)*k2_I1_Na, B_Na4 + (1./2)*k2_I1_Na,
```

```
      __pyx_v_k3_I1_Na = ((struct __pyx_vtabstruct_5model_Bondarenko *)__pyx_v_self->__pyx_vtab)->f_I1_Na(__pyx_v_self, (__pyx_v_a_Na4 + ((1. / 2.0) * __pyx_v_k2_I1_Na)), (__pyx_v_IF_Na + ((1. / 2.0) * __pyx_v_k2_I1_Na)), (__pyx_v_B_Na4 + ((1. / 2.0) * __pyx_v_k2_I1_Na)), (__pyx_v_I1_Na + ((1. / 2.0) * __pyx_v_k2_I1_Na)), (__pyx_v_B_Na5 + ((1. / 2.0) * __pyx_v_k2_I1_Na)), (__pyx_v_I2_Na + ((1. / 2.0) * __pyx_v_k2_I1_Na)), (__pyx_v_a_Na5 + ((1. / 2.0) * __pyx_v_k2_I1_Na)));
```

```
 957:                                  I1_Na + (1./2)*k2_I1_Na, B_Na5 + (1./2)*k2_I1_Na, I2_Na + (1./2)*k2_I1_Na,
```

```
 958:                                  a_Na5 + (1./2)*k2_I1_Na)
```

```
 959:
```

```
 960:                 k4_I1_Na = self.f_I1_Na(a_Na4 + k3_I1_Na, IF_Na + k3_I1_Na, B_Na4 + k3_I1_Na,
```

```
      __pyx_v_k4_I1_Na = ((struct __pyx_vtabstruct_5model_Bondarenko *)__pyx_v_self->__pyx_vtab)->f_I1_Na(__pyx_v_self, (__pyx_v_a_Na4 + __pyx_v_k3_I1_Na), (__pyx_v_IF_Na + __pyx_v_k3_I1_Na), (__pyx_v_B_Na4 + __pyx_v_k3_I1_Na), (__pyx_v_I1_Na + __pyx_v_k3_I1_Na), (__pyx_v_B_Na5 + __pyx_v_k3_I1_Na), (__pyx_v_I2_Na + __pyx_v_k3_I1_Na), (__pyx_v_a_Na5 + __pyx_v_k3_I1_Na));
```

```
 961:                                  I1_Na + k3_I1_Na, B_Na5 + k3_I1_Na, I2_Na + k3_I1_Na,
```

```
 962:                                  a_Na5 + k3_I1_Na)
```

```
 963:
```

```
 964:                 dI1_Na = (1./6)*(k1_I1_Na + 2*k2_I1_Na + 2*k3_I1_Na + k4_I1_Na)
```

```
      __pyx_v_dI1_Na = ((1. / 6.0) * (((__pyx_v_k1_I1_Na + (2.0 * __pyx_v_k2_I1_Na)) + (2.0 * __pyx_v_k3_I1_Na)) + __pyx_v_k4_I1_Na));
```

```
 965:
```

```
 966:
```

```
 967:
```

```
 968:
```

```
 969:                 k1_I2_Na = self.f_I2_Na (a_Na5, I1_Na, B_Na5, I2_Na)
```

```
      __pyx_v_k1_I2_Na = ((struct __pyx_vtabstruct_5model_Bondarenko *)__pyx_v_self->__pyx_vtab)->f_I2_Na(__pyx_v_self, __pyx_v_a_Na5, __pyx_v_I1_Na, __pyx_v_B_Na5, __pyx_v_I2_Na);
```

```
 970:
```

```
 971:                 k2_I2_Na = self.f_I2_Na (a_Na5 + (1./2)*k1_I2_Na, I1_Na + (1./2)*k1_I2_Na,
```

```
      __pyx_v_k2_I2_Na = ((struct __pyx_vtabstruct_5model_Bondarenko *)__pyx_v_self->__pyx_vtab)->f_I2_Na(__pyx_v_self, (__pyx_v_a_Na5 + ((1. / 2.0) * __pyx_v_k1_I2_Na)), (__pyx_v_I1_Na + ((1. / 2.0) * __pyx_v_k1_I2_Na)), (__pyx_v_B_Na5 + ((1. / 2.0) * __pyx_v_k1_I2_Na)), (__pyx_v_I2_Na + ((1. / 2.0) * __pyx_v_k1_I2_Na)));
```

```
 972:                                   B_Na5 + (1./2)*k1_I2_Na, I2_Na + (1./2)*k1_I2_Na)
```

```
 973:
```

```
 974:                 k3_I2_Na = self.f_I2_Na (a_Na5 + (1./2)*k2_I2_Na, I1_Na + (1./2)*k2_I2_Na,
```

```
      __pyx_v_k3_I2_Na = ((struct __pyx_vtabstruct_5model_Bondarenko *)__pyx_v_self->__pyx_vtab)->f_I2_Na(__pyx_v_self, (__pyx_v_a_Na5 + ((1. / 2.0) * __pyx_v_k2_I2_Na)), (__pyx_v_I1_Na + ((1. / 2.0) * __pyx_v_k2_I2_Na)), (__pyx_v_B_Na5 + ((1. / 2.0) * __pyx_v_k2_I2_Na)), (__pyx_v_I2_Na + ((1. / 2.0) * __pyx_v_k2_I2_Na)));
```

```
 975:                                   B_Na5 + (1./2)*k2_I2_Na, I2_Na + (1./2)*k2_I2_Na)
```

```
 976:
```

```
 977:                 k4_I2_Na = self.f_I2_Na (a_Na5 + k3_I2_Na, I1_Na + k3_I2_Na,
```

```
      __pyx_v_k4_I2_Na = ((struct __pyx_vtabstruct_5model_Bondarenko *)__pyx_v_self->__pyx_vtab)->f_I2_Na(__pyx_v_self, (__pyx_v_a_Na5 + __pyx_v_k3_I2_Na), (__pyx_v_I1_Na + __pyx_v_k3_I2_Na), (__pyx_v_B_Na5 + __pyx_v_k3_I2_Na), (__pyx_v_I2_Na + __pyx_v_k3_I2_Na));
```

```
 978:                                   B_Na5 + k3_I2_Na, I2_Na + k3_I2_Na)
```

```
 979:
```

```
 980:
```

```
 981:                 dI2_Na = (1./6)*(k1_I2_Na + 2*k2_I2_Na + 2*k3_I2_Na + k4_I2_Na)
```

```
      __pyx_v_dI2_Na = ((1. / 6.0) * (((__pyx_v_k1_I2_Na + (2.0 * __pyx_v_k2_I2_Na)) + (2.0 * __pyx_v_k3_I2_Na)) + __pyx_v_k4_I2_Na));
```

```
 982:
```

```
 983:
```

```
 984:
```

```
 985:
```

```
 986:                 k1_IC_Na2 = self.f_IC_Na2 (a_Na11, IC_Na3, B_Na11, IC_Na2, B_Na12, IF_Na,
```

```
      __pyx_v_k1_IC_Na2 = ((struct __pyx_vtabstruct_5model_Bondarenko *)__pyx_v_self->__pyx_vtab)->f_IC_Na2(__pyx_v_self, __pyx_v_a_Na11, __pyx_v_IC_Na3, __pyx_v_B_Na11, __pyx_v_IC_Na2, __pyx_v_B_Na12, __pyx_v_IF_Na, __pyx_v_a_Na12, __pyx_v_B_Na3, __pyx_v_C_Na2, __pyx_v_a_Na3);
```

```
 987:                                     a_Na12, B_Na3, C_Na2, a_Na3)
```

```
 988:
```

```
 989:                 k2_IC_Na2 = self.f_IC_Na2 (a_Na11 + (1./2)*k1_IC_Na2, IC_Na3 + (1./2)*k1_IC_Na2, B_Na11 + (1./2)*k1_IC_Na2,
```

```
      __pyx_v_k2_IC_Na2 = ((struct __pyx_vtabstruct_5model_Bondarenko *)__pyx_v_self->__pyx_vtab)->f_IC_Na2(__pyx_v_self, (__pyx_v_a_Na11 + ((1. / 2.0) * __pyx_v_k1_IC_Na2)), (__pyx_v_IC_Na3 + ((1. / 2.0) * __pyx_v_k1_IC_Na2)), (__pyx_v_B_Na11 + ((1. / 2.0) * __pyx_v_k1_IC_Na2)), (__pyx_v_IC_Na2 + ((1. / 2.0) * __pyx_v_k1_IC_Na2)), (__pyx_v_B_Na12 + ((1. / 2.0) * __pyx_v_k1_IC_Na2)), (__pyx_v_IF_Na + ((1. / 2.0) * __pyx_v_k1_IC_Na2)), (__pyx_v_a_Na12 + ((1. / 2.0) * __pyx_v_k1_IC_Na2)), (__pyx_v_B_Na3 + ((1. / 2.0) * __pyx_v_k1_IC_Na2)), (__pyx_v_C_Na2 + ((1. / 2.0) * __pyx_v_k1_IC_Na2)), (__pyx_v_a_Na3 + ((1. / 2.0) * __pyx_v_k1_IC_Na2)));
```

```
 990:                                     IC_Na2 + (1./2)*k1_IC_Na2, B_Na12 + (1./2)*k1_IC_Na2, IF_Na + (1./2)*k1_IC_Na2,
```

```
 991:                                     a_Na12 + (1./2)*k1_IC_Na2, B_Na3 + (1./2)*k1_IC_Na2, C_Na2 + (1./2)*k1_IC_Na2,
```

```
 992:                                     a_Na3 + (1./2)*k1_IC_Na2 )
```

```
 993:
```

```
 994:                 k3_IC_Na2 = self.f_IC_Na2 (a_Na11 + (1./2)*k2_IC_Na2, IC_Na3 + (1./2)*k2_IC_Na2, B_Na11 + (1./2)*k2_IC_Na2,
```

```
      __pyx_v_k3_IC_Na2 = ((struct __pyx_vtabstruct_5model_Bondarenko *)__pyx_v_self->__pyx_vtab)->f_IC_Na2(__pyx_v_self, (__pyx_v_a_Na11 + ((1. / 2.0) * __pyx_v_k2_IC_Na2)), (__pyx_v_IC_Na3 + ((1. / 2.0) * __pyx_v_k2_IC_Na2)), (__pyx_v_B_Na11 + ((1. / 2.0) * __pyx_v_k2_IC_Na2)), (__pyx_v_IC_Na2 + ((1. / 2.0) * __pyx_v_k2_IC_Na2)), (__pyx_v_B_Na12 + ((1. / 2.0) * __pyx_v_k2_IC_Na2)), (__pyx_v_IF_Na + ((1. / 2.0) * __pyx_v_k2_IC_Na2)), (__pyx_v_a_Na12 + ((1. / 2.0) * __pyx_v_k2_IC_Na2)), (__pyx_v_B_Na3 + ((1. / 2.0) * __pyx_v_k2_IC_Na2)), (__pyx_v_C_Na2 + ((1. / 2.0) * __pyx_v_k2_IC_Na2)), (__pyx_v_a_Na3 + ((1. / 2.0) * __pyx_v_k2_IC_Na2)));
```

```
 995:                                     IC_Na2 + (1./2)*k2_IC_Na2, B_Na12 + (1./2)*k2_IC_Na2, IF_Na + (1./2)*k2_IC_Na2,
```

```
 996:                                     a_Na12 + (1./2)*k2_IC_Na2, B_Na3 + (1./2)*k2_IC_Na2, C_Na2 + (1./2)*k2_IC_Na2,
```

```
 997:                                     a_Na3 + (1./2)*k2_IC_Na2 )
```

```
 998:
```

```
 999:                 k4_IC_Na2 = self.f_IC_Na2 (a_Na11 + k3_IC_Na2, IC_Na3 + k3_IC_Na2, B_Na11 + k3_IC_Na2,
```

```
      __pyx_v_k4_IC_Na2 = ((struct __pyx_vtabstruct_5model_Bondarenko *)__pyx_v_self->__pyx_vtab)->f_IC_Na2(__pyx_v_self, (__pyx_v_a_Na11 + __pyx_v_k3_IC_Na2), (__pyx_v_IC_Na3 + __pyx_v_k3_IC_Na2), (__pyx_v_B_Na11 + __pyx_v_k3_IC_Na2), (__pyx_v_IC_Na2 + __pyx_v_k3_IC_Na2), (__pyx_v_B_Na12 + __pyx_v_k3_IC_Na2), (__pyx_v_IF_Na + __pyx_v_k3_IC_Na2), (__pyx_v_a_Na12 + __pyx_v_k3_IC_Na2), (__pyx_v_B_Na3 + __pyx_v_k3_IC_Na2), (__pyx_v_C_Na2 + __pyx_v_k3_IC_Na2), (__pyx_v_a_Na3 + __pyx_v_k3_IC_Na2));
```

```
 1000:                                     IC_Na2 + k3_IC_Na2, B_Na12 + k3_IC_Na2, IF_Na + k3_IC_Na2,
```

```
 1001:                                     a_Na12 + k3_IC_Na2, B_Na3 + k3_IC_Na2, C_Na2 + k3_IC_Na2,
```

```
 1002:                                     a_Na3 + k3_IC_Na2 )
```

```
 1003:
```

```
 1004:                 dIC_Na2 = (1./6)*(k1_IC_Na2 + 2*k2_IC_Na2 + 2*k3_IC_Na2 + k4_IC_Na2)
```

```
      __pyx_v_dIC_Na2 = ((1. / 6.0) * (((__pyx_v_k1_IC_Na2 + (2.0 * __pyx_v_k2_IC_Na2)) + (2.0 * __pyx_v_k3_IC_Na2)) + __pyx_v_k4_IC_Na2));
```

```
 1005:
```

```
 1006:
```

```
 1007:
```

```
 1008:
```

```
 1009:                 k1_IC_Na3 = self.f_IC_Na3(B_Na11, IC_Na2, a_Na11, IC_Na3, B_Na3, C_Na3, a_Na3)
```

```
      __pyx_v_k1_IC_Na3 = ((struct __pyx_vtabstruct_5model_Bondarenko *)__pyx_v_self->__pyx_vtab)->f_IC_Na3(__pyx_v_self, __pyx_v_B_Na11, __pyx_v_IC_Na2, __pyx_v_a_Na11, __pyx_v_IC_Na3, __pyx_v_B_Na3, __pyx_v_C_Na3, __pyx_v_a_Na3);
```

```
 1010:
```

```
 1011:                 k2_IC_Na3 = self.f_IC_Na3(B_Na11 + (1./2)*k1_IC_Na3, IC_Na2 + (1./2)*k1_IC_Na3, a_Na11 + (1./2)*k1_IC_Na3,
```

```
      __pyx_v_k2_IC_Na3 = ((struct __pyx_vtabstruct_5model_Bondarenko *)__pyx_v_self->__pyx_vtab)->f_IC_Na3(__pyx_v_self, (__pyx_v_B_Na11 + ((1. / 2.0) * __pyx_v_k1_IC_Na3)), (__pyx_v_IC_Na2 + ((1. / 2.0) * __pyx_v_k1_IC_Na3)), (__pyx_v_a_Na11 + ((1. / 2.0) * __pyx_v_k1_IC_Na3)), (__pyx_v_IC_Na3 + ((1. / 2.0) * __pyx_v_k1_IC_Na3)), (__pyx_v_B_Na3 + ((1. / 2.0) * __pyx_v_k1_IC_Na3)), (__pyx_v_C_Na3 + ((1. / 2.0) * __pyx_v_k1_IC_Na3)), (__pyx_v_a_Na3 + ((1. / 2.0) * __pyx_v_k1_IC_Na3)));
```

```
 1012:                                    IC_Na3 + (1./2)*k1_IC_Na3, B_Na3 + (1./2)*k1_IC_Na3, C_Na3 + (1./2)*k1_IC_Na3,
```

```
 1013:                                    a_Na3 + (1./2)*k1_IC_Na3)
```

```
 1014:
```

```
 1015:                 k3_IC_Na3 = self.f_IC_Na3(B_Na11 + (1./2)*k2_IC_Na3, IC_Na2 + (1./2)*k2_IC_Na3, a_Na11 + (1./2)*k2_IC_Na3,
```

```
      __pyx_v_k3_IC_Na3 = ((struct __pyx_vtabstruct_5model_Bondarenko *)__pyx_v_self->__pyx_vtab)->f_IC_Na3(__pyx_v_self, (__pyx_v_B_Na11 + ((1. / 2.0) * __pyx_v_k2_IC_Na3)), (__pyx_v_IC_Na2 + ((1. / 2.0) * __pyx_v_k2_IC_Na3)), (__pyx_v_a_Na11 + ((1. / 2.0) * __pyx_v_k2_IC_Na3)), (__pyx_v_IC_Na3 + ((1. / 2.0) * __pyx_v_k2_IC_Na3)), (__pyx_v_B_Na3 + ((1. / 2.0) * __pyx_v_k2_IC_Na3)), (__pyx_v_C_Na3 + ((1. / 2.0) * __pyx_v_k2_IC_Na3)), (__pyx_v_a_Na3 + ((1. / 2.0) * __pyx_v_k2_IC_Na3)));
```

```
 1016:                                    IC_Na3 + (1./2)*k2_IC_Na3, B_Na3 + (1./2)*k2_IC_Na3, C_Na3 + (1./2)*k2_IC_Na3,
```

```
 1017:                                    a_Na3 + (1./2)*k2_IC_Na3)
```

```
 1018:
```

```
 1019:                 k4_IC_Na3 = self.f_IC_Na3(B_Na11 + k3_IC_Na3, IC_Na2 + k3_IC_Na3, a_Na11 + k3_IC_Na3,
```

```
      __pyx_v_k4_IC_Na3 = ((struct __pyx_vtabstruct_5model_Bondarenko *)__pyx_v_self->__pyx_vtab)->f_IC_Na3(__pyx_v_self, (__pyx_v_B_Na11 + __pyx_v_k3_IC_Na3), (__pyx_v_IC_Na2 + __pyx_v_k3_IC_Na3), (__pyx_v_a_Na11 + __pyx_v_k3_IC_Na3), (__pyx_v_IC_Na3 + __pyx_v_k3_IC_Na3), (__pyx_v_B_Na3 + __pyx_v_k3_IC_Na3), (__pyx_v_C_Na3 + __pyx_v_k3_IC_Na3), (__pyx_v_a_Na3 + __pyx_v_k3_IC_Na3));
```

```
 1020:                                    IC_Na3 + k3_IC_Na3, B_Na3 + k3_IC_Na3, C_Na3 + k3_IC_Na3,
```

```
 1021:                                    a_Na3 + k3_IC_Na3)
```

```
 1022:
```

```
 1023:                 dIC_Na3 = (1./6)*(k1_IC_Na3 + 2*k2_IC_Na3 + 2*k3_IC_Na3 + k4_IC_Na3)
```

```
      __pyx_v_dIC_Na3 = ((1. / 6.0) * (((__pyx_v_k1_IC_Na3 + (2.0 * __pyx_v_k2_IC_Na3)) + (2.0 * __pyx_v_k3_IC_Na3)) + __pyx_v_k4_IC_Na3));
```

```
 1024:
```

```
 1025:
```

```
 1026:
```

```
 1027:
```

```
 1028:                 a_a = 0.18064*exp(0.03577*(V + 30.))
```

```
      __pyx_v_a_a = (0.18064 * exp((0.03577 * (__pyx_v_V + 30.))));
```

```
 1029:
```

```
 1030:                 B_a = 0.3956*exp(-0.06237*(V+30.))
```

```
      __pyx_v_B_a = (0.3956 * exp((-0.06237 * (__pyx_v_V + 30.))));
```

```
 1031:
```

```
 1032:                 a_i = (0.000152*exp(-(V+13.5)/7.))/(0.067083*exp(-(V+33.5)/7.) + 1)
```

```
      __pyx_v_a_i = ((0.000152 * exp(((-(__pyx_v_V + 13.5)) / 7.))) / ((0.067083 * exp(((-(__pyx_v_V + 33.5)) / 7.))) + 1.0));
```

```
 1033:
```

```
 1034:                 B_i_Na = (0.00095*exp((V+33.5)/7.))/(0.051335*exp((V+33.5)/7.) + 1)
```

```
      __pyx_v_B_i_Na = ((0.00095 * exp(((__pyx_v_V + 33.5) / 7.))) / ((0.051335 * exp(((__pyx_v_V + 33.5) / 7.))) + 1.0));
```

```
 1035:
```

```
 1036:
```

```
 1037:
```

```
 1038:                 k1_a_tof = self.f_a_tof (a_a, a_tof, B_a)
```

```
      __pyx_v_k1_a_tof = ((struct __pyx_vtabstruct_5model_Bondarenko *)__pyx_v_self->__pyx_vtab)->f_a_tof(__pyx_v_self, __pyx_v_a_a, __pyx_v_a_tof, __pyx_v_B_a);
```

```
 1039:
```

```
 1040:                 k2_a_tof = self.f_a_tof (a_a + (1./2)*k1_a_tof, a_tof + (1./2)*k1_a_tof, B_a + (1./2)*k1_a_tof)
```

```
      __pyx_v_k2_a_tof = ((struct __pyx_vtabstruct_5model_Bondarenko *)__pyx_v_self->__pyx_vtab)->f_a_tof(__pyx_v_self, (__pyx_v_a_a + ((1. / 2.0) * __pyx_v_k1_a_tof)), (__pyx_v_a_tof + ((1. / 2.0) * __pyx_v_k1_a_tof)), (__pyx_v_B_a + ((1. / 2.0) * __pyx_v_k1_a_tof)));
```

```
 1041:
```

```
 1042:                 k3_a_tof = self.f_a_tof (a_a + (1./2)*k2_a_tof, a_tof + (1./2)*k2_a_tof, B_a + (1./2)*k2_a_tof)
```

```
      __pyx_v_k3_a_tof = ((struct __pyx_vtabstruct_5model_Bondarenko *)__pyx_v_self->__pyx_vtab)->f_a_tof(__pyx_v_self, (__pyx_v_a_a + ((1. / 2.0) * __pyx_v_k2_a_tof)), (__pyx_v_a_tof + ((1. / 2.0) * __pyx_v_k2_a_tof)), (__pyx_v_B_a + ((1. / 2.0) * __pyx_v_k2_a_tof)));
```

```
 1043:
```

```
 1044:                 k4_a_tof = self.f_a_tof (a_a + k3_a_tof, a_tof + k3_a_tof, B_a + k3_a_tof)
```

```
      __pyx_v_k4_a_tof = ((struct __pyx_vtabstruct_5model_Bondarenko *)__pyx_v_self->__pyx_vtab)->f_a_tof(__pyx_v_self, (__pyx_v_a_a + __pyx_v_k3_a_tof), (__pyx_v_a_tof + __pyx_v_k3_a_tof), (__pyx_v_B_a + __pyx_v_k3_a_tof));
```

```
 1045:
```

```
 1046:
```

```
 1047:                 da_tof = (1./6)*(k1_a_tof + 2*k2_a_tof + 2*k3_a_tof + k4_a_tof)
```

```
      __pyx_v_da_tof = ((1. / 6.0) * (((__pyx_v_k1_a_tof + (2.0 * __pyx_v_k2_a_tof)) + (2.0 * __pyx_v_k3_a_tof)) + __pyx_v_k4_a_tof));
```

```
 1048:
```

```
 1049:
```

```
 1050:
```

```
 1051:
```

```
 1052:                 k1_i_tof = self.f_i_tof(a_i, i_tof, B_i_Na)
```

```
      __pyx_v_k1_i_tof = ((struct __pyx_vtabstruct_5model_Bondarenko *)__pyx_v_self->__pyx_vtab)->f_i_tof(__pyx_v_self, __pyx_v_a_i, __pyx_v_i_tof, __pyx_v_B_i_Na);
```

```
 1053:
```

```
 1054:                 k2_i_tof = self.f_i_tof(a_i + (1./2)*k1_i_tof, i_tof+ (1./2)*k1_i_tof, B_i_Na+ (1./2)*k1_i_tof)
```

```
      __pyx_v_k2_i_tof = ((struct __pyx_vtabstruct_5model_Bondarenko *)__pyx_v_self->__pyx_vtab)->f_i_tof(__pyx_v_self, (__pyx_v_a_i + ((1. / 2.0) * __pyx_v_k1_i_tof)), (__pyx_v_i_tof + ((1. / 2.0) * __pyx_v_k1_i_tof)), (__pyx_v_B_i_Na + ((1. / 2.0) * __pyx_v_k1_i_tof)));
```

```
 1055:
```

```
 1056:                 k3_i_tof = self.f_i_tof(a_i + (1./2)*k2_i_tof, i_tof+ (1./2)*k2_i_tof, B_i_Na+ (1./2)*k2_i_tof)
```

```
      __pyx_v_k3_i_tof = ((struct __pyx_vtabstruct_5model_Bondarenko *)__pyx_v_self->__pyx_vtab)->f_i_tof(__pyx_v_self, (__pyx_v_a_i + ((1. / 2.0) * __pyx_v_k2_i_tof)), (__pyx_v_i_tof + ((1. / 2.0) * __pyx_v_k2_i_tof)), (__pyx_v_B_i_Na + ((1. / 2.0) * __pyx_v_k2_i_tof)));
```

```
 1057:
```

```
 1058:                 k4_i_tof = self.f_i_tof(a_i + k3_i_tof, i_tof+ k3_i_tof, B_i_Na + k3_i_tof)
```

```
      __pyx_v_k4_i_tof = ((struct __pyx_vtabstruct_5model_Bondarenko *)__pyx_v_self->__pyx_vtab)->f_i_tof(__pyx_v_self, (__pyx_v_a_i + __pyx_v_k3_i_tof), (__pyx_v_i_tof + __pyx_v_k3_i_tof), (__pyx_v_B_i_Na + __pyx_v_k3_i_tof));
```

```
 1059:
```

```
 1060:
```

```
 1061:                 di_tof = (1./6)*(k1_i_tof + 2*k2_i_tof + 2*k3_i_tof + k4_i_tof)
```

```
      __pyx_v_di_tof = ((1. / 6.0) * (((__pyx_v_k1_i_tof + (2.0 * __pyx_v_k2_i_tof)) + (2.0 * __pyx_v_k3_i_tof)) + __pyx_v_k4_i_tof));
```

```
 1062:
```

```
 1063:
```

```
 1064:
```

```
 1065:
```

```
 1066:                 t_tas = 0.493*exp(-0.0629*V) + 2.058
```

```
      __pyx_v_t_tas = ((0.493 * exp((-0.0629 * __pyx_v_V))) + 2.058);
```

```
 1067:
```

```
 1068:                 a_ss = 1./(1 + exp(-(V+22.5)/7.7))
```

```
      __pyx_v_a_ss = (1. / (1.0 + exp(((-(__pyx_v_V + 22.5)) / 7.7))));
```

```
 1069:
```

```
 1070:
```

```
 1071:
```

```
 1072:                 k1_a_tos = self.f_a_tos(a_ss, a_tos, t_tas)
```

```
      __pyx_v_k1_a_tos = ((struct __pyx_vtabstruct_5model_Bondarenko *)__pyx_v_self->__pyx_vtab)->f_a_tos(__pyx_v_self, __pyx_v_a_ss, __pyx_v_a_tos, __pyx_v_t_tas);
```

```
 1073:
```

```
 1074:                 k2_a_tos = self.f_a_tos(a_ss + (1./2)*k1_a_tos, a_tos + (1./2)*k1_a_tos, t_tas + (1./2)*k1_a_tos)
```

```
      __pyx_v_k2_a_tos = ((struct __pyx_vtabstruct_5model_Bondarenko *)__pyx_v_self->__pyx_vtab)->f_a_tos(__pyx_v_self, (__pyx_v_a_ss + ((1. / 2.0) * __pyx_v_k1_a_tos)), (__pyx_v_a_tos + ((1. / 2.0) * __pyx_v_k1_a_tos)), (__pyx_v_t_tas + ((1. / 2.0) * __pyx_v_k1_a_tos)));
```

```
 1075:
```

```
 1076:                 k3_a_tos = self.f_a_tos(a_ss + (1./2)*k2_a_tos, a_tos + (1./2)*k2_a_tos, t_tas + (1./2)*k2_a_tos)
```

```
      __pyx_v_k3_a_tos = ((struct __pyx_vtabstruct_5model_Bondarenko *)__pyx_v_self->__pyx_vtab)->f_a_tos(__pyx_v_self, (__pyx_v_a_ss + ((1. / 2.0) * __pyx_v_k2_a_tos)), (__pyx_v_a_tos + ((1. / 2.0) * __pyx_v_k2_a_tos)), (__pyx_v_t_tas + ((1. / 2.0) * __pyx_v_k2_a_tos)));
```

```
 1077:
```

```
 1078:                 k4_a_tos = self.f_a_tos(a_ss + k3_a_tos, a_tos + k3_a_tos, t_tas + k3_a_tos)
```

```
      __pyx_v_k4_a_tos = ((struct __pyx_vtabstruct_5model_Bondarenko *)__pyx_v_self->__pyx_vtab)->f_a_tos(__pyx_v_self, (__pyx_v_a_ss + __pyx_v_k3_a_tos), (__pyx_v_a_tos + __pyx_v_k3_a_tos), (__pyx_v_t_tas + __pyx_v_k3_a_tos));
```

```
 1079:
```

```
 1080:
```

```
 1081:                 da_tos = (1./6)*(k1_a_tos + 2*k2_a_tos + 2*k3_a_tos + k4_a_tos)
```

```
      __pyx_v_da_tos = ((1. / 6.0) * (((__pyx_v_k1_a_tos + (2.0 * __pyx_v_k2_a_tos)) + (2.0 * __pyx_v_k3_a_tos)) + __pyx_v_k4_a_tos));
```

```
 1082:
```

```
 1083:
```

```
 1084:
```

```
 1085:                 t_tis = 270. + (1050./(1 + exp((V+45.2)/5.7)))
```

```
      __pyx_v_t_tis = (270. + (1050. / (1.0 + exp(((__pyx_v_V + 45.2) / 5.7)))));
```

```
 1086:
```

```
 1087:                 i_ss = 1./(1 + exp((V+45.2)/5.7))
```

```
      __pyx_v_i_ss = (1. / (1.0 + exp(((__pyx_v_V + 45.2) / 5.7))));
```

```
 1088:
```

```
 1089:
```

```
 1090:
```

```
 1091:                 k1_i_tos = self.f_i_tos(i_ss, i_tos, t_tis)
```

```
      __pyx_v_k1_i_tos = ((struct __pyx_vtabstruct_5model_Bondarenko *)__pyx_v_self->__pyx_vtab)->f_i_tos(__pyx_v_self, __pyx_v_i_ss, __pyx_v_i_tos, __pyx_v_t_tis);
```

```
 1092:
```

```
 1093:                 k2_i_tos = self.f_i_tos(i_ss + (1./2)*k1_i_tos, i_tos + (1./2)*k1_i_tos, t_tis + (1./2)*k1_i_tos)
```

```
      __pyx_v_k2_i_tos = ((struct __pyx_vtabstruct_5model_Bondarenko *)__pyx_v_self->__pyx_vtab)->f_i_tos(__pyx_v_self, (__pyx_v_i_ss + ((1. / 2.0) * __pyx_v_k1_i_tos)), (__pyx_v_i_tos + ((1. / 2.0) * __pyx_v_k1_i_tos)), (__pyx_v_t_tis + ((1. / 2.0) * __pyx_v_k1_i_tos)));
```

```
 1094:
```

```
 1095:                 k3_i_tos = self.f_i_tos(i_ss + (1./2)*k2_i_tos, i_tos + (1./2)*k2_i_tos, t_tis + (1./2)*k2_i_tos)
```

```
      __pyx_v_k3_i_tos = ((struct __pyx_vtabstruct_5model_Bondarenko *)__pyx_v_self->__pyx_vtab)->f_i_tos(__pyx_v_self, (__pyx_v_i_ss + ((1. / 2.0) * __pyx_v_k2_i_tos)), (__pyx_v_i_tos + ((1. / 2.0) * __pyx_v_k2_i_tos)), (__pyx_v_t_tis + ((1. / 2.0) * __pyx_v_k2_i_tos)));
```

```
 1096:
```

```
 1097:                 k4_i_tos = self.f_i_tos(i_ss + k3_i_tos, i_tos + k3_i_tos, t_tis + k3_i_tos)
```

```
      __pyx_v_k4_i_tos = ((struct __pyx_vtabstruct_5model_Bondarenko *)__pyx_v_self->__pyx_vtab)->f_i_tos(__pyx_v_self, (__pyx_v_i_ss + __pyx_v_k3_i_tos), (__pyx_v_i_tos + __pyx_v_k3_i_tos), (__pyx_v_t_tis + __pyx_v_k3_i_tos));
```

```
 1098:
```

```
 1099:
```

```
 1100:                 di_tos = (1./6)*(k1_i_tos + 2*k2_i_tos + 2*k3_i_tos + k4_i_tos)
```

```
      __pyx_v_di_tos = ((1. / 6.0) * (((__pyx_v_k1_i_tos + (2.0 * __pyx_v_k2_i_tos)) + (2.0 * __pyx_v_k3_i_tos)) + __pyx_v_k4_i_tos));
```

```
 1101:
```

```
 1102:
```

```
 1103:
```

```
 1104:                 E_K = ((R*T)/F)*(log(K_0/K_i))
```

```
      __pyx_v_E_K = (((__pyx_v_R * __pyx_v_T) / __pyx_v_F) * log((__pyx_v_K_0 / __pyx_v_K_i)));
```

```
 1105:
```

```
 1106:                 I_Ktof = G_Ktof*(a_tof**3)*i_tof*(V - E_K)
```

```
      __pyx_v_I_Ktof = (((__pyx_v_G_Ktof * pow(__pyx_v_a_tof, 3.0)) * __pyx_v_i_tof) * (__pyx_v_V - __pyx_v_E_K));
```

```
 1107:
```

```
 1108:                 I_Ktos = G_Ktos*a_tos*i_tos*(V - E_K)
```

```
      __pyx_v_I_Ktos = (((__pyx_v_G_Ktos * __pyx_v_a_tos) * __pyx_v_i_tos) * (__pyx_v_V - __pyx_v_E_K));
```

```
 1109:
```

```
 1110:                 I_K1 = 0.3397*(K_0/(K_0 + 210.))*((V-E_K)/(1 + exp(0.0448*(V - E_K))))
```

```
      __pyx_v_I_K1 = ((0.3397 * (__pyx_v_K_0 / (__pyx_v_K_0 + 210.))) * ((__pyx_v_V - __pyx_v_E_K) / (1.0 + exp((0.0448 * (__pyx_v_V - __pyx_v_E_K))))));
```

```
 1111:
```

```
 1112:                 t_Kss = 39.3*exp(-0.0862*V) + 13.17
```

```
      __pyx_v_t_Kss = ((39.3 * exp((-0.0862 * __pyx_v_V))) + 13.17);
```

```
 1113:
```

```
 1114:
```

```
 1115:
```

```
 1116:                 k1_a_Kss = self.f_a_Kss(a_ss, a_Kss, t_Kss)
```

```
      __pyx_v_k1_a_Kss = ((struct __pyx_vtabstruct_5model_Bondarenko *)__pyx_v_self->__pyx_vtab)->f_a_Kss(__pyx_v_self, __pyx_v_a_ss, __pyx_v_a_Kss, __pyx_v_t_Kss);
```

```
 1117:
```

```
 1118:                 k2_a_Kss = self.f_a_Kss(a_ss + (1./2)*k1_a_Kss, a_Kss + (1./2)*k1_a_Kss, t_Kss + (1./2)*k1_a_Kss)
```

```
      __pyx_v_k2_a_Kss = ((struct __pyx_vtabstruct_5model_Bondarenko *)__pyx_v_self->__pyx_vtab)->f_a_Kss(__pyx_v_self, (__pyx_v_a_ss + ((1. / 2.0) * __pyx_v_k1_a_Kss)), (__pyx_v_a_Kss + ((1. / 2.0) * __pyx_v_k1_a_Kss)), (__pyx_v_t_Kss + ((1. / 2.0) * __pyx_v_k1_a_Kss)));
```

```
 1119:
```

```
 1120:                 k3_a_Kss = self.f_a_Kss(a_ss + (1./2)*k2_a_Kss, a_Kss + (1./2)*k2_a_Kss, t_Kss + (1./2)*k2_a_Kss)
```

```
      __pyx_v_k3_a_Kss = ((struct __pyx_vtabstruct_5model_Bondarenko *)__pyx_v_self->__pyx_vtab)->f_a_Kss(__pyx_v_self, (__pyx_v_a_ss + ((1. / 2.0) * __pyx_v_k2_a_Kss)), (__pyx_v_a_Kss + ((1. / 2.0) * __pyx_v_k2_a_Kss)), (__pyx_v_t_Kss + ((1. / 2.0) * __pyx_v_k2_a_Kss)));
```

```
 1121:
```

```
 1122:                 k4_a_Kss = self.f_a_Kss(a_ss + k3_a_Kss, a_Kss + k3_a_Kss, t_Kss + k3_a_Kss)
```

```
      __pyx_v_k4_a_Kss = ((struct __pyx_vtabstruct_5model_Bondarenko *)__pyx_v_self->__pyx_vtab)->f_a_Kss(__pyx_v_self, (__pyx_v_a_ss + __pyx_v_k3_a_Kss), (__pyx_v_a_Kss + __pyx_v_k3_a_Kss), (__pyx_v_t_Kss + __pyx_v_k3_a_Kss));
```

```
 1123:
```

```
 1124:
```

```
 1125:                 da_Kss = (1./6)*(k1_a_Kss + 2*k2_a_Kss + 2*k3_a_Kss + k4_a_Kss)
```

```
      __pyx_v_da_Kss = ((1. / 6.0) * (((__pyx_v_k1_a_Kss + (2.0 * __pyx_v_k2_a_Kss)) + (2.0 * __pyx_v_k3_a_Kss)) + __pyx_v_k4_a_Kss));
```

```
 1126:
```

```
 1127:
```

```
 1128:
```

```
 1129:                 I_Kss = G_Kss*a_Kss*i_Kss*(V-E_K)
```

```
      __pyx_v_I_Kss = (((__pyx_v_G_Kss * __pyx_v_a_Kss) * __pyx_v_i_Kss) * (__pyx_v_V - __pyx_v_E_K));
```

```
 1130:
```

```
 1131:                 t_aur = 0.493*exp(-0.0629*V) + 2.058
```

```
      __pyx_v_t_aur = ((0.493 * exp((-0.0629 * __pyx_v_V))) + 2.058);
```

```
 1132:
```

```
 1133:                 t_iur = 1200. - (170./(1 + exp((V+45.2)/5.7)))
```

```
      __pyx_v_t_iur = (1200. - (170. / (1.0 + exp(((__pyx_v_V + 45.2) / 5.7)))));
```

```
 1134:
```

```
 1135:                 a_n = (0.00000481333*(V + 26.5))/(1 - exp(-0.128*(V+26.5)))
```

```
      __pyx_v_a_n = ((0.00000481333 * (__pyx_v_V + 26.5)) / (1.0 - exp((-0.128 * (__pyx_v_V + 26.5)))));
```

```
 1136:
```

```
 1137:                 B_n = 0.0000953333*exp(-0.038*(V+26.5))
```

```
      __pyx_v_B_n = (0.0000953333 * exp((-0.038 * (__pyx_v_V + 26.5))));
```

```
 1138:
```

```
 1139:
```

```
 1140:
```

```
 1141:                 k1_n_Ks = self.f_n_Ks(a_n, n_Ks, B_n)
```

```
      __pyx_v_k1_n_Ks = ((struct __pyx_vtabstruct_5model_Bondarenko *)__pyx_v_self->__pyx_vtab)->f_n_Ks(__pyx_v_self, __pyx_v_a_n, __pyx_v_n_Ks, __pyx_v_B_n);
```

```
 1142:
```

```
 1143:                 k2_n_Ks = self.f_n_Ks(a_n + (1./2)*k1_n_Ks, n_Ks + (1./2)*k1_n_Ks, B_n + (1./2)*k1_n_Ks)
```

```
      __pyx_v_k2_n_Ks = ((struct __pyx_vtabstruct_5model_Bondarenko *)__pyx_v_self->__pyx_vtab)->f_n_Ks(__pyx_v_self, (__pyx_v_a_n + ((1. / 2.0) * __pyx_v_k1_n_Ks)), (__pyx_v_n_Ks + ((1. / 2.0) * __pyx_v_k1_n_Ks)), (__pyx_v_B_n + ((1. / 2.0) * __pyx_v_k1_n_Ks)));
```

```
 1144:
```

```
 1145:                 k3_n_Ks = self.f_n_Ks(a_n + (1./2)*k2_n_Ks, n_Ks + (1./2)*k2_n_Ks, B_n + (1./2)*k2_n_Ks)
```

```
      __pyx_v_k3_n_Ks = ((struct __pyx_vtabstruct_5model_Bondarenko *)__pyx_v_self->__pyx_vtab)->f_n_Ks(__pyx_v_self, (__pyx_v_a_n + ((1. / 2.0) * __pyx_v_k2_n_Ks)), (__pyx_v_n_Ks + ((1. / 2.0) * __pyx_v_k2_n_Ks)), (__pyx_v_B_n + ((1. / 2.0) * __pyx_v_k2_n_Ks)));
```

```
 1146:
```

```
 1147:                 k4_n_Ks = self.f_n_Ks(a_n + k3_n_Ks, n_Ks + k3_n_Ks, B_n + k3_n_Ks)
```

```
      __pyx_v_k4_n_Ks = ((struct __pyx_vtabstruct_5model_Bondarenko *)__pyx_v_self->__pyx_vtab)->f_n_Ks(__pyx_v_self, (__pyx_v_a_n + __pyx_v_k3_n_Ks), (__pyx_v_n_Ks + __pyx_v_k3_n_Ks), (__pyx_v_B_n + __pyx_v_k3_n_Ks));
```

```
 1148:
```

```
 1149:
```

```
 1150:                 dn_Ks = (1./6)*(k1_n_Ks + 2*k2_n_Ks + 2*k3_n_Ks + k4_n_Ks)
```

```
      __pyx_v_dn_Ks = ((1. / 6.0) * (((__pyx_v_k1_n_Ks + (2.0 * __pyx_v_k2_n_Ks)) + (2.0 * __pyx_v_k3_n_Ks)) + __pyx_v_k4_n_Ks));
```

```
 1151:
```

```
 1152:
```

```
 1153:
```

```
 1154:                 I_Ks = G_Ks*(n_Ks**2)*(V-E_K)
```

```
      __pyx_v_I_Ks = ((__pyx_v_G_Ks * pow(__pyx_v_n_Ks, 2.0)) * (__pyx_v_V - __pyx_v_E_K));
```

```
 1155:
```

```
 1156:
```

```
 1157:                 k1_a_ur = self.f_a_ur(a_ss, a_ur, t_aur)
```

```
      __pyx_v_k1_a_ur = ((struct __pyx_vtabstruct_5model_Bondarenko *)__pyx_v_self->__pyx_vtab)->f_a_ur(__pyx_v_self, __pyx_v_a_ss, __pyx_v_a_ur, __pyx_v_t_aur);
```

```
 1158:
```

```
 1159:                 k2_a_ur = self.f_a_ur(a_ss + (1./2)*k1_a_ur, a_ur + (1./2)*k1_a_ur, t_aur + (1./2)*k1_a_ur)
```

```
      __pyx_v_k2_a_ur = ((struct __pyx_vtabstruct_5model_Bondarenko *)__pyx_v_self->__pyx_vtab)->f_a_ur(__pyx_v_self, (__pyx_v_a_ss + ((1. / 2.0) * __pyx_v_k1_a_ur)), (__pyx_v_a_ur + ((1. / 2.0) * __pyx_v_k1_a_ur)), (__pyx_v_t_aur + ((1. / 2.0) * __pyx_v_k1_a_ur)));
```

```
 1160:
```

```
 1161:                 k3_a_ur = self.f_a_ur(a_ss + (1./2)*k2_a_ur, a_ur + (1./2)*k2_a_ur, t_aur + (1./2)*k2_a_ur)
```

```
      __pyx_v_k3_a_ur = ((struct __pyx_vtabstruct_5model_Bondarenko *)__pyx_v_self->__pyx_vtab)->f_a_ur(__pyx_v_self, (__pyx_v_a_ss + ((1. / 2.0) * __pyx_v_k2_a_ur)), (__pyx_v_a_ur + ((1. / 2.0) * __pyx_v_k2_a_ur)), (__pyx_v_t_aur + ((1. / 2.0) * __pyx_v_k2_a_ur)));
```

```
 1162:
```

```
 1163:                 k4_a_ur = self.f_a_ur(a_ss + k3_a_ur, a_ur + k3_a_ur, t_aur + k3_a_ur)
```

```
      __pyx_v_k4_a_ur = ((struct __pyx_vtabstruct_5model_Bondarenko *)__pyx_v_self->__pyx_vtab)->f_a_ur(__pyx_v_self, (__pyx_v_a_ss + __pyx_v_k3_a_ur), (__pyx_v_a_ur + __pyx_v_k3_a_ur), (__pyx_v_t_aur + __pyx_v_k3_a_ur));
```

```
 1164:
```

```
 1165:
```

```
 1166:                 da_ur = (1./6)*(k1_a_ur + 2*k2_a_ur + 2*k3_a_ur + k4_a_ur)
```

```
      __pyx_v_da_ur = ((1. / 6.0) * (((__pyx_v_k1_a_ur + (2.0 * __pyx_v_k2_a_ur)) + (2.0 * __pyx_v_k3_a_ur)) + __pyx_v_k4_a_ur));
```

```
 1167:
```

```
 1168:
```

```
 1169:
```

```
 1170:
```

```
 1171:                 k1_i_ur = self.f_i_ur(i_ss, i_ur, t_iur)
```

```
      __pyx_v_k1_i_ur = ((struct __pyx_vtabstruct_5model_Bondarenko *)__pyx_v_self->__pyx_vtab)->f_i_ur(__pyx_v_self, __pyx_v_i_ss, __pyx_v_i_ur, __pyx_v_t_iur);
```

```
 1172:
```

```
 1173:                 k2_i_ur = self.f_i_ur(i_ss + (1./2)*k1_i_ur, i_ur + (1./2)*k1_i_ur, t_iur + (1./2)*k1_i_ur)
```

```
      __pyx_v_k2_i_ur = ((struct __pyx_vtabstruct_5model_Bondarenko *)__pyx_v_self->__pyx_vtab)->f_i_ur(__pyx_v_self, (__pyx_v_i_ss + ((1. / 2.0) * __pyx_v_k1_i_ur)), (__pyx_v_i_ur + ((1. / 2.0) * __pyx_v_k1_i_ur)), (__pyx_v_t_iur + ((1. / 2.0) * __pyx_v_k1_i_ur)));
```

```
 1174:
```

```
 1175:                 k3_i_ur = self.f_i_ur(i_ss + (1./2)*k2_i_ur, i_ur + (1./2)*k2_i_ur, t_iur + (1./2)*k2_i_ur)
```

```
      __pyx_v_k3_i_ur = ((struct __pyx_vtabstruct_5model_Bondarenko *)__pyx_v_self->__pyx_vtab)->f_i_ur(__pyx_v_self, (__pyx_v_i_ss + ((1. / 2.0) * __pyx_v_k2_i_ur)), (__pyx_v_i_ur + ((1. / 2.0) * __pyx_v_k2_i_ur)), (__pyx_v_t_iur + ((1. / 2.0) * __pyx_v_k2_i_ur)));
```

```
 1176:
```

```
 1177:                 k4_i_ur = self.f_i_ur(i_ss + k3_i_ur, i_ur + k3_i_ur, t_iur + k3_i_ur)
```

```
      __pyx_v_k4_i_ur = ((struct __pyx_vtabstruct_5model_Bondarenko *)__pyx_v_self->__pyx_vtab)->f_i_ur(__pyx_v_self, (__pyx_v_i_ss + __pyx_v_k3_i_ur), (__pyx_v_i_ur + __pyx_v_k3_i_ur), (__pyx_v_t_iur + __pyx_v_k3_i_ur));
```

```
 1178:
```

```
 1179:
```

```
 1180:                 di_ur =  (1./6)*(k1_i_ur + 2*k2_i_ur + 2*k3_i_ur + k4_i_ur)
```

```
      __pyx_v_di_ur = ((1. / 6.0) * (((__pyx_v_k1_i_ur + (2.0 * __pyx_v_k2_i_ur)) + (2.0 * __pyx_v_k3_i_ur)) + __pyx_v_k4_i_ur));
```

```
 1181:
```

```
 1182:
```

```
 1183:
```

```
 1184:                 I_Kur = G_Kur*a_ur*i_ur*(V - E_K)
```

```
      __pyx_v_I_Kur = (((__pyx_v_G_Kur * __pyx_v_a_ur) * __pyx_v_i_ur) * (__pyx_v_V - __pyx_v_E_K));
```

```
 1185:
```

```
 1186:                 I_Kr = O_K*G_Kr*(V - ((R*T)/F)*log((0.98*K_0 + 0.02*Na_0)/(0.98*K_i + 0.02*Na_i)))
```

```
      __pyx_v_I_Kr = ((__pyx_v_O_K * __pyx_v_G_Kr) * (__pyx_v_V - (((__pyx_v_R * __pyx_v_T) / __pyx_v_F) * log((((0.98 * __pyx_v_K_0) + (0.02 * __pyx_v_Na_0)) / ((0.98 * __pyx_v_K_i) + (0.02 * __pyx_v_Na_i)))))));
```

```
 1187:
```

```
 1188:
```

```
 1189:
```

```
 1190:                 k1_K_i = self.f_K_i(I_Ktof, I_Ktos, I_K1, I_Kss, I_Ks, I_Kur, I_Kr, I_NaK)
```

```
      __pyx_v_k1_K_i = ((struct __pyx_vtabstruct_5model_Bondarenko *)__pyx_v_self->__pyx_vtab)->f_K_i(__pyx_v_self, __pyx_v_I_Ktof, __pyx_v_I_Ktos, __pyx_v_I_K1, __pyx_v_I_Kss, __pyx_v_I_Ks, __pyx_v_I_Kur, __pyx_v_I_Kr, __pyx_v_I_NaK);
```

```
 1191:
```

```
 1192:                 k2_K_i = self.f_K_i(I_Ktof + (1./2)*k1_K_i, I_Ktos + (1./2)*k1_K_i, I_K1 + (1./2)*k1_K_i,
```

```
      __pyx_v_k2_K_i = ((struct __pyx_vtabstruct_5model_Bondarenko *)__pyx_v_self->__pyx_vtab)->f_K_i(__pyx_v_self, (__pyx_v_I_Ktof + ((1. / 2.0) * __pyx_v_k1_K_i)), (__pyx_v_I_Ktos + ((1. / 2.0) * __pyx_v_k1_K_i)), (__pyx_v_I_K1 + ((1. / 2.0) * __pyx_v_k1_K_i)), (__pyx_v_I_Kss + ((1. / 2.0) * __pyx_v_k1_K_i)), (__pyx_v_I_Ks + ((1. / 2.0) * __pyx_v_k1_K_i)), (__pyx_v_I_Kur + ((1. / 2.0) * __pyx_v_k1_K_i)), (__pyx_v_I_Kr + ((1. / 2.0) * __pyx_v_k1_K_i)), (__pyx_v_I_NaK + ((1. / 2.0) * __pyx_v_k1_K_i)));
```

```
 1193:                            I_Kss + (1./2)*k1_K_i, I_Ks + (1./2)*k1_K_i, I_Kur + (1./2)*k1_K_i,
```

```
 1194:                             I_Kr + (1./2)*k1_K_i, I_NaK + (1./2)*k1_K_i)
```

```
 1195:
```

```
 1196:                 k3_K_i = self.f_K_i(I_Ktof + (1./2)*k2_K_i, I_Ktos + (1./2)*k2_K_i, I_K1 + (1./2)*k2_K_i,
```

```
      __pyx_v_k3_K_i = ((struct __pyx_vtabstruct_5model_Bondarenko *)__pyx_v_self->__pyx_vtab)->f_K_i(__pyx_v_self, (__pyx_v_I_Ktof + ((1. / 2.0) * __pyx_v_k2_K_i)), (__pyx_v_I_Ktos + ((1. / 2.0) * __pyx_v_k2_K_i)), (__pyx_v_I_K1 + ((1. / 2.0) * __pyx_v_k2_K_i)), (__pyx_v_I_Kss + ((1. / 2.0) * __pyx_v_k2_K_i)), (__pyx_v_I_Ks + ((1. / 2.0) * __pyx_v_k2_K_i)), (__pyx_v_I_Kur + ((1. / 2.0) * __pyx_v_k2_K_i)), (__pyx_v_I_Kr + ((1. / 2.0) * __pyx_v_k2_K_i)), (__pyx_v_I_NaK + ((1. / 2.0) * __pyx_v_k2_K_i)));
```

```
 1197:                            I_Kss + (1./2)*k2_K_i, I_Ks + (1./2)*k2_K_i, I_Kur + (1./2)*k2_K_i,
```

```
 1198:                             I_Kr + (1./2)*k2_K_i, I_NaK + (1./2)*k2_K_i)
```

```
 1199:
```

```
 1200:                 k4_K_i = self.f_K_i(I_Ktof + k3_K_i, I_Ktos + k3_K_i, I_K1 + k3_K_i,
```

```
      __pyx_v_k4_K_i = ((struct __pyx_vtabstruct_5model_Bondarenko *)__pyx_v_self->__pyx_vtab)->f_K_i(__pyx_v_self, (__pyx_v_I_Ktof + __pyx_v_k3_K_i), (__pyx_v_I_Ktos + __pyx_v_k3_K_i), (__pyx_v_I_K1 + __pyx_v_k3_K_i), (__pyx_v_I_Kss + __pyx_v_k3_K_i), (__pyx_v_I_Ks + __pyx_v_k3_K_i), (__pyx_v_I_Kur + __pyx_v_k3_K_i), (__pyx_v_I_Kr + __pyx_v_k3_K_i), (__pyx_v_I_NaK + __pyx_v_k3_K_i));
```

```
 1201:                            I_Kss + k3_K_i, I_Ks + k3_K_i, I_Kur + k3_K_i,
```

```
 1202:                             I_Kr + k3_K_i, I_NaK + k3_K_i)
```

```
 1203:
```

```
 1204:
```

```
 1205:                 dK_i = (1./6)*(k1_K_i + 2*k2_K_i + 2*k3_K_i + k4_K_i)
```

```
      __pyx_v_dK_i = ((1. / 6.0) * (((__pyx_v_k1_K_i + (2.0 * __pyx_v_k2_K_i)) + (2.0 * __pyx_v_k3_K_i)) + __pyx_v_k4_K_i));
```

```
 1206:
```

```
 1207:
```

```
 1208:
```

```
 1209:
```

```
 1210:
```

```
 1211:                 C_K0 = 1 - (C_K1 + C_K2 + O_K + I_K)
```

```
      __pyx_v_C_K0 = (1.0 - (((__pyx_v_C_K1 + __pyx_v_C_K2) + __pyx_v_O_K) + __pyx_v_I_K));
```

```
 1212:
```

```
 1213:                 a_a0 = 0.022348*exp(0.01176*V)
```

```
      __pyx_v_a_a0 = (0.022348 * exp((0.01176 * __pyx_v_V)));
```

```
 1214:
```

```
 1215:                 B_a0 = 0.047002*exp(-0.0631*V)
```

```
      __pyx_v_B_a0 = (0.047002 * exp((-0.0631 * __pyx_v_V)));
```

```
 1216:
```

```
 1217:                 a_a1 = 0.013733*exp(0.038198*V)
```

```
      __pyx_v_a_a1 = (0.013733 * exp((0.038198 * __pyx_v_V)));
```

```
 1218:
```

```
 1219:                 B_a1 = 0.0000689*exp(-0.04178*V)
```

```
      __pyx_v_B_a1 = (0.0000689 * exp((-0.04178 * __pyx_v_V)));
```

```
 1220:
```

```
 1221:                 a_i_r = 0.090821*exp(0.023391*(V + 5.))
```

```
      __pyx_v_a_i_r = (0.090821 * exp((0.023391 * (__pyx_v_V + 5.))));
```

```
 1222:
```

```
 1223:                 B_i_r = 0.006497*exp(-0.03268*(V + 5.))
```

```
      __pyx_v_B_i_r = (0.006497 * exp((-0.03268 * (__pyx_v_V + 5.))));
```

```
 1224:
```

```
 1225:
```

```
 1226:
```

```
 1227:                 k1_C_K1 = self.f_C_K1(a_a0, C_K0, B_a0, C_K1, C_K2)
```

```
      __pyx_v_k1_C_K1 = ((struct __pyx_vtabstruct_5model_Bondarenko *)__pyx_v_self->__pyx_vtab)->f_C_K1(__pyx_v_self, __pyx_v_a_a0, __pyx_v_C_K0, __pyx_v_B_a0, __pyx_v_C_K1, __pyx_v_C_K2);
```

```
 1228:
```

```
 1229:                 k2_C_K1 = self.f_C_K1(a_a0 + (1./2)*k1_C_K1, C_K0 + (1./2)*k1_C_K1, B_a0 + (1./2)*k1_C_K1,
```

```
      __pyx_v_k2_C_K1 = ((struct __pyx_vtabstruct_5model_Bondarenko *)__pyx_v_self->__pyx_vtab)->f_C_K1(__pyx_v_self, (__pyx_v_a_a0 + ((1. / 2.0) * __pyx_v_k1_C_K1)), (__pyx_v_C_K0 + ((1. / 2.0) * __pyx_v_k1_C_K1)), (__pyx_v_B_a0 + ((1. / 2.0) * __pyx_v_k1_C_K1)), (__pyx_v_C_K1 + ((1. / 2.0) * __pyx_v_k1_C_K1)), (__pyx_v_C_K2 + ((1. / 2.0) * __pyx_v_k1_C_K1)));
```

```
 1230:                                C_K1 + (1./2)*k1_C_K1, C_K2 + (1./2)*k1_C_K1)
```

```
 1231:
```

```
 1232:                 k3_C_K1 = self.f_C_K1(a_a0 + (1./2)*k2_C_K1, C_K0 + (1./2)*k2_C_K1, B_a0 + (1./2)*k2_C_K1,
```

```
      __pyx_v_k3_C_K1 = ((struct __pyx_vtabstruct_5model_Bondarenko *)__pyx_v_self->__pyx_vtab)->f_C_K1(__pyx_v_self, (__pyx_v_a_a0 + ((1. / 2.0) * __pyx_v_k2_C_K1)), (__pyx_v_C_K0 + ((1. / 2.0) * __pyx_v_k2_C_K1)), (__pyx_v_B_a0 + ((1. / 2.0) * __pyx_v_k2_C_K1)), (__pyx_v_C_K1 + ((1. / 2.0) * __pyx_v_k2_C_K1)), (__pyx_v_C_K2 + ((1. / 2.0) * __pyx_v_k2_C_K1)));
```

```
 1233:                                C_K1 + (1./2)*k2_C_K1, C_K2 + (1./2)*k2_C_K1)
```

```
 1234:
```

```
 1235:                 k4_C_K1 = self.f_C_K1(a_a0 + k3_C_K1, C_K0 + k3_C_K1, B_a0 + k3_C_K1,
```

```
      __pyx_v_k4_C_K1 = ((struct __pyx_vtabstruct_5model_Bondarenko *)__pyx_v_self->__pyx_vtab)->f_C_K1(__pyx_v_self, (__pyx_v_a_a0 + __pyx_v_k3_C_K1), (__pyx_v_C_K0 + __pyx_v_k3_C_K1), (__pyx_v_B_a0 + __pyx_v_k3_C_K1), (__pyx_v_C_K1 + __pyx_v_k3_C_K1), (__pyx_v_C_K2 + __pyx_v_k3_C_K1));
```

```
 1236:                                C_K1 + k3_C_K1, C_K2 + k3_C_K1)
```

```
 1237:
```

```
 1238:                 dC_K1 =  (1./6)*(k1_C_K1 + 2*k2_C_K1 + 2*k3_C_K1 + k4_C_K1)
```

```
      __pyx_v_dC_K1 = ((1. / 6.0) * (((__pyx_v_k1_C_K1 + (2.0 * __pyx_v_k2_C_K1)) + (2.0 * __pyx_v_k3_C_K1)) + __pyx_v_k4_C_K1));
```

```
 1239:
```

```
 1240:
```

```
 1241:
```

```
 1242:
```

```
 1243:                 k1_C_K2 = self.f_C_K2(C_K1, C_K2, B_a1, O_K, a_a1)
```

```
      __pyx_v_k1_C_K2 = ((struct __pyx_vtabstruct_5model_Bondarenko *)__pyx_v_self->__pyx_vtab)->f_C_K2(__pyx_v_self, __pyx_v_C_K1, __pyx_v_C_K2, __pyx_v_B_a1, __pyx_v_O_K, __pyx_v_a_a1);
```

```
 1244:
```

```
 1245:                 k2_C_K2 = self.f_C_K2(C_K1 + (1./2)*k1_C_K2, C_K2 + (1./2)*k1_C_K2,
```

```
      __pyx_v_k2_C_K2 = ((struct __pyx_vtabstruct_5model_Bondarenko *)__pyx_v_self->__pyx_vtab)->f_C_K2(__pyx_v_self, (__pyx_v_C_K1 + ((1. / 2.0) * __pyx_v_k1_C_K2)), (__pyx_v_C_K2 + ((1. / 2.0) * __pyx_v_k1_C_K2)), (__pyx_v_B_a1 + ((1. / 2.0) * __pyx_v_k1_C_K2)), (__pyx_v_O_K + ((1. / 2.0) * __pyx_v_k1_C_K2)), (__pyx_v_a_a1 + ((1. / 2.0) * __pyx_v_k1_C_K2)));
```

```
 1246:                                  B_a1 + (1./2)*k1_C_K2,  O_K + (1./2)*k1_C_K2, a_a1 + (1./2)*k1_C_K2)
```

```
 1247:
```

```
 1248:                 k3_C_K2 = self.f_C_K2(C_K1 + (1./2)*k2_C_K2, C_K2 + (1./2)*k2_C_K2,
```

```
      __pyx_v_k3_C_K2 = ((struct __pyx_vtabstruct_5model_Bondarenko *)__pyx_v_self->__pyx_vtab)->f_C_K2(__pyx_v_self, (__pyx_v_C_K1 + ((1. / 2.0) * __pyx_v_k2_C_K2)), (__pyx_v_C_K2 + ((1. / 2.0) * __pyx_v_k2_C_K2)), (__pyx_v_B_a1 + ((1. / 2.0) * __pyx_v_k2_C_K2)), (__pyx_v_O_K + ((1. / 2.0) * __pyx_v_k2_C_K2)), (__pyx_v_a_a1 + ((1. / 2.0) * __pyx_v_k2_C_K2)));
```

```
 1249:                                  B_a1 + (1./2)*k2_C_K2,  O_K + (1./2)*k2_C_K2, a_a1 + (1./2)*k2_C_K2)
```

```
 1250:
```

```
 1251:                 k4_C_K2 = self.f_C_K2(C_K1 + k3_C_K2, C_K2 + k3_C_K2,
```

```
      __pyx_v_k4_C_K2 = ((struct __pyx_vtabstruct_5model_Bondarenko *)__pyx_v_self->__pyx_vtab)->f_C_K2(__pyx_v_self, (__pyx_v_C_K1 + __pyx_v_k3_C_K2), (__pyx_v_C_K2 + __pyx_v_k3_C_K2), (__pyx_v_B_a1 + __pyx_v_k3_C_K2), (__pyx_v_O_K + __pyx_v_k3_C_K2), (__pyx_v_a_a1 + __pyx_v_k3_C_K2));
```

```
 1252:                                  B_a1 + k3_C_K2,  O_K + k3_C_K2, a_a1 + k3_C_K2)
```

```
 1253:
```

```
 1254:
```

```
 1255:                 dC_K2 = (1./6)*(k1_C_K2 + 2*k2_C_K2 + 2*k3_C_K2 + k4_C_K2)
```

```
      __pyx_v_dC_K2 = ((1. / 6.0) * (((__pyx_v_k1_C_K2 + (2.0 * __pyx_v_k2_C_K2)) + (2.0 * __pyx_v_k3_C_K2)) + __pyx_v_k4_C_K2));
```

```
 1256:
```

```
 1257:
```

```
 1258:
```

```
 1259:
```

```
 1260:                 k1_O_K = self.f_O_K(a_a1, C_K2, B_a1, O_K, B_i_r, I_K, a_i_r)
```

```
      __pyx_v_k1_O_K = ((struct __pyx_vtabstruct_5model_Bondarenko *)__pyx_v_self->__pyx_vtab)->f_O_K(__pyx_v_self, __pyx_v_a_a1, __pyx_v_C_K2, __pyx_v_B_a1, __pyx_v_O_K, __pyx_v_B_i_r, __pyx_v_I_K, __pyx_v_a_i_r);
```

```
 1261:
```

```
 1262:                 k2_O_K = self.f_O_K(a_a1 + (1./2)*k1_O_K, C_K2 + (1./2)*k1_O_K, B_a1 + (1./2)*k1_O_K,
```

```
      __pyx_v_k2_O_K = ((struct __pyx_vtabstruct_5model_Bondarenko *)__pyx_v_self->__pyx_vtab)->f_O_K(__pyx_v_self, (__pyx_v_a_a1 + ((1. / 2.0) * __pyx_v_k1_O_K)), (__pyx_v_C_K2 + ((1. / 2.0) * __pyx_v_k1_O_K)), (__pyx_v_B_a1 + ((1. / 2.0) * __pyx_v_k1_O_K)), (__pyx_v_O_K + ((1. / 2.0) * __pyx_v_k1_O_K)), (__pyx_v_B_i_r + ((1. / 2.0) * __pyx_v_k1_O_K)), (__pyx_v_I_K + ((1. / 2.0) * __pyx_v_k1_O_K)), (__pyx_v_a_i_r + ((1. / 2.0) * __pyx_v_k1_O_K)));
```

```
 1263:                              O_K + (1./2)*k1_O_K, B_i_r + (1./2)*k1_O_K, I_K + (1./2)*k1_O_K, a_i_r + (1./2)*k1_O_K)
```

```
 1264:
```

```
 1265:                 k3_O_K = self.f_O_K(a_a1 + (1./2)*k2_O_K, C_K2 + (1./2)*k2_O_K, B_a1 + (1./2)*k2_O_K,
```

```
      __pyx_v_k3_O_K = ((struct __pyx_vtabstruct_5model_Bondarenko *)__pyx_v_self->__pyx_vtab)->f_O_K(__pyx_v_self, (__pyx_v_a_a1 + ((1. / 2.0) * __pyx_v_k2_O_K)), (__pyx_v_C_K2 + ((1. / 2.0) * __pyx_v_k2_O_K)), (__pyx_v_B_a1 + ((1. / 2.0) * __pyx_v_k2_O_K)), (__pyx_v_O_K + ((1. / 2.0) * __pyx_v_k2_O_K)), (__pyx_v_B_i_r + ((1. / 2.0) * __pyx_v_k2_O_K)), (__pyx_v_I_K + ((1. / 2.0) * __pyx_v_k2_O_K)), (__pyx_v_a_i_r + ((1. / 2.0) * __pyx_v_k2_O_K)));
```

```
 1266:                              O_K + (1./2)*k2_O_K, B_i_r + (1./2)*k2_O_K, I_K + (1./2)*k2_O_K, a_i_r + (1./2)*k2_O_K)
```

```
 1267:
```

```
 1268:                 k4_O_K = self.f_O_K(a_a1 + k3_O_K, C_K2 + k3_O_K, B_a1 + k3_O_K,
```

```
      __pyx_v_k4_O_K = ((struct __pyx_vtabstruct_5model_Bondarenko *)__pyx_v_self->__pyx_vtab)->f_O_K(__pyx_v_self, (__pyx_v_a_a1 + __pyx_v_k3_O_K), (__pyx_v_C_K2 + __pyx_v_k3_O_K), (__pyx_v_B_a1 + __pyx_v_k3_O_K), (__pyx_v_O_K + __pyx_v_k3_O_K), (__pyx_v_B_i_r + __pyx_v_k3_O_K), (__pyx_v_I_K + __pyx_v_k3_O_K), (__pyx_v_a_i_r + __pyx_v_k3_O_K));
```

```
 1269:                              O_K + k3_O_K, B_i_r + k3_O_K, I_K + k3_O_K, a_i_r + k3_O_K)
```

```
 1270:
```

```
 1271:
```

```
 1272:                 dO_K =  (1./6)*(k1_O_K + 2*k2_O_K + 2*k3_O_K + k4_O_K)
```

```
      __pyx_v_dO_K = ((1. / 6.0) * (((__pyx_v_k1_O_K + (2.0 * __pyx_v_k2_O_K)) + (2.0 * __pyx_v_k3_O_K)) + __pyx_v_k4_O_K));
```

```
 1273:
```

```
 1274:
```

```
 1275:
```

```
 1276:
```

```
 1277:                 k1_I_K = self.f_I_K(a_i_r, O_K, B_i_r, I_K)
```

```
      __pyx_v_k1_I_K = ((struct __pyx_vtabstruct_5model_Bondarenko *)__pyx_v_self->__pyx_vtab)->f_I_K(__pyx_v_self, __pyx_v_a_i_r, __pyx_v_O_K, __pyx_v_B_i_r, __pyx_v_I_K);
```

```
 1278:
```

```
 1279:                 k2_I_K = self.f_I_K(a_i_r + (1./2)*k1_I_K, O_K + (1./2)*k1_I_K, B_i_r + (1./2)*k1_I_K, I_K + (1./2)*k1_I_K)
```

```
      __pyx_v_k2_I_K = ((struct __pyx_vtabstruct_5model_Bondarenko *)__pyx_v_self->__pyx_vtab)->f_I_K(__pyx_v_self, (__pyx_v_a_i_r + ((1. / 2.0) * __pyx_v_k1_I_K)), (__pyx_v_O_K + ((1. / 2.0) * __pyx_v_k1_I_K)), (__pyx_v_B_i_r + ((1. / 2.0) * __pyx_v_k1_I_K)), (__pyx_v_I_K + ((1. / 2.0) * __pyx_v_k1_I_K)));
```

```
 1280:
```

```
 1281:                 k3_I_K = self.f_I_K(a_i_r + (1./2)*k2_I_K, O_K + (1./2)*k2_I_K, B_i_r + (1./2)*k2_I_K, I_K + (1./2)*k2_I_K)
```

```
      __pyx_v_k3_I_K = ((struct __pyx_vtabstruct_5model_Bondarenko *)__pyx_v_self->__pyx_vtab)->f_I_K(__pyx_v_self, (__pyx_v_a_i_r + ((1. / 2.0) * __pyx_v_k2_I_K)), (__pyx_v_O_K + ((1. / 2.0) * __pyx_v_k2_I_K)), (__pyx_v_B_i_r + ((1. / 2.0) * __pyx_v_k2_I_K)), (__pyx_v_I_K + ((1. / 2.0) * __pyx_v_k2_I_K)));
```

```
 1282:
```

```
 1283:                 k4_I_K = self.f_I_K(a_i_r + k3_I_K, O_K + k3_I_K, B_i_r + k3_I_K, I_K + k3_I_K)
```

```
      __pyx_v_k4_I_K = ((struct __pyx_vtabstruct_5model_Bondarenko *)__pyx_v_self->__pyx_vtab)->f_I_K(__pyx_v_self, (__pyx_v_a_i_r + __pyx_v_k3_I_K), (__pyx_v_O_K + __pyx_v_k3_I_K), (__pyx_v_B_i_r + __pyx_v_k3_I_K), (__pyx_v_I_K + __pyx_v_k3_I_K));
```

```
 1284:
```

```
 1285:
```

```
 1286:                 dI_K =  (1./6)*(k1_I_K + 2*k2_O_K + 2*k3_I_K + k4_I_K)
```

```
      __pyx_v_dI_K = ((1. / 6.0) * (((__pyx_v_k1_I_K + (2.0 * __pyx_v_k2_O_K)) + (2.0 * __pyx_v_k3_I_K)) + __pyx_v_k4_I_K));
```

```
 1287:
```

```
 1288:
```

```
 1289:
```

```
 1290:
```

```
 1291:                 O_ClCa = 0.2/(1 + exp(-(V-46.7)/7.8))
```

```
      __pyx_v_O_ClCa = (0.2 / (1.0 + exp(((-(__pyx_v_V - 46.7)) / 7.8))));
```

```
 1292:
```

```
 1293:                 I_ClCa = G_ClCa*O_ClCa*(Ca2_i/(Ca2_i + K_mCl))*(V - E_Cl)
```

```
      __pyx_v_I_ClCa = (((__pyx_v_G_ClCa * __pyx_v_O_ClCa) * (__pyx_v_Ca2_i / (__pyx_v_Ca2_i + __pyx_v_K_mCl))) * (__pyx_v_V - __pyx_v_E_Cl));
```

```
 1294:
```

```
 1295:
```

```
 1296:
```

```
 1297:
```

```
 1298:                 k1_V = self.f_V(I_CaL, I_pCa, I_NaCa, I_Cab, I_Na, I_Nab,
```

```
      __pyx_v_k1_V = ((struct __pyx_vtabstruct_5model_Bondarenko *)__pyx_v_self->__pyx_vtab)->f_V(__pyx_v_self, __pyx_v_I_CaL, __pyx_v_I_pCa, __pyx_v_I_NaCa, __pyx_v_I_Cab, __pyx_v_I_Na, __pyx_v_I_Nab, __pyx_v_I_NaK, __pyx_v_I_Ktof, __pyx_v_I_Ktos, __pyx_v_I_K1, __pyx_v_I_Ks, __pyx_v_I_Kur, __pyx_v_I_Kss, __pyx_v_I_Kr, __pyx_v_I_ClCa, __pyx_v_I_stim);
```

```
 1299:                          I_NaK, I_Ktof, I_Ktos, I_K1, I_Ks, I_Kur,
```

```
 1300:                          I_Kss, I_Kr, I_ClCa, I_stim)
```

```
 1301:
```

```
 1302:                 k2_V = self.f_V(I_CaL + (1./2)*k1_V, I_pCa + (1./2)*k1_V, I_NaCa + (1./2)*k1_V, I_Cab + (1./2)*k1_V, I_Na + (1./2)*k1_V, I_Nab + (1./2)*k1_V,
```

```
      __pyx_v_k2_V = ((struct __pyx_vtabstruct_5model_Bondarenko *)__pyx_v_self->__pyx_vtab)->f_V(__pyx_v_self, (__pyx_v_I_CaL + ((1. / 2.0) * __pyx_v_k1_V)), (__pyx_v_I_pCa + ((1. / 2.0) * __pyx_v_k1_V)), (__pyx_v_I_NaCa + ((1. / 2.0) * __pyx_v_k1_V)), (__pyx_v_I_Cab + ((1. / 2.0) * __pyx_v_k1_V)), (__pyx_v_I_Na + ((1. / 2.0) * __pyx_v_k1_V)), (__pyx_v_I_Nab + ((1. / 2.0) * __pyx_v_k1_V)), (__pyx_v_I_NaK + ((1. / 2.0) * __pyx_v_k1_V)), (__pyx_v_I_Ktof + ((1. / 2.0) * __pyx_v_k1_V)), (__pyx_v_I_Ktos + ((1. / 2.0) * __pyx_v_k1_V)), (__pyx_v_I_K1 + ((1. / 2.0) * __pyx_v_k1_V)), (__pyx_v_I_Ks + ((1. / 2.0) * __pyx_v_k1_V)), (__pyx_v_I_Kur + ((1. / 2.0) * __pyx_v_k1_V)), (__pyx_v_I_Kss + ((1. / 2.0) * __pyx_v_k1_V)), (__pyx_v_I_Kr + ((1. / 2.0) * __pyx_v_k1_V)), (__pyx_v_I_ClCa + ((1. / 2.0) * __pyx_v_k1_V)), __pyx_v_I_stim);
```

```
 1303:                          I_NaK + (1./2)*k1_V, I_Ktof + (1./2)*k1_V, I_Ktos + (1./2)*k1_V, I_K1 + (1./2)*k1_V, I_Ks + (1./2)*k1_V, I_Kur + (1./2)*k1_V,
```

```
 1304:                          I_Kss + (1./2)*k1_V, I_Kr + (1./2)*k1_V, I_ClCa + (1./2)*k1_V, I_stim)
```

```
 1305:
```

```
 1306:                 k3_V = self.f_V(I_CaL + (1./2)*k2_V, I_pCa + (1./2)*k2_V, I_NaCa + (1./2)*k2_V, I_Cab + (1./2)*k2_V, I_Na + (1./2)*k2_V, I_Nab + (1./2)*k2_V,
```

```
      __pyx_v_k3_V = ((struct __pyx_vtabstruct_5model_Bondarenko *)__pyx_v_self->__pyx_vtab)->f_V(__pyx_v_self, (__pyx_v_I_CaL + ((1. / 2.0) * __pyx_v_k2_V)), (__pyx_v_I_pCa + ((1. / 2.0) * __pyx_v_k2_V)), (__pyx_v_I_NaCa + ((1. / 2.0) * __pyx_v_k2_V)), (__pyx_v_I_Cab + ((1. / 2.0) * __pyx_v_k2_V)), (__pyx_v_I_Na + ((1. / 2.0) * __pyx_v_k2_V)), (__pyx_v_I_Nab + ((1. / 2.0) * __pyx_v_k2_V)), (__pyx_v_I_NaK + ((1. / 2.0) * __pyx_v_k2_V)), (__pyx_v_I_Ktof + ((1. / 2.0) * __pyx_v_k2_V)), (__pyx_v_I_Ktos + ((1. / 2.0) * __pyx_v_k2_V)), (__pyx_v_I_K1 + ((1. / 2.0) * __pyx_v_k2_V)), (__pyx_v_I_Ks + ((1. / 2.0) * __pyx_v_k2_V)), (__pyx_v_I_Kur + ((1. / 2.0) * __pyx_v_k2_V)), (__pyx_v_I_Kss + ((1. / 2.0) * __pyx_v_k2_V)), (__pyx_v_I_Kr + ((1. / 2.0) * __pyx_v_k2_V)), (__pyx_v_I_ClCa + ((1. / 2.0) * __pyx_v_k2_V)), __pyx_v_I_stim);
```

```
 1307:                          I_NaK + (1./2)*k2_V, I_Ktof + (1./2)*k2_V, I_Ktos + (1./2)*k2_V, I_K1 + (1./2)*k2_V, I_Ks + (1./2)*k2_V, I_Kur + (1./2)*k2_V,
```

```
 1308:                          I_Kss + (1./2)*k2_V, I_Kr + (1./2)*k2_V, I_ClCa + (1./2)*k2_V, I_stim)
```

```
 1309:
```

```
 1310:                 k4_V = self.f_V(I_CaL + k3_V, I_pCa + k3_V, I_NaCa + k3_V, I_Cab + k3_V, I_Na + k3_V, I_Nab + k3_V,
```

```
      __pyx_v_k4_V = ((struct __pyx_vtabstruct_5model_Bondarenko *)__pyx_v_self->__pyx_vtab)->f_V(__pyx_v_self, (__pyx_v_I_CaL + __pyx_v_k3_V), (__pyx_v_I_pCa + __pyx_v_k3_V), (__pyx_v_I_NaCa + __pyx_v_k3_V), (__pyx_v_I_Cab + __pyx_v_k3_V), (__pyx_v_I_Na + __pyx_v_k3_V), (__pyx_v_I_Nab + __pyx_v_k3_V), (__pyx_v_I_NaK + __pyx_v_k3_V), (__pyx_v_I_Ktof + __pyx_v_k3_V), (__pyx_v_I_Ktos + __pyx_v_k3_V), (__pyx_v_I_K1 + __pyx_v_k3_V), (__pyx_v_I_Ks + __pyx_v_k3_V), (__pyx_v_I_Kur + __pyx_v_k3_V), (__pyx_v_I_Kss + __pyx_v_k3_V), (__pyx_v_I_Kr + __pyx_v_k3_V), (__pyx_v_I_ClCa + __pyx_v_k3_V), __pyx_v_I_stim);
```

```
 1311:                          I_NaK + k3_V, I_Ktof + k3_V, I_Ktos + k3_V, I_K1 + k3_V, I_Ks + k3_V, I_Kur + k3_V,
```

```
 1312:                          I_Kss + k3_V, I_Kr + k3_V, I_ClCa + k3_V, I_stim)
```

```
 1313:
```

```
 1314:                 dV = (1./6)*(k1_V + 2*k2_V + 2*k3_V + k4_V) + dudx2[x, y] + dudy2[x, y]
```

```
      __pyx_t_97 = __pyx_v_x;
      __pyx_t_98 = __pyx_v_y;
      __pyx_t_99 = __pyx_v_x;
      __pyx_t_100 = __pyx_v_y;
      __pyx_t_6 = PyFloat_FromDouble(((((1. / 6.0) * (((__pyx_v_k1_V + (2.0 * __pyx_v_k2_V)) + (2.0 * __pyx_v_k3_V)) + __pyx_v_k4_V)) + (*((double *) ( /* dim=1 */ ((char *) (((double *) ( /* dim=0 */ (__pyx_v_dudx2.data + __pyx_t_97 * __pyx_v_dudx2.strides[0]) )) + __pyx_t_98)) )))) + (*((double *) ( /* dim=1 */ ((char *) (((double *) ( /* dim=0 */ (__pyx_v_dudy2.data + __pyx_t_99 * __pyx_v_dudy2.strides[0]) )) + __pyx_t_100)) ))))); if (unlikely(!__pyx_t_6)) {__pyx_filename = __pyx_f[0]; __pyx_lineno = 1314; __pyx_clineno = __LINE__; goto __pyx_L1_error;}
      __Pyx_GOTREF(__pyx_t_6);
      __Pyx_XDECREF_SET(__pyx_v_dV, __pyx_t_6);
      __pyx_t_6 = 0;
```

```
 1315:
```

```
 1316:
```

```
 1317:
```

```
 1318:                 self.V[x, y] = V + h*dV
```

```
      __pyx_t_6 = PyFloat_FromDouble(__pyx_v_V); if (unlikely(!__pyx_t_6)) {__pyx_filename = __pyx_f[0]; __pyx_lineno = 1318; __pyx_clineno = __LINE__; goto __pyx_L1_error;}
      __Pyx_GOTREF(__pyx_t_6);
      __pyx_t_12 = PyFloat_FromDouble(__pyx_v_h); if (unlikely(!__pyx_t_12)) {__pyx_filename = __pyx_f[0]; __pyx_lineno = 1318; __pyx_clineno = __LINE__; goto __pyx_L1_error;}
      __Pyx_GOTREF(__pyx_t_12);
      __pyx_t_2 = PyNumber_Multiply(__pyx_t_12, __pyx_v_dV); if (unlikely(!__pyx_t_2)) {__pyx_filename = __pyx_f[0]; __pyx_lineno = 1318; __pyx_clineno = __LINE__; goto __pyx_L1_error;}
      __Pyx_GOTREF(__pyx_t_2);
      __Pyx_DECREF(__pyx_t_12); __pyx_t_12 = 0;
      __pyx_t_12 = PyNumber_Add(__pyx_t_6, __pyx_t_2); if (unlikely(!__pyx_t_12)) {__pyx_filename = __pyx_f[0]; __pyx_lineno = 1318; __pyx_clineno = __LINE__; goto __pyx_L1_error;}
      __Pyx_GOTREF(__pyx_t_12);
      __Pyx_DECREF(__pyx_t_6); __pyx_t_6 = 0;
      __Pyx_DECREF(__pyx_t_2); __pyx_t_2 = 0;
      __pyx_t_2 = __Pyx_PyObject_GetAttrStr(((PyObject *)__pyx_v_self), __pyx_n_s_V); if (unlikely(!__pyx_t_2)) {__pyx_filename = __pyx_f[0]; __pyx_lineno = 1318; __pyx_clineno = __LINE__; goto __pyx_L1_error;}
      __Pyx_GOTREF(__pyx_t_2);
      __pyx_t_6 = __Pyx_PyInt_From_int(__pyx_v_x); if (unlikely(!__pyx_t_6)) {__pyx_filename = __pyx_f[0]; __pyx_lineno = 1318; __pyx_clineno = __LINE__; goto __pyx_L1_error;}
      __Pyx_GOTREF(__pyx_t_6);
      __pyx_t_5 = __Pyx_PyInt_From_int(__pyx_v_y); if (unlikely(!__pyx_t_5)) {__pyx_filename = __pyx_f[0]; __pyx_lineno = 1318; __pyx_clineno = __LINE__; goto __pyx_L1_error;}
      __Pyx_GOTREF(__pyx_t_5);
      __pyx_t_1 = PyTuple_New(2); if (unlikely(!__pyx_t_1)) {__pyx_filename = __pyx_f[0]; __pyx_lineno = 1318; __pyx_clineno = __LINE__; goto __pyx_L1_error;}
      __Pyx_GOTREF(__pyx_t_1);
      PyTuple_SET_ITEM(__pyx_t_1, 0, __pyx_t_6);
      __Pyx_GIVEREF(__pyx_t_6);
      PyTuple_SET_ITEM(__pyx_t_1, 1, __pyx_t_5);
      __Pyx_GIVEREF(__pyx_t_5);
      __pyx_t_6 = 0;
      __pyx_t_5 = 0;
      if (unlikely(PyObject_SetItem(__pyx_t_2, __pyx_t_1, __pyx_t_12) < 0)) {__pyx_filename = __pyx_f[0]; __pyx_lineno = 1318; __pyx_clineno = __LINE__; goto __pyx_L1_error;}
      __Pyx_DECREF(__pyx_t_2); __pyx_t_2 = 0;
      __Pyx_DECREF(__pyx_t_1); __pyx_t_1 = 0;
      __Pyx_DECREF(__pyx_t_12); __pyx_t_12 = 0;
```

```
 1319:                 self.Ca2_i[x, y] = Ca2_i + h*dCa2_i
```

```
      __pyx_t_12 = PyFloat_FromDouble((__pyx_v_Ca2_i + (__pyx_v_h * __pyx_v_dCa2_i))); if (unlikely(!__pyx_t_12)) {__pyx_filename = __pyx_f[0]; __pyx_lineno = 1319; __pyx_clineno = __LINE__; goto __pyx_L1_error;}
      __Pyx_GOTREF(__pyx_t_12);
      __pyx_t_1 = __Pyx_PyObject_GetAttrStr(((PyObject *)__pyx_v_self), __pyx_n_s_Ca2_i); if (unlikely(!__pyx_t_1)) {__pyx_filename = __pyx_f[0]; __pyx_lineno = 1319; __pyx_clineno = __LINE__; goto __pyx_L1_error;}
      __Pyx_GOTREF(__pyx_t_1);
      __pyx_t_2 = __Pyx_PyInt_From_int(__pyx_v_x); if (unlikely(!__pyx_t_2)) {__pyx_filename = __pyx_f[0]; __pyx_lineno = 1319; __pyx_clineno = __LINE__; goto __pyx_L1_error;}
      __Pyx_GOTREF(__pyx_t_2);
      __pyx_t_5 = __Pyx_PyInt_From_int(__pyx_v_y); if (unlikely(!__pyx_t_5)) {__pyx_filename = __pyx_f[0]; __pyx_lineno = 1319; __pyx_clineno = __LINE__; goto __pyx_L1_error;}
      __Pyx_GOTREF(__pyx_t_5);
      __pyx_t_6 = PyTuple_New(2); if (unlikely(!__pyx_t_6)) {__pyx_filename = __pyx_f[0]; __pyx_lineno = 1319; __pyx_clineno = __LINE__; goto __pyx_L1_error;}
      __Pyx_GOTREF(__pyx_t_6);
      PyTuple_SET_ITEM(__pyx_t_6, 0, __pyx_t_2);
      __Pyx_GIVEREF(__pyx_t_2);
      PyTuple_SET_ITEM(__pyx_t_6, 1, __pyx_t_5);
      __Pyx_GIVEREF(__pyx_t_5);
      __pyx_t_2 = 0;
      __pyx_t_5 = 0;
      if (unlikely(PyObject_SetItem(__pyx_t_1, __pyx_t_6, __pyx_t_12) < 0)) {__pyx_filename = __pyx_f[0]; __pyx_lineno = 1319; __pyx_clineno = __LINE__; goto __pyx_L1_error;}
      __Pyx_DECREF(__pyx_t_1); __pyx_t_1 = 0;
      __Pyx_DECREF(__pyx_t_6); __pyx_t_6 = 0;
      __Pyx_DECREF(__pyx_t_12); __pyx_t_12 = 0;
```

```
 1320:                 self.Ca2_ss[x, y] = Ca2_ss + h*dCa2_ss
```

```
      if (unlikely(!__pyx_v_self->Ca2_ss.memview)) {PyErr_SetString(PyExc_AttributeError,"Memoryview is not initialized");{__pyx_filename = __pyx_f[0]; __pyx_lineno = 1320; __pyx_clineno = __LINE__; goto __pyx_L1_error;}}
      __pyx_t_101 = __pyx_v_x;
      __pyx_t_102 = __pyx_v_y;
      *((double *) ( /* dim=1 */ ((char *) (((double *) ( /* dim=0 */ (__pyx_v_self->Ca2_ss.data + __pyx_t_101 * __pyx_v_self->Ca2_ss.strides[0]) )) + __pyx_t_102)) )) = (__pyx_v_Ca2_ss + (__pyx_v_h * __pyx_v_dCa2_ss));
```

```
 1321:                 self.Ca2_JSR[x, y] = Ca2_JSR + h*dCa2_JSR
```

```
      if (unlikely(!__pyx_v_self->Ca2_JSR.memview)) {PyErr_SetString(PyExc_AttributeError,"Memoryview is not initialized");{__pyx_filename = __pyx_f[0]; __pyx_lineno = 1321; __pyx_clineno = __LINE__; goto __pyx_L1_error;}}
      __pyx_t_103 = __pyx_v_x;
      __pyx_t_104 = __pyx_v_y;
      *((double *) ( /* dim=1 */ ((char *) (((double *) ( /* dim=0 */ (__pyx_v_self->Ca2_JSR.data + __pyx_t_103 * __pyx_v_self->Ca2_JSR.strides[0]) )) + __pyx_t_104)) )) = (__pyx_v_Ca2_JSR + (__pyx_v_h * __pyx_v_dCa2_JSR));
```

```
 1322:                 self.Ca2_NSR[x, y] = Ca2_NSR + h*dCa2_NSR
```

```
      if (unlikely(!__pyx_v_self->Ca2_NSR.memview)) {PyErr_SetString(PyExc_AttributeError,"Memoryview is not initialized");{__pyx_filename = __pyx_f[0]; __pyx_lineno = 1322; __pyx_clineno = __LINE__; goto __pyx_L1_error;}}
      __pyx_t_105 = __pyx_v_x;
      __pyx_t_106 = __pyx_v_y;
      *((double *) ( /* dim=1 */ ((char *) (((double *) ( /* dim=0 */ (__pyx_v_self->Ca2_NSR.data + __pyx_t_105 * __pyx_v_self->Ca2_NSR.strides[0]) )) + __pyx_t_106)) )) = (__pyx_v_Ca2_NSR + (__pyx_v_h * __pyx_v_dCa2_NSR));
```

```
 1323:                 self.LTRPNCa[x, y] = LTRPNCa + h*dLTRPNCa
```

```
      if (unlikely(!__pyx_v_self->LTRPNCa.memview)) {PyErr_SetString(PyExc_AttributeError,"Memoryview is not initialized");{__pyx_filename = __pyx_f[0]; __pyx_lineno = 1323; __pyx_clineno = __LINE__; goto __pyx_L1_error;}}
      __pyx_t_107 = __pyx_v_x;
      __pyx_t_108 = __pyx_v_y;
      *((double *) ( /* dim=1 */ ((char *) (((double *) ( /* dim=0 */ (__pyx_v_self->LTRPNCa.data + __pyx_t_107 * __pyx_v_self->LTRPNCa.strides[0]) )) + __pyx_t_108)) )) = (__pyx_v_LTRPNCa + (__pyx_v_h * __pyx_v_dLTRPNCa));
```

```
 1324:                 self.HTRPNCa[x, y] = HTRPNCa + h*dHTRPNCa
```

```
      if (unlikely(!__pyx_v_self->HTRPNCa.memview)) {PyErr_SetString(PyExc_AttributeError,"Memoryview is not initialized");{__pyx_filename = __pyx_f[0]; __pyx_lineno = 1324; __pyx_clineno = __LINE__; goto __pyx_L1_error;}}
      __pyx_t_109 = __pyx_v_x;
      __pyx_t_110 = __pyx_v_y;
      *((double *) ( /* dim=1 */ ((char *) (((double *) ( /* dim=0 */ (__pyx_v_self->HTRPNCa.data + __pyx_t_109 * __pyx_v_self->HTRPNCa.strides[0]) )) + __pyx_t_110)) )) = (__pyx_v_HTRPNCa + (__pyx_v_h * __pyx_v_dHTRPNCa));
```

```
 1325:                 self.O[x, y] = O + h*dO
```

```
      if (unlikely(!__pyx_v_self->O.memview)) {PyErr_SetString(PyExc_AttributeError,"Memoryview is not initialized");{__pyx_filename = __pyx_f[0]; __pyx_lineno = 1325; __pyx_clineno = __LINE__; goto __pyx_L1_error;}}
      __pyx_t_111 = __pyx_v_x;
      __pyx_t_112 = __pyx_v_y;
      *((double *) ( /* dim=1 */ ((char *) (((double *) ( /* dim=0 */ (__pyx_v_self->O.data + __pyx_t_111 * __pyx_v_self->O.strides[0]) )) + __pyx_t_112)) )) = (__pyx_v_O + (__pyx_v_h * __pyx_v_dO));
```

```
 1326:                 self.C2[x, y] = C2 + h*dC2
```

```
      if (unlikely(!__pyx_v_self->C2.memview)) {PyErr_SetString(PyExc_AttributeError,"Memoryview is not initialized");{__pyx_filename = __pyx_f[0]; __pyx_lineno = 1326; __pyx_clineno = __LINE__; goto __pyx_L1_error;}}
      __pyx_t_113 = __pyx_v_x;
      __pyx_t_114 = __pyx_v_y;
      *((double *) ( /* dim=1 */ ((char *) (((double *) ( /* dim=0 */ (__pyx_v_self->C2.data + __pyx_t_113 * __pyx_v_self->C2.strides[0]) )) + __pyx_t_114)) )) = (__pyx_v_C2 + (__pyx_v_h * __pyx_v_dC2));
```

```
 1327:                 self.C3[x, y] = C3 + h*dC3
```

```
      if (unlikely(!__pyx_v_self->C3.memview)) {PyErr_SetString(PyExc_AttributeError,"Memoryview is not initialized");{__pyx_filename = __pyx_f[0]; __pyx_lineno = 1327; __pyx_clineno = __LINE__; goto __pyx_L1_error;}}
      __pyx_t_115 = __pyx_v_x;
      __pyx_t_116 = __pyx_v_y;
      *((double *) ( /* dim=1 */ ((char *) (((double *) ( /* dim=0 */ (__pyx_v_self->C3.data + __pyx_t_115 * __pyx_v_self->C3.strides[0]) )) + __pyx_t_116)) )) = (__pyx_v_C3 + (__pyx_v_h * __pyx_v_dC3));
```

```
 1328:                 self.C4[x, y] = C4 + h*dC4
```

```
      if (unlikely(!__pyx_v_self->C4.memview)) {PyErr_SetString(PyExc_AttributeError,"Memoryview is not initialized");{__pyx_filename = __pyx_f[0]; __pyx_lineno = 1328; __pyx_clineno = __LINE__; goto __pyx_L1_error;}}
      __pyx_t_117 = __pyx_v_x;
      __pyx_t_118 = __pyx_v_y;
      *((double *) ( /* dim=1 */ ((char *) (((double *) ( /* dim=0 */ (__pyx_v_self->C4.data + __pyx_t_117 * __pyx_v_self->C4.strides[0]) )) + __pyx_t_118)) )) = (__pyx_v_C4 + (__pyx_v_h * __pyx_v_dC4));
```

```
 1329:                 self.I1[x, y] = I1 + h*dI1
```

```
      if (unlikely(!__pyx_v_self->I1.memview)) {PyErr_SetString(PyExc_AttributeError,"Memoryview is not initialized");{__pyx_filename = __pyx_f[0]; __pyx_lineno = 1329; __pyx_clineno = __LINE__; goto __pyx_L1_error;}}
      __pyx_t_119 = __pyx_v_x;
      __pyx_t_120 = __pyx_v_y;
      *((double *) ( /* dim=1 */ ((char *) (((double *) ( /* dim=0 */ (__pyx_v_self->I1.data + __pyx_t_119 * __pyx_v_self->I1.strides[0]) )) + __pyx_t_120)) )) = (__pyx_v_I1 + (__pyx_v_h * __pyx_v_dI1));
```

```
 1330:                 self.I2[x, y] = I2 + h*dI2
```

```
      if (unlikely(!__pyx_v_self->I2.memview)) {PyErr_SetString(PyExc_AttributeError,"Memoryview is not initialized");{__pyx_filename = __pyx_f[0]; __pyx_lineno = 1330; __pyx_clineno = __LINE__; goto __pyx_L1_error;}}
      __pyx_t_121 = __pyx_v_x;
      __pyx_t_122 = __pyx_v_y;
      *((double *) ( /* dim=1 */ ((char *) (((double *) ( /* dim=0 */ (__pyx_v_self->I2.data + __pyx_t_121 * __pyx_v_self->I2.strides[0]) )) + __pyx_t_122)) )) = (__pyx_v_I2 + (__pyx_v_h * __pyx_v_dI2));
```

```
 1331:                 self.I3[x, y] = I3 + h*dI3
```

```
      if (unlikely(!__pyx_v_self->I3.memview)) {PyErr_SetString(PyExc_AttributeError,"Memoryview is not initialized");{__pyx_filename = __pyx_f[0]; __pyx_lineno = 1331; __pyx_clineno = __LINE__; goto __pyx_L1_error;}}
      __pyx_t_123 = __pyx_v_x;
      __pyx_t_124 = __pyx_v_y;
      *((double *) ( /* dim=1 */ ((char *) (((double *) ( /* dim=0 */ (__pyx_v_self->I3.data + __pyx_t_123 * __pyx_v_self->I3.strides[0]) )) + __pyx_t_124)) )) = (__pyx_v_I3 + (__pyx_v_h * __pyx_v_dI3));
```

```
 1332:                 self.Pc2[x, y] = Pc2 + h*dPc2
```

```
      if (unlikely(!__pyx_v_self->Pc2.memview)) {PyErr_SetString(PyExc_AttributeError,"Memoryview is not initialized");{__pyx_filename = __pyx_f[0]; __pyx_lineno = 1332; __pyx_clineno = __LINE__; goto __pyx_L1_error;}}
      __pyx_t_125 = __pyx_v_x;
      __pyx_t_126 = __pyx_v_y;
      *((double *) ( /* dim=1 */ ((char *) (((double *) ( /* dim=0 */ (__pyx_v_self->Pc2.data + __pyx_t_125 * __pyx_v_self->Pc2.strides[0]) )) + __pyx_t_126)) )) = (__pyx_v_Pc2 + (__pyx_v_h * __pyx_v_dPc2));
```

```
 1333:                 self.Po1[x, y] = Po1 + h*dPo1
```

```
      if (unlikely(!__pyx_v_self->Po1.memview)) {PyErr_SetString(PyExc_AttributeError,"Memoryview is not initialized");{__pyx_filename = __pyx_f[0]; __pyx_lineno = 1333; __pyx_clineno = __LINE__; goto __pyx_L1_error;}}
      __pyx_t_127 = __pyx_v_x;
      __pyx_t_128 = __pyx_v_y;
      *((double *) ( /* dim=1 */ ((char *) (((double *) ( /* dim=0 */ (__pyx_v_self->Po1.data + __pyx_t_127 * __pyx_v_self->Po1.strides[0]) )) + __pyx_t_128)) )) = (__pyx_v_Po1 + (__pyx_v_h * __pyx_v_dPo1));
```

```
 1334:                 self.Po2[x, y] = Po2 + h*dPo2
```

```
      if (unlikely(!__pyx_v_self->Po2.memview)) {PyErr_SetString(PyExc_AttributeError,"Memoryview is not initialized");{__pyx_filename = __pyx_f[0]; __pyx_lineno = 1334; __pyx_clineno = __LINE__; goto __pyx_L1_error;}}
      __pyx_t_129 = __pyx_v_x;
      __pyx_t_130 = __pyx_v_y;
      *((double *) ( /* dim=1 */ ((char *) (((double *) ( /* dim=0 */ (__pyx_v_self->Po2.data + __pyx_t_129 * __pyx_v_self->Po2.strides[0]) )) + __pyx_t_130)) )) = (__pyx_v_Po2 + (__pyx_v_h * __pyx_v_dPo2));
```

```
 1335:                 self.P_RyR[x, y] = P_RyR + h*dP_RyR
```

```
      if (unlikely(!__pyx_v_self->P_RyR.memview)) {PyErr_SetString(PyExc_AttributeError,"Memoryview is not initialized");{__pyx_filename = __pyx_f[0]; __pyx_lineno = 1335; __pyx_clineno = __LINE__; goto __pyx_L1_error;}}
      __pyx_t_131 = __pyx_v_x;
      __pyx_t_132 = __pyx_v_y;
      *((double *) ( /* dim=1 */ ((char *) (((double *) ( /* dim=0 */ (__pyx_v_self->P_RyR.data + __pyx_t_131 * __pyx_v_self->P_RyR.strides[0]) )) + __pyx_t_132)) )) = (__pyx_v_P_RyR + (__pyx_v_h * __pyx_v_dP_RyR));
```

```
 1336:                 self.C_Na2[x, y] = C_Na2 + h*dC_Na2
```

```
      if (unlikely(!__pyx_v_self->C_Na2.memview)) {PyErr_SetString(PyExc_AttributeError,"Memoryview is not initialized");{__pyx_filename = __pyx_f[0]; __pyx_lineno = 1336; __pyx_clineno = __LINE__; goto __pyx_L1_error;}}
      __pyx_t_133 = __pyx_v_x;
      __pyx_t_134 = __pyx_v_y;
      *((double *) ( /* dim=1 */ ((char *) (((double *) ( /* dim=0 */ (__pyx_v_self->C_Na2.data + __pyx_t_133 * __pyx_v_self->C_Na2.strides[0]) )) + __pyx_t_134)) )) = (__pyx_v_C_Na2 + (__pyx_v_h * __pyx_v_dC_Na2));
```

```
 1337:                 self.C_Na1[x, y] = C_Na1 + h*dC_Na1
```

```
      if (unlikely(!__pyx_v_self->C_Na1.memview)) {PyErr_SetString(PyExc_AttributeError,"Memoryview is not initialized");{__pyx_filename = __pyx_f[0]; __pyx_lineno = 1337; __pyx_clineno = __LINE__; goto __pyx_L1_error;}}
      __pyx_t_135 = __pyx_v_x;
      __pyx_t_136 = __pyx_v_y;
      *((double *) ( /* dim=1 */ ((char *) (((double *) ( /* dim=0 */ (__pyx_v_self->C_Na1.data + __pyx_t_135 * __pyx_v_self->C_Na1.strides[0]) )) + __pyx_t_136)) )) = (__pyx_v_C_Na1 + (__pyx_v_h * __pyx_v_dC_Na1));
```

```
 1338:                 self.O_Na[x, y] = O_Na + h*dO_Na
```

```
      if (unlikely(!__pyx_v_self->O_Na.memview)) {PyErr_SetString(PyExc_AttributeError,"Memoryview is not initialized");{__pyx_filename = __pyx_f[0]; __pyx_lineno = 1338; __pyx_clineno = __LINE__; goto __pyx_L1_error;}}
      __pyx_t_137 = __pyx_v_x;
      __pyx_t_138 = __pyx_v_y;
      *((double *) ( /* dim=1 */ ((char *) (((double *) ( /* dim=0 */ (__pyx_v_self->O_Na.data + __pyx_t_137 * __pyx_v_self->O_Na.strides[0]) )) + __pyx_t_138)) )) = (__pyx_v_O_Na + (__pyx_v_h * __pyx_v_dO_Na));
```

```
 1339:                 self.IF_Na[x, y] = IF_Na + h*dIF_Na
```

```
      if (unlikely(!__pyx_v_self->IF_Na.memview)) {PyErr_SetString(PyExc_AttributeError,"Memoryview is not initialized");{__pyx_filename = __pyx_f[0]; __pyx_lineno = 1339; __pyx_clineno = __LINE__; goto __pyx_L1_error;}}
      __pyx_t_139 = __pyx_v_x;
      __pyx_t_140 = __pyx_v_y;
      *((double *) ( /* dim=1 */ ((char *) (((double *) ( /* dim=0 */ (__pyx_v_self->IF_Na.data + __pyx_t_139 * __pyx_v_self->IF_Na.strides[0]) )) + __pyx_t_140)) )) = (__pyx_v_IF_Na + (__pyx_v_h * __pyx_v_dIF_Na));
```

```
 1340:                 self.I1_Na[x, y] = I1_Na + h*dI1_Na
```

```
      if (unlikely(!__pyx_v_self->I1_Na.memview)) {PyErr_SetString(PyExc_AttributeError,"Memoryview is not initialized");{__pyx_filename = __pyx_f[0]; __pyx_lineno = 1340; __pyx_clineno = __LINE__; goto __pyx_L1_error;}}
      __pyx_t_141 = __pyx_v_x;
      __pyx_t_142 = __pyx_v_y;
      *((double *) ( /* dim=1 */ ((char *) (((double *) ( /* dim=0 */ (__pyx_v_self->I1_Na.data + __pyx_t_141 * __pyx_v_self->I1_Na.strides[0]) )) + __pyx_t_142)) )) = (__pyx_v_I1_Na + (__pyx_v_h * __pyx_v_dI1_Na));
```

```
 1341:                 self.I2_Na[x, y] = I2_Na + h*dI2_Na
```

```
      if (unlikely(!__pyx_v_self->I2_Na.memview)) {PyErr_SetString(PyExc_AttributeError,"Memoryview is not initialized");{__pyx_filename = __pyx_f[0]; __pyx_lineno = 1341; __pyx_clineno = __LINE__; goto __pyx_L1_error;}}
      __pyx_t_143 = __pyx_v_x;
      __pyx_t_144 = __pyx_v_y;
      *((double *) ( /* dim=1 */ ((char *) (((double *) ( /* dim=0 */ (__pyx_v_self->I2_Na.data + __pyx_t_143 * __pyx_v_self->I2_Na.strides[0]) )) + __pyx_t_144)) )) = (__pyx_v_I2_Na + (__pyx_v_h * __pyx_v_dI2_Na));
```

```
 1342:                 self.IC_Na2[x, y] = IC_Na2 + h*dIC_Na2
```

```
      if (unlikely(!__pyx_v_self->IC_Na2.memview)) {PyErr_SetString(PyExc_AttributeError,"Memoryview is not initialized");{__pyx_filename = __pyx_f[0]; __pyx_lineno = 1342; __pyx_clineno = __LINE__; goto __pyx_L1_error;}}
      __pyx_t_145 = __pyx_v_x;
      __pyx_t_146 = __pyx_v_y;
      *((double *) ( /* dim=1 */ ((char *) (((double *) ( /* dim=0 */ (__pyx_v_self->IC_Na2.data + __pyx_t_145 * __pyx_v_self->IC_Na2.strides[0]) )) + __pyx_t_146)) )) = (__pyx_v_IC_Na2 + (__pyx_v_h * __pyx_v_dIC_Na2));
```

```
 1343:                 self.IC_Na3[x, y] = IC_Na3 + h*dIC_Na3
```

```
      if (unlikely(!__pyx_v_self->IC_Na3.memview)) {PyErr_SetString(PyExc_AttributeError,"Memoryview is not initialized");{__pyx_filename = __pyx_f[0]; __pyx_lineno = 1343; __pyx_clineno = __LINE__; goto __pyx_L1_error;}}
      __pyx_t_147 = __pyx_v_x;
      __pyx_t_148 = __pyx_v_y;
      *((double *) ( /* dim=1 */ ((char *) (((double *) ( /* dim=0 */ (__pyx_v_self->IC_Na3.data + __pyx_t_147 * __pyx_v_self->IC_Na3.strides[0]) )) + __pyx_t_148)) )) = (__pyx_v_IC_Na3 + (__pyx_v_h * __pyx_v_dIC_Na3));
```

```
 1344:                 self.Na_i[x, y] = Na_i + h*dNa_i
```

```
      if (unlikely(!__pyx_v_self->Na_i.memview)) {PyErr_SetString(PyExc_AttributeError,"Memoryview is not initialized");{__pyx_filename = __pyx_f[0]; __pyx_lineno = 1344; __pyx_clineno = __LINE__; goto __pyx_L1_error;}}
      __pyx_t_149 = __pyx_v_x;
      __pyx_t_150 = __pyx_v_y;
      *((double *) ( /* dim=1 */ ((char *) (((double *) ( /* dim=0 */ (__pyx_v_self->Na_i.data + __pyx_t_149 * __pyx_v_self->Na_i.strides[0]) )) + __pyx_t_150)) )) = (__pyx_v_Na_i + (__pyx_v_h * __pyx_v_dNa_i));
```

```
 1345:                 self.K_i[x, y] = K_i + h*dK_i
```

```
      if (unlikely(!__pyx_v_self->K_i.memview)) {PyErr_SetString(PyExc_AttributeError,"Memoryview is not initialized");{__pyx_filename = __pyx_f[0]; __pyx_lineno = 1345; __pyx_clineno = __LINE__; goto __pyx_L1_error;}}
      __pyx_t_151 = __pyx_v_x;
      __pyx_t_152 = __pyx_v_y;
      *((double *) ( /* dim=1 */ ((char *) (((double *) ( /* dim=0 */ (__pyx_v_self->K_i.data + __pyx_t_151 * __pyx_v_self->K_i.strides[0]) )) + __pyx_t_152)) )) = (__pyx_v_K_i + (__pyx_v_h * __pyx_v_dK_i));
```

```
 1346:                 self.a_tof[x, y] = a_tof + h*da_tof
```

```
      if (unlikely(!__pyx_v_self->a_tof.memview)) {PyErr_SetString(PyExc_AttributeError,"Memoryview is not initialized");{__pyx_filename = __pyx_f[0]; __pyx_lineno = 1346; __pyx_clineno = __LINE__; goto __pyx_L1_error;}}
      __pyx_t_153 = __pyx_v_x;
      __pyx_t_154 = __pyx_v_y;
      *((double *) ( /* dim=1 */ ((char *) (((double *) ( /* dim=0 */ (__pyx_v_self->a_tof.data + __pyx_t_153 * __pyx_v_self->a_tof.strides[0]) )) + __pyx_t_154)) )) = (__pyx_v_a_tof + (__pyx_v_h * __pyx_v_da_tof));
```

```
 1347:                 self.i_tof[x, y] = i_tof + h*di_tof
```

```
      if (unlikely(!__pyx_v_self->i_tof.memview)) {PyErr_SetString(PyExc_AttributeError,"Memoryview is not initialized");{__pyx_filename = __pyx_f[0]; __pyx_lineno = 1347; __pyx_clineno = __LINE__; goto __pyx_L1_error;}}
      __pyx_t_155 = __pyx_v_x;
      __pyx_t_156 = __pyx_v_y;
      *((double *) ( /* dim=1 */ ((char *) (((double *) ( /* dim=0 */ (__pyx_v_self->i_tof.data + __pyx_t_155 * __pyx_v_self->i_tof.strides[0]) )) + __pyx_t_156)) )) = (__pyx_v_i_tof + (__pyx_v_h * __pyx_v_di_tof));
```

```
 1348:                 self.n_Ks[x, y] = n_Ks + h*dn_Ks
```

```
      if (unlikely(!__pyx_v_self->n_Ks.memview)) {PyErr_SetString(PyExc_AttributeError,"Memoryview is not initialized");{__pyx_filename = __pyx_f[0]; __pyx_lineno = 1348; __pyx_clineno = __LINE__; goto __pyx_L1_error;}}
      __pyx_t_157 = __pyx_v_x;
      __pyx_t_158 = __pyx_v_y;
      *((double *) ( /* dim=1 */ ((char *) (((double *) ( /* dim=0 */ (__pyx_v_self->n_Ks.data + __pyx_t_157 * __pyx_v_self->n_Ks.strides[0]) )) + __pyx_t_158)) )) = (__pyx_v_n_Ks + (__pyx_v_h * __pyx_v_dn_Ks));
```

```
 1349:                 self.a_tos[x, y] = a_tos + h*da_tos
```

```
      if (unlikely(!__pyx_v_self->a_tos.memview)) {PyErr_SetString(PyExc_AttributeError,"Memoryview is not initialized");{__pyx_filename = __pyx_f[0]; __pyx_lineno = 1349; __pyx_clineno = __LINE__; goto __pyx_L1_error;}}
      __pyx_t_159 = __pyx_v_x;
      __pyx_t_160 = __pyx_v_y;
      *((double *) ( /* dim=1 */ ((char *) (((double *) ( /* dim=0 */ (__pyx_v_self->a_tos.data + __pyx_t_159 * __pyx_v_self->a_tos.strides[0]) )) + __pyx_t_160)) )) = (__pyx_v_a_tos + (__pyx_v_h * __pyx_v_da_tos));
```

```
 1350:                 self.i_tos[x, y] = i_tos + h*di_tos
```

```
      if (unlikely(!__pyx_v_self->i_tos.memview)) {PyErr_SetString(PyExc_AttributeError,"Memoryview is not initialized");{__pyx_filename = __pyx_f[0]; __pyx_lineno = 1350; __pyx_clineno = __LINE__; goto __pyx_L1_error;}}
      __pyx_t_161 = __pyx_v_x;
      __pyx_t_162 = __pyx_v_y;
      *((double *) ( /* dim=1 */ ((char *) (((double *) ( /* dim=0 */ (__pyx_v_self->i_tos.data + __pyx_t_161 * __pyx_v_self->i_tos.strides[0]) )) + __pyx_t_162)) )) = (__pyx_v_i_tos + (__pyx_v_h * __pyx_v_di_tos));
```

```
 1351:                 self.a_ur[x, y] = a_ur + h*da_ur
```

```
      if (unlikely(!__pyx_v_self->a_ur.memview)) {PyErr_SetString(PyExc_AttributeError,"Memoryview is not initialized");{__pyx_filename = __pyx_f[0]; __pyx_lineno = 1351; __pyx_clineno = __LINE__; goto __pyx_L1_error;}}
      __pyx_t_163 = __pyx_v_x;
      __pyx_t_164 = __pyx_v_y;
      *((double *) ( /* dim=1 */ ((char *) (((double *) ( /* dim=0 */ (__pyx_v_self->a_ur.data + __pyx_t_163 * __pyx_v_self->a_ur.strides[0]) )) + __pyx_t_164)) )) = (__pyx_v_a_ur + (__pyx_v_h * __pyx_v_da_ur));
```

```
 1352:                 self.i_ur[x, y] = i_ur + h*di_ur
```

```
      if (unlikely(!__pyx_v_self->i_ur.memview)) {PyErr_SetString(PyExc_AttributeError,"Memoryview is not initialized");{__pyx_filename = __pyx_f[0]; __pyx_lineno = 1352; __pyx_clineno = __LINE__; goto __pyx_L1_error;}}
      __pyx_t_165 = __pyx_v_x;
      __pyx_t_166 = __pyx_v_y;
      *((double *) ( /* dim=1 */ ((char *) (((double *) ( /* dim=0 */ (__pyx_v_self->i_ur.data + __pyx_t_165 * __pyx_v_self->i_ur.strides[0]) )) + __pyx_t_166)) )) = (__pyx_v_i_ur + (__pyx_v_h * __pyx_v_di_ur));
```

```
 1353:                 self.a_Kss[x, y] = a_Kss + h*da_Kss
```

```
      if (unlikely(!__pyx_v_self->a_Kss.memview)) {PyErr_SetString(PyExc_AttributeError,"Memoryview is not initialized");{__pyx_filename = __pyx_f[0]; __pyx_lineno = 1353; __pyx_clineno = __LINE__; goto __pyx_L1_error;}}
      __pyx_t_167 = __pyx_v_x;
      __pyx_t_168 = __pyx_v_y;
      *((double *) ( /* dim=1 */ ((char *) (((double *) ( /* dim=0 */ (__pyx_v_self->a_Kss.data + __pyx_t_167 * __pyx_v_self->a_Kss.strides[0]) )) + __pyx_t_168)) )) = (__pyx_v_a_Kss + (__pyx_v_h * __pyx_v_da_Kss));
```

```
 1354:                 self.C_K1[x, y] = C_K1 + h*dC_K1
```

```
      if (unlikely(!__pyx_v_self->C_K1.memview)) {PyErr_SetString(PyExc_AttributeError,"Memoryview is not initialized");{__pyx_filename = __pyx_f[0]; __pyx_lineno = 1354; __pyx_clineno = __LINE__; goto __pyx_L1_error;}}
      __pyx_t_169 = __pyx_v_x;
      __pyx_t_170 = __pyx_v_y;
      *((double *) ( /* dim=1 */ ((char *) (((double *) ( /* dim=0 */ (__pyx_v_self->C_K1.data + __pyx_t_169 * __pyx_v_self->C_K1.strides[0]) )) + __pyx_t_170)) )) = (__pyx_v_C_K1 + (__pyx_v_h * __pyx_v_dC_K1));
```

```
 1355:                 self.C_K2[x, y] = C_K2 + h*dC_K2
```

```
      if (unlikely(!__pyx_v_self->C_K2.memview)) {PyErr_SetString(PyExc_AttributeError,"Memoryview is not initialized");{__pyx_filename = __pyx_f[0]; __pyx_lineno = 1355; __pyx_clineno = __LINE__; goto __pyx_L1_error;}}
      __pyx_t_171 = __pyx_v_x;
      __pyx_t_172 = __pyx_v_y;
      *((double *) ( /* dim=1 */ ((char *) (((double *) ( /* dim=0 */ (__pyx_v_self->C_K2.data + __pyx_t_171 * __pyx_v_self->C_K2.strides[0]) )) + __pyx_t_172)) )) = (__pyx_v_C_K2 + (__pyx_v_h * __pyx_v_dC_K2));
```

```
 1356:                 self.O_K[x, y] = O_K + h*dO_K
```

```
      if (unlikely(!__pyx_v_self->O_K.memview)) {PyErr_SetString(PyExc_AttributeError,"Memoryview is not initialized");{__pyx_filename = __pyx_f[0]; __pyx_lineno = 1356; __pyx_clineno = __LINE__; goto __pyx_L1_error;}}
      __pyx_t_173 = __pyx_v_x;
      __pyx_t_174 = __pyx_v_y;
      *((double *) ( /* dim=1 */ ((char *) (((double *) ( /* dim=0 */ (__pyx_v_self->O_K.data + __pyx_t_173 * __pyx_v_self->O_K.strides[0]) )) + __pyx_t_174)) )) = (__pyx_v_O_K + (__pyx_v_h * __pyx_v_dO_K));
```

```
 1357:                 self.I_K[x, y] = I_K + h*dI_K
```

```
      if (unlikely(!__pyx_v_self->I_K.memview)) {PyErr_SetString(PyExc_AttributeError,"Memoryview is not initialized");{__pyx_filename = __pyx_f[0]; __pyx_lineno = 1357; __pyx_clineno = __LINE__; goto __pyx_L1_error;}}
      __pyx_t_175 = __pyx_v_x;
      __pyx_t_176 = __pyx_v_y;
      *((double *) ( /* dim=1 */ ((char *) (((double *) ( /* dim=0 */ (__pyx_v_self->I_K.data + __pyx_t_175 * __pyx_v_self->I_K.strides[0]) )) + __pyx_t_176)) )) = (__pyx_v_I_K + (__pyx_v_h * __pyx_v_dI_K));
    }
  }
```

```
 1358:
```

```
 1359:         self.t += h
```

```
  __pyx_v_self->t = (__pyx_v_self->t + __pyx_v_h);
```

```
 1360:         self.V[0,:]  = self.V[1,:]
```

```
  __pyx_t_12 = __Pyx_PyObject_GetAttrStr(((PyObject *)__pyx_v_self), __pyx_n_s_V); if (unlikely(!__pyx_t_12)) {__pyx_filename = __pyx_f[0]; __pyx_lineno = 1360; __pyx_clineno = __LINE__; goto __pyx_L1_error;}
  __Pyx_GOTREF(__pyx_t_12);
/* … */
  __pyx_slice__3 = PySlice_New(Py_None, Py_None, Py_None); if (unlikely(!__pyx_slice__3)) {__pyx_filename = __pyx_f[0]; __pyx_lineno = 1360; __pyx_clineno = __LINE__; goto __pyx_L1_error;}
  __Pyx_GOTREF(__pyx_slice__3);
  __Pyx_GIVEREF(__pyx_slice__3);
  __pyx_t_6 = PyObject_GetItem(__pyx_t_12, __pyx_tuple__4); if (unlikely(__pyx_t_6 == NULL)) {__pyx_filename = __pyx_f[0]; __pyx_lineno = 1360; __pyx_clineno = __LINE__; goto __pyx_L1_error;};
  __Pyx_GOTREF(__pyx_t_6);
  __Pyx_DECREF(__pyx_t_12); __pyx_t_12 = 0;
  __pyx_t_12 = __Pyx_PyObject_GetAttrStr(((PyObject *)__pyx_v_self), __pyx_n_s_V); if (unlikely(!__pyx_t_12)) {__pyx_filename = __pyx_f[0]; __pyx_lineno = 1360; __pyx_clineno = __LINE__; goto __pyx_L1_error;}
  __Pyx_GOTREF(__pyx_t_12);
  __pyx_tuple__4 = PyTuple_Pack(2, __pyx_int_1, __pyx_slice__3); if (unlikely(!__pyx_tuple__4)) {__pyx_filename = __pyx_f[0]; __pyx_lineno = 1360; __pyx_clineno = __LINE__; goto __pyx_L1_error;}
  __Pyx_GOTREF(__pyx_tuple__4);
  __Pyx_GIVEREF(__pyx_tuple__4);
  __pyx_slice__5 = PySlice_New(Py_None, Py_None, Py_None); if (unlikely(!__pyx_slice__5)) {__pyx_filename = __pyx_f[0]; __pyx_lineno = 1360; __pyx_clineno = __LINE__; goto __pyx_L1_error;}
  __Pyx_GOTREF(__pyx_slice__5);
  __Pyx_GIVEREF(__pyx_slice__5);
  if (unlikely(PyObject_SetItem(__pyx_t_12, __pyx_tuple__6, __pyx_t_6) < 0)) {__pyx_filename = __pyx_f[0]; __pyx_lineno = 1360; __pyx_clineno = __LINE__; goto __pyx_L1_error;}
  __Pyx_DECREF(__pyx_t_12); __pyx_t_12 = 0;
  __Pyx_DECREF(__pyx_t_6); __pyx_t_6 = 0;
  __pyx_tuple__6 = PyTuple_Pack(2, __pyx_int_0, __pyx_slice__5); if (unlikely(!__pyx_tuple__6)) {__pyx_filename = __pyx_f[0]; __pyx_lineno = 1360; __pyx_clineno = __LINE__; goto __pyx_L1_error;}
  __Pyx_GOTREF(__pyx_tuple__6);
  __Pyx_GIVEREF(__pyx_tuple__6);
```

```
 1361:         self.V[nx-1,:] = self.V[nx-2,:]
```

```
  __pyx_t_6 = __Pyx_PyObject_GetAttrStr(((PyObject *)__pyx_v_self), __pyx_n_s_V); if (unlikely(!__pyx_t_6)) {__pyx_filename = __pyx_f[0]; __pyx_lineno = 1361; __pyx_clineno = __LINE__; goto __pyx_L1_error;}
  __Pyx_GOTREF(__pyx_t_6);
  __pyx_t_12 = __Pyx_PyInt_From_long((__pyx_v_nx - 2)); if (unlikely(!__pyx_t_12)) {__pyx_filename = __pyx_f[0]; __pyx_lineno = 1361; __pyx_clineno = __LINE__; goto __pyx_L1_error;}
  __Pyx_GOTREF(__pyx_t_12);
  __pyx_t_1 = PyTuple_New(2); if (unlikely(!__pyx_t_1)) {__pyx_filename = __pyx_f[0]; __pyx_lineno = 1361; __pyx_clineno = __LINE__; goto __pyx_L1_error;}
  __Pyx_GOTREF(__pyx_t_1);
  PyTuple_SET_ITEM(__pyx_t_1, 0, __pyx_t_12);
  __Pyx_GIVEREF(__pyx_t_12);
  __Pyx_INCREF(__pyx_slice__7);
  PyTuple_SET_ITEM(__pyx_t_1, 1, __pyx_slice__7);
  __Pyx_GIVEREF(__pyx_slice__7);
  __pyx_t_12 = 0;
  __pyx_t_12 = PyObject_GetItem(__pyx_t_6, __pyx_t_1); if (unlikely(__pyx_t_12 == NULL)) {__pyx_filename = __pyx_f[0]; __pyx_lineno = 1361; __pyx_clineno = __LINE__; goto __pyx_L1_error;};
  __Pyx_GOTREF(__pyx_t_12);
  __Pyx_DECREF(__pyx_t_6); __pyx_t_6 = 0;
  __Pyx_DECREF(__pyx_t_1); __pyx_t_1 = 0;
  __pyx_t_1 = __Pyx_PyObject_GetAttrStr(((PyObject *)__pyx_v_self), __pyx_n_s_V); if (unlikely(!__pyx_t_1)) {__pyx_filename = __pyx_f[0]; __pyx_lineno = 1361; __pyx_clineno = __LINE__; goto __pyx_L1_error;}
  __Pyx_GOTREF(__pyx_t_1);
  __pyx_t_6 = __Pyx_PyInt_From_long((__pyx_v_nx - 1)); if (unlikely(!__pyx_t_6)) {__pyx_filename = __pyx_f[0]; __pyx_lineno = 1361; __pyx_clineno = __LINE__; goto __pyx_L1_error;}
  __Pyx_GOTREF(__pyx_t_6);
/* … */
  __pyx_slice__7 = PySlice_New(Py_None, Py_None, Py_None); if (unlikely(!__pyx_slice__7)) {__pyx_filename = __pyx_f[0]; __pyx_lineno = 1361; __pyx_clineno = __LINE__; goto __pyx_L1_error;}
  __Pyx_GOTREF(__pyx_slice__7);
  __Pyx_GIVEREF(__pyx_slice__7);
  __pyx_t_5 = PyTuple_New(2); if (unlikely(!__pyx_t_5)) {__pyx_filename = __pyx_f[0]; __pyx_lineno = 1361; __pyx_clineno = __LINE__; goto __pyx_L1_error;}
  __Pyx_GOTREF(__pyx_t_5);
  PyTuple_SET_ITEM(__pyx_t_5, 0, __pyx_t_6);
  __Pyx_GIVEREF(__pyx_t_6);
  __Pyx_INCREF(__pyx_slice__8);
  PyTuple_SET_ITEM(__pyx_t_5, 1, __pyx_slice__8);
  __Pyx_GIVEREF(__pyx_slice__8);
  __pyx_t_6 = 0;
  if (unlikely(PyObject_SetItem(__pyx_t_1, __pyx_t_5, __pyx_t_12) < 0)) {__pyx_filename = __pyx_f[0]; __pyx_lineno = 1361; __pyx_clineno = __LINE__; goto __pyx_L1_error;}
  __Pyx_DECREF(__pyx_t_1); __pyx_t_1 = 0;
  __Pyx_DECREF(__pyx_t_5); __pyx_t_5 = 0;
  __Pyx_DECREF(__pyx_t_12); __pyx_t_12 = 0;
  __pyx_slice__8 = PySlice_New(Py_None, Py_None, Py_None); if (unlikely(!__pyx_slice__8)) {__pyx_filename = __pyx_f[0]; __pyx_lineno = 1361; __pyx_clineno = __LINE__; goto __pyx_L1_error;}
  __Pyx_GOTREF(__pyx_slice__8);
  __Pyx_GIVEREF(__pyx_slice__8);
```

```
 1362:         self.V[:,0]  = self.V[:,1]
```

```
  __pyx_t_12 = __Pyx_PyObject_GetAttrStr(((PyObject *)__pyx_v_self), __pyx_n_s_V); if (unlikely(!__pyx_t_12)) {__pyx_filename = __pyx_f[0]; __pyx_lineno = 1362; __pyx_clineno = __LINE__; goto __pyx_L1_error;}
  __Pyx_GOTREF(__pyx_t_12);
/* … */
  __pyx_slice__9 = PySlice_New(Py_None, Py_None, Py_None); if (unlikely(!__pyx_slice__9)) {__pyx_filename = __pyx_f[0]; __pyx_lineno = 1362; __pyx_clineno = __LINE__; goto __pyx_L1_error;}
  __Pyx_GOTREF(__pyx_slice__9);
  __Pyx_GIVEREF(__pyx_slice__9);
  __pyx_t_5 = PyObject_GetItem(__pyx_t_12, __pyx_tuple__10); if (unlikely(__pyx_t_5 == NULL)) {__pyx_filename = __pyx_f[0]; __pyx_lineno = 1362; __pyx_clineno = __LINE__; goto __pyx_L1_error;};
  __Pyx_GOTREF(__pyx_t_5);
  __Pyx_DECREF(__pyx_t_12); __pyx_t_12 = 0;
  __pyx_t_12 = __Pyx_PyObject_GetAttrStr(((PyObject *)__pyx_v_self), __pyx_n_s_V); if (unlikely(!__pyx_t_12)) {__pyx_filename = __pyx_f[0]; __pyx_lineno = 1362; __pyx_clineno = __LINE__; goto __pyx_L1_error;}
  __Pyx_GOTREF(__pyx_t_12);
  __pyx_tuple__10 = PyTuple_Pack(2, __pyx_slice__9, __pyx_int_1); if (unlikely(!__pyx_tuple__10)) {__pyx_filename = __pyx_f[0]; __pyx_lineno = 1362; __pyx_clineno = __LINE__; goto __pyx_L1_error;}
  __Pyx_GOTREF(__pyx_tuple__10);
  __Pyx_GIVEREF(__pyx_tuple__10);
  __pyx_slice__11 = PySlice_New(Py_None, Py_None, Py_None); if (unlikely(!__pyx_slice__11)) {__pyx_filename = __pyx_f[0]; __pyx_lineno = 1362; __pyx_clineno = __LINE__; goto __pyx_L1_error;}
  __Pyx_GOTREF(__pyx_slice__11);
  __Pyx_GIVEREF(__pyx_slice__11);
  if (unlikely(PyObject_SetItem(__pyx_t_12, __pyx_tuple__12, __pyx_t_5) < 0)) {__pyx_filename = __pyx_f[0]; __pyx_lineno = 1362; __pyx_clineno = __LINE__; goto __pyx_L1_error;}
  __Pyx_DECREF(__pyx_t_12); __pyx_t_12 = 0;
  __Pyx_DECREF(__pyx_t_5); __pyx_t_5 = 0;
  __pyx_tuple__12 = PyTuple_Pack(2, __pyx_slice__11, __pyx_int_0); if (unlikely(!__pyx_tuple__12)) {__pyx_filename = __pyx_f[0]; __pyx_lineno = 1362; __pyx_clineno = __LINE__; goto __pyx_L1_error;}
  __Pyx_GOTREF(__pyx_tuple__12);
  __Pyx_GIVEREF(__pyx_tuple__12);
```

```
 1363:         self.V[:,ny-1] = self.V[:,ny-2]
```

```
  __pyx_t_5 = __Pyx_PyObject_GetAttrStr(((PyObject *)__pyx_v_self), __pyx_n_s_V); if (unlikely(!__pyx_t_5)) {__pyx_filename = __pyx_f[0]; __pyx_lineno = 1363; __pyx_clineno = __LINE__; goto __pyx_L1_error;}
  __Pyx_GOTREF(__pyx_t_5);
  __pyx_t_12 = __Pyx_PyInt_From_long((__pyx_v_ny - 2)); if (unlikely(!__pyx_t_12)) {__pyx_filename = __pyx_f[0]; __pyx_lineno = 1363; __pyx_clineno = __LINE__; goto __pyx_L1_error;}
  __Pyx_GOTREF(__pyx_t_12);
  __pyx_t_1 = PyTuple_New(2); if (unlikely(!__pyx_t_1)) {__pyx_filename = __pyx_f[0]; __pyx_lineno = 1363; __pyx_clineno = __LINE__; goto __pyx_L1_error;}
  __Pyx_GOTREF(__pyx_t_1);
  __Pyx_INCREF(__pyx_slice__13);
  PyTuple_SET_ITEM(__pyx_t_1, 0, __pyx_slice__13);
  __Pyx_GIVEREF(__pyx_slice__13);
  PyTuple_SET_ITEM(__pyx_t_1, 1, __pyx_t_12);
  __Pyx_GIVEREF(__pyx_t_12);
  __pyx_t_12 = 0;
  __pyx_t_12 = PyObject_GetItem(__pyx_t_5, __pyx_t_1); if (unlikely(__pyx_t_12 == NULL)) {__pyx_filename = __pyx_f[0]; __pyx_lineno = 1363; __pyx_clineno = __LINE__; goto __pyx_L1_error;};
  __Pyx_GOTREF(__pyx_t_12);
  __Pyx_DECREF(__pyx_t_5); __pyx_t_5 = 0;
  __Pyx_DECREF(__pyx_t_1); __pyx_t_1 = 0;
  __pyx_t_1 = __Pyx_PyObject_GetAttrStr(((PyObject *)__pyx_v_self), __pyx_n_s_V); if (unlikely(!__pyx_t_1)) {__pyx_filename = __pyx_f[0]; __pyx_lineno = 1363; __pyx_clineno = __LINE__; goto __pyx_L1_error;}
  __Pyx_GOTREF(__pyx_t_1);
/* … */
  __pyx_slice__13 = PySlice_New(Py_None, Py_None, Py_None); if (unlikely(!__pyx_slice__13)) {__pyx_filename = __pyx_f[0]; __pyx_lineno = 1363; __pyx_clineno = __LINE__; goto __pyx_L1_error;}
  __Pyx_GOTREF(__pyx_slice__13);
  __Pyx_GIVEREF(__pyx_slice__13);
  __pyx_t_5 = __Pyx_PyInt_From_long((__pyx_v_ny - 1)); if (unlikely(!__pyx_t_5)) {__pyx_filename = __pyx_f[0]; __pyx_lineno = 1363; __pyx_clineno = __LINE__; goto __pyx_L1_error;}
  __Pyx_GOTREF(__pyx_t_5);
  __pyx_t_6 = PyTuple_New(2); if (unlikely(!__pyx_t_6)) {__pyx_filename = __pyx_f[0]; __pyx_lineno = 1363; __pyx_clineno = __LINE__; goto __pyx_L1_error;}
  __Pyx_GOTREF(__pyx_t_6);
  __Pyx_INCREF(__pyx_slice__14);
  PyTuple_SET_ITEM(__pyx_t_6, 0, __pyx_slice__14);
  __Pyx_GIVEREF(__pyx_slice__14);
  PyTuple_SET_ITEM(__pyx_t_6, 1, __pyx_t_5);
  __Pyx_GIVEREF(__pyx_t_5);
  __pyx_t_5 = 0;
  if (unlikely(PyObject_SetItem(__pyx_t_1, __pyx_t_6, __pyx_t_12) < 0)) {__pyx_filename = __pyx_f[0]; __pyx_lineno = 1363; __pyx_clineno = __LINE__; goto __pyx_L1_error;}
  __Pyx_DECREF(__pyx_t_1); __pyx_t_1 = 0;
  __Pyx_DECREF(__pyx_t_6); __pyx_t_6 = 0;
  __Pyx_DECREF(__pyx_t_12); __pyx_t_12 = 0;
  __pyx_slice__14 = PySlice_New(Py_None, Py_None, Py_None); if (unlikely(!__pyx_slice__14)) {__pyx_filename = __pyx_f[0]; __pyx_lineno = 1363; __pyx_clineno = __LINE__; goto __pyx_L1_error;}
  __Pyx_GOTREF(__pyx_slice__14);
  __Pyx_GIVEREF(__pyx_slice__14);
```

```
 1364:
```

```
 1365:
```

```
 1366:
```

```
 1367:
```

```
 1368:
```

```
 1369:
```

```
 1370:
```

```
 1371:
```

```
 1372:
```

```
 1373:
```

```
 1374:
```

```
 1375:
```

```
 1376:
```

```
 1377:
```

```
 1378:
```

```
 1379:
```

```
 1380:
```

```
 1381:
```

```
 1382:     cdef double f_V(self, double I_CaL, double I_pCa, double I_NaCa, double I_Cab, double I_Na, double I_Nab, double I_NaK, double I_Ktof, double I_Ktos, double I_K1, double I_Ks, double I_Kur, double I_Kss, double I_Kr, double I_ClCa, double I_stim):
```

```
static double __pyx_f_5model_10Bondarenko_f_V(struct __pyx_obj_5model_Bondarenko *__pyx_v_self, double __pyx_v_I_CaL, double __pyx_v_I_pCa, double __pyx_v_I_NaCa, double __pyx_v_I_Cab, double __pyx_v_I_Na, double __pyx_v_I_Nab, double __pyx_v_I_NaK, double __pyx_v_I_Ktof, double __pyx_v_I_Ktos, double __pyx_v_I_K1, double __pyx_v_I_Ks, double __pyx_v_I_Kur, double __pyx_v_I_Kss, double __pyx_v_I_Kr, double __pyx_v_I_ClCa, double __pyx_v_I_stim) {
  double __pyx_r;
  __Pyx_RefNannyDeclarations
  __Pyx_RefNannySetupContext("f_V", 0);
/* … */
  /* function exit code */
  __pyx_L0:;
  __Pyx_RefNannyFinishContext();
  return __pyx_r;
}
```

```
 1383:
```

```
 1384:             return -(I_CaL + I_pCa + I_NaCa + I_Cab + I_Na + I_Nab + I_NaK + I_Ktof + I_Ktos + I_K1 + I_Ks + I_Kur + I_Kss + I_Kr + I_ClCa - I_stim)/self.C_m
```

```
  __pyx_r = ((-(((((((((((((((__pyx_v_I_CaL + __pyx_v_I_pCa) + __pyx_v_I_NaCa) + __pyx_v_I_Cab) + __pyx_v_I_Na) + __pyx_v_I_Nab) + __pyx_v_I_NaK) + __pyx_v_I_Ktof) + __pyx_v_I_Ktos) + __pyx_v_I_K1) + __pyx_v_I_Ks) + __pyx_v_I_Kur) + __pyx_v_I_Kss) + __pyx_v_I_Kr) + __pyx_v_I_ClCa) - __pyx_v_I_stim)) / __pyx_v_self->C_m);
  goto __pyx_L0;
```

```
 1385:
```

```
 1386:
```

```
 1387:     cdef double f_Ca2_i (self,double B_i,double  J_leak,double  J_xfer,double  J_up,double  J_trpn,double  I_Cab,double  I_NaCa,double  I_pCa):
```

```
static double __pyx_f_5model_10Bondarenko_f_Ca2_i(struct __pyx_obj_5model_Bondarenko *__pyx_v_self, double __pyx_v_B_i, double __pyx_v_J_leak, double __pyx_v_J_xfer, double __pyx_v_J_up, double __pyx_v_J_trpn, double __pyx_v_I_Cab, double __pyx_v_I_NaCa, double __pyx_v_I_pCa) {
  double __pyx_r;
  __Pyx_RefNannyDeclarations
  __Pyx_RefNannySetupContext("f_Ca2_i", 0);
/* … */
  /* function exit code */
  __pyx_L0:;
  __Pyx_RefNannyFinishContext();
  return __pyx_r;
}
```

```
 1388:
```

```
 1389:             return B_i*(J_leak + J_xfer - J_up - J_trpn - (I_Cab - 2*I_NaCa + I_pCa)*((self.A_cap*self.C_m)/(2*self.V_myo*self.F)))
```

```
  __pyx_r = (__pyx_v_B_i * ((((__pyx_v_J_leak + __pyx_v_J_xfer) - __pyx_v_J_up) - __pyx_v_J_trpn) - (((__pyx_v_I_Cab - (2.0 * __pyx_v_I_NaCa)) + __pyx_v_I_pCa) * ((__pyx_v_self->A_cap * __pyx_v_self->C_m) / ((2.0 * __pyx_v_self->V_myo) * __pyx_v_self->F)))));
  goto __pyx_L0;
```

```
 1390:
```

```
 1391:
```

```
 1392:     cdef double f_Ca2_ss(self,double B_ss, double J_rel, double J_xfer, double I_CaL):
```

```
static double __pyx_f_5model_10Bondarenko_f_Ca2_ss(struct __pyx_obj_5model_Bondarenko *__pyx_v_self, double __pyx_v_B_ss, double __pyx_v_J_rel, double __pyx_v_J_xfer, double __pyx_v_I_CaL) {
  double __pyx_r;
  __Pyx_RefNannyDeclarations
  __Pyx_RefNannySetupContext("f_Ca2_ss", 0);
/* … */
  /* function exit code */
  __pyx_L0:;
  __Pyx_RefNannyFinishContext();
  return __pyx_r;
}
```

```
 1393:
```

```
 1394:             return B_ss*(J_rel*(self.V_JSR/self.V_ss) - J_xfer*(self.V_myo/self.V_ss) - I_CaL*((self.A_cap*self.C_m)/(2*self.V_ss*self.F)))
```

```
  __pyx_r = (__pyx_v_B_ss * (((__pyx_v_J_rel * (__pyx_v_self->V_JSR / __pyx_v_self->V_ss)) - (__pyx_v_J_xfer * (__pyx_v_self->V_myo / __pyx_v_self->V_ss))) - (__pyx_v_I_CaL * ((__pyx_v_self->A_cap * __pyx_v_self->C_m) / ((2.0 * __pyx_v_self->V_ss) * __pyx_v_self->F)))));
  goto __pyx_L0;
```

```
 1395:
```

```
 1396:
```

```
 1397:     cdef double f_Ca2_JSR (self,double B_JSR, double J_tr, double J_rel):
```

```
static double __pyx_f_5model_10Bondarenko_f_Ca2_JSR(CYTHON_UNUSED struct __pyx_obj_5model_Bondarenko *__pyx_v_self, double __pyx_v_B_JSR, double __pyx_v_J_tr, double __pyx_v_J_rel) {
  double __pyx_r;
  __Pyx_RefNannyDeclarations
  __Pyx_RefNannySetupContext("f_Ca2_JSR", 0);
/* … */
  /* function exit code */
  __pyx_L0:;
  __Pyx_RefNannyFinishContext();
  return __pyx_r;
}
```

```
 1398:
```

```
 1399:             return B_JSR*(J_tr - J_rel)
```

```
  __pyx_r = (__pyx_v_B_JSR * (__pyx_v_J_tr - __pyx_v_J_rel));
  goto __pyx_L0;
```

```
 1400:
```

```
 1401:
```

```
 1402:     cdef double f_Ca2_NSR(self,double J_up, double J_leak, double J_tr):
```

```
static double __pyx_f_5model_10Bondarenko_f_Ca2_NSR(struct __pyx_obj_5model_Bondarenko *__pyx_v_self, double __pyx_v_J_up, double __pyx_v_J_leak, double __pyx_v_J_tr) {
  double __pyx_r;
  __Pyx_RefNannyDeclarations
  __Pyx_RefNannySetupContext("f_Ca2_NSR", 0);
/* … */
  /* function exit code */
  __pyx_L0:;
  __Pyx_RefNannyFinishContext();
  return __pyx_r;
}
```

```
 1403:
```

```
 1404:             return (J_up - J_leak)*(self.V_myo/self.V_NSR) - J_tr*(self.V_JSR/self.V_NSR)
```

```
  __pyx_r = (((__pyx_v_J_up - __pyx_v_J_leak) * (__pyx_v_self->V_myo / __pyx_v_self->V_NSR)) - (__pyx_v_J_tr * (__pyx_v_self->V_JSR / __pyx_v_self->V_NSR)));
  goto __pyx_L0;
```

```
 1405:
```

```
 1406:
```

```
 1407:     cdef double f_P_RyR (self,double P_RyR, double I_CaL, double V):
```

```
static double __pyx_f_5model_10Bondarenko_f_P_RyR(struct __pyx_obj_5model_Bondarenko *__pyx_v_self, double __pyx_v_P_RyR, double __pyx_v_I_CaL, double __pyx_v_V) {
  double __pyx_r;
  __Pyx_RefNannyDeclarations
  __Pyx_RefNannySetupContext("f_P_RyR", 0);
/* … */
  /* function exit code */
  __pyx_L0:;
  __Pyx_RefNannyFinishContext();
  return __pyx_r;
}
```

```
 1408:
```

```
 1409:             return -.04*P_RyR - 0.1*(I_CaL/self.I_CaLmax)*exp(-(V - 5.)**2/648.)
```

```
  __pyx_r = ((-.04 * __pyx_v_P_RyR) - ((0.1 * (__pyx_v_I_CaL / __pyx_v_self->I_CaLmax)) * exp(((-pow((__pyx_v_V - 5.), 2.0)) / 648.))));
  goto __pyx_L0;
```

```
 1410:
```

```
 1411:     cdef double f_LTRPNCa(self,double Ca2_i, double LTRPNCa):
```

```
static double __pyx_f_5model_10Bondarenko_f_LTRPNCa(struct __pyx_obj_5model_Bondarenko *__pyx_v_self, double __pyx_v_Ca2_i, double __pyx_v_LTRPNCa) {
  double __pyx_r;
  __Pyx_RefNannyDeclarations
  __Pyx_RefNannySetupContext("f_LTRPNCa", 0);
/* … */
  /* function exit code */
  __pyx_L0:;
  __Pyx_RefNannyFinishContext();
  return __pyx_r;
}
```

```
 1412:
```

```
 1413:             return self.k_p_ltrpn*Ca2_i*(self.LTRPN_tot - LTRPNCa) -self. k_n_ltrpn*LTRPNCa
```

```
  __pyx_r = (((__pyx_v_self->k_p_ltrpn * __pyx_v_Ca2_i) * (__pyx_v_self->LTRPN_tot - __pyx_v_LTRPNCa)) - (__pyx_v_self->k_n_ltrpn * __pyx_v_LTRPNCa));
  goto __pyx_L0;
```

```
 1414:
```

```
 1415:
```

```
 1416:     cdef double f_HTRPNCa(self,double Ca2_i, double HTRPNCa):
```

```
static double __pyx_f_5model_10Bondarenko_f_HTRPNCa(struct __pyx_obj_5model_Bondarenko *__pyx_v_self, double __pyx_v_Ca2_i, double __pyx_v_HTRPNCa) {
  double __pyx_r;
  __Pyx_RefNannyDeclarations
  __Pyx_RefNannySetupContext("f_HTRPNCa", 0);
/* … */
  /* function exit code */
  __pyx_L0:;
  __Pyx_RefNannyFinishContext();
  return __pyx_r;
}
```

```
 1417:
```

```
 1418:             return self.k_p_htrpn*Ca2_i*(self.HTRPN_tot - HTRPNCa) - self.k_n_htrpn*HTRPNCa
```

```
  __pyx_r = (((__pyx_v_self->k_p_htrpn * __pyx_v_Ca2_i) * (__pyx_v_self->HTRPN_tot - __pyx_v_HTRPNCa)) - (__pyx_v_self->k_n_htrpn * __pyx_v_HTRPNCa));
  goto __pyx_L0;
```

```
 1419:
```

```
 1420:
```

```
 1421:     cdef double f_Po1(self,double Ca2_ss, double Pc1, double Po1, double Po2, double Pc2):
```

```
static double __pyx_f_5model_10Bondarenko_f_Po1(struct __pyx_obj_5model_Bondarenko *__pyx_v_self, double __pyx_v_Ca2_ss, double __pyx_v_Pc1, double __pyx_v_Po1, double __pyx_v_Po2, double __pyx_v_Pc2) {
  double __pyx_r;
  __Pyx_RefNannyDeclarations
  __Pyx_RefNannySetupContext("f_Po1", 0);
/* … */
  /* function exit code */
  __pyx_L0:;
  __Pyx_RefNannyFinishContext();
  return __pyx_r;
}
```

```
 1422:
```

```
 1423:             return self.k_p_a*Ca2_ss**self.n*Pc1 - self.k_n_a*Po1 - self.k_p_b*Ca2_ss**self.m*Po1 + self.k_n_b*Po2 - self.k_p_c*Po1 + self.k_n_c*Pc2
```

```
  __pyx_r = (((((((__pyx_v_self->k_p_a * pow(__pyx_v_Ca2_ss, __pyx_v_self->n)) * __pyx_v_Pc1) - (__pyx_v_self->k_n_a * __pyx_v_Po1)) - ((__pyx_v_self->k_p_b * pow(__pyx_v_Ca2_ss, __pyx_v_self->m)) * __pyx_v_Po1)) + (__pyx_v_self->k_n_b * __pyx_v_Po2)) - (__pyx_v_self->k_p_c * __pyx_v_Po1)) + (__pyx_v_self->k_n_c * __pyx_v_Pc2));
  goto __pyx_L0;
```

```
 1424:
```

```
 1425:
```

```
 1426:     cdef double f_Po2(self,double Ca2_ss, double Po1, double Po2):
```

```
static double __pyx_f_5model_10Bondarenko_f_Po2(struct __pyx_obj_5model_Bondarenko *__pyx_v_self, double __pyx_v_Ca2_ss, double __pyx_v_Po1, double __pyx_v_Po2) {
  double __pyx_r;
  __Pyx_RefNannyDeclarations
  __Pyx_RefNannySetupContext("f_Po2", 0);
/* … */
  /* function exit code */
  __pyx_L0:;
  __Pyx_RefNannyFinishContext();
  return __pyx_r;
}
```

```
 1427:
```

```
 1428:             return self.k_p_b*Ca2_ss**self.m*Po1 - self.k_n_b*Po2
```

```
  __pyx_r = (((__pyx_v_self->k_p_b * pow(__pyx_v_Ca2_ss, __pyx_v_self->m)) * __pyx_v_Po1) - (__pyx_v_self->k_n_b * __pyx_v_Po2));
  goto __pyx_L0;
```

```
 1429:
```

```
 1430:
```

```
 1431:     cdef double f_Pc2(self,double Po1, double Pc2):
```

```
static double __pyx_f_5model_10Bondarenko_f_Pc2(struct __pyx_obj_5model_Bondarenko *__pyx_v_self, double __pyx_v_Po1, double __pyx_v_Pc2) {
  double __pyx_r;
  __Pyx_RefNannyDeclarations
  __Pyx_RefNannySetupContext("f_Pc2", 0);
/* … */
  /* function exit code */
  __pyx_L0:;
  __Pyx_RefNannyFinishContext();
  return __pyx_r;
}
```

```
 1432:
```

```
 1433:             return self.k_p_c*Po1 - self.k_n_c*Pc2
```

```
  __pyx_r = ((__pyx_v_self->k_p_c * __pyx_v_Po1) - (__pyx_v_self->k_n_c * __pyx_v_Pc2));
  goto __pyx_L0;
```

```
 1434:
```

```
 1435:
```

```
 1436:     cdef double f_O(self,double a, double c4, double B, double o, double I1, double g, double I2, double k_pcf):
```

```
static double __pyx_f_5model_10Bondarenko_f_O(struct __pyx_obj_5model_Bondarenko *__pyx_v_self, double __pyx_v_a, double __pyx_v_c4, double __pyx_v_B, double __pyx_v_o, double __pyx_v_I1, double __pyx_v_g, double __pyx_v_I2, double __pyx_v_k_pcf) {
  double __pyx_r;
  __Pyx_RefNannyDeclarations
  __Pyx_RefNannySetupContext("f_O", 0);
/* … */
  /* function exit code */
  __pyx_L0:;
  __Pyx_RefNannyFinishContext();
  return __pyx_r;
}
```

```
 1437:
```

```
 1438:             return a*c4 - 4*B*o + self.k_pcb*I1 - g*o + 0.001*(a*I2 - k_pcf*o)
```

```
  __pyx_r = (((((__pyx_v_a * __pyx_v_c4) - ((4.0 * __pyx_v_B) * __pyx_v_o)) + (__pyx_v_self->k_pcb * __pyx_v_I1)) - (__pyx_v_g * __pyx_v_o)) + (0.001 * ((__pyx_v_a * __pyx_v_I2) - (__pyx_v_k_pcf * __pyx_v_o))));
  goto __pyx_L0;
```

```
 1439:
```

```
 1440:
```

```
 1441:     cdef double f_C2(self,double c1, double c2, double c3, double a, double B):
```

```
static double __pyx_f_5model_10Bondarenko_f_C2(CYTHON_UNUSED struct __pyx_obj_5model_Bondarenko *__pyx_v_self, double __pyx_v_c1, double __pyx_v_c2, double __pyx_v_c3, double __pyx_v_a, double __pyx_v_B) {
  double __pyx_r;
  __Pyx_RefNannyDeclarations
  __Pyx_RefNannySetupContext("f_C2", 0);
/* … */
  /* function exit code */
  __pyx_L0:;
  __Pyx_RefNannyFinishContext();
  return __pyx_r;
}
```

```
 1442:
```

```
 1443:             return 4*a*c1 - B*c2 + 2*B*c3 - 3*a*c2
```

```
  __pyx_r = (((((4.0 * __pyx_v_a) * __pyx_v_c1) - (__pyx_v_B * __pyx_v_c2)) + ((2.0 * __pyx_v_B) * __pyx_v_c3)) - ((3.0 * __pyx_v_a) * __pyx_v_c2));
  goto __pyx_L0;
```

```
 1444:
```

```
 1445:
```

```
 1446:     cdef double f_C3(self,double c2, double c3, double c4, double a, double B):
```

```
static double __pyx_f_5model_10Bondarenko_f_C3(CYTHON_UNUSED struct __pyx_obj_5model_Bondarenko *__pyx_v_self, double __pyx_v_c2, double __pyx_v_c3, double __pyx_v_c4, double __pyx_v_a, double __pyx_v_B) {
  double __pyx_r;
  __Pyx_RefNannyDeclarations
  __Pyx_RefNannySetupContext("f_C3", 0);
/* … */
  /* function exit code */
  __pyx_L0:;
  __Pyx_RefNannyFinishContext();
  return __pyx_r;
}
```

```
 1447:
```

```
 1448:             return 3*a*c2 - 2*B*c3 + 3*B*c4 - 2*a*c3
```

```
  __pyx_r = (((((3.0 * __pyx_v_a) * __pyx_v_c2) - ((2.0 * __pyx_v_B) * __pyx_v_c3)) + ((3.0 * __pyx_v_B) * __pyx_v_c4)) - ((2.0 * __pyx_v_a) * __pyx_v_c3));
  goto __pyx_L0;
```

```
 1449:
```

```
 1450:
```

```
 1451:     cdef double f_C4(self,double c3, double c4, double o, double I1, double I2, double I3, double a, double B, double g, double k_pcf):
```

```
static double __pyx_f_5model_10Bondarenko_f_C4(struct __pyx_obj_5model_Bondarenko *__pyx_v_self, double __pyx_v_c3, double __pyx_v_c4, double __pyx_v_o, double __pyx_v_I1, double __pyx_v_I2, double __pyx_v_I3, double __pyx_v_a, double __pyx_v_B, double __pyx_v_g, double __pyx_v_k_pcf) {
  double __pyx_r;
  __Pyx_RefNannyDeclarations
  __Pyx_RefNannySetupContext("f_C4", 0);
/* … */
  /* function exit code */
  __pyx_L0:;
  __Pyx_RefNannyFinishContext();
  return __pyx_r;
}
```

```
 1452:
```

```
 1453:             return (2*a*c3 - 3*B*c4 + 4*B*o - a*c4 + 0.01*(4*self.k_pcb*B*I1 - a*g*c4) +
```

```
 1454:                     0.002*(4*B*I2 - k_pcf*c4) + 4*B*self.k_pcb*I3 - g*k_pcf*c4)
```

```
  __pyx_r = (((((((((2.0 * __pyx_v_a) * __pyx_v_c3) - ((3.0 * __pyx_v_B) * __pyx_v_c4)) + ((4.0 * __pyx_v_B) * __pyx_v_o)) - (__pyx_v_a * __pyx_v_c4)) + (0.01 * ((((4.0 * __pyx_v_self->k_pcb) * __pyx_v_B) * __pyx_v_I1) - ((__pyx_v_a * __pyx_v_g) * __pyx_v_c4)))) + (0.002 * (((4.0 * __pyx_v_B) * __pyx_v_I2) - (__pyx_v_k_pcf * __pyx_v_c4)))) + (((4.0 * __pyx_v_B) * __pyx_v_self->k_pcb) * __pyx_v_I3)) - ((__pyx_v_g * __pyx_v_k_pcf) * __pyx_v_c4));
  goto __pyx_L0;
```

```
 1455:
```

```
 1456:
```

```
 1457:     cdef double f_I1(self,double o, double I1, double I3, double c4, double a, double B, double g, double k_pcf):
```

```
static double __pyx_f_5model_10Bondarenko_f_I1(struct __pyx_obj_5model_Bondarenko *__pyx_v_self, double __pyx_v_o, double __pyx_v_I1, double __pyx_v_I3, double __pyx_v_c4, double __pyx_v_a, double __pyx_v_B, double __pyx_v_g, double __pyx_v_k_pcf) {
  double __pyx_r;
  __Pyx_RefNannyDeclarations
  __Pyx_RefNannySetupContext("f_I1", 0);
/* … */
  /* function exit code */
  __pyx_L0:;
  __Pyx_RefNannyFinishContext();
  return __pyx_r;
}
```

```
 1458:
```

```
 1459:             return g*o - self.k_pcb*I1 + 0.001*(a*I3 - k_pcf*I1) + 0.01*(a*g*c4 - 4*B*self.k_pcb*I1)
```

```
  __pyx_r = ((((__pyx_v_g * __pyx_v_o) - (__pyx_v_self->k_pcb * __pyx_v_I1)) + (0.001 * ((__pyx_v_a * __pyx_v_I3) - (__pyx_v_k_pcf * __pyx_v_I1)))) + (0.01 * (((__pyx_v_a * __pyx_v_g) * __pyx_v_c4) - (((4.0 * __pyx_v_B) * __pyx_v_self->k_pcb) * __pyx_v_I1))));
  goto __pyx_L0;
```

```
 1460:
```

```
 1461:
```

```
 1462:     cdef double f_I2(self,double o, double I2, double I3, double c4, double k_pcf, double a, double g, double B):
```

```
static double __pyx_f_5model_10Bondarenko_f_I2(struct __pyx_obj_5model_Bondarenko *__pyx_v_self, double __pyx_v_o, double __pyx_v_I2, double __pyx_v_I3, double __pyx_v_c4, double __pyx_v_k_pcf, double __pyx_v_a, double __pyx_v_g, double __pyx_v_B) {
  double __pyx_r;
  __Pyx_RefNannyDeclarations
  __Pyx_RefNannySetupContext("f_I2", 0);
/* … */
  /* function exit code */
  __pyx_L0:;
  __Pyx_RefNannyFinishContext();
  return __pyx_r;
}
```

```
 1463:
```

```
 1464:             return 0.001*(k_pcf*o - a*I2) + self.k_pcb*I3 - g*I2 + 0.002*(k_pcf*c4 - 4*B*I2)
```

```
  __pyx_r = ((((0.001 * ((__pyx_v_k_pcf * __pyx_v_o) - (__pyx_v_a * __pyx_v_I2))) + (__pyx_v_self->k_pcb * __pyx_v_I3)) - (__pyx_v_g * __pyx_v_I2)) + (0.002 * ((__pyx_v_k_pcf * __pyx_v_c4) - ((4.0 * __pyx_v_B) * __pyx_v_I2))));
  goto __pyx_L0;
```

```
 1465:
```

```
 1466:
```

```
 1467:     cdef double f_I3(self,double I1, double I2, double I3, double c4, double a, double B, double g, double k_pcf):
```

```
static double __pyx_f_5model_10Bondarenko_f_I3(struct __pyx_obj_5model_Bondarenko *__pyx_v_self, double __pyx_v_I1, double __pyx_v_I2, double __pyx_v_I3, double __pyx_v_c4, double __pyx_v_a, double __pyx_v_B, double __pyx_v_g, double __pyx_v_k_pcf) {
  double __pyx_r;
  __Pyx_RefNannyDeclarations
  __Pyx_RefNannySetupContext("f_I3", 0);
/* … */
  /* function exit code */
  __pyx_L0:;
  __Pyx_RefNannyFinishContext();
  return __pyx_r;
}
```

```
 1468:
```

```
 1469:             return 0.001*(k_pcf*I1 - a*I3) + g*I2 - self.k_pcb*I3 + g*k_pcf*c4 - 4*B*self.k_pcb*I3
```

```
  __pyx_r = (((((0.001 * ((__pyx_v_k_pcf * __pyx_v_I1) - (__pyx_v_a * __pyx_v_I3))) + (__pyx_v_g * __pyx_v_I2)) - (__pyx_v_self->k_pcb * __pyx_v_I3)) + ((__pyx_v_g * __pyx_v_k_pcf) * __pyx_v_c4)) - (((4.0 * __pyx_v_B) * __pyx_v_self->k_pcb) * __pyx_v_I3));
  goto __pyx_L0;
```

```
 1470:
```

```
 1471:     #Na concentration
```

```
 1472:
```

```
 1473:     cdef double f_Na_i (self,double I_Na, double I_Nab, double I_NaCa, double I_NaK):
```

```
static double __pyx_f_5model_10Bondarenko_f_Na_i(struct __pyx_obj_5model_Bondarenko *__pyx_v_self, double __pyx_v_I_Na, double __pyx_v_I_Nab, double __pyx_v_I_NaCa, double __pyx_v_I_NaK) {
  double __pyx_r;
  __Pyx_RefNannyDeclarations
  __Pyx_RefNannySetupContext("f_Na_i", 0);
/* … */
  /* function exit code */
  __pyx_L0:;
  __Pyx_RefNannyFinishContext();
  return __pyx_r;
}
```

```
 1474:
```

```
 1475:             return -(I_Na + I_Nab + 3*I_NaCa + 3*I_NaK )*((self.A_cap*self.C_m)/(self.V_myo*self.F))
```

```
  __pyx_r = ((-(((__pyx_v_I_Na + __pyx_v_I_Nab) + (3.0 * __pyx_v_I_NaCa)) + (3.0 * __pyx_v_I_NaK))) * ((__pyx_v_self->A_cap * __pyx_v_self->C_m) / (__pyx_v_self->V_myo * __pyx_v_self->F)));
  goto __pyx_L0;
```

```
 1476:
```

```
 1477:
```

```
 1478:     cdef double f_C_Na2 (self,double a_Na11, double C_Na3, double B_Na11, double C_Na2, double B_Na12, double a_Na12, double a_Na3, double IC_Na2, double B_Na3, double C_Na1):
```

```
static double __pyx_f_5model_10Bondarenko_f_C_Na2(CYTHON_UNUSED struct __pyx_obj_5model_Bondarenko *__pyx_v_self, double __pyx_v_a_Na11, double __pyx_v_C_Na3, double __pyx_v_B_Na11, double __pyx_v_C_Na2, double __pyx_v_B_Na12, double __pyx_v_a_Na12, double __pyx_v_a_Na3, double __pyx_v_IC_Na2, double __pyx_v_B_Na3, double __pyx_v_C_Na1) {
  double __pyx_r;
  __Pyx_RefNannyDeclarations
  __Pyx_RefNannySetupContext("f_C_Na2", 0);
/* … */
  /* function exit code */
  __pyx_L0:;
  __Pyx_RefNannyFinishContext();
  return __pyx_r;
}
```

```
 1479:
```

```
 1480:             return a_Na11*C_Na3 - B_Na11*C_Na2 + B_Na12*C_Na1 - a_Na12*C_Na2 + a_Na3*IC_Na2 - B_Na3*C_Na2
```

```
  __pyx_r = ((((((__pyx_v_a_Na11 * __pyx_v_C_Na3) - (__pyx_v_B_Na11 * __pyx_v_C_Na2)) + (__pyx_v_B_Na12 * __pyx_v_C_Na1)) - (__pyx_v_a_Na12 * __pyx_v_C_Na2)) + (__pyx_v_a_Na3 * __pyx_v_IC_Na2)) - (__pyx_v_B_Na3 * __pyx_v_C_Na2));
  goto __pyx_L0;
```

```
 1481:
```

```
 1482:
```

```
 1483:     cdef double f_C_Na1 (self,double a_Na12, double C_Na2, double B_Na12, double C_Na1, double B_Na13, double O_Na, double a_Na13, double a_Na3, double IF_Na, double B_Na3): #F_na? what is this?
```

```
static double __pyx_f_5model_10Bondarenko_f_C_Na1(CYTHON_UNUSED struct __pyx_obj_5model_Bondarenko *__pyx_v_self, double __pyx_v_a_Na12, double __pyx_v_C_Na2, double __pyx_v_B_Na12, double __pyx_v_C_Na1, double __pyx_v_B_Na13, double __pyx_v_O_Na, double __pyx_v_a_Na13, double __pyx_v_a_Na3, double __pyx_v_IF_Na, double __pyx_v_B_Na3) {
  double __pyx_r;
  __Pyx_RefNannyDeclarations
  __Pyx_RefNannySetupContext("f_C_Na1", 0);
/* … */
  /* function exit code */
  __pyx_L0:;
  __Pyx_RefNannyFinishContext();
  return __pyx_r;
}
```

```
 1484:
```

```
 1485:             return a_Na12*C_Na2 - B_Na12*C_Na1 + B_Na13*O_Na - a_Na13*C_Na1 + a_Na3*IF_Na - B_Na3*C_Na1
```

```
  __pyx_r = ((((((__pyx_v_a_Na12 * __pyx_v_C_Na2) - (__pyx_v_B_Na12 * __pyx_v_C_Na1)) + (__pyx_v_B_Na13 * __pyx_v_O_Na)) - (__pyx_v_a_Na13 * __pyx_v_C_Na1)) + (__pyx_v_a_Na3 * __pyx_v_IF_Na)) - (__pyx_v_B_Na3 * __pyx_v_C_Na1));
  goto __pyx_L0;
```

```
 1486:
```

```
 1487:
```

```
 1488:     cdef double f_O_Na (self,double a_Na13, double C_Na1, double B_Na13, double O_Na, double B_Na2, double IF_Na, double a_Na2):
```

```
static double __pyx_f_5model_10Bondarenko_f_O_Na(CYTHON_UNUSED struct __pyx_obj_5model_Bondarenko *__pyx_v_self, double __pyx_v_a_Na13, double __pyx_v_C_Na1, double __pyx_v_B_Na13, double __pyx_v_O_Na, double __pyx_v_B_Na2, double __pyx_v_IF_Na, double __pyx_v_a_Na2) {
  double __pyx_r;
  __Pyx_RefNannyDeclarations
  __Pyx_RefNannySetupContext("f_O_Na", 0);
/* … */
  /* function exit code */
  __pyx_L0:;
  __Pyx_RefNannyFinishContext();
  return __pyx_r;
}
```

```
 1489:
```

```
 1490:             return a_Na13*C_Na1 - B_Na13*O_Na + B_Na2*IF_Na - a_Na2*O_Na
```

```
  __pyx_r = ((((__pyx_v_a_Na13 * __pyx_v_C_Na1) - (__pyx_v_B_Na13 * __pyx_v_O_Na)) + (__pyx_v_B_Na2 * __pyx_v_IF_Na)) - (__pyx_v_a_Na2 * __pyx_v_O_Na));
  goto __pyx_L0;
```

```
 1491:
```

```
 1492:
```

```
 1493:     cdef double f_IF_Na (self,double a_Na2, double O_Na, double B_Na2, double IF_Na, double B_Na3, double C_Na1, double a_Na3, double B_Na4, double I1_Na, double a_Na4, double a_Na12, double IC_Na2, double B_Na12):
```

```
static double __pyx_f_5model_10Bondarenko_f_IF_Na(CYTHON_UNUSED struct __pyx_obj_5model_Bondarenko *__pyx_v_self, double __pyx_v_a_Na2, double __pyx_v_O_Na, double __pyx_v_B_Na2, double __pyx_v_IF_Na, double __pyx_v_B_Na3, double __pyx_v_C_Na1, double __pyx_v_a_Na3, double __pyx_v_B_Na4, double __pyx_v_I1_Na, double __pyx_v_a_Na4, double __pyx_v_a_Na12, double __pyx_v_IC_Na2, double __pyx_v_B_Na12) {
  double __pyx_r;
  __Pyx_RefNannyDeclarations
  __Pyx_RefNannySetupContext("f_IF_Na", 0);
/* … */
  /* function exit code */
  __pyx_L0:;
  __Pyx_RefNannyFinishContext();
  return __pyx_r;
}
```

```
 1494:
```

```
 1495:             return a_Na2*O_Na - B_Na2*IF_Na + B_Na3*C_Na1 - a_Na3*IF_Na + B_Na4*I1_Na - a_Na4*IF_Na + a_Na12*IC_Na2 - B_Na12*IF_Na
```

```
  __pyx_r = ((((((((__pyx_v_a_Na2 * __pyx_v_O_Na) - (__pyx_v_B_Na2 * __pyx_v_IF_Na)) + (__pyx_v_B_Na3 * __pyx_v_C_Na1)) - (__pyx_v_a_Na3 * __pyx_v_IF_Na)) + (__pyx_v_B_Na4 * __pyx_v_I1_Na)) - (__pyx_v_a_Na4 * __pyx_v_IF_Na)) + (__pyx_v_a_Na12 * __pyx_v_IC_Na2)) - (__pyx_v_B_Na12 * __pyx_v_IF_Na));
  goto __pyx_L0;
```

```
 1496:
```

```
 1497:
```

```
 1498:     cdef double f_I1_Na (self,double a_Na4, double IF_Na, double B_Na4, double I1_Na, double B_Na5, double I2_Na, double a_Na5):
```

```
static double __pyx_f_5model_10Bondarenko_f_I1_Na(CYTHON_UNUSED struct __pyx_obj_5model_Bondarenko *__pyx_v_self, double __pyx_v_a_Na4, double __pyx_v_IF_Na, double __pyx_v_B_Na4, double __pyx_v_I1_Na, double __pyx_v_B_Na5, double __pyx_v_I2_Na, double __pyx_v_a_Na5) {
  double __pyx_r;
  __Pyx_RefNannyDeclarations
  __Pyx_RefNannySetupContext("f_I1_Na", 0);
/* … */
  /* function exit code */
  __pyx_L0:;
  __Pyx_RefNannyFinishContext();
  return __pyx_r;
}
```

```
 1499:
```

```
 1500:             return a_Na4*IF_Na - B_Na4*I1_Na + B_Na5*I2_Na - a_Na5*I1_Na
```

```
  __pyx_r = ((((__pyx_v_a_Na4 * __pyx_v_IF_Na) - (__pyx_v_B_Na4 * __pyx_v_I1_Na)) + (__pyx_v_B_Na5 * __pyx_v_I2_Na)) - (__pyx_v_a_Na5 * __pyx_v_I1_Na));
  goto __pyx_L0;
```

```
 1501:
```

```
 1502:
```

```
 1503:     cdef double f_I2_Na (self,double a_Na5, double I1_Na, double B_Na5, double I2_Na):
```

```
static double __pyx_f_5model_10Bondarenko_f_I2_Na(CYTHON_UNUSED struct __pyx_obj_5model_Bondarenko *__pyx_v_self, double __pyx_v_a_Na5, double __pyx_v_I1_Na, double __pyx_v_B_Na5, double __pyx_v_I2_Na) {
  double __pyx_r;
  __Pyx_RefNannyDeclarations
  __Pyx_RefNannySetupContext("f_I2_Na", 0);
/* … */
  /* function exit code */
  __pyx_L0:;
  __Pyx_RefNannyFinishContext();
  return __pyx_r;
}
```

```
 1504:
```

```
 1505:             return a_Na5*I1_Na - B_Na5*I2_Na
```

```
  __pyx_r = ((__pyx_v_a_Na5 * __pyx_v_I1_Na) - (__pyx_v_B_Na5 * __pyx_v_I2_Na));
  goto __pyx_L0;
```

```
 1506:
```

```
 1507:
```

```
 1508:     cdef double f_IC_Na2 (self,double a_Na11, double IC_Na3, double B_Na11, double IC_Na2, double B_Na12, double IF_Na, double a_Na12, double B_Na3, double C_Na2, double a_Na3 ):
```

```
static double __pyx_f_5model_10Bondarenko_f_IC_Na2(CYTHON_UNUSED struct __pyx_obj_5model_Bondarenko *__pyx_v_self, double __pyx_v_a_Na11, double __pyx_v_IC_Na3, double __pyx_v_B_Na11, double __pyx_v_IC_Na2, double __pyx_v_B_Na12, double __pyx_v_IF_Na, double __pyx_v_a_Na12, double __pyx_v_B_Na3, double __pyx_v_C_Na2, double __pyx_v_a_Na3) {
  double __pyx_r;
  __Pyx_RefNannyDeclarations
  __Pyx_RefNannySetupContext("f_IC_Na2", 0);
/* … */
  /* function exit code */
  __pyx_L0:;
  __Pyx_RefNannyFinishContext();
  return __pyx_r;
}
```

```
 1509:
```

```
 1510:              return a_Na11*IC_Na3 - B_Na11*IC_Na2 + B_Na12*IF_Na - a_Na12*IC_Na2 + B_Na3*C_Na2 - a_Na3*IC_Na2
```

```
  __pyx_r = ((((((__pyx_v_a_Na11 * __pyx_v_IC_Na3) - (__pyx_v_B_Na11 * __pyx_v_IC_Na2)) + (__pyx_v_B_Na12 * __pyx_v_IF_Na)) - (__pyx_v_a_Na12 * __pyx_v_IC_Na2)) + (__pyx_v_B_Na3 * __pyx_v_C_Na2)) - (__pyx_v_a_Na3 * __pyx_v_IC_Na2));
  goto __pyx_L0;
```

```
 1511:
```

```
 1512:
```

```
 1513:     cdef double f_IC_Na3 (self,double B_Na11, double IC_Na2, double a_Na11, double IC_Na3, double B_Na3, double C_Na3, double a_Na3):
```

```
static double __pyx_f_5model_10Bondarenko_f_IC_Na3(CYTHON_UNUSED struct __pyx_obj_5model_Bondarenko *__pyx_v_self, double __pyx_v_B_Na11, double __pyx_v_IC_Na2, double __pyx_v_a_Na11, double __pyx_v_IC_Na3, double __pyx_v_B_Na3, double __pyx_v_C_Na3, double __pyx_v_a_Na3) {
  double __pyx_r;
  __Pyx_RefNannyDeclarations
  __Pyx_RefNannySetupContext("f_IC_Na3", 0);
/* … */
  /* function exit code */
  __pyx_L0:;
  __Pyx_RefNannyFinishContext();
  return __pyx_r;
}
```

```
 1514:
```

```
 1515:              return B_Na11*IC_Na2 - a_Na11*IC_Na3 + B_Na3*C_Na3 - a_Na3*IC_Na3
```

```
  __pyx_r = ((((__pyx_v_B_Na11 * __pyx_v_IC_Na2) - (__pyx_v_a_Na11 * __pyx_v_IC_Na3)) + (__pyx_v_B_Na3 * __pyx_v_C_Na3)) - (__pyx_v_a_Na3 * __pyx_v_IC_Na3));
  goto __pyx_L0;
```

```
 1516:
```

```
 1517:     #K concentration
```

```
 1518:     cdef double f_K_i (self,double I_Ktof, double I_Ktos, double I_K1, double I_Ks, double I_Kss, double I_Kur, double I_Kr, double I_NaK):
```

```
static double __pyx_f_5model_10Bondarenko_f_K_i(struct __pyx_obj_5model_Bondarenko *__pyx_v_self, double __pyx_v_I_Ktof, double __pyx_v_I_Ktos, double __pyx_v_I_K1, double __pyx_v_I_Ks, double __pyx_v_I_Kss, double __pyx_v_I_Kur, double __pyx_v_I_Kr, double __pyx_v_I_NaK) {
  double __pyx_r;
  __Pyx_RefNannyDeclarations
  __Pyx_RefNannySetupContext("f_K_i", 0);
/* … */
  /* function exit code */
  __pyx_L0:;
  __Pyx_RefNannyFinishContext();
  return __pyx_r;
}
```

```
 1519:
```

```
 1520:              return -(I_Ktof + I_Ktos + I_K1 + I_Ks + I_Kss + I_Kur + I_Kr - 2*I_NaK)*((self.A_cap*self.C_m)/(self.V_myo*self.F))
```

```
  __pyx_r = ((-(((((((__pyx_v_I_Ktof + __pyx_v_I_Ktos) + __pyx_v_I_K1) + __pyx_v_I_Ks) + __pyx_v_I_Kss) + __pyx_v_I_Kur) + __pyx_v_I_Kr) - (2.0 * __pyx_v_I_NaK))) * ((__pyx_v_self->A_cap * __pyx_v_self->C_m) / (__pyx_v_self->V_myo * __pyx_v_self->F)));
  goto __pyx_L0;
```

```
 1521:
```

```
 1522:     cdef double f_a_tof (self,double a_a, double a_tof, double B_a):
```

```
static double __pyx_f_5model_10Bondarenko_f_a_tof(CYTHON_UNUSED struct __pyx_obj_5model_Bondarenko *__pyx_v_self, double __pyx_v_a_a, double __pyx_v_a_tof, double __pyx_v_B_a) {
  double __pyx_r;
  __Pyx_RefNannyDeclarations
  __Pyx_RefNannySetupContext("f_a_tof", 0);
/* … */
  /* function exit code */
  __pyx_L0:;
  __Pyx_RefNannyFinishContext();
  return __pyx_r;
}
```

```
 1523:
```

```
 1524:              return a_a*(1 - a_tof) - B_a*a_tof
```

```
  __pyx_r = ((__pyx_v_a_a * (1.0 - __pyx_v_a_tof)) - (__pyx_v_B_a * __pyx_v_a_tof));
  goto __pyx_L0;
```

```
 1525:
```

```
 1526:
```

```
 1527:     cdef double f_i_tof (self,double a_i, double i_tof, double B_i_Na):
```

```
static double __pyx_f_5model_10Bondarenko_f_i_tof(CYTHON_UNUSED struct __pyx_obj_5model_Bondarenko *__pyx_v_self, double __pyx_v_a_i, double __pyx_v_i_tof, double __pyx_v_B_i_Na) {
  double __pyx_r;
  __Pyx_RefNannyDeclarations
  __Pyx_RefNannySetupContext("f_i_tof", 0);
/* … */
  /* function exit code */
  __pyx_L0:;
  __Pyx_RefNannyFinishContext();
  return __pyx_r;
}
```

```
 1528:
```

```
 1529:             return a_i*(1 - i_tof) - B_i_Na*i_tof
```

```
  __pyx_r = ((__pyx_v_a_i * (1.0 - __pyx_v_i_tof)) - (__pyx_v_B_i_Na * __pyx_v_i_tof));
  goto __pyx_L0;
```

```
 1530:
```

```
 1531:
```

```
 1532:     cdef double f_a_tos(self,double a_ss, double a_tos, double t_tas):
```

```
static double __pyx_f_5model_10Bondarenko_f_a_tos(CYTHON_UNUSED struct __pyx_obj_5model_Bondarenko *__pyx_v_self, double __pyx_v_a_ss, double __pyx_v_a_tos, double __pyx_v_t_tas) {
  double __pyx_r;
  __Pyx_RefNannyDeclarations
  __Pyx_RefNannySetupContext("f_a_tos", 0);
/* … */
  /* function exit code */
  __pyx_L0:;
  __Pyx_RefNannyFinishContext();
  return __pyx_r;
}
```

```
 1533:
```

```
 1534:             return (a_ss - a_tos)/t_tas
```

```
  __pyx_r = ((__pyx_v_a_ss - __pyx_v_a_tos) / __pyx_v_t_tas);
  goto __pyx_L0;
```

```
 1535:
```

```
 1536:
```

```
 1537:     cdef double f_i_tos(self,double i_ss, double i_tos, double t_tis):
```

```
static double __pyx_f_5model_10Bondarenko_f_i_tos(CYTHON_UNUSED struct __pyx_obj_5model_Bondarenko *__pyx_v_self, double __pyx_v_i_ss, double __pyx_v_i_tos, double __pyx_v_t_tis) {
  double __pyx_r;
  __Pyx_RefNannyDeclarations
  __Pyx_RefNannySetupContext("f_i_tos", 0);
/* … */
  /* function exit code */
  __pyx_L0:;
  __Pyx_RefNannyFinishContext();
  return __pyx_r;
}
```

```
 1538:
```

```
 1539:             return (i_ss - i_tos)/t_tis
```

```
  __pyx_r = ((__pyx_v_i_ss - __pyx_v_i_tos) / __pyx_v_t_tis);
  goto __pyx_L0;
```

```
 1540:
```

```
 1541:
```

```
 1542:     cdef double f_n_Ks (self,double a_n, double n_Ks, double B_n):
```

```
static double __pyx_f_5model_10Bondarenko_f_n_Ks(CYTHON_UNUSED struct __pyx_obj_5model_Bondarenko *__pyx_v_self, double __pyx_v_a_n, double __pyx_v_n_Ks, double __pyx_v_B_n) {
  double __pyx_r;
  __Pyx_RefNannyDeclarations
  __Pyx_RefNannySetupContext("f_n_Ks", 0);
/* … */
  /* function exit code */
  __pyx_L0:;
  __Pyx_RefNannyFinishContext();
  return __pyx_r;
}
```

```
 1543:
```

```
 1544:             return a_n*(1 - n_Ks) - B_n*n_Ks
```

```
  __pyx_r = ((__pyx_v_a_n * (1.0 - __pyx_v_n_Ks)) - (__pyx_v_B_n * __pyx_v_n_Ks));
  goto __pyx_L0;
```

```
 1545:
```

```
 1546:
```

```
 1547:     cdef double f_a_ur (self,double a_ss, double a_ur, double t_aur):
```

```
static double __pyx_f_5model_10Bondarenko_f_a_ur(CYTHON_UNUSED struct __pyx_obj_5model_Bondarenko *__pyx_v_self, double __pyx_v_a_ss, double __pyx_v_a_ur, double __pyx_v_t_aur) {
  double __pyx_r;
  __Pyx_RefNannyDeclarations
  __Pyx_RefNannySetupContext("f_a_ur", 0);
/* … */
  /* function exit code */
  __pyx_L0:;
  __Pyx_RefNannyFinishContext();
  return __pyx_r;
}
```

```
 1548:
```

```
 1549:             return (a_ss - a_ur)/t_aur
```

```
  __pyx_r = ((__pyx_v_a_ss - __pyx_v_a_ur) / __pyx_v_t_aur);
  goto __pyx_L0;
```

```
 1550:
```

```
 1551:
```

```
 1552:     cdef double f_i_ur (self,double i_ss, double i_ur, double t_iur):
```

```
static double __pyx_f_5model_10Bondarenko_f_i_ur(CYTHON_UNUSED struct __pyx_obj_5model_Bondarenko *__pyx_v_self, double __pyx_v_i_ss, double __pyx_v_i_ur, double __pyx_v_t_iur) {
  double __pyx_r;
  __Pyx_RefNannyDeclarations
  __Pyx_RefNannySetupContext("f_i_ur", 0);
/* … */
  /* function exit code */
  __pyx_L0:;
  __Pyx_RefNannyFinishContext();
  return __pyx_r;
}
```

```
 1553:
```

```
 1554:             return (i_ss - i_ur)/t_iur
```

```
  __pyx_r = ((__pyx_v_i_ss - __pyx_v_i_ur) / __pyx_v_t_iur);
  goto __pyx_L0;
```

```
 1555:
```

```
 1556:
```

```
 1557:     cdef double f_a_Kss (self,double a_ss, double a_Kss, double t_Kss):
```

```
static double __pyx_f_5model_10Bondarenko_f_a_Kss(CYTHON_UNUSED struct __pyx_obj_5model_Bondarenko *__pyx_v_self, double __pyx_v_a_ss, double __pyx_v_a_Kss, double __pyx_v_t_Kss) {
  double __pyx_r;
  __Pyx_RefNannyDeclarations
  __Pyx_RefNannySetupContext("f_a_Kss", 0);
/* … */
  /* function exit code */
  __pyx_L0:;
  __Pyx_RefNannyFinishContext();
  return __pyx_r;
}
```

```
 1558:
```

```
 1559:             return (a_ss - a_Kss)/t_Kss
```

```
  __pyx_r = ((__pyx_v_a_ss - __pyx_v_a_Kss) / __pyx_v_t_Kss);
  goto __pyx_L0;
```

```
 1560:
```

```
 1561:
```

```
 1562:     cdef double f_C_K1 (self,double a_a0, double C_K0, double B_a0, double C_K1, double C_K2):
```

```
static double __pyx_f_5model_10Bondarenko_f_C_K1(struct __pyx_obj_5model_Bondarenko *__pyx_v_self, double __pyx_v_a_a0, double __pyx_v_C_K0, double __pyx_v_B_a0, double __pyx_v_C_K1, double __pyx_v_C_K2) {
  double __pyx_r;
  __Pyx_RefNannyDeclarations
  __Pyx_RefNannySetupContext("f_C_K1", 0);
/* … */
  /* function exit code */
  __pyx_L0:;
  __Pyx_RefNannyFinishContext();
  return __pyx_r;
}
```

```
 1563:
```

```
 1564:             return a_a0*C_K0 - B_a0*C_K1 + self.k_b*C_K2 - self.k_f*C_K1
```

```
  __pyx_r = ((((__pyx_v_a_a0 * __pyx_v_C_K0) - (__pyx_v_B_a0 * __pyx_v_C_K1)) + (__pyx_v_self->k_b * __pyx_v_C_K2)) - (__pyx_v_self->k_f * __pyx_v_C_K1));
  goto __pyx_L0;
```

```
 1565:
```

```
 1566:
```

```
 1567:     cdef double f_C_K2 (self,double C_K1, double C_K2, double B_a1, double O_K, double a_a1):
```

```
static double __pyx_f_5model_10Bondarenko_f_C_K2(struct __pyx_obj_5model_Bondarenko *__pyx_v_self, double __pyx_v_C_K1, double __pyx_v_C_K2, double __pyx_v_B_a1, double __pyx_v_O_K, double __pyx_v_a_a1) {
  double __pyx_r;
  __Pyx_RefNannyDeclarations
  __Pyx_RefNannySetupContext("f_C_K2", 0);
/* … */
  /* function exit code */
  __pyx_L0:;
  __Pyx_RefNannyFinishContext();
  return __pyx_r;
}
```

```
 1568:
```

```
 1569:             return self.k_f*C_K1 - self.k_b*C_K2 + B_a1*O_K - a_a1*C_K2
```

```
  __pyx_r = ((((__pyx_v_self->k_f * __pyx_v_C_K1) - (__pyx_v_self->k_b * __pyx_v_C_K2)) + (__pyx_v_B_a1 * __pyx_v_O_K)) - (__pyx_v_a_a1 * __pyx_v_C_K2));
  goto __pyx_L0;
```

```
 1570:
```

```
 1571:
```

```
 1572:     cdef double f_O_K (self,double a_a1, double C_K2, double B_a1, double O_K, double B_i_r, double I_K, double a_i_r):
```

```
static double __pyx_f_5model_10Bondarenko_f_O_K(CYTHON_UNUSED struct __pyx_obj_5model_Bondarenko *__pyx_v_self, double __pyx_v_a_a1, double __pyx_v_C_K2, double __pyx_v_B_a1, double __pyx_v_O_K, double __pyx_v_B_i_r, double __pyx_v_I_K, double __pyx_v_a_i_r) {
  double __pyx_r;
  __Pyx_RefNannyDeclarations
  __Pyx_RefNannySetupContext("f_O_K", 0);
/* … */
  /* function exit code */
  __pyx_L0:;
  __Pyx_RefNannyFinishContext();
  return __pyx_r;
}
```

```
 1573:
```

```
 1574:             return (a_a1*C_K2 - B_a1*O_K + B_i_r*I_K - a_i_r*O_K)
```

```
  __pyx_r = ((((__pyx_v_a_a1 * __pyx_v_C_K2) - (__pyx_v_B_a1 * __pyx_v_O_K)) + (__pyx_v_B_i_r * __pyx_v_I_K)) - (__pyx_v_a_i_r * __pyx_v_O_K));
  goto __pyx_L0;
```

```
 1575:
```

```
 1576:
```

```
 1577:     cdef double f_I_K (self, double a_i_r, double O_K, double B_i_r, double I_K):
```

```
static double __pyx_f_5model_10Bondarenko_f_I_K(CYTHON_UNUSED struct __pyx_obj_5model_Bondarenko *__pyx_v_self, double __pyx_v_a_i_r, double __pyx_v_O_K, double __pyx_v_B_i_r, double __pyx_v_I_K) {
  double __pyx_r;
  __Pyx_RefNannyDeclarations
  __Pyx_RefNannySetupContext("f_I_K", 0);
/* … */
  /* function exit code */
  __pyx_L0:;
  __Pyx_RefNannyFinishContext();
  return __pyx_r;
}
```

```
 1578:
```

```
 1579:             return a_i_r*O_K - B_i_r*I_K
```

```
  __pyx_r = ((__pyx_v_a_i_r * __pyx_v_O_K) - (__pyx_v_B_i_r * __pyx_v_I_K));
  goto __pyx_L0;
```

```
 1580:
```

```
 1581:
```

```
 1582:
```
